# A machine learning-based workflow for transaminase selection

**DOI:** 10.1039/d6sc00852f

**Published:** 2026-05-20

**Authors:** Alexander J. Rago, Priyanka Raghavan, Lisandra Santiago-Capeles, Ruijie Zhang, Ying Wang, Connor W. Coley

**Affiliations:** a Small Molecule Chemistry Technologies, AbbVie, Inc. 1 N Waukegan Rd North Chicago IL 60064 USA alex.rago@abbvie.com wang.ying@abbvie.com; b Department of Chemical Engineering, Massachusetts Institute of Technology 77 Massachusetts Ave Cambridge Massachusetts 02139 USA ccoley@mit.edu

## Abstract

Transaminases can be strategically used in medicinal chemistry for the synthesis of chiral amine building blocks in a straightforward manner that alleviates the need for chiral preparative separations. However, selection of the commercial transaminase variant to use for a given substrate can be a challenging task without prior knowledge or experience with their reactivity. Herein, we describe the construction of a dataset of 336 transaminase reactions using high-throughput experimentation (HTE) and the subsequent development of machine learning (ML) models to predict enzyme activities and selectivities. Our results demonstrate the ability of these models to predict substrate conversions and top-*k* enzyme selection for selectivity more accurately than baseline experiments. Finally, we demonstrate prospective use of the modeling workflow to select the transaminase to use for a held-out dataset of cyclic ketones, and the preparation of a small set of chiral amines of high-relevance to medicinal chemistry.

## Abstract 2

Predictive ML models enable the rank-ordering of transaminase enzymes for a given substrate based on their predicted reactivities, thus expediting enzyme selection for chiral amine synthesis in medicinal chemistry research.

## Graphical abstract

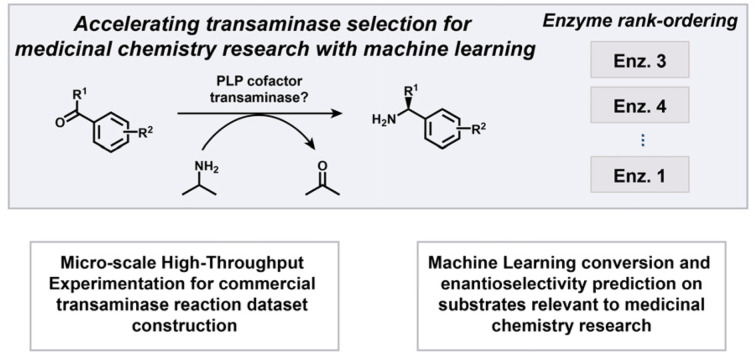

## Introduction

The accessibility to a variety of building blocks is essential to enable successful Structure–Activity Relationship (SAR) studies for medicinal chemistry programs.^1–3^ Many efforts have been dedicated towards identifying, sourcing, and sharing^4^ new building blocks for medicinal chemistry research; despite these efforts, the presence of chirality can still introduce challenges into the drug discovery process.^5,6^ Chiral building blocks are not always readily available in single enantiomer forms, introducing potential roadblocks to SAR studies as chemists seek methods to reliably access them. Biocatalysis can be a useful chemistry technology platform for addressing this gap,^7^ although surveying the literature suggests that it is more broadly used across process development applications.^8,9^ Despite challenges that can introduce barriers for the use of biocatalysis in medicinal chemistry,^10^ there exist literature reports of its use for specific needs including metabolite^11^ and novel building block synthesis.^12^

**Fig. 1:**
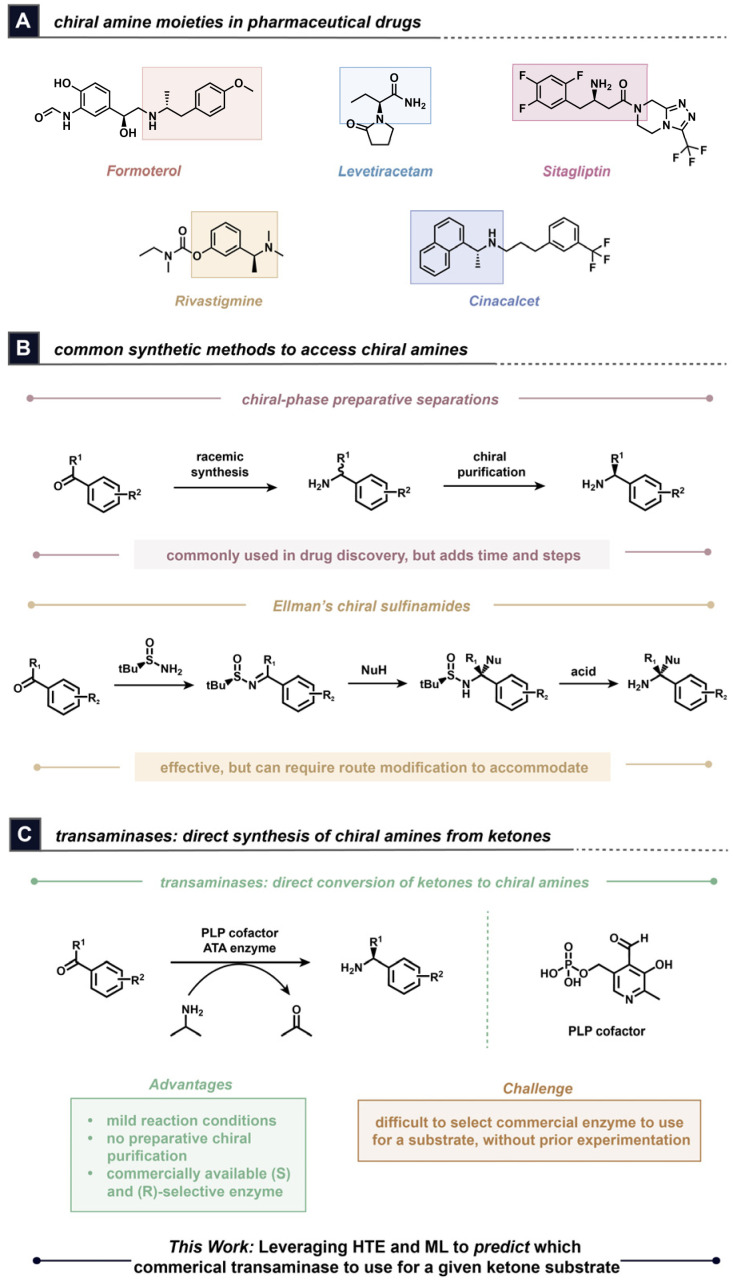
(A) The importance of chiral amine building blocks in drug discovery; (B) conventional methods to access chiral amines compared to transaminases; (C) this work: a ML-based workflow for transaminase selection.

Chiral amines represent a highly prevalent class of functional groups found in pharmaceutical drugs (Fig. 1a). Synthetic approaches to achieve the synthesis of chiral functional groups in high enantiopurity have historically involved the use of preparative chiral purifications^13^ or chiral auxiliaries,^14,15^ which can introduce added time, lost material, expenses, and synthetic steps to the Design-Make-Test-Analyse (DMTA) cycle of medicinal chemistry research (Fig. 1b). Catalytic methods have also been developed for chiral amine synthesis,^16^ such as asymmetric enamine hydrogenation,^17^ although these catalytic approaches can require the introduction/removal of protecting groups, may not allow direct access to 1° amines, and can potentially introduce other functional group incompatibilities. Biocatalysis is uniquely positioned to deliver these chiral building blocks in an efficient manner. Transaminases,^18^ imine reductases,^19^ amine dehydrogenases,^20^ and reductive aminases^19,21^ represent enzymes that can prepare chiral amines from ketone or imine precursors and have the potential to be useful for preparing these building blocks for drug discovery campaigns.

Transaminases are PLP-dependent enzymes that can enable access to chiral primary amine building blocks from readily available prochiral ketone precursors, oftentimes with high conversion and enantioselectivity (Fig. 1c).^22^ The required amine donor is transformed into a ketone co-product during the reaction. These enzymes have previously demonstrated effectiveness for chiral amine synthesis in medicinal chemistry endeavours at AbbVie, as well.^23^ Compared to other methods that furnish chiral primary amines with high enantioselectivity, transaminases offer mild reaction conditions and the potential to access either the (*S*) or the (*R*) enantiomer depending on the enzyme variant. Many studies in the literature involving transaminases have focused on the evolution and discovery of new transaminases to address a challenge with substrate activity, such as the collaboration between Merck and Codexis to evolve a new transaminase variant towards the synthesis of sitagliptin.^24^ The availability of commercial enzymes presents a streamlined opportunity to leverage transaminases for medicinal chemistry research without additional enzyme engineering efforts. Such enzymes have been leveraged to develop processes including a hybrid organo- and biocatalytic conversion of alcohols into chiral amines^25^ and have been used to achieve the transamination of bulky ketone substrates.^26^ However, it can oftentimes be unclear which commercial transaminase(s) should be chosen for a particular ketone substrate without prior experimentation. A key limitation of biocatalysis is scarcity of known enzyme–substrate pairs in databases and the difficulty of identifying patterns for specific enzymes that would extend their applicability to unreported, medicinally-interesting substrates. Databases such as RetroBioCat^27^ have begun to address these gaps, but generally rely on sparse literature data for biocatalytic reaction data curation. Furthermore, the rapid pace at which medicinal chemistry programs progress makes it important to use commercially available enzymes when leveraging biocatalysis to enable faster turnaround times. Therefore, the development of workflows that can enable the quick identification of an effective enzyme for a substrate would represent a significant advance in this space, while also reducing the time and amount of material spent on screening efforts.

For transaminases, interactions between substrates and the active sites of specific enzymes have been previously modelled with docking and molecular dynamics (MD) with the goal of predicting enantioselectivities.^28^ This work was later expanded to leverage a convolutional neural network to classify MD trajectories as leading to “reactive” or “non-reactive” outcomes.^29^ Meanwhile, EHReact has been developed by Green and co-workers as a general tool to predict the activity of an enzyme on a new substrate through the use of extended Hasse diagrams.^30^ More recently, the Narayan and Gomes groups have developed CATNIP, an ML-based tool to navigate between protein sequence space and chemical space that can be used to produce a list of enzymes that are predicted to be effective for a substrate.^31^ However, many commercial transaminases do not have information regarding their structures or even primary sequences readily available.

Our prior analysis of models designed to predict enzyme–substrate compatibility (as a special case of compound–protein interaction prediction) suggested that models do not truly learn interactions in a sequence-dependent manner that enables generalization to novel combinations.^32^ The practical consequence is that the inclusion of sequence information into enzyme–substrate models may not provide an advantage over models that do not make use of sequence information when making predictions on unseen substrates; therefore, the absence of sequence information for commercial transaminases in principle need not be a hindrance to modelling.

In this work, we detail our efforts to construct a dataset of 336 micro-scale transaminase reactions using a diverse set of 42 ketone substrates with 8 commercially available transaminases, along with the development of machine learning (ML) models to predict substrate conversion and enantioselectivity. We describe a workflow that predicts reaction success and rank-orders the enzymes based on their predicted enantioselectivities, then apply this workflow towards a held-out set of substrates. Finally, we demonstrate prospective use of the modelling workflow to suggest the commercial enzyme to use for the synthesis of a small number of chiral amine building blocks found in pharmaceutical drugs and bioactive molecules.

## Modelling approach and details

### Dataset generation

We began our dataset generation efforts by selecting 8 commercial transaminases commonly used at AbbVie,^33^ four of which are typically selective for the formation of the (*S*)-enantiomer of the amine product and the others exhibiting typical selectivity for the (*R*)-enantiomer (Table 1).^34^

**Table 1:** Commercial transaminases included in this study

Enzyme name	Typical selectivity	Vendor
ATA-026	*S*	Prozomix
ATA-031	*S*	Prozomix
ATA-237	*S*	Codexis
ATA-S125	*S*	EnzymeWorks
ATA-P2-A07	*R*	Codexis
ATA-025	*R*	Codexis
ATA-415	*R*	Codexis
ATA-R123	*R*	EnzymeWorks

**Fig. 2:**
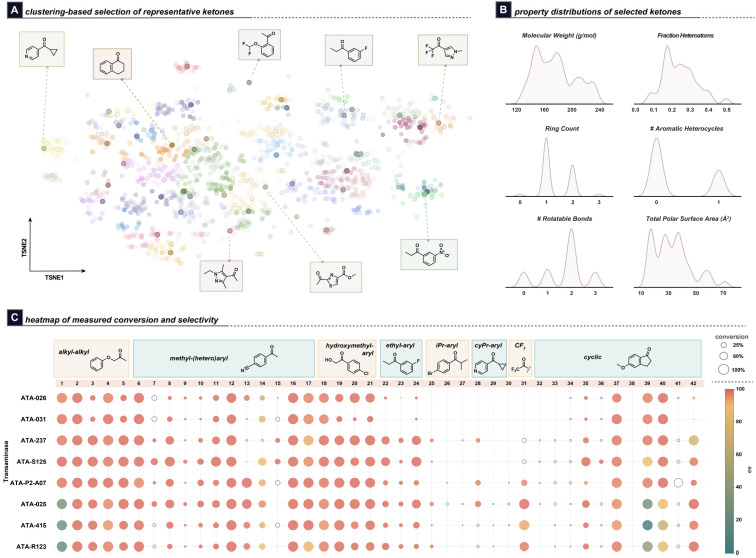
Characterization of the transaminase dataset showing (A) tSNE of the selected substrates from the larger pool of known/commercial ketones. The darker points, a few of which are explicitly shown in callouts, indicate the 42 experimentally-selected substrates; (B) distributions of substrate structural and physicochemical properties; (C) heatmap showing results from the HTE experiments. One representative ketone from each class is shown. The size of the points represents conversion, while the color represents selectivity. Hollow uncolored points represent reactions where the ee measurements could not be obtained due to low conversions, weak UV signals, *etc.* Note that the full grid of reactions was experimentally conducted; blank points indicate 0 or trace conversion. Reaction conditions: ketone (7.5 µmol; 500 mM in DMSO), transaminase (10 mg mL^−1^), PLP (0.5 mg mL^−1^), pH = 7.6 150 mM KPi buffer with 5% *v*/*v i*PrNH_2_ (25 mM), 35 °C, 24 h.

A diverse array of 42 substrates was selected for experimentation using an internal molecular diversity analysis tool that used a physiochemical property-based Pipeline Pilot clustering protocol to visualize and select substrates from an initial collection of 3.1k commercial, in-stock, or otherwise known prochiral ketones of several sub-types (Fig. 2a). The commercial availability of both the ketones and racemic amine products was taken into consideration when selecting the substrates to streamline SFC racemic standard preparation. These substrates represent several subtypes of prochiral ketones: alkyl–alkyl, alkyl–aryl, hydroxymethyl-aryl, trifluoromethyl, and cyclic ketones including indanones, tetralones, and other 5- and 6-membered ring systems. Further, they exhibit a range of physicochemical properties potentially of interest in building block set design (Fig. 2b; see SI and S16 for substrate structures).

The micro-scale biocatalytic HTE was conducted using isopropylamine as the amine donor since it was previously identified to be among the most general of several amine donors in a head-to-head study conducted by AbbVie process chemists using commercial transaminases.^33^ Furthermore, the resulting acetone co-product can evaporate to drive the reaction equilibrium towards product formation, simplifying reaction setup and analysis of the crude reactions compared to using methyl benzylamine or other amine donors that have a higher molecular weight, contain a chromophore, or need to be recycled by a second enzymatic system.

Crude reaction mixtures were analysed to determine reaction performance. The UV conversion of ketone to amine product was used to measure enzyme activity.^35^ Analysis of a sample set subjected to experimentation multiple times revealed generally low variation in the measured conversion values (see SI, Table S7). Meanwhile, ee values were determined using chiral analytical Supercritical Fluid Chromatography (SFC). The amine products were derivatized to simplify ee measurements. The amine derivatization methods used included conversion to hydroxypyridyl imines,^36^ Boc-protection, and Cbz-protection, although the amine ee was directly measured for a small number of substrates. A comparison between a derivatized and underivatized sample set found generally <1% difference in the measured ee, with the exception of one poorly-selective enzyme (see SI, Table S4). Furthermore, triplicate injection of an SFC sample set revealed generally small variation in the measured ee values (see SI, Table S5).

The overall results from these experiments are shown in Fig. 2c. Generally, enzyme activity decreases for more sterically hindered substrates, while enantioselectivity is generally high for many substrates. However, high substrate conversion is not always indicative of high enantioselectivity as observed with some alkyl–alkyl (Substrate 1), aryl-alkyl (Substrate 14), and cyclic (Substrate 39) substrates (Fig. 2). Furthermore, ee measurements could not be obtained for every reaction, as those with lower conversions presented challenges with accurately obtaining the ee data from the crude reaction mixtures. An additional dataset of 24 cyclic ketones was constructed in a similar manner, using k-means clustering to identify 24 substrates from a pool of ∼500 cyclic ketones, to use as a held-out dataset for evaluation of the models' performance.

### Machine learning modelling

**Fig. 3:**
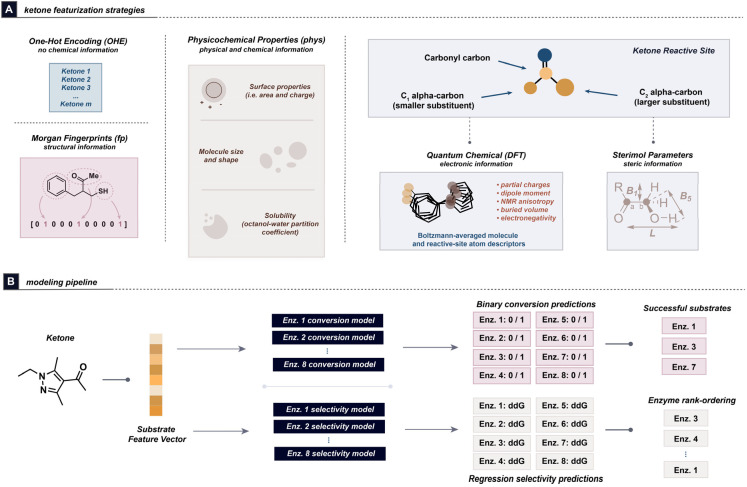
Modeling approach used to predict conversion and enantioselectivity for substrates, showing (A) the features investigated and (B) the modelling approach for this dataset. Individual models were trained for each of the 8 enzymes, and the results were taken together for model performance evaluation. As sequence (or structure) information is unavailable for the eight commercial enzymes, each is treated as a distinct single-task modeling objective.

The modelling of this dataset was approached with a similar featurization strategy compared to a previous study from our collaboration.^37^ Five sets of substrate features were investigated and compared against one another: One-Hot Encoding (OHE), Morgan Fingerprints (fp), RDKit^38^ physicochemical descriptors (phys), atom- and molecule-level quantum mechanical features (DFT), and the combination of physicochemical and DFT features (phys + DFT). QM features were obtained from Gaussian 16 software^39^ and were combined into Boltzmann-averaged distributions from conformational ensembles of up to 20 conformers per substrate. All DFT workflows were performed at the B3LYP/6-31G* level of theory. DFT features for the overall ketone molecules, as well as atom-level features of the reaction site (carbonyl and both *alpha*-carbonyl carbons), were extracted and used for modelling with the Auto-QChem pipeline.^40^ We additionally calculated Boltzmann-averaged Sterimol parameters^41^ of substituents down the two carbonyl-*alpha* carbon bonds. To maintain consistency in featurization across different ketones, the *alpha* carbons were designated as “large” or “small” based on their relative calculated buried volumes (*V*_bur_) (Fig. 3a).

Since the sequences of the transaminases used are not available, models were built from conversion and enantioselectivity data for each individual enzyme using a leave-one-out cross-validation (LOOCV) approach on the ketones. For medicinal chemistry research, a binary classification model can be sufficient for predicting whether enough of the target compounds can be obtained from a reaction.^37,42,43^ As such, binary Random Forest classification models using the aforementioned features were investigated for conversion, while regression was used for modelling enantioselectivity (Fig. 3b).^44^ A success threshold of 25% conversion was chosen, as this represents the point at which we consider the reaction to be useful for medicinal chemistry research, and for which the reaction is expected to reliably yield enough product for ee determination. Furthermore, a reaction can be further optimized from this point by adding additional enzyme, co-solvent, amine donor, increasing temperature/reaction time, changing the buffer composition and/or pH, *etc.* Any reactions where the ee could not be measured were dropped from the training dataset prior to conducting enantioselectivity modelling.

The distribution of ee values in this dataset skews towards the high end, with >88% of the measured ee values being at least 95%. Despite the seemingly high success rate of our dataset in terms of enantioselectivity, it is important to note that obtaining compounds with >99% ee can be necessary depending on the stage of a medicinal chemistry program. This emphasizes the importance of identifying the enzyme(s) that can afford the highest possible ee for a given substrate. To obtain more evenly-distributed selectivity values and align with best practices in the literature, ee values were transformed into ΔΔ*G*^‡^ values^44,45^ prior to model input (see SI, Section 2B). Additionally, alongside the Random Forest models, forward Multi-Variate Linear Regression (MVLR) algorithms using DFT and Sterimol features were investigated for modelling enantioselectivity (MVLR-DFT). These models provide the opportunity to actively select features that improve the model, and have successfully been applied by the Sigman group in numerous low-data selectivity tasks.^46–48^ The MVLR-DFT models initially begin with the highest-correlated feature and continue adding additional features to the model until the LOOCV *R*^2^ value decreases. For ΔΔ*G*^‡^ modelling, this typically resulted in no more than 9 features being selected for an enzyme's model.

As an evaluation metric, we use the top-*k* accuracy score obtained after rank-ordering the predicted ΔΔ*G*^‡^ values for a given substrate to assess the ΔΔ*G*^‡^ modelling performance. This was accomplished by comparing the top-*k* predicted enzyme identities to the actual top enzyme (or multiple top enzymes in the case of ties, see SI, Section 2f for details on scoring). Furthermore, as a predicted enzyme may still achieve a similar ee regardless of whether it is the best observed experimentally, we report a “regret” metric defined as the difference in selectivity experimentally obtained by the top model-ranked enzyme compared to the highest selectivity experimentally obtained for that substrate. The regret metric most closely reflects our primary goal of selecting a highly selective enzyme for each substrate given the fixed list of candidate transaminases.

### Workflow benchmarking experiments

Benchmarking the *connected* two-stage workflow was accomplished using experimental data generated from 24 additional cyclic substrates not included in the initial training dataset, for a total of 192 enzymatic reactions. Cyclic substrates represented about one-fourth of the original training data and exhibited a wide range of conversions and ee values (Fig. 2c). Here, we use the per-enzyme features selected by the best conversion and enantioselectivity models obtained from the initial modelling to test models on the held-out set.

The overall workflow is comprised of two parts: (1) binary prediction of whether the conversion for a given substrate will be greater than 25% for each enzyme and (2) prediction of the best-selective enzyme variant through ΔΔ*G*^‡^ regression prediction. For retrospective model building on the HTE dataset, we consider these two steps separately. In practice, a prediction can be considered optimal if the selected enzyme furnished >25% conversion with an ee regret of <1%. In addition to the regret, as described before, the maximum possible regret for each substrate (the difference between the highest and lowest experimentally-observed ee values) can be used as a comparison to benchmark the selection process. Substrates with higher maximum possible regret values represent those with the highest potential experimental loss from an incorrect model prediction.

### Prospective workflow validation experiments

The *connected* two-stage workflow was also applied in a prospective setting to prepare chiral amines representative of those present in existing pharmaceutical drug and bioactive molecules. For these experiments, only the enzyme selected by the workflow was chosen for experimental validation. We additionally assumed that the typical *S* or *R* selectivity of the transaminases applied to these substrates and only selected among enzymes that would furnish the enantiomer present in the active pharmaceutical ingredients. These reactions were conducted at a larger scale and were derivatized and subsequently purified to reflect how the models would be used in a laboratory setting for medicinal chemistry research.

## Results and discussion

### Model development

**Fig. 4:**
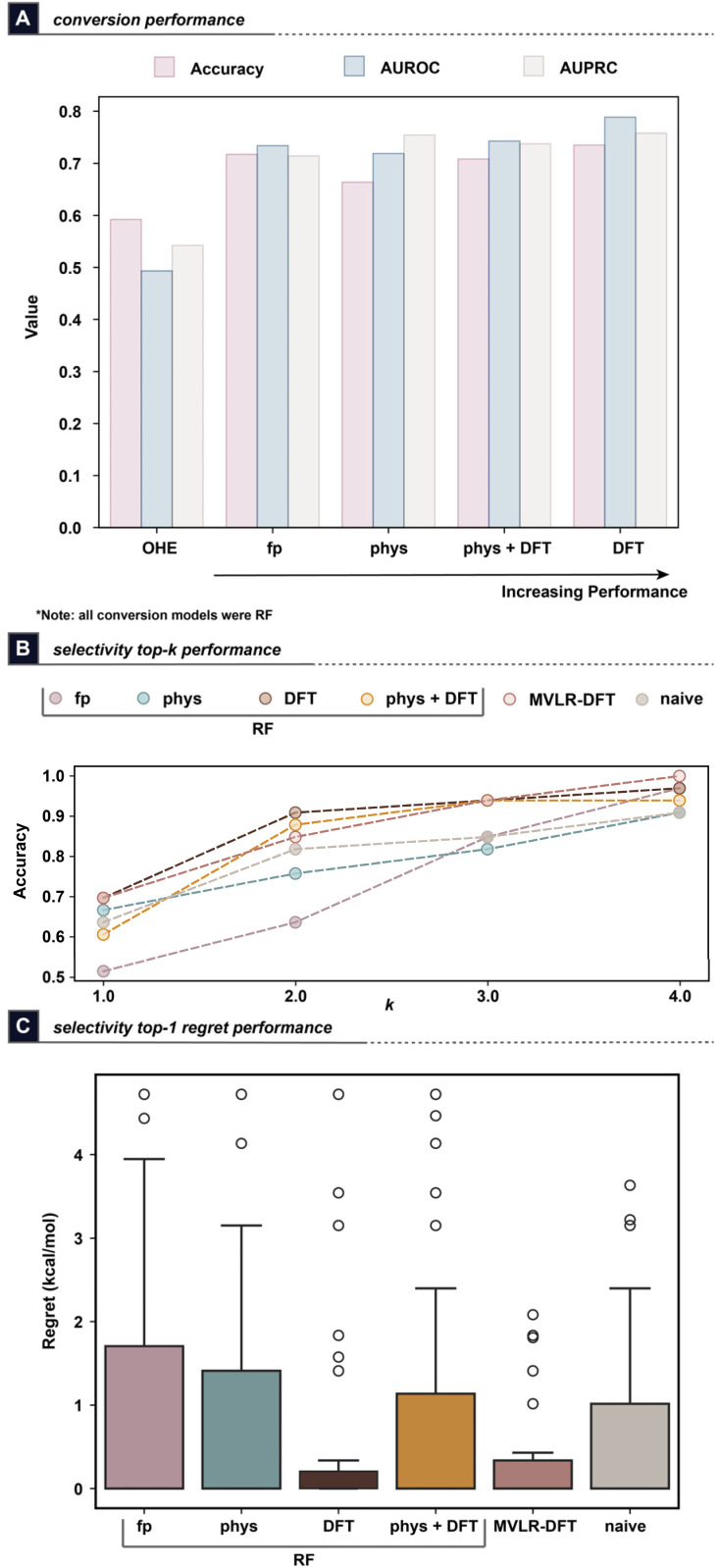
Results from modelling (A) conversion and (B and C) enantioselectivity (represented as ΔΔ*G*^‡^ to the models). Results for the ΔΔ*G*^‡^ modelling are depicted as (B) top-*k* accuracy for correctly selecting the top-*k* performing enzymes in terms of measured ee, and (C) top-1 regret in terms of ΔΔ*G*^‡^. Note that all results represent LOOCV metrics.

The results from modelling the initial dataset of 42 substrates exhibited promising performance for predicting both conversion and enantioselectivity (Fig. 4). As expected, the OHE models just slightly outperformed the positive rate of 56% and achieved an AUROC of only 0.49. Meanwhile, the feature-based models showed increasing levels of accuracy with the DFT-based models exhibiting the highest performance with an AUROC of 0.79 and 73.5% accuracy. Interestingly, fingerprint- and physicochemical property-based models exhibited similar overall performance to each other, while the inclusion of DFT features with the latter only resulted in a small improvement in overall accuracy (Fig. 4a). Analysis of the feature importance of the DFT conversion models revealed that features describing the polarization, charge, and steric-type features of the “smaller” carbonyl alpha-position were found to be common among the most-important features for each enzyme's models (see SI, Section 2g). As such, the DFT-based RF model was selected for future experiments modelling conversion.

A total of 233 ee measurements were used to train and evaluate selectivity models for this dataset, representing nearly 70% of all micro-scale enzymatic HTE reactions conducted for this exercise. Since each enzyme was modelled independently of the others, this resulted in 26–32 ee measurements being used to train and evaluate each of these models. As stated previously, the resulting ee values were transformed into ΔΔ*G*^‡^ values to improve the distribution of the highly positive-skewed ee values in this dataset (see SI, Fig. S1–S3 for histograms of the target properties). Similar trends in performance with respect to featurization were observed to those obtained in the conversion modelling, though the DFT models achieved a low *R*^2^ value of 0.066 for predicting ΔΔ*G*^‡^. Gratifyingly, the MVLR models using DFT and Sterimol features could achieve superior performance with an overall *R*^2^ of 0.543 (see SI, Table S2 for the full selectivity modelling results).

With the goal of using the models to suggest an enzyme to use for a given reaction, the enzymes were rank-ordered based on their predicted ΔΔ*G*^‡^ values for each substrate to analyze the top-*k* accuracy for suggesting the most selective enzyme (Fig. 4b). For ketones for which multiple enzymes exhibited the highest observed ee experimentally, we consider the model prediction a success if any of these enzymes were found in the predicted top-*k* set. The MVLR-DFT models achieved a promising 70% accuracy for selecting the top-1 best-performing enzyme, while 85% accuracy can be achieved when selecting two enzymes. Full accuracy is achieved by the MVLR-DFT models for top-4 enzyme prediction. Notably, the top models showed higher top-*k* accuracy than a naïve baseline of predicting the k-most frequent top-performing enzymes across the full dataset (Fig. 4b), which mimics how a chemist might select enzyme(s) for a substrate given this data. Analysis of the ee regret obtained from the top-1 predicted enzymes shows that the MVLR-DFT and DFT-based RF models exhibited the lowest regret values, with the MVLR-DFT model predicting fewer outlier outcomes compared to the DFT-based RF model (Fig. 4c). Therefore, the MVLR-DFT model was selected for all further experiments modelling enantioselectivity.

### Prospectively benchmarking the workflow with a held-out dataset

**Fig. 5:**
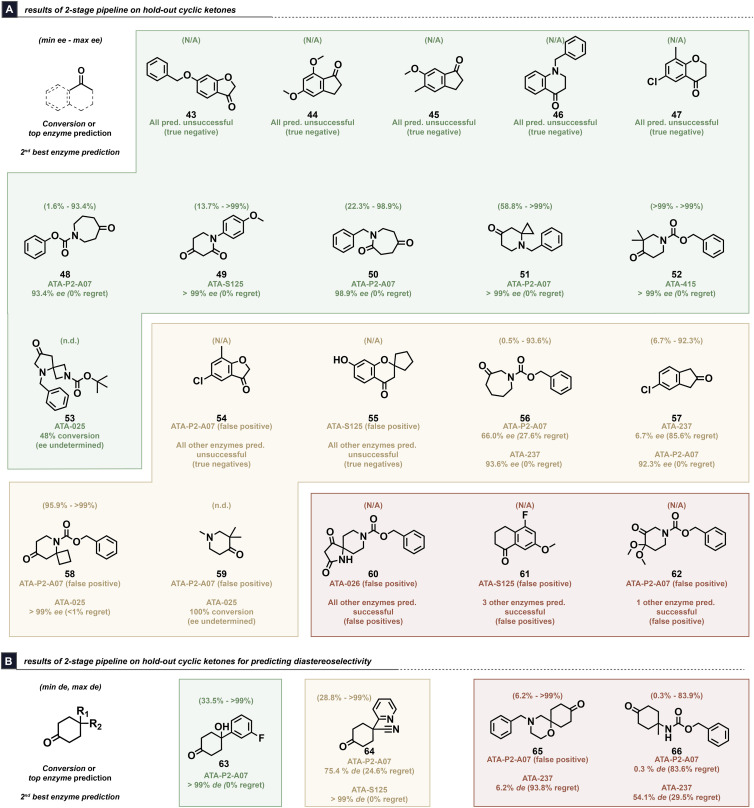
Results of the 2-stage conversion and enzyme selection on challenging held-out cyclic ketone substrates, predicting (A) enantioselectivity and (B) diastereoselectivity. Substrates are colored by whether the models afforded an ideal (green) outcome, acceptable (yellow) outcome, or poor (red) outcome. Within each category (green/yellow/red), substrates are ordered from highest to lowest maximum possible regret (*i.e.* the difference between the highest and lowest experimental ee, as depicted on top of each ketone). “N/A” indicates “not applicable,” while n.d. indicates “not determined.” ^*a*^The amine was too volatile for ee determination; ^*b*^product peaks could not be separated from impurities during ee determination. Reaction conditions: ketone (7.5 µmol; 500 mM in DMSO), transaminase (10 mg mL^−1^), PLP (0.5 mg mL^−1^), pH = 7.6 150 mM KPi buffer with 5% *v*/*v i*PrNH_2_ (25 mM), 35 °C, 24 h.

The combined 2-step workflow validation (conversion and subsequent selectivity prediction) was conducted on 24 cyclic substrates that did not appear in the initial dataset. Using the initial dataset as training, the conversion RF model with DFT features was used to predict whether the product could be formed with >25% conversion, while the top MVLR-selected DFT and Sterimol features for each enzyme were used to build models and rank-order the enzymes based on their predicted ΔΔ*G*^‡^ values. For each substrate, the top-ranked enzyme represented the highest selectivity prediction with a positive conversion prediction. The top-predicted enzymes, along with the maximum possible regret in ee for each substrate, were used to score the predictions from the workflow (Fig. 5). The conversion modelling on this held-out dataset exhibited an overall accuracy of 64% with an AUROC score of 0.71, exhibiting slightly lower performance compared to the LOOCV modeling used to evaluate the initial dataset (see SI, Fig. S23 for full classification metrics). This may be due to a distributional shift to lower conversions between the original dataset and held out set (see SI, Fig. S11). Additionally, considering that cyclic ketones represent roughly one-fourth of the training dataset, this observation is not necessarily surprising and implies that dataset enrichment could help bolster model performance on different classes of ketones.

The workflow was evaluated by classifying the outcomes as being ideal (green; the top-ranked enzyme furnished >25% conversion with <1% ee regret, or unsuccessful substrate received negative predictions from all conversion models), acceptable (yellow; the second-ranked enzyme fulfilled the conditions for ideal when the top-ranked enzyme did not, or unsuccessful substrate received only one positive conversion prediction), and poor (red; neither of the top-2 predicted enzymes afforded an ideal outcome, or unsuccessful substrate received >2 positive conversion predictions). Of the 24 substrates evaluated, 20 could potentially form two product enantiomers in line with the training dataset (Fig. 5a). There were a handful of substrates in this set where the top-ranked enzyme afforded sufficient conversion with minimal to no regret in the experimentally obtained ee (substrates 48–52). Meanwhile, three substrates were identified where the 2nd-ranked enzyme achieved sufficient conversion with low ee regret (substrates 56–58). The workflow was generally able to identify substrates where none of the eight enzymes could furnish the desired amine products, as well, with 5/10 unsuccessful substrates obtaining a negative conversion prediction from all enzymes (substrates 43–47) and two unsuccessful substrates (54 and 55) obtaining negative conversion predictions from 7 of the enzymes. The pre-identification of low-yielding substrates can be important, as this can enable a medicinal chemist to seek an alternative approach, consider preparative chiral separations, or to prioritize screening with a larger number of transaminases than those covered by our workflow. Gratifyingly, the models extended well to 7-membered cyclic substrates, which represent a class of molecules that were not included in the training dataset. Two of these substrates performed ideal in the workflow (substrates 48 and 50) and one obtained acceptable performance (substrate 56). The workflow furnished mixed results, however, with sterically hindered substrates. For example, the model correctly identified a top-performing enzyme for substrate 52 but failed to anticipate that substrate 60 would not produce sufficient conversion. It should be emphasized that despite these examples, which represent *false positive* predictions, that no substrate in this exercise exhibited an overall *false negative* conversion prediction from the workflow; all successful substrates received at least one positive prediction from a conversion model.

As a “stress-test” of the workflow, we evaluated 4 substrates that furnished *cis*- or *trans*-cyclohexane diastereomers as opposed to enantiomers (Fig. 5b). We anticipated that the information learned from training on ee would be challenging to apply towards diastereoselectivity. Despite this additional challenge, the workflow obtained ideal and acceptable performance for substrates 63 and 64, respectively. While these initial results are promising, they highlight the limitations of the workflow to expand beyond the typical use-case of transaminases.

Overall, the results from applying the two-stage workflow on a held-out set of 24 substrates highlight the potential to improve experimental turnaround time and reduce the amount of material used for screening. High-performing enzymes were identified among the top-two ranked enzymes suggested by the workflow for 10 substrates, along with correctly identifying 5 substrates that were non-reactive with the 8 transaminases included in this study. Furthermore, perturbing the measured conversion and ee values in this held-out dataset with uniformly-distributed random noise of up to ±5% conversion and ±1% ee did not result in changed assignments of the final predictions, suggesting that the two-stage workflow can be somewhat robust towards reasonable noise introduced into the measured endpoints (see SI, Table S8). A comparison of the two-stage fingerprint modelling performance on the held-out dataset was also conducted, finding more poor predictions than what was obtained using DFT features. This degradation in performance was primarily attributed to poorer rank-ordering of the enzymes' predicted ΔΔ*G*^‡^ values and suggests that the computational overhead of calculating DFT features is justified to improve modelling results. Similarly, applying the naïve baseline approach of always testing the top-2 enzymes identified from the HTE dataset resulted in more poor predictions, as this would result in substrates correctly identified as non-reactive by the DFT models being subjected to experimentation (see SI, Table S9). These results demonstrate the DFT models' potential to identify both high-performing transaminases and substrates that will be non-reactive.

### Prospective experiments for therapeutic molecules

**Fig. 6:**
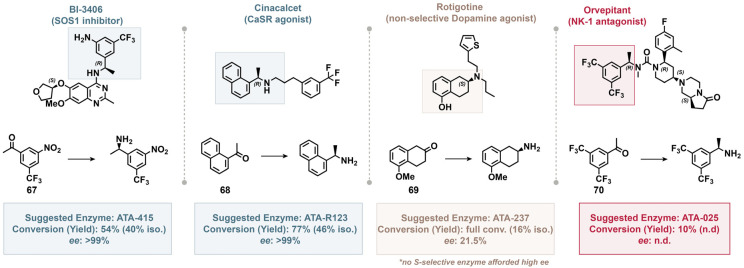
Prospective scale-up experiments showing the utility of the two-stage workflow. Suggested enzymes were limited to those with the desired selectivity for the enantiomer shown in the parent molecules. Isolated yields and ee values were determined after Boc protection and purification *via* reverse-phase preparative HPLC. Reaction conditions: ketone (112.5 µmol; 500 mM in DMSO), transaminase (10 mg mL^−1^), PLP (0.5 mg mL^−1^), pH = 7.6 150 mM KPi buffer with 5% *v*/*v i*PrNH_2_ (25 mM), 35 °C, 24 h; then Boc_2_O (2.5 equiv.) and Et_3_N (5.0 equiv.) in CH_2_Cl_2_ (18.75 mM).

To demonstrate the potential applicability of this workflow for medicinal chemistry research, prospective experiments were conducted to furnish representative chiral amines of those found in exemplary therapeutic/bioactive molecules (Fig. 6). For this exercise, the absolute stereochemistry of the resulting products was assumed to be that which the enzymes are typically selective for. Of the four substrates selected for scale-up and isolation, the top-ranked enzymes for three (67–69) were correctly predicted to exhibit >25% conversion. Gratifyingly, the naphthyl amine building block found in cinacalcet could be accessed with high conversion and excellent enantioselectivity using the top-ranked enzyme ATA-R123. Similarly, the nitro analog of the aniline found in BI-3406 could be prepared with sufficient conversion with an excellent enantioselectivity of >99% using the top-ranked *R*-selective enzyme ATA-415. Meanwhile, the methyl ether analog of the cyclohexylamine found in rotigotine afforded sufficient conversion, albeit with a low ee of ∼22% using ATA-237, the only *S*-selective enzyme predicted to succeed by the model. Further testing of this substrate verified that none of the *S*-selective enzymes could afford high enantioselectivity for this substrate (SI, Table S6), highlighting one scenario where the transaminases used in this study simply couldn't afford sufficient enantioselectivity. Finally, the bis-trifluoromethyl amine building block found in orvepitant exhibited a false positive prediction, as subsequent micro-scale experimentation identified that none of the 8 enzymes used in this study could furnish the product with >25% conversion. While there is literature precedence for a transaminase synthesizing this intermediate,^49^ the particular variant was not included in the panel of enzymes used for this study and the overall conditions (*i.e.* buffer pH, temperature, *etc.*) exhibited some differences from our standard protocol.

These results highlight the potential utility of the two-stage workflow in medicinal chemistry research by enabling the successful synthesis of two intermediates with high enantioselectivity, while also demonstrating that there can be instances where high enantioselectivity cannot be afforded by any of the enzymes available to this workflow.

## Conclusions

In summary, we have developed a dataset and modelling workflow for the selection of commercial transaminases from 8 possible choices given a prochiral ketone substrate, facilitating the synthesis of a diverse array of chiral amine building blocks relevant to medicinal chemistry. This was accomplished using HTE to construct a training dataset that served as the basis for an ML model development campaign. These efforts identified that a RF model using DFT features was performant for predicting conversions, while a forward MVLR model using DFT and steric features could best predict ΔΔ*G*^‡^ to rank-order the enzymes according to their predicted enantioselectivities. The workflow exhibited promising results when applied towards a challenging held-out dataset of cyclic substrates, demonstrating modest generalizability towards this class of substrates and successfully guided the synthesis of two pharmaceutically-relevant chiral amines with sufficient conversion and excellent enantioselectivity.

Our selectivity modelling results from the initial transaminase dataset suggest that the experimental screening burden can be cut in approximately half, while recapitulating the top-performing enzyme for each substrate. For the holdout cyclic dataset, in the majority of cases, the models recapitulated the highest experimentally-observed ee value (±1%) within the top-2 enzyme predictions. Additionally, in cases where the desired stereochemistry of the product is known, the two-stage workflow can be adapted to only consider the (*R*)- or (*S*)-selective enzymes, assuming the substrate fits into the enzyme active sites as expected. While the small selection of (*R*)- or (*S*)-selective enzymes evaluated in this study can represent a potential limitation when constraining enzyme selection to one of these subsets, evaluation of the held-out dataset within either subset revealed similar overall results compared to that which was obtained without constraining the enzyme choices when classifying the predictions according to the criteria described in Fig. 5 (see SI, Table S10).

Importantly, the results in this study were achieved using only features of the ketone substrates, with no explicit knowledge of each enzyme's sequence or active site structure. Furthermore, the DFT calculations for these smaller, building block substrates can be relatively fast. The results from this study suggest that the performance decrease associated with using features with faster calculation speed (*e.g.*, fingerprints) outweighs the potential gains in decision speed, particularly for selectivity modelling, as performance degradation was noted upon modelling the held-out dataset with fingerprint features (see SI, Table S9). This decrease in performance was primarily attributed to poorer rank-ordering of the enzyme selectivity predictions. The use of surrogate models to predict DFT features could potentially decrease the computational overhead required to conduct modelling and has been previously evaluated in other chemical reactivity prediction tasks.^43,50^

While there is much room for improvement of the models' quantitative results, for both binary conversion and regression-based selectivity prediction, further data collection could bolster model predictivity, particularly in identifying substrates for which conversion is not afforded across any enzyme. As only a small subset of the possible search space was selected for the initial HTE dataset (∼1.3%), we would anticipate that modelling performance would continue to improve as more substrates are evaluated. Furthermore, it would be prudent to include prochiral ketone subtypes that were not included in the initial search space, such as pyruvic acids/esters, to more broadly cover substrates of interest in medicinal chemistry. Ideally, sets of 24 diverse substrates would be chosen from new subsets for experimentation and further dataset expansion would be conducted in rounds until performance of the models plateaus. Finally, identification and inclusion of an additional 2–4 transaminases that can consistently convert conformationally rigid substrates, such as indanones and tetralones, would benefit by providing positive data for these substrate types. Active learning is one possible avenue for identifying and selecting these substrates, or more broadly, substrates with the potential to bolster model performance.^51,52^ Our results also highlight the inherent challenges presented when attempting to build models for enantioselectivity from HTE datasets, as measurements from roughly 1/3 of the initial dataset could not be obtained due to low conversions or challenges deriving ee from crude reaction mixtures. While HTE is a powerful tool for many transformations of relevance to medicinal chemistry, measuring enantioselectivity presents additional analytical challenges that can introduce a bottleneck to training data collection. Nevertheless, the modelling results achieved using this training data exhibited modest generalizability in this study, based on the LOOCV modelling results and the two-stage modelling workflow applied towards the held-out dataset.

We hope the full disclosure of our transaminase HTE datasets and modelling results will encourage further experimental efforts and substrate-centred modelling campaigns for biocatalytic reactions. Despite the potential of biocatalysis for chiral building block synthesis, its application in medicinal chemistry has remained limited. This is primarily due to the need for extensive enzyme screening in the absence of sufficient experience or literature precedent, as well as the time investment required for such investigations. Biocatalysis HTE campaigns can be labour-intensive, particularly when analysing ee across entire reaction plates. Speed is a critical factor in medicinal chemistry, and as a result biocatalysis is often considered a last-resort option for chiral synthesis, despite the high selectivity these enzymes can offer. Therefore, we hope the workflow and experimental data described herein can assist in lowering the barrier for biocatalysis use in medicinal chemistry efforts.

## Author contributions

A. J. R. conducted biocatalytic experiments, substrate/reference standard syntheses, and DFT calculations. P. R. built and conducted the workflows for descriptor generation and ML modelling experiments. L. S.-C. conducted all chiral SFC analyses. R. Z. contributed to enzyme and substrate selection. The manuscript was written through contributions from all authors. A. J. R., Y. W., and C. W. C. directed the research. All authors have given approval to the final version of the manuscript.

## Conflicts of interest

The authors declare the following competing financial interests. A. J. R., P. R., L. S.-C., and Y. W. are employees of AbbVie, Inc. R.Z. is a former employee of AbbVie, Inc. The design, study conduct, and financial support for this research were provided by AbbVie, Inc. AbbVie, Inc., participated in the interpretation of data, review, and approval of this publication. P. R. was an employee of MIT during the majority of the study. C. W. C. is an employee of MIT and has no conflicts to declare.

## Supplementary Material

SC-017-D6SC00852F-s001

SC-017-D6SC00852F-s002

SC-017-D6SC00852F-s003

## Data Availability

Datasets and code used in this study are available at https://github.com/priyanka-rag/transaminase_external. Supplementary information: detailed experimental methodology and spectra. See DOI: https://doi.org/10.1039/d6sc00852f.
