# Supplementary material for: A machine learning-based workflow for transaminase selection
Source: Chem Sci. 2026 May 20;17(25):12334–45. doi: 10.1039/d6sc00852f (PMC13187754; doi:10.1039/d6sc00852f)

Supplementary Information (SI) for Chemical Science.
This journal is © The Royal Society of Chemistry 2026

Supporting Information

A Machine Learning-Based Workflow for Transaminase Selection

Alexander J. Rago,*[a]‡ Priyanka Raghavan,[b,c]‡ Lisandra Santiago-Capeles,[a] Ruijie Zhang,[a] Ying Wang,*[a] and Connor W. Coley*[b]

[a]Small Molecule Chemistry Technologies, AbbVie, Inc., 1 N Waukegan Rd, North Chicago, IL 60064, United States; [b]Department of Chemical Engineering, Massachusetts Institute of Technology, 77 Massachusetts Ave, Cambridge, MA 02139, United States. [c]Computational Drug Discovery, AbbVie, Inc., 1000 Gateway Blvd, South San Francisco, CA 94080.

‡A.J.R. and P.R. contributed equally to this work.

*Email: alex.rago@abbvie.com; wang.ying@abbvie.com; ccoley@mit.edu

Table of Contents

1. General Information S2

2. Machine Learning Experimental Details S3

3. Supplementary Figures S19

4. Experimental Procedures for Racemic Standard Syntheses S164

5. Experimental Procedures for the Transaminase HTE and Scale-Up Reactions....S165

6. Racemic Standards Characterization S166

7. References S182

8. ^{1}H, ^{13}C, and ^{19}F NMR Spectra S183

1. General Information

General Experimental Considerations:

All transaminase reactions were conducted with an air atmosphere under standard benchtop conditions. Dimethyl sulfoxide (472301-4L, Millipore Sigma) was purchased and used to prepare ketone stock solutions for HTE. The enzymes used were obtained from Prozomix (ATA-026 and ATA-031), EnzymeWorks (ATA-S125 and ATA-R123), and Codexis (ATA-237, ATA-P2-A07, ATA-025, and ATA-415) as powders. Water used in the reactions was deionized using a Barnstead NANOpure Diamond water purifier. The ketones and racemic amine standards were sourced commercially or from previously reported procedures (please see **Figures S16 & S19** for compound structures). For analysis of the crude reactions, filtration was conducted using a 350 µL GHP 96-well filter plate (Pall Laboratories) into an LCMS plate (200 µL volume, V-bottom or U-bottom 96-well plate, Corning, part number 29444-102). All centrifugation steps were conducted using a ThermoScientific Multifuge X Pro. Transaminase reactions were conducted using a New Brunswick Scientific INNOVA 42R incubator with a 1-inch orbital shaker stroke.

Crude transaminase HTE conversion analyses were performed on a Waters Acquity UPLC system equipped with an in-line photodiode array detector (PDA) and an SQD mass spectrometer, running MassLynx 4.1 and Openlynx 4.1 software (Waters Corporation, Milford, MA, USA). The SQD mass spectrometer was operated under positive ESI ionization conditions. The column used was a Waters BEH C8, 1.7 µm (2.1 mm × 30 mm) at a temperature of 55 °C. The following ammonium acetate analytical method was used: a gradient of 1-100% acetonitrile (A) and 10 mM ammonium acetate in water with 2% acetonitrile (B) was used, at a flow rate of 1.0 mL/min (0-0.1 min 1% A, 0.1-2.85 min 1-100% A, 2.85-2.99 min 100% A, 2.99-3.0 min 100-1% A). Samples were analyzed using custom software to interact with the MassLynx report files. Alternatively, an Agilent Infinity II HPLC equipped with an InfinityLab LC/MSD XT detector was used for some samples, running Agilent software with a custom sample submission suite. The column used was a Waters Cortecs C18+, 2.7 µm (2.1 mm × 50 mm) at a temperature of 40 °C. The following ammonium acetate analytical method was used: a gradient of 2-100% acetonitrile (A) and 10 mM ammonium acetate in water (B) was used, at a flow rate of 1.8 mL/min (0-0.05 min 2% A, 0.05-2.8 min 2-100% A, 2.8-3.0 min 100% A). Unless noted otherwise in the dataset, the conversion calculated from 220 nm UV was used.

Chiral SFC column and co-solvent screening and enantiomeric excess determinations were performed using the Waters UltraPerformance Convergence Chromatography® (UPC2®) system, equipped with ACQUITY photodiode array and mass spectrometry (QDa) detection. The system features a binary solvent manager, sample manager, convergence manager, and two column ovens accommodating 15 columns with dimensions of 4.6 x 100 mm and a particle size of 3 µm. **Table S11** outlines the column, co-solvent, gradient or isocratic conditions used for analysis. The mobile phase consisted of CO_2 mixed with a co-solvent and was pumped at a flow rate of 3 mL/min (alternate flow rate is specified in **Table S11**). Additionally, it includes a makeup pump (0.45 mL/min) with a solution of 85:15 methanol:water containing 0.1% formic acid. Data acquisition and analysis were conducted using Empower 3 Pro software. The sample compartment was maintained at room temperature, and the maximum injection volume utilized was 7.5 µL.

Isolation of products from reaction mixtures was accomplished with a preparative-scale reverse-phase HPLC instrument. When possible, an ammonium acetate purification method was used: for example, a gradient of acetonitrile (A) and 10 mM ammonium acetate in water (B) was used, at a flow rate of 40 mL/min (0-0.5 min 15% A, 0.5-8.0 min linear gradient 15-100% A, 8.0-9.0 min 100% A, 9.0-9.1 min linear gradient 100-15% A, 9.1-10.0 min 15% A). Focused gradients were also employed for more challenging

separations. Samples were injected in 1.5-2.0 mL MeOH. The main instrument used was a custom purification system which consisted of two Kinetex 5 µm C18 100 Å columns (50mm x 30 mm) connected in series with the following modules: Gilson 305 and 306 pumps; Gilson 806 Manometric module; Gilson UV/Vis 155 detector; Gilson 506C interface box; Gilson FC204 fraction collector; Agilent G1968D Active Splitter; Thermo MSQ Plus mass spectrometer. A flow rate of 40 mL/min was used. The system was controlled through a combination of Thermo Xcalibur 2.0.7 software and a custom application written in-house using Microsoft Visual Basic 6.0. An Agilent preparative HPLC system was used for isolation of the scale-up transaminase products using analytical-to-preparative purification workflows to develop a custom gradient (10mM ammonium acetate and acetonitrile) for each sample. The instrument consisted of two Kinetex 5 µm C18 100 Å columns (50mm x 30 mm) connected in series (preparative) and one Luna 5 µm C18 100 Å column (50mm x 4.6mm; analytical) with the following modules: 1290 Prep HiP ALS, 2x 1290 Prep HiP FC, 1260 Quat Pump, 1290 Prep Pump, 1260 DAD WR, 1290 Valve Drive, 1290 MS Flow Modulator, 1260 FC Cluster Valve. The system was controlled through Agilent ChemStation.

NMR spectra were acquired on two Bruker Avance III HD 500 MHz spectrometers with DCH cryoprobes, a 600 MHz spectrometer, or a 400 MHz spectrometer, all equipped with sample changers. Samples were also acquired on two 400 MHz Varian MR-400 spectrometers, particularly those conducted at 90 °C. Chemical shifts are reported as parts per million (ppm) downfield from tetramethylsilane and referenced to the solvent (e.g. DMSO-*d6*, pyridine-*d5*, $CDCl_3$). NMR data are represented as follows: chemical shift, multiplicity (s = singlet, br s = broad singlet, d = doublet, dd = doublet of doublet, t = triplet, q = quartet, p = quintet, h = sextet, hept = septet, dtt = doublet of triplet of triplets, qd = quartet of doublets, qt = quartet of triplets, ddtd = doublet of doublet of triplet of doublets, m = multiplet, etc.), coupling constant, and integration. Coupling constants (J) are given in Hertz (Hz). The temperature was recorded for samples conducted at elevated temperatures, otherwise the samples were acquired at room temperature.

High-resolution mass spectrometry (HRMS) characterization was conducted using either a Thermo LQT Orbitrap or Thermo IQX Orbitrap mass spectrometer. Both instruments used positive mode heated electrospray ionization. HRMS data are reported as follows: Calcd for {formula} [{ion}]$^+$: {calcd value}; Found: {measured value}.

Machine learning was conducted using a workstation with the following configuration: 128 threads, 64 Core CPU (AMD Ryzen Threadripper PRO 3995WX 64-Cores); 3 x GPUs (NVIDIA GeForce RTX 3090); 512GB DDR4 RAM; 1x1TB SSD; 1 x 2TB SSD. DFT calculations were conducted using a high-performance compute cluster with the following node configuration: 32 cores/64 threads CPU (AMD EPYC 7543) with 256 GB RAM. Gaussian 16 version ES64L-G16RevC.02 was used for all DFT calculations.[1]

2. Machine Learning Experimental Details

Disclaimer: This code was tested on a few different machines, and we observed slight numerical variations across different hardware architectures.

Code Availability: Code for this manuscript is available at https://github.com/priyanka-rag/transaminase_external

a. Dataset availability and details

The datasets used in this study were constructed via HTE and has been included in the Supplemental Materials. For brevity of notation, the datasets list the enzymes from Table 1 of the main text as Enz1, Enz2, Enz3, Enz4, Enz6, Enz7, Enz8, Enz9, respectively.

b. Modelling details

Base model details: The Random Forest (RF) models were provided by Sci-kit learn (RandomForestClassifier / RandomForestRegressor), and the Multivariate Linear Regression (MVLR) model was provided by Sci-kit learn (LinearRegression). For both, models with default hyperparameters were used due to the small dataset size. The basic procedure for building the MVLR model for a single enzyme is as follows, where k refers to the number of descriptors used in the model:

- Identify most correlated descriptor ($k = 1$) to the target variable across the enzyme-specific dataset.
- Using the remaining features, identify all possible 2-descriptor sets by adding a feature to the $k = 1$ case. Evaluate multivariate linear regression modeling for each set using LOOCV on the ketones. Select the feature set providing the highest R^2 to take forward.
- Repeat with 3-descriptor sets, 4-descriptor sets, k+1 descriptor sets, etc. until the highest LOOCV R^2 drops as compared to the previous k^{th} iteration.
- Save the best identified descriptor set for inference.
- During inference on unseen data (i.e. the heldout cyclic dataset and prospective ketone experiments), use the saved descriptors to build a multivariate linear regression model on the enzyme-specific initial dataset subset, and evaluate it on the heldout test set.

Reaction modeling details: In all cases, features derived from the ketone substrates were used, as we built per-enzyme models and reaction conditions were constant across all reactions.

Reaction splitting details: The dataset was separated based on the enzyme used, and models were trained and tested on the per-enzyme sub-datasets. For the initial modeling, local models were constructed using leave-one-out cross-validation (LOOCV) on the ketones. Due to the nature of LOOCV, the model is tested on an unseen substrate each time.

Substrate Features:

- One-hot encoding (OHE): Obtained using Sci-Kit Learn's OneHotEncoder. The last position in the encoding is saved for unseen substrates (i.e. those in the test set).
- Morgan fingerprints (fp): Obtained using RDKit[2] (AllChem.GetMorganFingerprintAsBitVect). Radius was 2 and nBits was 2048 in all cases.
- Physicochemical features (phys): Obtained using RDKit's Descriptors module. This gives 208 features for each molecule.
- DFT features (DFT): Obtained using Gaussian16 and the Auto-QChem pipeline. See Section C for a detailed explanation of the DFT pipeline and descriptor histograms.
    - For the RF models: Certain DFT features were pre-selected to feed into the RF models, based on those that exhibited varied distributions across the dataset. The atom-level descriptors were concatenated with molecule-level descriptors to form the final feature vector of length 32 for each reaction, used as input to the models. The exact atom and molecule-level descriptors used for modeling are shown below:

    - Atom-Level Descriptors (for carbonyl carbon and both alpha-carbons): buried volume (VBur), Mulliken charge (Mulliken_charge), atomic polar tensor charge (APT_charge), natural population analysis charge (NPA_charge), natural population analysis valence (NPA_valence), NMR anisotropy (NMR_anisotropy), ES_root_Mulliken_charge, ES_root_NPA_charge
    - Molecule descriptors: dipole (dipole), molar mass (molar_mass), self-consistent field energy (E_scf), electronic spatial extent (electronic_spatial_extent), HOMO energy (homo_energy), LUMO energy (lumo_energy), electronegativity, Gibbs free energy (G)
  - For the MVLR-DFT models: MVLR provides an opportunity to actively select features based on what the model deems important. Therefore, all calculated DFT and steric descriptors were provided to the models. This represents 74 descriptors: ['dipole', 'molar_mass', 'molar_volume', 'electronic_spatial_extent', 'E_scf', 'zero_point_correction', 'E_thermal_correction', 'H_thermal_correction', 'G_thermal_correction', 'E_zpe', 'E', 'H', 'G', 'homo_energy', 'lumo_energy', 'electronegativity', 'hardness', 'ES_root_dipole', 'ES_root_molar_volume', 'ES_root_electronic_spatial_extent', 'VBur.Carbon1', 'Mulliken_charge.Carbon1', 'APT_charge.Carbon1', 'NPA_charge.Carbon1', 'NPA_core.Carbon1', 'NPA_valence.Carbon1', 'NPA_Rydberg.Carbon1', 'NPA_total.Carbon1', 'NMR_shift.Carbon1', 'NMR_anisotropy.Carbon1', 'ES_root_Mulliken_charge.Carbon1', 'ES_root_NPA_charge.Carbon1', 'ES_root_NPA_core.Carbon1', 'ES_root_NPA_valence.Carbon1', 'ES_root_NPA_Rydberg.Carbon1', 'ES_root_NPA_total.Carbon1', 'VBur.Carbonyl', 'Mulliken_charge.Carbonyl', 'APT_charge.Carbonyl', 'NPA_charge.Carbonyl', 'NPA_core.Carbonyl', 'NPA_valence.Carbonyl', 'NPA_Rydberg.Carbonyl', 'NPA_total.Carbonyl', 'NMR_shift.Carbonyl', 'NMR_anisotropy.Carbonyl', 'ES_root_Mulliken_charge.Carbonyl', 'ES_root_NPA_charge.Carbonyl', 'ES_root_NPA_core.Carbonyl', 'ES_root_NPA_valence.Carbonyl', 'ES_root_NPA_Rydberg.Carbonyl', 'ES_root_NPA_total.Carbonyl', 'VBur.Carbon2', 'Mulliken_charge.Carbon2', 'APT_charge.Carbon2', 'NPA_charge.Carbon2', 'NPA_core.Carbon2', 'NPA_valence.Carbon2', 'NPA_Rydberg.Carbon2', 'NPA_total.Carbon2', 'NMR_shift.Carbon2', 'NMR_anisotropy.Carbon2', 'ES_root_Mulliken_charge.Carbon2', 'ES_root_NPA_charge.Carbon2', 'ES_root_NPA_core.Carbon2', 'ES_root_NPA_valence.Carbon2', 'ES_root_NPA_Rydberg.Carbon2', 'ES_root_NPA_total.Carbon2', 'L.CC1', 'B1.CC1', 'B5.CC1', 'L.CC2', 'B1.CC2', 'B5.CC2']
- Sterimol Parameters: Bond-level sterimol parameters of both carbonyl-alpha carbon bonds were calculated from the DFT-optimized conformational ensemble. These were calculated using the Sterimol package provided by Morfeus.[3]

<u>Conversion modeling</u>: Binary RF classifiers were built for modeling conversion. Labels were assigned as 0/negative if the recorded conversion was 25% or less, or 1/positive if greater than 25%.

<u>Selectivity modeling</u>: Both RF regressors and MVLR models were explored for initial selectivity modeling. *ee* values were converted to ddG values via the following equation, where the *ee* value is a fraction between 0 and 1:

$$\Delta\Delta G^{\ddagger} = RT\ ln|e.r.| = RT\ ln\left(\frac{1+ee}{1-ee}\right)$$

The obtained values were multiplied by –1 for R-selective enzymes, to distinguish between S and R products. In cases where the recorded *ee* was 100% (1 as a fraction), to avoid division by 0 the value was set to 0.9999 prior to input into the equation. R = 0.0019872036 kcal/mol K, and T = 308.15 K (the reaction temperature).

c. DFT pipeline, descriptor histograms, and correlations

The full process to generate the DFT descriptors that were used as input to the ML models is detailed as follows. We use the AutoQ-Chem pipeline restructured to deploy locally:

1. Unique ketone structures were obtained from the datasets. Ketone carbonyl and alpha-carbon atoms were identified from the atom-mapped molecules.
2. For each substrate, conformers were generated in RDKit [https://www.rdkit.org] and initial optimization was conducted using molecular force field methods (*MMFF-94*). This resulted in 1-20 conformers per molecule. RDKit parameters used for conformer generation are given below (note: these are the same parameters used in Auto-QChem[2]):
   - useSymmetryForPruning = True
   - useSmallRingTorsions = True
   - useMacrocycleTorsions = True
   - ETversion = 2 (default = 1)
   - pruneRmsThresh = 0.35 (default = -1.0)
   - Max. # of conformers = 20 (default = 10)
   - randomSeed = 42
2. Gaussian16 geometry optimization and frequency calculations were then used to calculate several DFT descriptors for each conformer in the ensemble. The set of Gaussian input commands used for each conformer is given below:
   - # opt=CalcFc B3LYP/6-31G* scf=xqc
   - # freq B3LYP/6-31G* volume NMR pop=NPA density=current Geom=AllCheck Guess=Read
   - # TD(NStates=10, Root=1) B3LYP/6-31G* volume pop=NPA density=current Geom=AllCheck Guess=Read

   For molecules where geometry optimization converged but not frequency, an additional step was added before the final TD step:
   - # opt=ReadFC freq B3LYP/6-31G* volume NMR pop=NPA density=current Geom=AllCheck Guess=Read
   - Frequency analysis was used to confirm the nature of all stationary points. Most conformers were successfully run through Gaussian the first time around. Among those that failed, most were able to be corrected through increasing the maximum number of cycles allotted for geometry optimization. Conformers that could not be fixed with these solutions were dropped from the ensemble modelling.
3. 25 molecular descriptors per molecule and 19 atom descriptors per atom per molecule were extracted.

4. Post-DFT, the structures of conformers will have changed due to geometry optimization. Therefore, we re-prune conformers to remove redundant conformers and get a representative set, again using an RMSD-based threshold of 0.35. Amongst the kept conformers for each substrate, we calculate Boltzmann-averaged values of each descriptor using the relative conformer Gibbs free energies.
5. For the calculation of Sterimol parameters, we again compute Boltzmann averages, using the optimized geometries of the kept conformers, as described in the previous step.
6. Using the atom-mappings as obtained in Step 1, the specific atom descriptors of the carbonyl carbon, and both alpha carbons, as well as Sterimol parameters down both carbonyl-alpha carbon bonds, were extracted for each substrate. Example histograms of some pertinent descriptors across all reactions in our initial dataset are shown below:

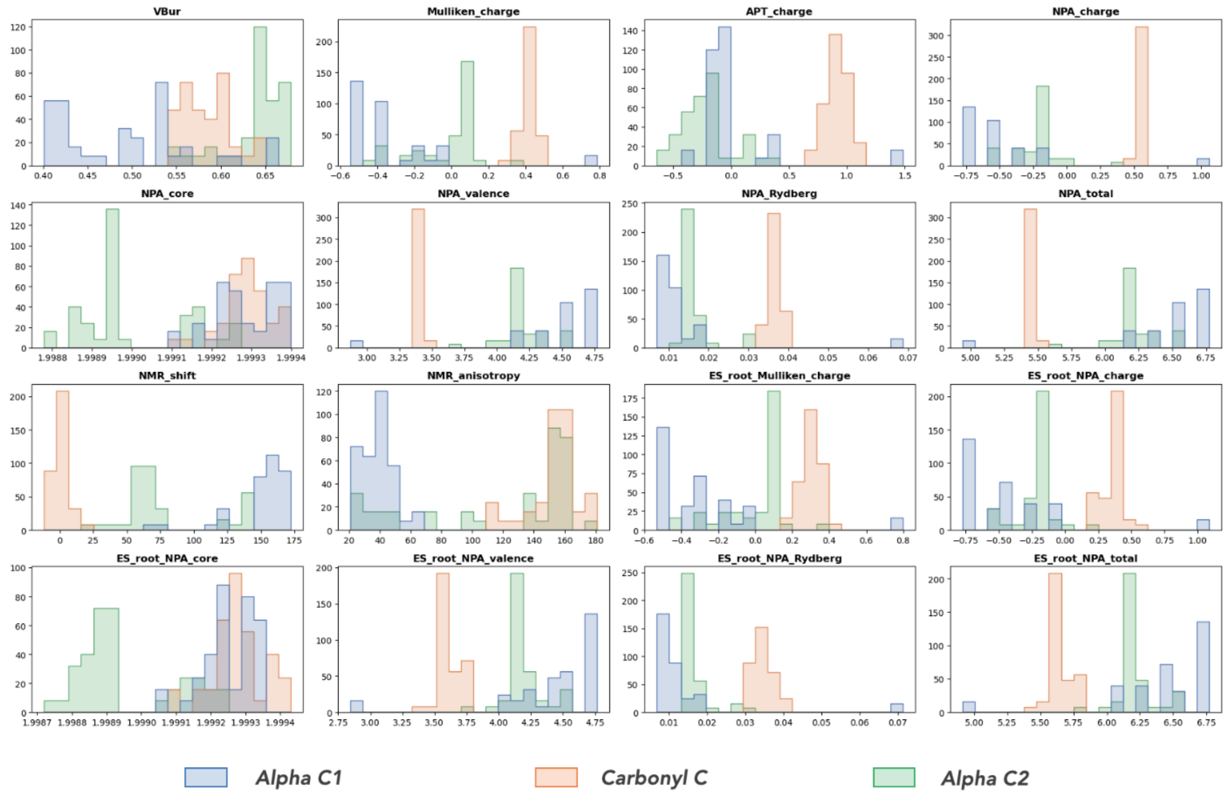

Figure S1. Histograms of 16 atom-level descriptors obtained from DFT across all reactions, colored by each type of atom (both alpha carbons, carbonyl carbon) in the reactive site. Recall that C1 is the less sterically-hindered alpha carbon, and C2 is the more sterically-hindered alpha carbon.

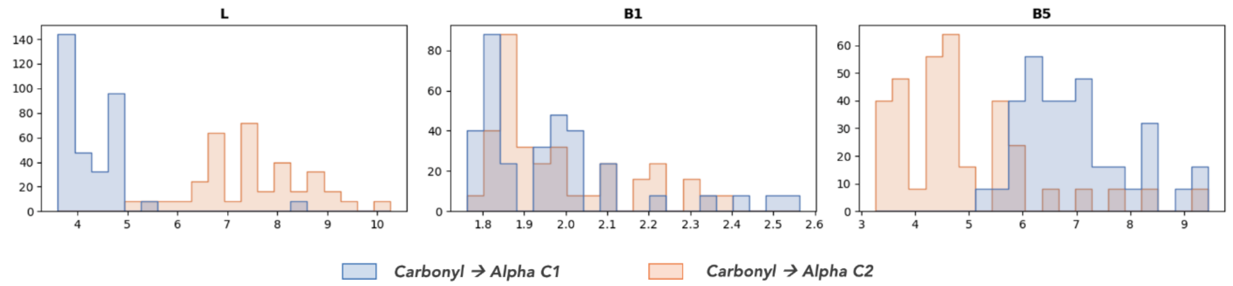

Figure S2. Histograms of 3 Sterimol parameters across all reactions (calculated from DFT-optimized ensemble geometries), colored by each bond in the reactive site.

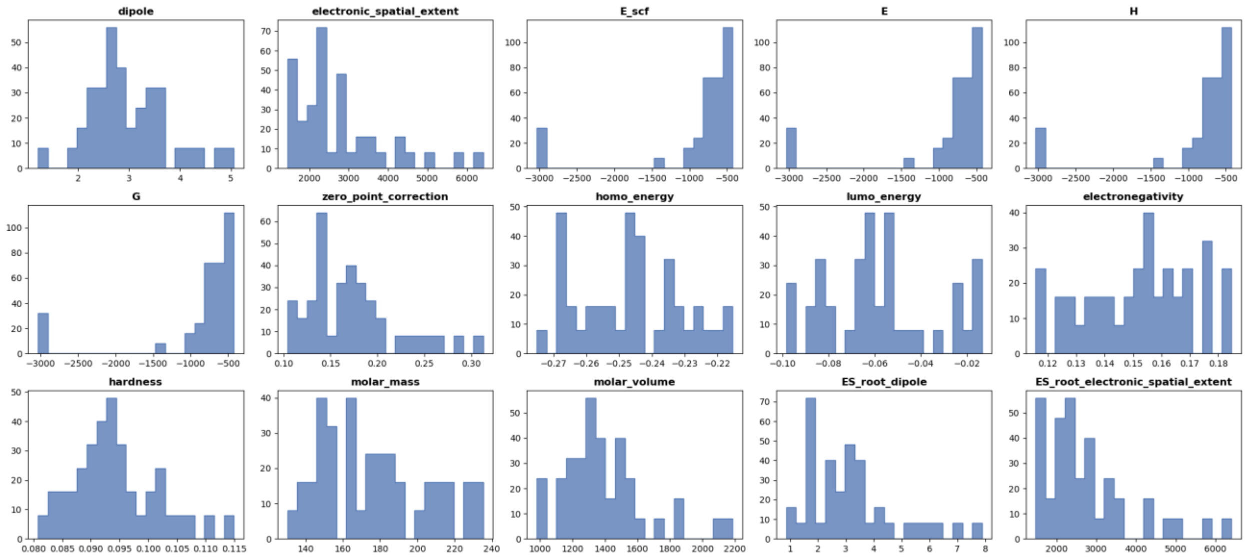

Figure S3. Histograms of 15 molecule-level descriptors across all reactions.

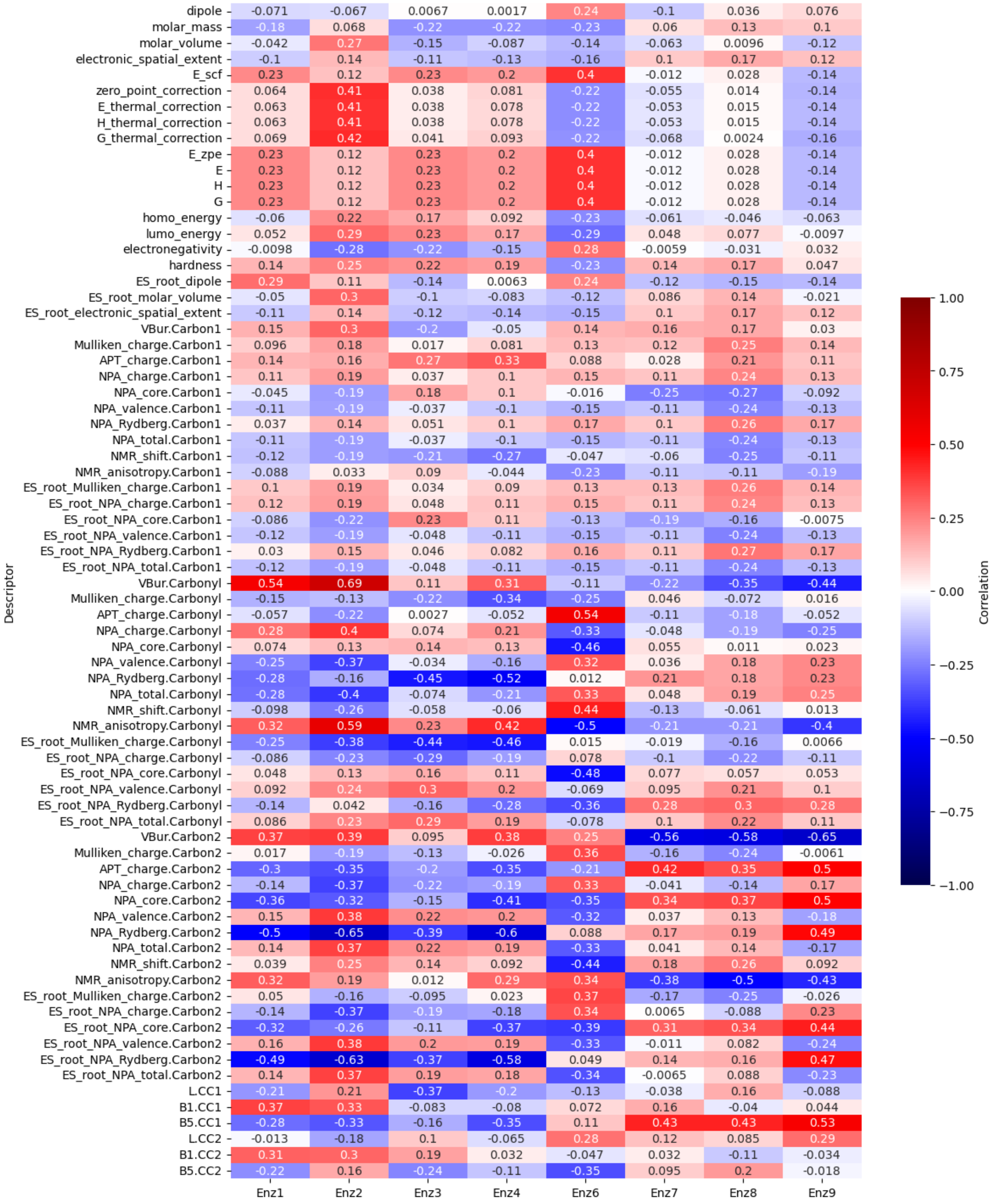

Figure S4. Heatmap of Pearson correlations of all DFT and Sterimol descriptors to the $\Delta\Delta G^{\ddagger}$ values. Deeper colors indicate stronger correlations, with red indicating positive correlation and blue indicating negative correlation.

d. Dataset Characterization

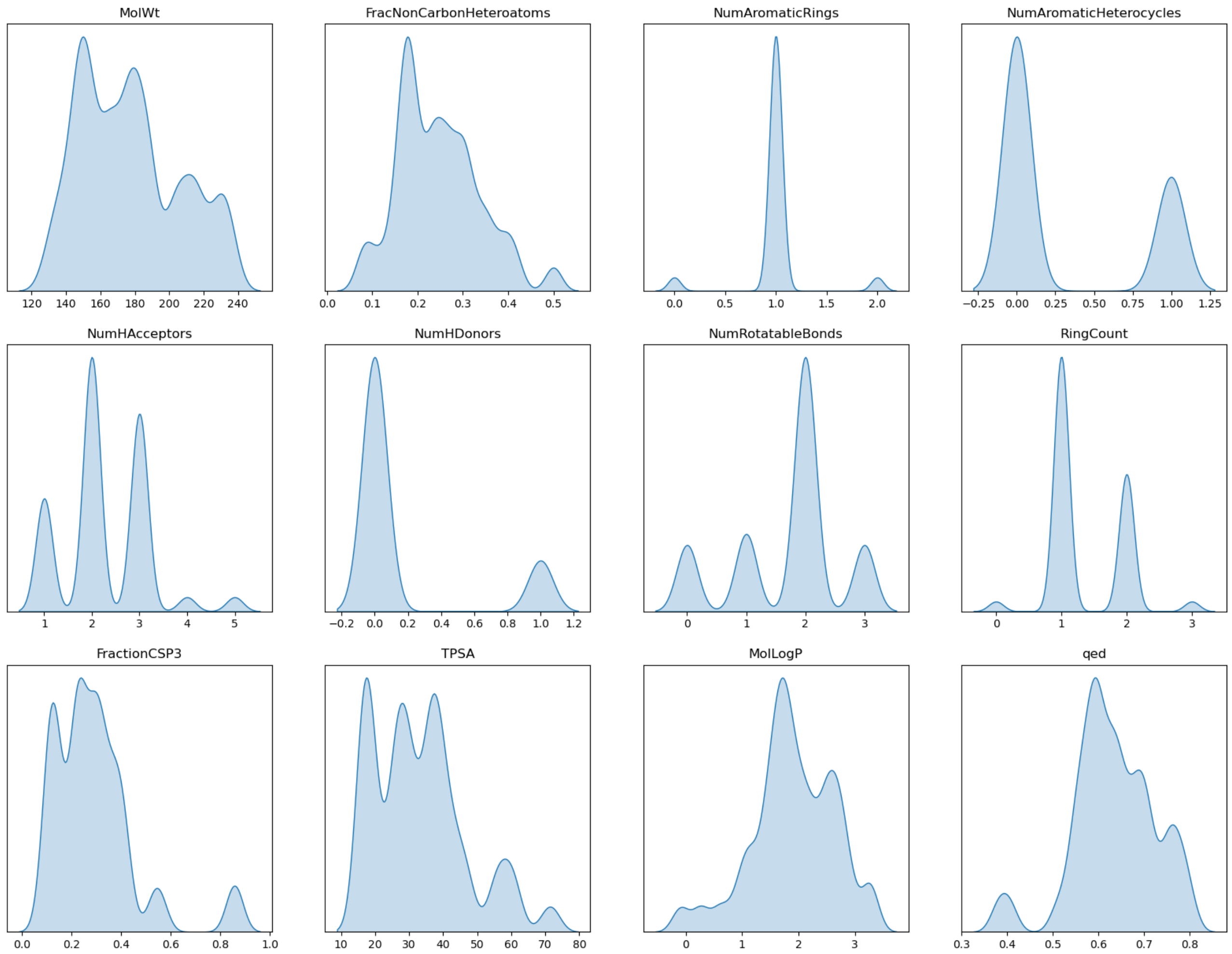

Figure S5. KDE (Kernel Density Estimation) plots of various structural and physiochemical properties across the structures in the initial HTE dataset.

Properties shown include MolWt (molecular weight), FracNonCarbonHeteroatoms (# of non-C heteroatoms/# of heavy atoms), NumAromaticRings (# of aromatic rings), NumAromaticHeterocycles (# of aromatic heterocycles), NumHAcceptors (# of H-bond acceptors), NumHDonors (# of H-bond donors), NumRotatableBonds (# of rotatable bonds), RingCount (ring count), FractionCSP3 (fraction of sp3 C atoms), TPSA (topological polar surface area), MolLogP (Crippen's computational LogP estimation),[4] and qed (quantitative estimate of druglikeness). All properties were computed via RDKit's Descriptors module.

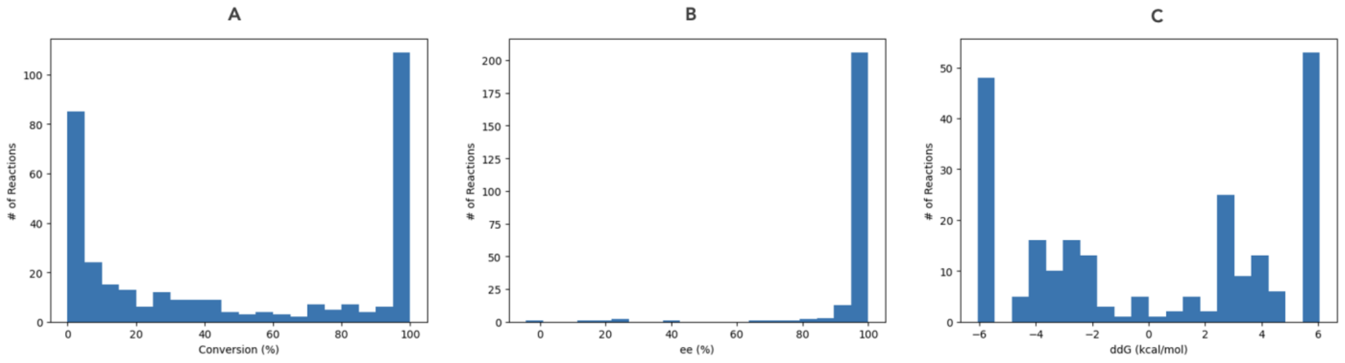

Figure S6. Distributions of (A) conversion, (B) enantiomeric excess, and (C) $\Delta\Delta G^{\ddagger}$ values across the initial HTE dataset. For (C), note that positive values correspond to reactions yielding the S-product, while negative values correspond to reactions yielding the R-product.

e. Held-Out Dataset Characterization

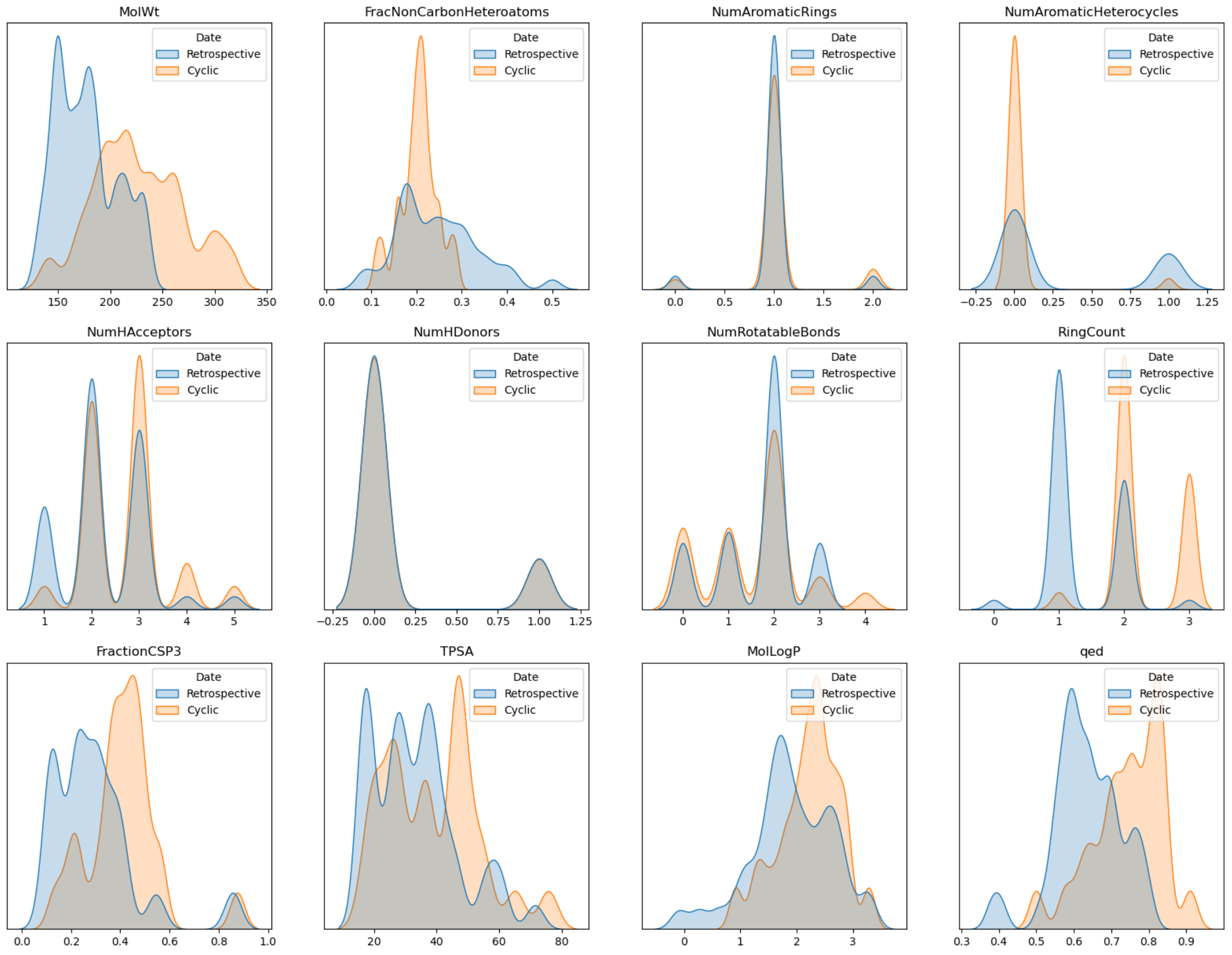

Figure S7. KDE (Kernel density estimation) plots showing distributions of the ketone structures in the initial (retrospective) and cyclic heldout datasets across various structural physiochemical properties.

Note that the initial and cyclic heldout distributions have been normalized separately for each property. See the discussion under Figure S5 for explanations of each property.

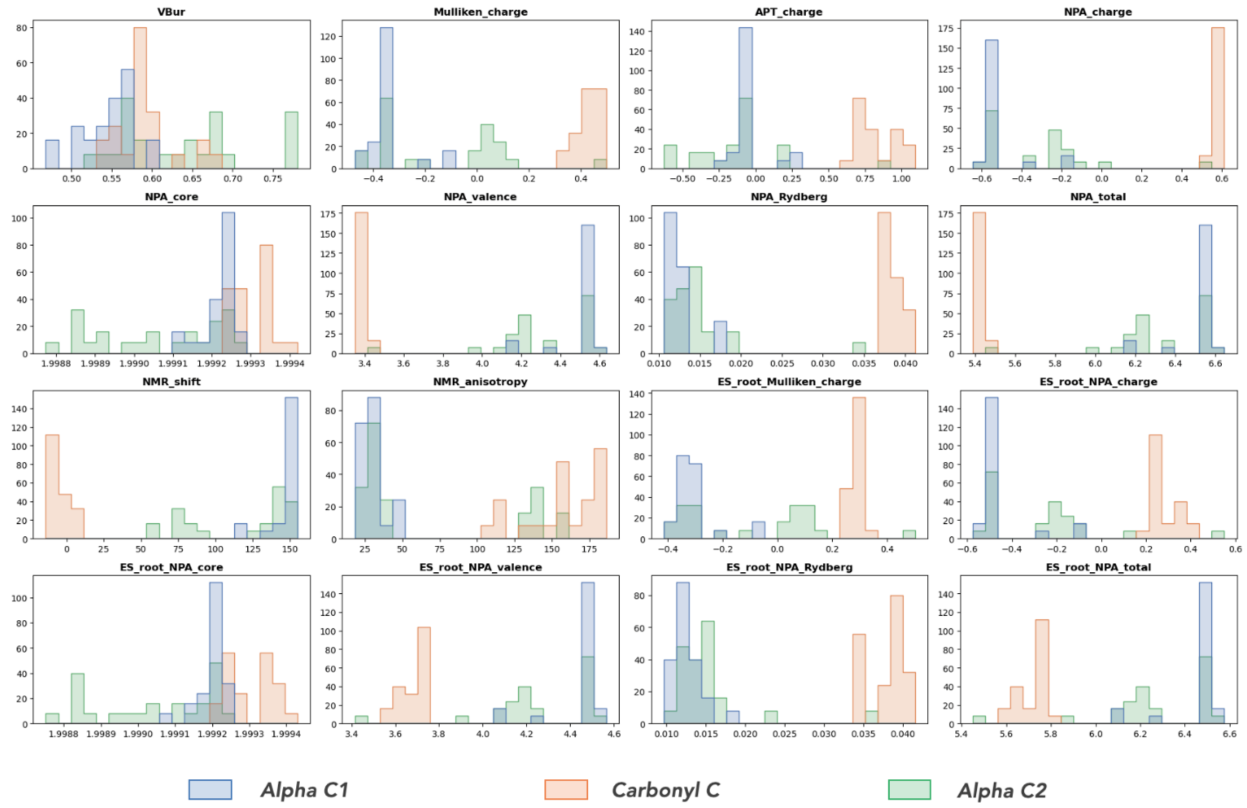

Figure S8. Histograms of 16 atom-level descriptors across all reactions in the cyclic heldout dataset, colored by each type of atom in the reactive site.

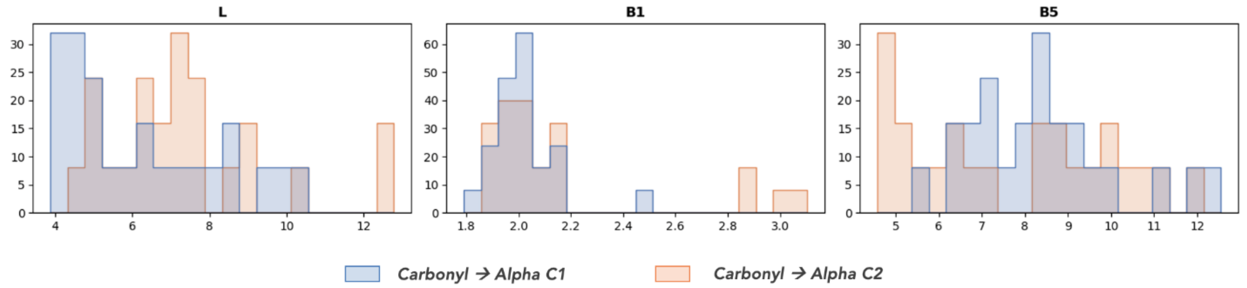

Figure S9. Histograms of 3 Sterimol parameters across all reactions in the cyclic heldout dataset, colored by each bond in the reactive site.

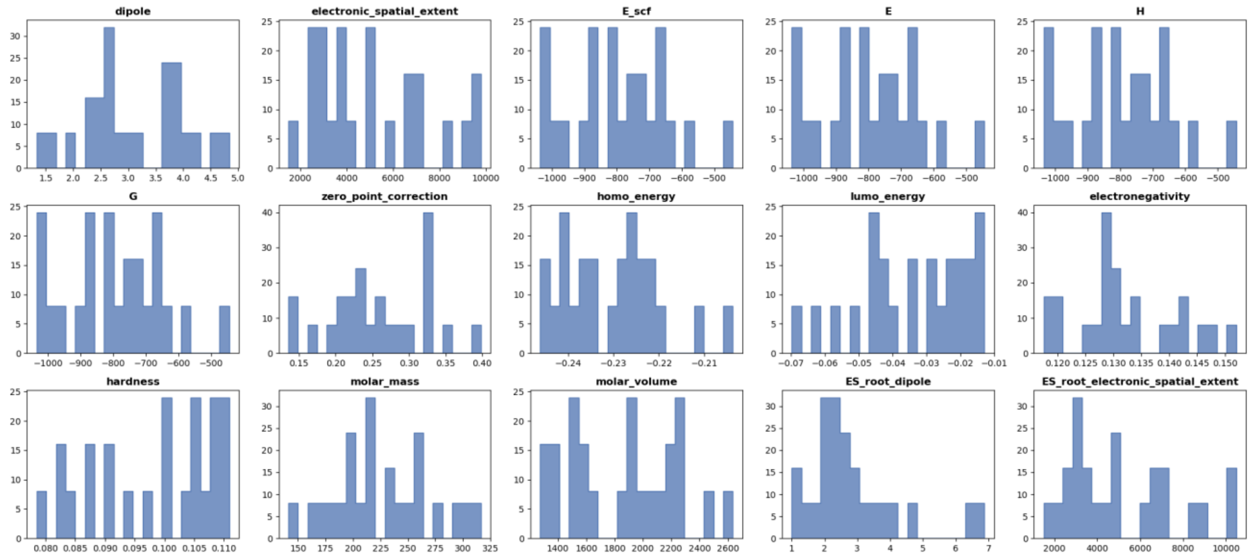

Figure S10. Histograms of 15 molecule-level descriptors across all reactions in the cyclic heldout dataset.

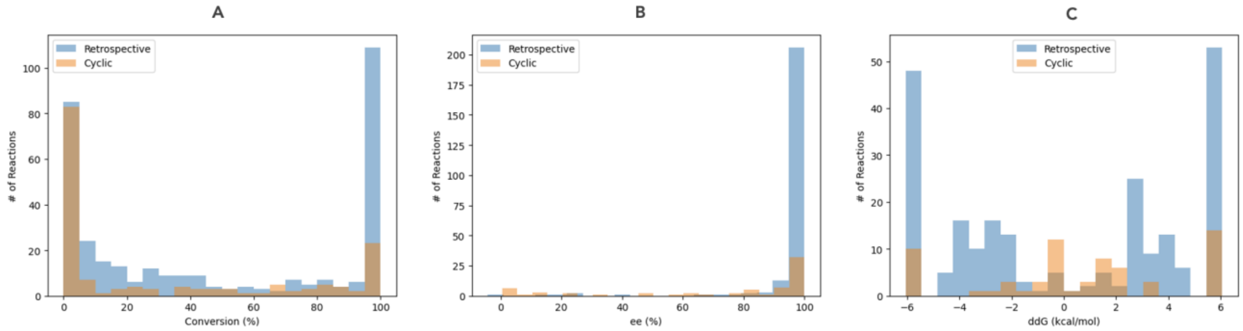

Figure S11. Distributions of (A) conversion, (B) enantiomeric excess, and (C) $\Delta\Delta G^{\ddagger}$ values across the cyclic heldout dataset (orange) as compared to the initial (retrospective) dataset (blue). For (C), note that positive values correspond to reactions yielding the S-product, while negative values correspond to reactions yielding the R-product. Note that the values for the cyclic heldout dataset were plotted after removing the diastereoselective substrates.

f. **Model Scoring and Evaluation**

This section details the calculation of the top-k accuracy and regret metrics used to score the models. We ask the questions: (1) for what fraction of ketones was the top-performing enzyme from experiment in the top k enzymes as predicted by the model? (2) What is the loss if I had gone with the top prediction?

For example, take the following ketone:

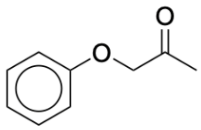

	ee	pred. $\Delta\Delta G^{\ddagger}$	
Enz 1	97.1	3.43	
Enz 2	98.7	2.58	
Enz 3	98.1	5.47	Failed top-1
Enz 4	99.9	4.99	Passed top-2
Enz 5	98.4	-3.60	
Enz 6	25.9	-1.77	
Enz 7	17.8	-2.18	
Enz 8	14.6	-1.89	

Figure S12. Example of top-k scoring for a ketone where all enzymes gave different selectivities experimentally. Ground truth ee values and MVLR-DFT model predictions in terms of $\Delta\Delta G^{\ddagger}$ are shown.

The highest-performing enzyme from experiment was Enz 4, with an ee of 99.9%. The highest-performing top-1 enzyme as predicted by the model is Enz 3, however, so the model fails top-1 prediction. (note that we take the absolute value of all $\Delta\Delta G^{\ddagger}$ predicted values since we are looking across R and S enzymes). However, the top-2 highest-performing enzymes by the model are Enz 3 and 4, so the model passes top-2 prediction. Here, the top-1 regret if we had gone with the model prediction of Enz 3, is 1.7% ee (99.9 - 98.1), or 1.81 kcal/mol in terms of $\Delta\Delta G^{\ddagger}$.

There exist ketones across the dataset where multiple enzymes gave the highest performance from experiment. For example, take the following ketone:

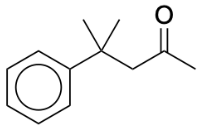

	ee	pred. $\Delta\Delta G^{\ddagger}$
Enz 1	100.0	6.03
Enz 2	100.0	7.11
Enz 3	98.4	5.37
Enz 4	100.0	5.51
Enz 5	93.4	-5.71
Enz 6	100.0	-6.04
Enz 7	100.0	-6.98
Enz 8	100.0	-7.60

Passes if at least 1 top-*k* prediction is in the highlighted set

Figure S13. Example of top-k scoring for a ketone where multiple enzymes gave the highest selectivity experimentally. Ground truth ee values and MVLR-DFT model predictions in terms of $\Delta\Delta G^{\ddagger}$ are shown.

The highest-performing enzymes from experiment were Enzymes 1, 2, 4, 6, 7, and 8, all giving 100% ee. The highest-performing top-1 enzyme as predicted by the model is Enz 8 (again note that we take the absolute value of all $\Delta\Delta G^{\ddagger}$ predicted values since we are looking across R and S enzymes). Since Enz 8 is within the set of best experimental enzymes, this ketone passes top-1 prediction. As such, the top-1 regret is 0.

In general, in these cases the model will pass top-k accuracy for a particular ketone if there is a set intersection size of at least 1 between the set of best experimental enzymes, and the top-k predicted enzymes from the model.

For the naive baseline, we take the top-k "model predictions" to be the k-highest performing enzymes across the entire dataset. This is calculated by finding, for each ketone, the top enzyme (or set of enzymes) from experiment. These enzymes are then collated across the dataset substrates and sorted in order of occurrence (the enzyme that appears most frequently would be the top-1 prediction in the naive case, the 2 most frequent enzymes would be the top-2 predictions, etc.).

For our initial dataset, the 4 highest-performing enzymes experimentally (from most to least frequent) were: Enz4, Enz2, Enz9, Enz7.

g. Additional Modelling Results on the Initial Dataset

As described in the main text, all results in this section represent per-enzyme LOOCV metrics across the initial dataset.

Conversion modeling results:

Model	Feature	Accuracy	AUROC	AUPRC	Precision	Recall
**RF**	OHE	0.592	0.493	0.542	0.603	0.804
	fp	0.717	0.734	0.714	0.726	0.799
	phys	0.664	0.719	0.755	0.698	0.709
	phys + DFT	0.708	0.743	0.738	0.724	0.778
	**DFT**	**0.735**	**0.789**	**0.758**	**0.745**	**0.804**

Table S1. Overall classification model metrics on the initial dataset using 5 different types of ketone features.

Enantioselectivity regression modeling results:

Model	Feature	Metric	Enz1	Enz2	Enz3	Enz4	Enz6	Enz7	Enz8	Enz9	Overall
**RF**	fp	R^2	-0.181	-0.208	0.027	-0.066	-0.307	-0.348	-0.347	0.037	-0.140
		PearsonR	0.045	0.016	0.229	0.234	-0.146	-0.117	-0.040	0.274	0.144
		MAE (kcal/mol)	1.605	1.430	1.141	1.300	1.320	1.739	2.096	1.655	1.530
		MAE (% ee)	2.582	1.419	1.948	1.461	1.370	5.928	9.225	6.573	3.801
	phys	R^2	-0.405	-0.138	-0.317	-0.074	-0.030	-0.186	-0.151	-0.094	-0.132
		PearsonR	-0.292	0.057	-0.273	0.141	0.161	-0.086	-0.041	0.048	0.050
		MAE (kcal/mol)	1.830	1.480	1.397	1.393	1.205	1.659	1.964	1.829	1.588
		MAE (% ee)	2.682	1.422	2.132	1.476	1.340	5.917	9.421	6.687	3.871
	phys + DFT	R^2	-0.174	0.094	-0.250	0.149	-0.033	-0.268	-0.148	-0.091	-0.062
		PearsonR	0.025	0.350	-0.167	0.397	0.167	-0.230	-0.046	0.089	0.150
		MAE (kcal/mol)	1.596	1.242	1.342	1.236	1.220	1.735	1.974	1.802	1.515
		MAE (% ee)	2.561	1.290	2.061	1.348	1.362	5.978	9.453	6.717	3.834
	DFT	R^2	0.024	0.248	-0.160	0.276	0.023	-0.215	0.022	0.075	0.066
		PearsonR	0.309	0.516	0.045	0.532	0.265	-0.068	0.256	0.337	0.332
		MAE (kcal/mol)	1.365	1.052	1.259	1.099	1.204	1.666	1.784	1.587	1.377
		MAE (% ee)	**2.391**	**1.165**	1.990	1.145	1.374	5.974	9.403	6.599	3.743
**MVLR**	**DFT + Sterimol**	**R^2**	**0.348**	**0.609**	**0.284**	**0.704**	**0.629**	**0.553**	**0.437**	**0.632**	**0.543**
		**PearsonR**	**0.616**	**0.786**	**0.553**	**0.840**	**0.805**	**0.760**	**0.665**	**0.801**	**0.745**
		**MAE (kcal/mol)**	**1.131**	**0.781**	**0.951**	**0.702**	**0.660**	**1.026**	**1.336**	**0.976**	**0.940**
		MAE (% ee)	3.512	1.314	**1.969**	**0.590**	**1.015**	**3.329**	**6.406**	**4.729**	**2.820**

Table S2. Per-enzyme and overall regression model metrics (for $\Delta\Delta G^{\ddagger}$) on the initial dataset using 5 different types of models and ketone feature combinations.

Note that for the overall metrics, both ground truth and predicted $\Delta\Delta G^{\ddagger}$ values were absolute-valued before computing the scores, to avoid inflation in R^2 due to our convention of positive $\Delta\Delta G^{\ddagger}$ for S-selective products and negative $\Delta\Delta G^{\ddagger}$ for R-selective products. The displayed MAE (% ee) values were obtained after converting the predicted $\Delta\Delta G^{\ddagger}$ values back to ee values.

Model	Feature	k = 1 Acc.	k = 2 Acc.	k = 3 Acc.	k = 4 Acc.
**naive**		0.636	0.818	0.848	0.909
**RF**	fp	0.515	0.636	0.848	0.970
	phys	0.667	0.758	0.818	0.909
	phys + DFT	0.606	0.879	**0.939**	0.939
	DFT	**0.697**	**0.909**	**0.939**	0.970
**MVLR**	**DFT + Sterimol**	**0.697**	0.848	**0.939**	**1.000**

Table S3. Top-*k* accuracy results for modelling on the initial dataset using 5 different types of models and ketone feature combinations. Results up to *k*=4 are included.

Sample Regression parity plots:

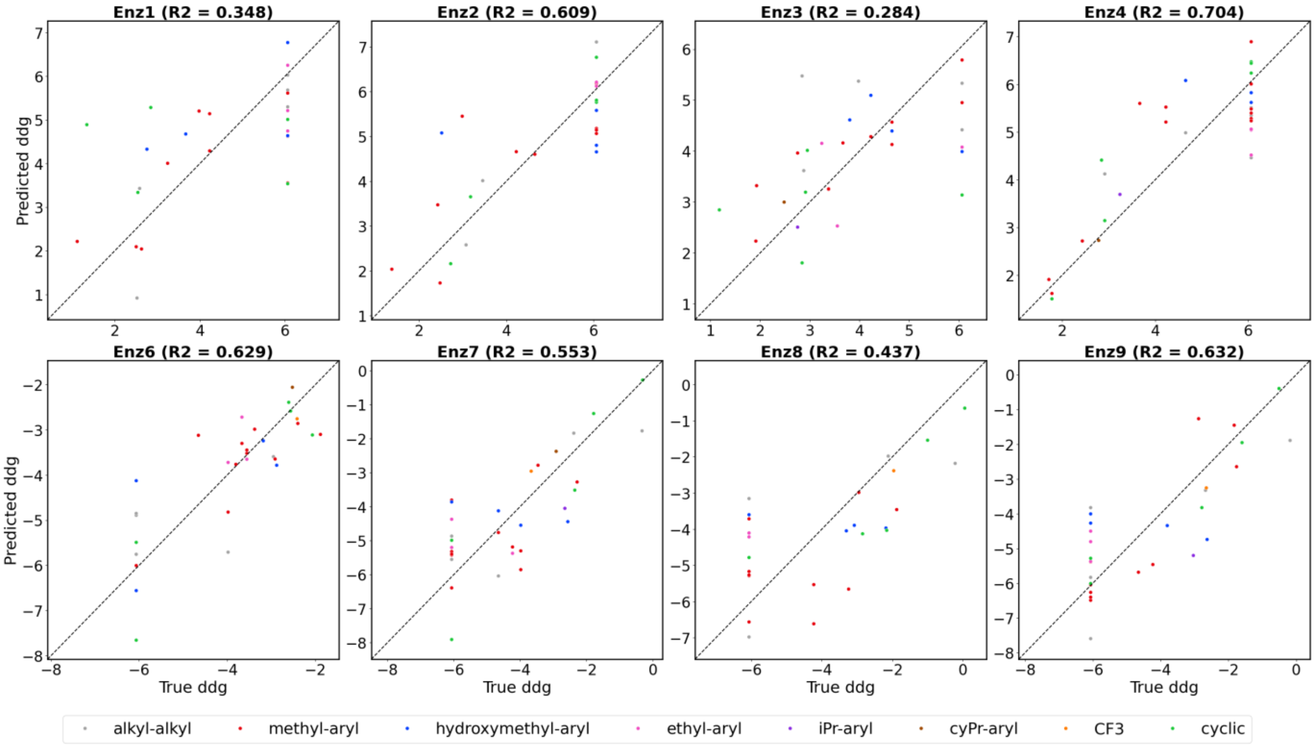

Figure S14. Per-enzyme regression parity plots (in terms of $\Delta\Delta G^{\ddagger}$) for the MVLR-DFT model on the initial dataset. Points are colored by the class of ketone.

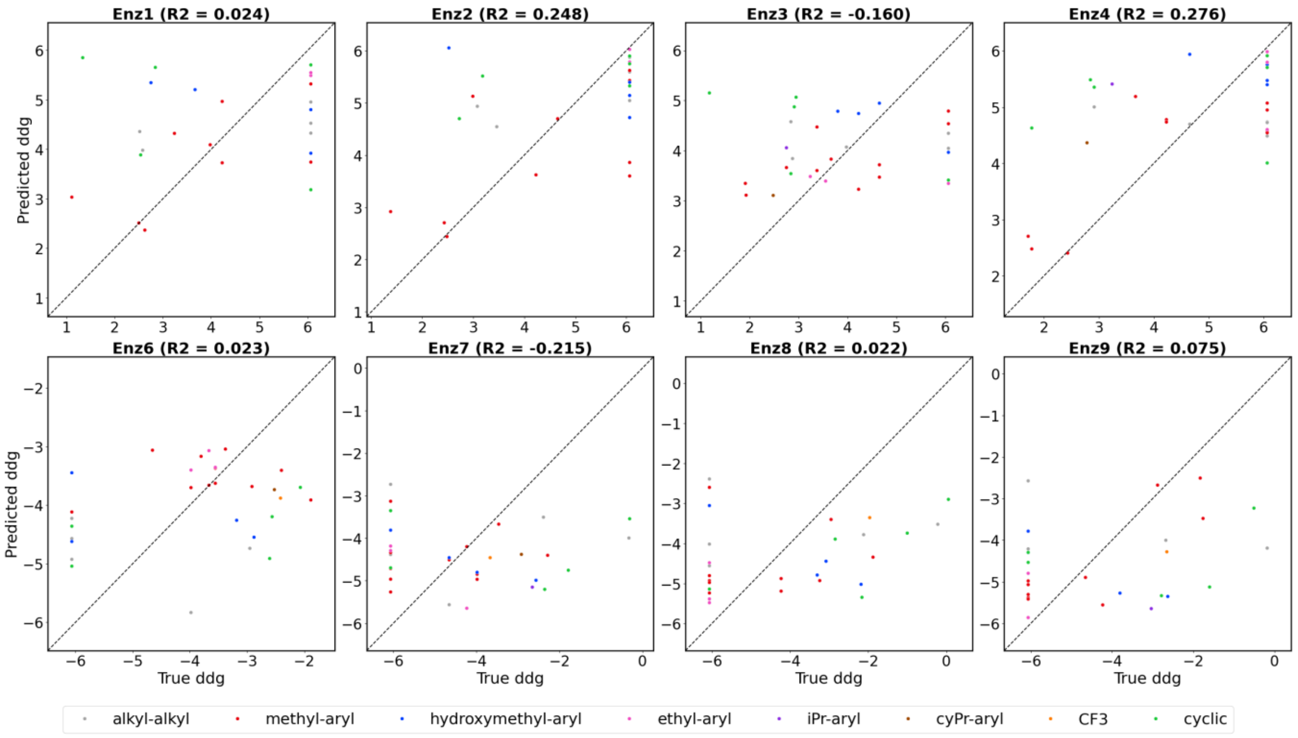

Figure S15. Per-enzyme regression parity plots (in terms of $\Delta\Delta G^{\ddagger}$) for the RF model using DFT features on the initial dataset. Points are colored by the class of ketone.

Selected features: The final selected features from the MVLR-DFT models for each enzyme are given in the following table. These features were then used to train full models on the initial dataset and subsequent inference on unseen data (i.e. the cyclic held-out dataset and scale-up prospective validation experiments).

	**Ketone features**
**Enz 1**	['VBur.Carbonyl', 'ES_root_dipole', 'B1.CC2', 'NPA_core.Carbon2', 'APT_charge.Carbon2']
**Enz 2**	['VBur.Carbonyl', 'NPA_Rydberg.Carbon2', 'E_scf', 'NPA_core.Carbon1', 'NMR_anisotropy.Carbon1', 'ES_root_electronic_spatial_extent', 'NMR_anisotropy.Carbonyl']
**Enz 3**	['NPA_Rydberg.Carbonyl', 'lumo_energy', 'B5.CC2', 'NPA_valence.Carbonyl']
**Enz 4**	['NPA_Rydberg.Carbon2', 'NPA_Rydberg.Carbonyl', 'NMR_anisotropy.Carbon1', 'NMR_anisotropy.Carbonyl', 'APT_charge.Carbon1', 'ES_root_NPA_Rydberg.Carbon2']
**Enz 6**	['APT_charge.Carbonyl', 'E_scf', 'NMR_anisotropy.Carbonyl', 'ES_root_NPA_core.Carbon1', 'VBur.Carbon1', 'ES_root_NPA_Rydberg.Carbonyl', 'dipole', 'APT_charge.Carbon2', 'lumo_energy']
**Enz 7**	['VBur.Carbon2', 'ES_root_NPA_core.Carbon1', 'NMR_anisotropy.Carbonyl', 'L.CC1', 'NMR_anisotropy.Carbon2', 'ES_root_NPA_core.Carbon2', 'B5.CC1', 'electronic_spatial_extent']
**Enz 8**	['VBur.Carbon2', 'NPA_core.Carbonyl', 'ES_root_NPA_core.Carbon1']
**Enz 9**	['VBur.Carbon2', 'ES_root_NPA_core.Carbonyl', 'ES_root_NPA_valence.Carbon2', 'NMR_anisotropy.Carbon2', 'homo_energy', 'ES_root_NPA_core.Carbon1']

For the conversion modeling, the most important ketone DFT feature for each enzyme, as found from the native Random Forest feature importances, are listed below:

Enz1 VBur.Carbon1

Enz2 NPA_valence.Carbon1

Enz3 APT_charge.Carbonyl

Enz4 NPA_valence.Carbon1

Enz6 APT_charge.Carbon2

Enz7 NPA_valence.Carbon1

Enz8 APT_charge.Carbonyl

Enz9 NPA_valence.Carbon1

3. Supplementary Figures

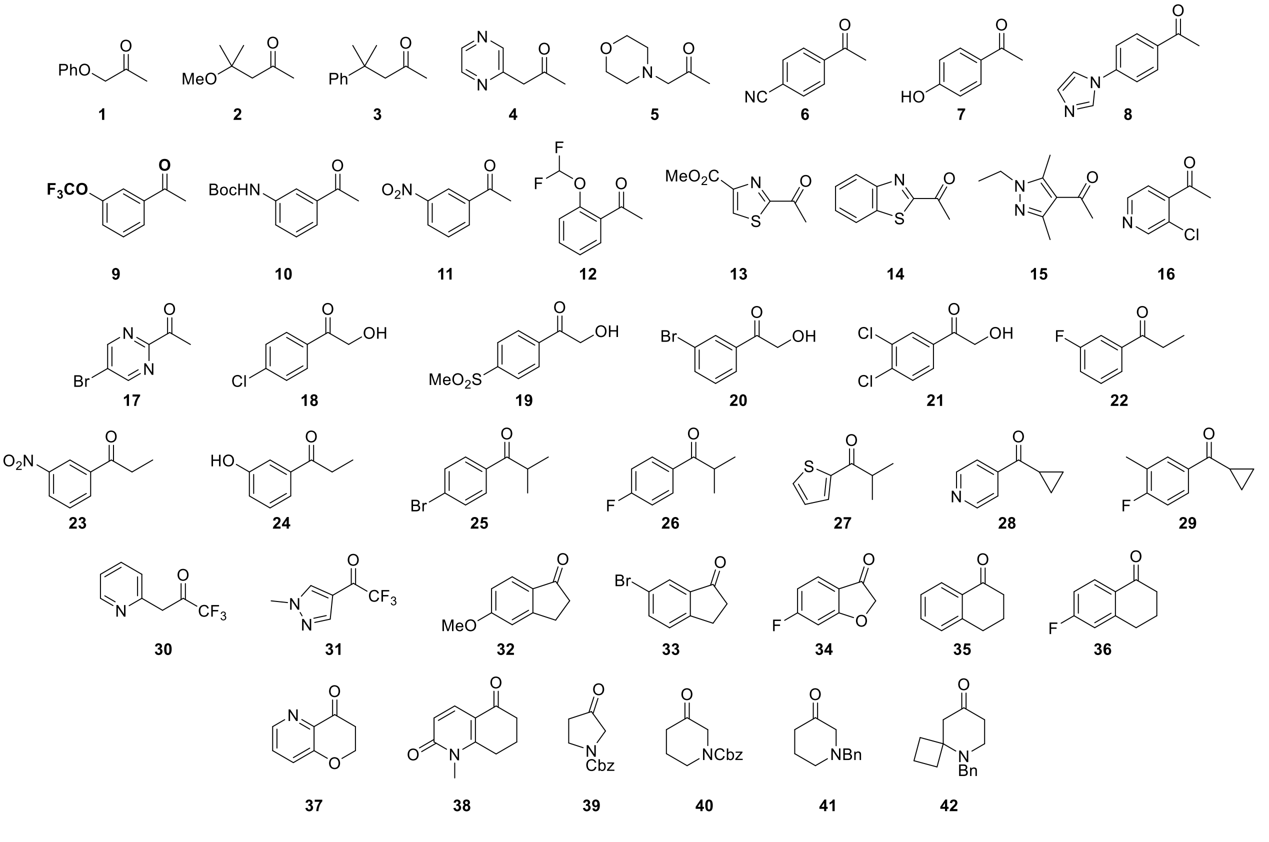

Figure S16. Structures of Ketone Substrates (HTE Dataset)

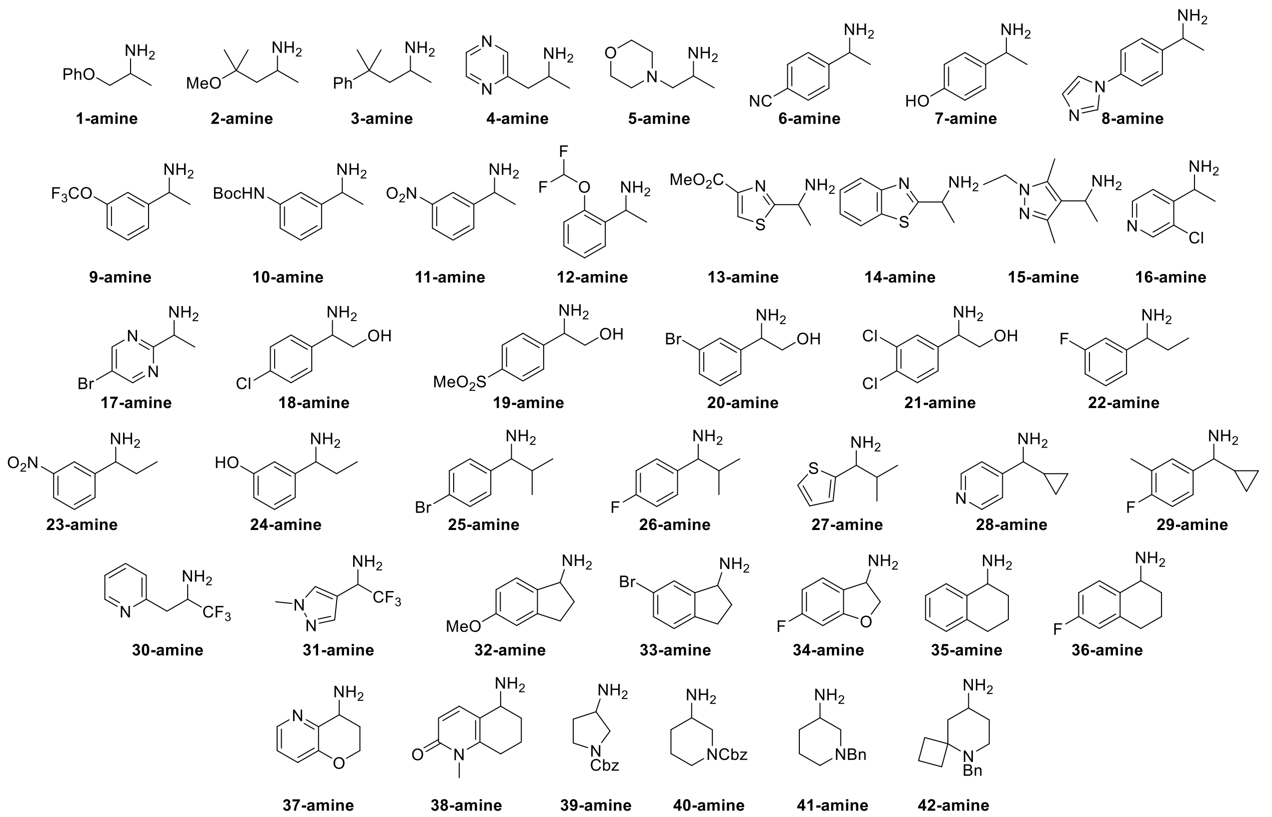

Figure S17. Structures of Amine Products (HTE Dataset)

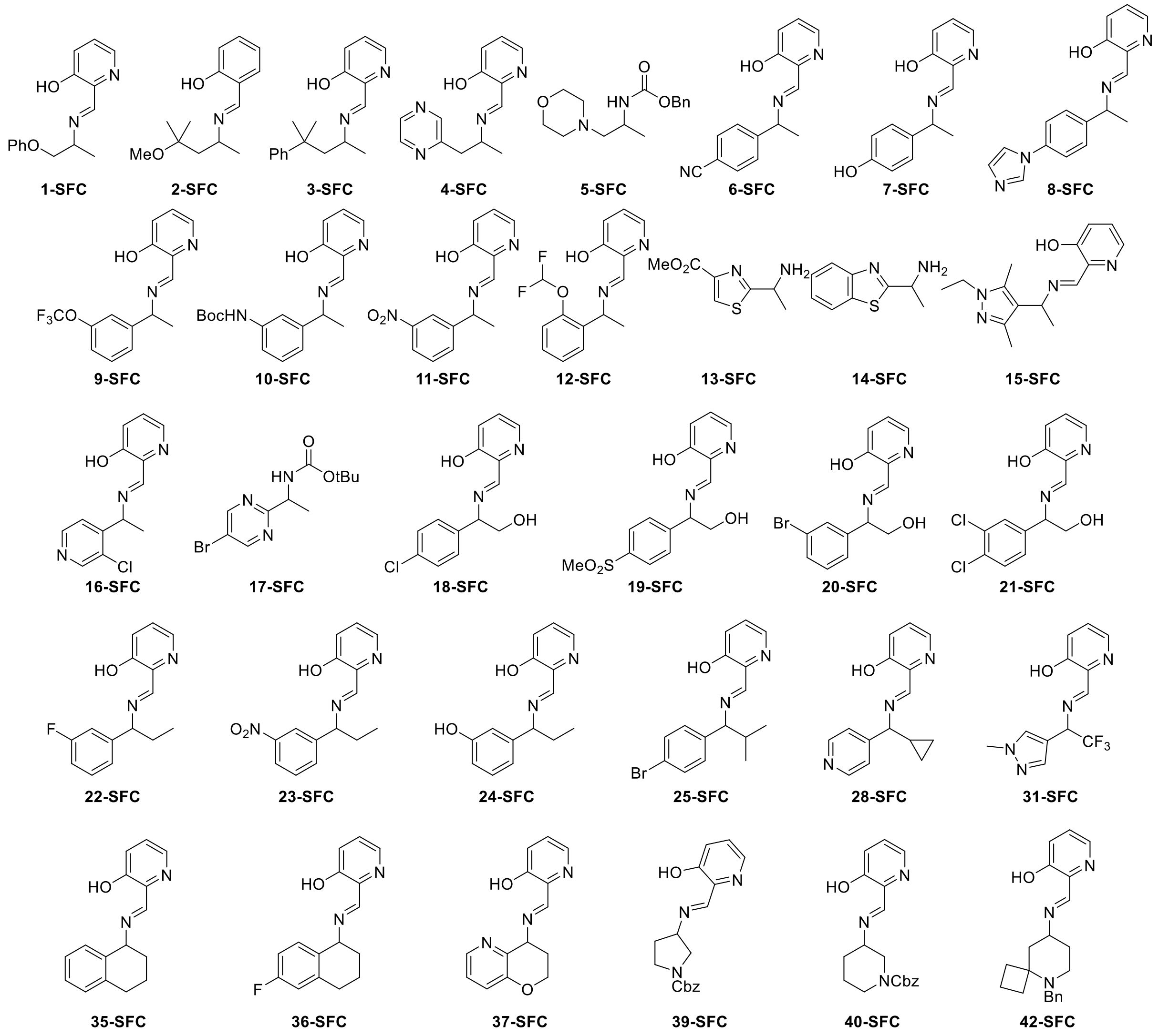

Figure S18. Structures of Compounds Used for *ee* Measurement (HTE Dataset)

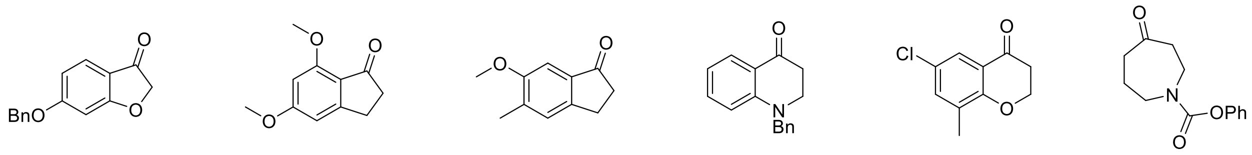

43 44 45 46 47 48

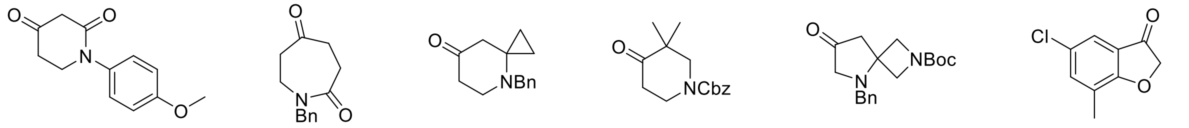

49 50 51 52 53 54

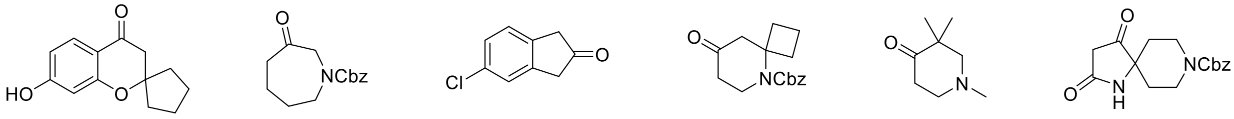

55 56 57 58 59 60

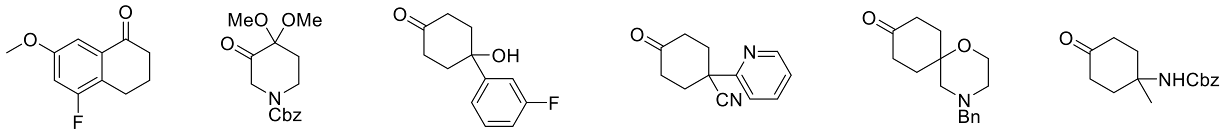

61 62 63 64 65 66

Note: Compounds **53**,[5] **55**,[6] and **59**[7] were prepared according to previously reported procedures.

Figure S19. Structures of Ketone Substrates (held-out dataset)

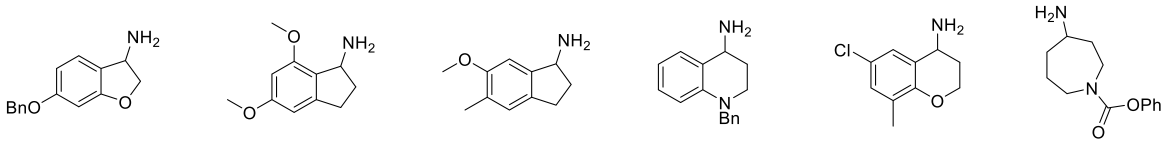

43-amine 44-amine 45-amine 46-amine 47-amine 48-amine

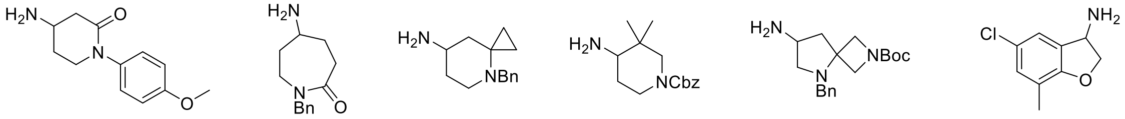

49-amine 50-amine 51-amine 52-amine 53-amine 54-amine

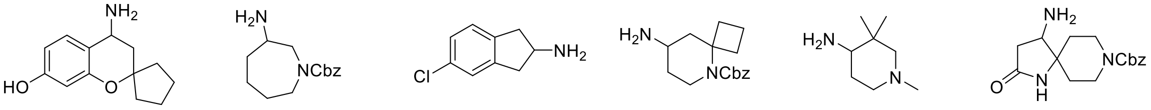

55-amine 56-amine 57-amine 58-amine 59-amine 60-amine

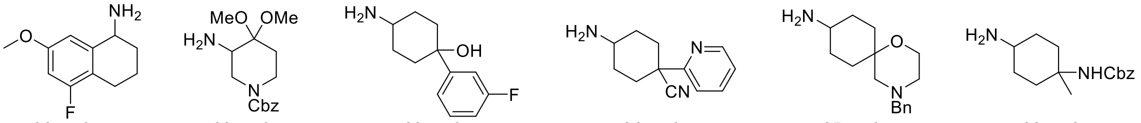

61-amine 62-amine 63-amine 64-amine 65-amine 66-amine

Figure S20. Structures of Amine Products (held-out dataset)

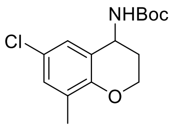

47-SFC

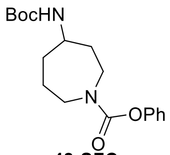

48-SFC

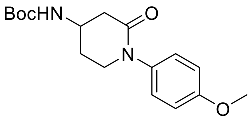

49-SFC

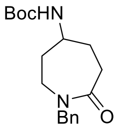

50-SFC

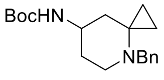

51-SFC

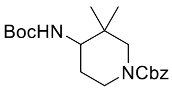

52-SFC

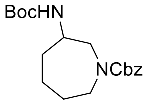

56-SFC

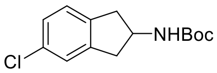

57-SFC

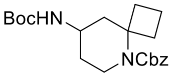

58-SFC

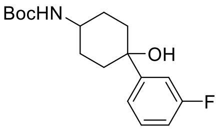

63-SFC

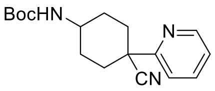

64-SFC

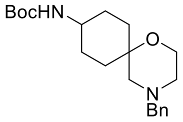

65-SFC

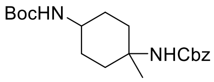

66-SFC

Figure S21. Structures of compounds Used for *ee* Measurement (held-out dataset)

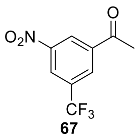

67

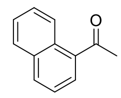

68

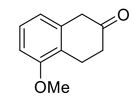

69

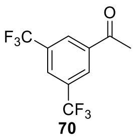

70

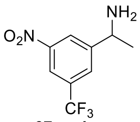

67-amine

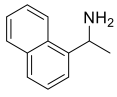

68-amine

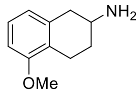

69-amine

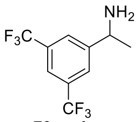

70-amine

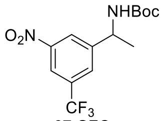

67-SFC

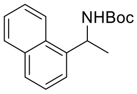

68-SFC

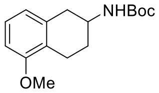

69-SFC

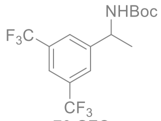

70-SFC

Figure S22. Structures of Scale-Up compounds

Table S4. HTE results comparing *ee* measurements using hydroxypyridyl imines or directly measuring amine *ee*

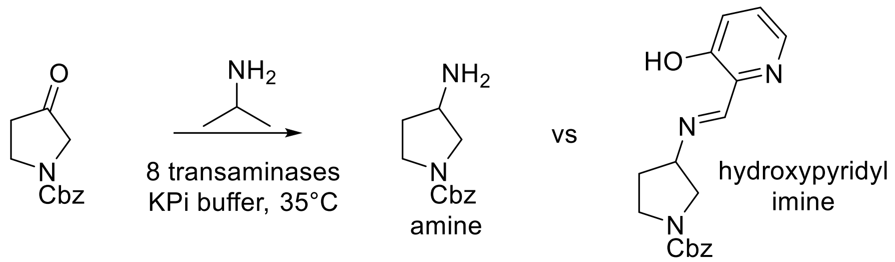

Enzyme	Hydroxypyridyl imine *ee*	Amine *ee*
ATA-026	96.9%	97.0%
ATA-031	97.7%	97.6%
ATA-237	98.3%	98.2%
ATA-S125	89.6%	89.8%
ATA-P2-A07	97.2%	98.0%
ATA-025	24.6%	25.6%
ATA-415	-4.2%	-4.4%
ATA-R123	39.2%	31.8%

Note: We have observed variations in the measured ee of less-selective substrate-enzyme pairs in reactions conducted at different times, such as that observed with ATA-123. Moreover, the peak shape of the direct amine SFC measurements are broad and can be impacted by the presence of salts, further complicating the determination of less-selective reactions. Due to the good alignment of the measured *ee* values from this exercise (<1% difference in measured *ee* for most), we proceeded forward using derivatized amines to generate the *ee* data for the study.

Table S5. Variation across multiple SFC injections on measured *ee* values (6-SFC)

Enzyme	Injection 1 *ee*	Injection 2 *ee*	Injection 3 *ee*	Average *ee*	Std. Dev.
ATA-026	99.84%	99.83%	99.81%	99.82%	0.02%
ATA-031	99.94%	99.95%	99.94%	99.94%	0.002%
ATA-237	99.52%	99.54%	99.52%	99.52%	0.01%
ATA-S125	>99.99%	>99.99%	99.97%	99.99%	0.02%
ATA-P2-A07	95.97%	96.11%	96.22%	96.10%	0.12%
ATA-025	99.82%	99.84%	99.84%	99.83%	0.01%
ATA-415	98.95%	98.96%	98.95%	98.96%	0.001%
ATA-R123	99.85%	99.85%	99.84%	99.85%	0.01%

Note: Low variation was introduced by the SFC instrument across multiple injections, with the largest error observed being ~0.13% RSD.

Table S6. Micro-scale results from 5-methoxy-3,4-dihydronaphthalen-2(1H)-one

Enzyme	Peak 1 area (1.53 min)	Peak 2 area (1.73 min)	*ee*
ATA-026	1045189	640265	24%
ATA-031	967962	729303	14.1%
ATA-237	1089549	590017	29.7%
ATA-S125	615970	795196	12.7%
ATA-P2-A07	268682	1056773	59.5%
ATA-025	816520	915961	5.7%
ATA-415	589178	650500	4.9%
ATA-R123	532254	772324	18.4%

Note: we have observed differences in ee when low-selectivity substrates were scaled-up. These were not observed with highly-selective substrates.

Table S7. Conversion variation across one sample set

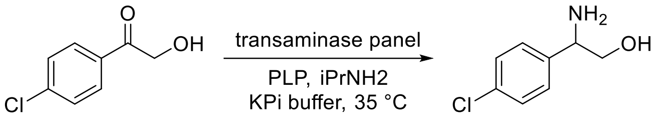

Trial	ATA-026	ATA-031	ATA-237	ATA-S125	ATA-P2-A07	ATA-025	ATA-415	ATA-R123
1	89	79	94	99	94	94	92	91
2	92	77	98	100	93	96	87	92
3	93	88	99	100	98	100	96	95
Avg	88	81.3	97	99.7	95	96.7	91.7	92.7
StDev	5.6	5.9	2.6	0.6	2.6	3.1	4.5	2.1

Figure S23. Confusion matrix showing performance of the conversion modelling on the held-out set.

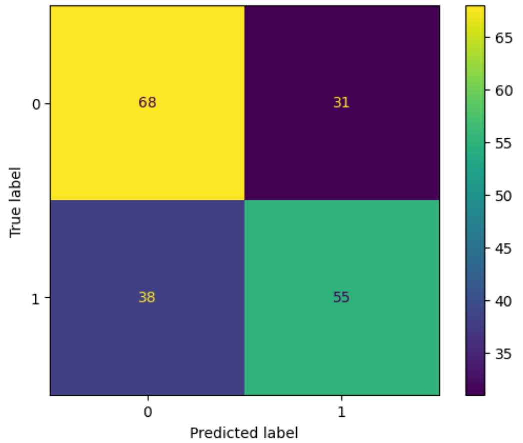

Accuracy: 0.64

Precision: 0.64

Recall: 0.59

AUROC: 0.71

AUPRC: 0.65

Baseline positive rate: 0.48

Table S8. Comparison of held-out dataset's original modelling results to those obtained with random noise added to the conversion/ee measurements

Noise type	Ideal Predictions	Acceptable Predictions	Poor Predictions
None (original results)	12	7	5
Random ±1% to ee<br>Random ± 5% to conversion (trial 1)	12	7	5
Random ±1% to ee<br>Random ± 5% to conversion (trial 2)	12	7	5
Random ±1% to ee<br>Random ± 5% to conversion (trial 3)	12	7	5

Table S9. Comparison of different feature types for held-out dataset enzyme selection

Feature Class	Ideal Predictions	Acceptable Predictions	Poor Predictions
MVLR DFT (original results)	12	7	5
Fingerprints	9	2	13
Naïve (top 2 enzymes from initial HTE)	8	1	15

Table S10. Comparison of (S)- and (R)-selective enzyme results from the held-out dataset (*ee* substrates only)

Enzyme Class	Ideal Predictions	Acceptable Predictions	Poor Predictions
All Enzymes<br>(Figure 5 results)	11	6	3
(*S*)-Selective	12	3	5
(*R*)-Selective	13	4	3

a. SFC Methods and Traces

Note: A handful of SFC samples were injected in triplicate, and the resulting ee measurements were averaged in the dataset. As little variation from the instrument was found, only one representative trace from each datapoint in the ee dataset is shown.

Table S11. Summary of column, co-solvent, and elution conditions used for SFC analysis

ID	Derivatized Amine Substrate	Column Name	Co-solvent (B solvent)	Elution Conditions: Time (min)	%CO_2	%B
1-SFC[a] (Method A)	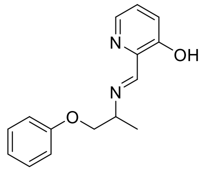	ChiralCel OD-3	Methanol 0.1% Diethylamine	Initial	99	1
				0.10	99	1
				6.00	90	10
				7.00	90	10
				7.10	50	50
				9.10	50	50
				9.11	99	1
				10.00	99	1
1-SFC[a] (Method B)	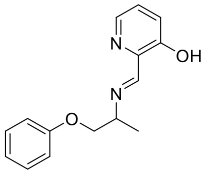	ChiralCel OD-3	Methanol 0.1% Diethylamine	Initial	95	5
				0.10	95	5
				7.00	70	30
				8.00	70	30
				8.10	50	50
				9.10	50	50
				9.11	95	5
				10.00	95	5
2-SFC[a]	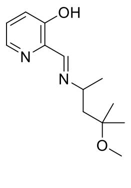	ChiralPak IG-3	Methanol 0.1% Diethylamine	Initial	99	1
				0.10	99	1
				6.00	90	10
				7.00	90	10
				7.10	50	50
				9.10	50	50
				9.11	99	1
				10.00	99	1
3-SFC[a]	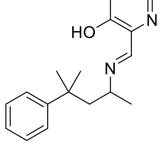	ChiralPak IG-3	Methanol 0.1% Diethylamine	Initial	99	1
				0.10	99	1
				7.10	50	50
				9.10	50	50
				9.11	99	1
				10.00	99	1

4-SFC[a]	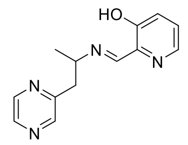	ChiralPak IA-3	Isopropanol 0.1% Diethylamine	Time (min) / $\%CO_2$ / %B<br>Initial / 99 / 1<br>0.10 / 99 / 1<br>7.00 / 90 / 10<br>8.00 / 90 / 10<br>8.10 / 50 / 50<br>9.10 / 50 / 50<br>9.11 / 99 / 1<br>10.00 / 99 / 1
5-SFC[a]	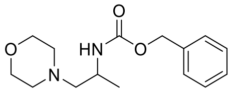	ChiralPak IG-3	Isopropanol 0.1% Diethylamine	Time (min) / $\%CO_2$ / %B<br>Initial / 95 / 5<br>10.00 / 95 / 5
6-SFC[a]	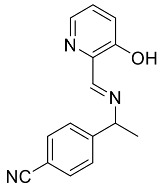	ChiralPak IG-3	Methanol 0.1% Diethylamine	Time (min) / $\%CO_2$ / %B<br>Initial / 99 / 1<br>0.10 / 99 / 1<br>7.10 / 50 / 50<br>9.10 / 50 / 50<br>9.11 / 99 / 1<br>10.00 / 99 / 1
7-SFC[a]	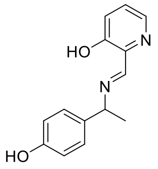	ChiralCel OZ-3	Methanol 0.1% Diethylamine	Time (min) / $\%CO_2$ / %B<br>Initial / 99 / 1<br>0.10 / 99 / 1<br>7.10 / 50 / 50<br>9.10 / 50 / 50<br>9.11 / 99 / 1<br>10.00 / 99 / 1
8-SFC[a]	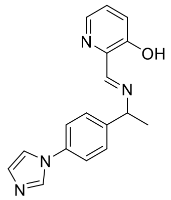	ChiralCel OD-3	Methanol 0.1% Diethylamine	Time (min) / $\%CO_2$ / %B<br>Initial / 99 / 1<br>0.10 / 99 / 1<br>7.10 / 50 / 50<br>9.10 / 50 / 50<br>9.11 / 99 / 1<br>10.00 / 99 / 1
9-SFC[a]	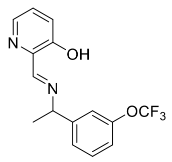	ChiralPak IG-3	Methanol 0.1% Diethylamine	Time (min) / $\%CO_2$ / %B<br>Initial / 99 / 1<br>0.10 / 99 / 1<br>6.00 / 90 / 10<br>7.00 / 90 / 10<br>7.10 / 50 / 50<br>9.10 / 50 / 50<br>9.11 / 99 / 1<br>10.00 / 99 / 1

<table>
<tr><td>10-SFC[a]</td><td>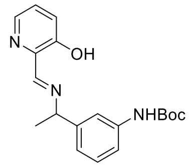</td><td>ChiralPak AD-3</td><td>Methanol 0.1% Diethylamine</td><td>
<table>
<tr><th>Time (min)</th><th>%CO_2</th><th>%B</th></tr>
<tr><td>Initial</td><td>99</td><td>1</td></tr>
<tr><td>0.10</td><td>99</td><td>1</td></tr>
<tr><td>7.10</td><td>50</td><td>50</td></tr>
<tr><td>9.10</td><td>50</td><td>50</td></tr>
<tr><td>9.11</td><td>99</td><td>1</td></tr>
<tr><td>10.00</td><td>99</td><td>1</td></tr>
</table>
</td></tr>
<tr><td>11-SFC[a]</td><td>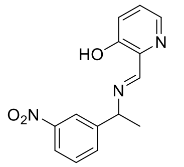</td><td>ChiralCel OZ-3</td><td>Isopropanol 0.1% Diethylamine</td><td>
<table>
<tr><th>Time (min)</th><th>%CO_2</th><th>%B</th></tr>
<tr><td>Initial</td><td>99</td><td>1</td></tr>
<tr><td>0.10</td><td>99</td><td>1</td></tr>
<tr><td>7.10</td><td>50</td><td>50</td></tr>
<tr><td>9.10</td><td>50</td><td>50</td></tr>
<tr><td>9.11</td><td>99</td><td>1</td></tr>
<tr><td>10.00</td><td>99</td><td>1</td></tr>
</table>
</td></tr>
<tr><td>12-SFC[a]</td><td>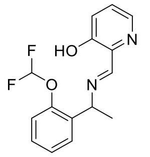</td><td>ChiralCel OZ-3</td><td>Methanol 0.1% Diethylamine</td><td>
<table>
<tr><th>Time (min)</th><th>%CO_2</th><th>%B</th></tr>
<tr><td>Initial</td><td>99</td><td>1</td></tr>
<tr><td>0.10</td><td>99</td><td>1</td></tr>
<tr><td>6.00</td><td>90</td><td>10</td></tr>
<tr><td>7.00</td><td>90</td><td>10</td></tr>
<tr><td>7.10</td><td>50</td><td>50</td></tr>
<tr><td>9.10</td><td>50</td><td>50</td></tr>
<tr><td>9.11</td><td>99</td><td>1</td></tr>
<tr><td>10.00</td><td>99</td><td>1</td></tr>
</table>
</td></tr>
<tr><td>13-SFC[a]</td><td>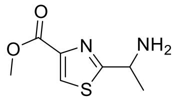</td><td>ChiralCel OZ-3</td><td>Ethanol 190
0.1% Diethylamine</td><td>
<table>
<tr><th>Time (min)</th><th>Flow Rate (mL/min)</th><th>%CO_2</th><th>%B</th></tr>
<tr><td>Initial</td><td>2</td><td>99</td><td>1</td></tr>
<tr><td>0.50</td><td>2</td><td>99</td><td>1</td></tr>
<tr><td>7.10</td><td>2</td><td>70</td><td>30</td></tr>
<tr><td>8.10</td><td>2</td><td>70</td><td>30</td></tr>
<tr><td>8.11</td><td>2</td><td>50</td><td>50</td></tr>
<tr><td>9.12</td><td>2</td><td>50</td><td>50</td></tr>
<tr><td>9.13</td><td>2</td><td>99</td><td>1</td></tr>
<tr><td>10.00</td><td>2</td><td>99</td><td>1</td></tr>
</table>
</td></tr>
<tr><td>14-SFC[a]</td><td>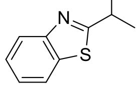</td><td>ChiralPak IG-3</td><td>Ethanol 190
0.1% Diethylamine</td><td>
<table>
<tr><th>Time (min)</th><th>%CO_2</th><th>%B</th></tr>
<tr><td>Initial</td><td>99</td><td>1</td></tr>
<tr><td>0.10</td><td>99</td><td>1</td></tr>
<tr><td>7.10</td><td>60</td><td>40</td></tr>
<tr><td>9.10</td><td>60</td><td>40</td></tr>
<tr><td>9.11</td><td>99</td><td>1</td></tr>
<tr><td>10.00</td><td>99</td><td>1</td></tr>
</table>
</td></tr>
</table>

15-SFC[a]	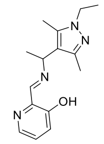	ChiralPak IG-3	Methanol 0.1% Diethylamine	Time (min) | $\%CO_2$ | %B<br>Initial | 99 | 1<br>0.10 | 99 | 1<br>7.10 | 50 | 50<br>9.10 | 50 | 50<br>9.11 | 99 | 1<br>10.00 | 99 | 1
16-SFC[a]	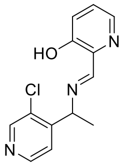	ChiralCel OD-3	Methanol 0.1% Diethylamine	Time (min) | $\%CO_2$ | %B<br>Initial | 99 | 1<br>0.10 | 99 | 1<br>6.00 | 90 | 10<br>7.00 | 90 | 10<br>7.10 | 50 | 50<br>9.10 | 50 | 50<br>9.11 | 99 | 1<br>10.00 | 99 | 1
17-SFC[a]	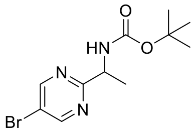	ChiralPak IG-3	Ethanol 190<br>0.1% Diethylamine	Time (min) | $\%CO_2$ | %B<br>Initial | 95 | 5<br>2.5 | 95 | 5
18-SFC[a]	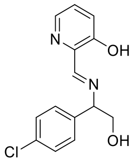	ChiralPak IG-3	Isopropanol 0.1% Diethylamine	Time (min) | $\%CO_2$ | %B<br>Initial | 99 | 1<br>0.10 | 99 | 1<br>7.10 | 50 | 50<br>9.10 | 50 | 50<br>9.11 | 99 | 1<br>10.00 | 99 | 1
19-SFC[a]	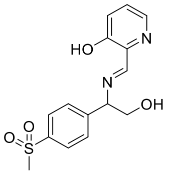	ChiralPak IG-3	Isopropanol 0.1% Diethylamine	Time (min) | $\%CO_2$ | %B<br>Initial | 99 | 1<br>0.10 | 99 | 1<br>7.10 | 50 | 50<br>9.10 | 50 | 50<br>9.11 | 99 | 1<br>10.00 | 99 | 1
20-SFC[a]	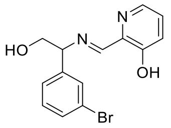	ChiralCel OD-3	Methanol 0.1% Diethylamine	Time (min) | $\%CO_2$ | %B<br>Initial | 99 | 1<br>0.10 | 99 | 1<br>6.00 | 90 | 10<br>7.00 | 90 | 10<br>7.10 | 50 | 50<br>9.10 | 50 | 50<br>9.11 | 99 | 1<br>10.00 | 99 | 1

Entry	Structure	Column	Co-solvent	Time (min)	$\%CO_2$	%B
21-SFC[a]	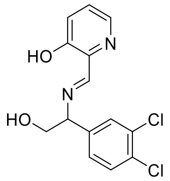	ChiralPak IG-3	Isopropanol 0.1% Diethylamine	Initial	99	1
				0.10	99	1
				7.10	50	50
				9.10	50	50
				9.11	99	1
				10.00	99	1
22-SFC[a]	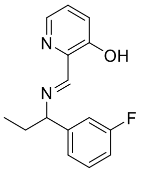	ChiralPak IG-3	Methanol 0.1% Diethylamine	Initial	99	1
				0.10	99	1
				6.00	90	10
				7.00	90	10
				7.10	50	50
				9.10	50	50
				9.11	99	1
				10.00	99	1
23-SFC[a]	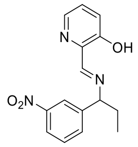	ChiralPak IG-3	Methanol 0.1% Diethylamine	Initial	95	5
				0.10	95	5
				7.00	70	30
				8.00	70	30
				8.10	50	50
				9.10	50	50
				9.11	95	5
				10.00	95	5
24-SFC[a]	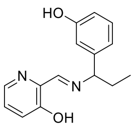	ChiralPak IG-3	Methanol 0.1% Diethylamine	Initial	99	1
				0.10	99	1
				7.10	50	50
				9.10	50	50
				9.11	99	1
				10.00	99	1
25-SFC[a]	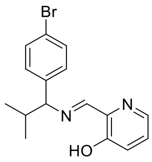	ChiralPak IG-3	Methanol 0.1% Diethylamine	Initial	99	1
				0.10	99	1
				7.10	50	50
				9.10	50	50
				9.11	99	1
				10.00	99	1
28-SFC[a]	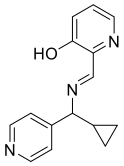	ChiralCel OZ-3	Methanol 0.1% Diethylamine	Initial	99	1
				0.10	99	1
				7.10	50	50

<table>
<tr><td></td><td></td><td></td><td></td><td><table><tr><td>9.10</td><td>50</td><td>50</td></tr><tr><td>9.11</td><td>99</td><td>1</td></tr><tr><td>10.00</td><td>99</td><td>1</td></tr></table></td></tr>
<tr><td>31-SFC[a]</td><td>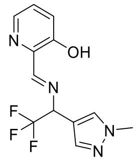</td><td>ChiralPak IG-3</td><td>Isopropanol 0.1% Diethylamine</td><td><table><tr><th>Time (min)</th><th>%CO_2</th><th>%B</th></tr><tr><td>Initial</td><td>99</td><td>1</td></tr><tr><td>0.10</td><td>99</td><td>1</td></tr><tr><td>7.10</td><td>50</td><td>50</td></tr><tr><td>9.10</td><td>50</td><td>50</td></tr><tr><td>9.11</td><td>99</td><td>1</td></tr><tr><td>10.00</td><td>99</td><td>1</td></tr></table></td></tr>
<tr><td>35-SFC[a]</td><td>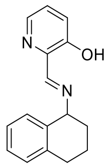</td><td>ChiralPak IG-3</td><td>Methanol 0.1% Diethylamine</td><td><table><tr><th>Time (min)</th><th>%CO_2</th><th>%B</th></tr><tr><td>Initial</td><td>99</td><td>1</td></tr><tr><td>0.10</td><td>99</td><td>1</td></tr><tr><td>7.10</td><td>50</td><td>50</td></tr><tr><td>9.10</td><td>50</td><td>50</td></tr><tr><td>9.11</td><td>99</td><td>1</td></tr><tr><td>10.00</td><td>99</td><td>1</td></tr></table></td></tr>
<tr><td>36-SFC[a]</td><td>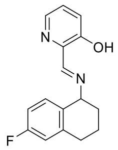</td><td>ChiralPak IG-3</td><td>Methanol 0.1% Diethylamine</td><td><table><tr><th>Time (min)</th><th>%CO_2</th><th>%B</th></tr><tr><td>Initial</td><td>99</td><td>1</td></tr><tr><td>0.10</td><td>99</td><td>1</td></tr><tr><td>7.10</td><td>50</td><td>50</td></tr><tr><td>9.10</td><td>50</td><td>50</td></tr><tr><td>9.11</td><td>99</td><td>1</td></tr><tr><td>10.00</td><td>99</td><td>1</td></tr></table></td></tr>
<tr><td>37-SFC[a]</td><td>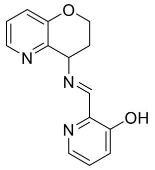</td><td>ChiralPak IC-3</td><td>Isopropanol 0.1% Diethylamine</td><td><table><tr><th>Time (min)</th><th>%CO_2</th><th>%B</th></tr><tr><td>Initial</td><td>99</td><td>1</td></tr><tr><td>0.10</td><td>99</td><td>1</td></tr><tr><td>7.10</td><td>50</td><td>50</td></tr><tr><td>9.10</td><td>50</td><td>50</td></tr><tr><td>9.11</td><td>99</td><td>1</td></tr><tr><td>10.00</td><td>99</td><td>1</td></tr></table></td></tr>
<tr><td>39-SFC[a]</td><td>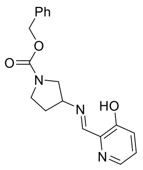</td><td>ChiralPak IG-3</td><td>Methanol 0.1% Diethylamine</td><td><table><tr><th>Time (min)</th><th>%CO_2</th><th>%B</th></tr><tr><td>Initial</td><td>99</td><td>1</td></tr><tr><td>0.10</td><td>99</td><td>1</td></tr><tr><td>7.10</td><td>50</td><td>50</td></tr><tr><td>9.10</td><td>50</td><td>50</td></tr><tr><td>9.11</td><td>99</td><td>1</td></tr><tr><td>10.00</td><td>99</td><td>1</td></tr></table></td></tr>
<tr><td>40-SFC[a]</td><td>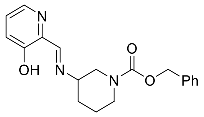</td><td>ChiralPak IA-3</td><td>Methanol 0.1% Diethylamine</td><td><table><tr><th>Time (min)</th><th>%CO_2</th><th>%B</th></tr><tr><td>Initial</td><td>99</td><td>1</td></tr><tr><td>0.10</td><td>99</td><td>1</td></tr><tr><td>7.10</td><td>50</td><td>50</td></tr><tr><td>9.10</td><td>50</td><td>50</td></tr></table></td></tr>
</table>

				9.11	99	1
				10.00	99	1
42-SFC[a]	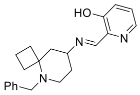	ChiralPak AD-3	Methanol 0.1% Diethylamine	**Time (min)**	**%CO_2**	**%B**
				Initial	99	1
				0.10	99	1
				7.10	50	50
				9.10	50	50
				9.11	99	1
				10.00	99	1
47-SFC[b]	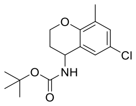	ChiralPak IH-3	Ethanol 190 0.1% Diethylamine	**Time (min)**	**%CO_2**	**%B**
				Initial	97.0	3.0
				0.25	97.0	3.0
				2.50	90	10
				3.00	90	10
				3.01	50	50
				3.70	50	50
				3.71	97.0	3.0
				4.00	97.0	3.0
48-SFC[b]	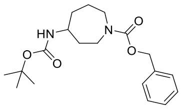	ChiralPak AD-3	Ethanol 190 0.1% Diethylamine	**Time (min)**	**%CO_2**	**%B**
				Initial	95	5
				0.25	95	5
				4.00	50	50
				4.70	50	50
				4.71	95	5
				5.00	95	5
49-SFC[b]	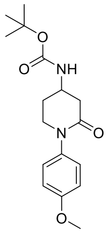	ChiralPak IH-3	Ethanol 190 0.1% Diethylamine	**Time (min)**	**%CO_2**	**%B**
				Initial	95	5
				0.25	95	5
				4.00	50	50
				4.70	50	50
				4.71	95	5
				5.00	95	5
50-SFC[c]	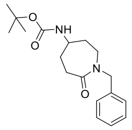	ChiralPak IG-3	Methanol 0.1% Diethylamine	**Time (min)**	**%CO_2**	**%B**
				Initial	97.0	3.0
				0.25	97.0	3.0
				3.00	40.0	60.0
				3.70	40.0	60.0
				3.71	97.0	3.0
				4.00	97.0	3.0
52-SFC[b]	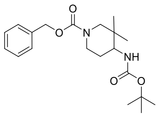	ChiralPak IG-3	Methanol 0.1% Diethylamine	**Time (min)**	**%CO_2**	**%B**
				Initial	95	5
				0.25	95	5
				2.50	70	30

<table>
<tr><td></td><td></td><td></td><td></td><td><table>
<tr><td>3.00</td><td>70</td><td>30</td></tr>
<tr><td>3.01</td><td>50</td><td>50</td></tr>
<tr><td>3.70</td><td>50</td><td>50</td></tr>
<tr><td>3.71</td><td>95</td><td>5</td></tr>
<tr><td>4.00</td><td>95</td><td>5</td></tr>
</table></td></tr>
<tr><td>56-SFC[b]</td><td>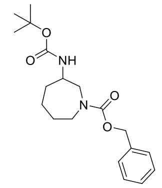</td><td>ChiralPak AD-3</td><td>Isopropanol 0.1% Diethylamine</td><td><table>
<tr><th>Time (min)</th><th>%CO_2</th><th>%B</th></tr>
<tr><td>Initial</td><td>95</td><td>5</td></tr>
<tr><td>0.25</td><td>95</td><td>5</td></tr>
<tr><td>2.50</td><td>70</td><td>30</td></tr>
<tr><td>3.00</td><td>70</td><td>30</td></tr>
<tr><td>3.01</td><td>50</td><td>50</td></tr>
<tr><td>3.70</td><td>50</td><td>50</td></tr>
<tr><td>3.71</td><td>95</td><td>5</td></tr>
<tr><td>4.00</td><td>95</td><td>5</td></tr>
</table></td></tr>
<tr><td>57-SFC[b]</td><td>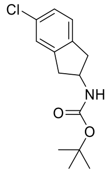</td><td>ChiralPak IJ-3</td><td>Isopropanol 0.1% Diethylamine</td><td><table>
<tr><th>Time (min)</th><th>%CO_2</th><th>%B</th></tr>
<tr><td>Initial</td><td>97.0</td><td>3.0</td></tr>
<tr><td>0.25</td><td>97.0</td><td>3.0</td></tr>
<tr><td>2.50</td><td>90</td><td>10</td></tr>
<tr><td>3.00</td><td>90</td><td>10</td></tr>
<tr><td>3.01</td><td>50</td><td>50</td></tr>
<tr><td>3.70</td><td>50</td><td>50</td></tr>
<tr><td>3.71</td><td>97.0</td><td>3.0</td></tr>
<tr><td>4.00</td><td>97.0</td><td>3.0</td></tr>
</table></td></tr>
<tr><td>64-SFC[b]</td><td>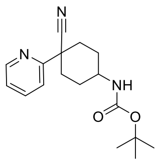</td><td>ChiralPak IG-3</td><td>Methanol 0.1% Diethylamine</td><td><table>
<tr><th>Time (min)</th><th>%CO_2</th><th>%B</th></tr>
<tr><td>Initial</td><td>97.0</td><td>3.0</td></tr>
<tr><td>0.25</td><td>97.0</td><td>3.0</td></tr>
<tr><td>3.00</td><td>40.0</td><td>60.0</td></tr>
<tr><td>3.70</td><td>40.0</td><td>60.0</td></tr>
<tr><td>3.71</td><td>97.0</td><td>3.0</td></tr>
<tr><td>4.00</td><td>97.0</td><td>3.0</td></tr>
</table></td></tr>
<tr><td>66-SFC[b]</td><td>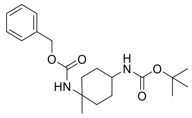</td><td>ChiralPak IK-3</td><td>Isopropanol 0.1% Diethylamine</td><td><table>
<tr><th>Time (min)</th><th>%CO_2</th><th>%B</th></tr>
<tr><td>Initial</td><td>95</td><td>5</td></tr>
<tr><td>0.25</td><td>95</td><td>5</td></tr>
<tr><td>4.00</td><td>50</td><td>50</td></tr>
<tr><td>4.70</td><td>50</td><td>50</td></tr>
<tr><td>4.71</td><td>95</td><td>5</td></tr>
<tr><td>5.00</td><td>95</td><td>5</td></tr>
</table></td></tr>
<tr><td>67-SFC[c]</td><td>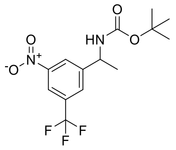</td><td>Whelk-O (S,S) 3.5 µm</td><td>Methanol 0.1% Diethylamine</td><td><table>
<tr><th>Time (min)</th><th>%CO_2</th><th>%B</th></tr>
<tr><td>Initial</td><td>97.0</td><td>3.0</td></tr>
<tr><td>0.25</td><td>97.0</td><td>3.0</td></tr>
<tr><td>3.00</td><td>40.0</td><td>60.0</td></tr>
<tr><td>3.70</td><td>40.0</td><td>60.0</td></tr>
<tr><td>3.71</td><td>97.0</td><td>3.0</td></tr>
</table></td></tr>
</table>

<table>
<tr><td></td><td></td><td></td><td></td><td><table><tr><td>4.00</td><td>97.0</td><td>3.0</td></tr></table></td></tr>
<tr><td>68-SFC[b]</td><td>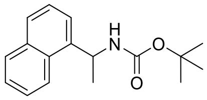</td><td>ChiralPak AD-3</td><td>Isopropanol 0.1% Diethylamine</td><td><table><tr><th>Time (min)</th><th>%CO_2</th><th>%B</th></tr><tr><td>Initial</td><td>97.0</td><td>3.0</td></tr><tr><td>0.25</td><td>97.0</td><td>3.0</td></tr><tr><td>3.00</td><td>40.0</td><td>60.0</td></tr><tr><td>3.70</td><td>40.0</td><td>60.0</td></tr><tr><td>3.71</td><td>97.0</td><td>3.0</td></tr><tr><td>4.00</td><td>97.0</td><td>3.0</td></tr></table></td></tr>
<tr><td>69-SFC[c]</td><td>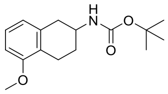</td><td>ChiralCel OD-3</td><td>Methanol 0.1% Diethylamine</td><td><table><tr><th>Time (min)</th><th>%CO_2</th><th>%B</th></tr><tr><td>Initial</td><td>97.0</td><td>3.0</td></tr><tr><td>0.25</td><td>97.0</td><td>3.0</td></tr><tr><td>3.00</td><td>40.0</td><td>60.0</td></tr><tr><td>3.70</td><td>40.0</td><td>60.0</td></tr><tr><td>3.71</td><td>97.0</td><td>3.0</td></tr><tr><td>4.00</td><td>97.0</td><td>3.0</td></tr></table></td></tr>
<tr><td>69-SFC[b]</td><td>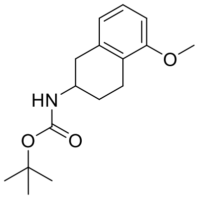</td><td>ChiralPak IB-3</td><td>Methanol 0.1% Diethylamine</td><td><table><tr><th>Time (min)</th><th>%CO_2</th><th>%B</th></tr><tr><td>Initial</td><td>97.0</td><td>3.0</td></tr><tr><td>0.25</td><td>97.0</td><td>3.0</td></tr><tr><td>3.00</td><td>40.0</td><td>60.0</td></tr><tr><td>3.70</td><td>40.0</td><td>60.0</td></tr><tr><td>3.71</td><td>97.0</td><td>3.0</td></tr><tr><td>4.00</td><td>97.0</td><td>3.0</td></tr></table></td></tr>
</table>

[a] ABPR pressure 152 bar and column temperature 35°C; [b] ABPR pressure 120 bar and column temperature 40°C; [c] ABPR pressure 120 bar and column temperature 35°C

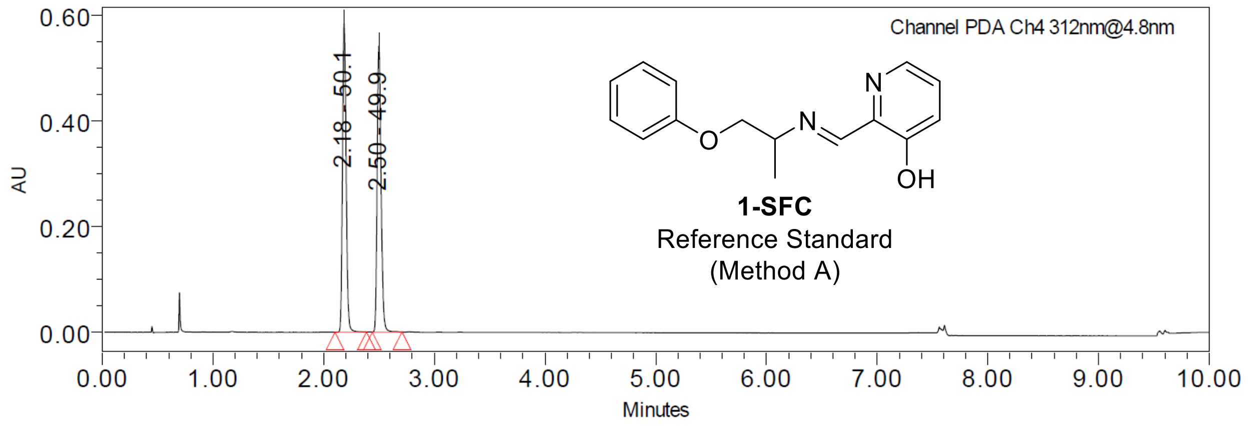

0.60
0.40
0.20
0.00
AU
2.18 - 50.1
2.50 - 49.9
Channel PDA Ch4 312nm@4.8nm
N
O
N
OH
1-SFC
Reference Standard
(Method A)
0.00
1.00
2.00
3.00
4.00
5.00
6.00
7.00
8.00
9.00
10.00
Minutes

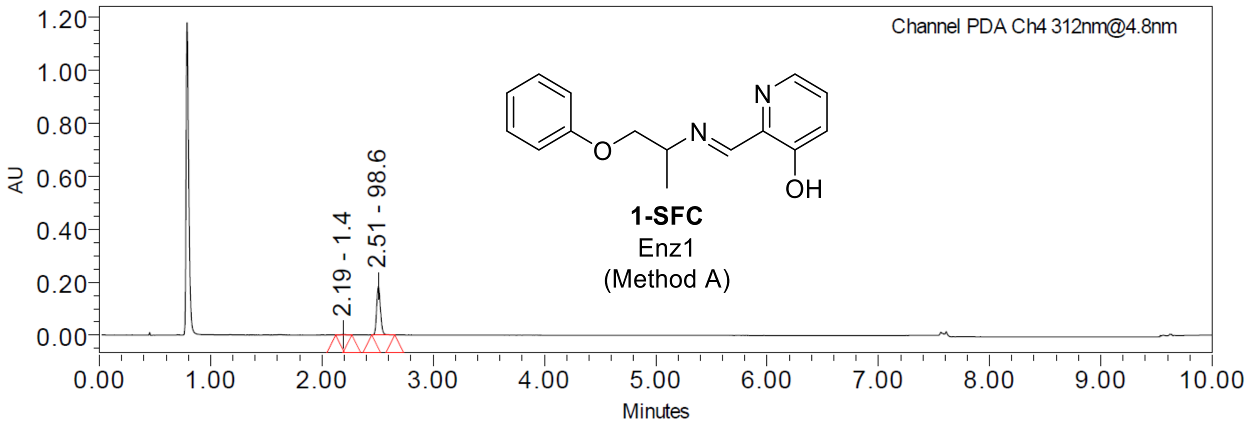

1.20
1.00
0.80
0.60
0.40
0.20
0.00
AU
2.19 - 1.4
2.51 - 98.6
Channel PDA Ch4 312nm@4.8nm
N
O
N
OH
1-SFC
Enz1
(Method A)
0.00
1.00
2.00
3.00
4.00
5.00
6.00
7.00
8.00
9.00
10.00
Minutes

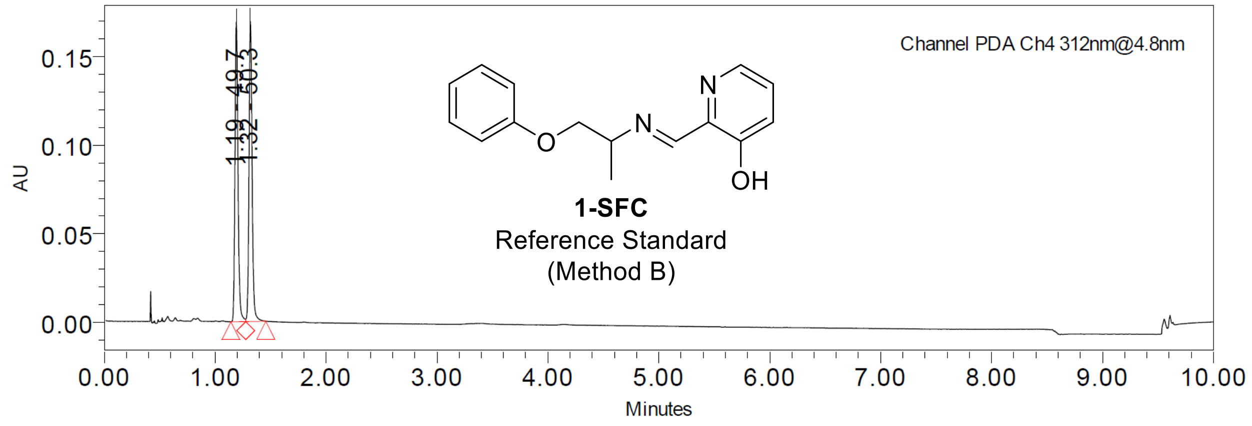

0.15
0.10
0.05
0.00
AU
1.19 - 49.7
1.32 - 50.3
Channel PDA Ch4 312nm@4.8nm
N
O
N
OH
1-SFC
Reference Standard
(Method B)
0.00
1.00
2.00
3.00
4.00
5.00
6.00
7.00
8.00
9.00
10.00
Minutes

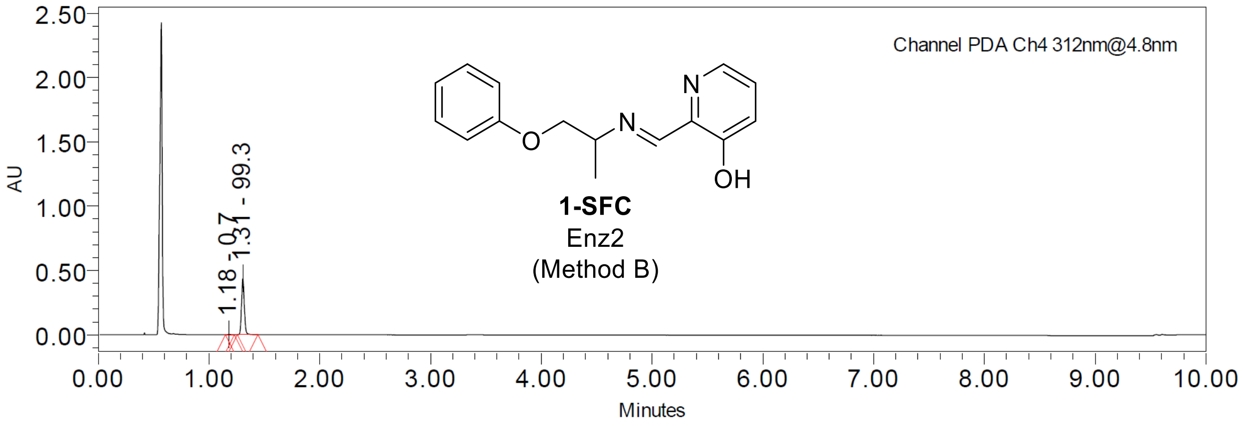

Channel PDA Ch4 312nm@4.8nm
1-SFC
Enz2
(Method B)
1.18 - 0.7
1.31 - 99.3
AU
Minutes

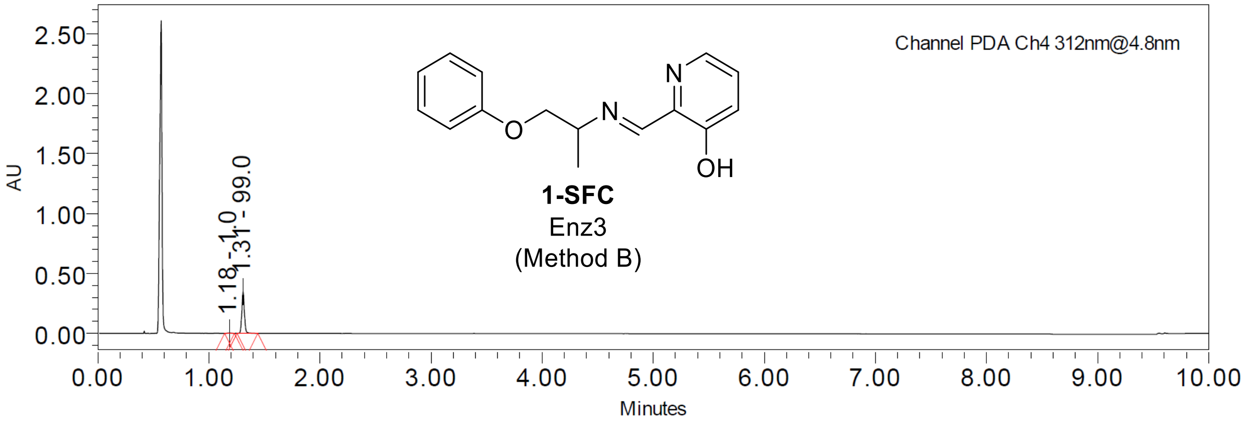

Channel PDA Ch4 312nm@4.8nm
1-SFC
Enz3
(Method B)
1.18 - 1.0
1.31 - 99.0
AU
Minutes

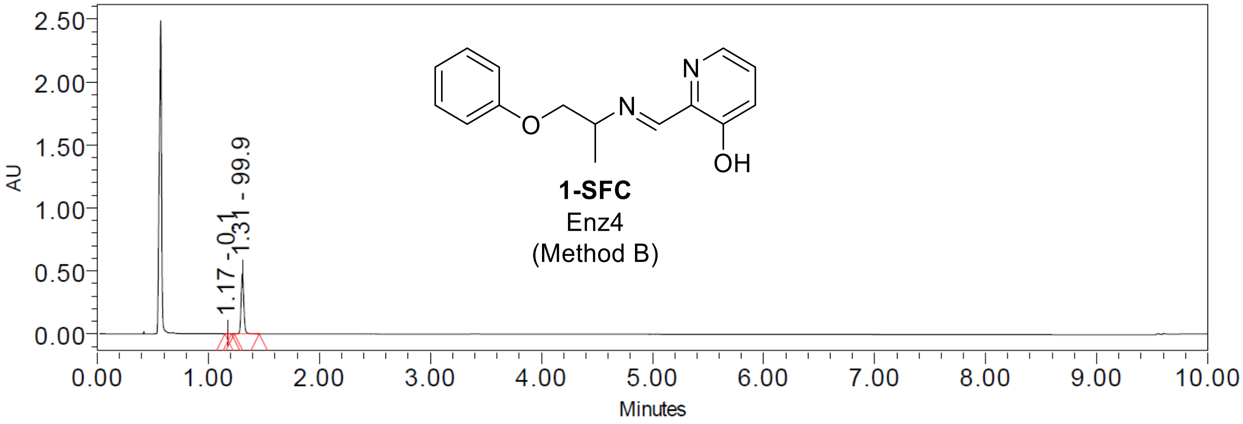

1-SFC
Enz4
(Method B)
1.17 - 0.1
1.31 - 99.9
AU
Minutes

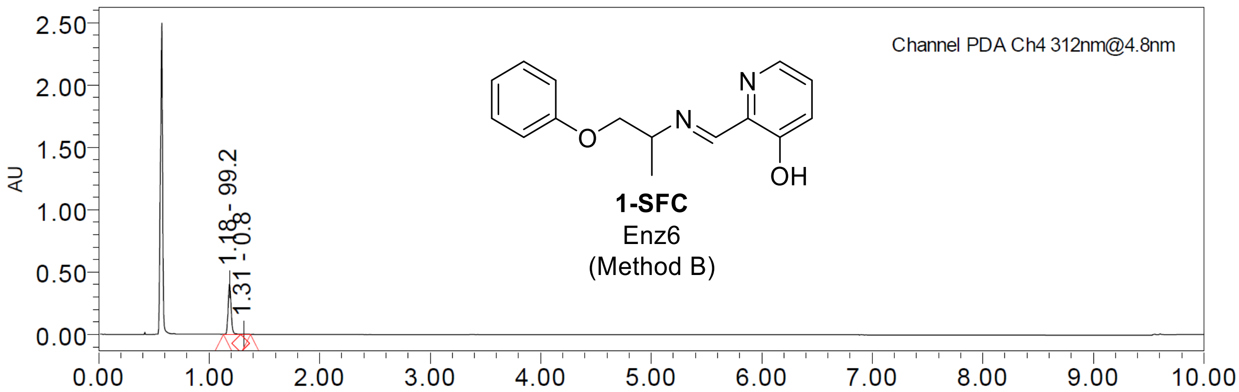
Channel PDA Ch4 312nm@4.8nm
AU
1.18 - 99.2
1.31 - 0.8
N
O
N
OH
1-SFC
Enz6
(Method B)

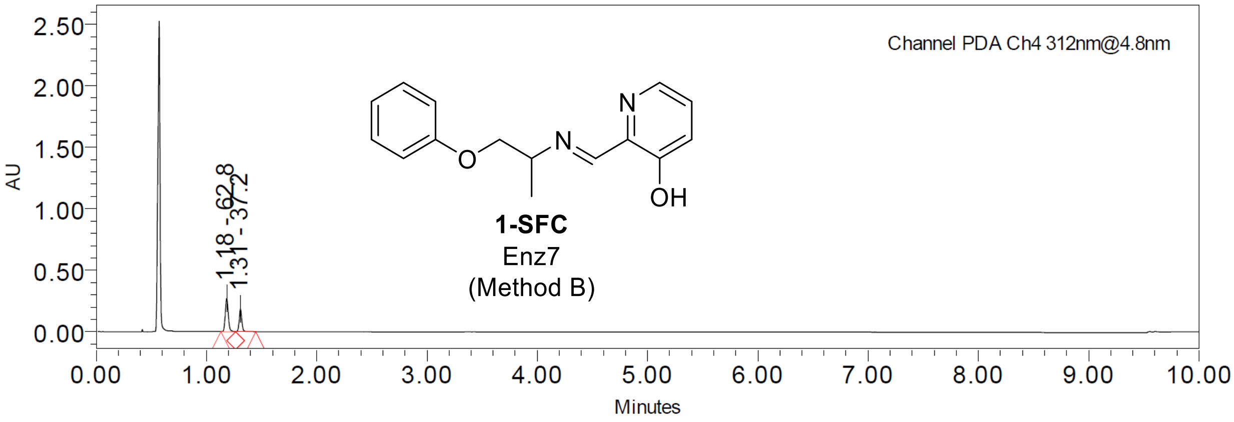
Channel PDA Ch4 312nm@4.8nm
AU
1.18 - 62.8
1.31 - 37.2
N
O
N
OH
1-SFC
Enz7
(Method B)
Minutes

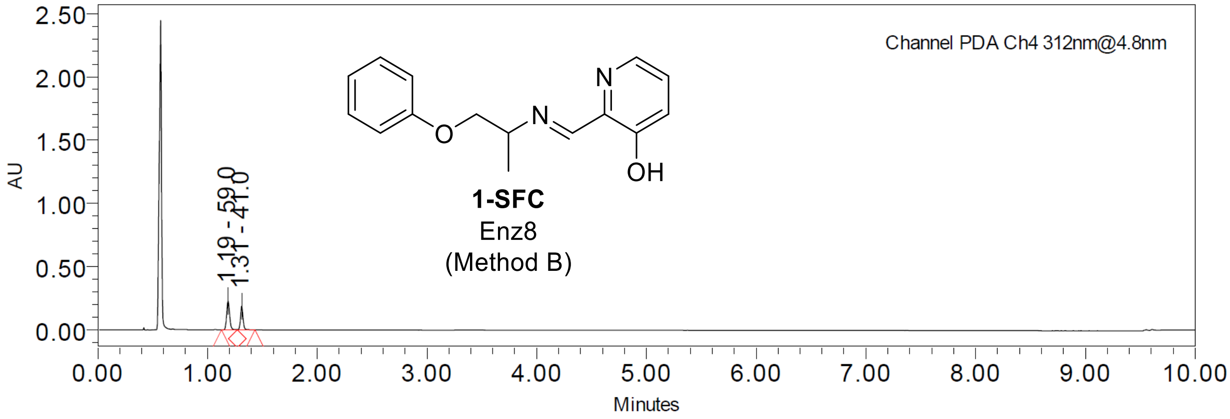
Channel PDA Ch4 312nm@4.8nm
AU
1.19 - 59.0
1.31 - 41.0
N
O
N
OH
1-SFC
Enz8
(Method B)
Minutes

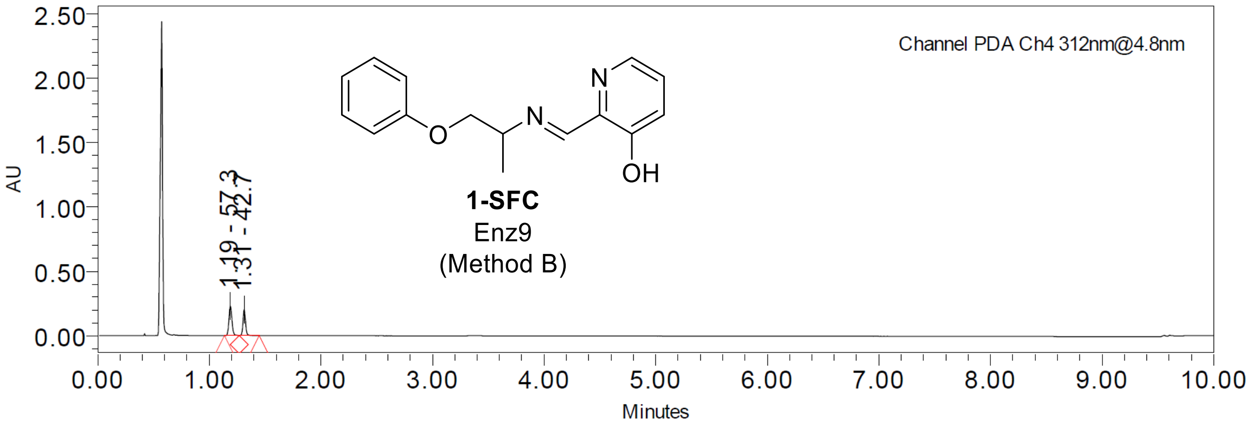
Channel PDA Ch4 312nm@4.8nm
1-SFC
Enz9
(Method B)
1.19 - 57.3
1.31 - 42.7
AU
Minutes

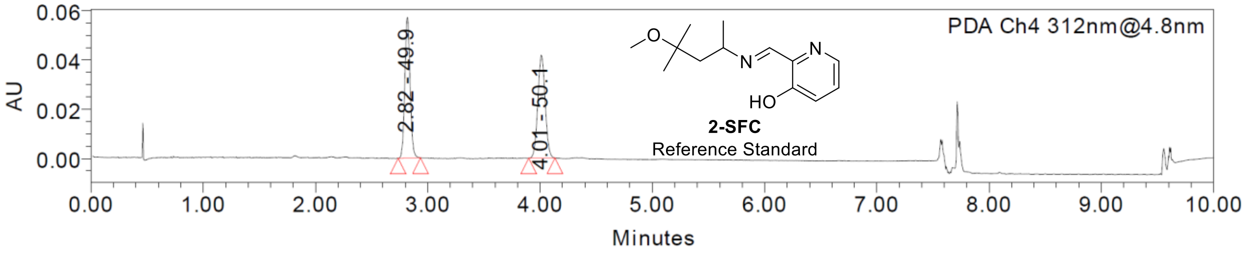
PDA Ch4 312nm@4.8nm
2-SFC
Reference Standard
2.82 - 49.9
4.01 - 50.1
AU
Minutes

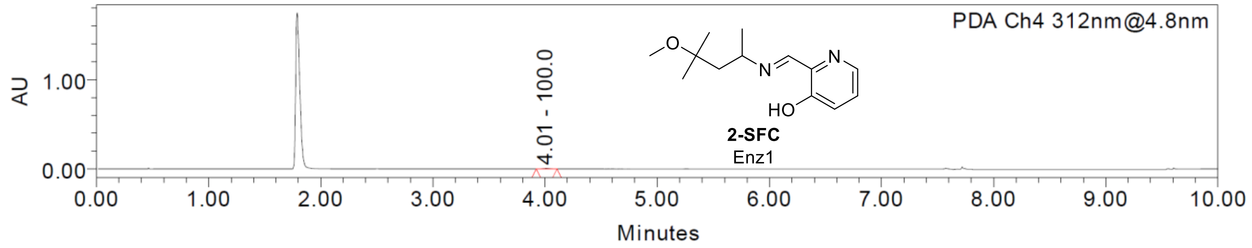
PDA Ch4 312nm@4.8nm
2-SFC
Enz1
4.01 - 100.0
AU
Minutes

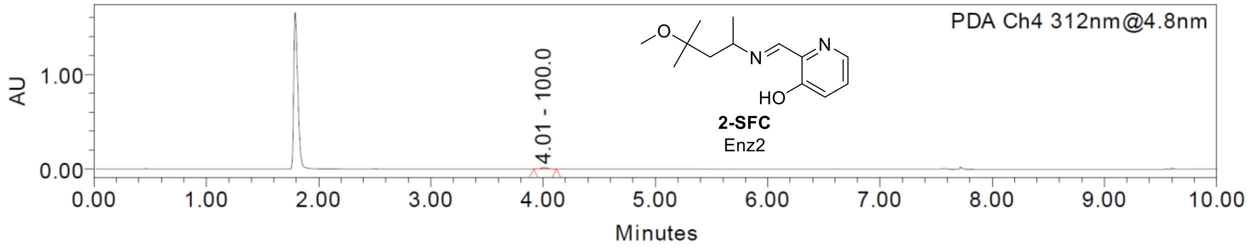
PDA Ch4 312nm@4.8nm
2-SFC
Enz2
4.01 - 100.0
AU
Minutes

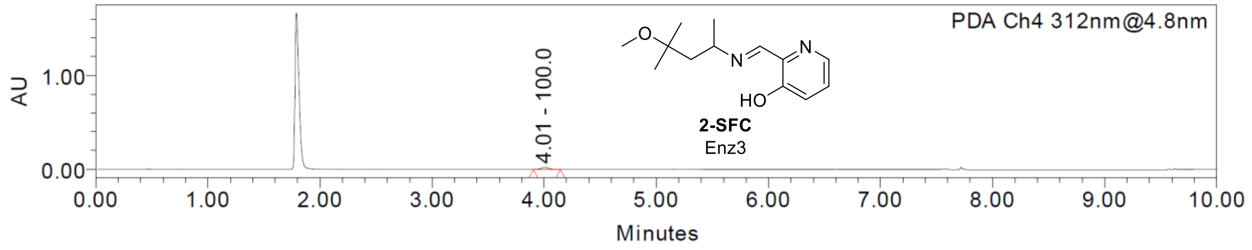
PDA Ch4 312nm@4.8nm
2-SFC
Enz3
4.01 - 100.0
AU
Minutes

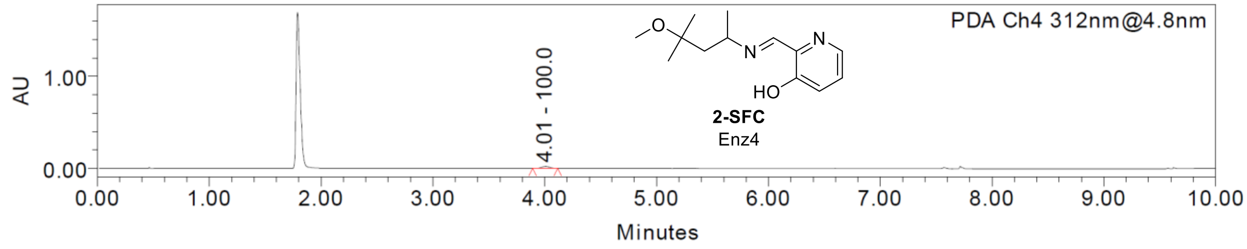
PDA Ch4 312nm@4.8nm
AU
1.00
0.00
4.01 - 100.0
HO
N
O
2-SFC
Enz4
0.00 1.00 2.00 3.00 4.00 5.00 6.00 7.00 8.00 9.00 10.00
Minutes

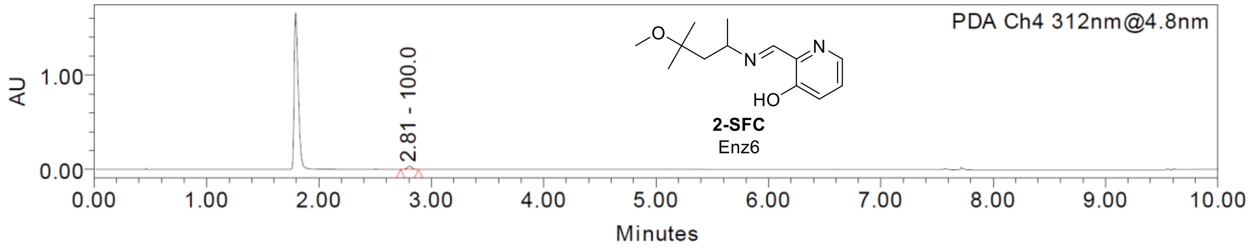
PDA Ch4 312nm@4.8nm
AU
1.00
0.00
2.81 - 100.0
HO
N
O
2-SFC
Enz6
0.00 1.00 2.00 3.00 4.00 5.00 6.00 7.00 8.00 9.00 10.00
Minutes

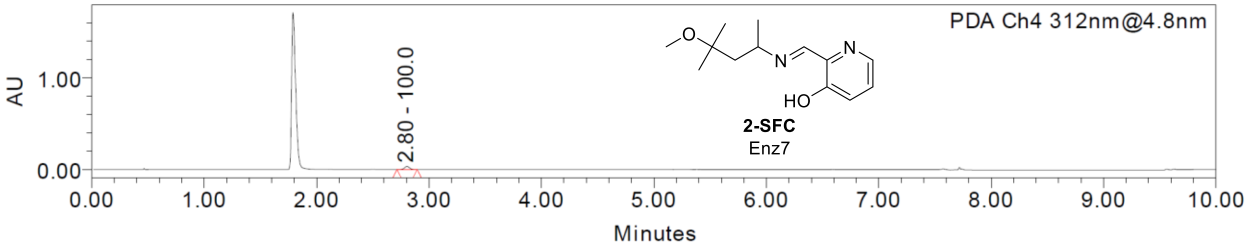
PDA Ch4 312nm@4.8nm
AU
1.00
0.00
2.80 - 100.0
HO
N
O
2-SFC
Enz7
0.00 1.00 2.00 3.00 4.00 5.00 6.00 7.00 8.00 9.00 10.00
Minutes

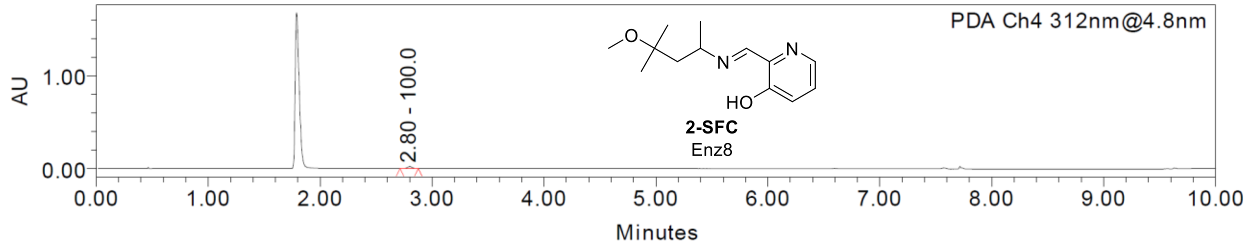
PDA Ch4 312nm@4.8nm
AU
1.00
0.00
2.80 - 100.0
HO
N
O
2-SFC
Enz8
0.00 1.00 2.00 3.00 4.00 5.00 6.00 7.00 8.00 9.00 10.00
Minutes

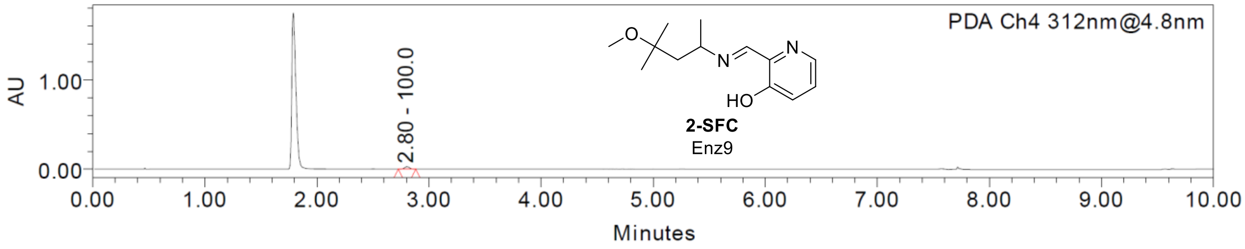
PDA Ch4 312nm@4.8nm
AU
1.00
0.00
2.80 - 100.0
HO
N
O
2-SFC
Enz9
0.00 1.00 2.00 3.00 4.00 5.00 6.00 7.00 8.00 9.00 10.00
Minutes

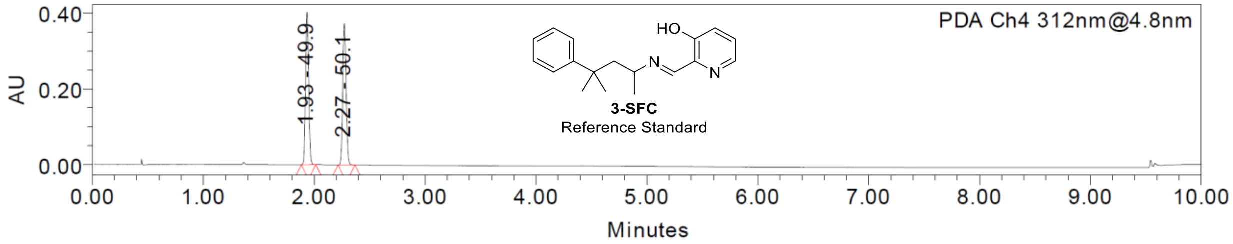
PDA Ch4 312nm@4.8nm
AU
0.40
0.20
0.00
1.93 - 49.9
2.27 - 50.1
HO
N
3-SFC
Reference Standard
0.00 1.00 2.00 3.00 4.00 5.00 6.00 7.00 8.00 9.00 10.00
Minutes

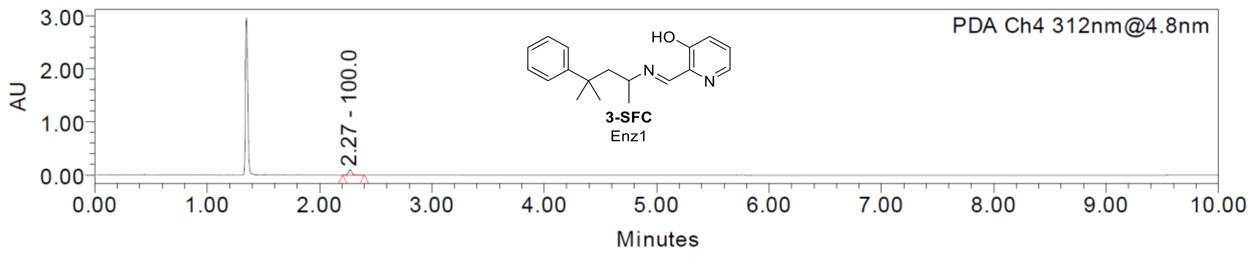
PDA Ch4 312nm@4.8nm
2.27 - 100.0
3-SFC
Enz1
AU
Minutes

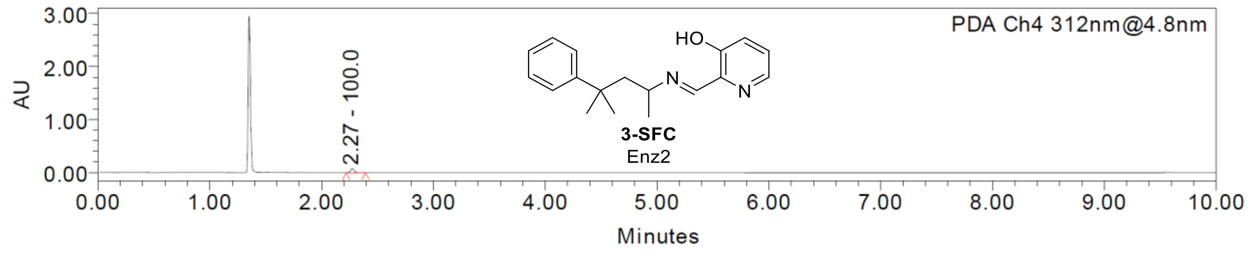
PDA Ch4 312nm@4.8nm
2.27 - 100.0
3-SFC
Enz2
AU
Minutes

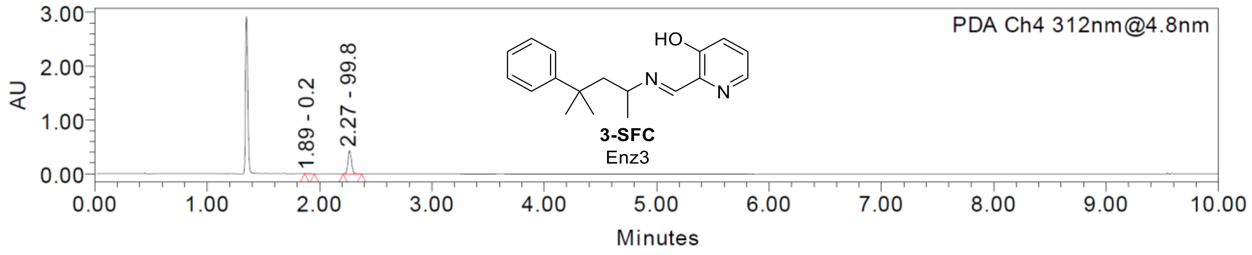
PDA Ch4 312nm@4.8nm
1.89 - 0.2
2.27 - 99.8
3-SFC
Enz3
AU
Minutes

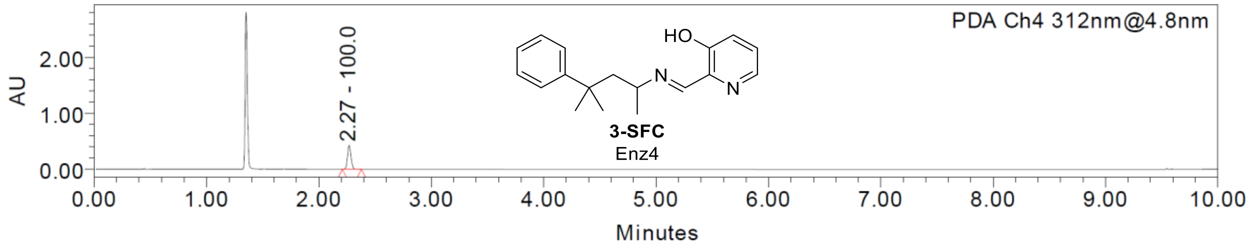
PDA Ch4 312nm@4.8nm
2.27 - 100.0
3-SFC
Enz4
AU
Minutes

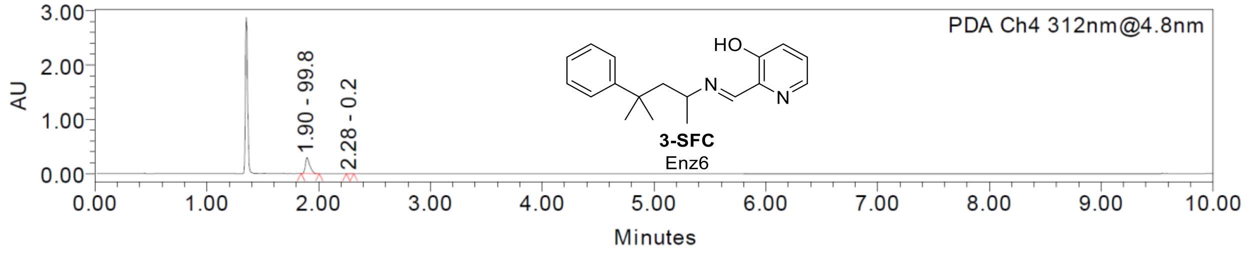
PDA Ch4 312nm@4.8nm
1.90 - 99.8
2.28 - 0.2
3-SFC
Enz6
AU
Minutes

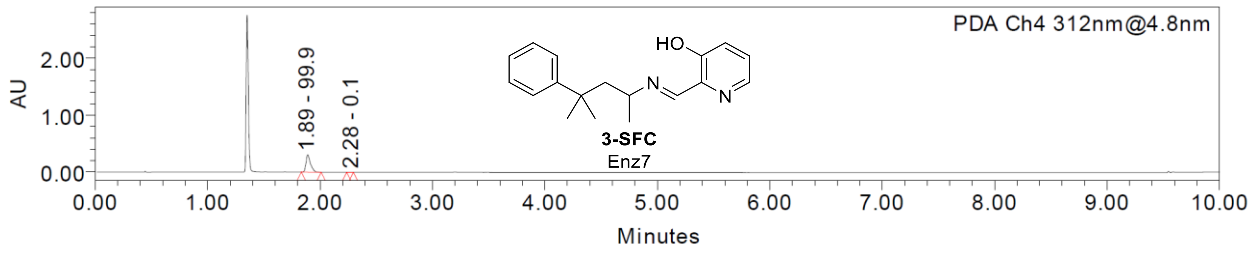
PDA Ch4 312nm@4.8nm
1.89 - 99.9
2.28 - 0.1
3-SFC
Enz7
AU
Minutes

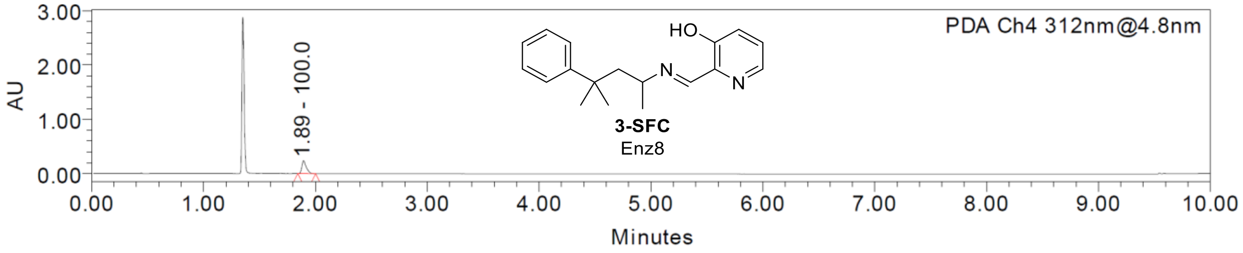
PDA Ch4 312nm@4.8nm
1.89 - 100.0
HO
N
N
3-SFC
Enz8
AU
Minutes

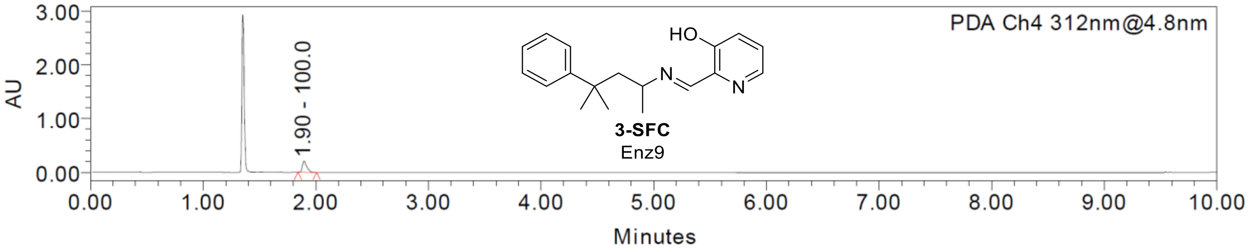
PDA Ch4 312nm@4.8nm
1.90 - 100.0
HO
N
N
3-SFC
Enz9
AU
Minutes

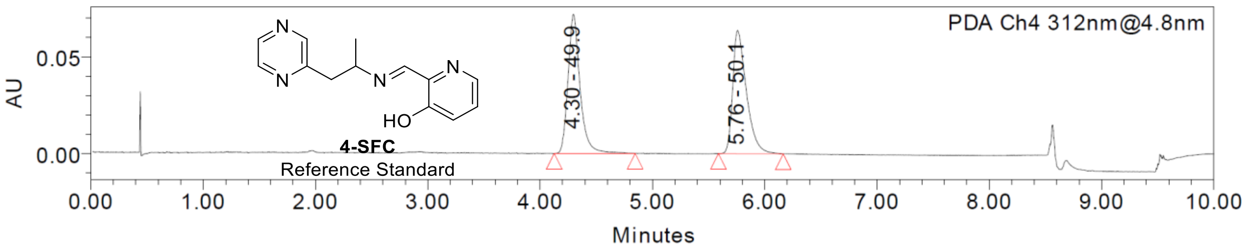
PDA Ch4 312nm@4.8nm
4.30 - 49.9
5.76 - 50.1
N
N
N
N
HO
4-SFC
Reference Standard
AU
Minutes

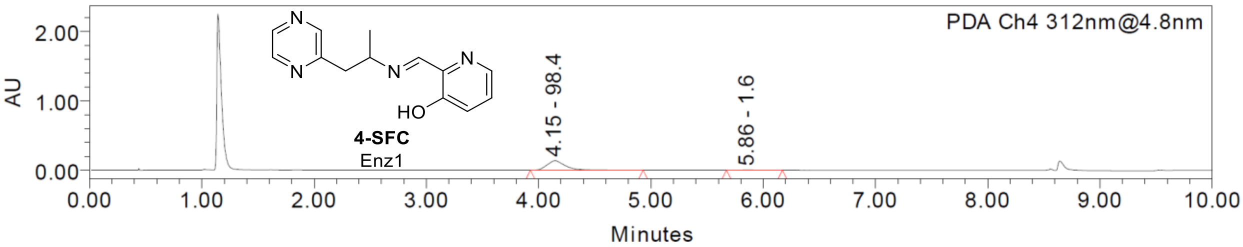
PDA Ch4 312nm@4.8nm
4.15 - 98.4
5.86 - 1.6
N
N
N
N
HO
4-SFC
Enz1
AU
Minutes

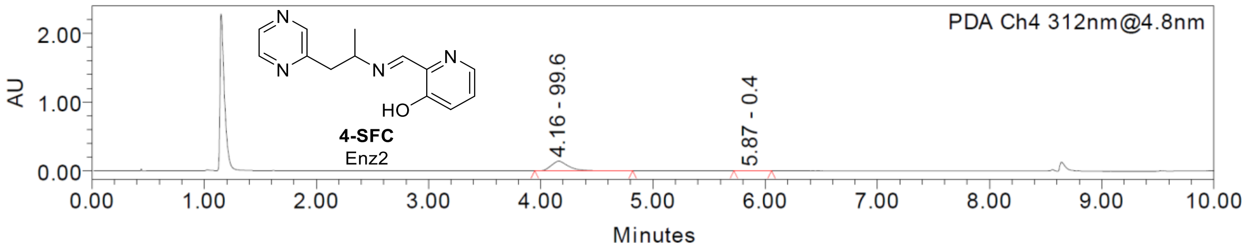
PDA Ch4 312nm@4.8nm
4.16 - 99.6
5.87 - 0.4
N
N
N
N
HO
4-SFC
Enz2
AU
Minutes

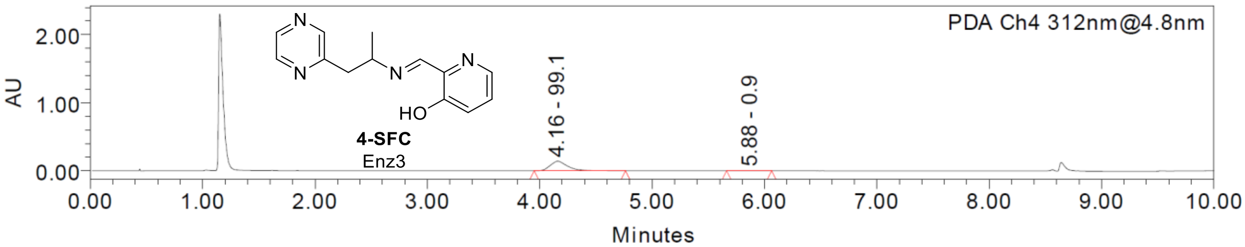
PDA Ch4 312nm@4.8nm
4.16 - 99.1
5.88 - 0.9
N
N
N
N
HO
4-SFC
Enz3
AU
Minutes

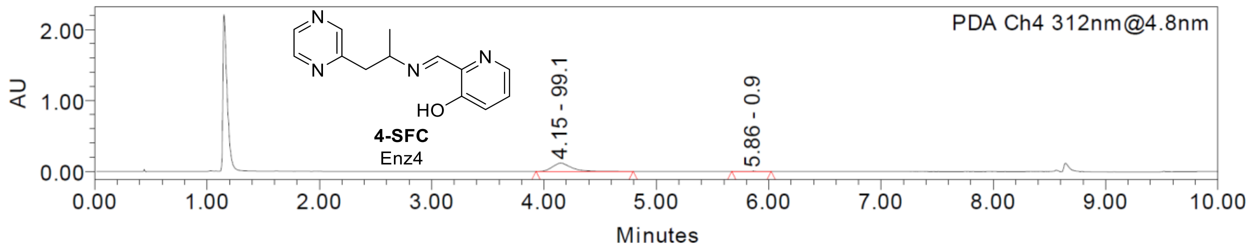
PDA Ch4 312nm@4.8nm
4-SFC
Enz4
4.15 - 99.1
5.86 - 0.9
AU
Minutes

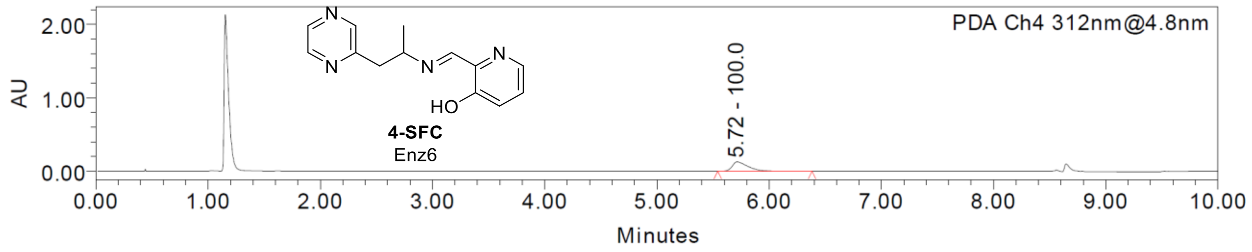
PDA Ch4 312nm@4.8nm
4-SFC
Enz6
5.72 - 100.0
AU
Minutes

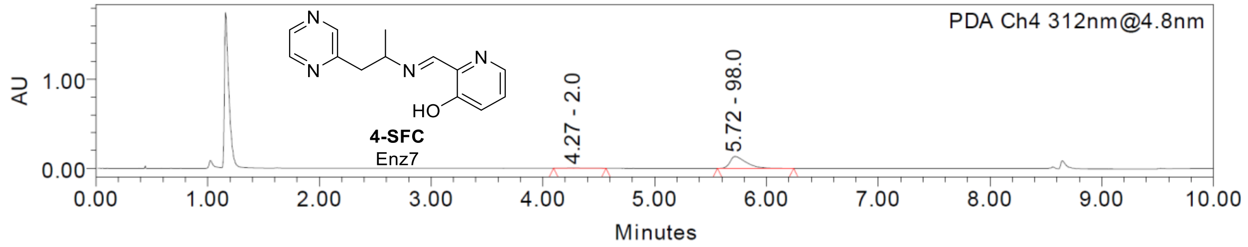
PDA Ch4 312nm@4.8nm
4-SFC
Enz7
4.27 - 2.0
5.72 - 98.0
AU
Minutes

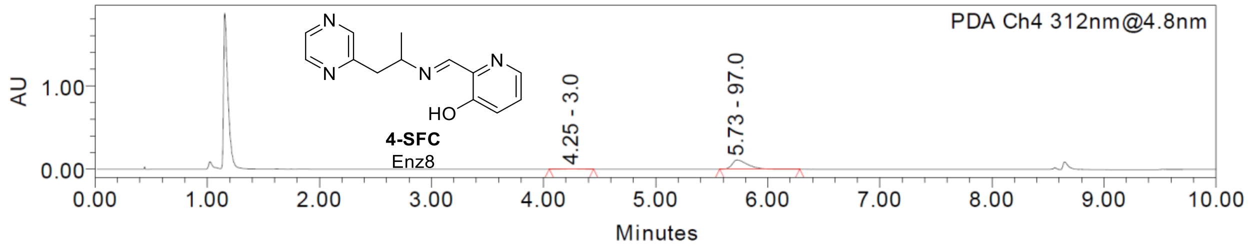
PDA Ch4 312nm@4.8nm
4-SFC
Enz8
4.25 - 3.0
5.73 - 97.0
AU
Minutes

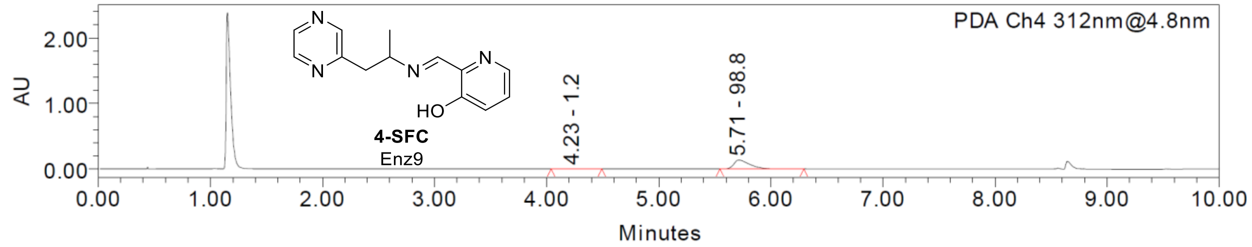
PDA Ch4 312nm@4.8nm
4-SFC
Enz9
4.23 - 1.2
5.71 - 98.8
AU
Minutes

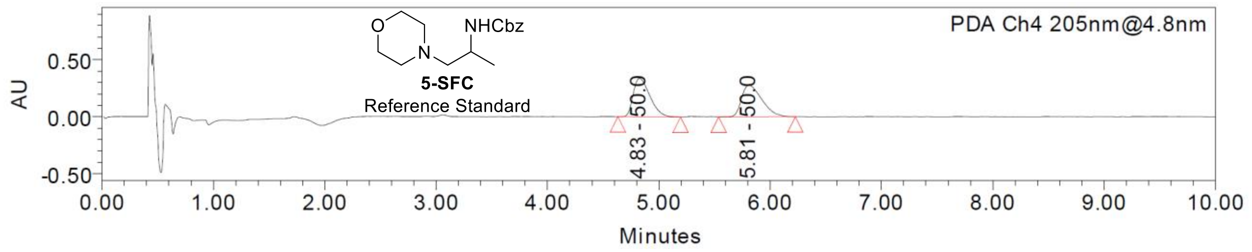
PDA Ch4 205nm@4.8nm
NHCbz
5-SFC
Reference Standard
4.83 - 50.0
5.81 - 50.0
AU
Minutes

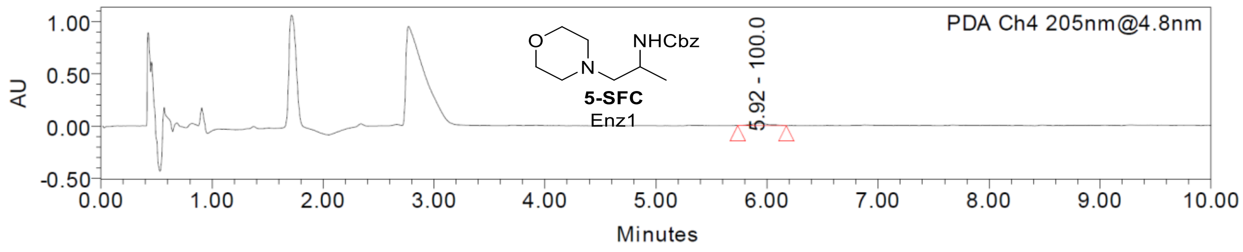
PDA Ch4 205nm@4.8nm
NHCbz
O
N
5-SFC
Enz1
5.92 - 100.0
AU
Minutes

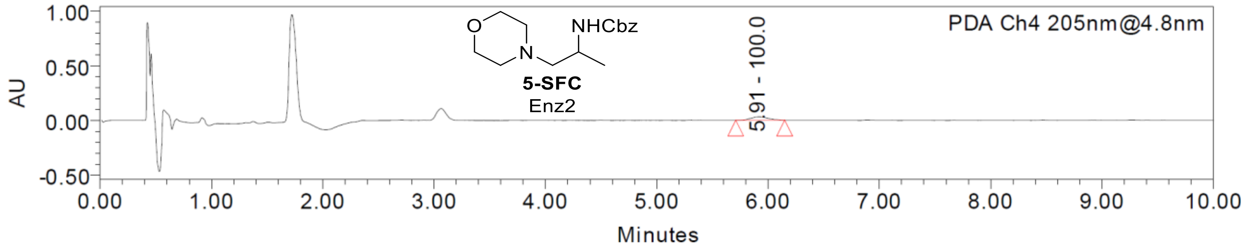
PDA Ch4 205nm@4.8nm
NHCbz
O
N
5-SFC
Enz2
5.91 - 100.0
AU
Minutes

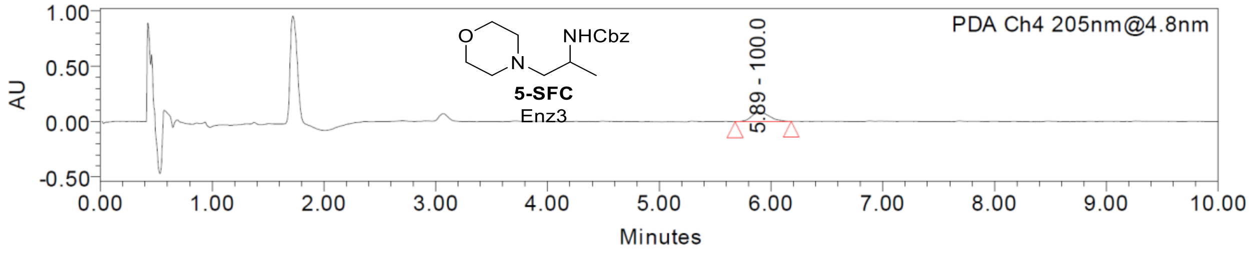
PDA Ch4 205nm@4.8nm
NHCbz
O
N
5-SFC
Enz3
5.89 - 100.0
AU
Minutes

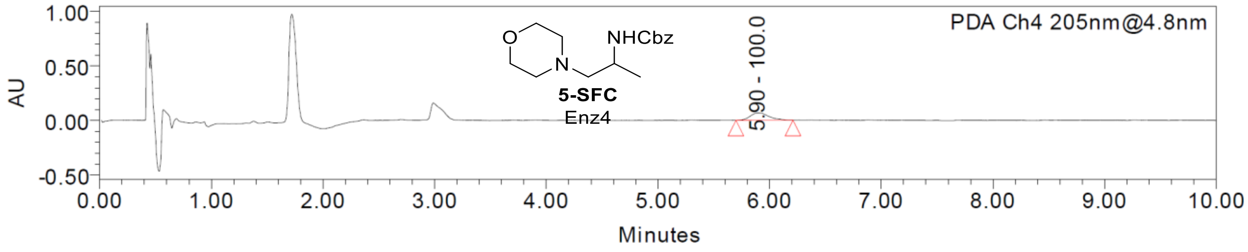
PDA Ch4 205nm@4.8nm
NHCbz
O
N
5-SFC
Enz4
5.90 - 100.0
AU
Minutes

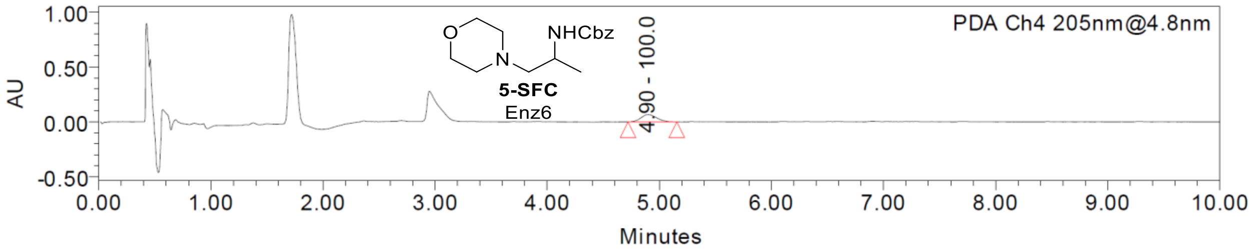
PDA Ch4 205nm@4.8nm
NHCbz
O
N
5-SFC
Enz6
4.90 - 100.0
AU
Minutes

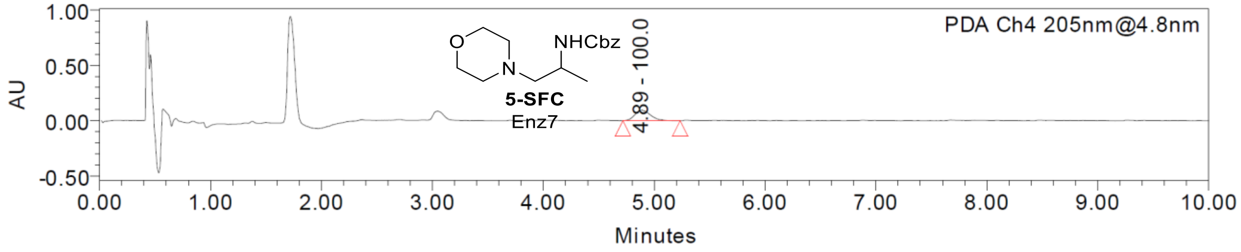
PDA Ch4 205nm@4.8nm
NHCbz
O
N
5-SFC
Enz7
4.89 - 100.0
AU
Minutes

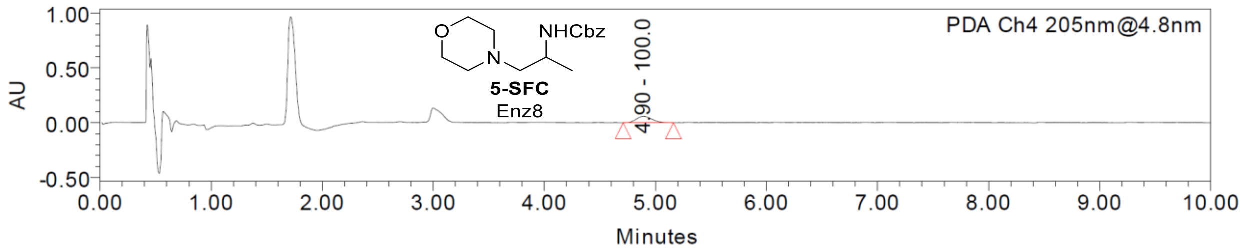
PDA Ch4 205nm@4.8nm
O
N
NHCbz
5-SFC
Enz8
4.90 - 100.0
AU
Minutes

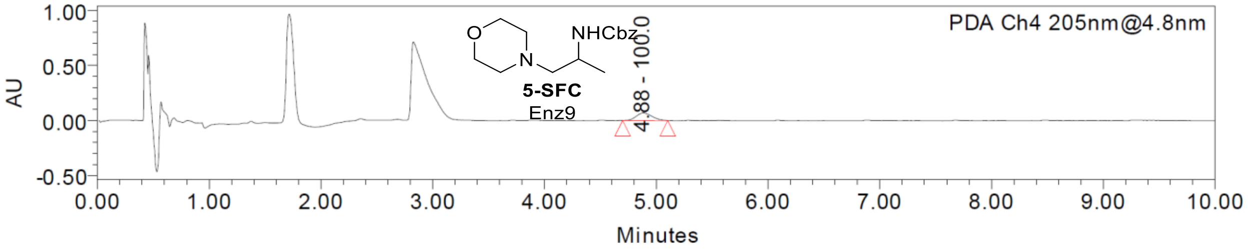
PDA Ch4 205nm@4.8nm
O
N
NHCbz
5-SFC
Enz9
4.88 - 100.0
AU
Minutes

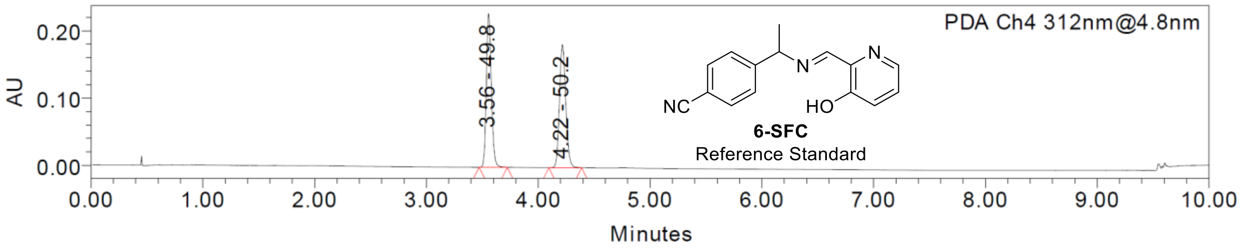
PDA Ch4 312nm@4.8nm
3.56 - 49.8
4.22 - 50.2
NC
N
N
HO
6-SFC
Reference Standard
AU
Minutes

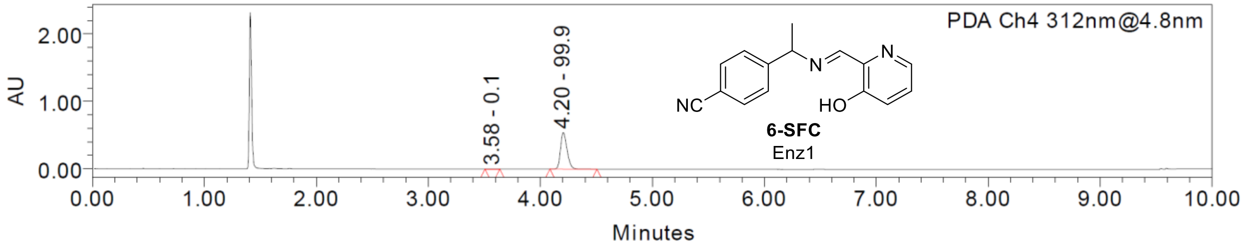
PDA Ch4 312nm@4.8nm
3.58 - 0.1
4.20 - 99.9
NC
N
N
HO
6-SFC
Enz1
AU
Minutes

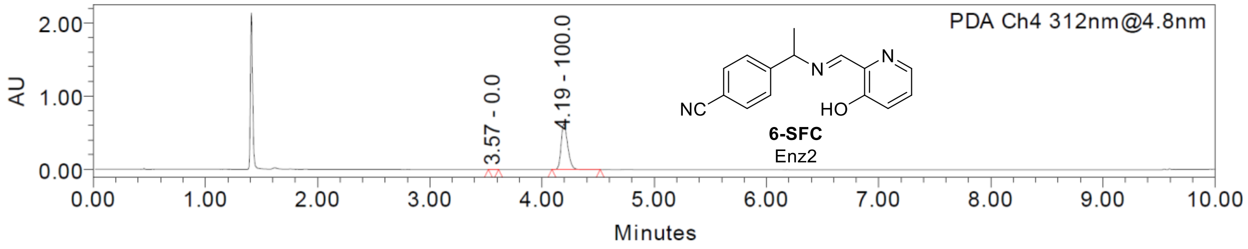
PDA Ch4 312nm@4.8nm
3.57 - 0.0
4.19 - 100.0
NC
N
N
HO
6-SFC
Enz2
AU
Minutes

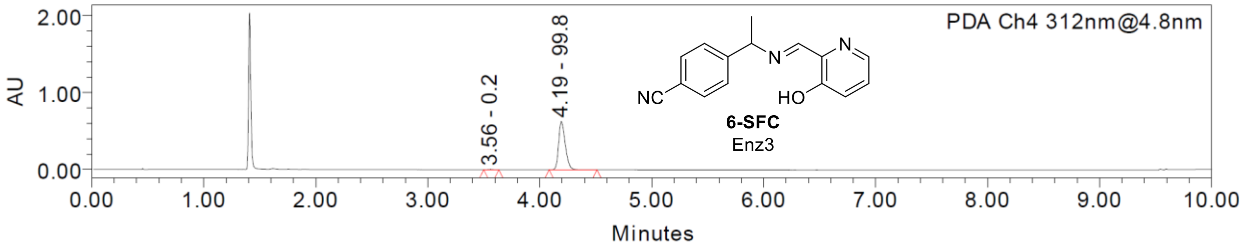
PDA Ch4 312nm@4.8nm
3.56 - 0.2
4.19 - 99.8
NC
N
N
HO
6-SFC
Enz3
AU
Minutes

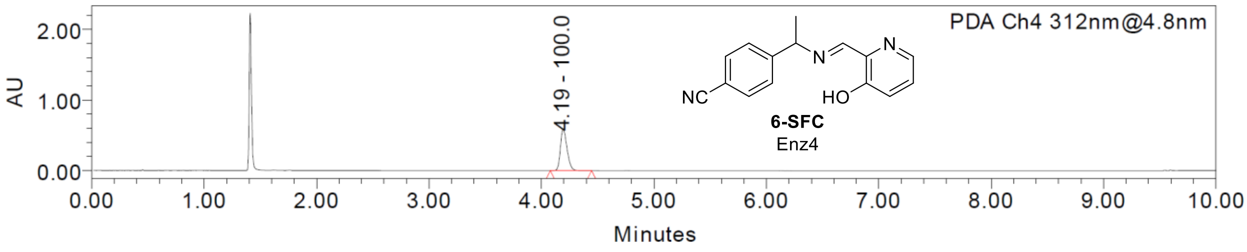
PDA Ch4 312nm@4.8nm
4.19 - 100.0
NC
N
HO
6-SFC
Enz4
AU
Minutes

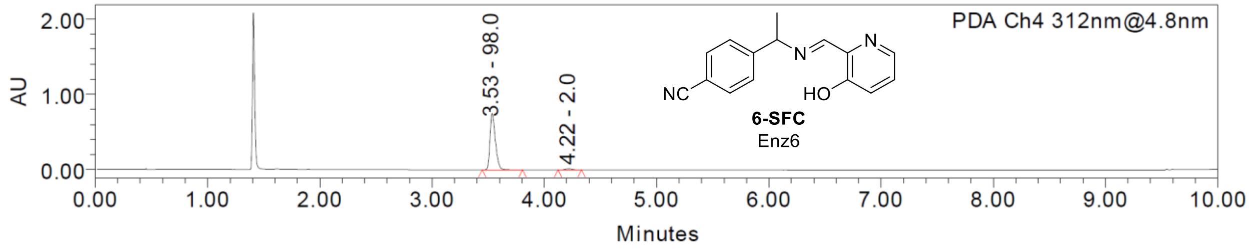
PDA Ch4 312nm@4.8nm
3.53 - 98.0
4.22 - 2.0
NC
N
HO
6-SFC
Enz6
AU
Minutes

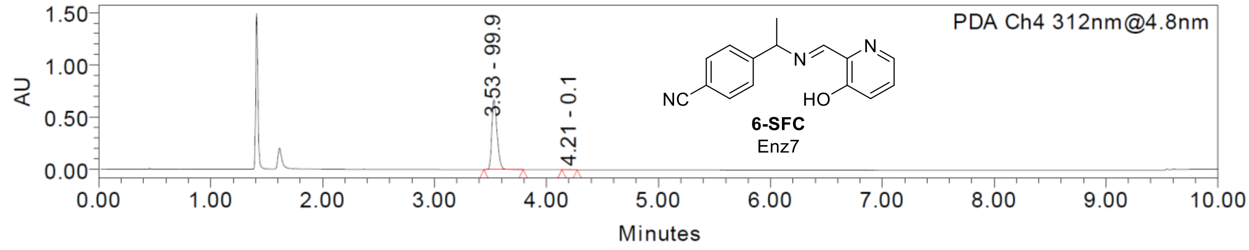
PDA Ch4 312nm@4.8nm
3.53 - 99.9
4.21 - 0.1
NC
N
HO
6-SFC
Enz7
AU
Minutes

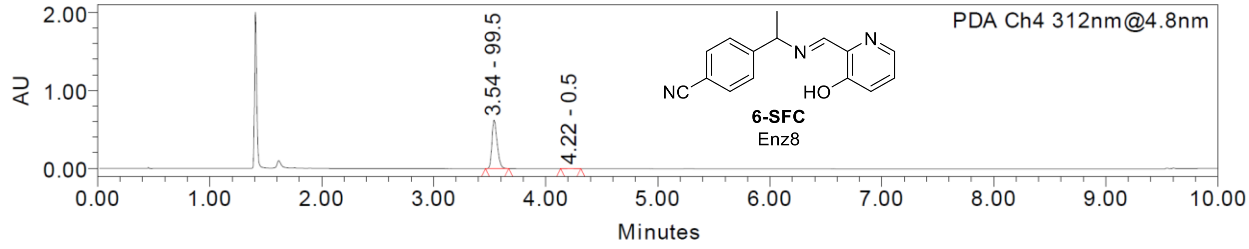
PDA Ch4 312nm@4.8nm
3.54 - 99.5
4.22 - 0.5
NC
N
HO
6-SFC
Enz8
AU
Minutes

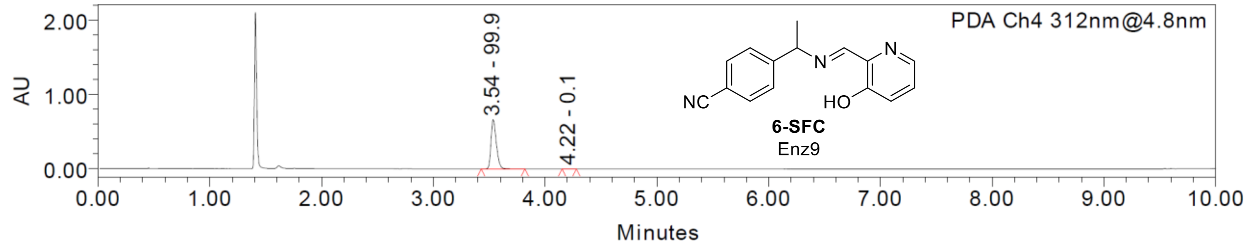
PDA Ch4 312nm@4.8nm
3.54 - 99.9
4.22 - 0.1
NC
N
HO
6-SFC
Enz9
AU
Minutes

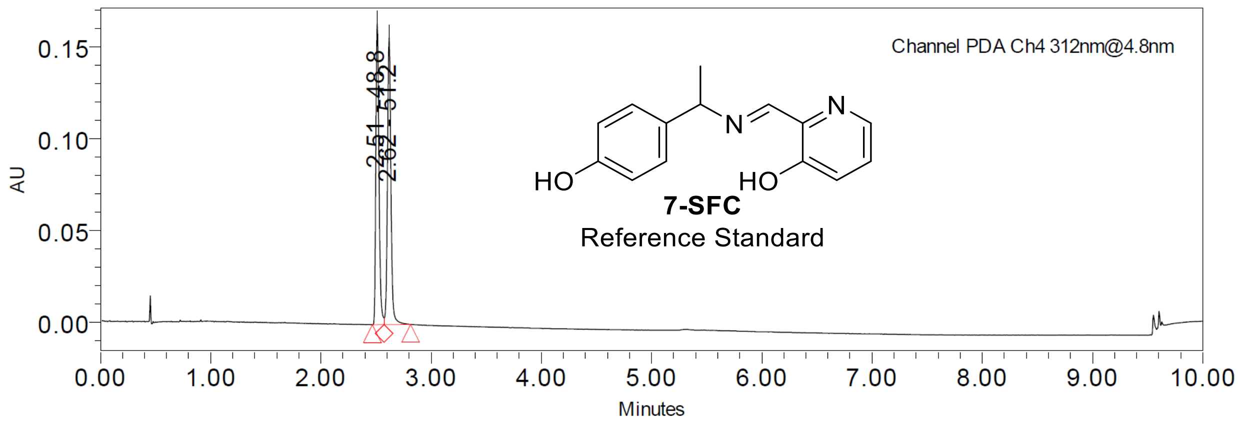
Channel PDA Ch4 312nm@4.8nm
AU
0.15
0.10
0.05
0.00
0.00
1.00
2.00
3.00
4.00
5.00
6.00
7.00
8.00
9.00
10.00
Minutes
2.51 - 48.8
2.62 - 51.2
HO
HO
N
N
7-SFC
Reference Standard

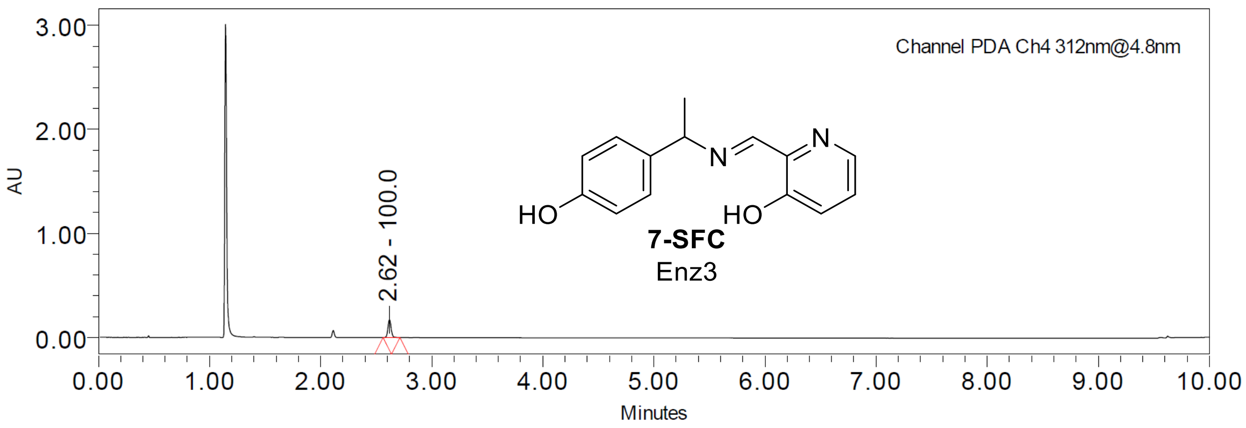
Channel PDA Ch4 312nm@4.8nm
AU
3.00
2.00
1.00
0.00
0.00
1.00
2.00
3.00
4.00
5.00
6.00
7.00
8.00
9.00
10.00
Minutes
2.62 - 100.0
HO
HO
N
N
7-SFC
Enz3

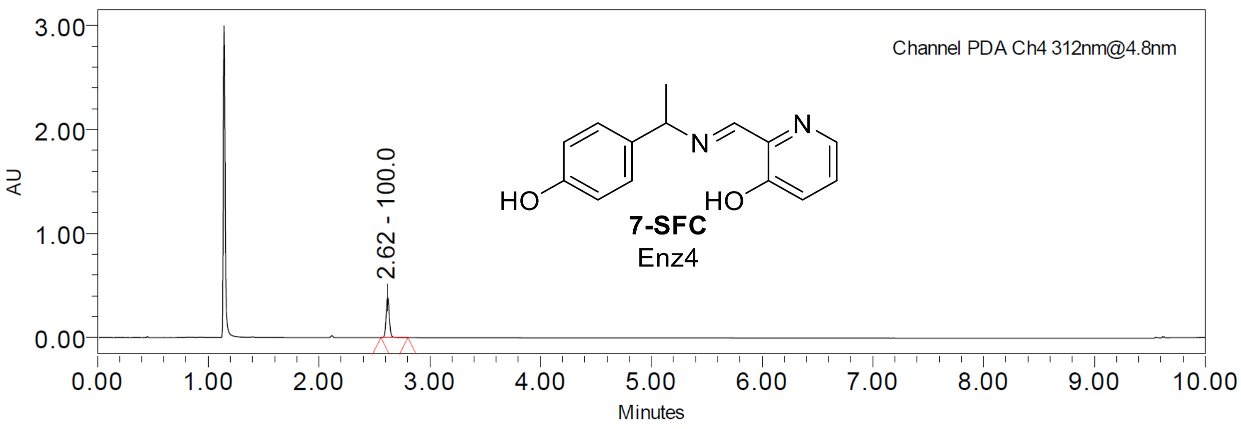
Channel PDA Ch4 312nm@4.8nm
AU
3.00
2.00
1.00
0.00
0.00
1.00
2.00
3.00
4.00
5.00
6.00
7.00
8.00
9.00
10.00
Minutes
2.62 - 100.0
HO
HO
N
N
7-SFC
Enz4

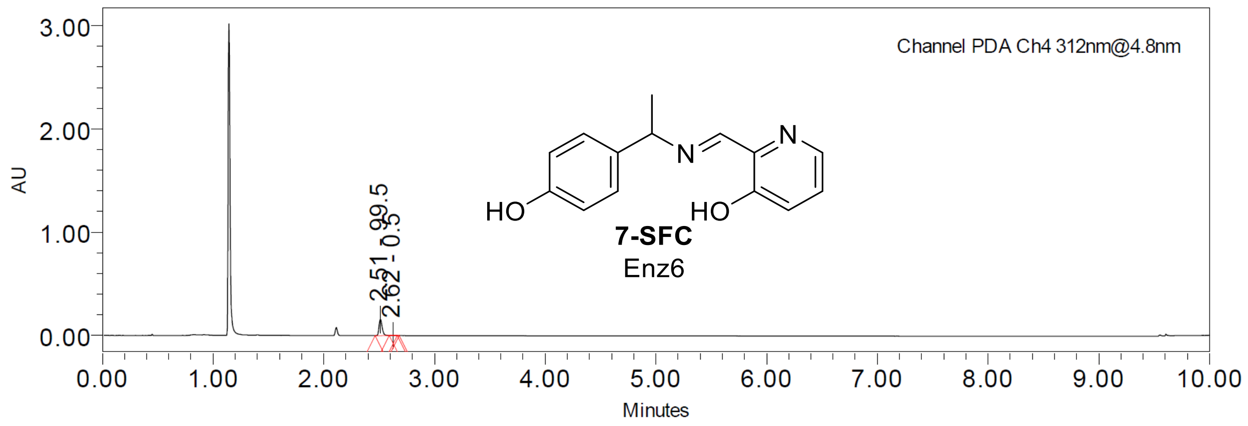
Channel PDA Ch4 312nm@4.8nm
7-SFC
Enz6
2.51 - 99.5
2.62 - 0.5
AU
Minutes

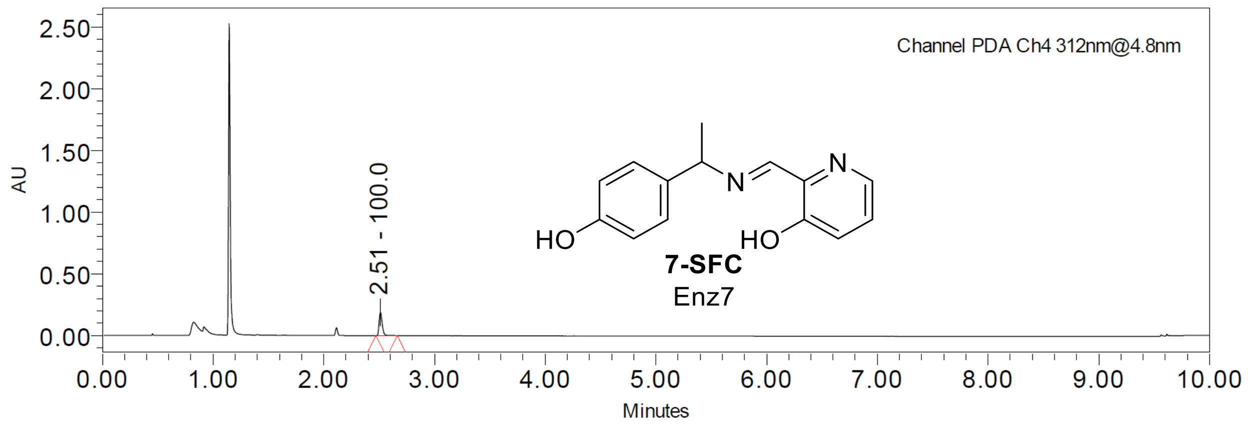
Channel PDA Ch4 312nm@4.8nm
7-SFC
Enz7
2.51 - 100.0
AU
Minutes

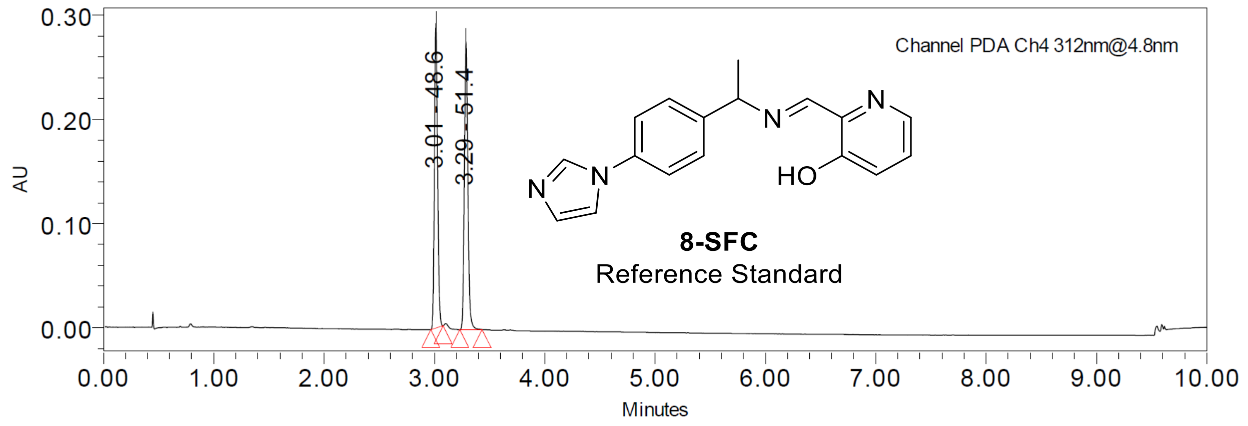
Channel PDA Ch4 312nm@4.8nm
8-SFC
Reference Standard
3.01 - 48.6
3.29 - 51.4
AU
Minutes

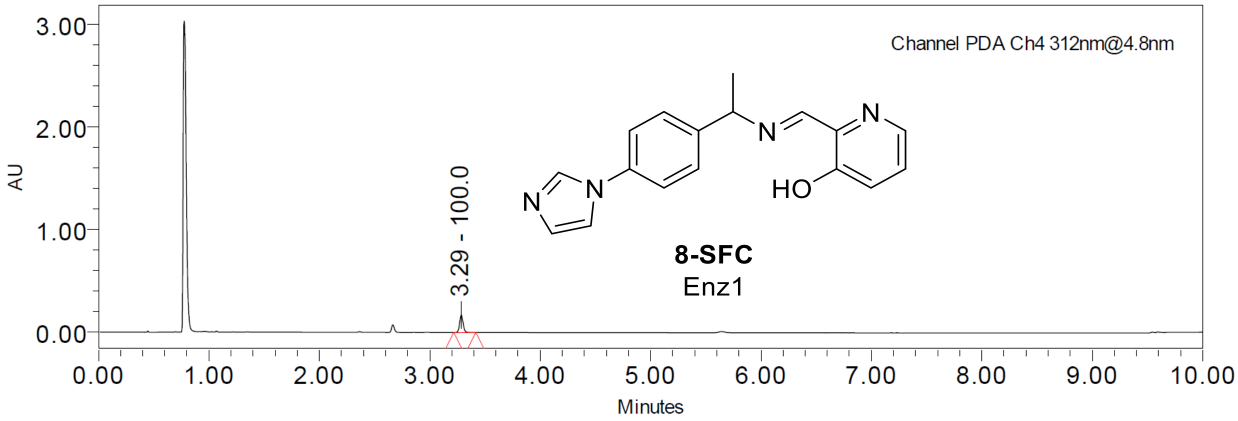
Channel PDA Ch4 312nm@4.8nm
3.29 - 100.0
8-SFC
Enz1
AU
Minutes

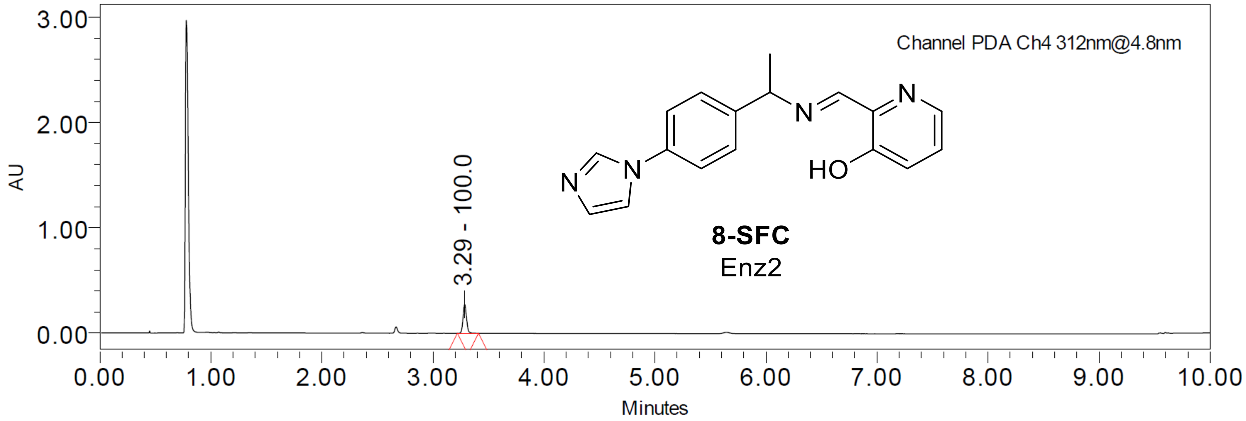
Channel PDA Ch4 312nm@4.8nm
3.29 - 100.0
8-SFC
Enz2
AU
Minutes

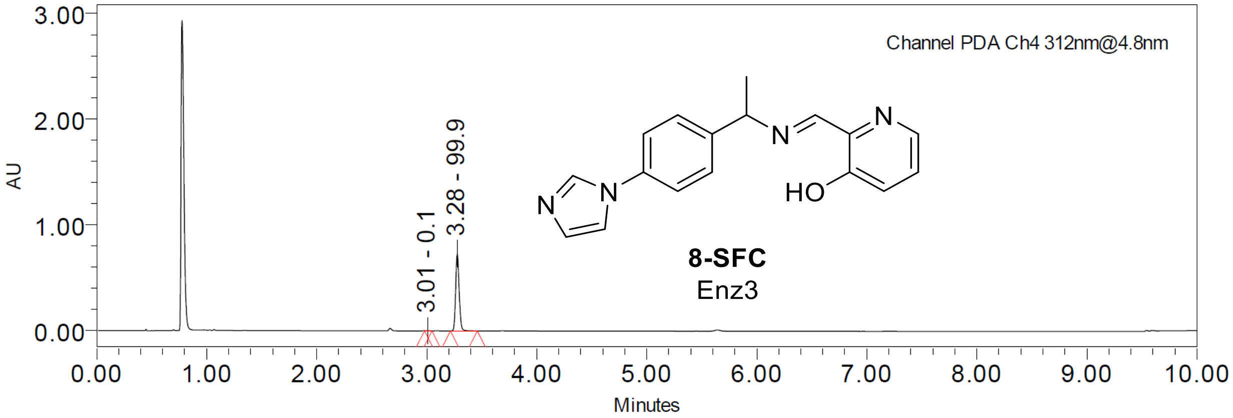
Channel PDA Ch4 312nm@4.8nm
3.01 - 0.1
3.28 - 99.9
8-SFC
Enz3
AU
Minutes

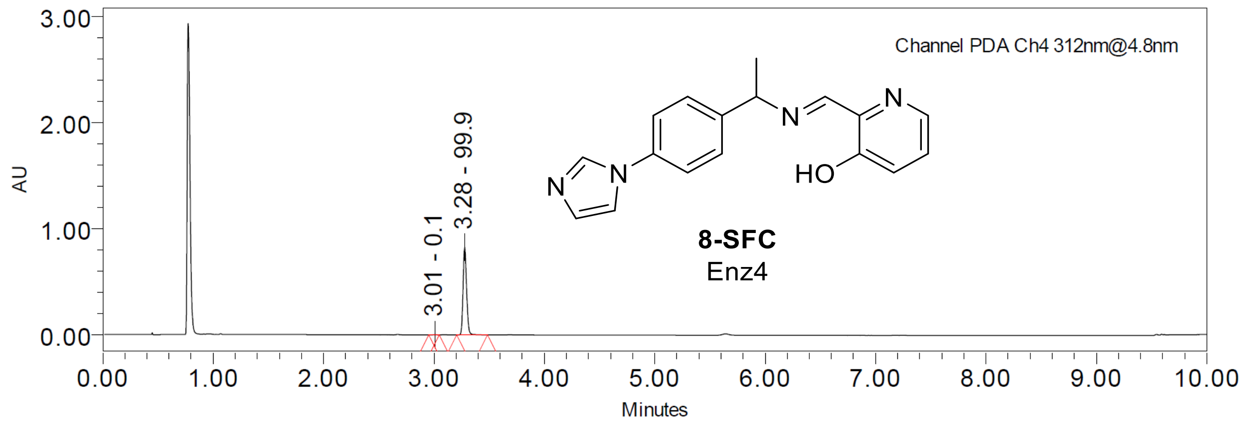

Channel PDA Ch4 312nm@4.8nm
AU
Minutes
3.01 - 0.1
3.28 - 99.9
8-SFC
Enz4

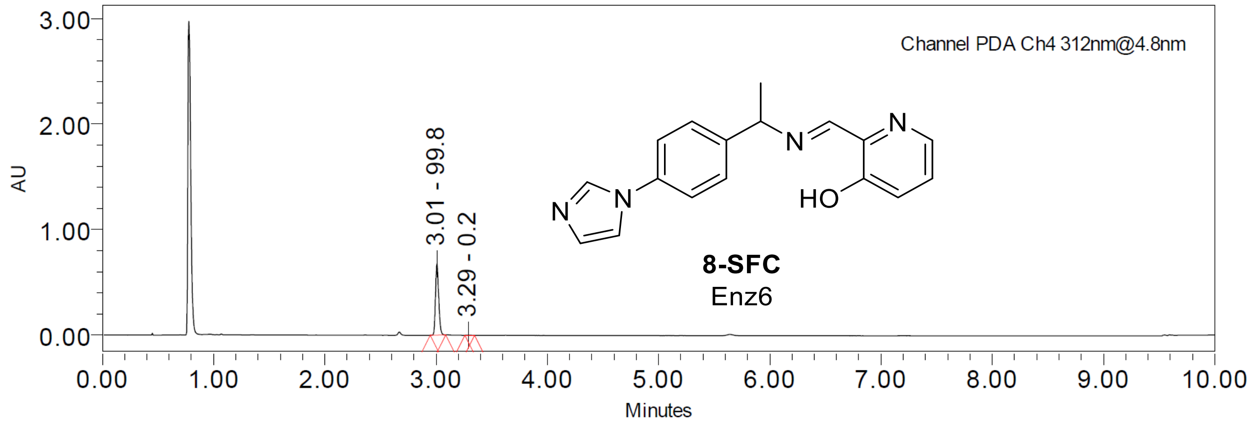

Channel PDA Ch4 312nm@4.8nm
AU
Minutes
3.01 - 99.8
3.29 - 0.2
8-SFC
Enz6

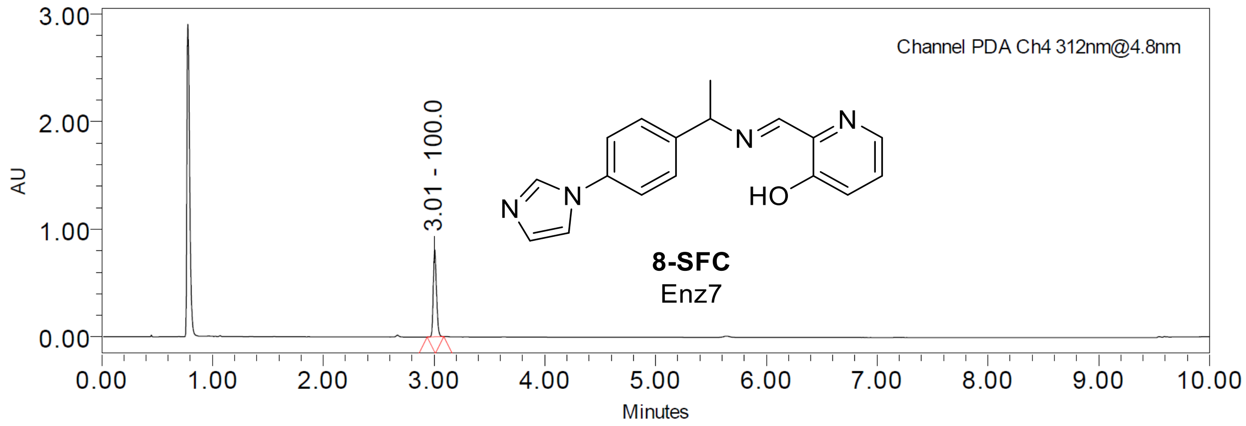

Channel PDA Ch4 312nm@4.8nm
AU
Minutes
3.01 - 100.0
8-SFC
Enz7

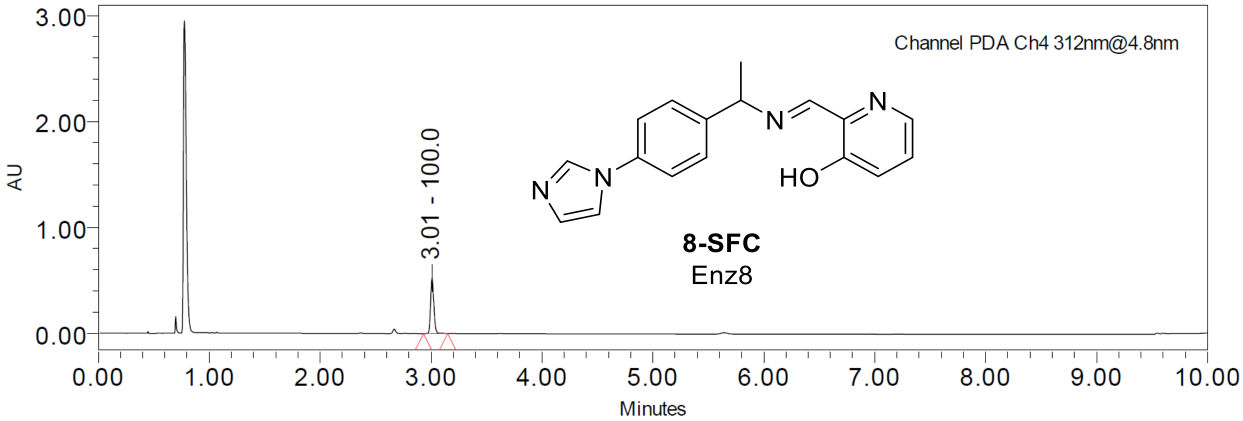
Channel PDA Ch4 312nm@4.8nm
AU
Minutes
3.01 - 100.0
N
N
N
HO
N
8-SFC
Enz8

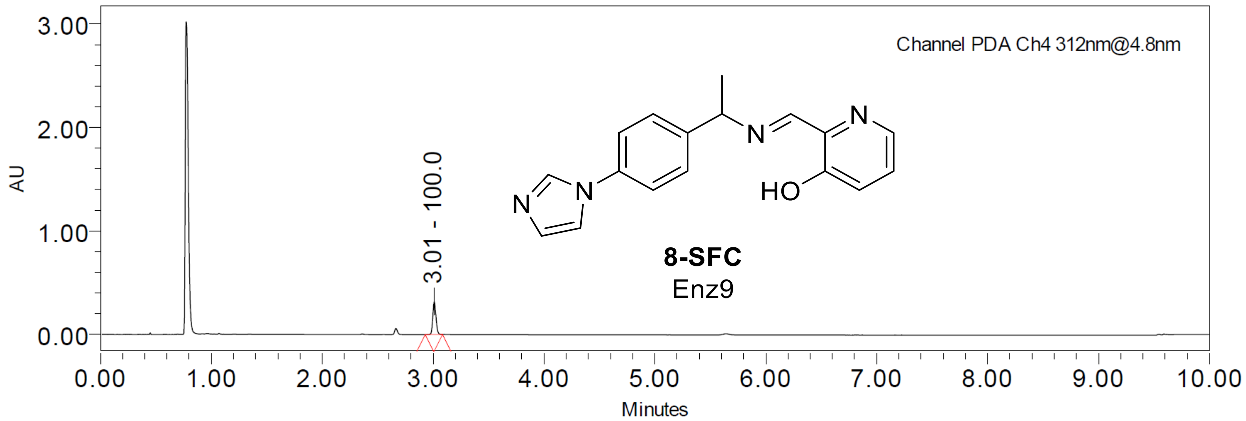
Channel PDA Ch4 312nm@4.8nm
AU
Minutes
3.01 - 100.0
N
N
N
HO
N
8-SFC
Enz9

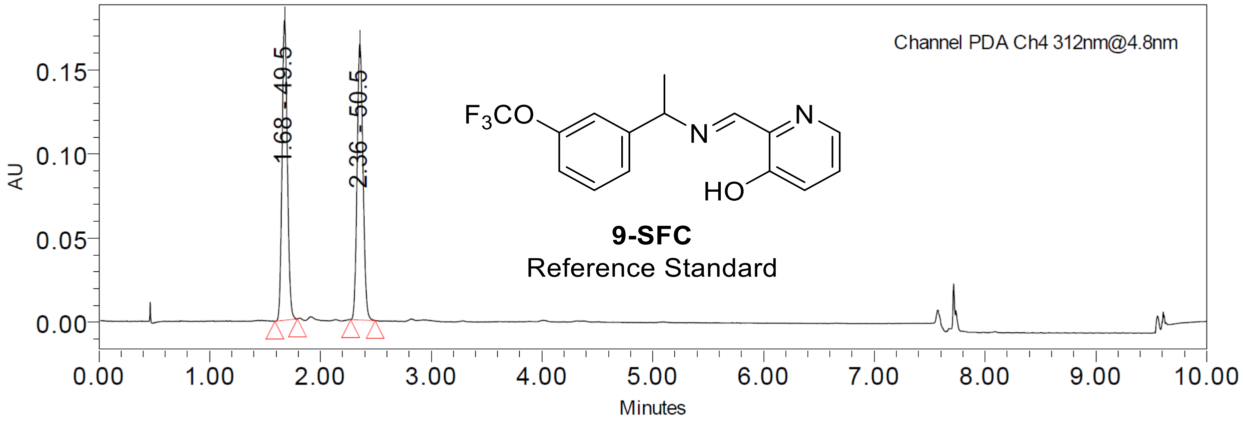
Channel PDA Ch4 312nm@4.8nm
AU
Minutes
1.68 - 49.5
2.36 - 50.5
F_3CO
N
N
HO
9-SFC
Reference Standard

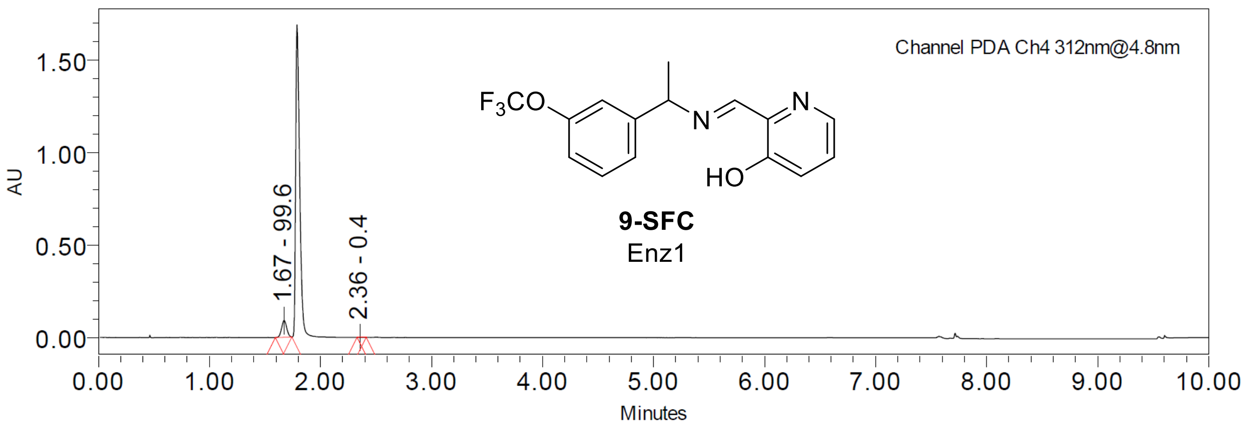
Channel PDA Ch4 312nm@4.8nm
F_3CO
N
N
HO
9-SFC
Enz1
1.67 - 99.6
2.36 - 0.4
AU
Minutes

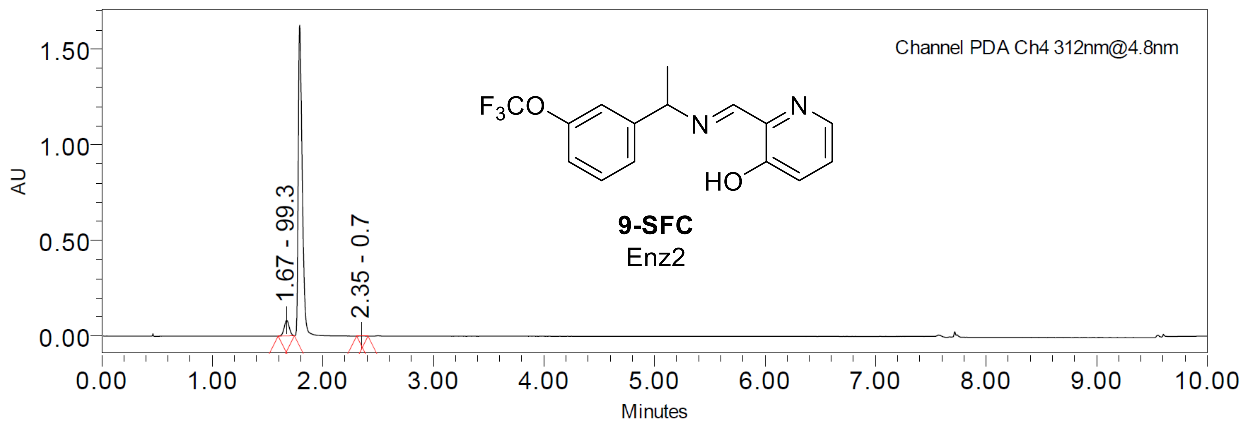
Channel PDA Ch4 312nm@4.8nm
F_3CO
N
N
HO
9-SFC
Enz2
1.67 - 99.3
2.35 - 0.7
AU
Minutes

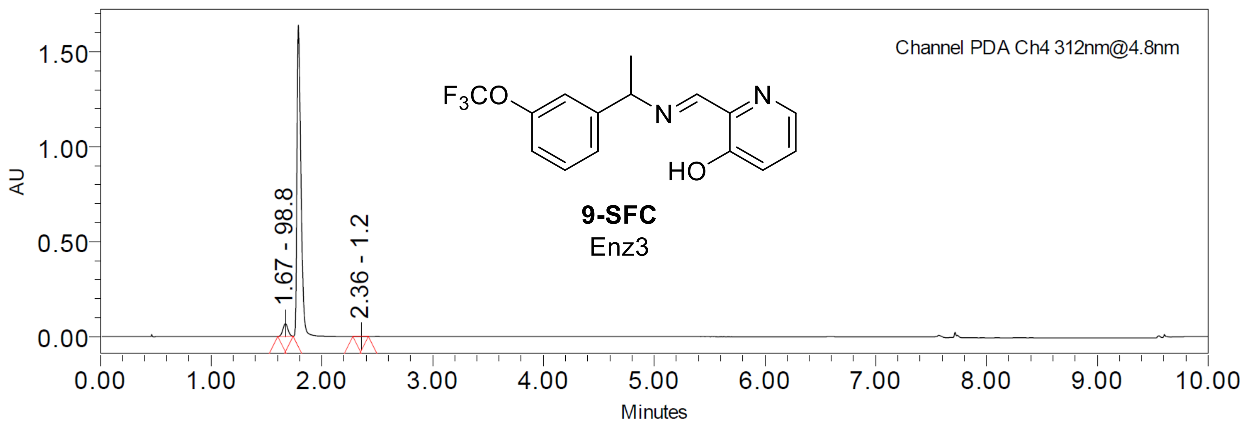
Channel PDA Ch4 312nm@4.8nm
F_3CO
N
N
HO
9-SFC
Enz3
1.67 - 98.8
2.36 - 1.2
AU
Minutes

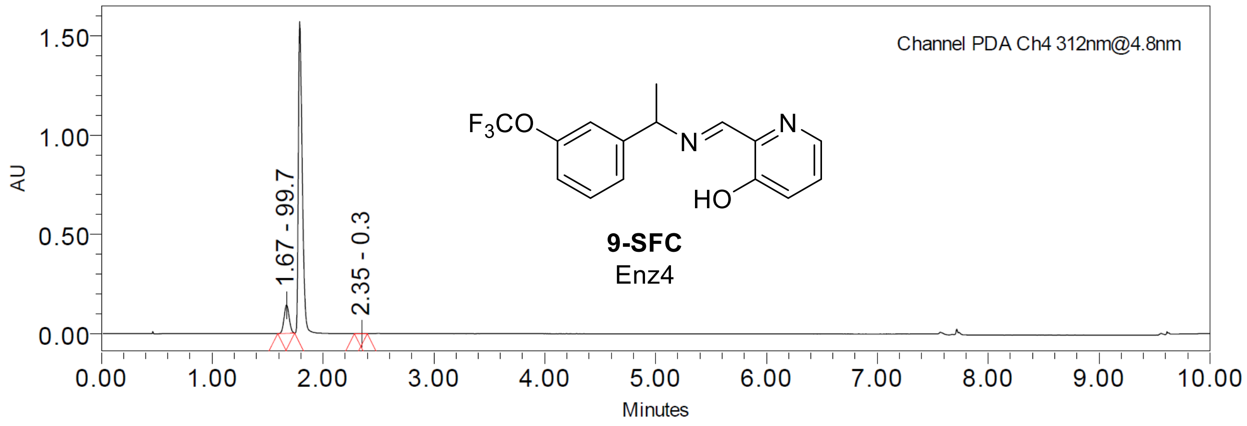

Channel PDA Ch4 312nm@4.8nm
9-SFC
Enz4
1.67 - 99.7
2.35 - 0.3
AU
Minutes

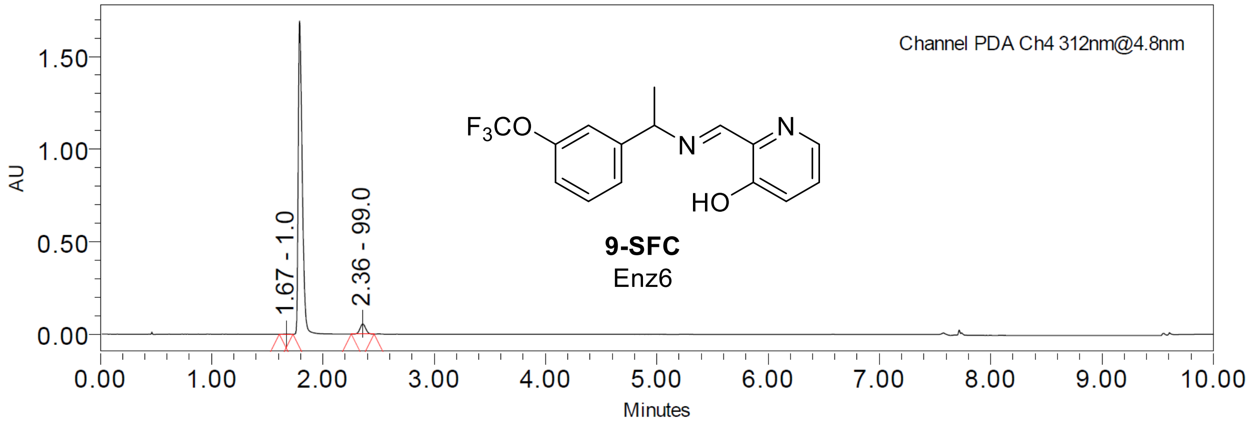

Channel PDA Ch4 312nm@4.8nm
9-SFC
Enz6
1.67 - 1.0
2.36 - 99.0
AU
Minutes

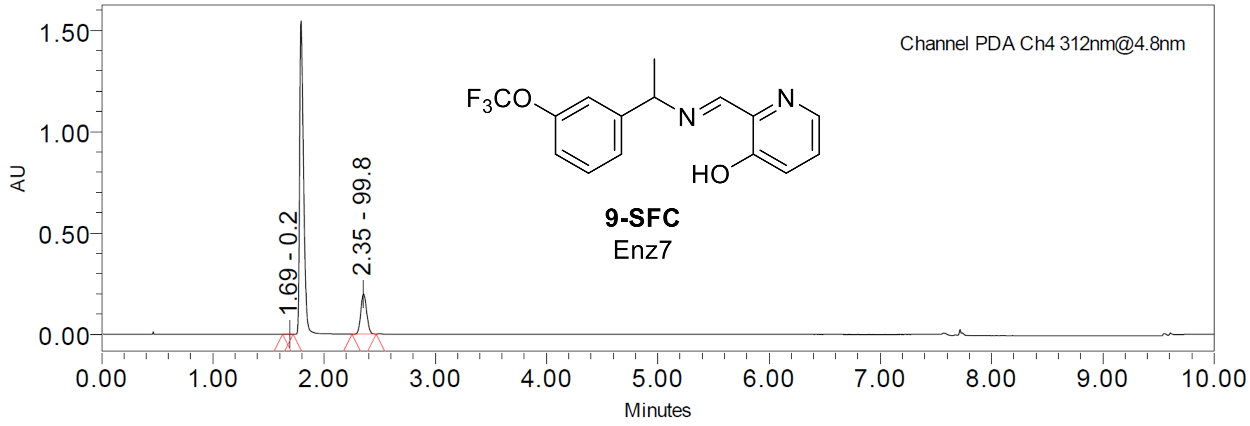

Channel PDA Ch4 312nm@4.8nm
9-SFC
Enz7
1.69 - 0.2
2.35 - 99.8
AU
Minutes

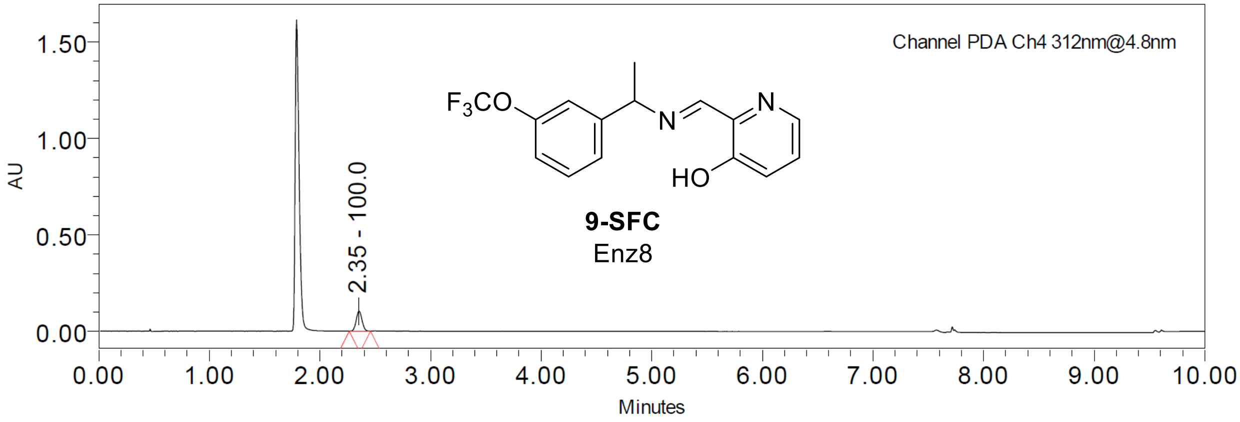
Channel PDA Ch4 312nm@4.8nm
AU
2.35 - 100.0
9-SFC
Enz8
Minutes

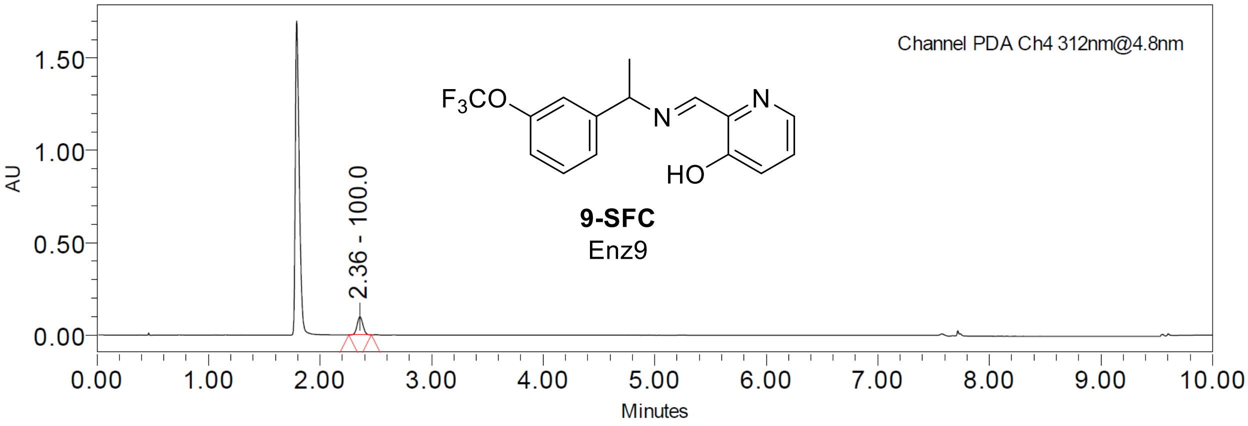
Channel PDA Ch4 312nm@4.8nm
AU
2.36 - 100.0
9-SFC
Enz9
Minutes

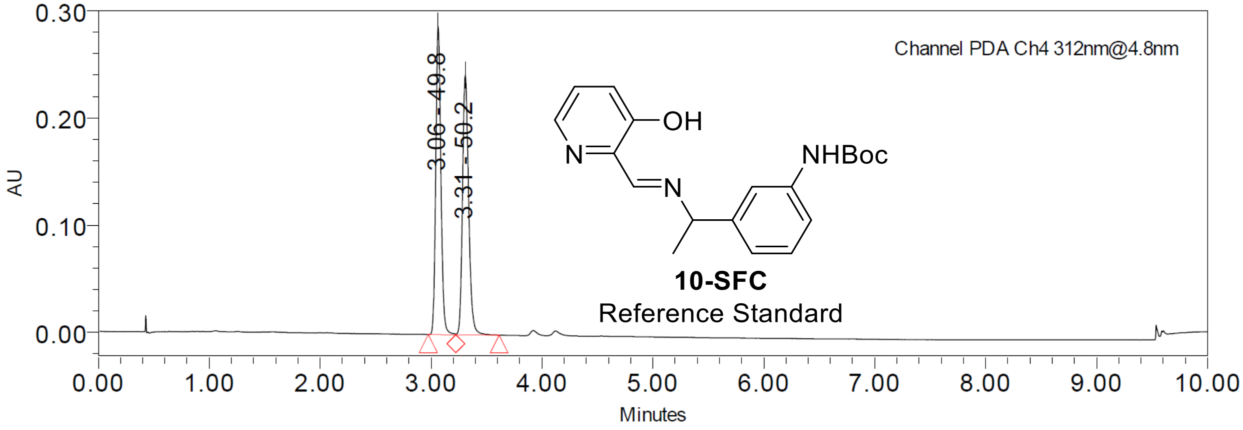
Channel PDA Ch4 312nm@4.8nm
AU
3.06 - 49.8
3.31 - 50.2
10-SFC
Reference Standard
Minutes

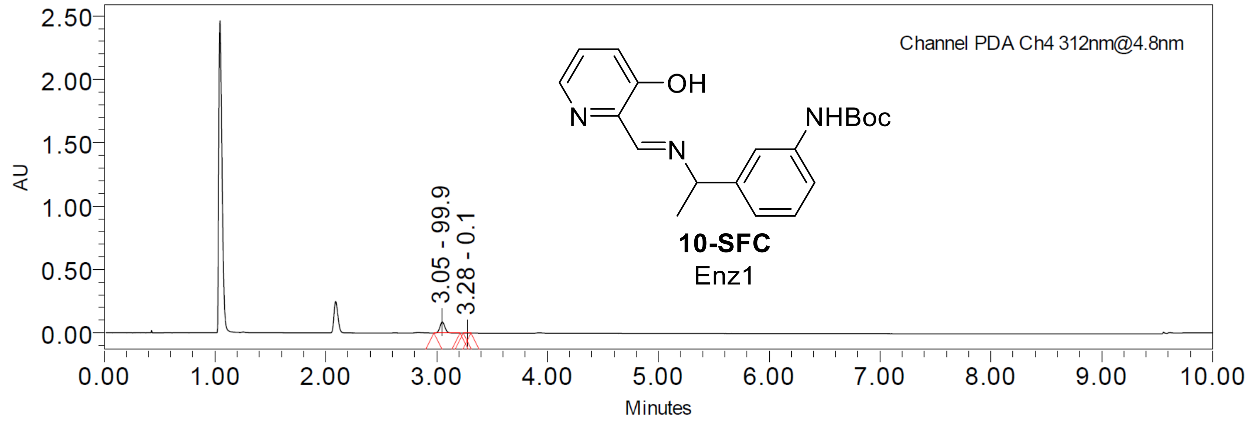
Channel PDA Ch4 312nm@4.8nm
OH
N
N
NHBoc
10-SFC
Enz1
3.05 - 99.9
3.28 - 0.1
AU
Minutes

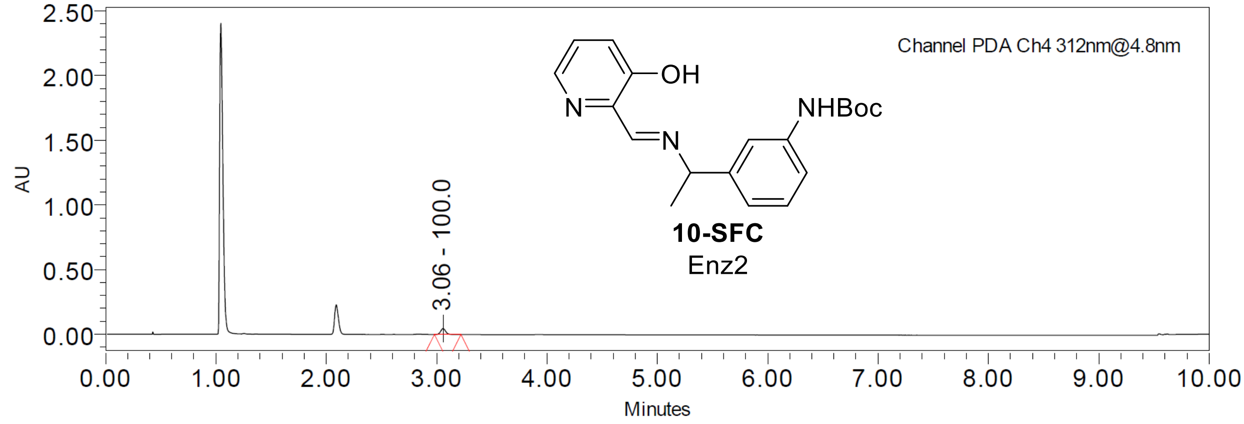
Channel PDA Ch4 312nm@4.8nm
OH
N
N
NHBoc
10-SFC
Enz2
3.06 - 100.0
AU
Minutes

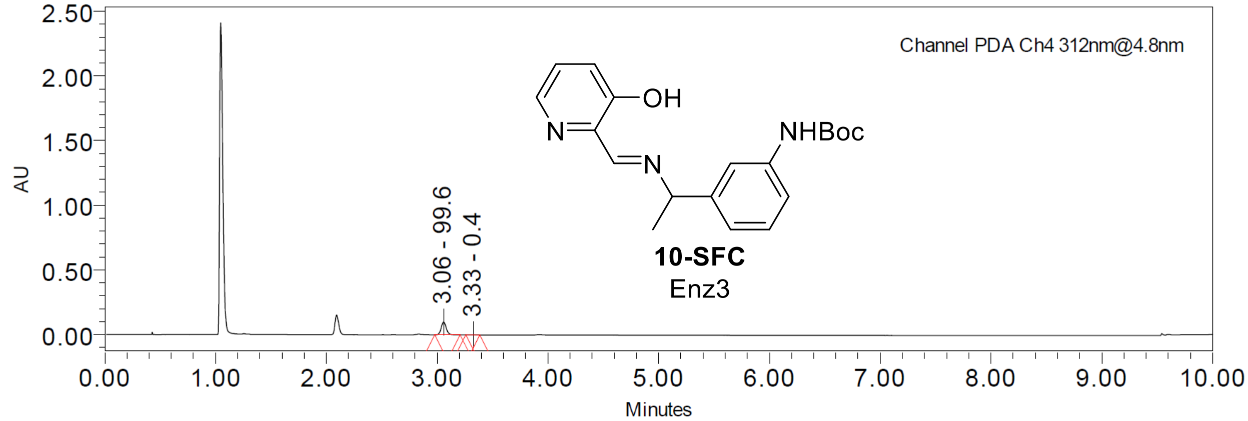
Channel PDA Ch4 312nm@4.8nm
OH
N
N
NHBoc
10-SFC
Enz3
3.06 - 99.6
3.33 - 0.4
AU
Minutes

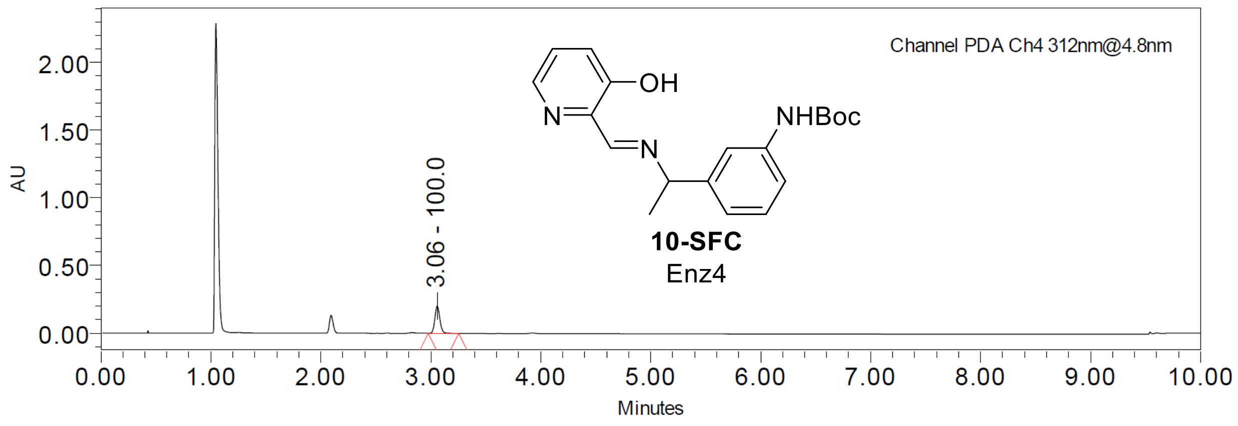
Channel PDA Ch4 312nm@4.8nm
OH
N
N
NHBoc
10-SFC
Enz4
3.06 - 100.0
AU
2.00
1.50
1.00
0.50
0.00
0.00 1.00 2.00 3.00 4.00 5.00 6.00 7.00 8.00 9.00 10.00
Minutes

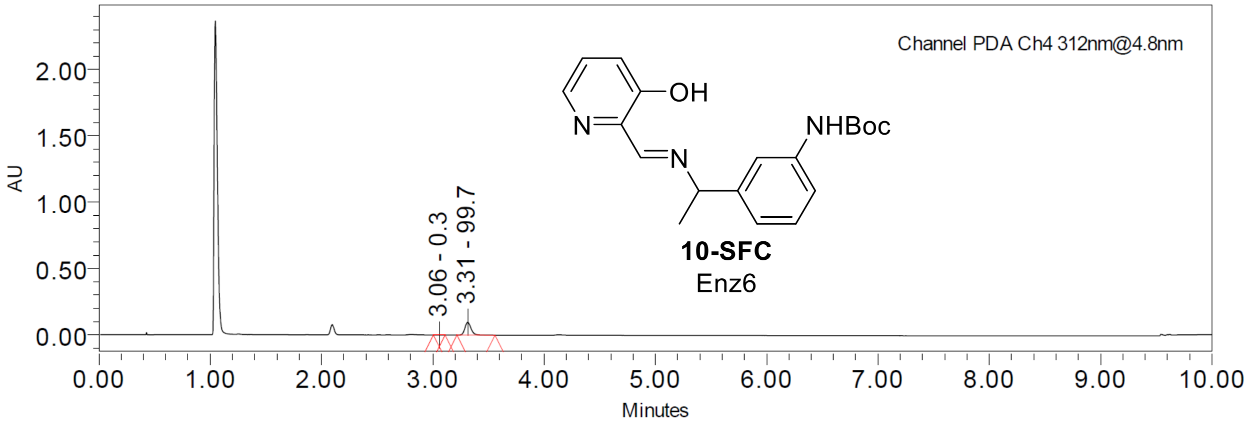
Channel PDA Ch4 312nm@4.8nm
OH
N
N
NHBoc
10-SFC
Enz6
3.06 - 0.3
3.31 - 99.7
AU
2.00
1.50
1.00
0.50
0.00
0.00 1.00 2.00 3.00 4.00 5.00 6.00 7.00 8.00 9.00 10.00
Minutes

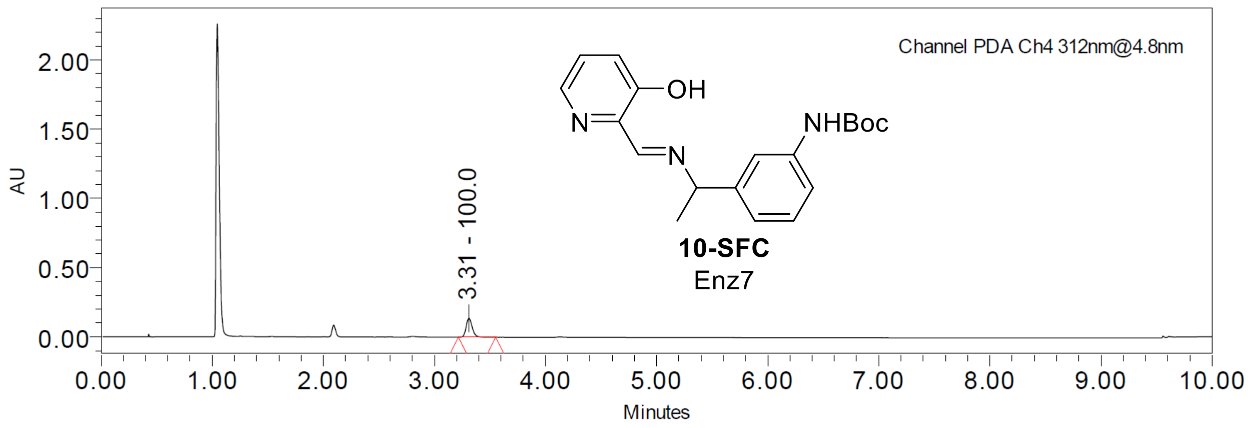
Channel PDA Ch4 312nm@4.8nm
OH
N
N
NHBoc
10-SFC
Enz7
3.31 - 100.0
AU
2.00
1.50
1.00
0.50
0.00
0.00 1.00 2.00 3.00 4.00 5.00 6.00 7.00 8.00 9.00 10.00
Minutes

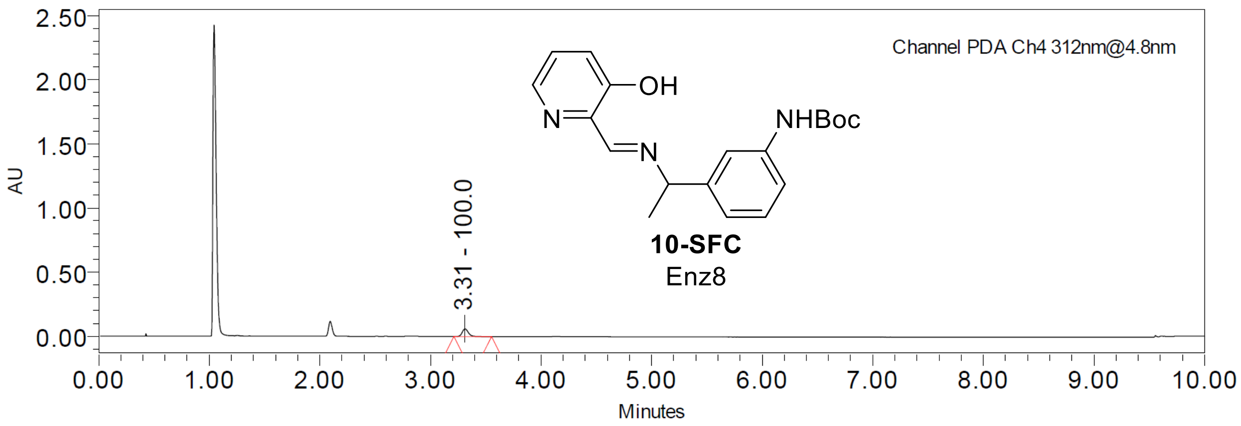
Channel PDA Ch4 312nm@4.8nm
OH
N
NHBoc
N
3.31 - 100.0
10-SFC
Enz8
AU
Minutes
2.50
2.00
1.50
1.00
0.50
0.00
0.00
1.00
2.00
3.00
4.00
5.00
6.00
7.00
8.00
9.00
10.00

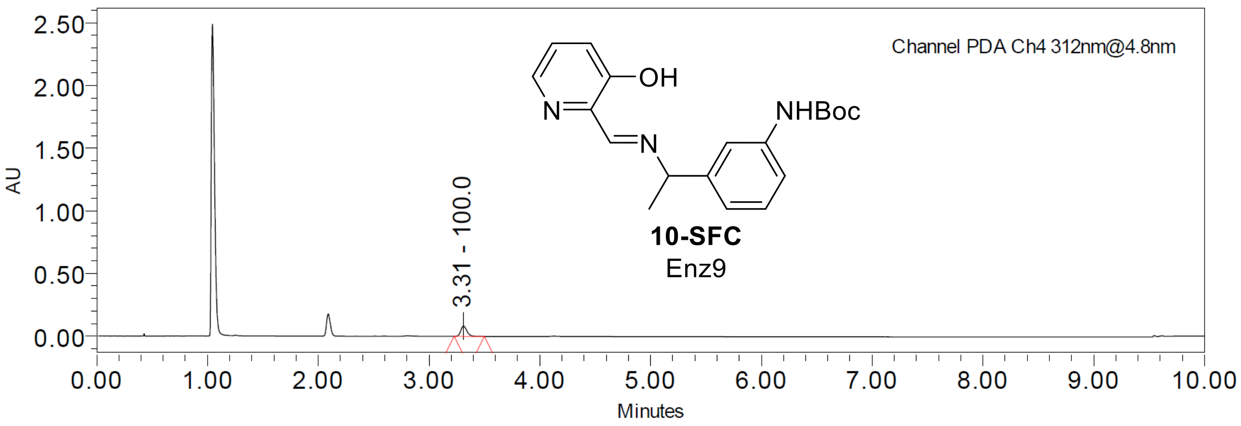
Channel PDA Ch4 312nm@4.8nm
OH
N
NHBoc
N
3.31 - 100.0
10-SFC
Enz9
AU
Minutes
2.50
2.00
1.50
1.00
0.50
0.00
0.00
1.00
2.00
3.00
4.00
5.00
6.00
7.00
8.00
9.00
10.00

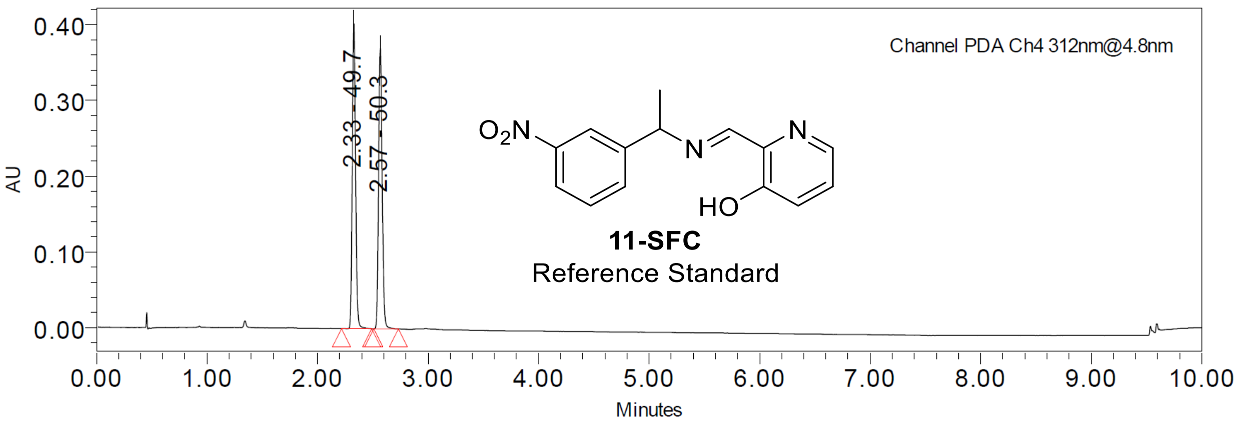
Channel PDA Ch4 312nm@4.8nm
2.33 - 49.7
2.57 - 50.3
O2N
N
N
HO
11-SFC
Reference Standard
AU
Minutes
0.40
0.30
0.20
0.10
0.00
0.00
1.00
2.00
3.00
4.00
5.00
6.00
7.00
8.00
9.00
10.00

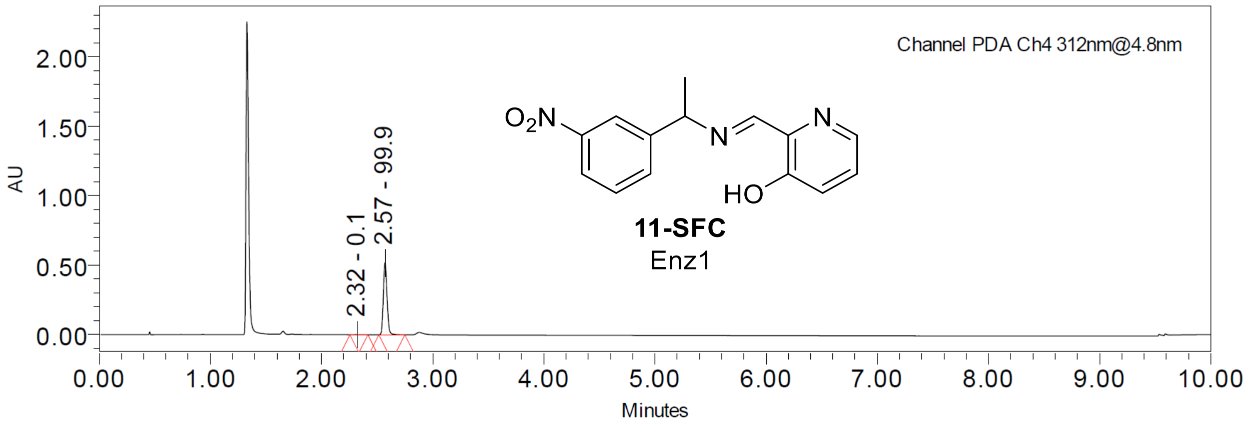
Channel PDA Ch4 312nm@4.8nm
AU
2.32 - 0.1
2.57 - 99.9
11-SFC
Enz1
Minutes

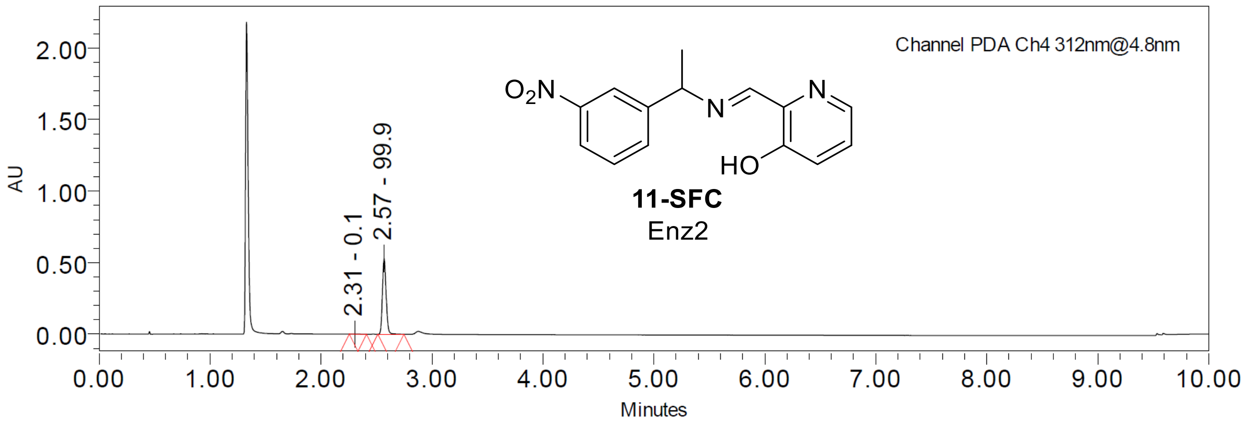
Channel PDA Ch4 312nm@4.8nm
AU
2.31 - 0.1
2.57 - 99.9
11-SFC
Enz2
Minutes

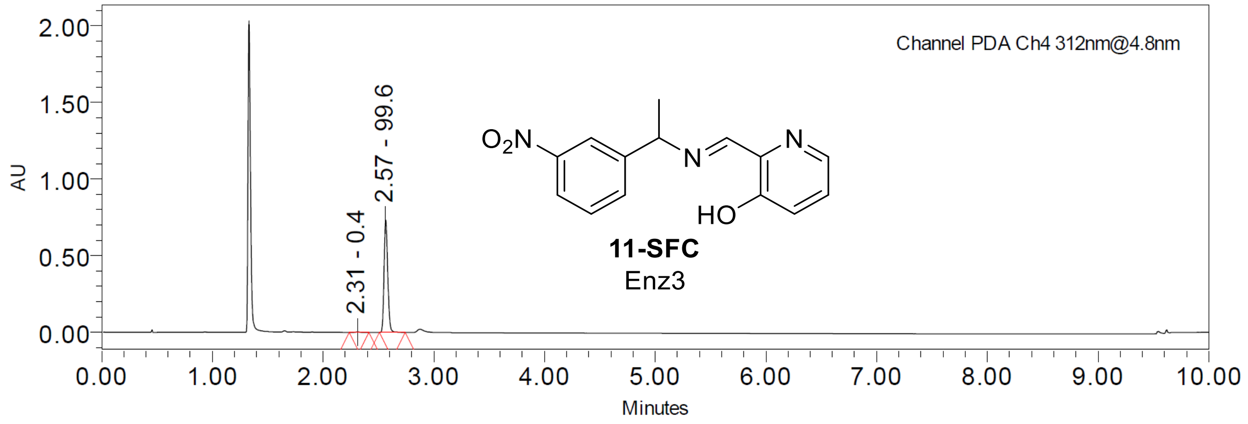
Channel PDA Ch4 312nm@4.8nm
AU
2.31 - 0.4
2.57 - 99.6
11-SFC
Enz3
Minutes

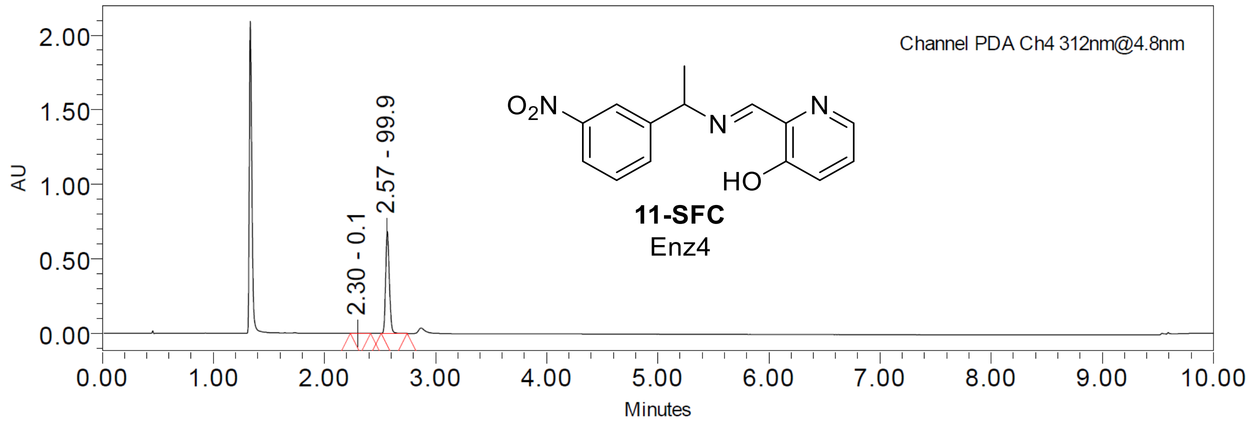

Channel PDA Ch4 312nm@4.8nm
AU
2.00
1.50
1.00
0.50
0.00
2.30 - 0.1
2.57 - 99.9
O_2N
N
N
HO
11-SFC
Enz4
0.00
1.00
2.00
3.00
4.00
5.00
6.00
7.00
8.00
9.00
10.00
Minutes

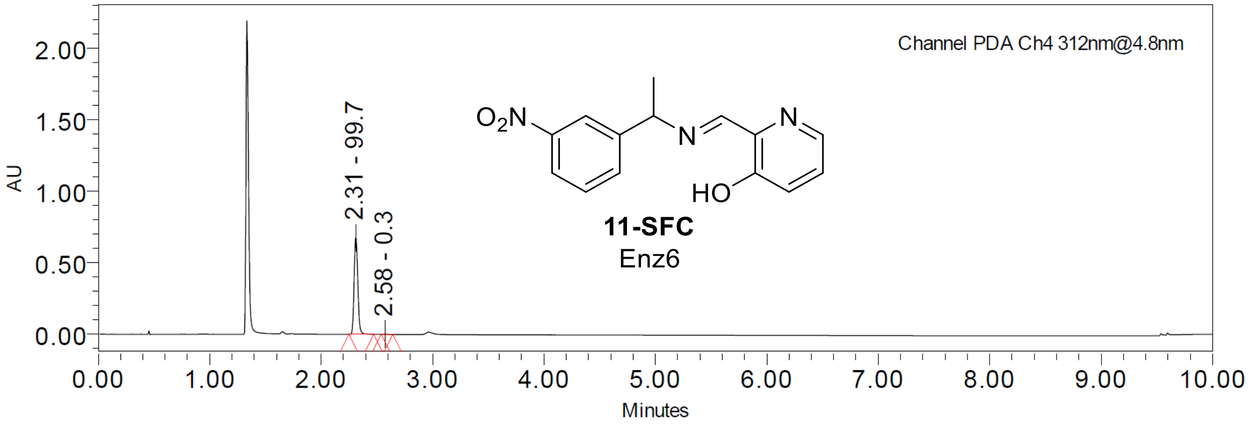

Channel PDA Ch4 312nm@4.8nm
AU
2.00
1.50
1.00
0.50
0.00
2.31 - 99.7
2.58 - 0.3
O_2N
N
N
HO
11-SFC
Enz6
0.00
1.00
2.00
3.00
4.00
5.00
6.00
7.00
8.00
9.00
10.00
Minutes

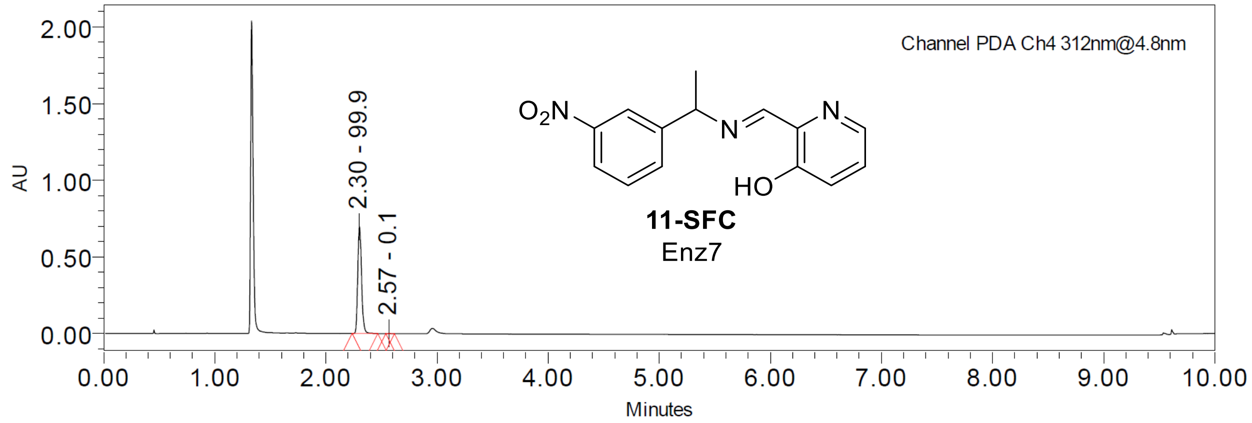

Channel PDA Ch4 312nm@4.8nm
AU
2.00
1.50
1.00
0.50
0.00
2.30 - 99.9
2.57 - 0.1
O_2N
N
N
HO
11-SFC
Enz7
0.00
1.00
2.00
3.00
4.00
5.00
6.00
7.00
8.00
9.00
10.00
Minutes

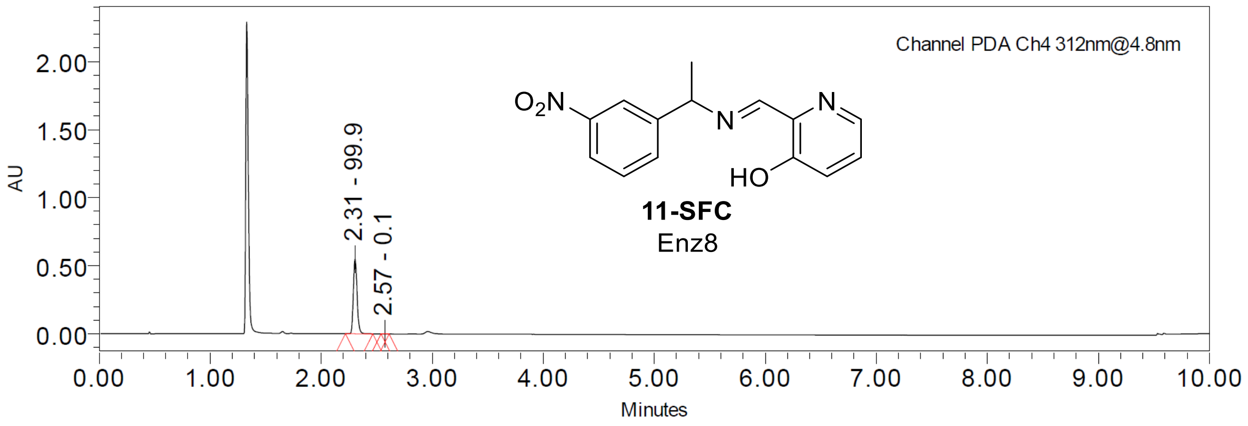
Channel PDA Ch4 312nm@4.8nm
AU
Minutes
2.31 - 99.9
2.57 - 0.1
11-SFC
Enz8

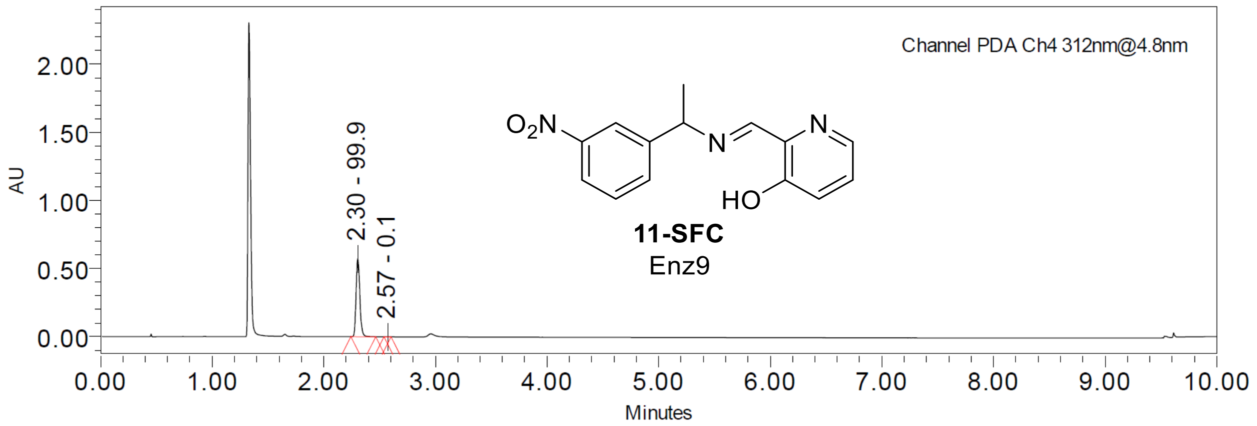
Channel PDA Ch4 312nm@4.8nm
AU
Minutes
2.30 - 99.9
2.57 - 0.1
11-SFC
Enz9

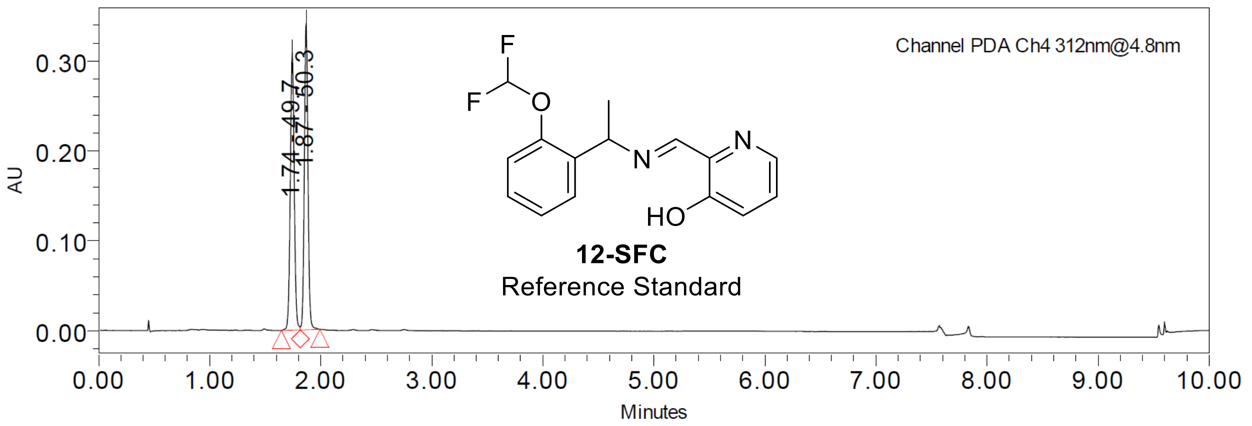
Channel PDA Ch4 312nm@4.8nm
AU
Minutes
1.74 - 49.7
1.87 - 50.3
12-SFC
Reference Standard

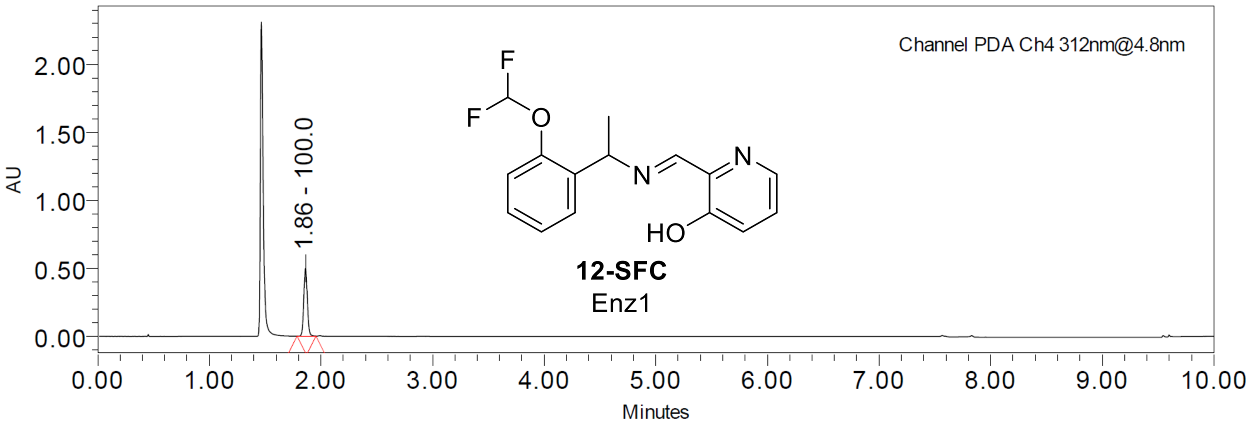
Channel PDA Ch4 312nm@4.8nm
AU
Minutes
1.86 - 100.0
F
F
O
N
N
HO
12-SFC
Enz1

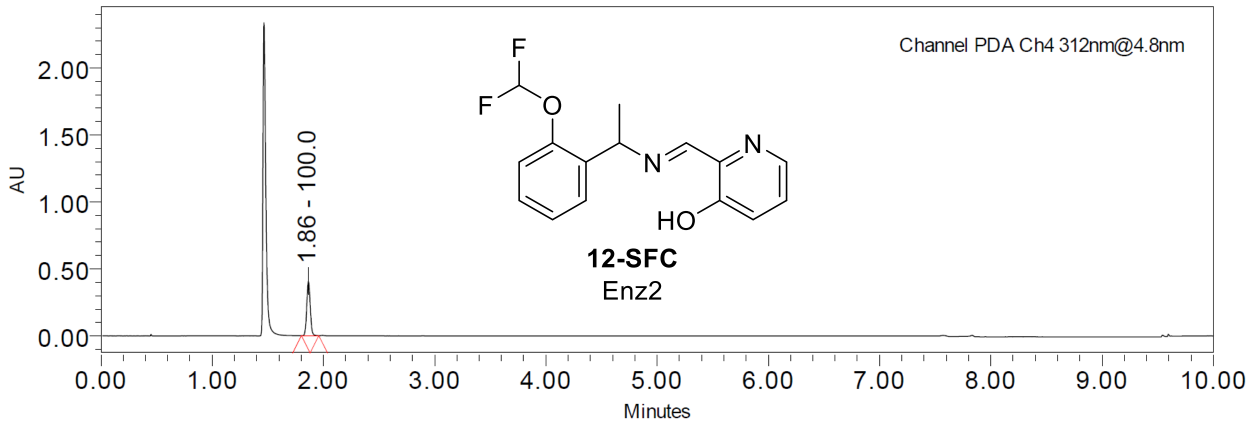
Channel PDA Ch4 312nm@4.8nm
AU
Minutes
1.86 - 100.0
F
F
O
N
N
HO
12-SFC
Enz2

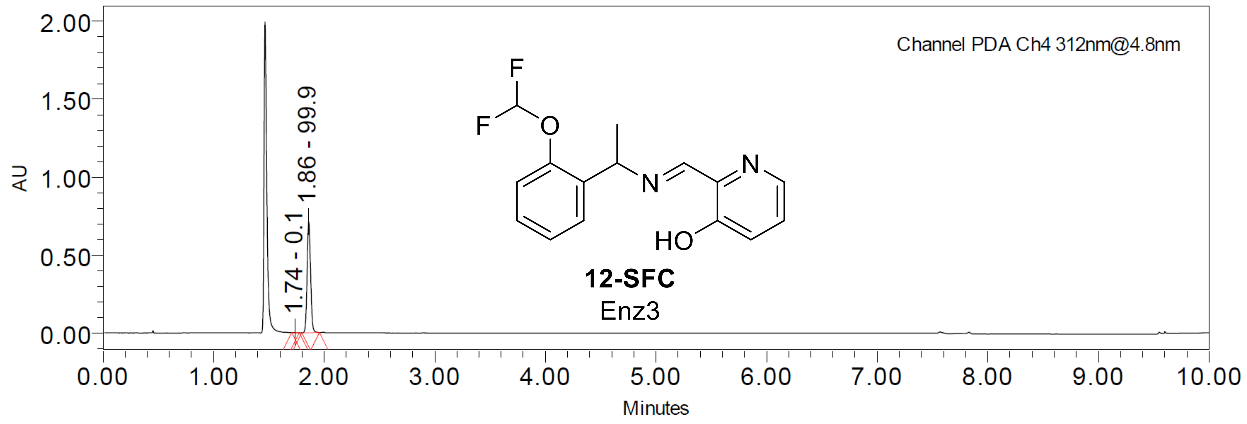
Channel PDA Ch4 312nm@4.8nm
AU
Minutes
1.74 - 0.1
1.86 - 99.9
F
F
O
N
N
HO
12-SFC
Enz3

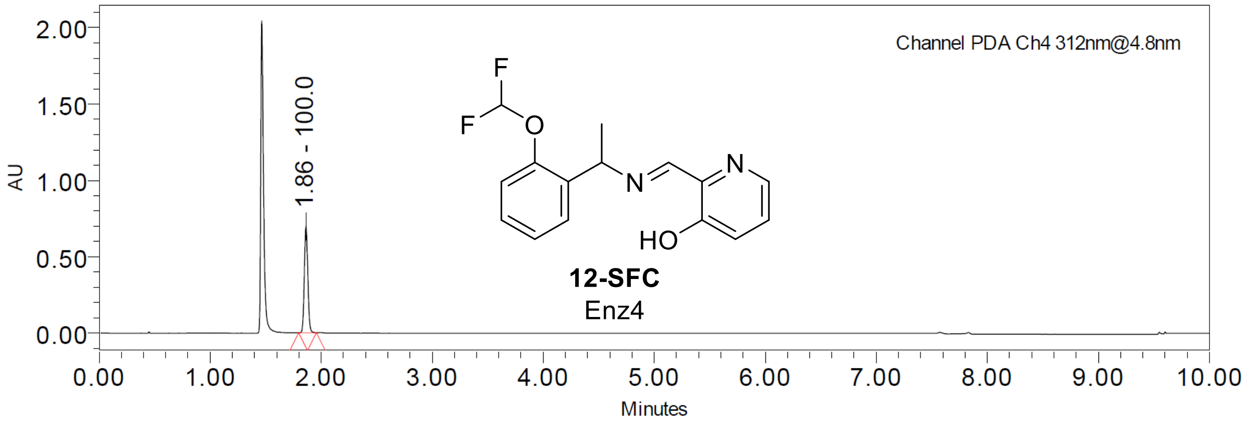

Channel PDA Ch4 312nm@4.8nm
1.86 - 100.0
12-SFC
Enz4
AU
Minutes

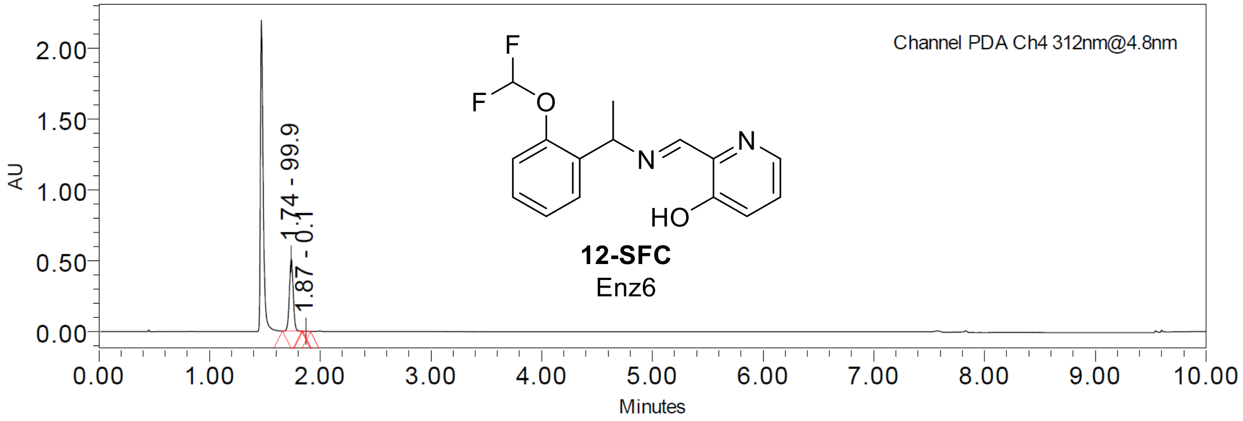

Channel PDA Ch4 312nm@4.8nm
1.74 - 99.9
1.87 - 0.1
12-SFC
Enz6
AU
Minutes

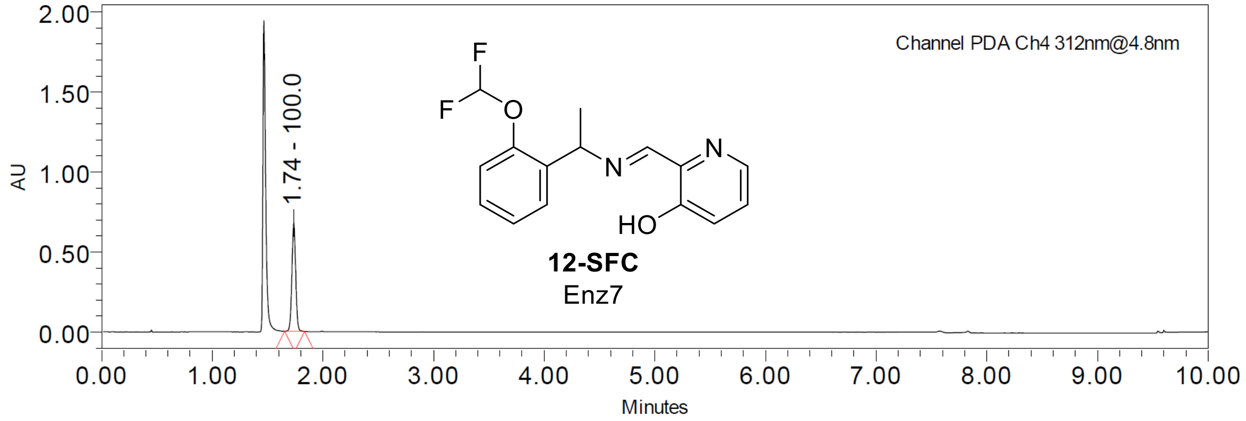

Channel PDA Ch4 312nm@4.8nm
1.74 - 100.0
12-SFC
Enz7
AU
Minutes

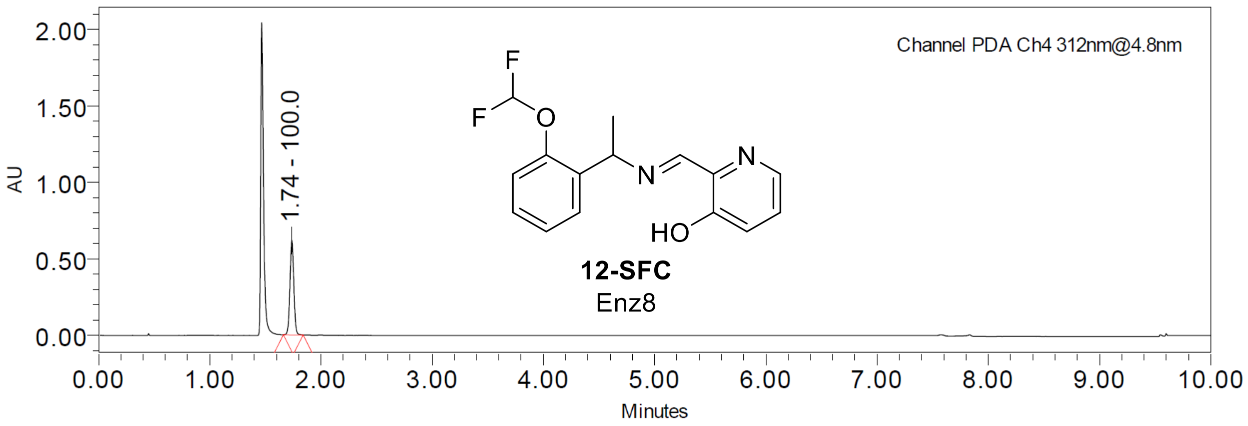

Channel PDA Ch4 312nm@4.8nm
AU
Minutes
1.74 - 100.0
12-SFC
Enz8

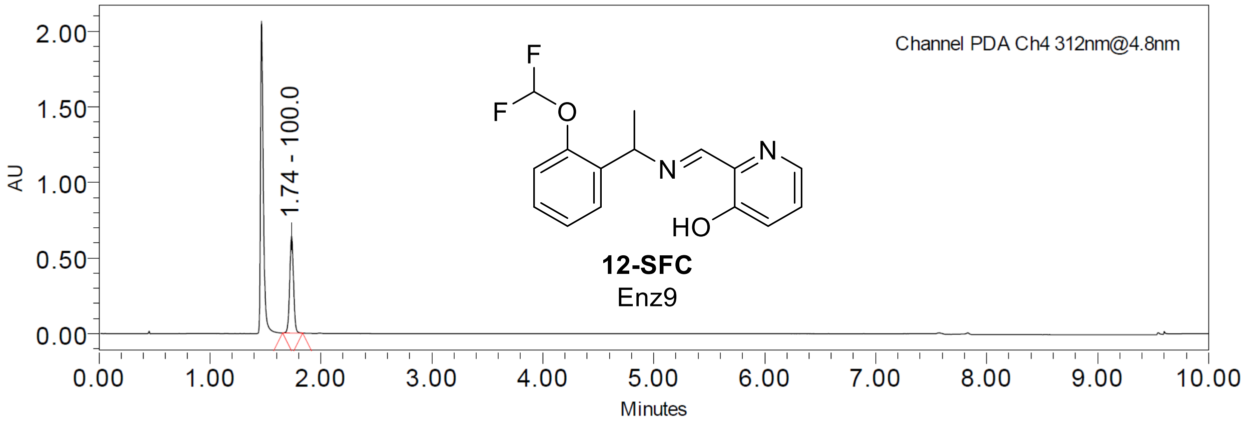

Channel PDA Ch4 312nm@4.8nm
AU
Minutes
1.74 - 100.0
12-SFC
Enz9

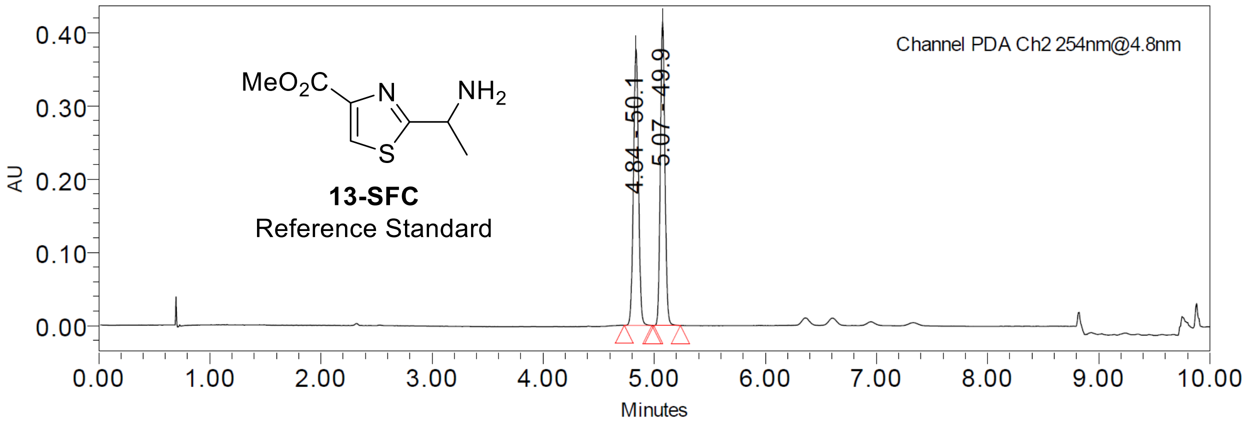

Channel PDA Ch2 254nm@4.8nm
AU
Minutes
4.84 - 50.1
5.07 - 49.9
13-SFC
Reference Standard

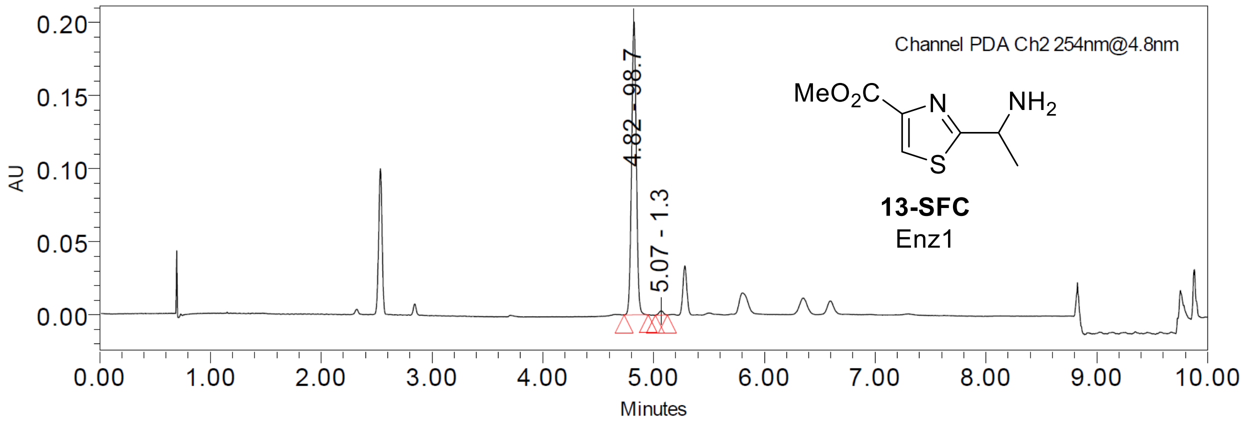
Channel PDA Ch2 254nm@4.8nm
MeO2C
N
NH2
S
13-SFC
Enz1
4.82 - 98.7
5.07 - 1.3
AU
Minutes
0.20
0.15
0.10
0.05
0.00
0.00
1.00
2.00
3.00
4.00
5.00
6.00
7.00
8.00
9.00
10.00

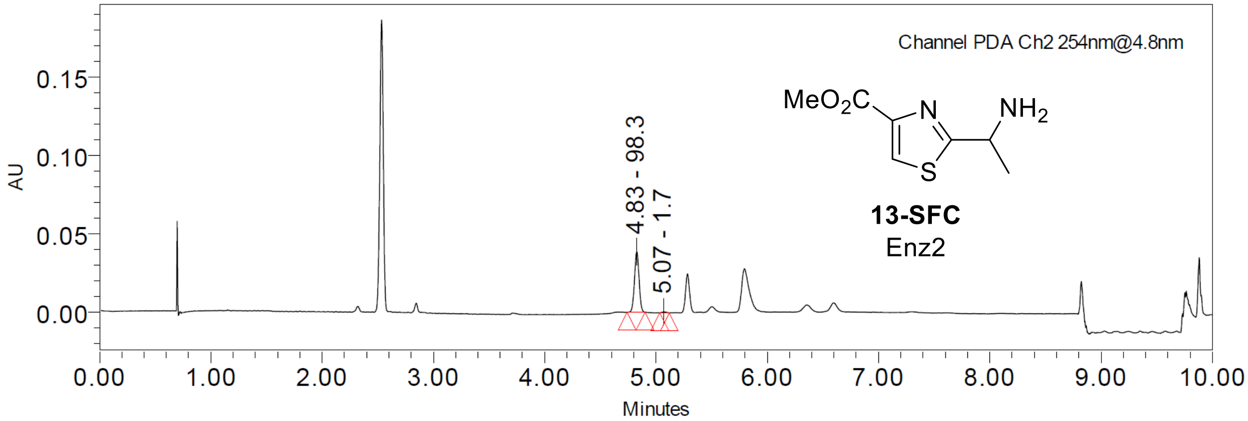
Channel PDA Ch2 254nm@4.8nm
MeO2C
N
NH2
S
13-SFC
Enz2
4.83 - 98.3
5.07 - 1.7
AU
Minutes
0.15
0.10
0.05
0.00
0.00
1.00
2.00
3.00
4.00
5.00
6.00
7.00
8.00
9.00
10.00

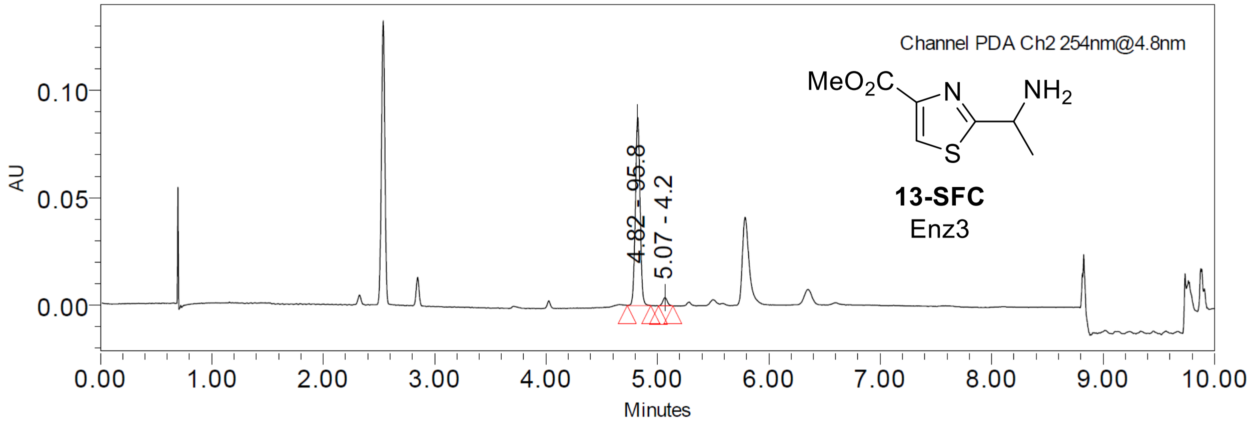
Channel PDA Ch2 254nm@4.8nm
MeO2C
N
NH2
S
13-SFC
Enz3
4.82 - 95.8
5.07 - 4.2
AU
Minutes
0.10
0.05
0.00
0.00
1.00
2.00
3.00
4.00
5.00
6.00
7.00
8.00
9.00
10.00

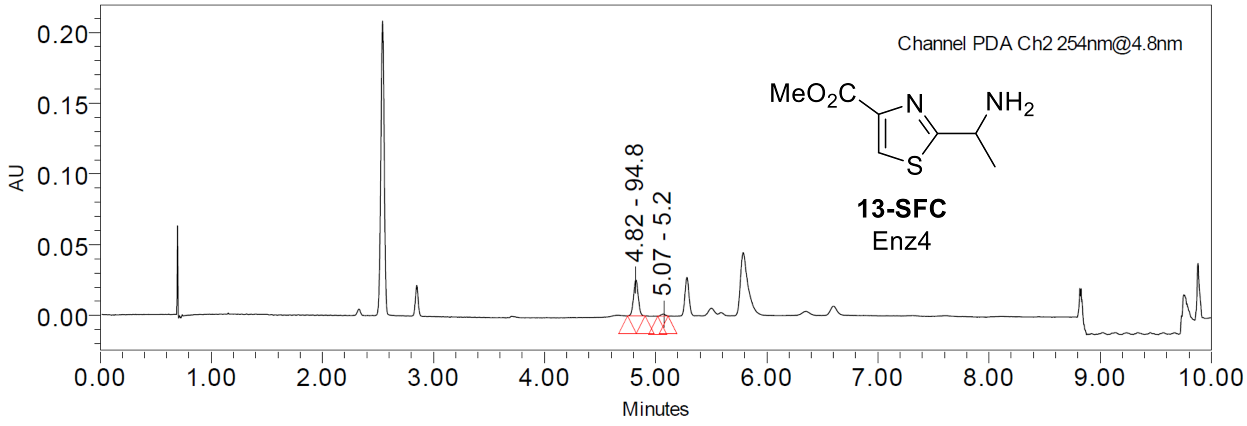
Channel PDA Ch2 254nm@4.8nm
MeO2C
N
NH2
S
13-SFC
Enz4
4.82 - 94.8
5.07 - 5.2
AU
0.20
0.15
0.10
0.05
0.00
0.00
1.00
2.00
3.00
4.00
5.00
6.00
7.00
8.00
9.00
10.00
Minutes

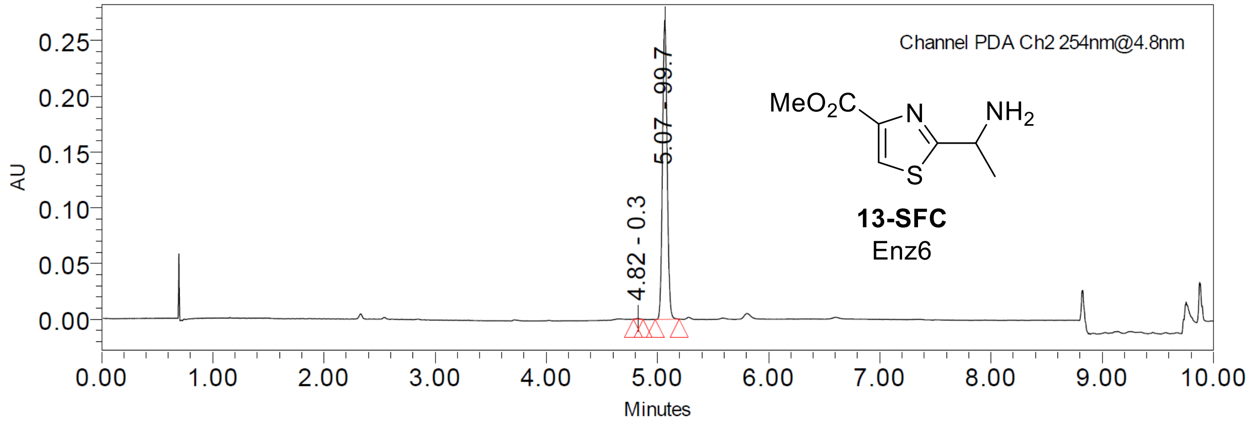
Channel PDA Ch2 254nm@4.8nm
MeO2C
N
NH2
S
13-SFC
Enz6
5.07 - 99.7
4.82 - 0.3
AU
0.25
0.20
0.15
0.10
0.05
0.00
0.00
1.00
2.00
3.00
4.00
5.00
6.00
7.00
8.00
9.00
10.00
Minutes

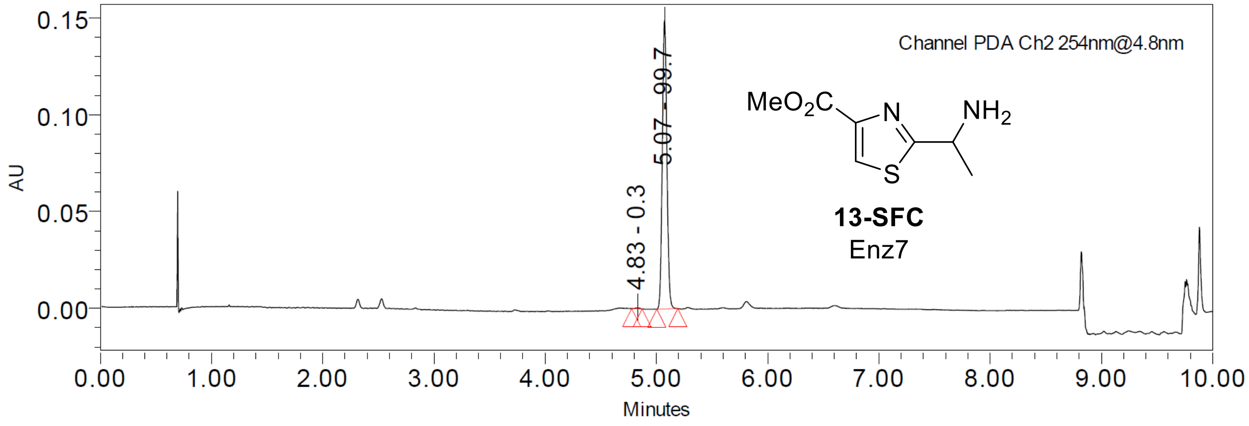
Channel PDA Ch2 254nm@4.8nm
MeO2C
N
NH2
S
13-SFC
Enz7
5.07 - 99.7
4.83 - 0.3
AU
0.15
0.10
0.05
0.00
0.00
1.00
2.00
3.00
4.00
5.00
6.00
7.00
8.00
9.00
10.00
Minutes

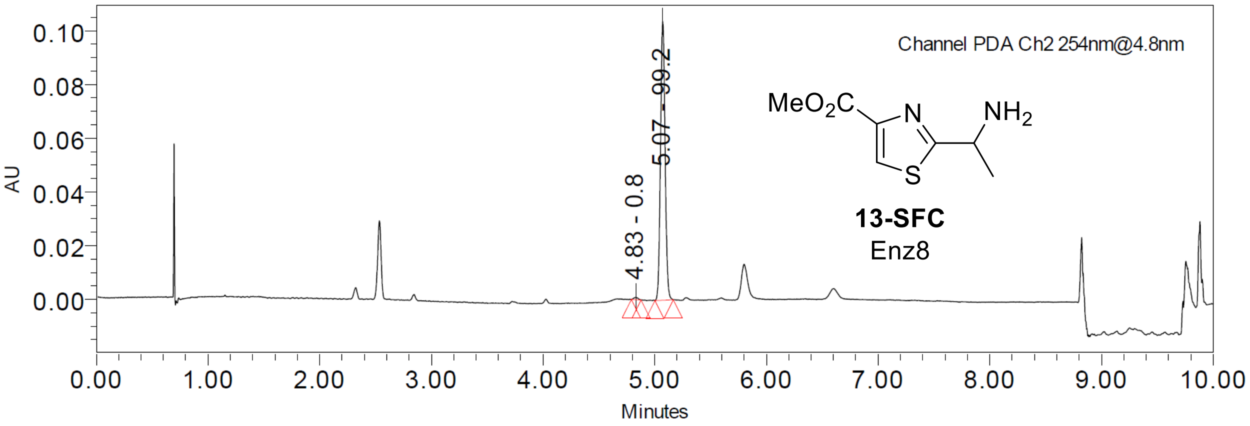
Channel PDA Ch2 254nm@4.8nm
4.83 - 0.8
5.07 - 99.2
13-SFC
Enz8
AU
Minutes

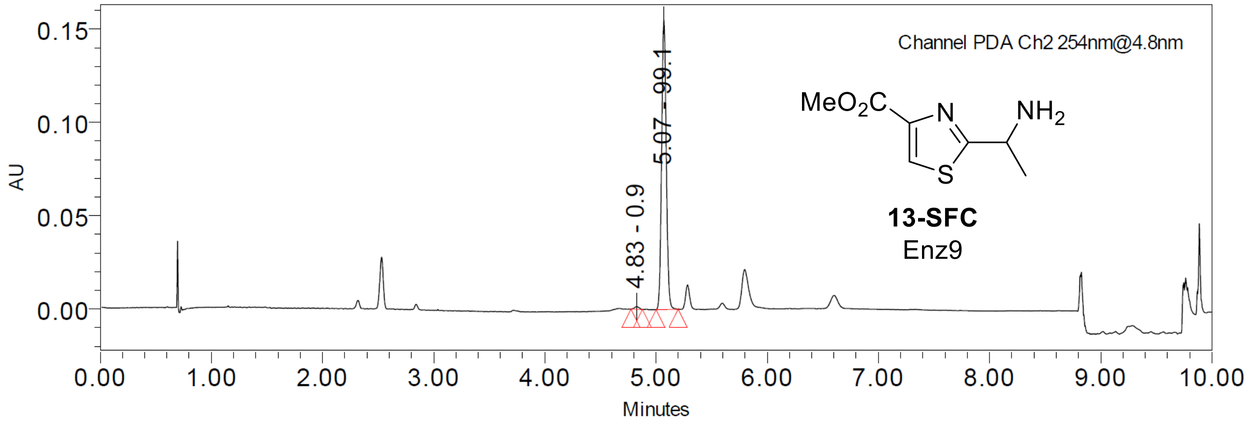
Channel PDA Ch2 254nm@4.8nm
4.83 - 0.9
5.07 - 99.1
13-SFC
Enz9
AU
Minutes

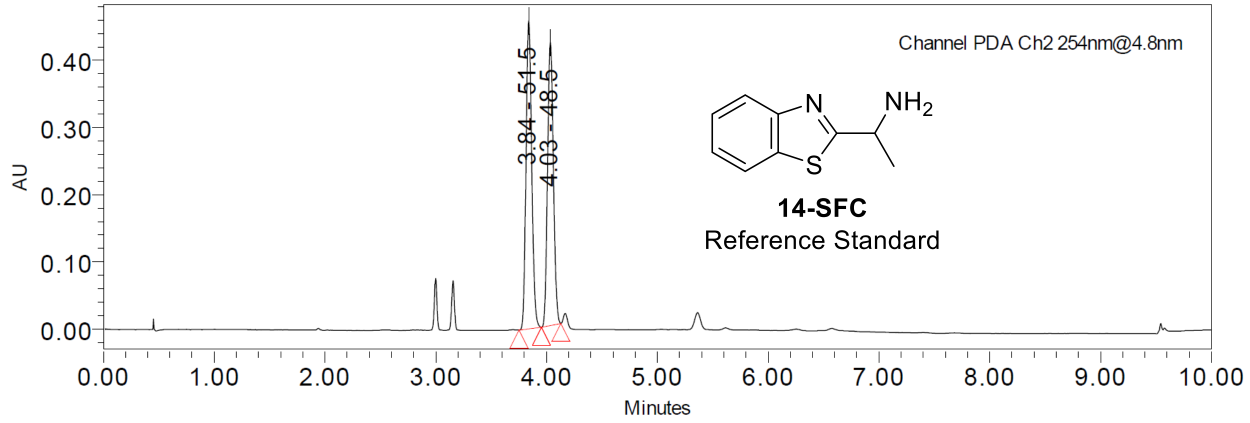
Channel PDA Ch2 254nm@4.8nm
3.84 - 51.5
4.03 - 48.5
14-SFC
Reference Standard
AU
Minutes

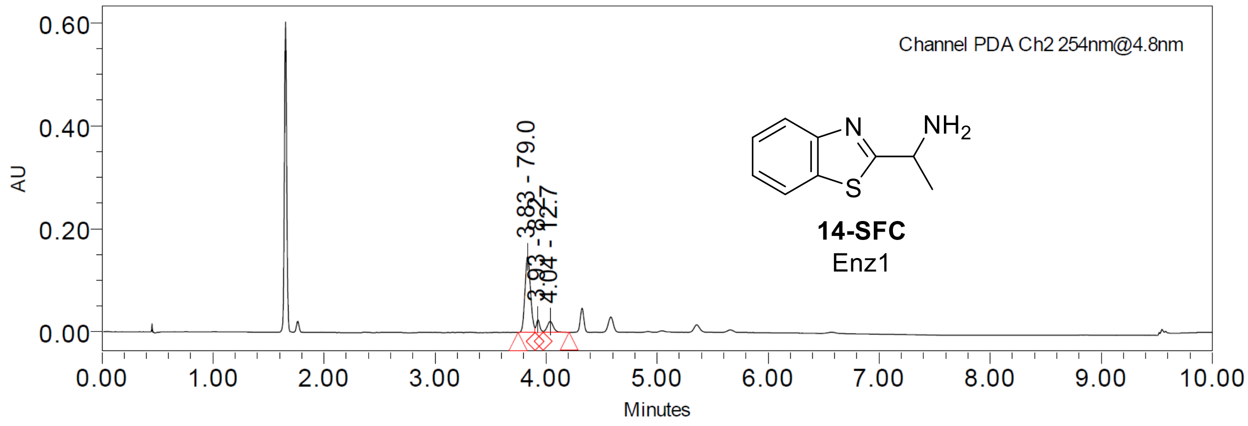
Channel PDA Ch2 254nm@4.8nm
3.83 - 79.0
3.93 - 8.2
4.04 - 12.7
N
NH2
S
14-SFC
Enz1
AU
Minutes

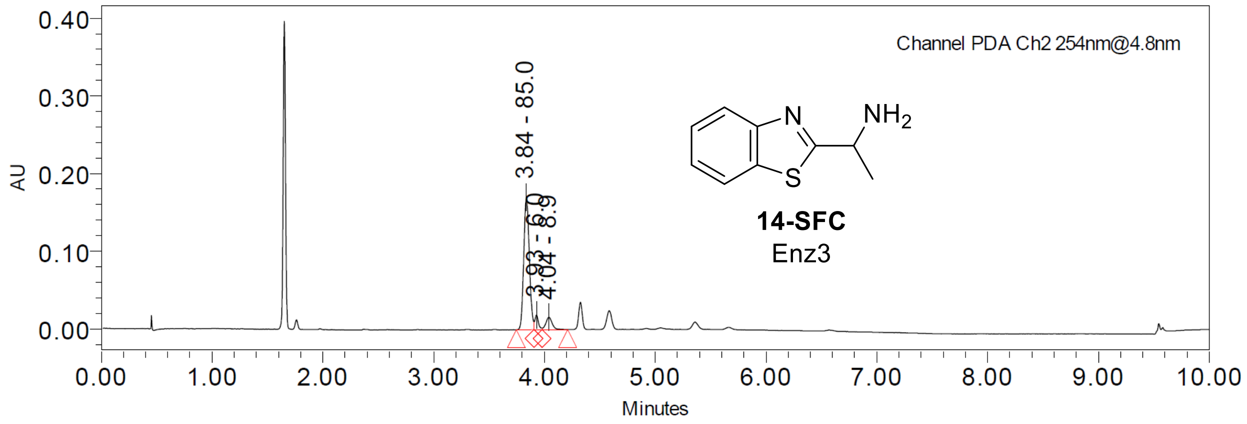
Channel PDA Ch2 254nm@4.8nm
3.84 - 85.0
3.93 - 6.0
4.04 - 8.9
N
NH2
S
14-SFC
Enz3
AU
Minutes

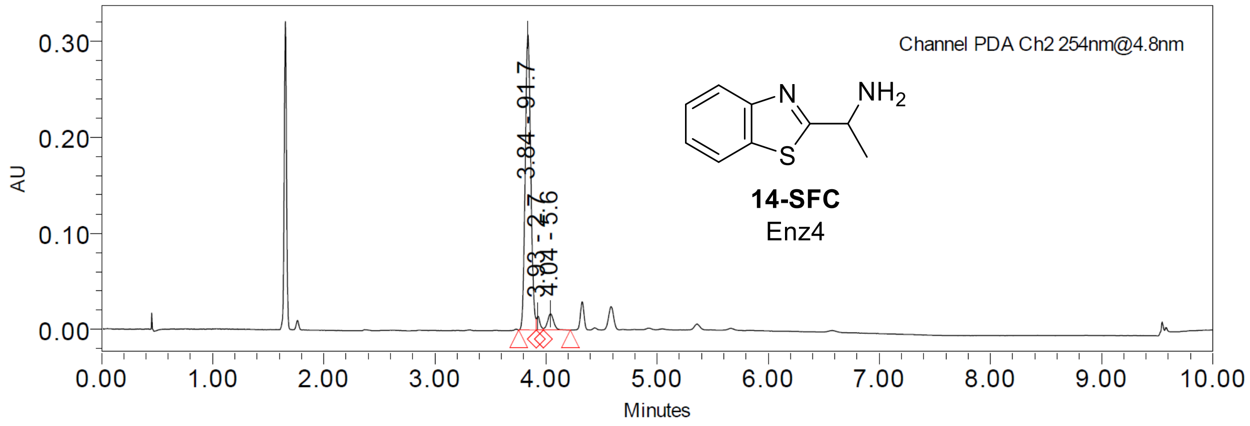
Channel PDA Ch2 254nm@4.8nm
3.84 - 91.7
3.93 - 2.7
4.04 - 5.6
N
NH2
S
14-SFC
Enz4
AU
Minutes

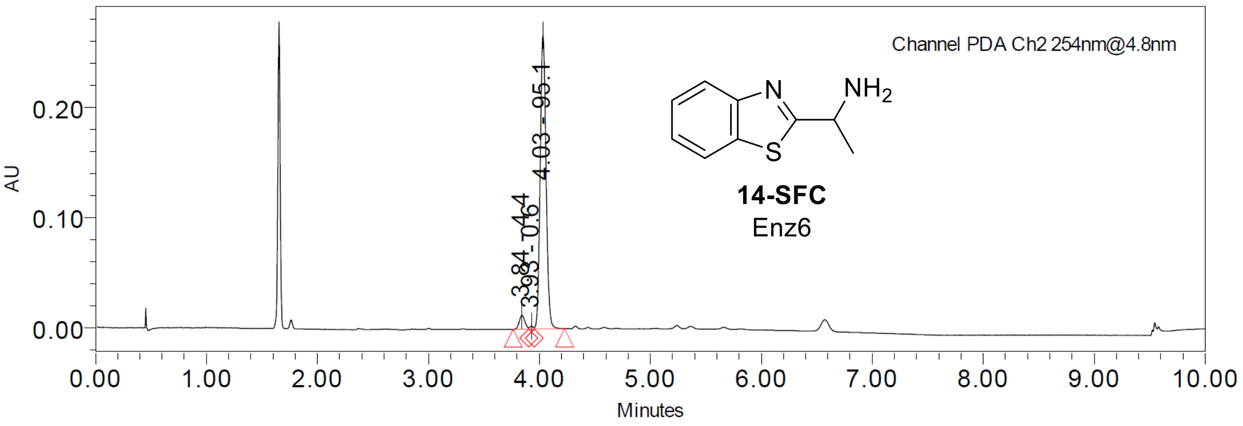
Channel PDA Ch2 254nm@4.8nm
NH2
N
S
14-SFC
Enz6
3.84 - 4.4
3.93 - 0.6
4.03 - 95.1
AU
Minutes

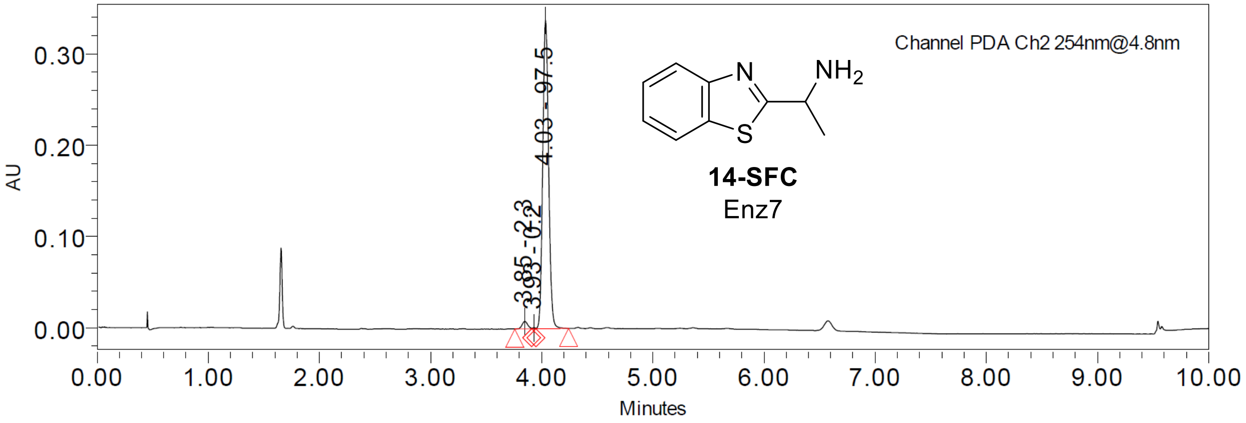
Channel PDA Ch2 254nm@4.8nm
NH2
N
S
14-SFC
Enz7
3.85 - 2.3
3.93 - 0.2
4.03 - 97.5
AU
Minutes

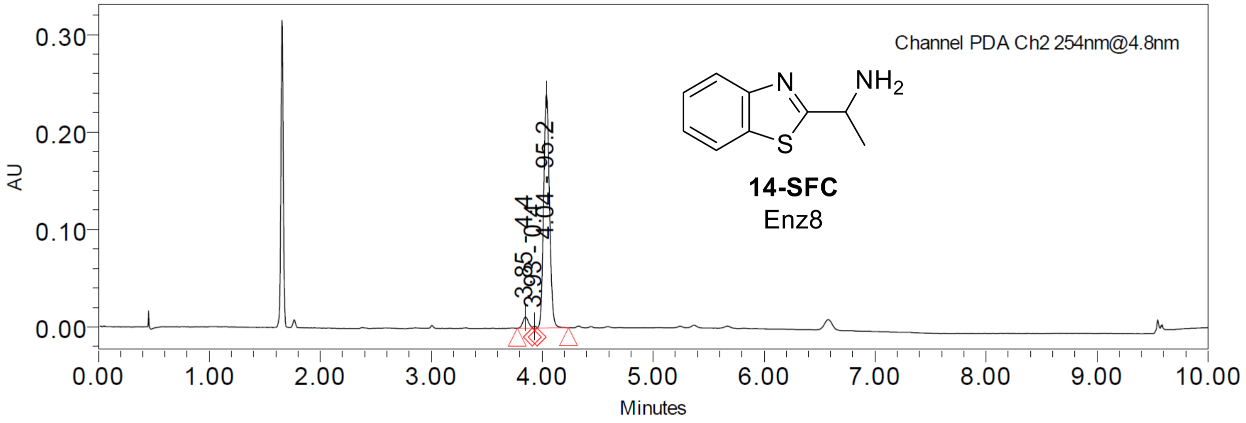
Channel PDA Ch2 254nm@4.8nm
NH2
N
S
14-SFC
Enz8
3.85 - 4.4
3.93 - 0.4
4.04 - 95.2
AU
Minutes

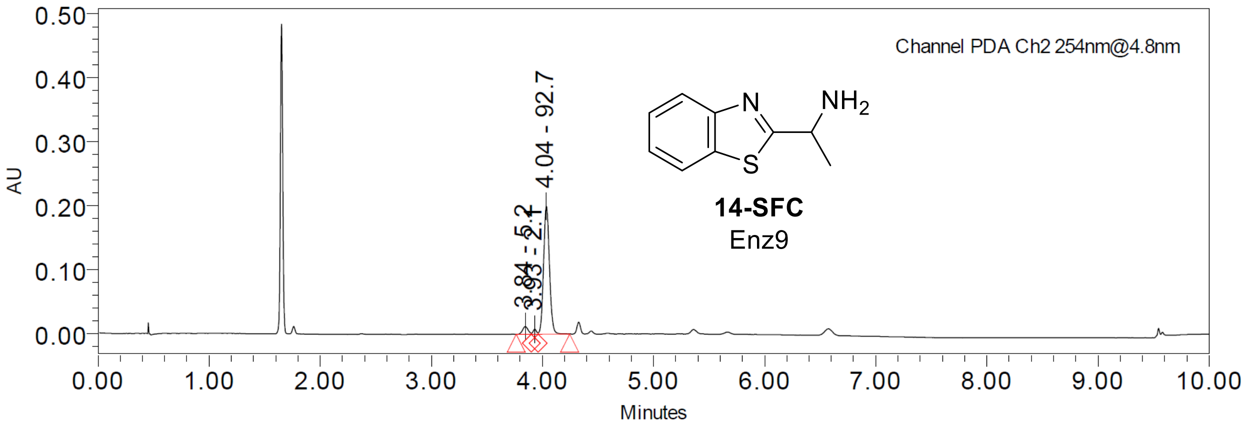
Channel PDA Ch2 254nm@4.8nm
14-SFC
Enz9
AU
Minutes
4.04 - 92.7

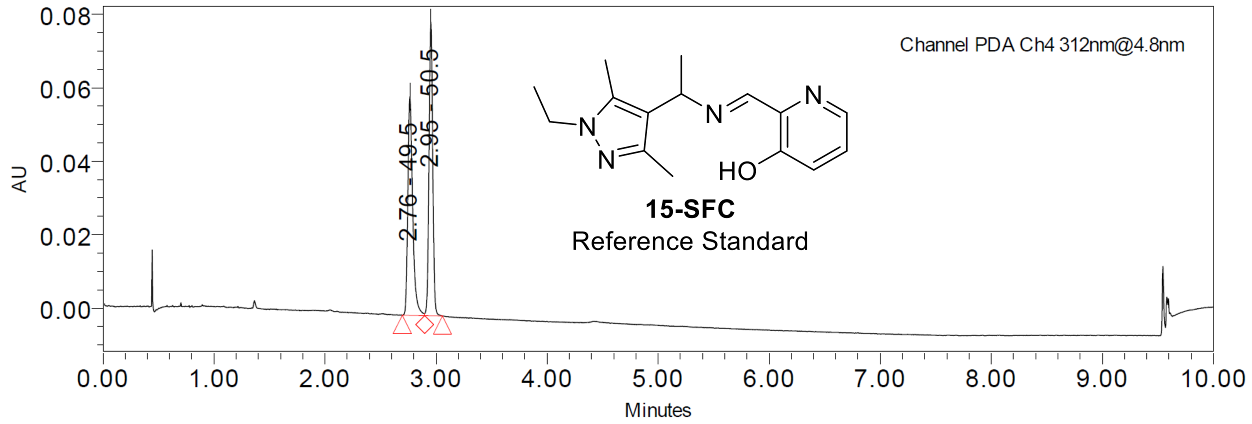
Channel PDA Ch4 312nm@4.8nm
15-SFC
Reference Standard
AU
Minutes
2.76 - 49.5
2.95 - 50.5

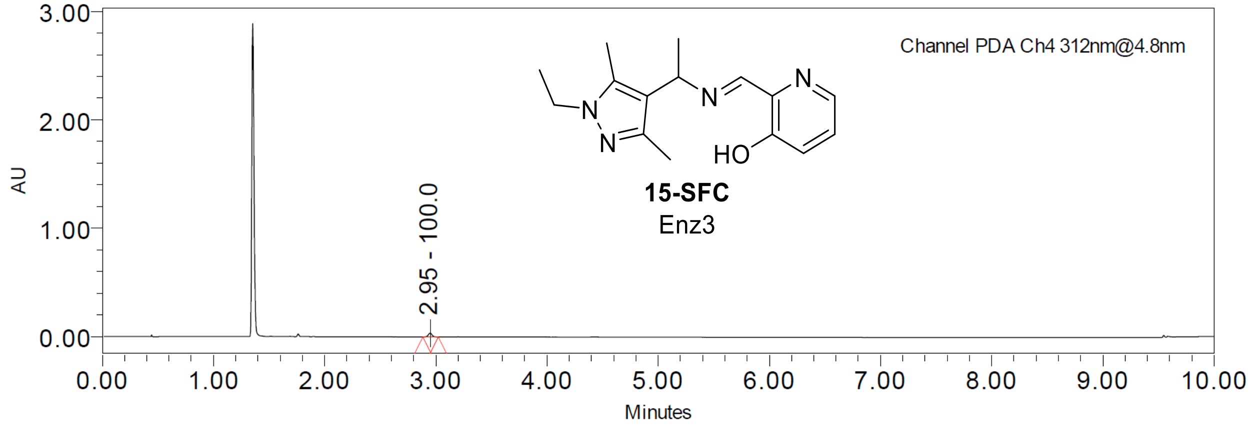
Channel PDA Ch4 312nm@4.8nm
15-SFC
Enz3
AU
Minutes
2.95 - 100.0

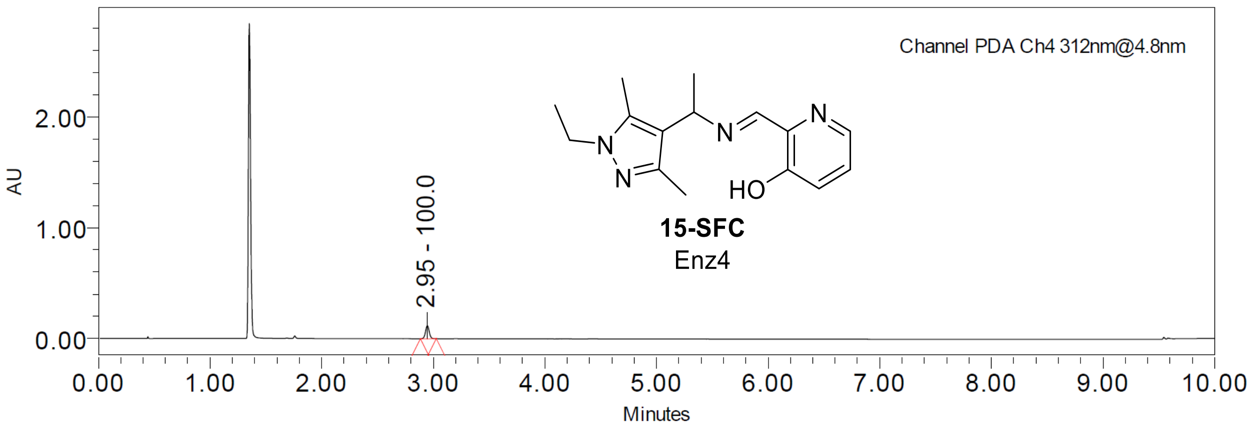

Channel PDA Ch4 312nm@4.8nm
15-SFC
Enz4
2.95 - 100.0
AU
Minutes

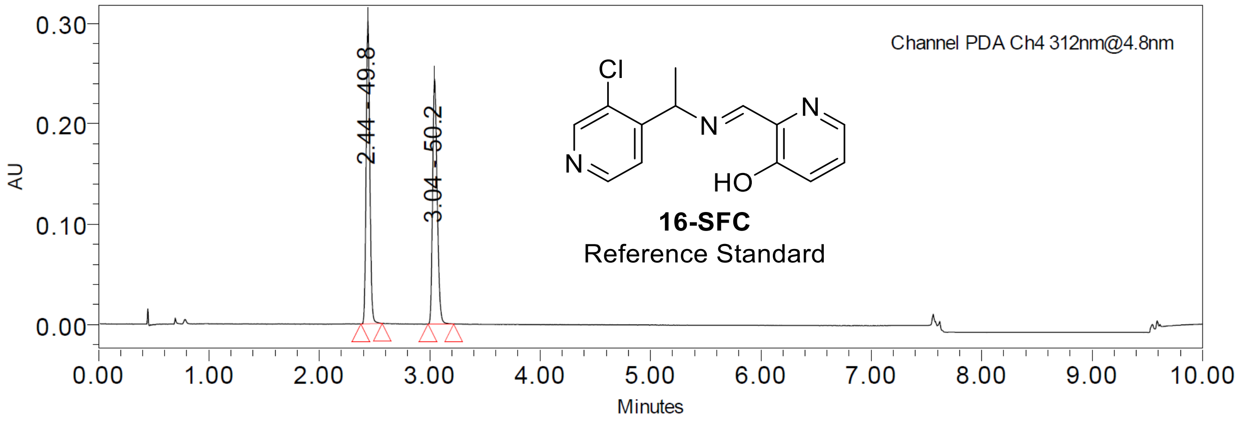

Channel PDA Ch4 312nm@4.8nm
16-SFC
Reference Standard
2.44 - 49.8
3.04 - 50.2
AU
Minutes

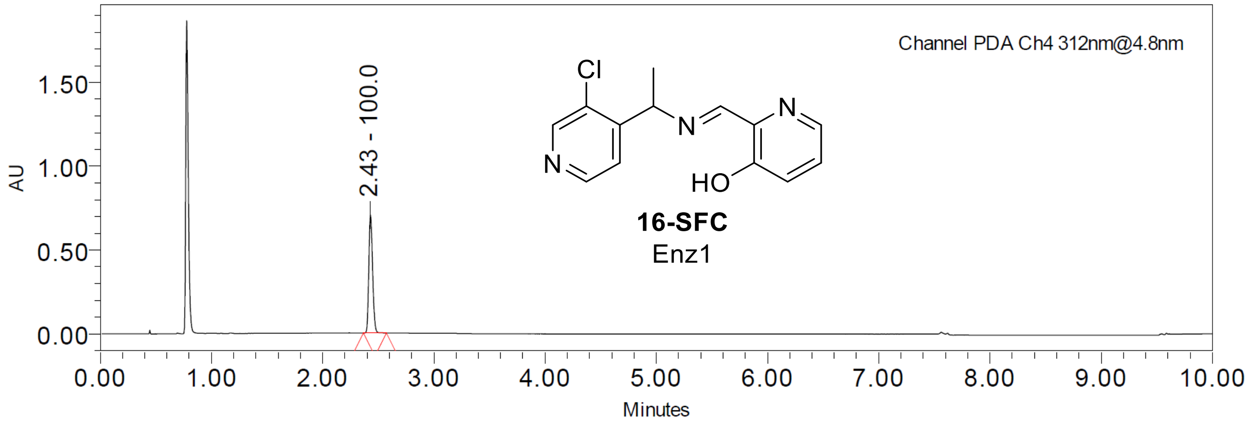

Channel PDA Ch4 312nm@4.8nm
16-SFC
Enz1
2.43 - 100.0
AU
Minutes

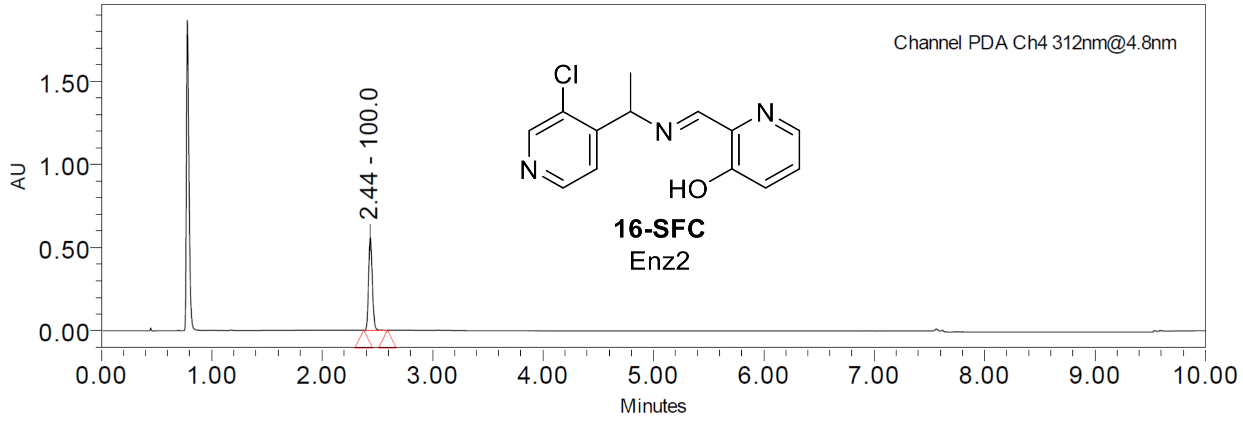
Channel PDA Ch4 312nm@4.8nm
2.44 - 100.0
Cl
N
N
N
HO
16-SFC
Enz2
AU
1.50
1.00
0.50
0.00
0.00
1.00
2.00
3.00
4.00
5.00
6.00
7.00
8.00
9.00
10.00
Minutes

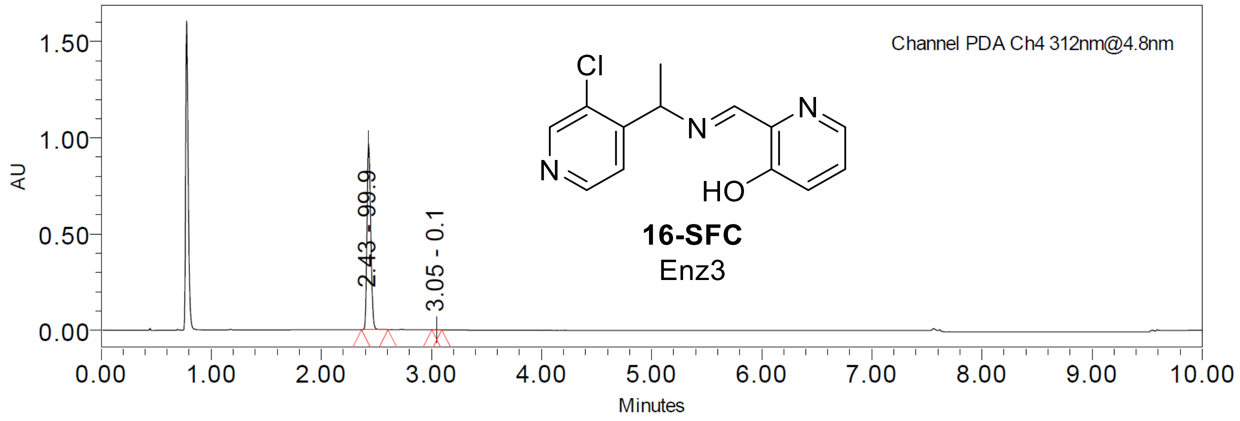
Channel PDA Ch4 312nm@4.8nm
2.43 - 99.9
3.05 - 0.1
Cl
N
N
N
HO
16-SFC
Enz3
AU
1.50
1.00
0.50
0.00
0.00
1.00
2.00
3.00
4.00
5.00
6.00
7.00
8.00
9.00
10.00
Minutes

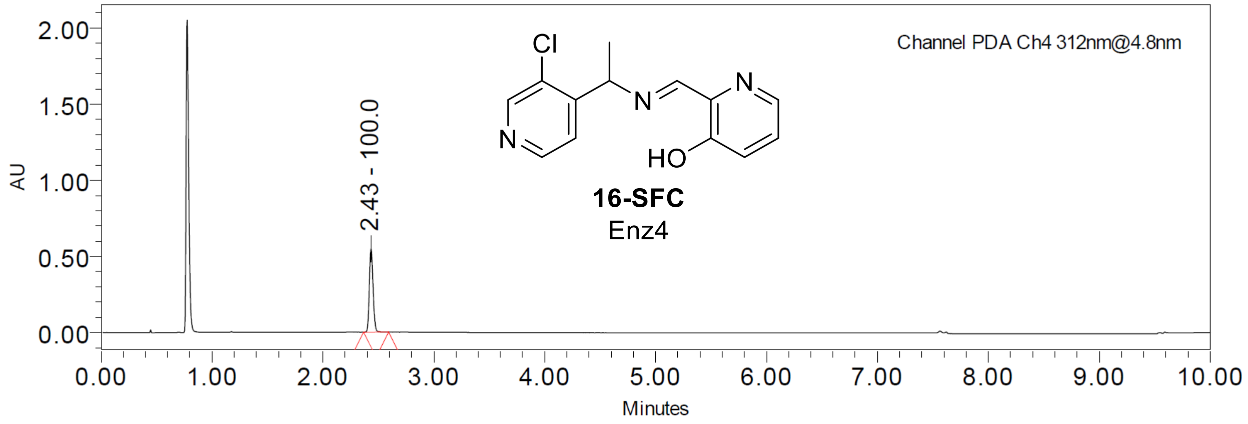
Channel PDA Ch4 312nm@4.8nm
2.43 - 100.0
Cl
N
N
N
HO
16-SFC
Enz4
AU
2.00
1.50
1.00
0.50
0.00
0.00
1.00
2.00
3.00
4.00
5.00
6.00
7.00
8.00
9.00
10.00
Minutes

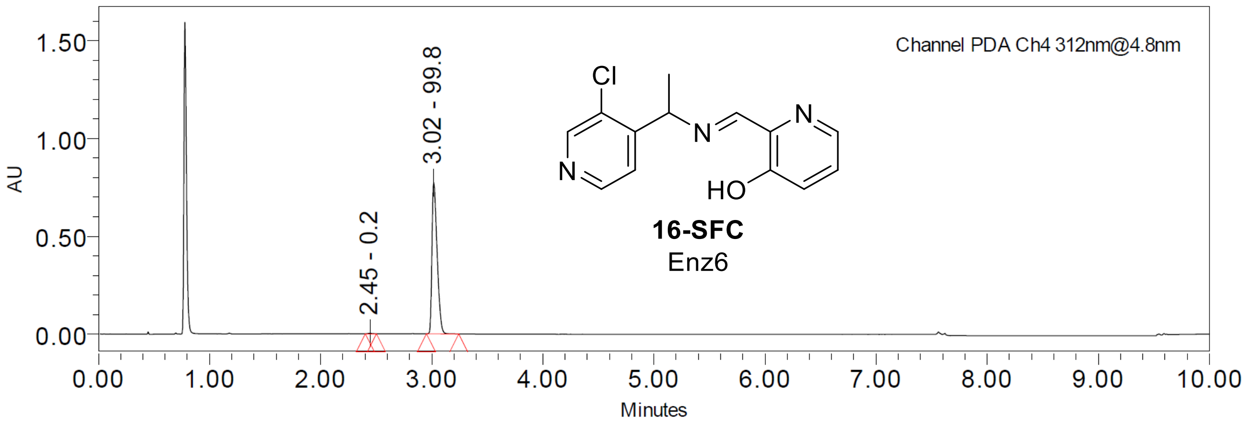

Channel PDA Ch4 312nm@4.8nm
AU
Minutes
2.45 - 0.2
3.02 - 99.8
Cl
N
N
N
HO
16-SFC
Enz6

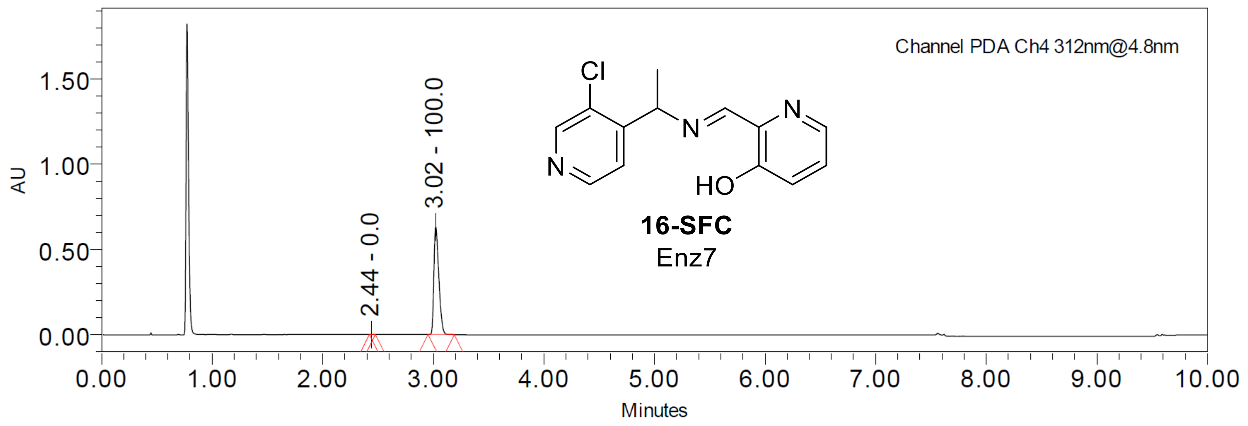

Channel PDA Ch4 312nm@4.8nm
AU
Minutes
2.44 - 0.0
3.02 - 100.0
Cl
N
N
N
HO
16-SFC
Enz7

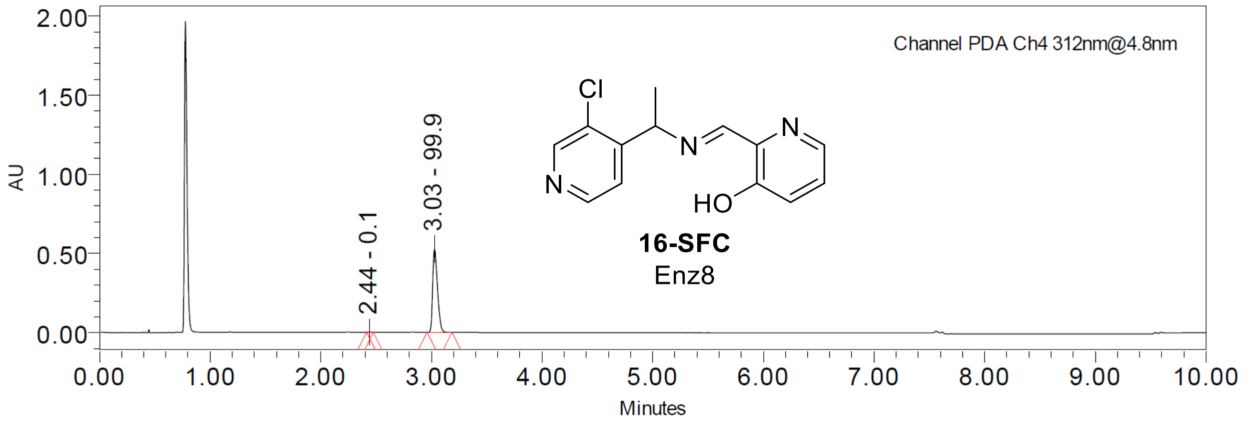

Channel PDA Ch4 312nm@4.8nm
AU
Minutes
2.44 - 0.1
3.03 - 99.9
Cl
N
N
N
HO
16-SFC
Enz8

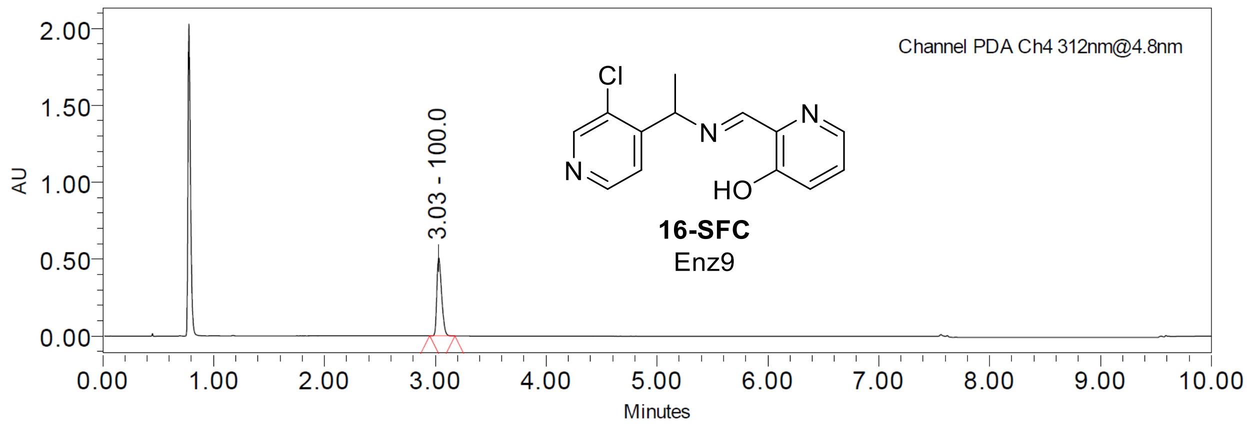

Channel PDA Ch4 312nm@4.8nm
AU
2.00
1.50
1.00
0.50
0.00
3.03 - 100.0
Cl
N
N
N
HO
16-SFC
Enz9
0.00
1.00
2.00
3.00
4.00
5.00
6.00
7.00
8.00
9.00
10.00
Minutes

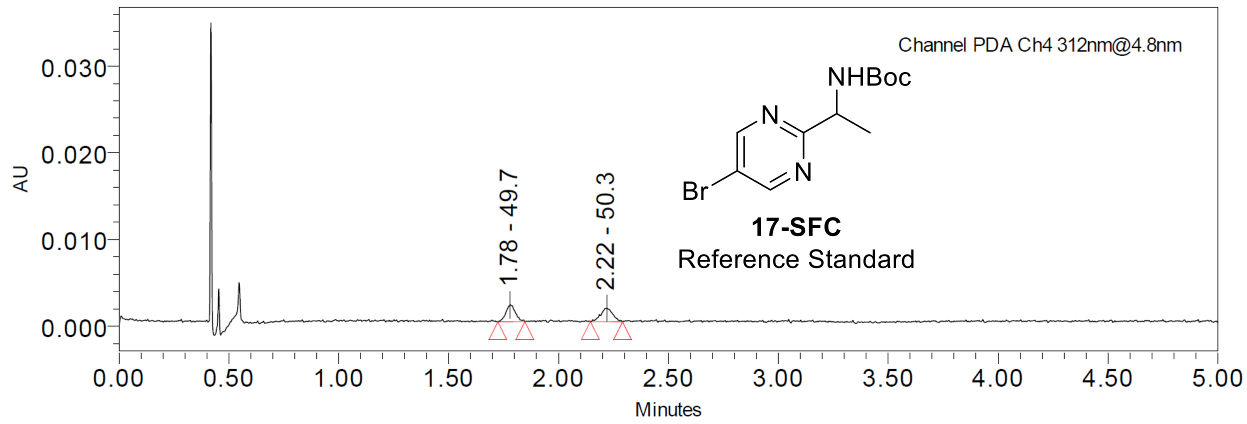

Channel PDA Ch4 312nm@4.8nm
AU
0.030
0.020
0.010
0.000
1.78 - 49.7
2.22 - 50.3
NHBoc
N
N
Br
17-SFC
Reference Standard
0.00
0.50
1.00
1.50
2.00
2.50
3.00
3.50
4.00
4.50
5.00
Minutes

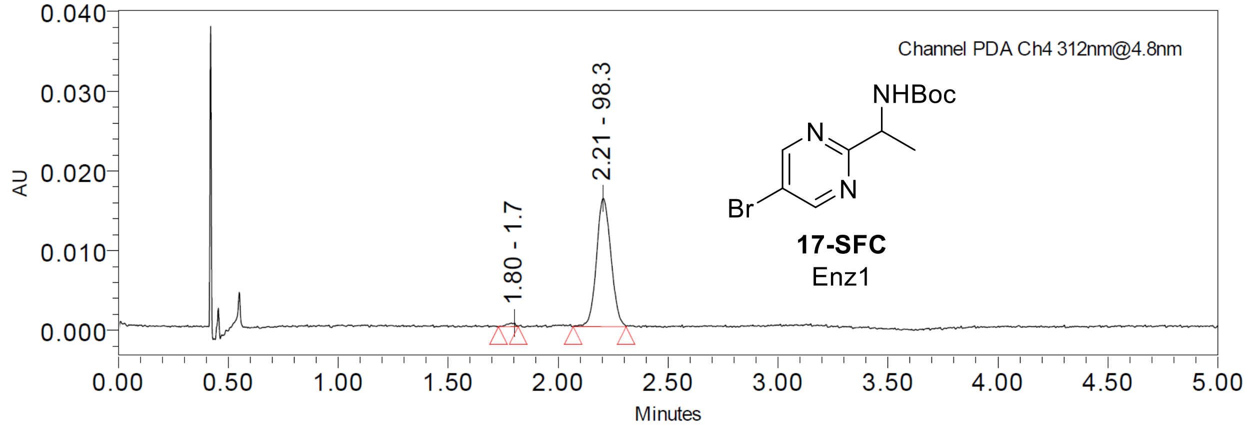

Channel PDA Ch4 312nm@4.8nm
AU
0.040
0.030
0.020
0.010
0.000
1.80 - 1.7
2.21 - 98.3
NHBoc
N
N
Br
17-SFC
Enz1
0.00
0.50
1.00
1.50
2.00
2.50
3.00
3.50
4.00
4.50
5.00
Minutes

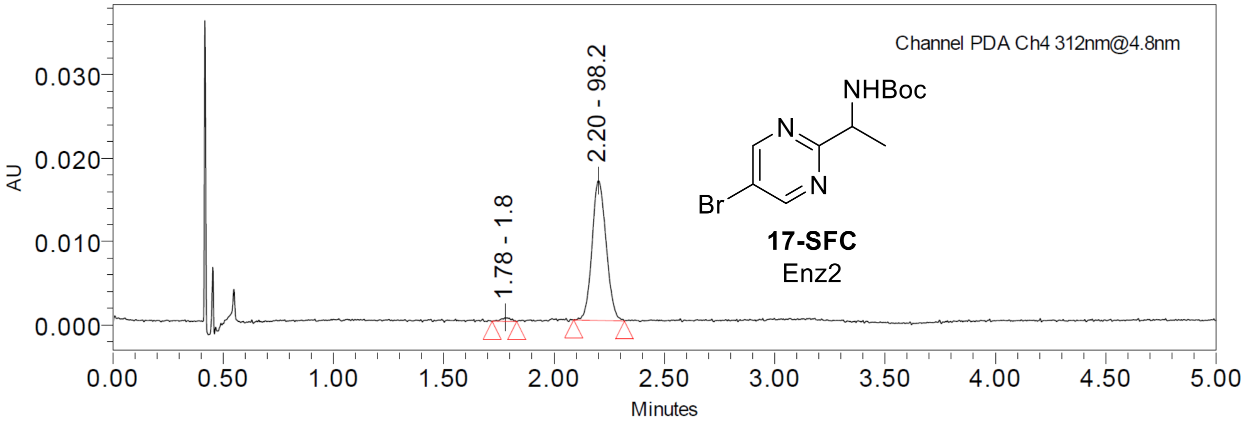
Channel PDA Ch4 312nm@4.8nm
AU
Minutes
1.78 - 1.8
2.20 - 98.2
NHBoc
N
N
Br
17-SFC
Enz2

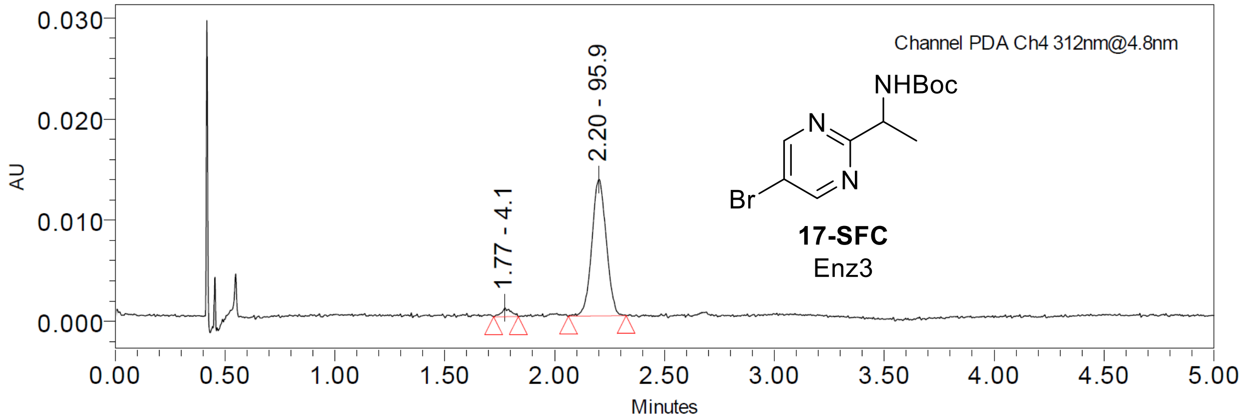
Channel PDA Ch4 312nm@4.8nm
AU
Minutes
1.77 - 4.1
2.20 - 95.9
NHBoc
N
N
Br
17-SFC
Enz3

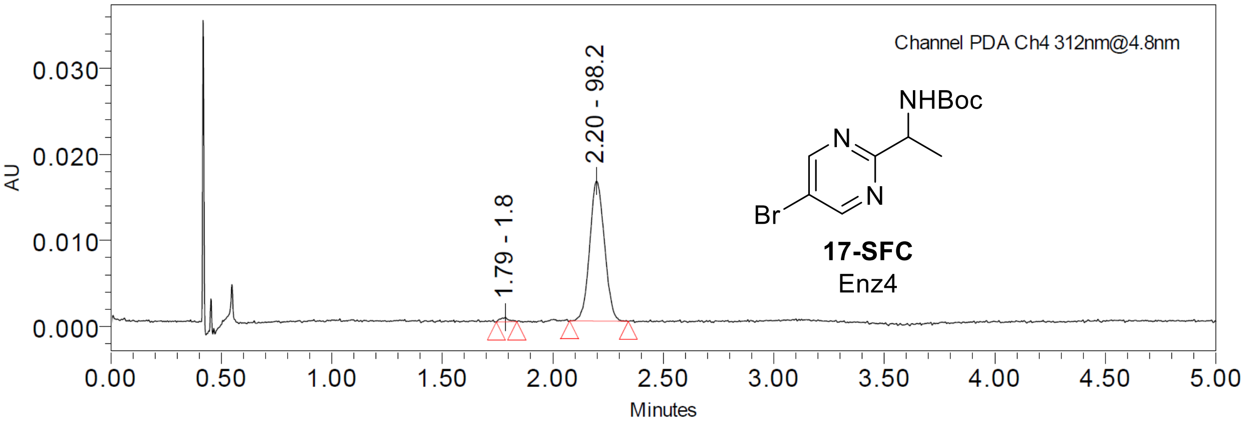
Channel PDA Ch4 312nm@4.8nm
AU
Minutes
1.79 - 1.8
2.20 - 98.2
NHBoc
N
N
Br
17-SFC
Enz4

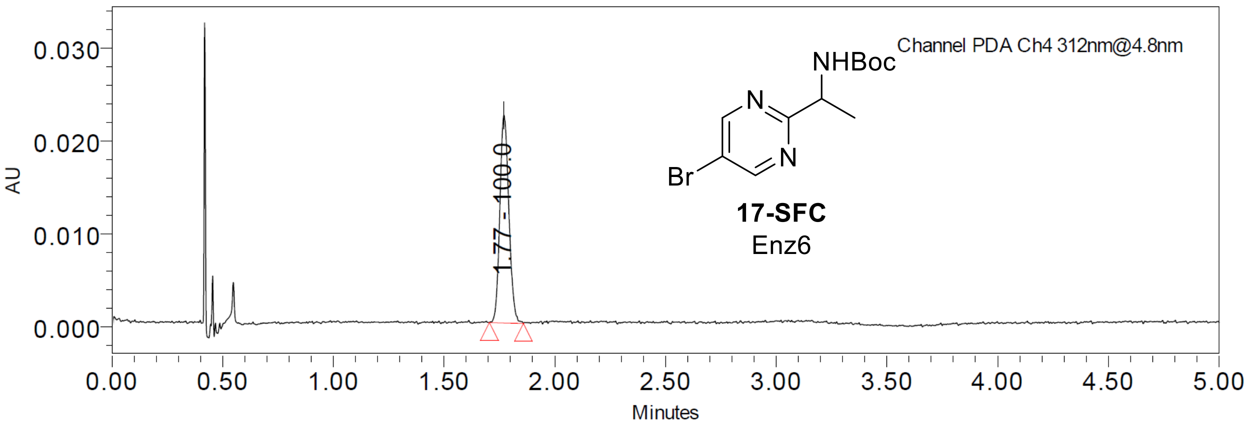

Channel PDA Ch4 312nm@4.8nm
NHBoc
N
N
Br
17-SFC
Enz6
1.77 - 100.0
AU
Minutes

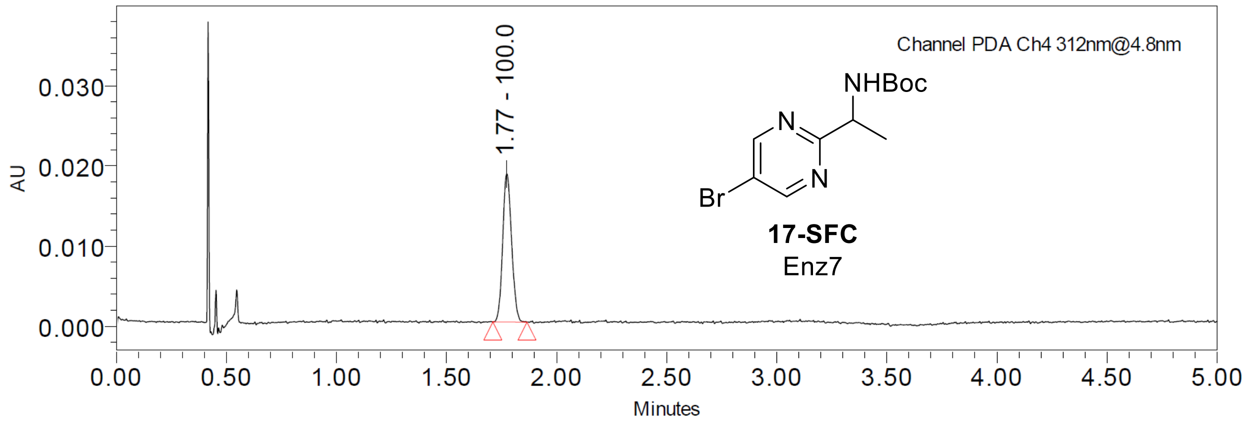

Channel PDA Ch4 312nm@4.8nm
NHBoc
N
N
Br
17-SFC
Enz7
1.77 - 100.0
AU
Minutes

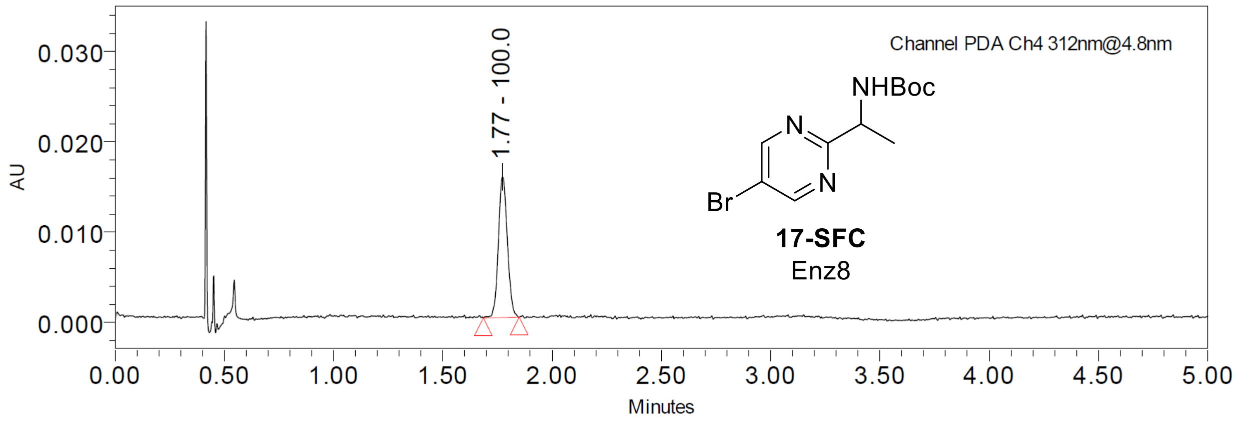

Channel PDA Ch4 312nm@4.8nm
NHBoc
N
N
Br
17-SFC
Enz8
1.77 - 100.0
AU
Minutes

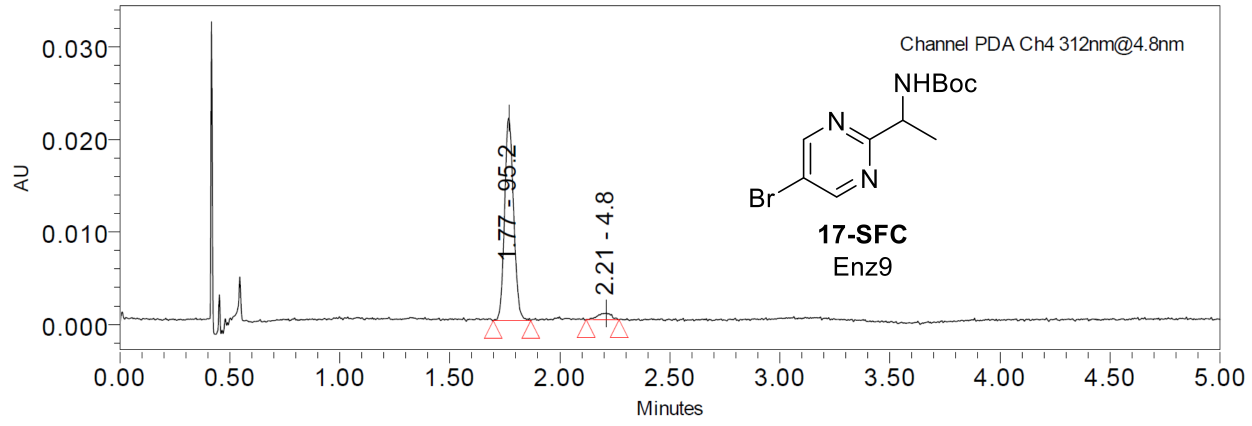

Channel PDA Ch4 312nm@4.8nm
NHBoc
N
N
Br
17-SFC
Enz9
1.77 - 95.2
2.21 - 4.8
AU
Minutes

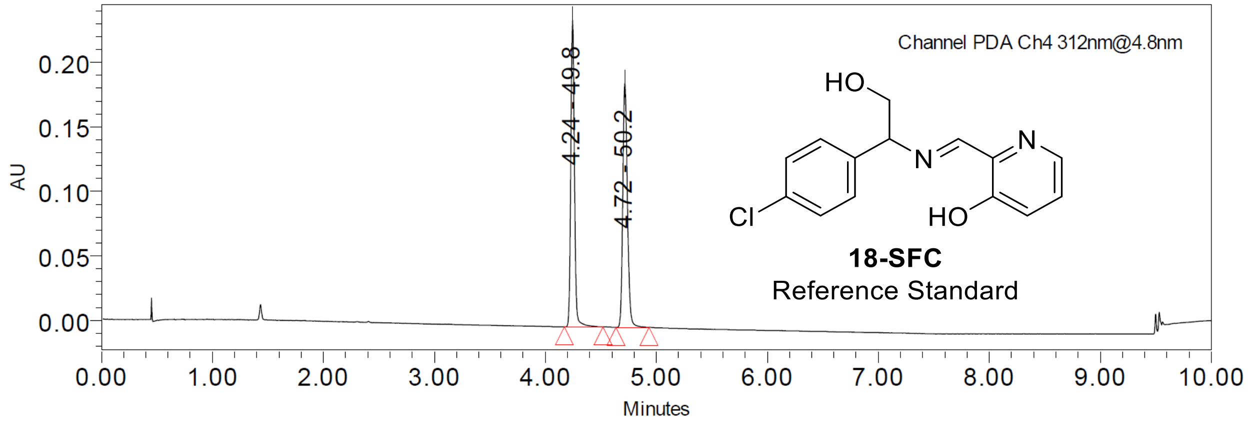

Channel PDA Ch4 312nm@4.8nm
HO
N
N
Cl
HO
18-SFC
Reference Standard
4.24 - 49.8
4.72 - 50.2
AU
Minutes

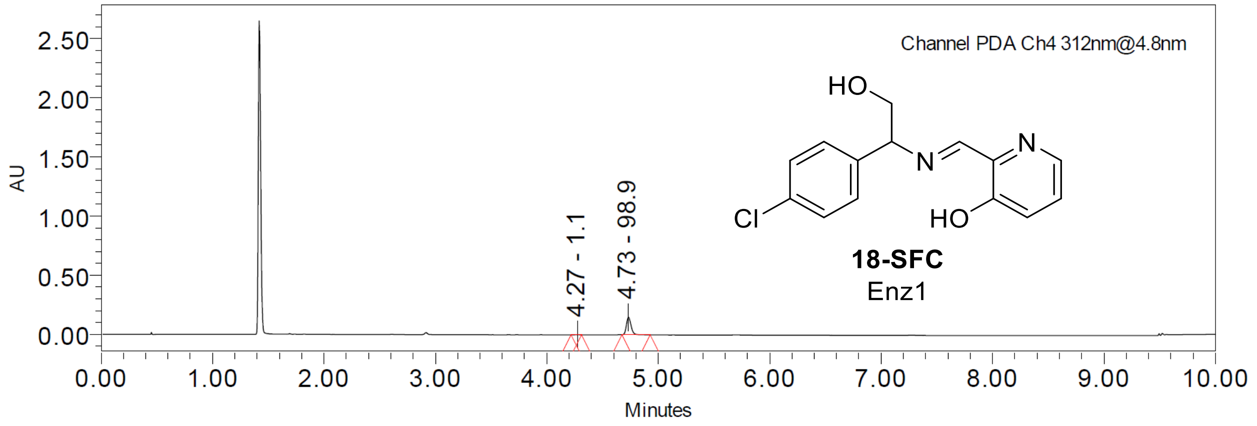

Channel PDA Ch4 312nm@4.8nm
HO
N
N
Cl
HO
18-SFC
Enz1
4.27 - 1.1
4.73 - 98.9
AU
Minutes

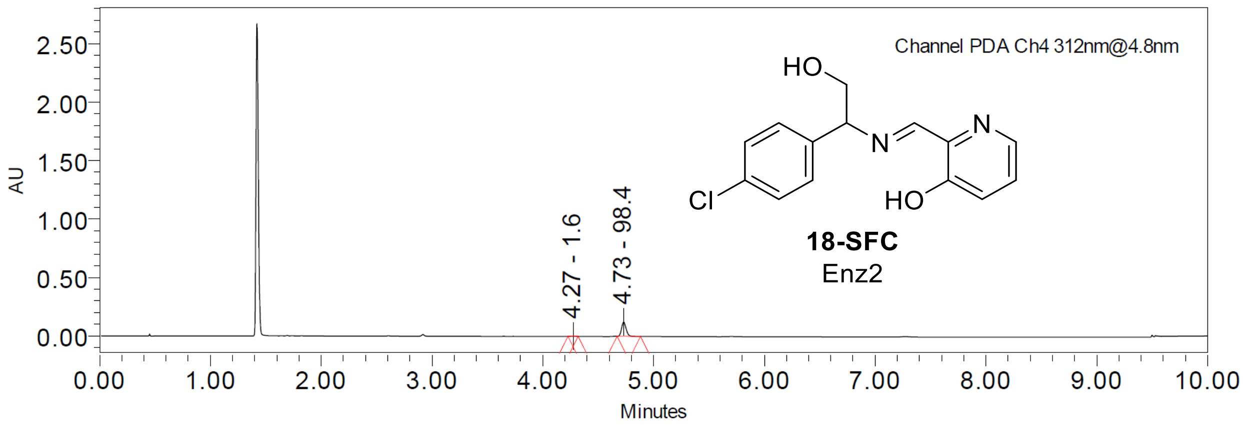

Channel PDA Ch4 312nm@4.8nm
HO
N
N
Cl
HO
18-SFC
Enz2
4.27 - 1.6
4.73 - 98.4
AU
Minutes

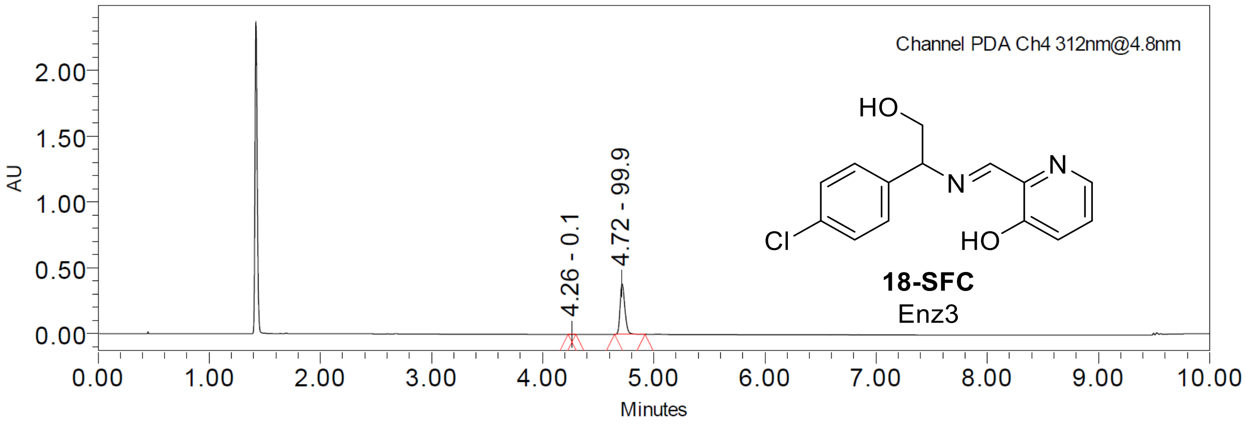

Channel PDA Ch4 312nm@4.8nm
HO
N
N
Cl
HO
18-SFC
Enz3
4.26 - 0.1
4.72 - 99.9
AU
Minutes

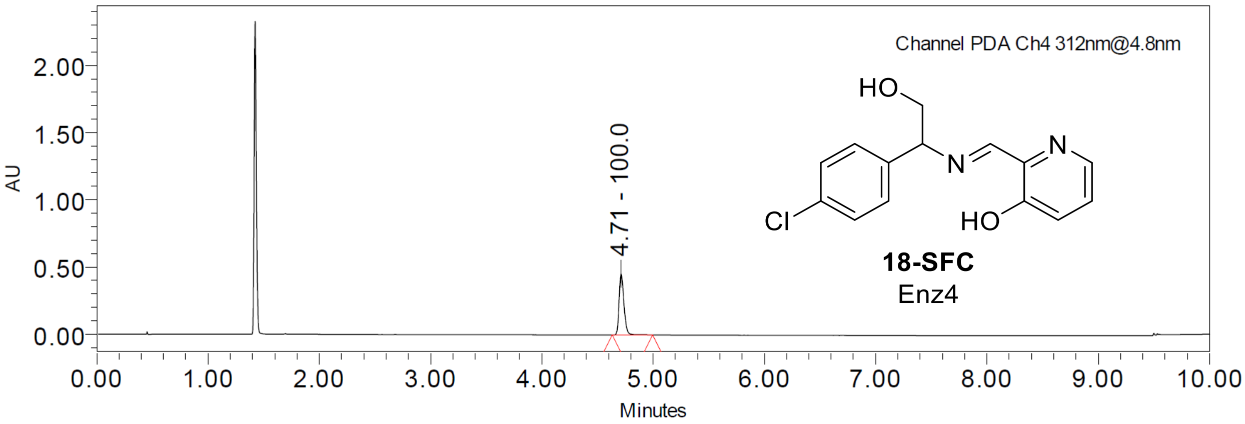

Channel PDA Ch4 312nm@4.8nm
HO
N
N
Cl
HO
18-SFC
Enz4
4.71 - 100.0
AU
Minutes

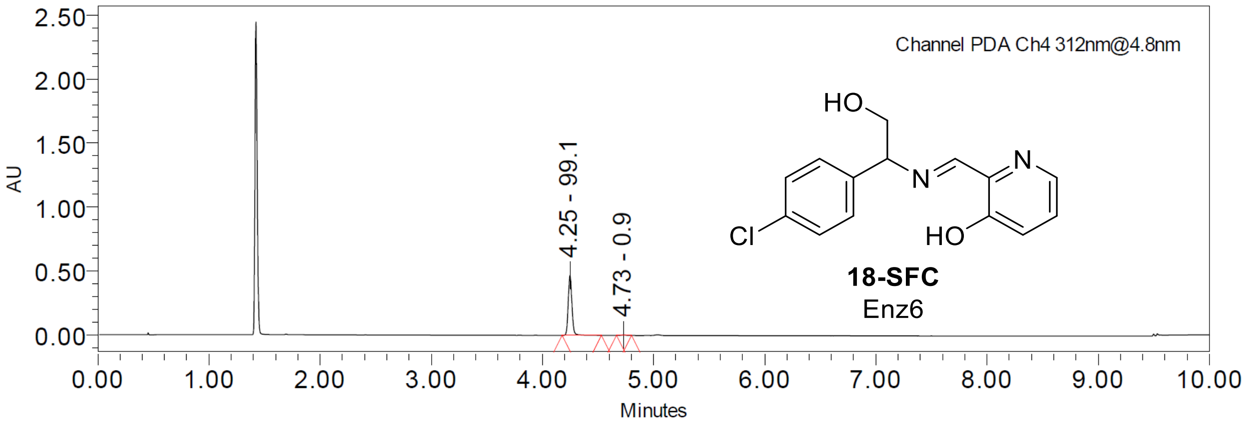
Channel PDA Ch4 312nm@4.8nm
4.25 - 99.1
4.73 - 0.9
18-SFC
Enz6
AU
Minutes

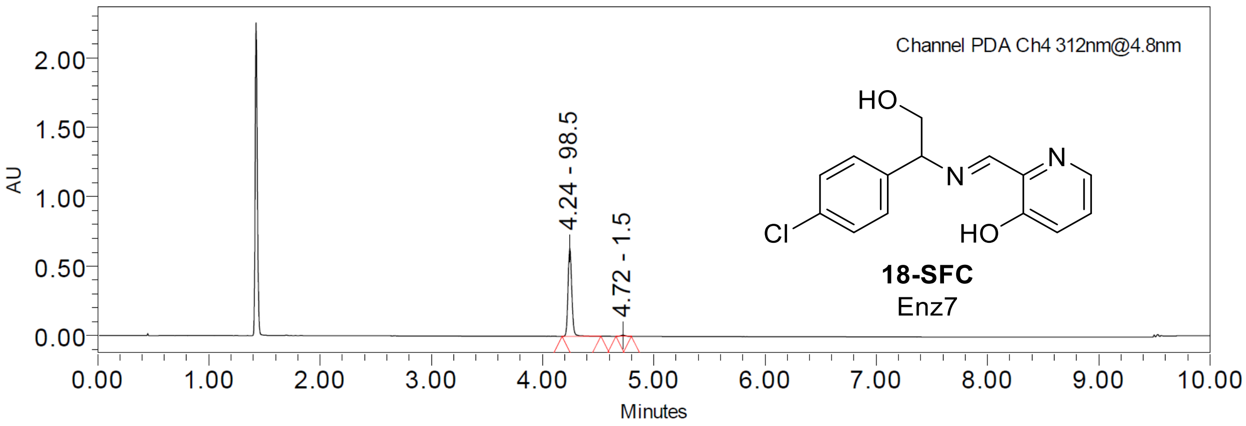
Channel PDA Ch4 312nm@4.8nm
4.24 - 98.5
4.72 - 1.5
18-SFC
Enz7
AU
Minutes

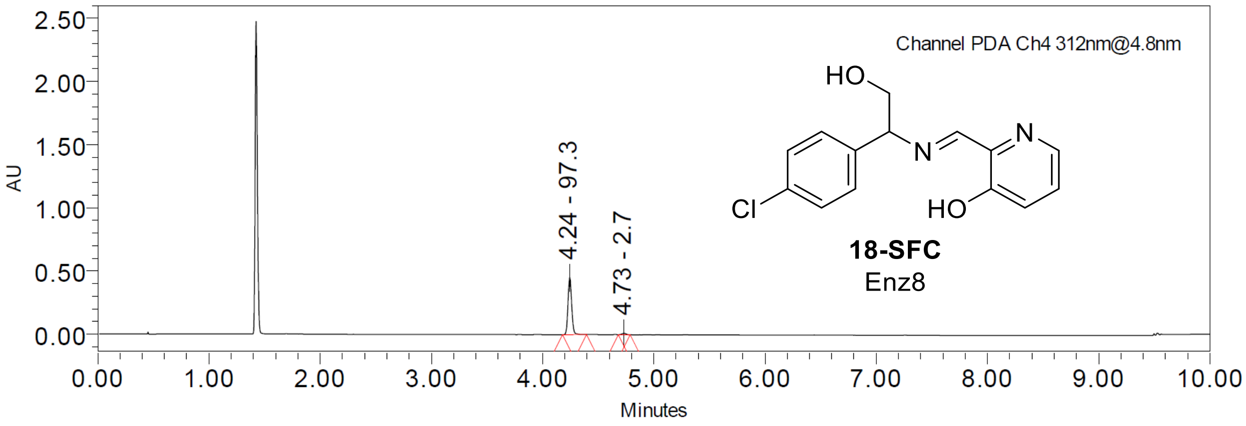
Channel PDA Ch4 312nm@4.8nm
4.24 - 97.3
4.73 - 2.7
18-SFC
Enz8
AU
Minutes

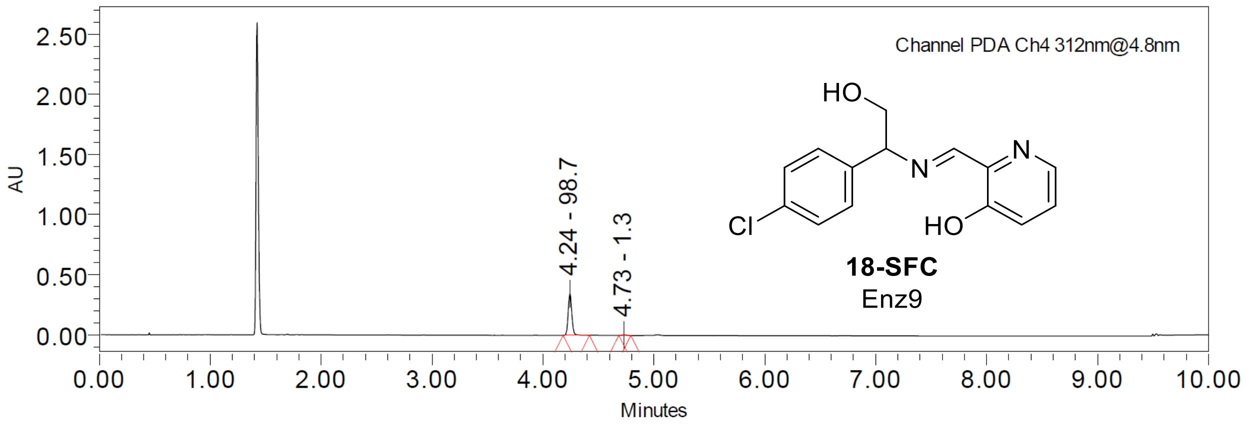

Channel PDA Ch4 312nm@4.8nm
4.24 - 98.7
4.73 - 1.3
HO
N
N
Cl
HO
18-SFC
Enz9
AU
2.50
2.00
1.50
1.00
0.50
0.00
0.00
1.00
2.00
3.00
4.00
5.00
6.00
7.00
8.00
9.00
10.00
Minutes

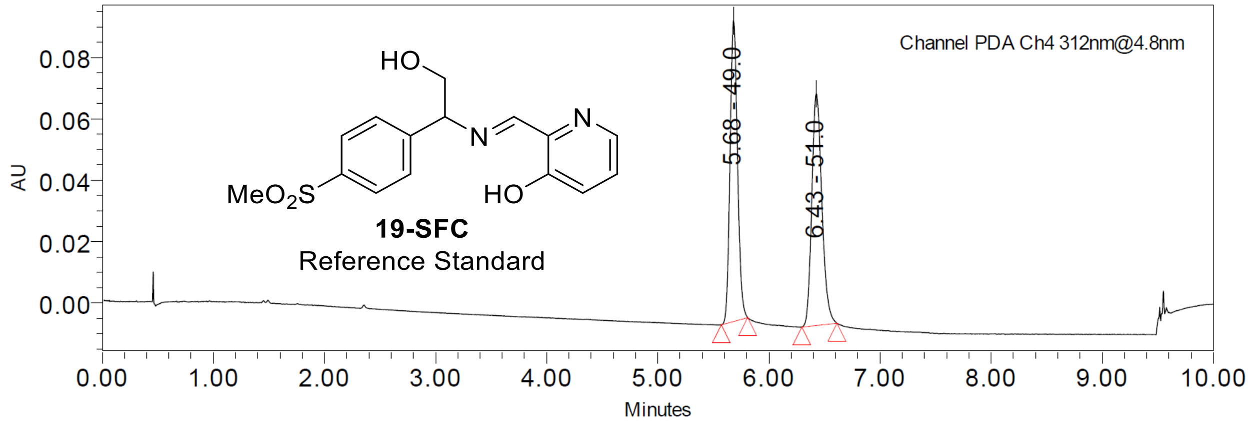

Channel PDA Ch4 312nm@4.8nm
HO
N
N
MeO2S
HO
19-SFC
Reference Standard
5.68 - 49.0
6.43 - 51.0
AU
0.08
0.06
0.04
0.02
0.00
0.00
1.00
2.00
3.00
4.00
5.00
6.00
7.00
8.00
9.00
10.00
Minutes

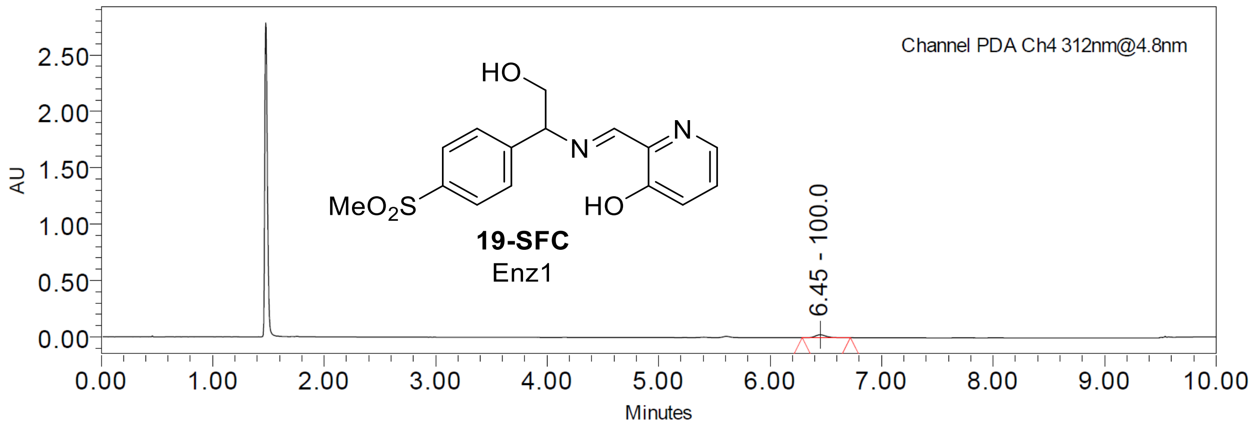

Channel PDA Ch4 312nm@4.8nm
HO
N
N
MeO2S
HO
19-SFC
Enz1
6.45 - 100.0
AU
2.50
2.00
1.50
1.00
0.50
0.00
0.00
1.00
2.00
3.00
4.00
5.00
6.00
7.00
8.00
9.00
10.00
Minutes

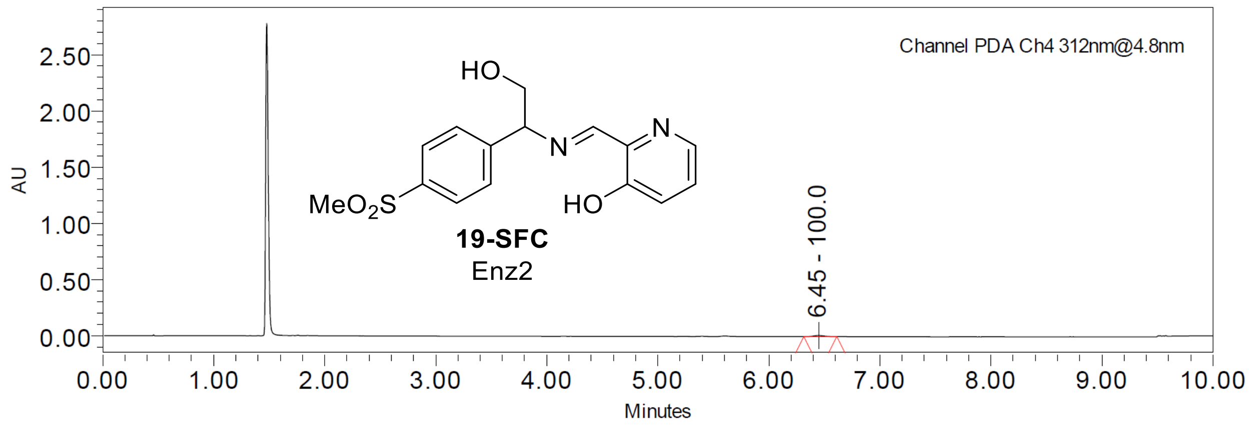
Channel PDA Ch4 312nm@4.8nm
HO
N
N
MeO2S
HO
19-SFC
Enz2
6.45 - 100.0
AU
2.50
2.00
1.50
1.00
0.50
0.00
0.00
1.00
2.00
3.00
4.00
5.00
6.00
7.00
8.00
9.00
10.00
Minutes

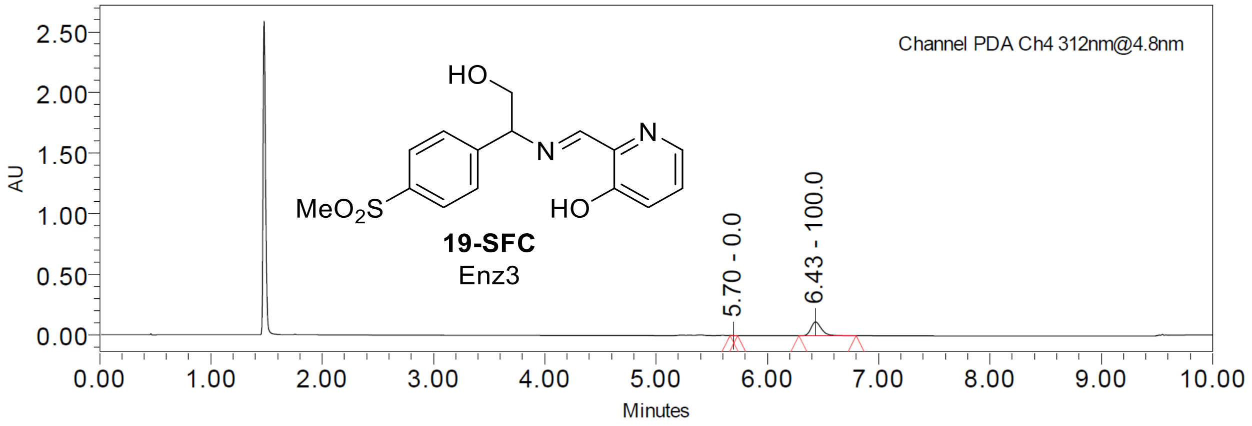
Channel PDA Ch4 312nm@4.8nm
HO
N
N
MeO2S
HO
19-SFC
Enz3
5.70 - 0.0
6.43 - 100.0
AU
2.50
2.00
1.50
1.00
0.50
0.00
0.00
1.00
2.00
3.00
4.00
5.00
6.00
7.00
8.00
9.00
10.00
Minutes

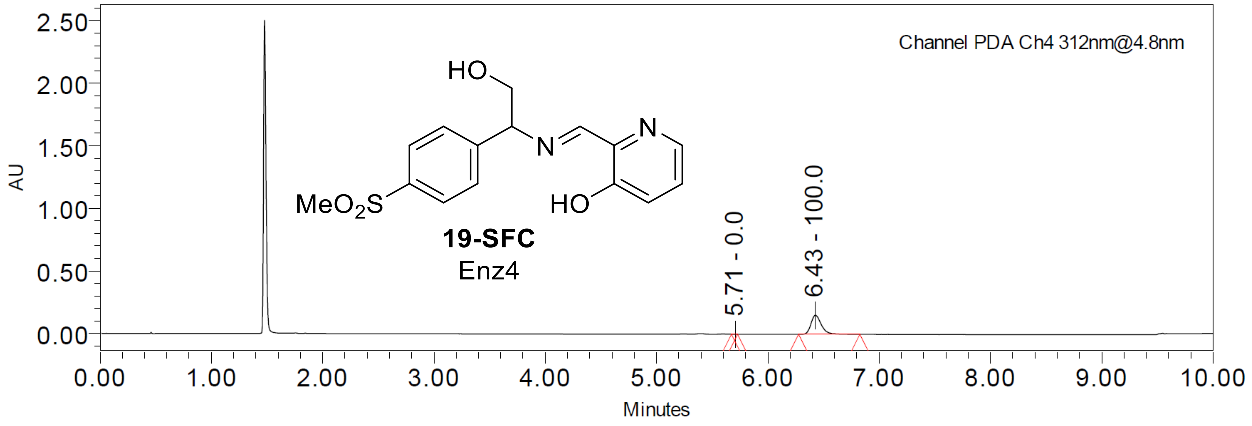
Channel PDA Ch4 312nm@4.8nm
HO
N
N
MeO2S
HO
19-SFC
Enz4
5.71 - 0.0
6.43 - 100.0
AU
2.50
2.00
1.50
1.00
0.50
0.00
0.00
1.00
2.00
3.00
4.00
5.00
6.00
7.00
8.00
9.00
10.00
Minutes

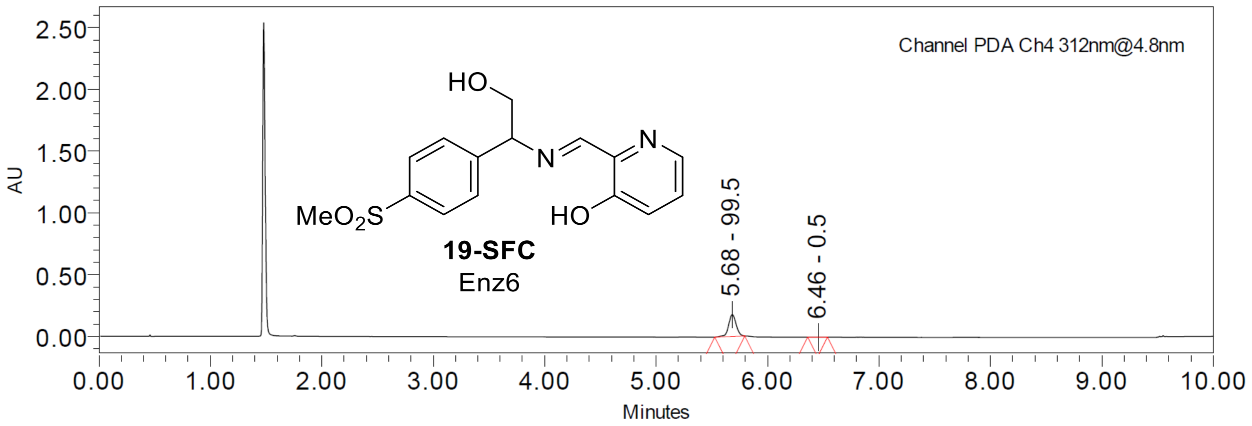
Channel PDA Ch4 312nm@4.8nm
AU
Minutes
19-SFC
Enz6
5.68 - 99.5
6.46 - 0.5

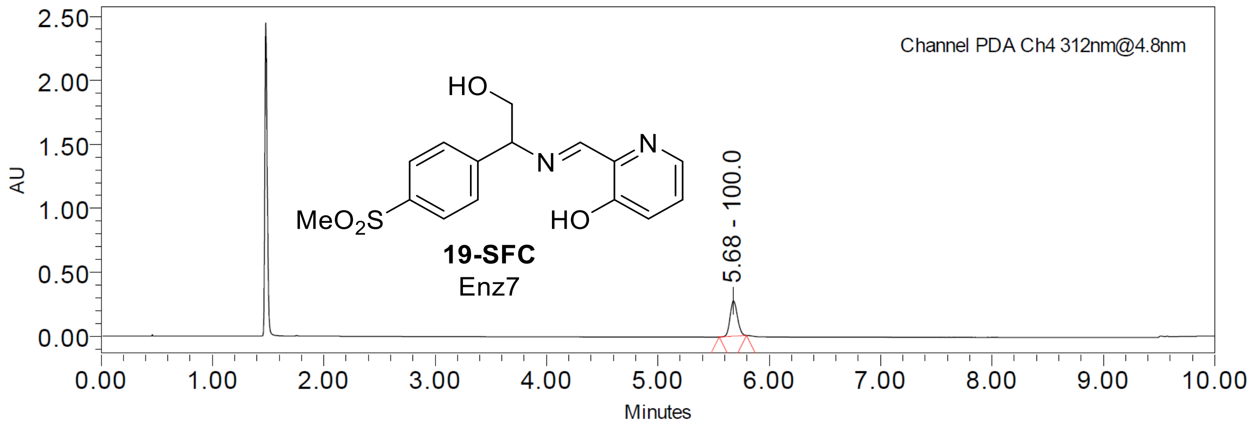
Channel PDA Ch4 312nm@4.8nm
AU
Minutes
19-SFC
Enz7
5.68 - 100.0

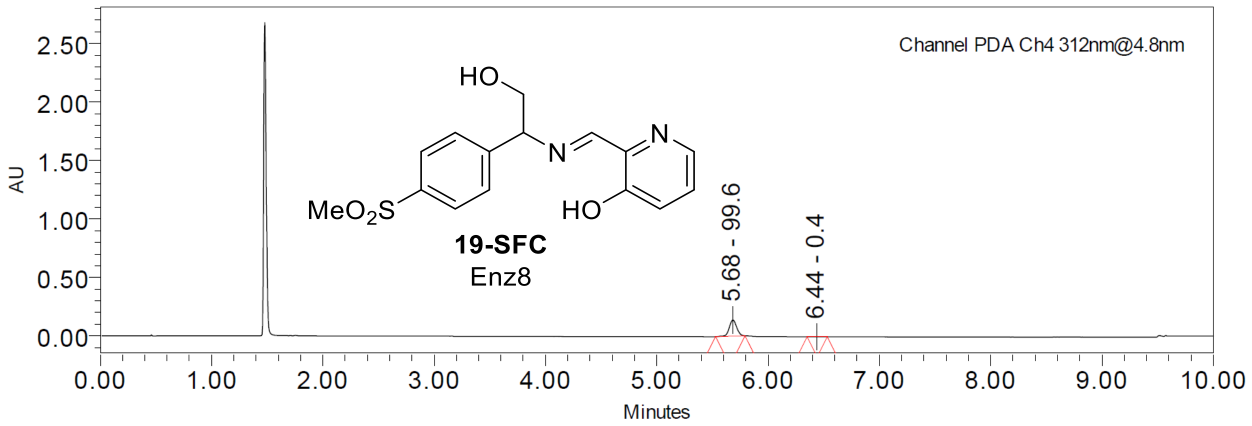
Channel PDA Ch4 312nm@4.8nm
AU
Minutes
19-SFC
Enz8
5.68 - 99.6
6.44 - 0.4

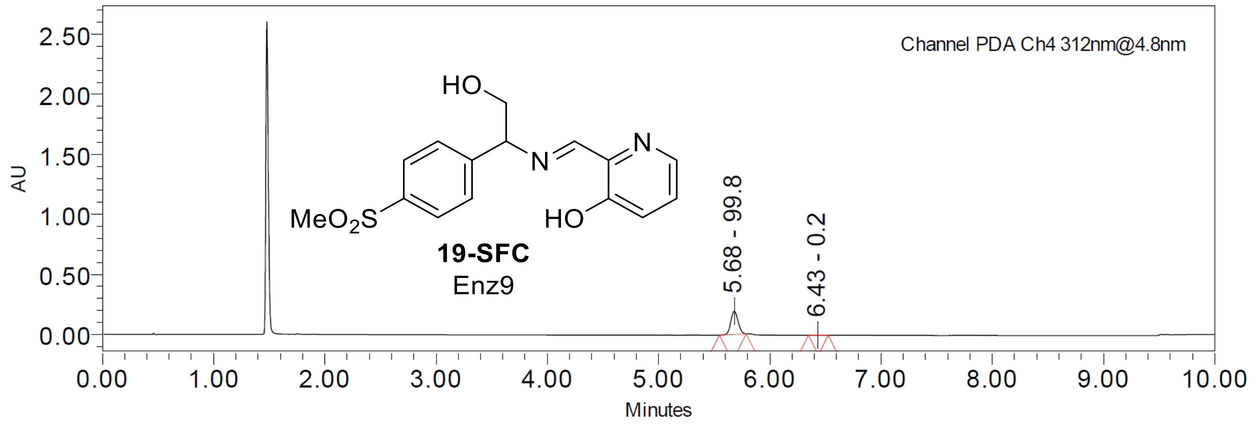
Channel PDA Ch4 312nm@4.8nm
HO
N
N
MeO2S
HO
19-SFC
Enz9
5.68 - 99.8
6.43 - 0.2
AU
Minutes

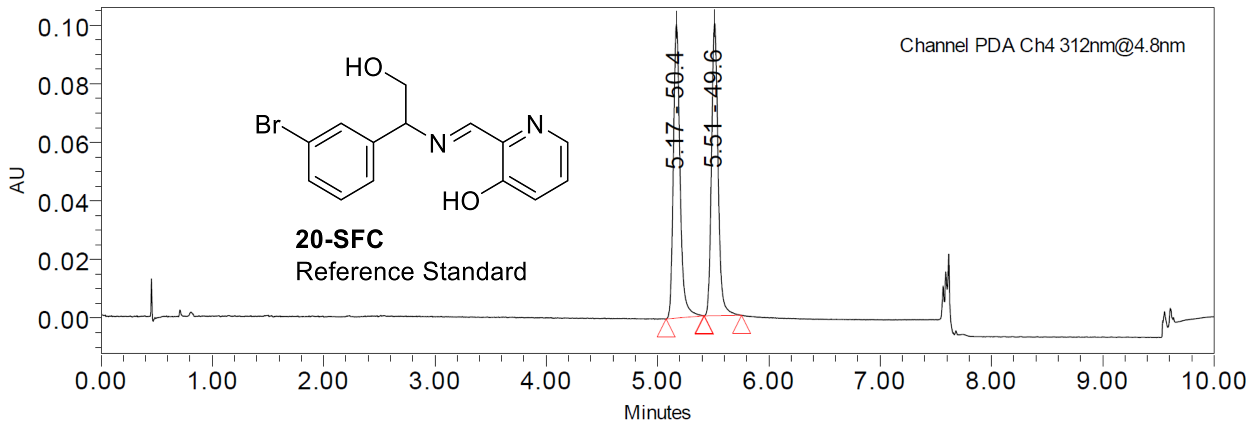
Channel PDA Ch4 312nm@4.8nm
HO
Br
N
N
HO
20-SFC
Reference Standard
5.17 - 50.4
5.51 - 49.6
AU
Minutes

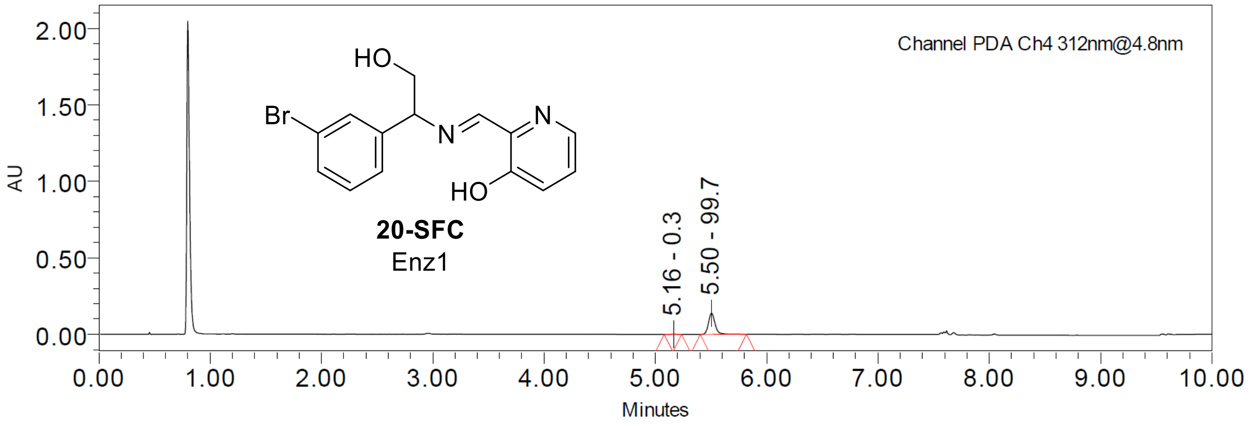
Channel PDA Ch4 312nm@4.8nm
HO
Br
N
N
HO
20-SFC
Enz1
5.16 - 0.3
5.50 - 99.7
AU
Minutes

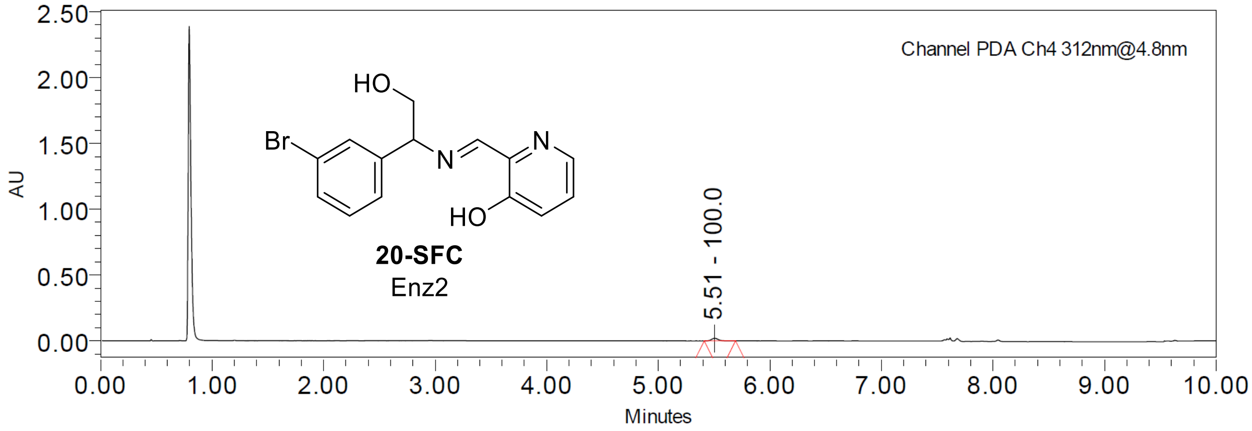

Channel PDA Ch4 312nm@4.8nm
20-SFC
Enz2
5.51 - 100.0
AU
Minutes

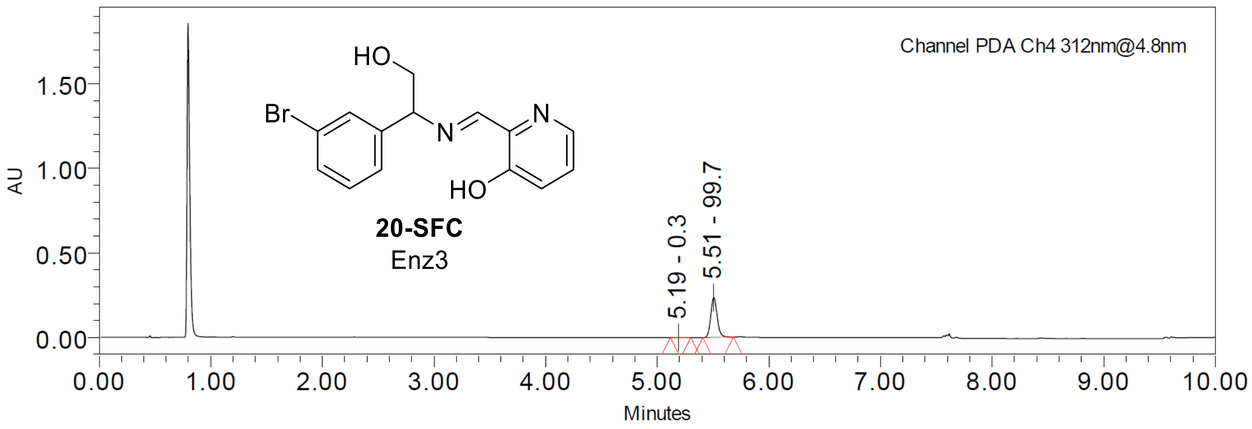

Channel PDA Ch4 312nm@4.8nm
20-SFC
Enz3
5.19 - 0.3
5.51 - 99.7
AU
Minutes

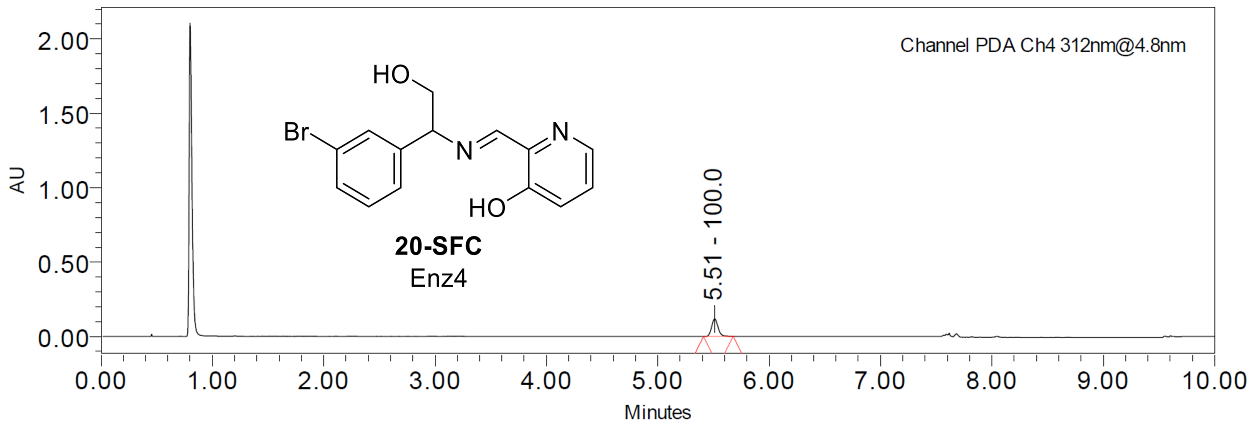

Channel PDA Ch4 312nm@4.8nm
20-SFC
Enz4
5.51 - 100.0
AU
Minutes

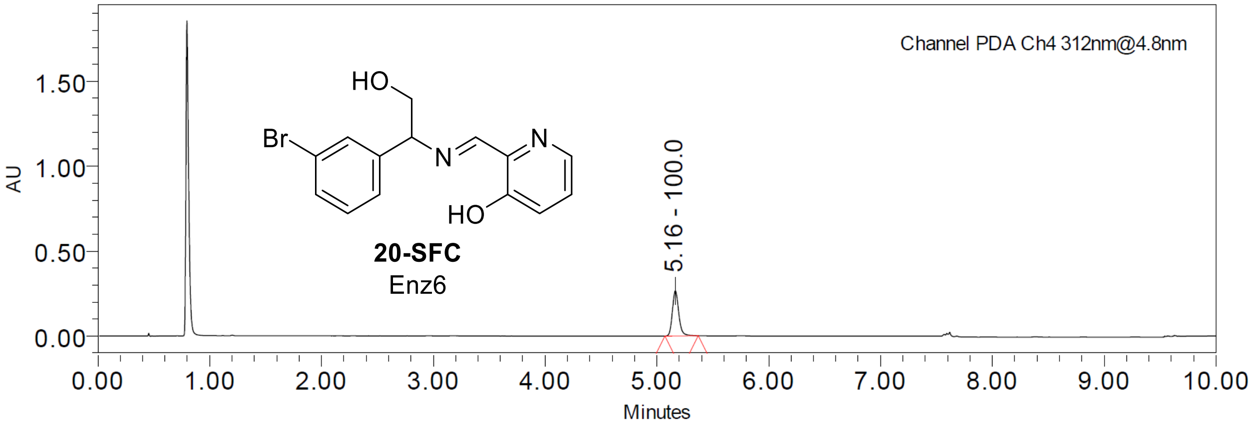
Channel PDA Ch4 312nm@4.8nm
HO
Br
N
N
HO
20-SFC
Enz6
5.16 - 100.0
AU
1.50
1.00
0.50
0.00
0.00
1.00
2.00
3.00
4.00
5.00
6.00
7.00
8.00
9.00
10.00
Minutes

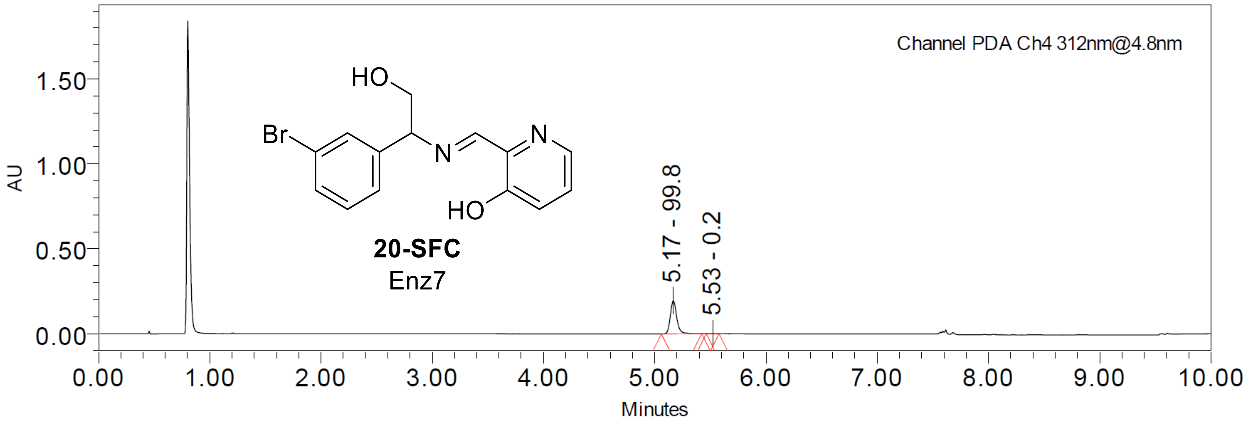
Channel PDA Ch4 312nm@4.8nm
HO
Br
N
N
HO
20-SFC
Enz7
5.17 - 99.8
5.53 - 0.2
AU
1.50
1.00
0.50
0.00
0.00
1.00
2.00
3.00
4.00
5.00
6.00
7.00
8.00
9.00
10.00
Minutes

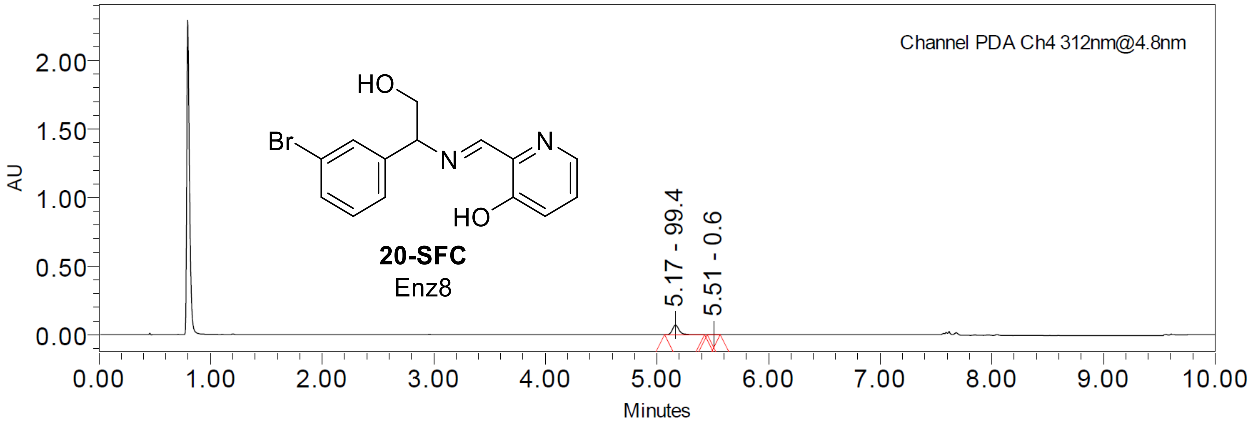
Channel PDA Ch4 312nm@4.8nm
HO
Br
N
N
HO
20-SFC
Enz8
5.17 - 99.4
5.51 - 0.6
AU
2.00
1.50
1.00
0.50
0.00
0.00
1.00
2.00
3.00
4.00
5.00
6.00
7.00
8.00
9.00
10.00
Minutes

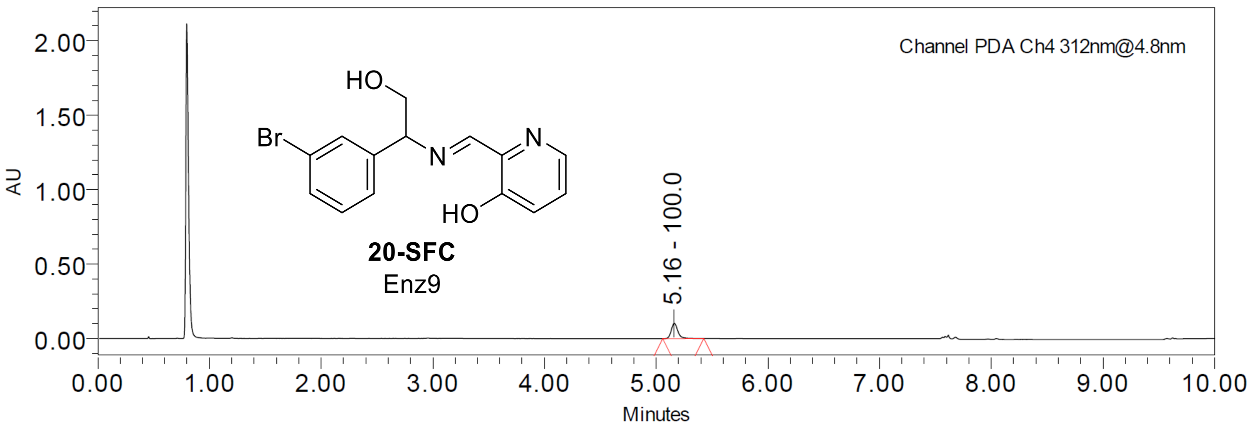
Channel PDA Ch4 312nm@4.8nm
AU
Minutes
5.16 - 100.0
20-SFC
Enz9

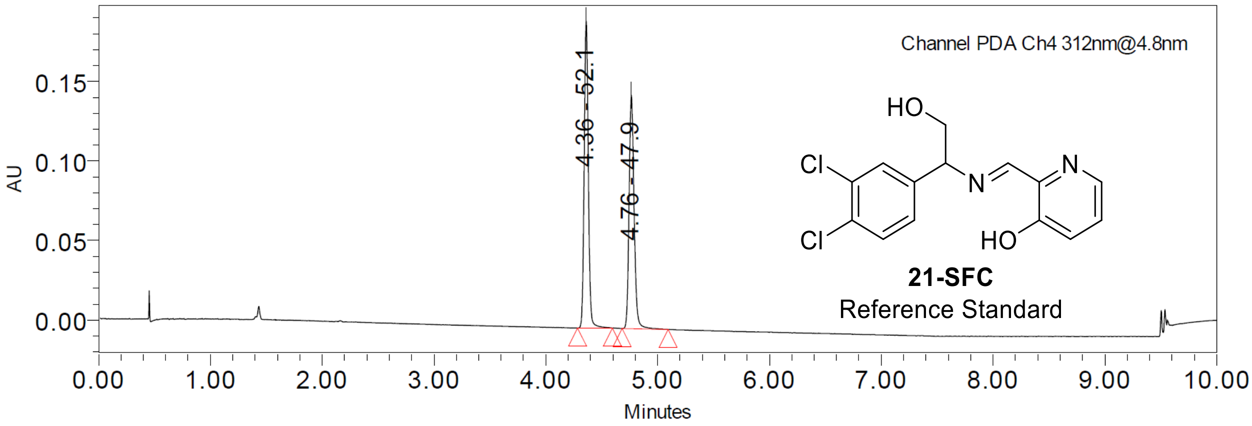
Channel PDA Ch4 312nm@4.8nm
AU
Minutes
4.36 - 52.1
4.76 - 47.9
21-SFC
Reference Standard

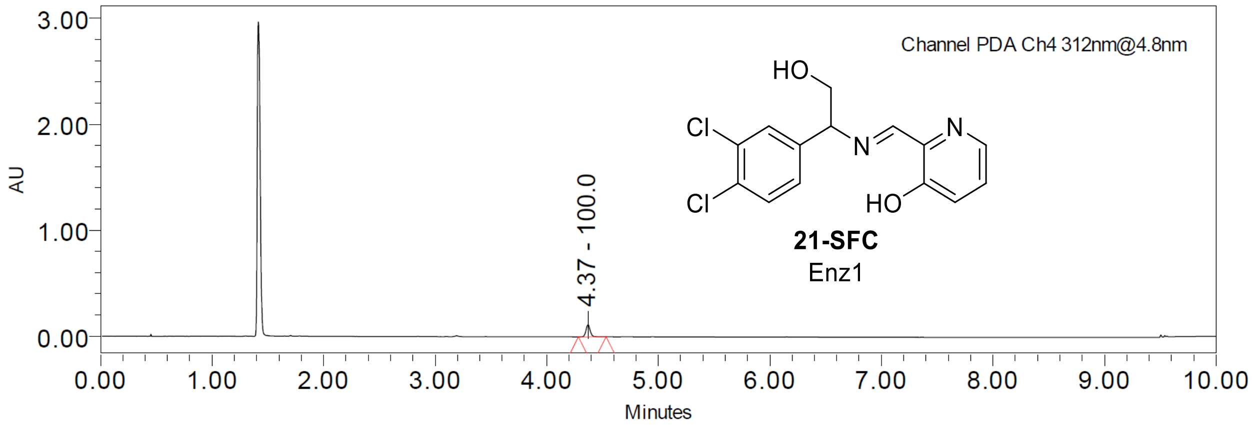
Channel PDA Ch4 312nm@4.8nm
AU
Minutes
4.37 - 100.0
21-SFC
Enz1

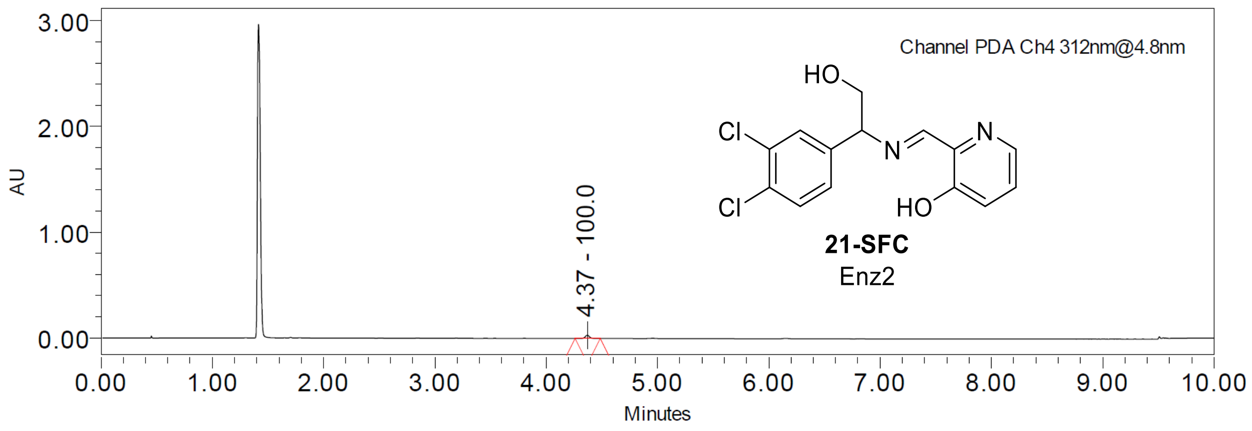

Channel PDA Ch4 312nm@4.8nm
HO
Cl
Cl
N
N
HO
21-SFC
Enz2
4.37 - 100.0
AU
3.00
2.00
1.00
0.00
0.00
1.00
2.00
3.00
4.00
5.00
6.00
7.00
8.00
9.00
10.00
Minutes

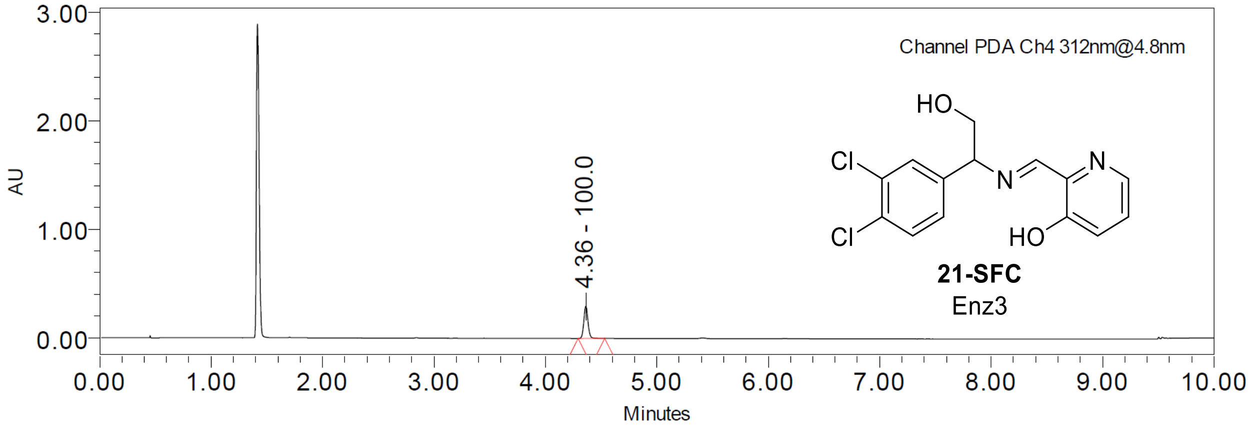

Channel PDA Ch4 312nm@4.8nm
HO
Cl
Cl
N
N
HO
21-SFC
Enz3
4.36 - 100.0
AU
3.00
2.00
1.00
0.00
0.00
1.00
2.00
3.00
4.00
5.00
6.00
7.00
8.00
9.00
10.00
Minutes

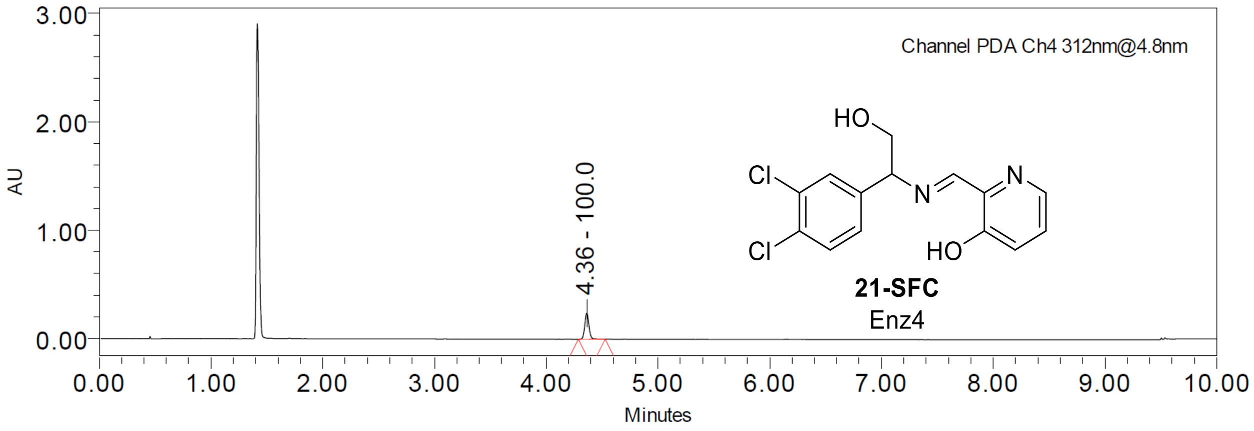

Channel PDA Ch4 312nm@4.8nm
HO
Cl
Cl
N
N
HO
21-SFC
Enz4
4.36 - 100.0
AU
3.00
2.00
1.00
0.00
0.00
1.00
2.00
3.00
4.00
5.00
6.00
7.00
8.00
9.00
10.00
Minutes

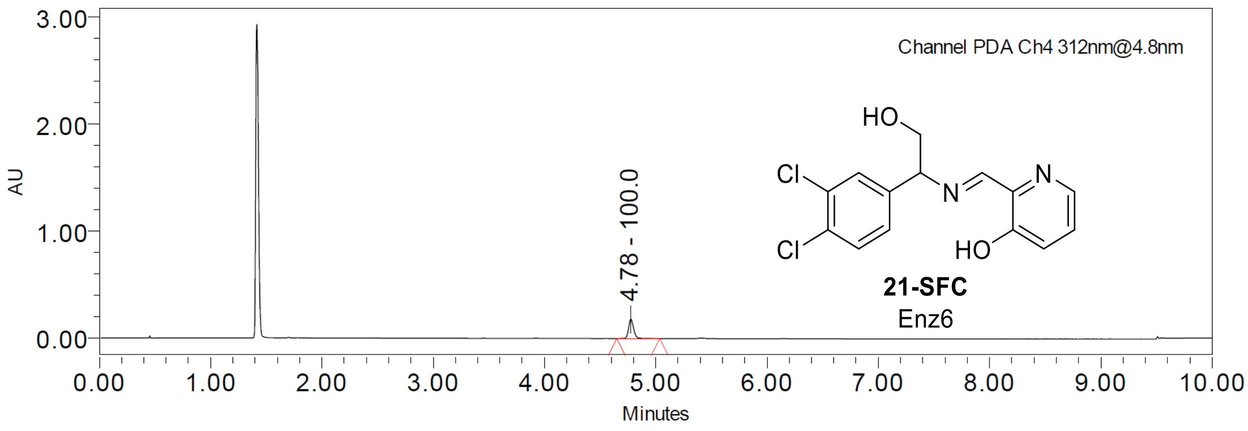

Channel PDA Ch4 312nm@4.8nm
4.78 - 100.0
21-SFC
Enz6
AU
Minutes

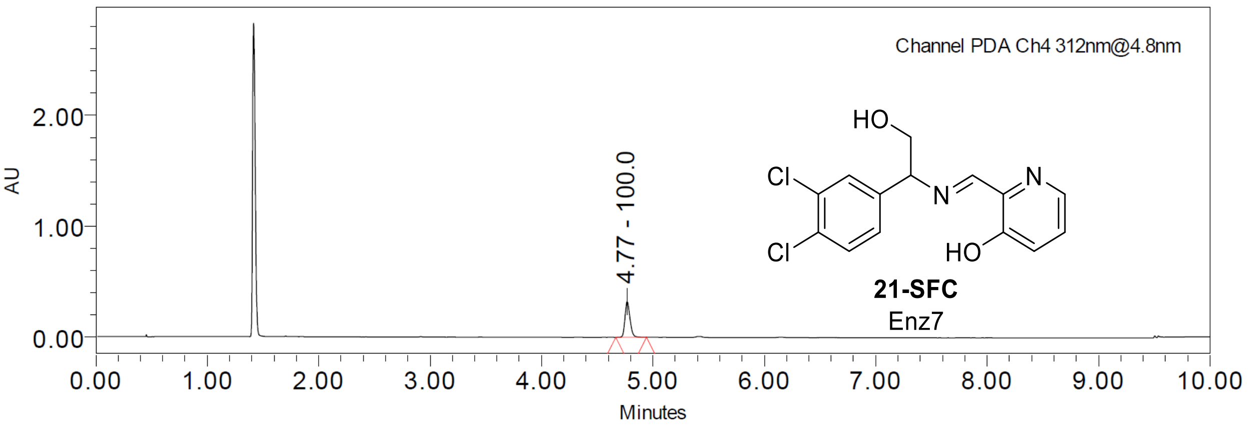

Channel PDA Ch4 312nm@4.8nm
4.77 - 100.0
21-SFC
Enz7
AU
Minutes

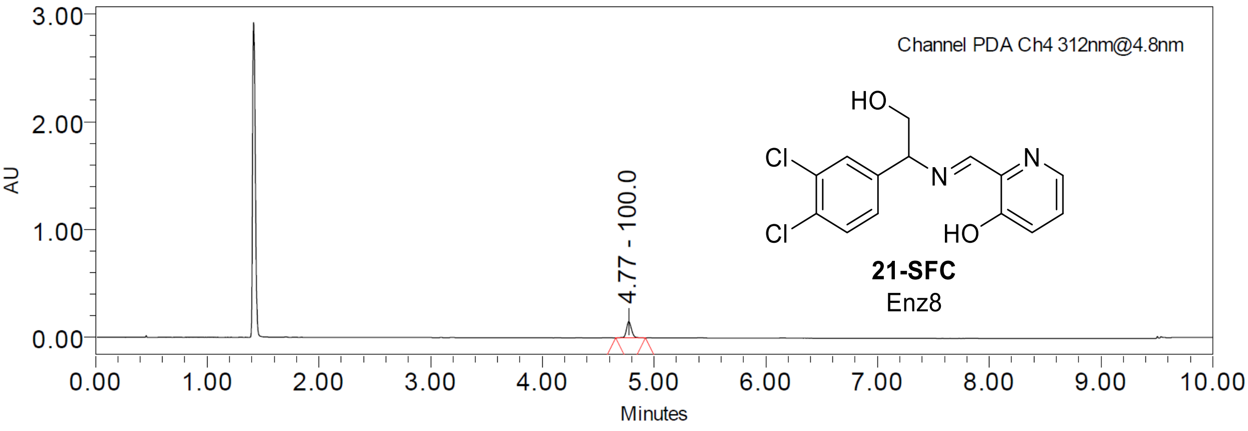

Channel PDA Ch4 312nm@4.8nm
4.77 - 100.0
21-SFC
Enz8
AU
Minutes

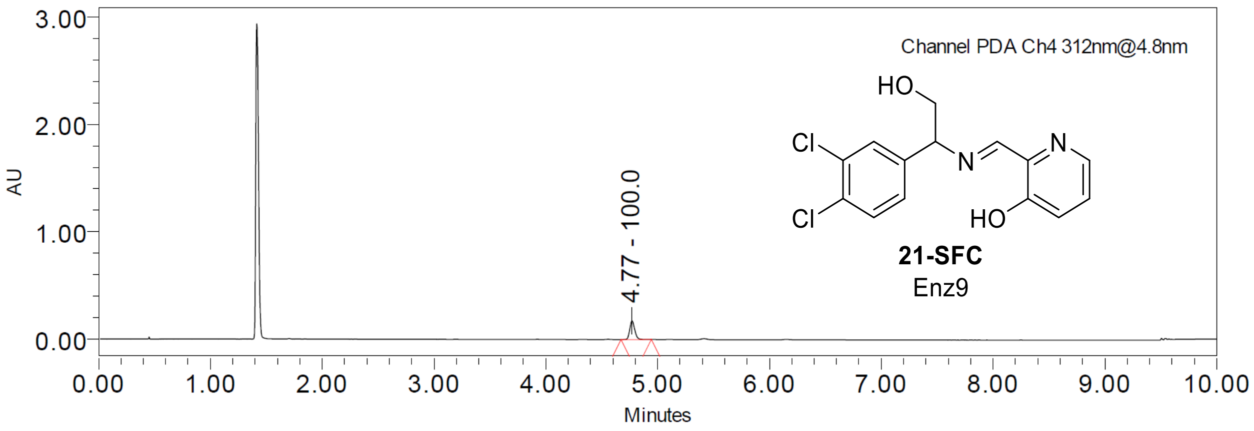
Channel PDA Ch4 312nm@4.8nm
HO
Cl
Cl
N
N
HO
21-SFC
Enz9
4.77 - 100.0
AU
Minutes

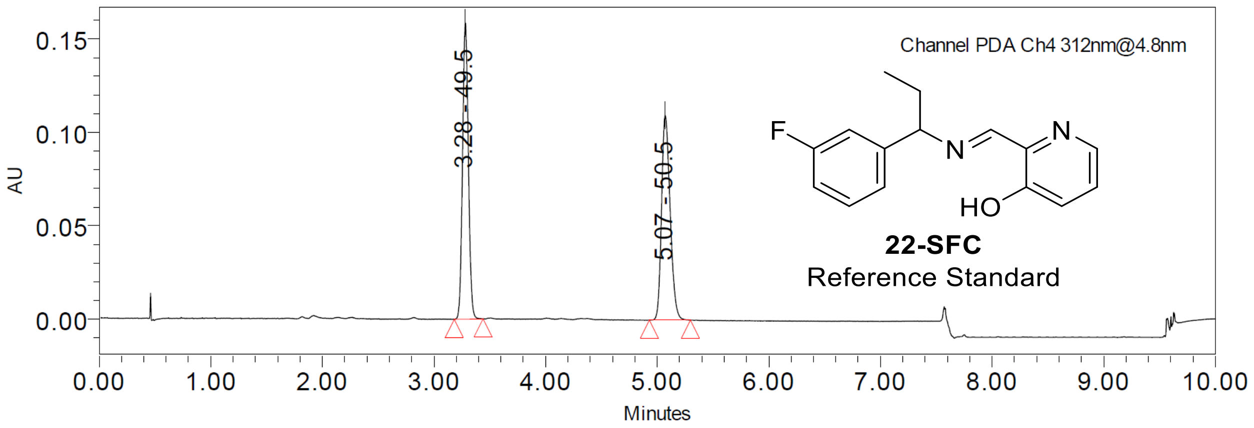
Channel PDA Ch4 312nm@4.8nm
F
N
N
HO
22-SFC
Reference Standard
3.28 - 49.5
5.07 - 50.5
AU
Minutes

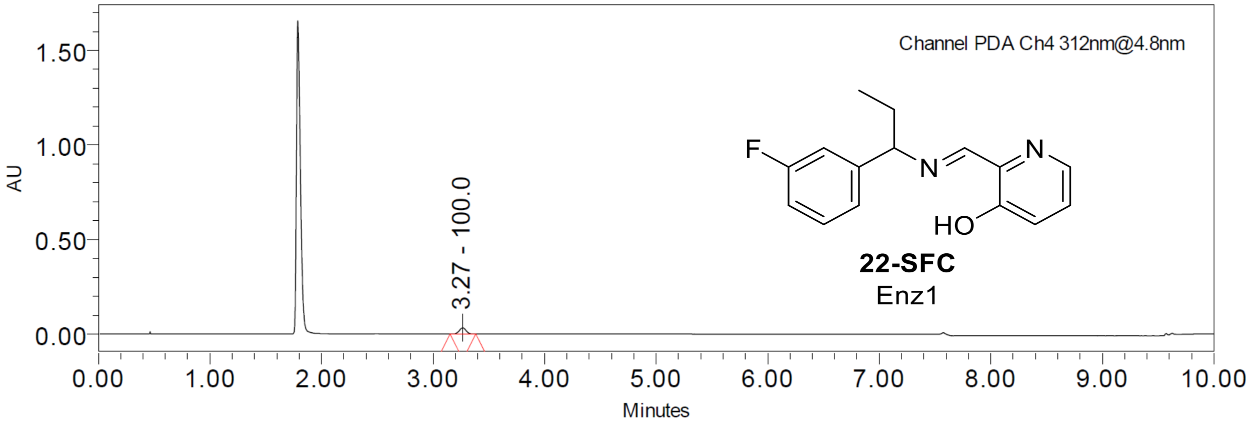
Channel PDA Ch4 312nm@4.8nm
F
N
N
HO
22-SFC
Enz1
3.27 - 100.0
AU
Minutes

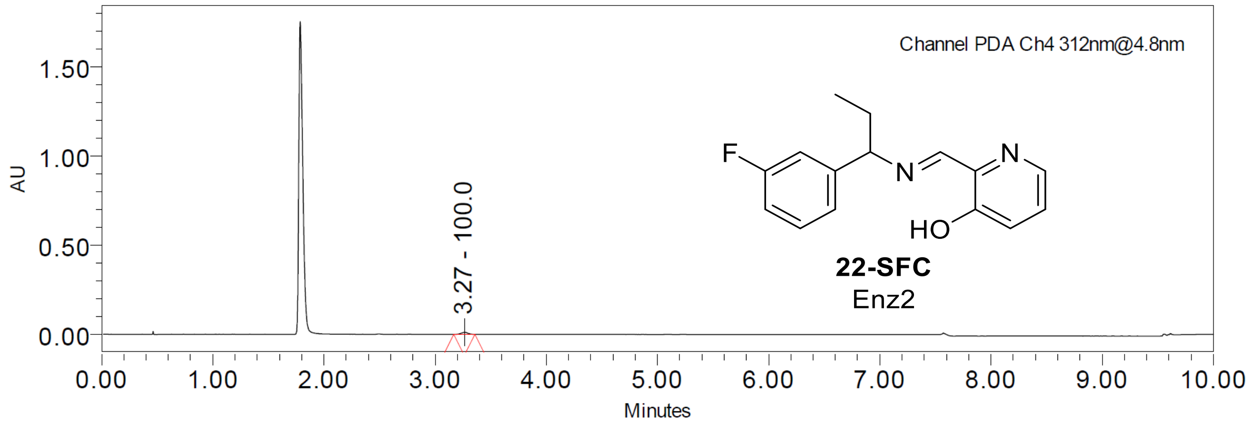
Channel PDA Ch4 312nm@4.8nm
3.27 - 100.0
AU
Minutes
22-SFC
Enz2

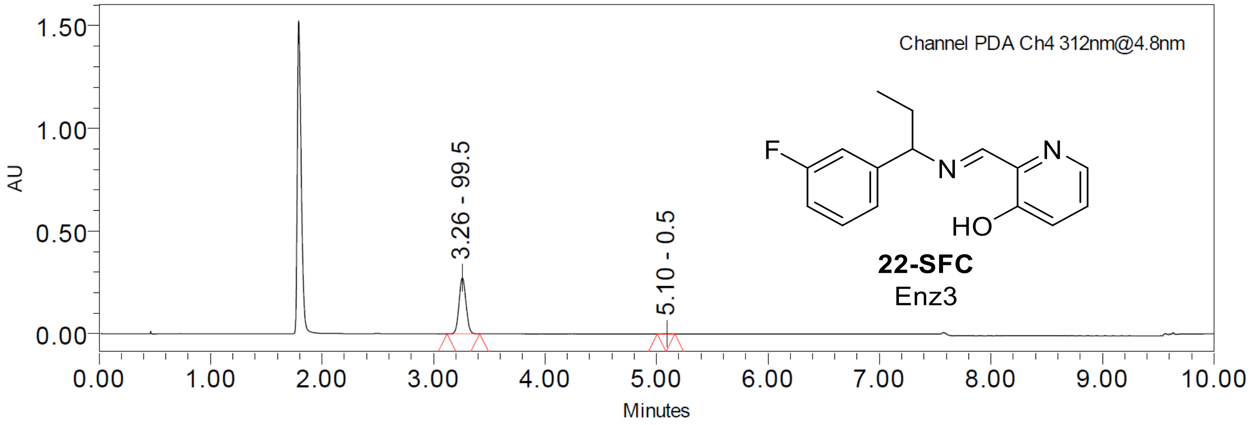
Channel PDA Ch4 312nm@4.8nm
3.26 - 99.5
5.10 - 0.5
AU
Minutes
22-SFC
Enz3

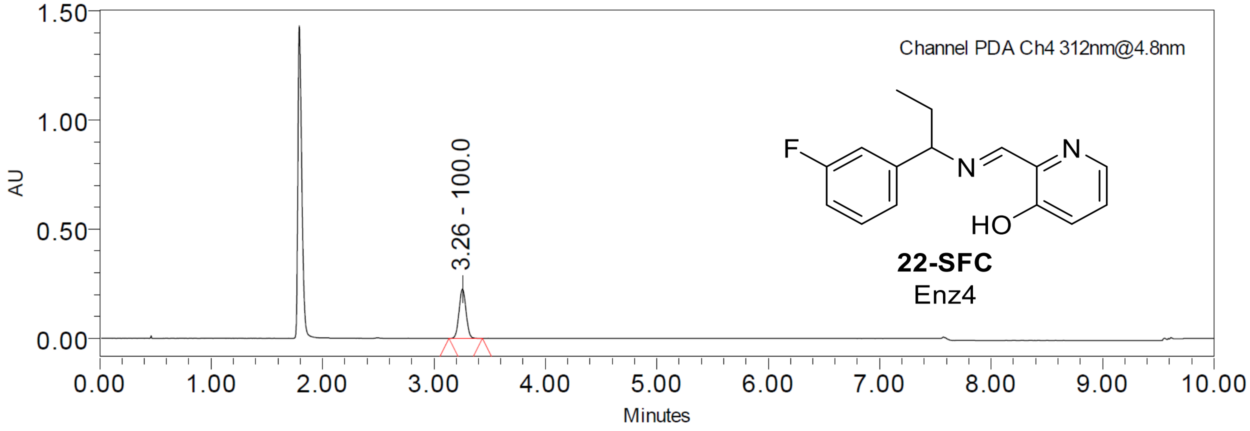
Channel PDA Ch4 312nm@4.8nm
3.26 - 100.0
AU
Minutes
22-SFC
Enz4

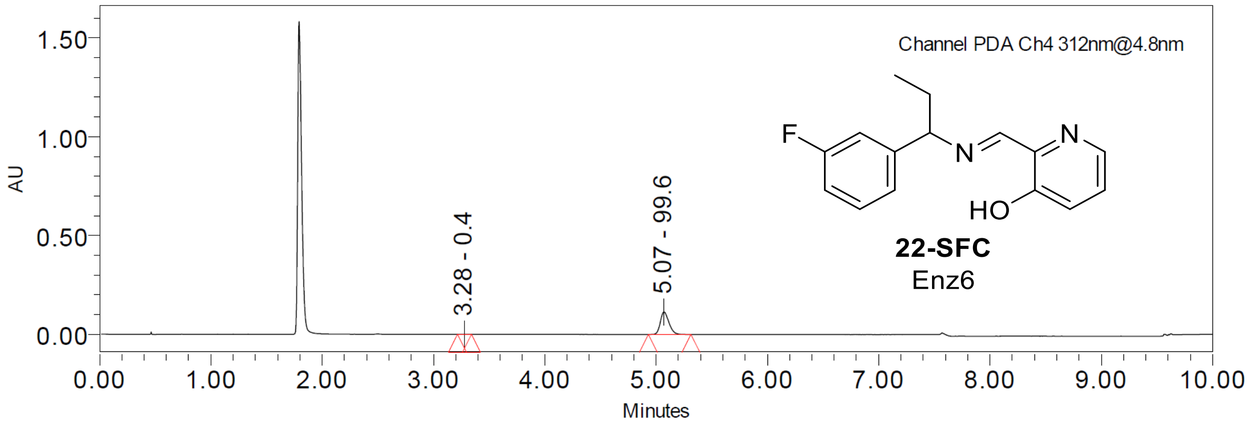
Channel PDA Ch4 312nm@4.8nm
F
N
N
HO
22-SFC
Enz6
3.28 - 0.4
5.07 - 99.6
AU
Minutes

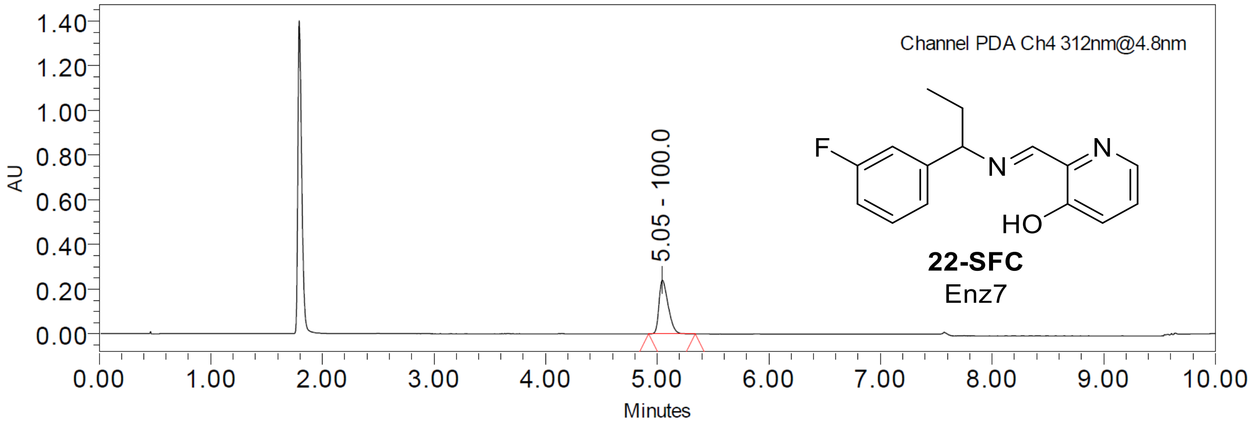
Channel PDA Ch4 312nm@4.8nm
F
N
N
HO
22-SFC
Enz7
5.05 - 100.0
AU
Minutes

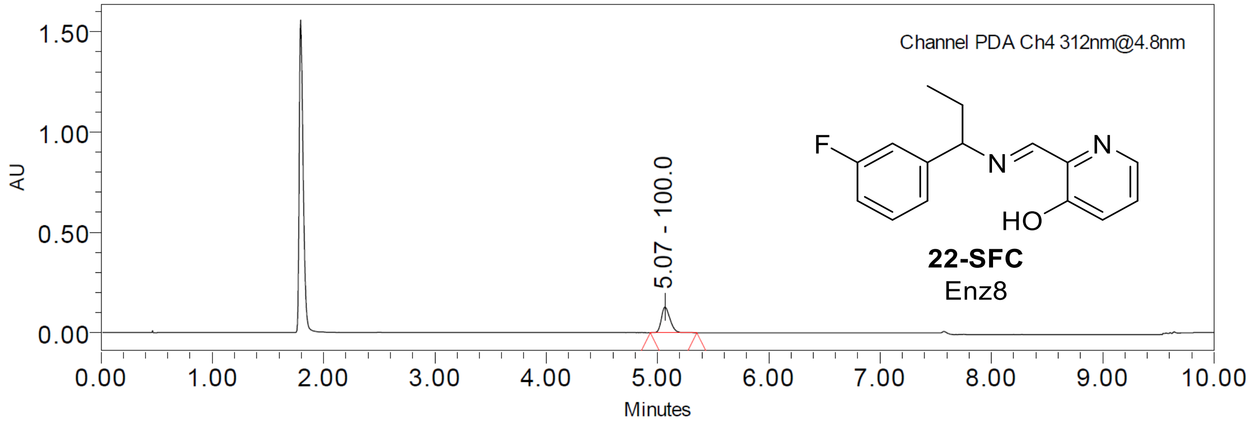
Channel PDA Ch4 312nm@4.8nm
F
N
N
HO
22-SFC
Enz8
5.07 - 100.0
AU
Minutes

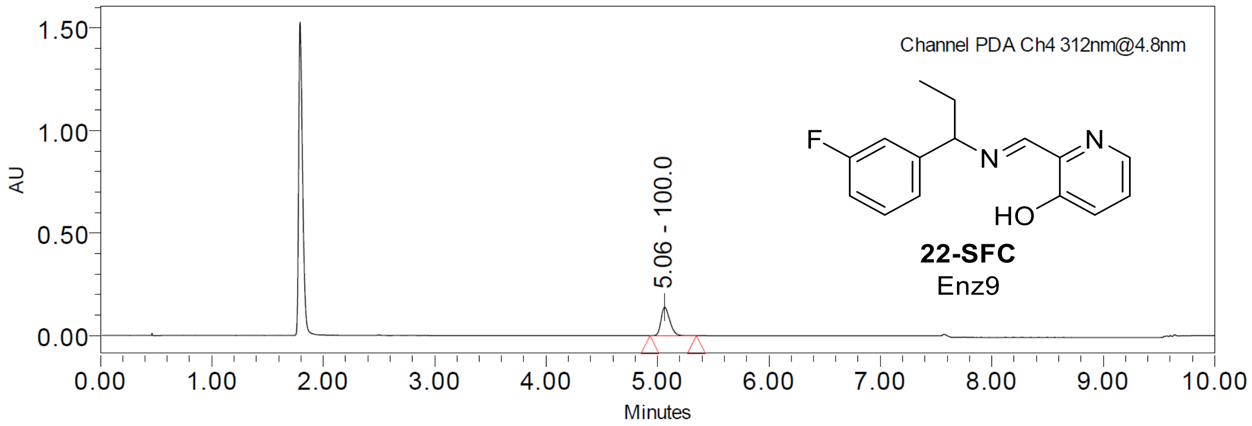
Channel PDA Ch4 312nm@4.8nm
5.06 - 100.0
F
N
N
HO
22-SFC
Enz9
AU
Minutes

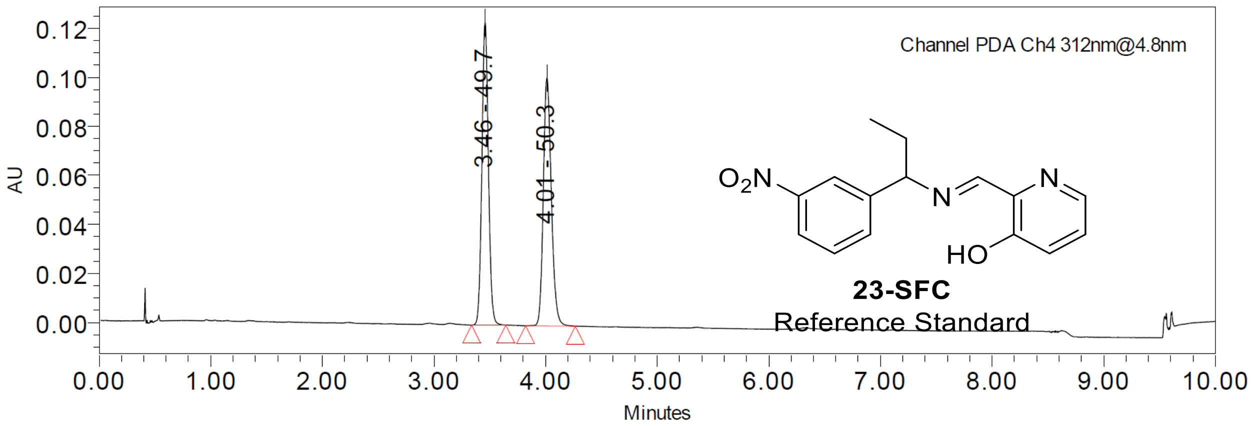
Channel PDA Ch4 312nm@4.8nm
3.46 - 49.7
4.01 - 50.3
O2N
N
N
HO
23-SFC
Reference Standard
AU
Minutes

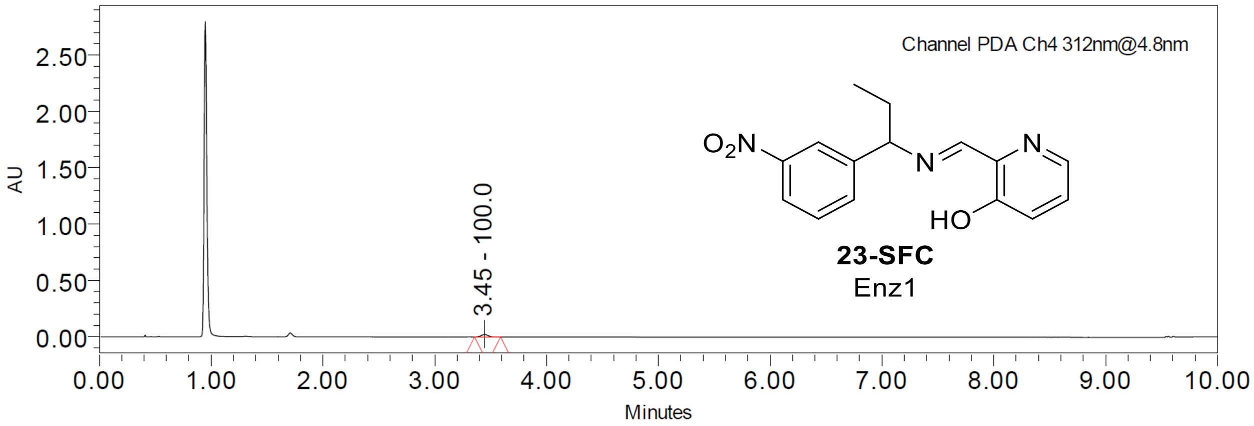
Channel PDA Ch4 312nm@4.8nm
3.45 - 100.0
O2N
N
N
HO
23-SFC
Enz1
AU
Minutes

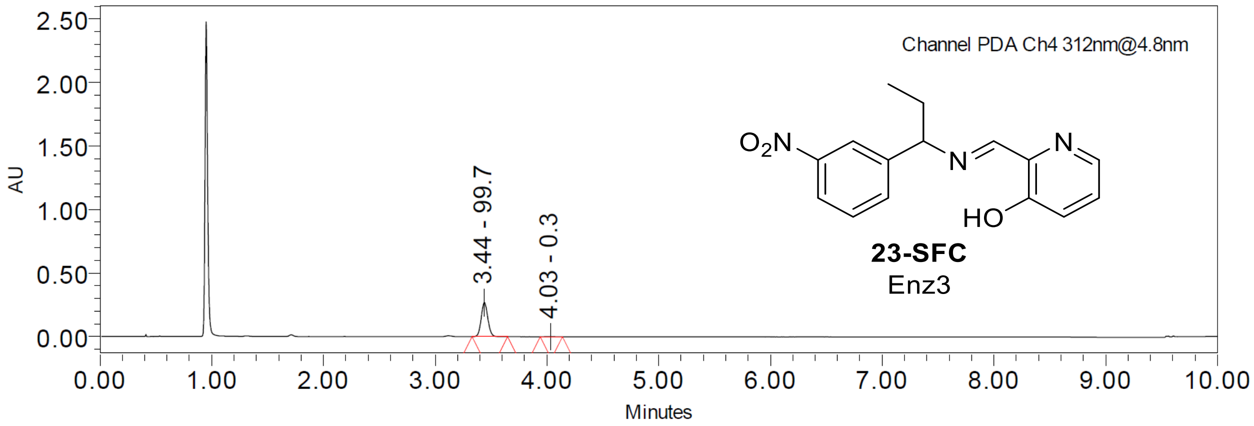

Channel PDA Ch4 312nm@4.8nm
AU
Minutes
3.44 - 99.7
4.03 - 0.3
O2N
N
N
HO
23-SFC
Enz3

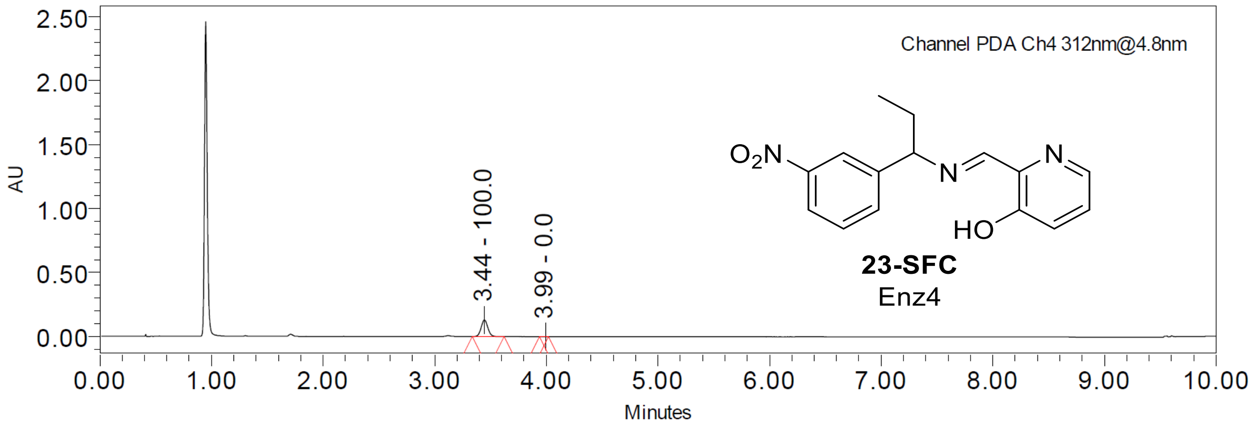

Channel PDA Ch4 312nm@4.8nm
AU
Minutes
3.44 - 100.0
3.99 - 0.0
O2N
N
N
HO
23-SFC
Enz4

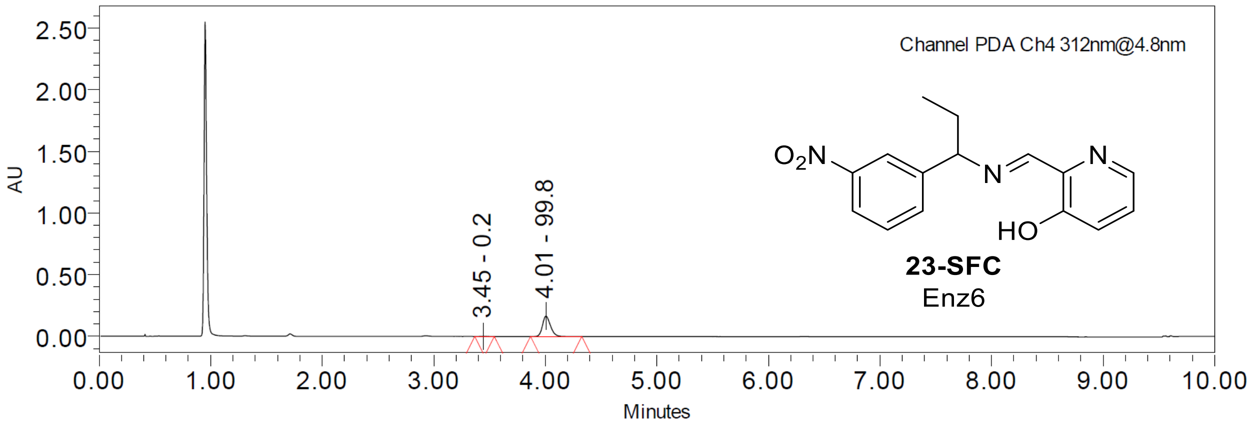

Channel PDA Ch4 312nm@4.8nm
AU
Minutes
3.45 - 0.2
4.01 - 99.8
O2N
N
N
HO
23-SFC
Enz6

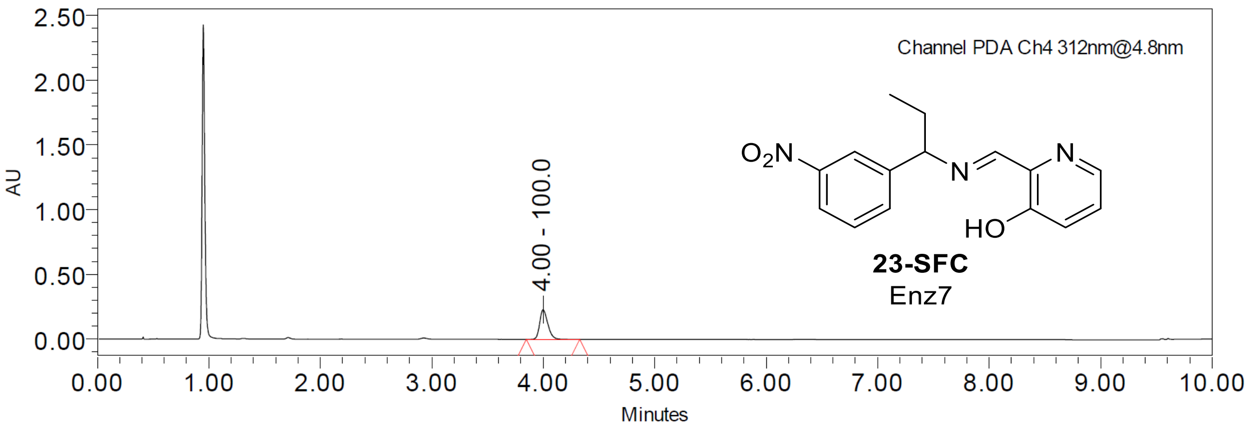
Channel PDA Ch4 312nm@4.8nm
AU
Minutes
4.00 - 100.0
O_2N
N
N
HO
23-SFC
Enz7

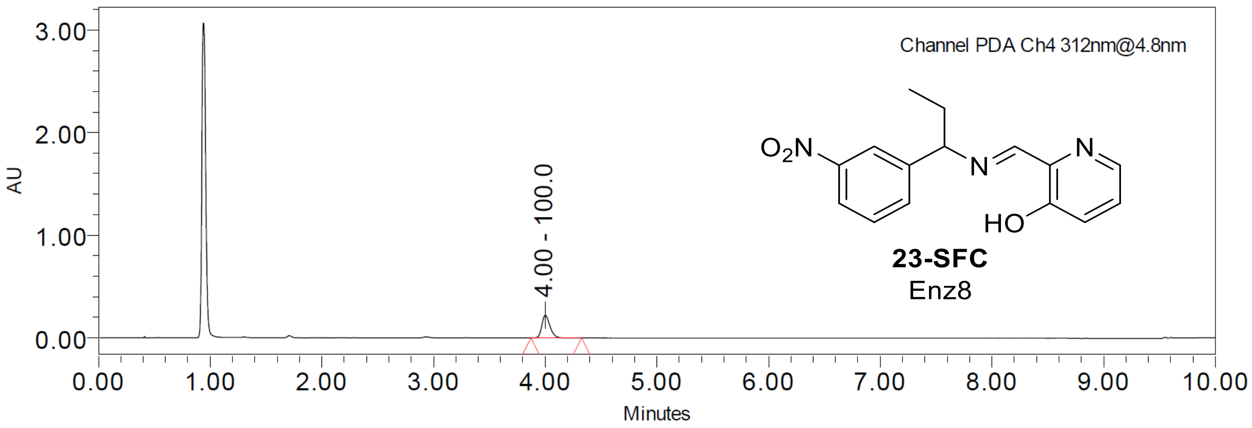
Channel PDA Ch4 312nm@4.8nm
AU
Minutes
4.00 - 100.0
O_2N
N
N
HO
23-SFC
Enz8

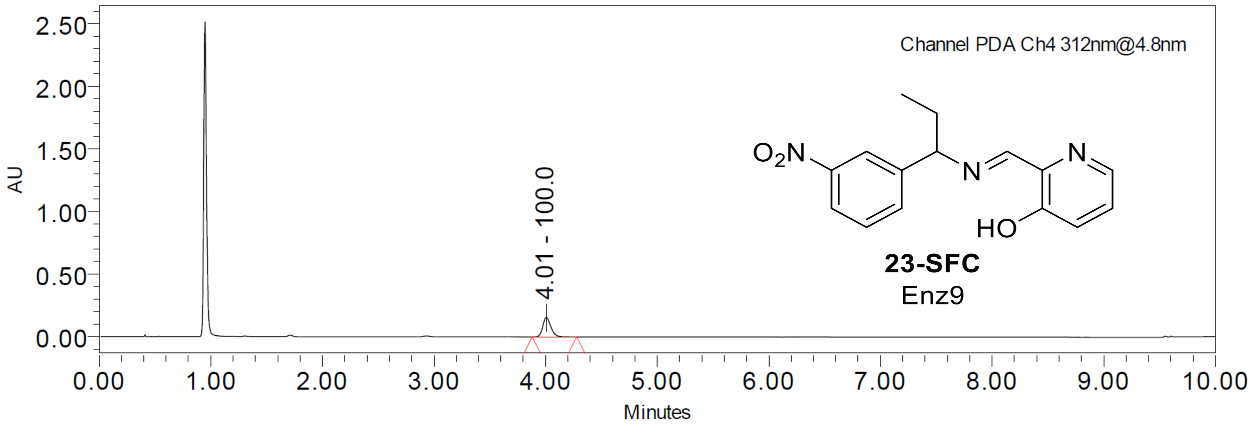
Channel PDA Ch4 312nm@4.8nm
AU
Minutes
4.01 - 100.0
O_2N
N
N
HO
23-SFC
Enz9

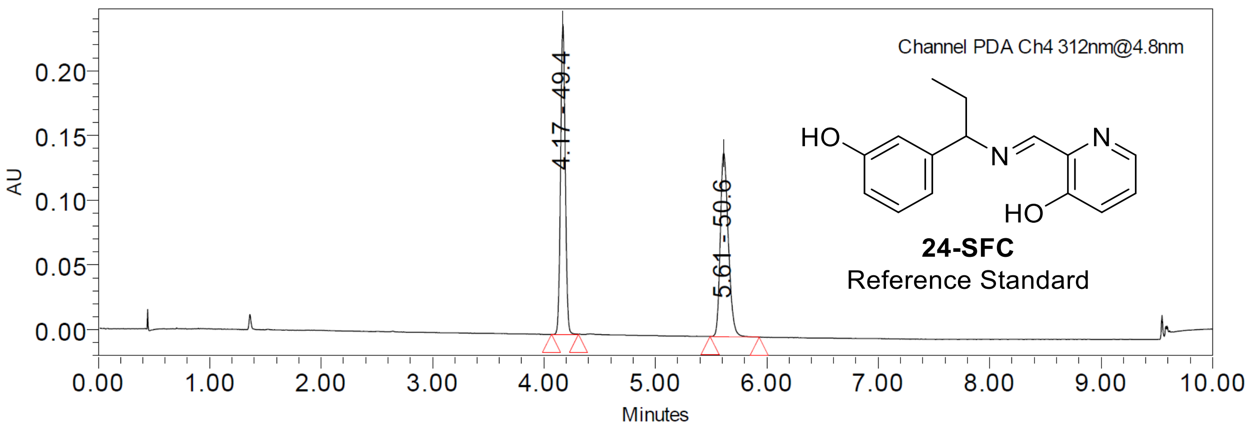

Channel PDA Ch4 312nm@4.8nm
4.17 - 49.4
5.61 - 50.6
HO
N
N
HO
24-SFC
Reference Standard
AU
Minutes

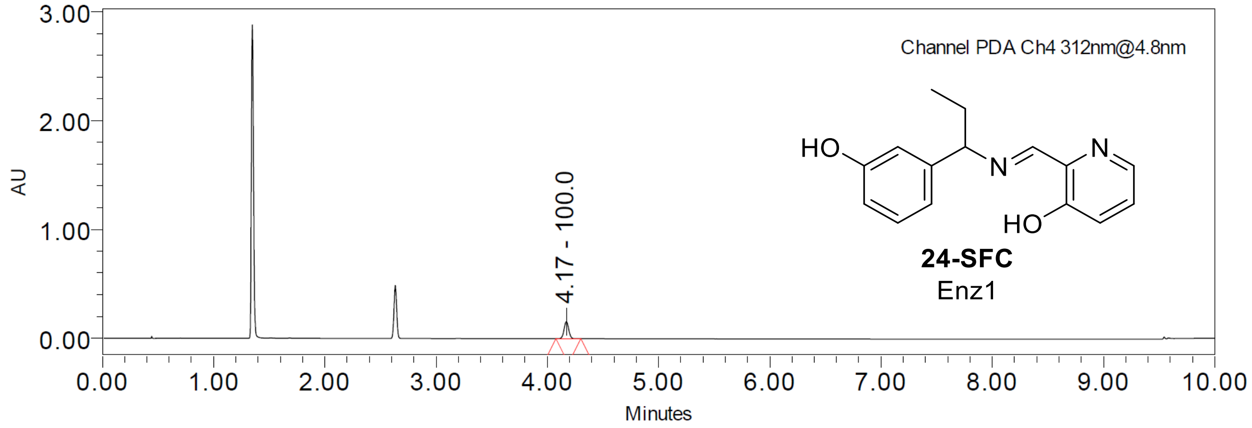

Channel PDA Ch4 312nm@4.8nm
4.17 - 100.0
HO
N
N
HO
24-SFC
Enz1
AU
Minutes

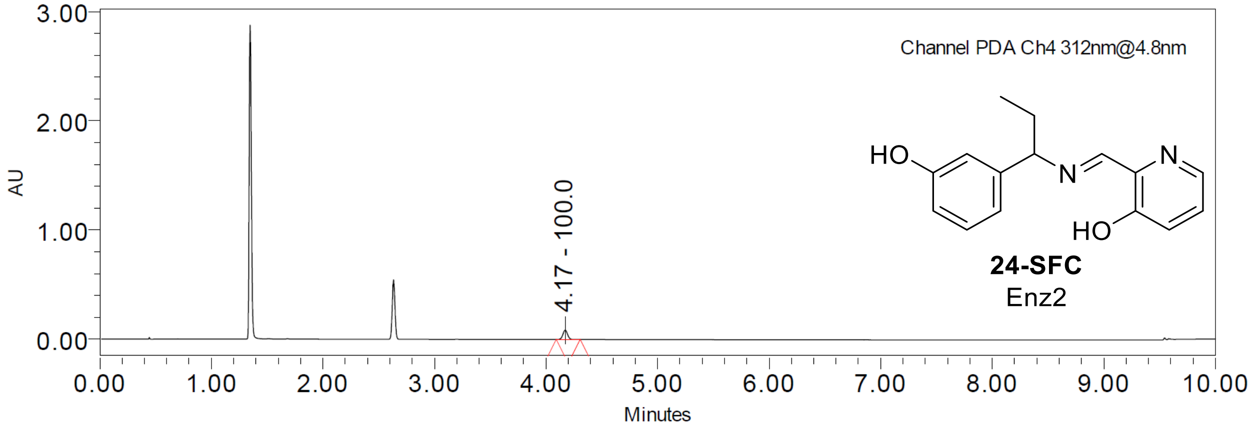

Channel PDA Ch4 312nm@4.8nm
4.17 - 100.0
HO
N
N
HO
24-SFC
Enz2
AU
Minutes

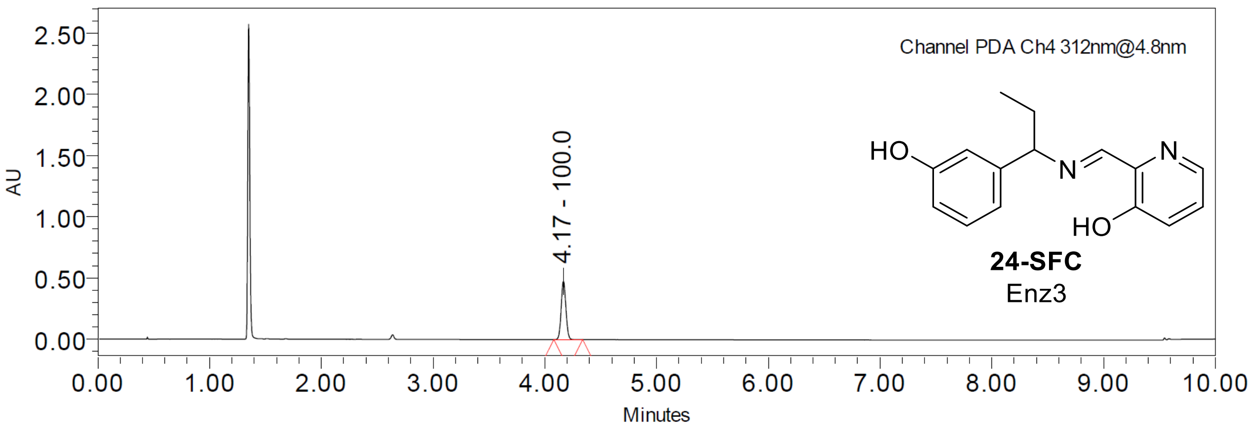

Channel PDA Ch4 312nm@4.8nm
HO
N
N
HO
24-SFC
Enz3
4.17 - 100.0
AU
Minutes

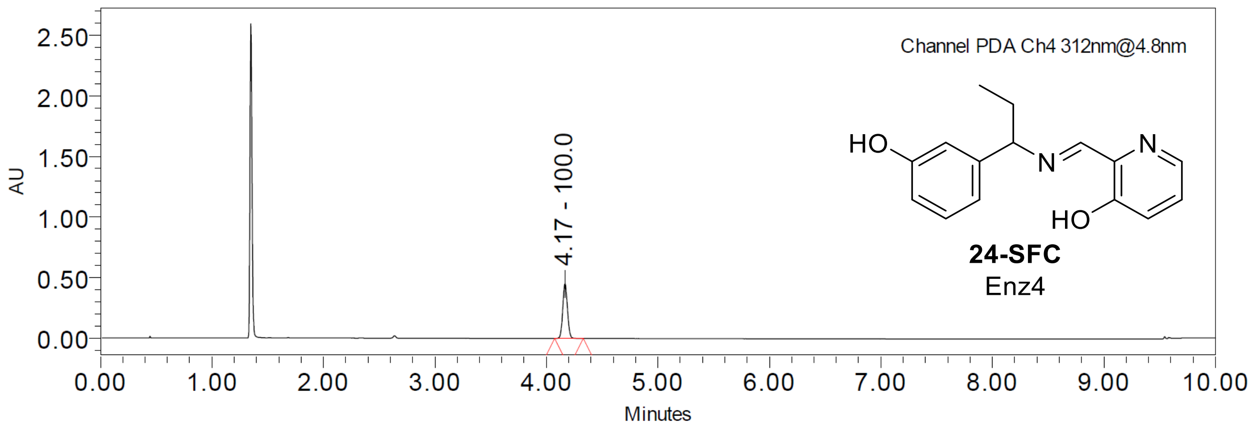

Channel PDA Ch4 312nm@4.8nm
HO
N
N
HO
24-SFC
Enz4
4.17 - 100.0
AU
Minutes

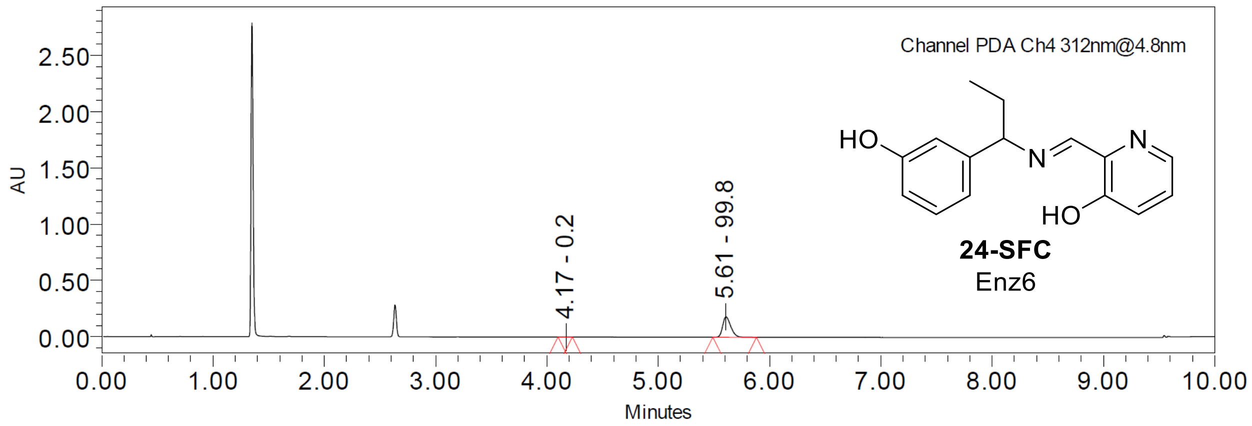

Channel PDA Ch4 312nm@4.8nm
HO
N
N
HO
24-SFC
Enz6
4.17 - 0.2
5.61 - 99.8
AU
Minutes

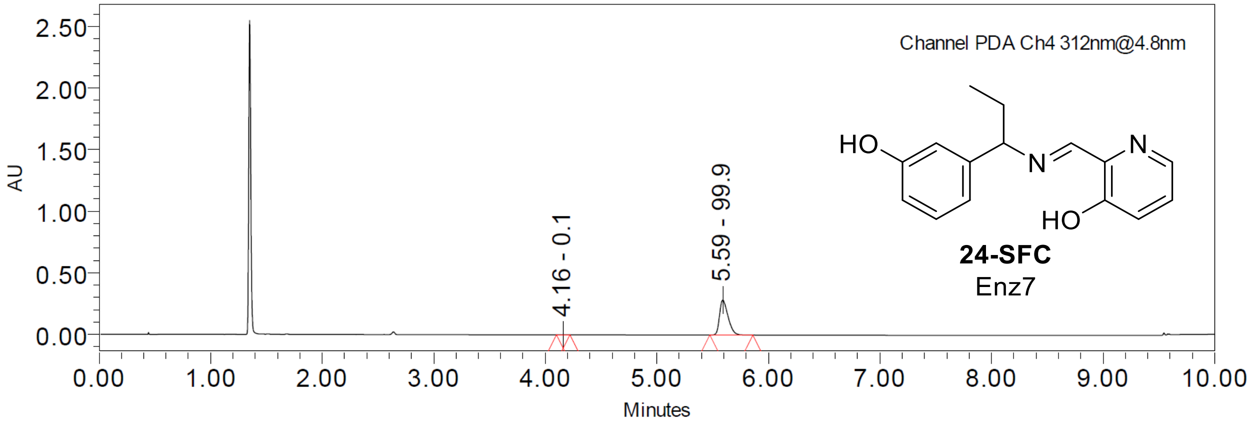
Channel PDA Ch4 312nm@4.8nm
AU
Minutes
4.16 - 0.1
5.59 - 99.9
HO
N
N
HO
24-SFC
Enz7

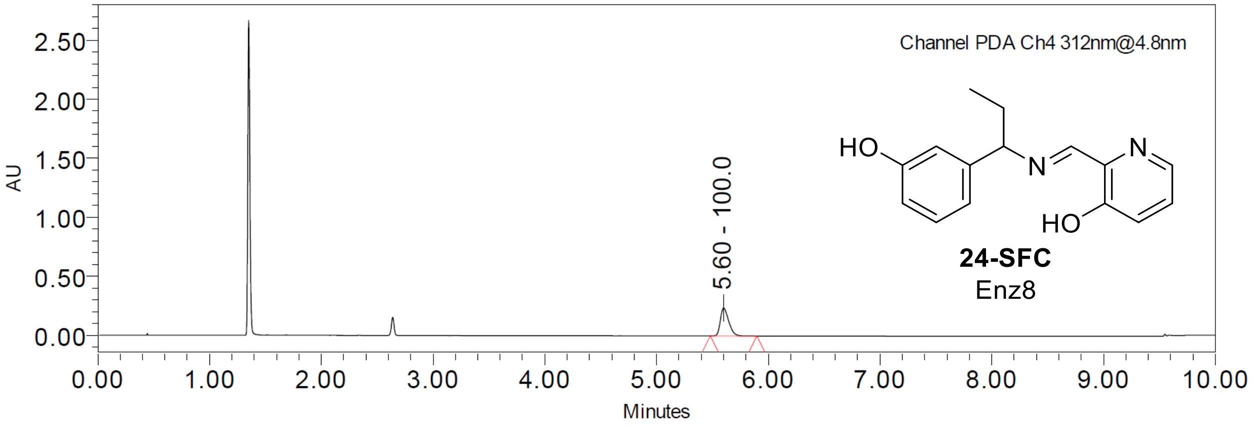
Channel PDA Ch4 312nm@4.8nm
AU
Minutes
5.60 - 100.0
HO
N
N
HO
24-SFC
Enz8

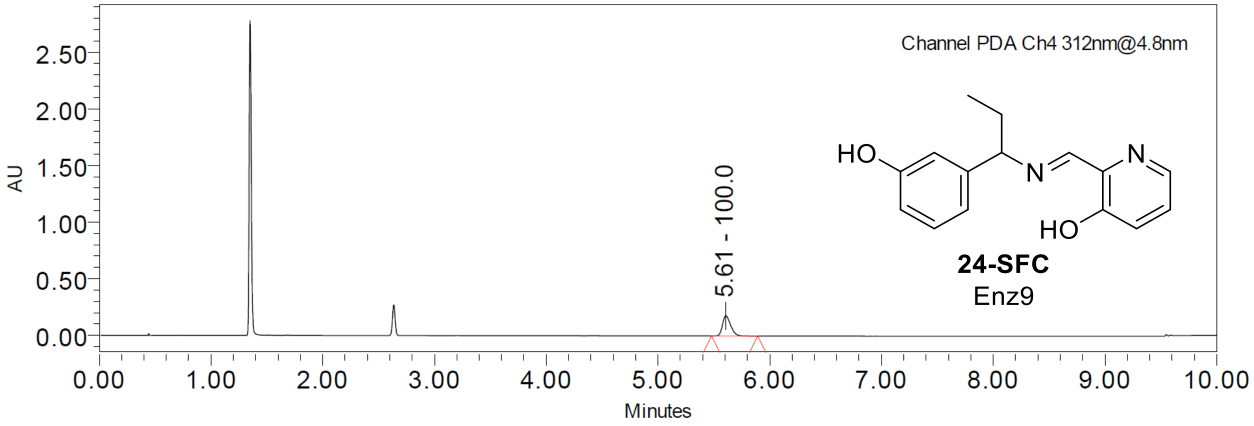
Channel PDA Ch4 312nm@4.8nm
AU
Minutes
5.61 - 100.0
HO
N
N
HO
24-SFC
Enz9

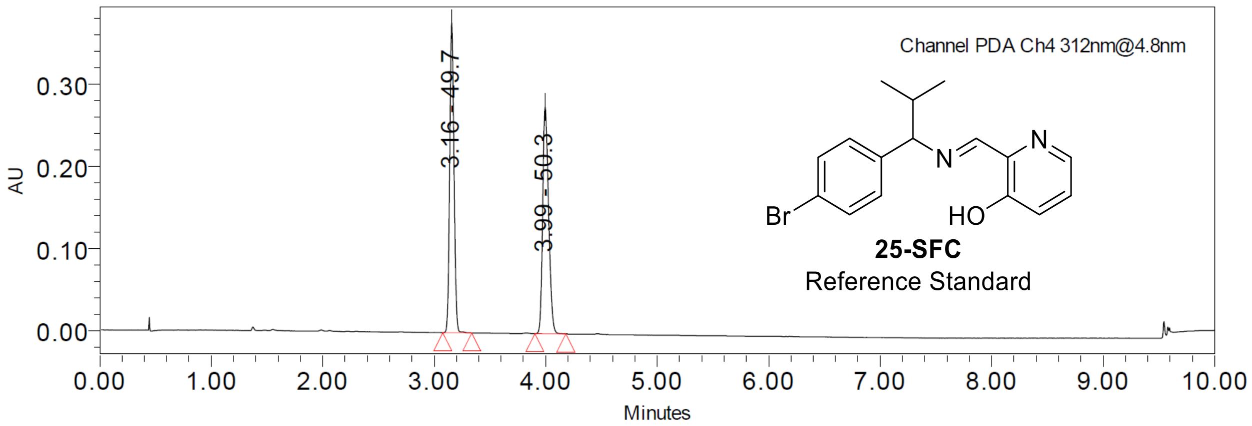
Channel PDA Ch4 312nm@4.8nm
AU
0.30
0.20
0.10
0.00
0.00
1.00
2.00
3.00
4.00
5.00
6.00
7.00
8.00
9.00
10.00
Minutes
3.16 - 49.7
3.99 - 50.3
N
N
Br
HO
25-SFC
Reference Standard

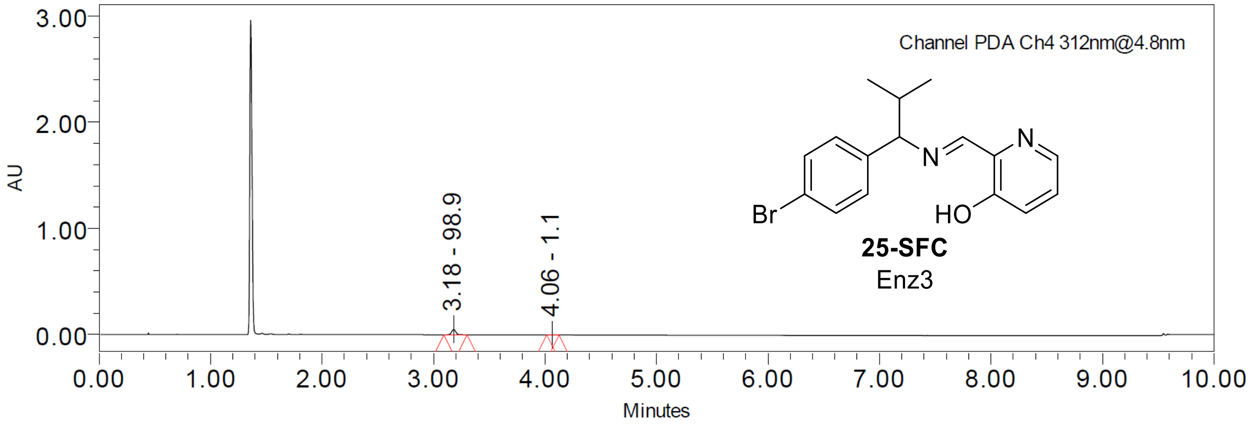
Channel PDA Ch4 312nm@4.8nm
AU
3.00
2.00
1.00
0.00
0.00
1.00
2.00
3.00
4.00
5.00
6.00
7.00
8.00
9.00
10.00
Minutes
3.18 - 98.9
4.06 - 1.1
N
N
Br
HO
25-SFC
Enz3

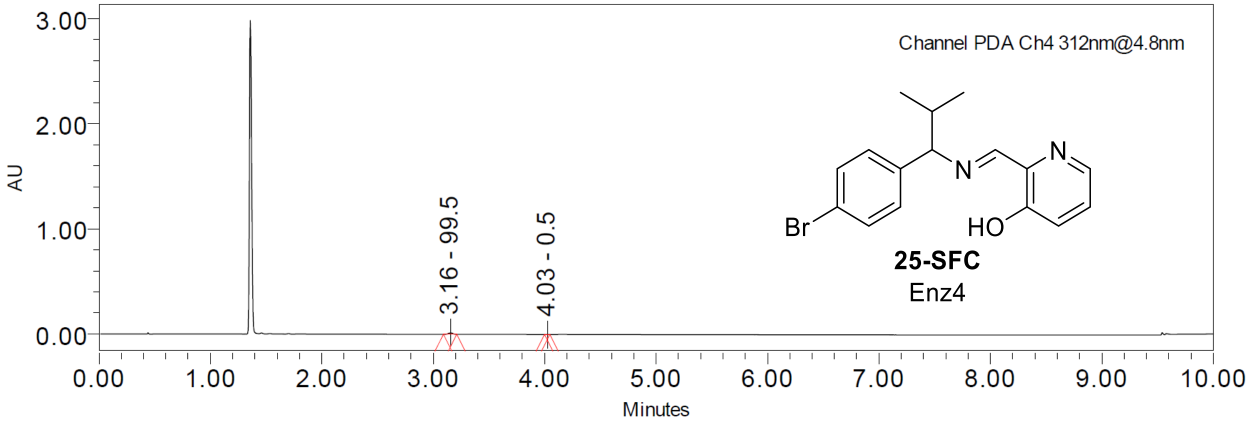
Channel PDA Ch4 312nm@4.8nm
AU
3.00
2.00
1.00
0.00
0.00
1.00
2.00
3.00
4.00
5.00
6.00
7.00
8.00
9.00
10.00
Minutes
3.16 - 99.5
4.03 - 0.5
N
N
Br
HO
25-SFC
Enz4

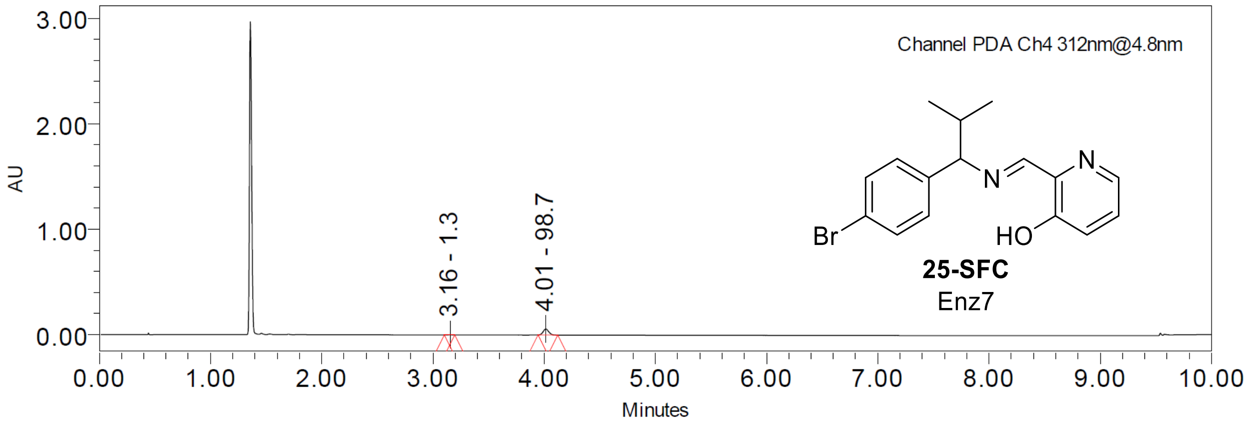
Channel PDA Ch4 312nm@4.8nm
AU
Minutes
3.16 - 1.3
4.01 - 98.7
Br
N
N
HO
25-SFC
Enz7

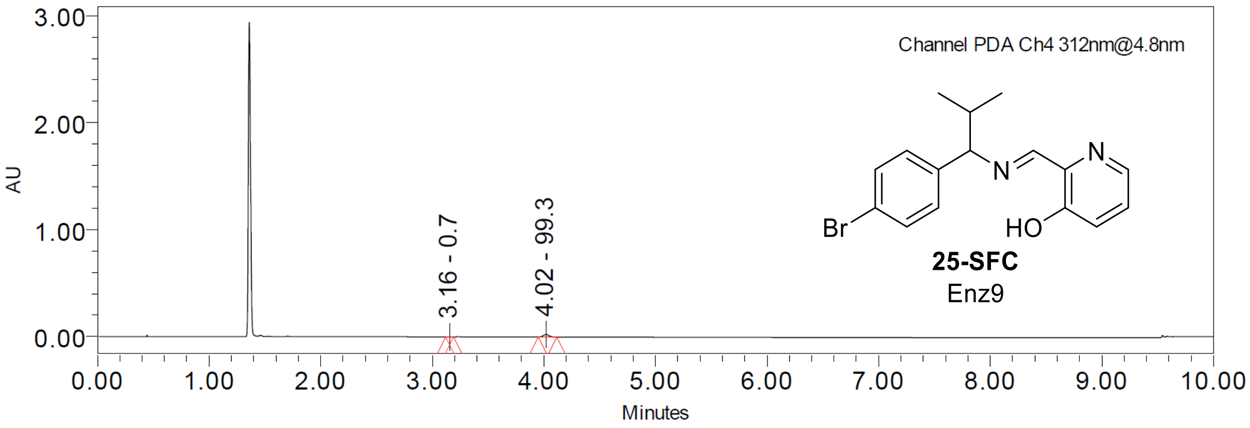
Channel PDA Ch4 312nm@4.8nm
AU
Minutes
3.16 - 0.7
4.02 - 99.3
Br
N
N
HO
25-SFC
Enz9

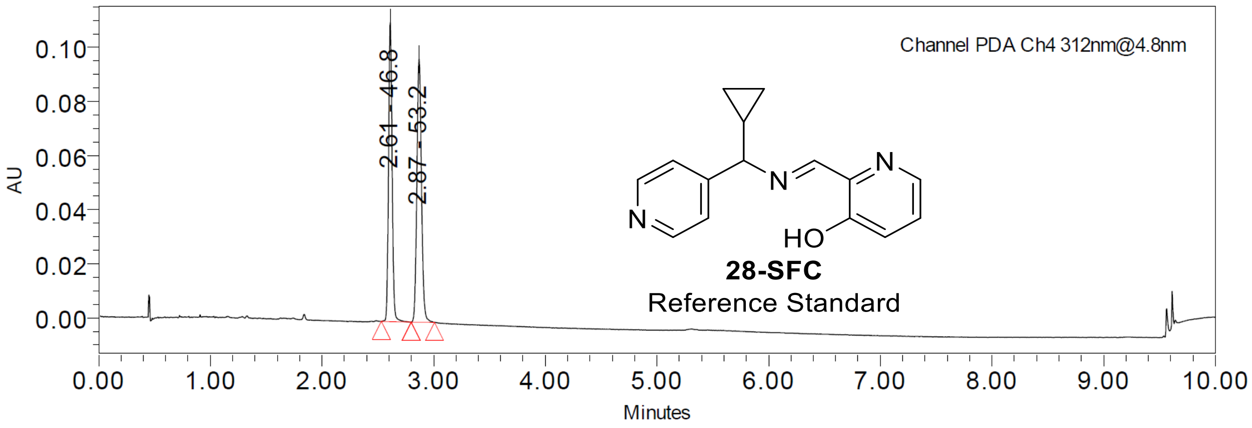
Channel PDA Ch4 312nm@4.8nm
AU
Minutes
2.61 - 46.8
2.87 - 53.2
N
N
N
HO
28-SFC
Reference Standard

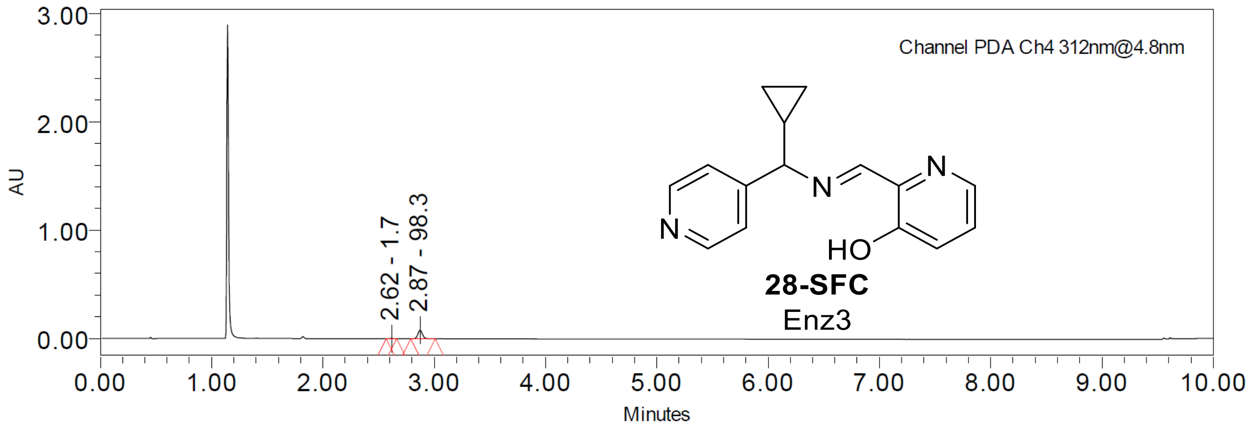
Channel PDA Ch4 312nm@4.8nm
AU
Minutes
2.62 - 1.7
2.87 - 98.3
N
N
N
HO
28-SFC
Enz3

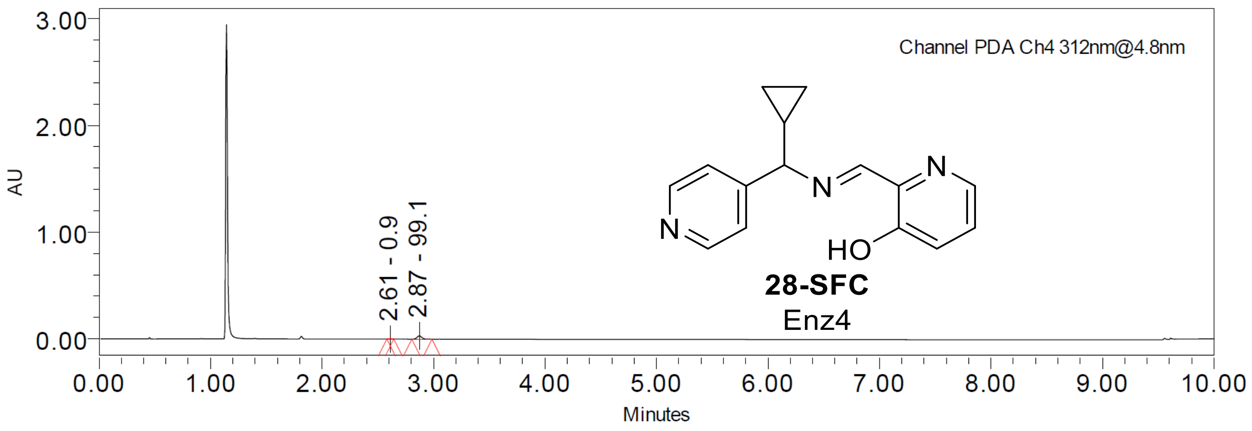
Channel PDA Ch4 312nm@4.8nm
AU
Minutes
2.61 - 0.9
2.87 - 99.1
N
N
N
HO
28-SFC
Enz4

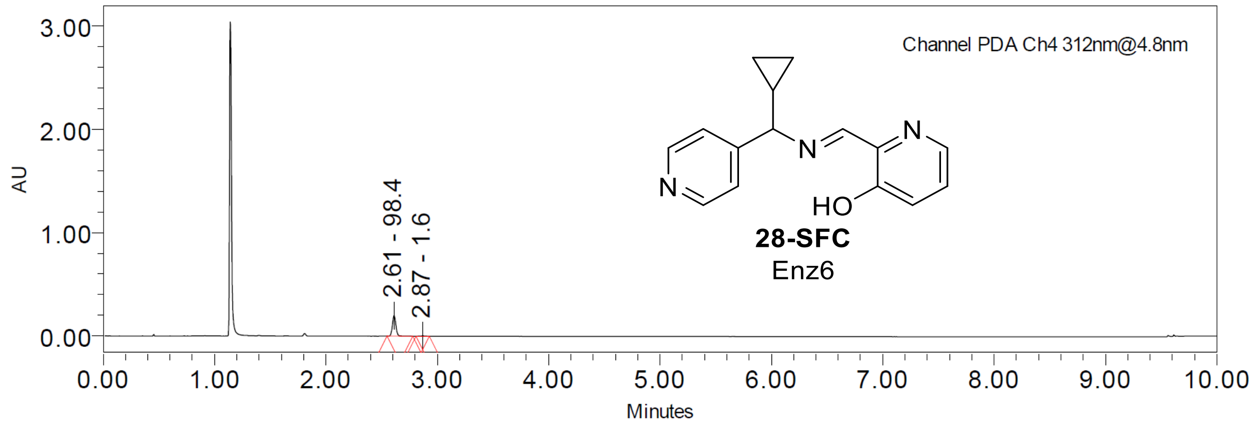
Channel PDA Ch4 312nm@4.8nm
AU
Minutes
2.61 - 98.4
2.87 - 1.6
N
N
N
HO
28-SFC
Enz6

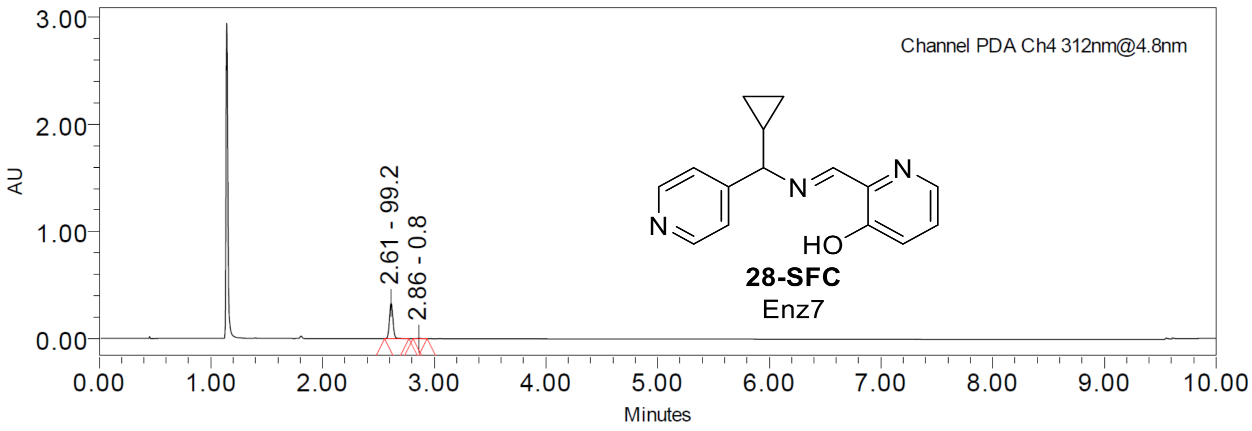

Channel PDA Ch4 312nm@4.8nm
2.61 - 99.2
2.86 - 0.8
28-SFC
Enz7
AU
Minutes

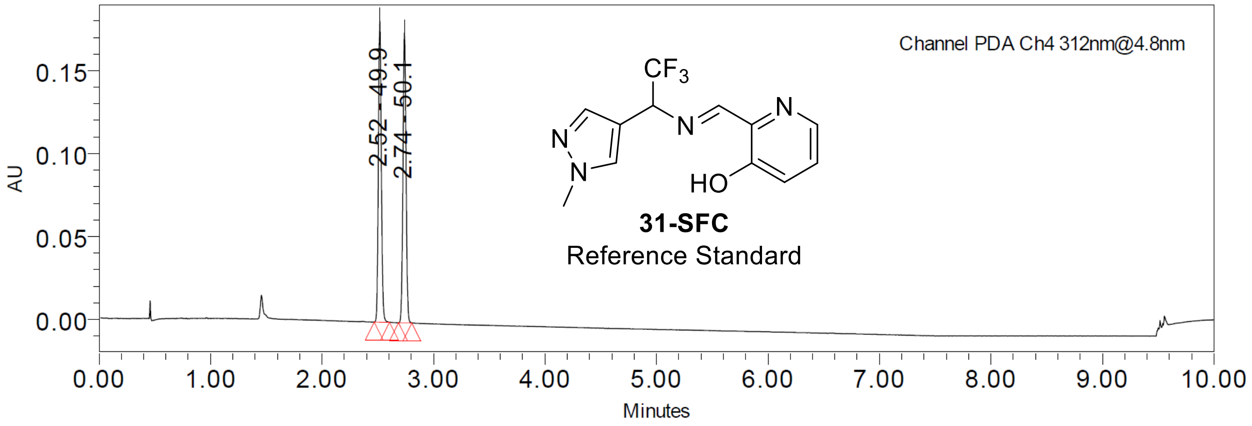

Channel PDA Ch4 312nm@4.8nm
2.52 - 49.9
2.74 - 50.1
31-SFC
Reference Standard
AU
Minutes

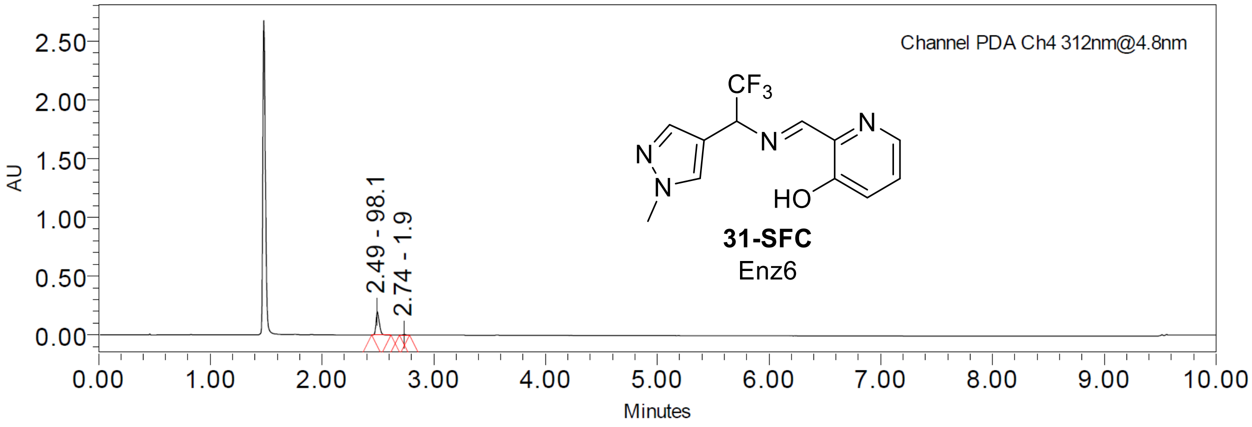

Channel PDA Ch4 312nm@4.8nm
2.49 - 98.1
2.74 - 1.9
31-SFC
Enz6
AU
Minutes

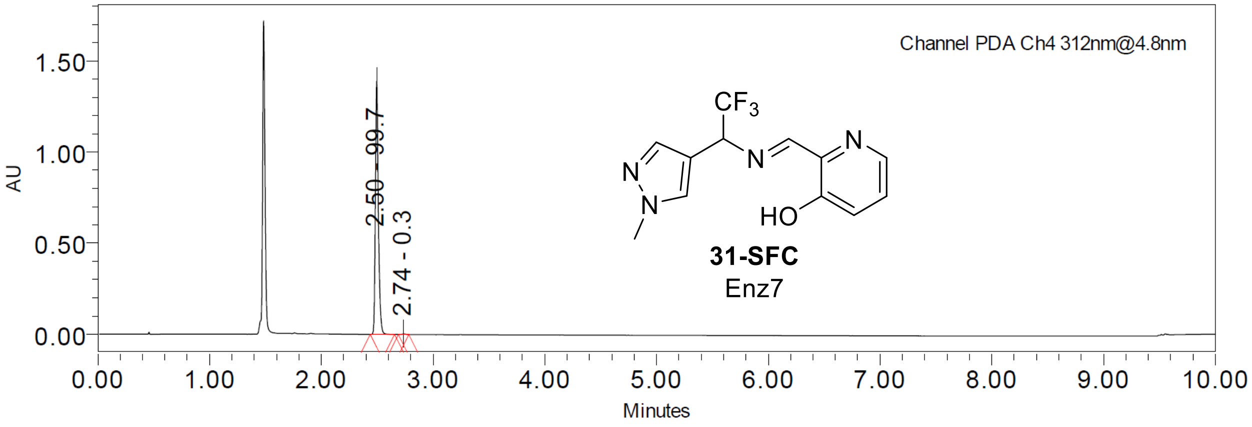

Channel PDA Ch4 312nm@4.8nm
AU
Minutes
2.50 - 99.7
2.74 - 0.3
CF3
N
N
N
HO
N
31-SFC
Enz7

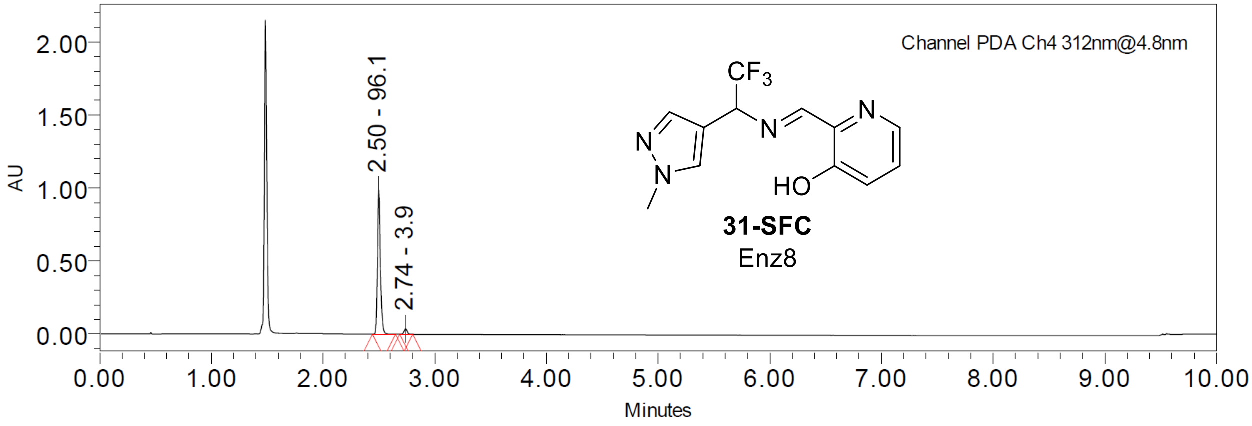

Channel PDA Ch4 312nm@4.8nm
AU
Minutes
2.50 - 96.1
2.74 - 3.9
CF3
31-SFC
Enz8

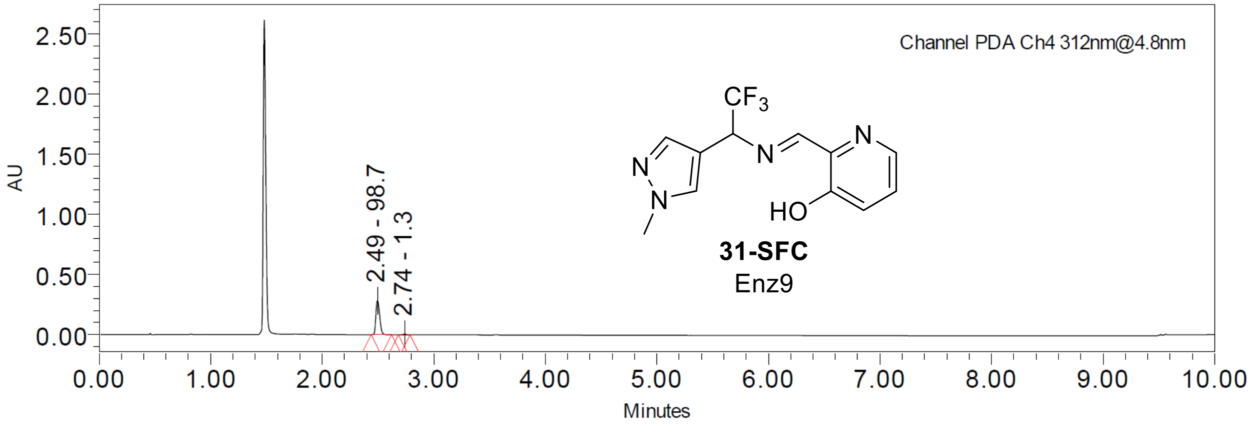

Channel PDA Ch4 312nm@4.8nm
AU
Minutes
2.49 - 98.7
2.74 - 1.3
CF3
31-SFC
Enz9

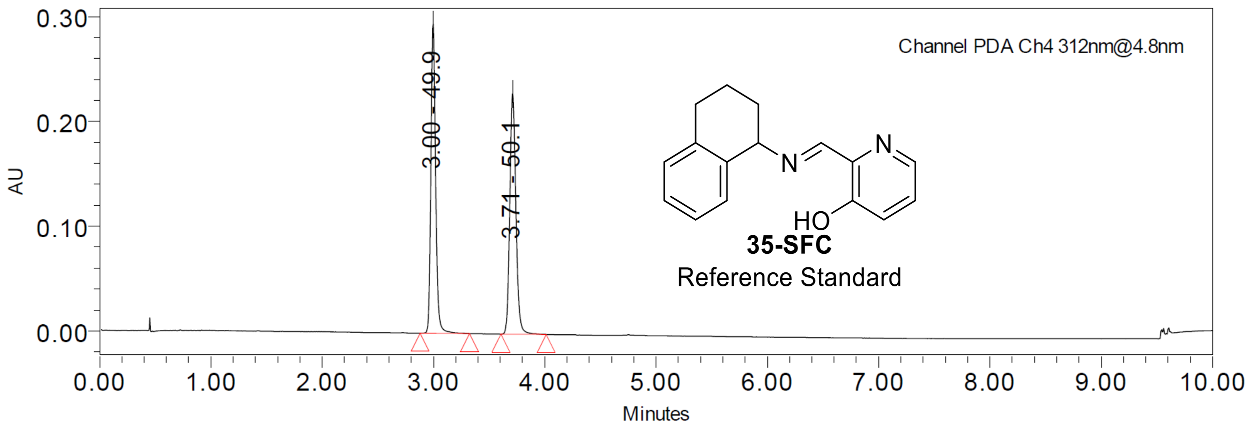

Channel PDA Ch4 312nm@4.8nm
AU
Minutes
3.00 - 49.9
3.71 - 50.1
HO
N
N
35-SFC
Reference Standard

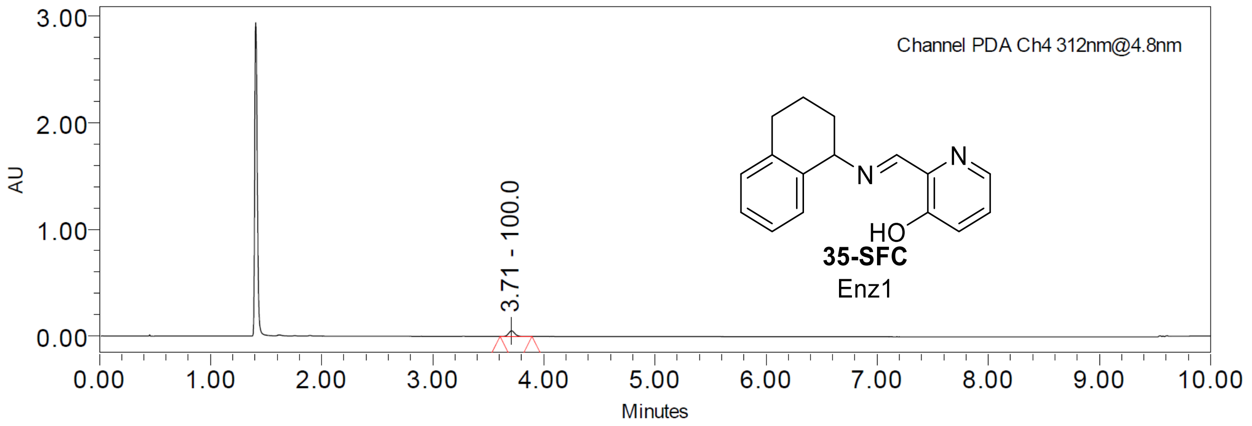

Channel PDA Ch4 312nm@4.8nm
AU
Minutes
3.71 - 100.0
HO
N
N
35-SFC
Enz1

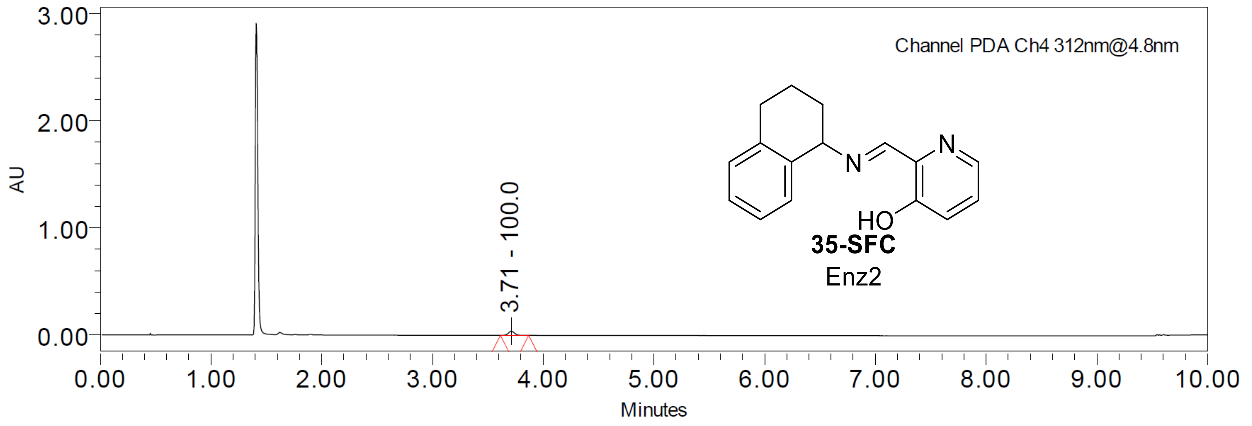

Channel PDA Ch4 312nm@4.8nm
AU
Minutes
3.71 - 100.0
HO
N
N
35-SFC
Enz2

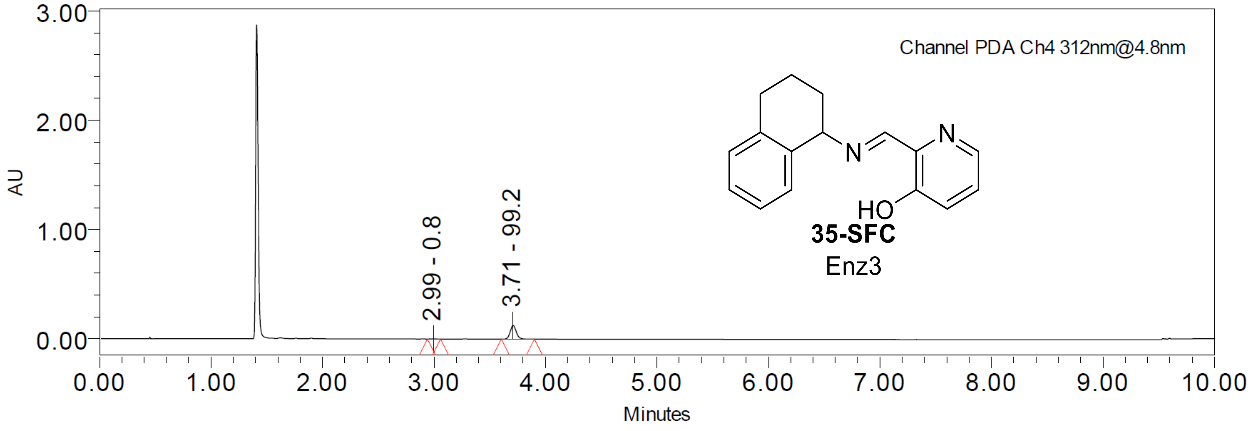

Channel PDA Ch4 312nm@4.8nm
AU
Minutes
2.99 - 0.8
3.71 - 99.2
N
N
HO
35-SFC
Enz3

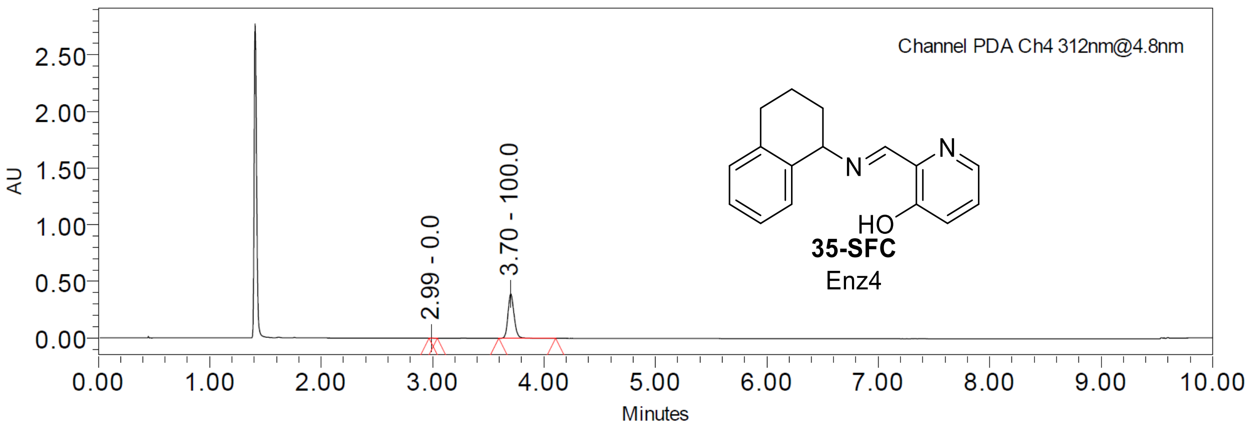

Channel PDA Ch4 312nm@4.8nm
AU
Minutes
2.99 - 0.0
3.70 - 100.0
N
N
HO
35-SFC
Enz4

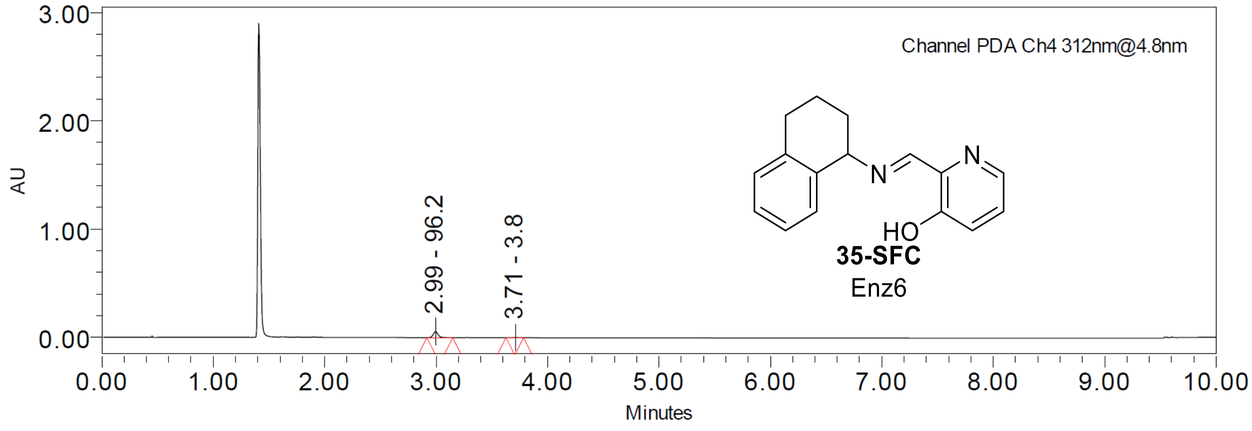

Channel PDA Ch4 312nm@4.8nm
AU
Minutes
2.99 - 96.2
3.71 - 3.8
N
N
HO
35-SFC
Enz6

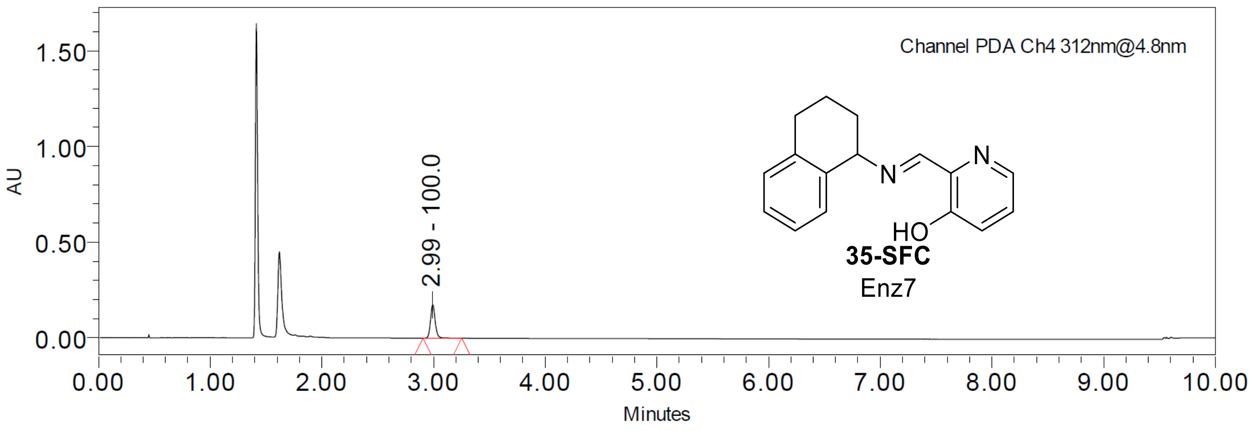
Channel PDA Ch4 312nm@4.8nm
AU
1.50
1.00
0.50
0.00
2.99 - 100.0
N
N
HO
35-SFC
Enz7
0.00 1.00 2.00 3.00 4.00 5.00 6.00 7.00 8.00 9.00 10.00
Minutes

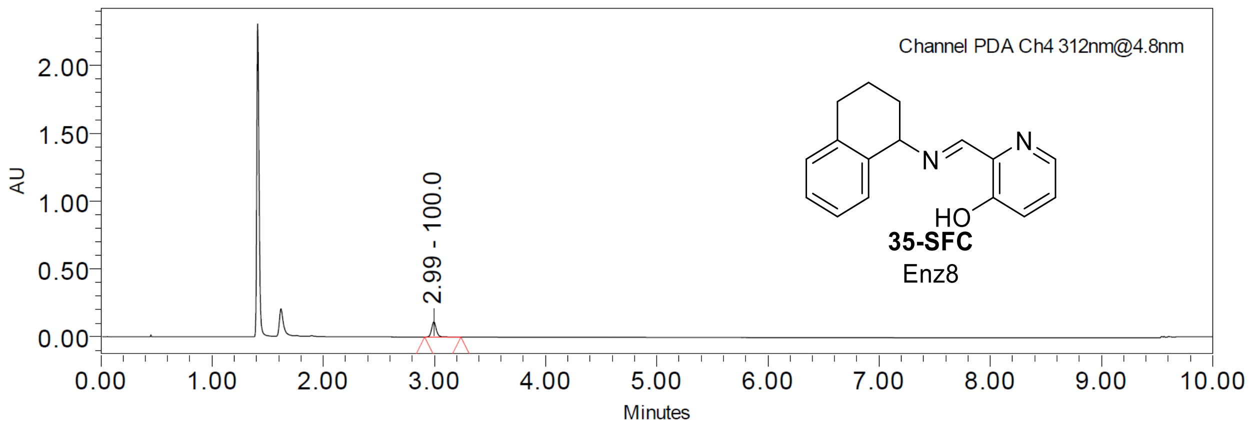
Channel PDA Ch4 312nm@4.8nm
AU
2.00
1.50
1.00
0.50
0.00
2.99 - 100.0
N
N
HO
35-SFC
Enz8
0.00 1.00 2.00 3.00 4.00 5.00 6.00 7.00 8.00 9.00 10.00
Minutes

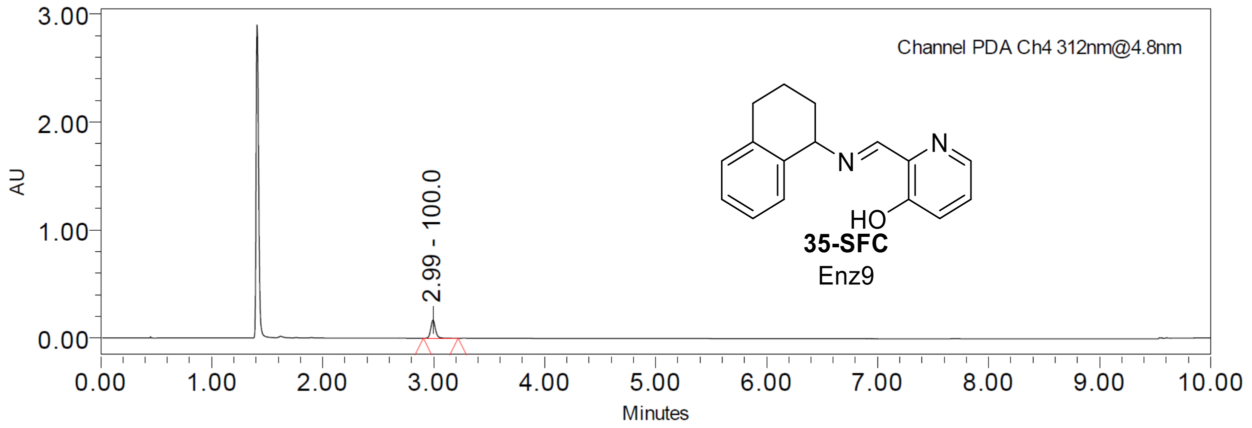
Channel PDA Ch4 312nm@4.8nm
AU
3.00
2.00
1.00
0.00
2.99 - 100.0
N
N
HO
35-SFC
Enz9
0.00 1.00 2.00 3.00 4.00 5.00 6.00 7.00 8.00 9.00 10.00
Minutes

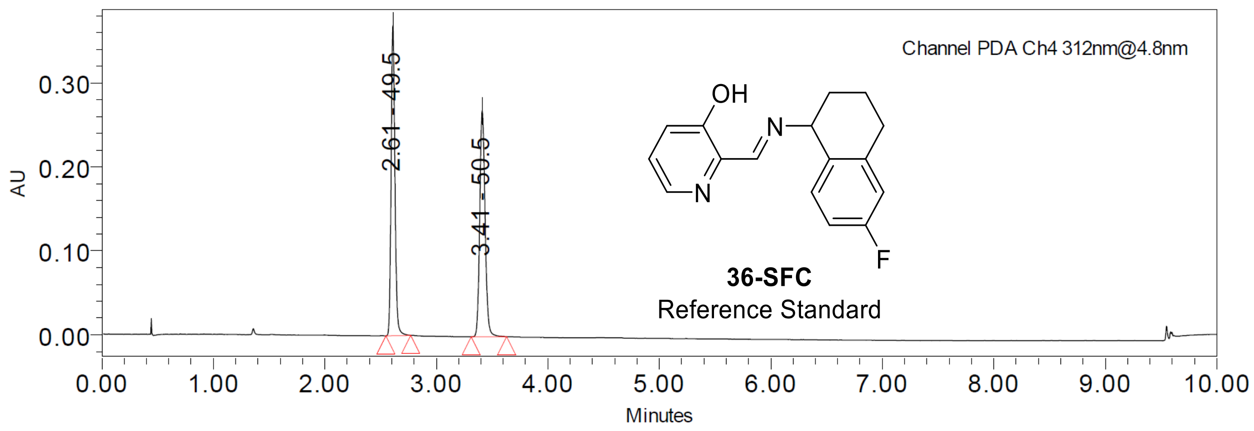
Channel PDA Ch4 312nm@4.8nm
2.61 - 49.5
3.41 - 50.5
OH
N
N
F
36-SFC
Reference Standard
AU
Minutes

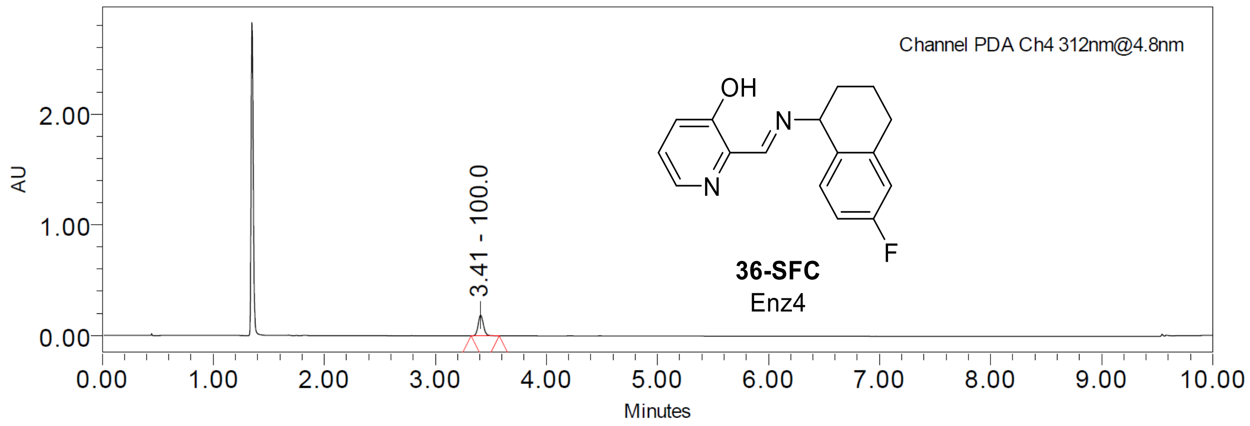
Channel PDA Ch4 312nm@4.8nm
3.41 - 100.0
OH
N
N
F
36-SFC
Enz4
AU
Minutes

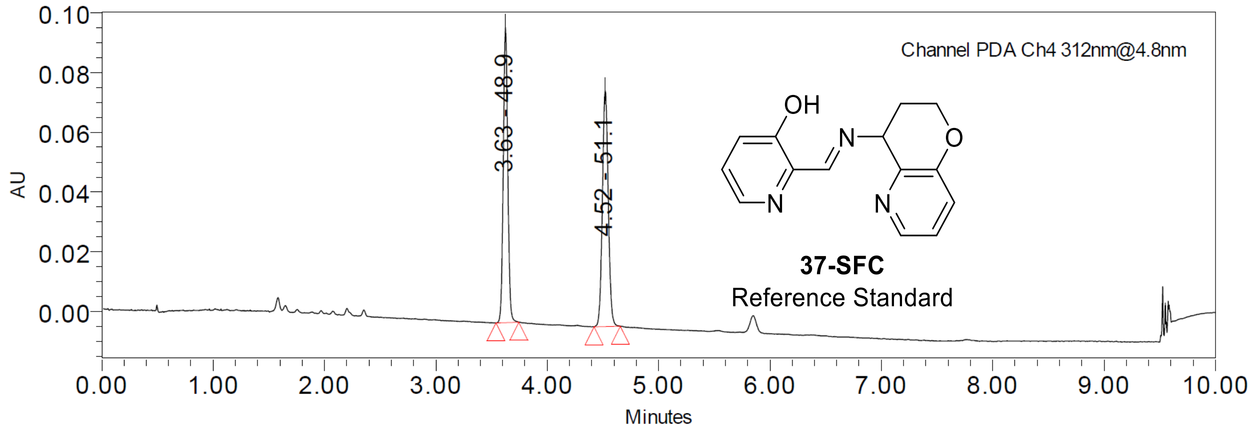
Channel PDA Ch4 312nm@4.8nm
3.63 - 48.9
4.52 - 51.1
OH
N
O
N
N
37-SFC
Reference Standard
AU
Minutes

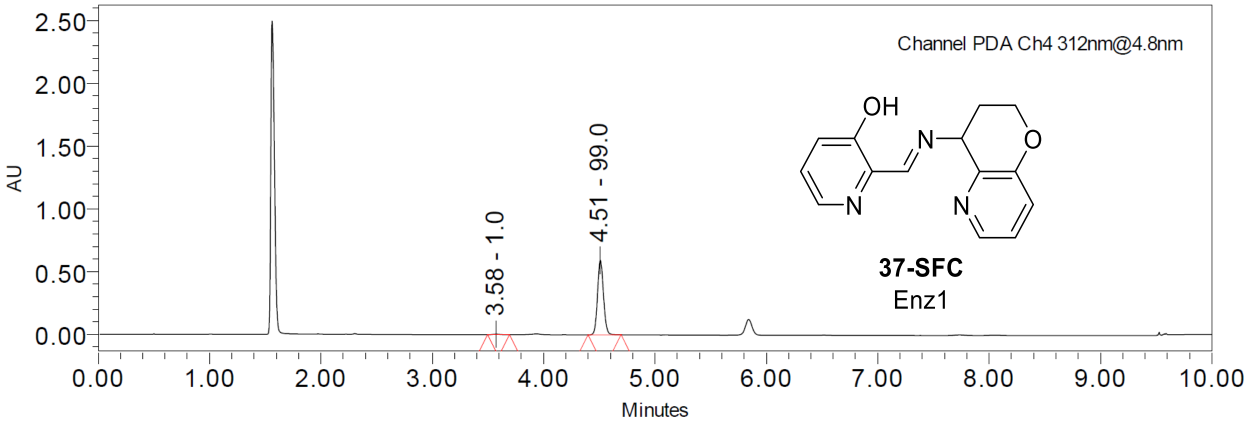
Channel PDA Ch4 312nm@4.8nm
OH
N
O
N
N
37-SFC
Enz1
3.58 - 1.0
4.51 - 99.0
AU
Minutes
2.50
2.00
1.50
1.00
0.50
0.00
0.00 1.00 2.00 3.00 4.00 5.00 6.00 7.00 8.00 9.00 10.00

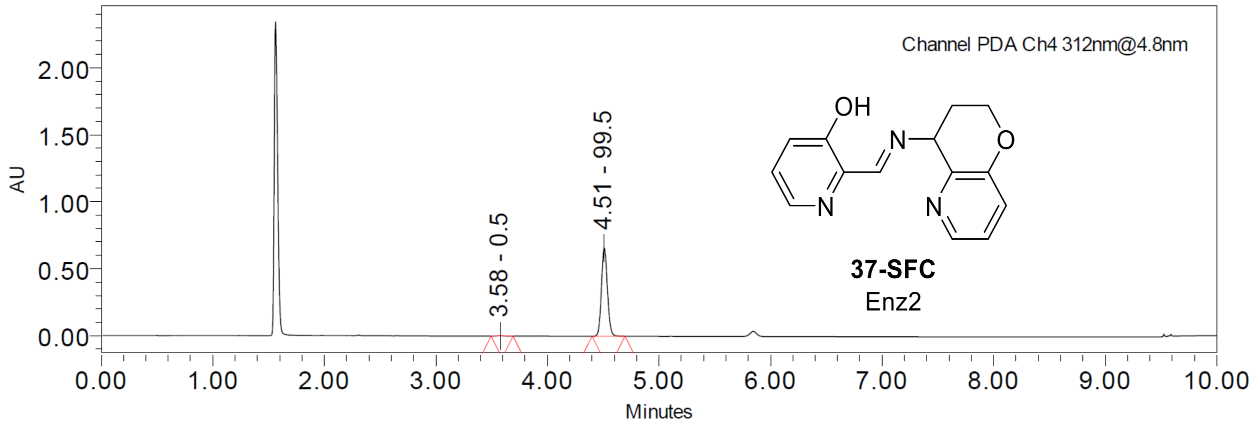
Channel PDA Ch4 312nm@4.8nm
OH
N
O
N
N
37-SFC
Enz2
3.58 - 0.5
4.51 - 99.5
AU
Minutes
2.00
1.50
1.00
0.50
0.00
0.00 1.00 2.00 3.00 4.00 5.00 6.00 7.00 8.00 9.00 10.00

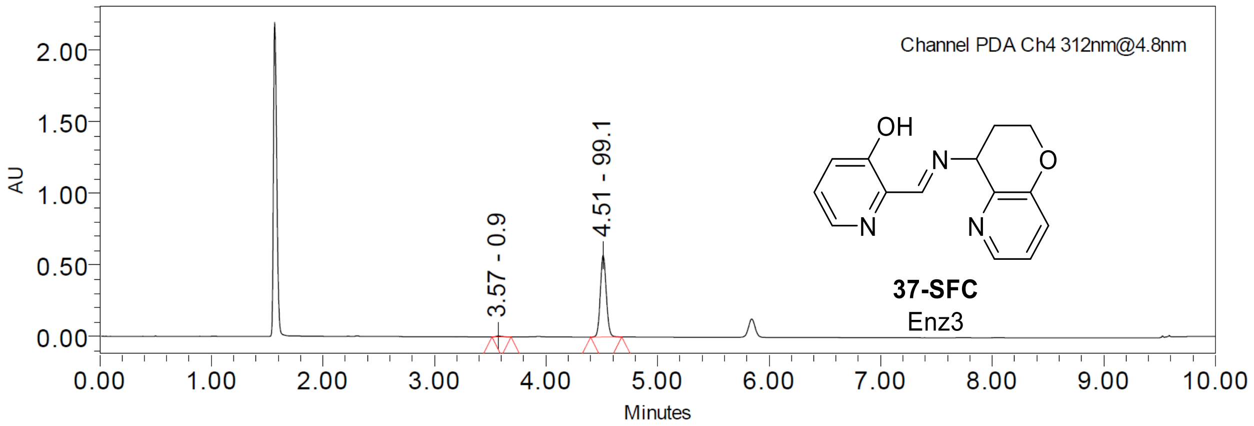
Channel PDA Ch4 312nm@4.8nm
AU
Minutes
3.57 - 0.9
4.51 - 99.1
OH
N
O
37-SFC
Enz3

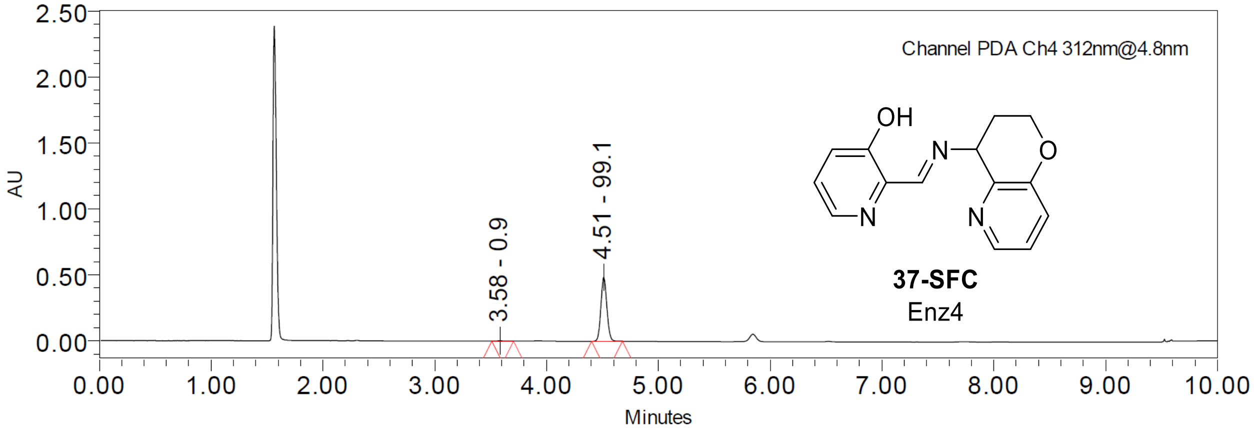
Channel PDA Ch4 312nm@4.8nm
AU
Minutes
3.58 - 0.9
4.51 - 99.1
OH
N
O
37-SFC
Enz4

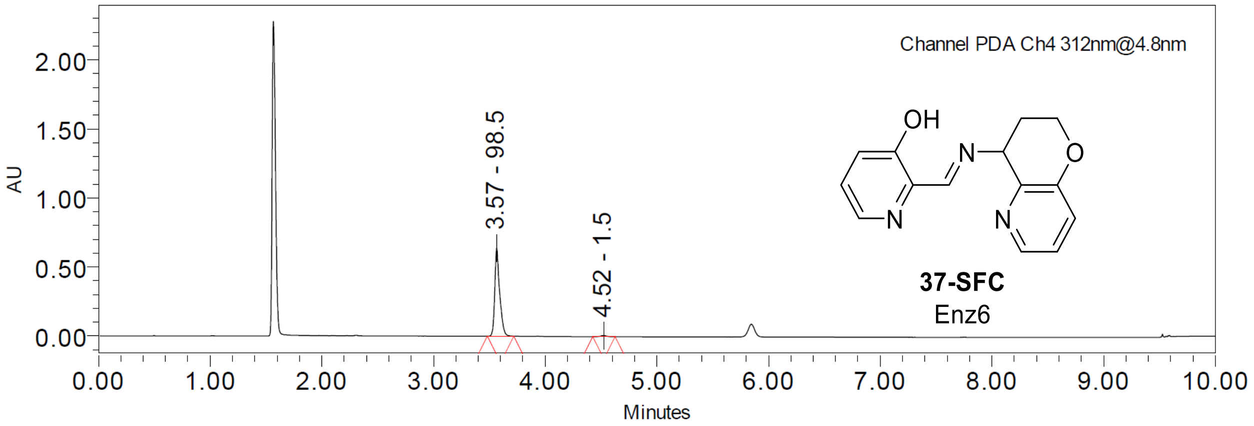
Channel PDA Ch4 312nm@4.8nm
AU
Minutes
3.57 - 98.5
4.52 - 1.5
OH
N
O
37-SFC
Enz6

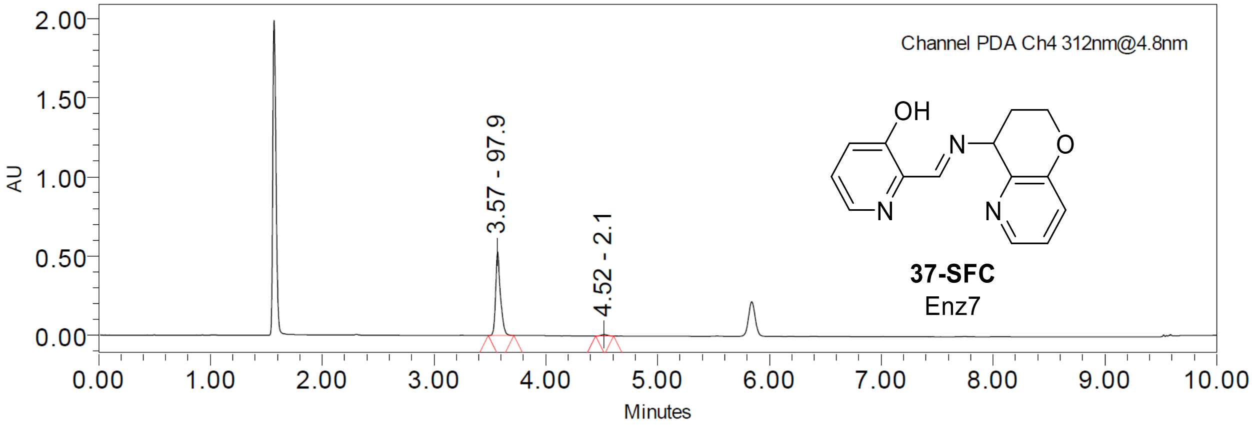

Channel PDA Ch4 312nm@4.8nm
OH
N
O
N
N
37-SFC
Enz7
3.57 - 97.9
4.52 - 2.1
AU
Minutes

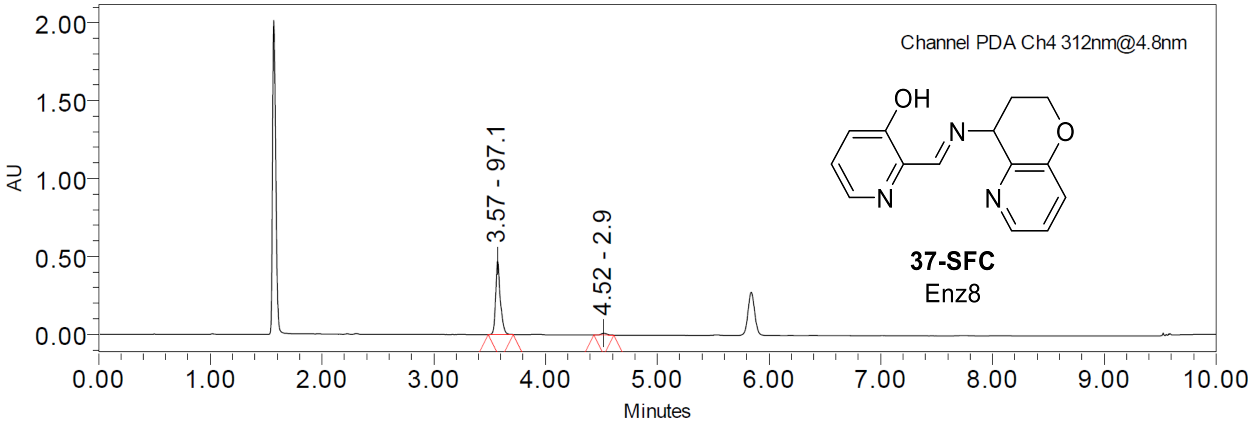

Channel PDA Ch4 312nm@4.8nm
OH
N
O
N
N
37-SFC
Enz8
3.57 - 97.1
4.52 - 2.9
AU
Minutes

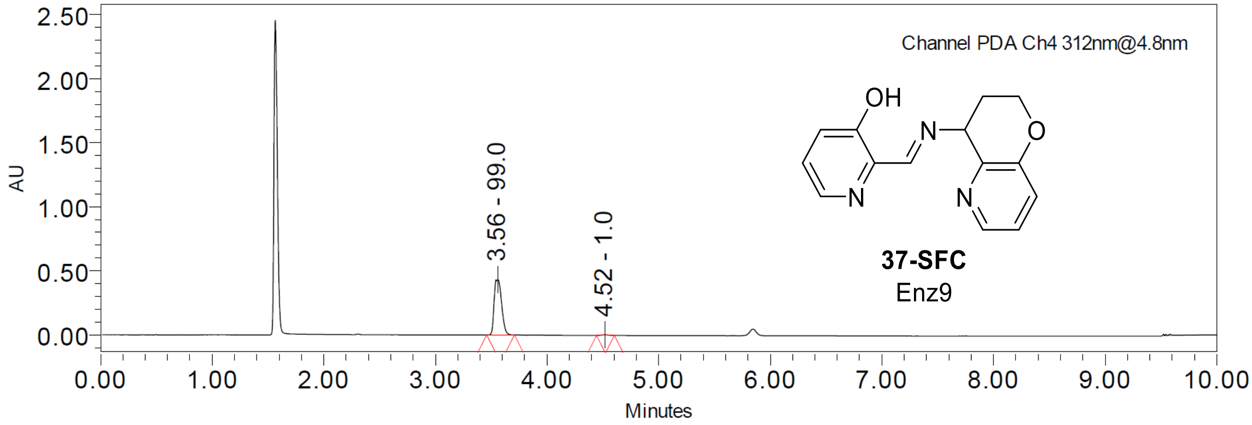

Channel PDA Ch4 312nm@4.8nm
OH
N
O
N
N
37-SFC
Enz9
3.56 - 99.0
4.52 - 1.0
AU
Minutes

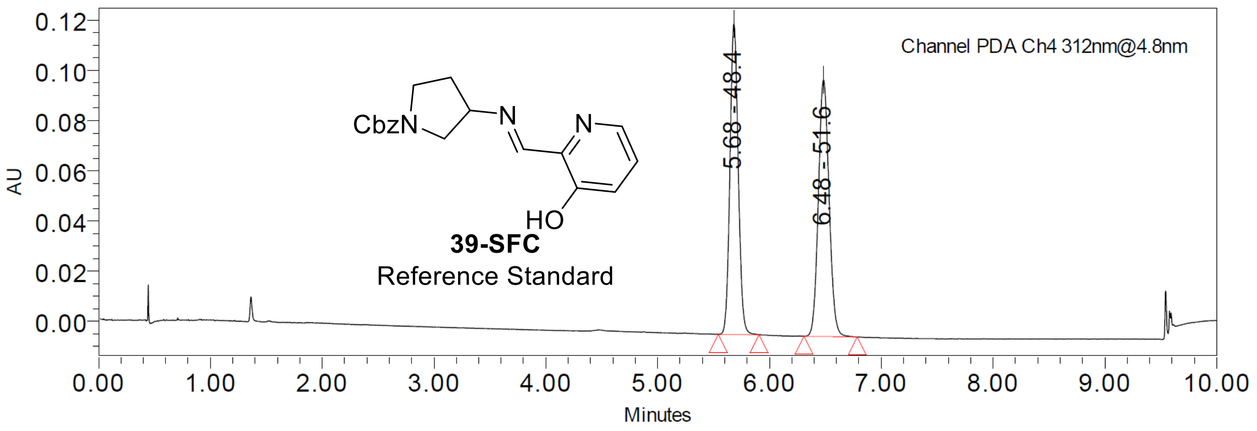
Channel PDA Ch4 312nm@4.8nm
CbzN
N
N
HO
39-SFC
Reference Standard
5.68 - 48.4
6.48 - 51.6
AU
Minutes

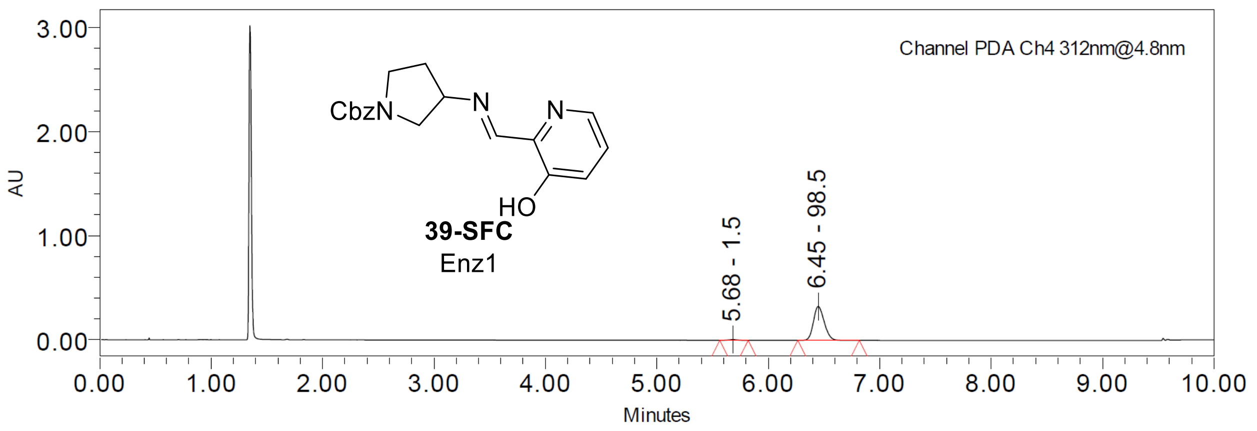
Channel PDA Ch4 312nm@4.8nm
CbzN
N
N
HO
39-SFC
Enz1
5.68 - 1.5
6.45 - 98.5
AU
Minutes

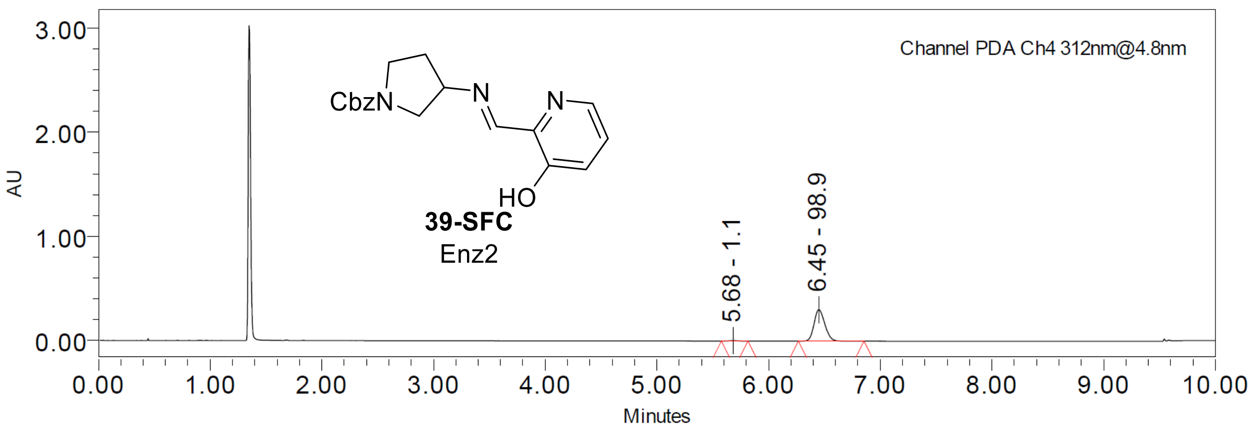
Channel PDA Ch4 312nm@4.8nm
CbzN
N
N
HO
39-SFC
Enz2
5.68 - 1.1
6.45 - 98.9
AU
Minutes

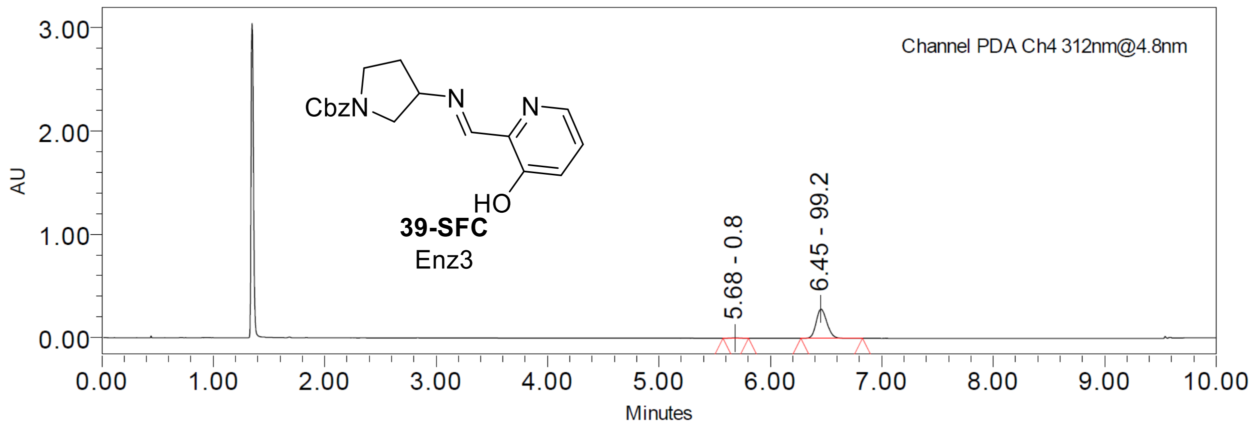

3.00
2.00
1.00
0.00
AU
Channel PDA Ch4 312nm@4.8nm
CbzN
N
N
HO
39-SFC
Enz3
5.68 - 0.8
6.45 - 99.2
0.00
1.00
2.00
3.00
4.00
5.00
6.00
7.00
8.00
9.00
10.00
Minutes

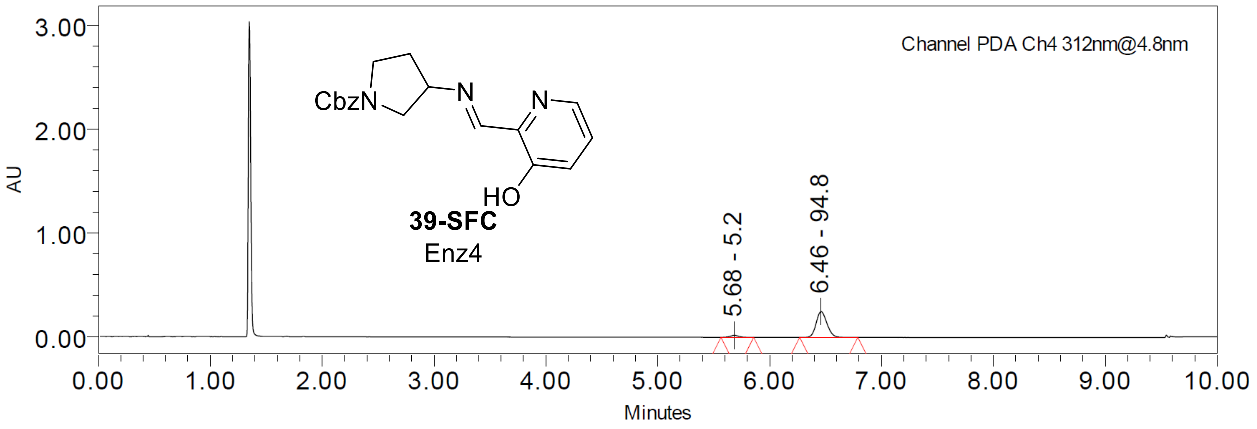

3.00
2.00
1.00
0.00
AU
Channel PDA Ch4 312nm@4.8nm
CbzN
N
N
HO
39-SFC
Enz4
5.68 - 5.2
6.46 - 94.8
0.00
1.00
2.00
3.00
4.00
5.00
6.00
7.00
8.00
9.00
10.00
Minutes

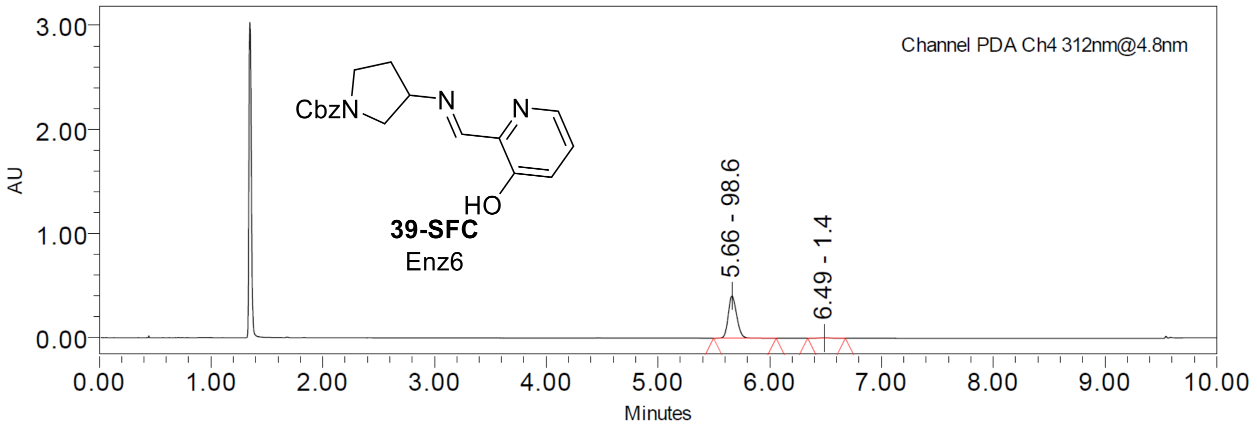

3.00
2.00
1.00
0.00
AU
Channel PDA Ch4 312nm@4.8nm
CbzN
N
N
HO
39-SFC
Enz6
5.66 - 98.6
6.49 - 1.4
0.00
1.00
2.00
3.00
4.00
5.00
6.00
7.00
8.00
9.00
10.00
Minutes

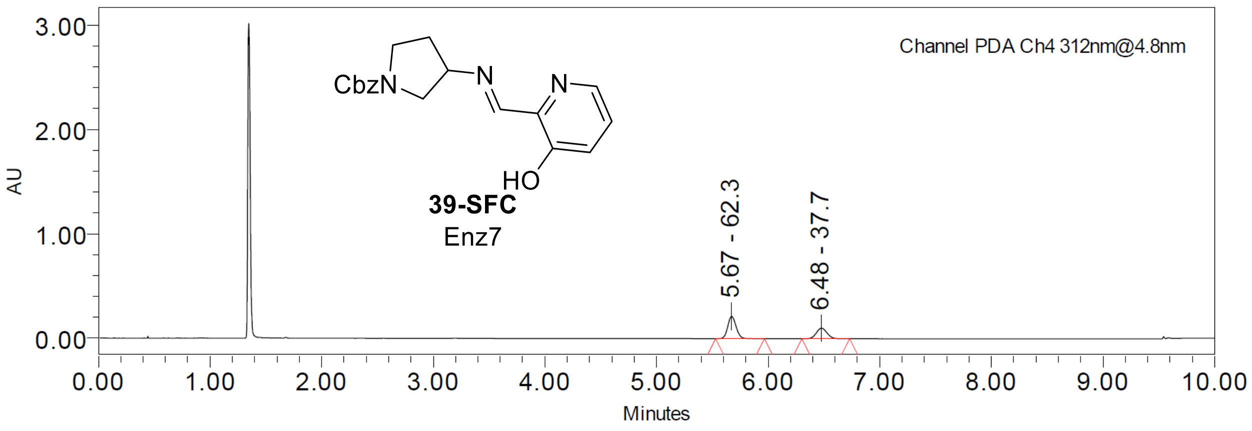
Channel PDA Ch4 312nm@4.8nm
CbzN
N
N
HO
39-SFC
Enz7
5.67 - 62.3
6.48 - 37.7
AU
Minutes

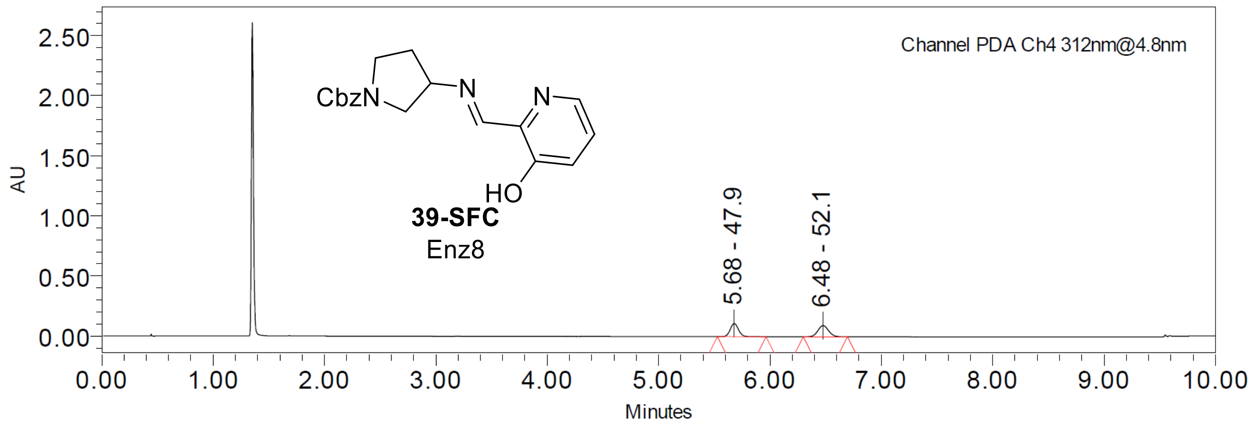
Channel PDA Ch4 312nm@4.8nm
CbzN
N
N
HO
39-SFC
Enz8
5.68 - 47.9
6.48 - 52.1
AU
Minutes

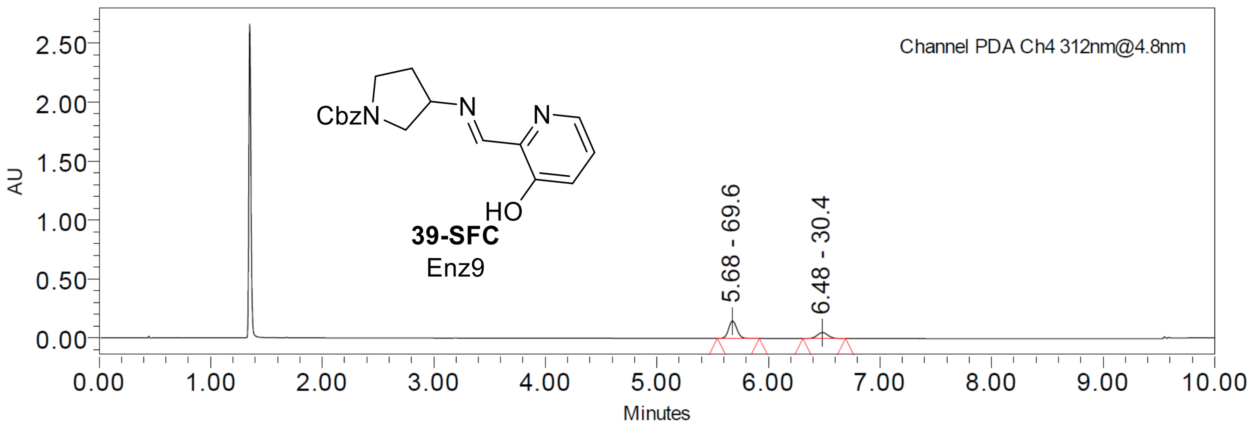
Channel PDA Ch4 312nm@4.8nm
CbzN
N
N
HO
39-SFC
Enz9
5.68 - 69.6
6.48 - 30.4
AU
Minutes

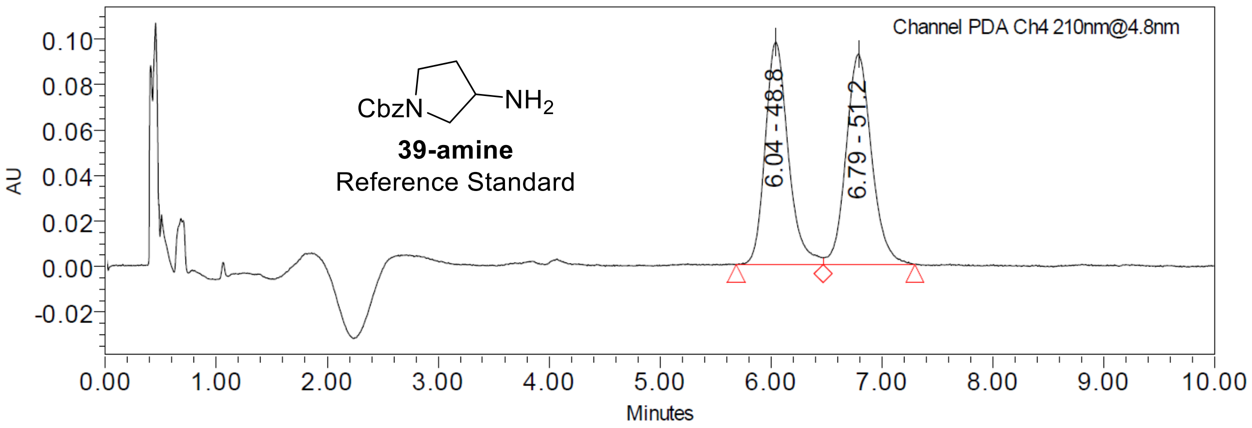

Note: column used was ChiralCel OZ-3, cosolvent was Ethanol 190 0.1% diethylamine, and isocratic gradient of 90% CO2/10% cosolvent was used.

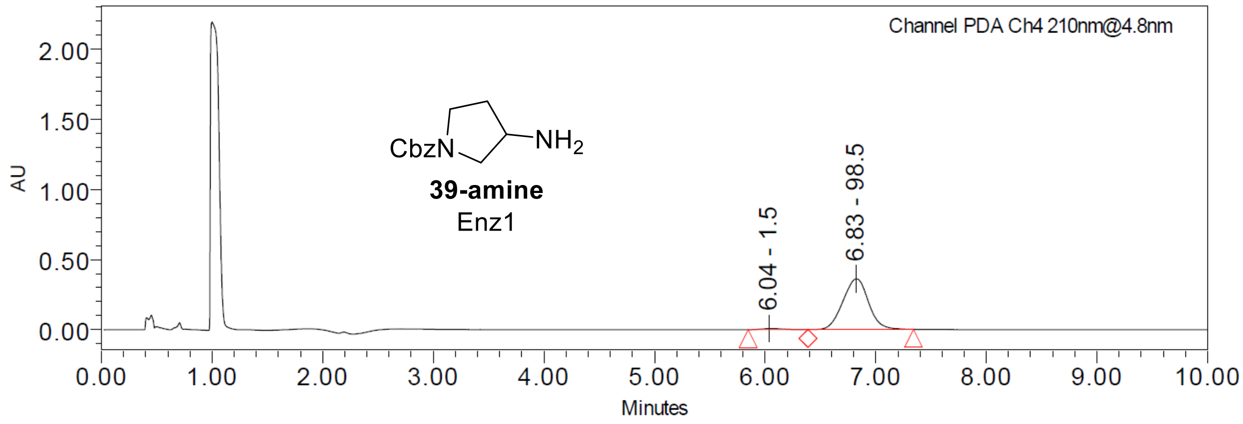

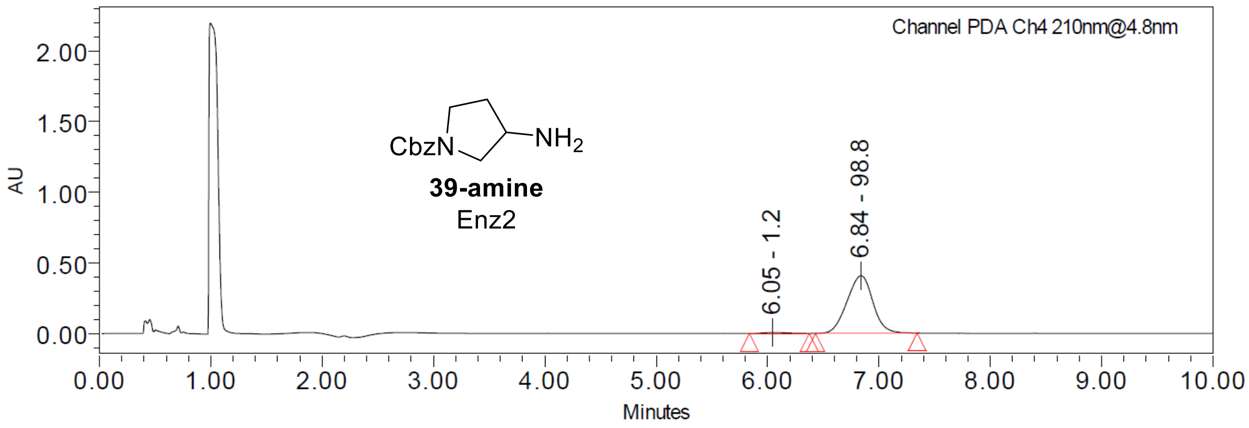

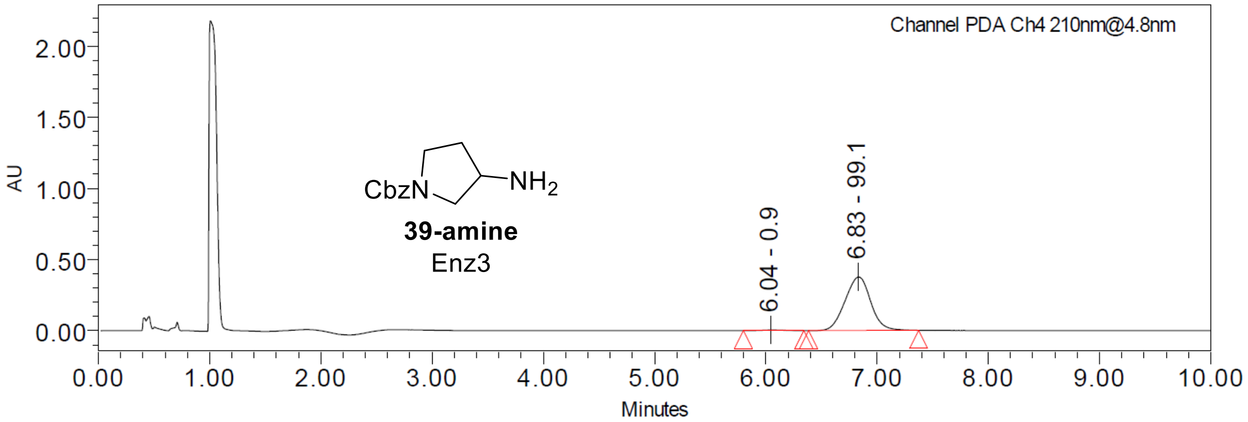
Channel PDA Ch4 210nm@4.8nm
CbzN
NH2
39-amine
Enz3
6.04 - 0.9
6.83 - 99.1
AU
Minutes

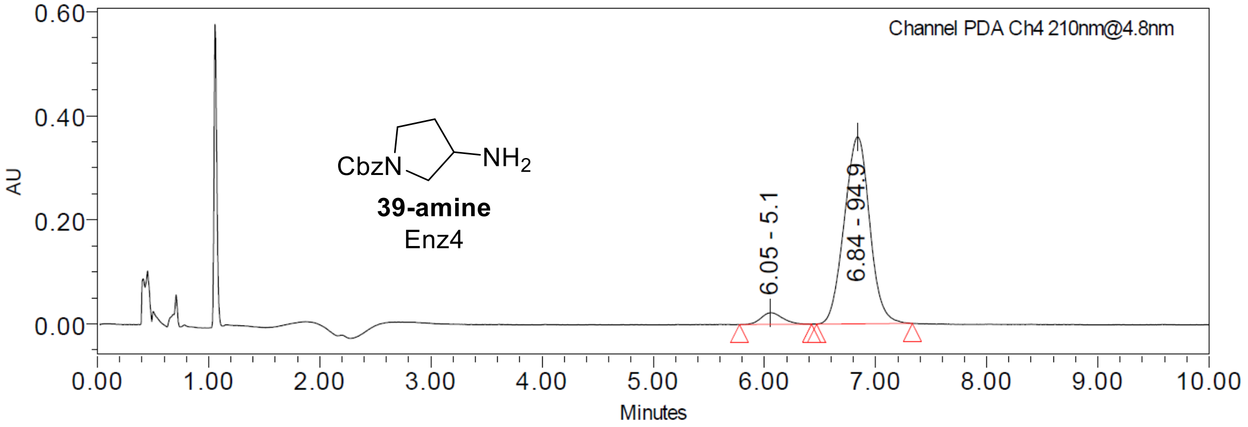
Channel PDA Ch4 210nm@4.8nm
CbzN
NH2
39-amine
Enz4
6.05 - 5.1
6.84 - 94.9
AU
Minutes

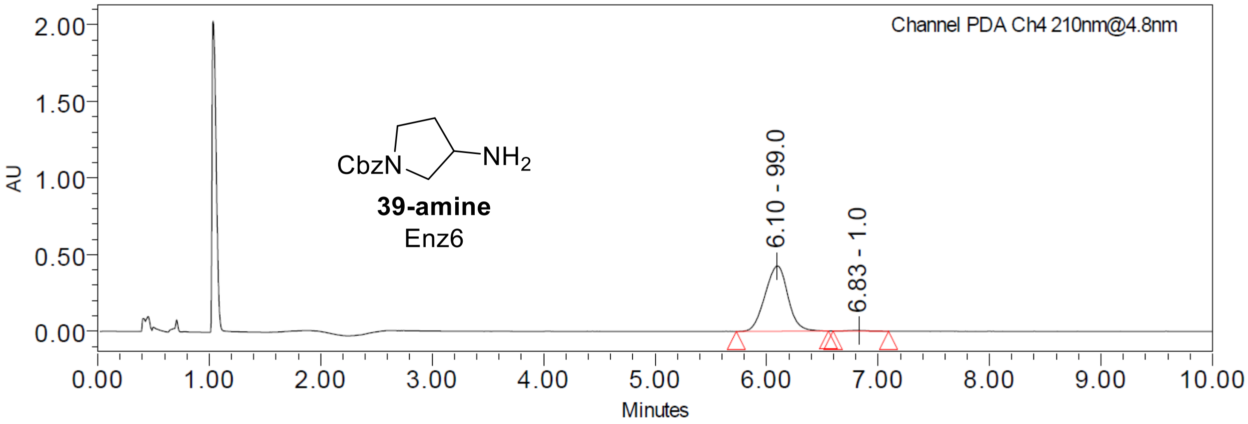
Channel PDA Ch4 210nm@4.8nm
CbzN
NH2
39-amine
Enz6
6.10 - 99.0
6.83 - 1.0
AU
Minutes

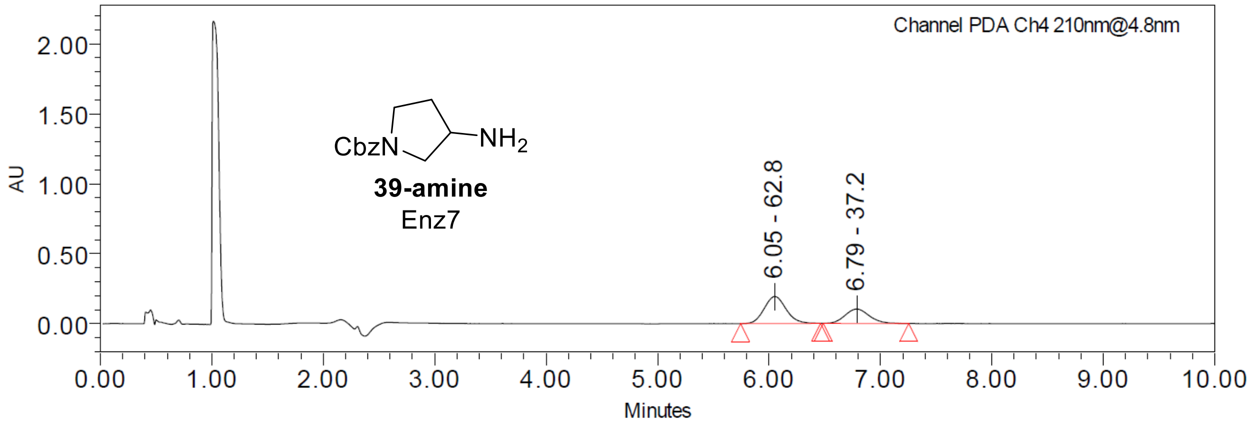
Channel PDA Ch4 210nm@4.8nm
CbzN
NH2
39-amine
Enz7
6.05 - 62.8
6.79 - 37.2
AU
Minutes

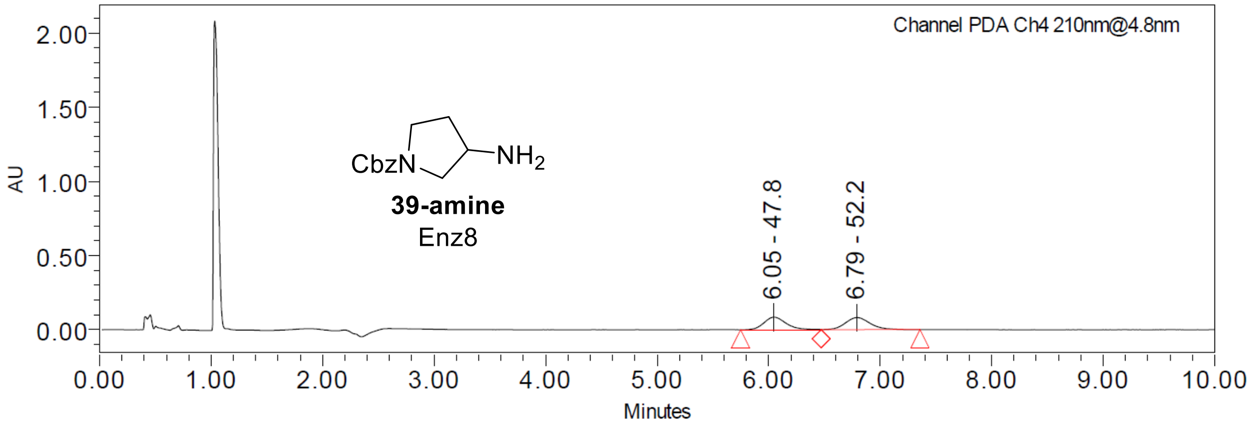
Channel PDA Ch4 210nm@4.8nm
CbzN
NH2
39-amine
Enz8
6.05 - 47.8
6.79 - 52.2
AU
Minutes

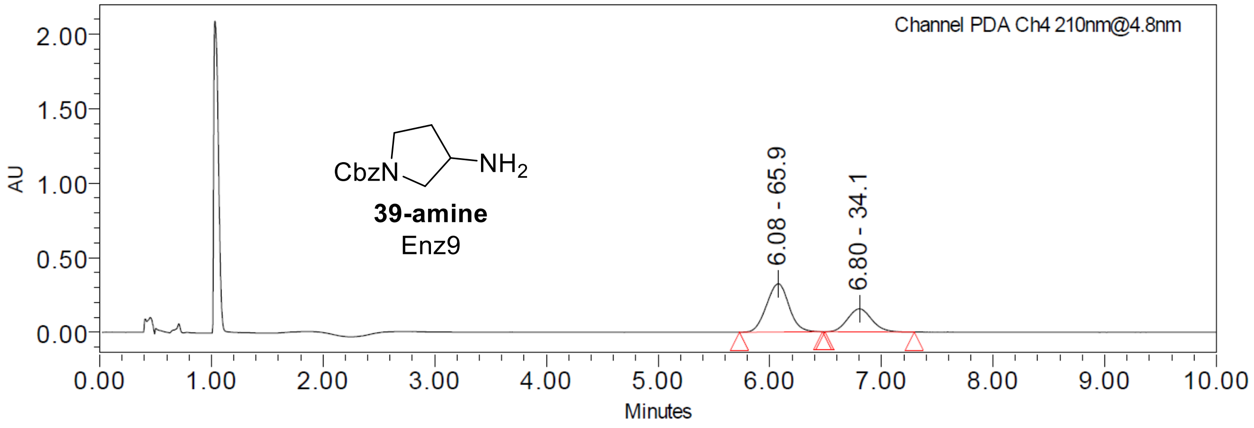
Channel PDA Ch4 210nm@4.8nm
CbzN
NH2
39-amine
Enz9
6.08 - 65.9
6.80 - 34.1
AU
Minutes

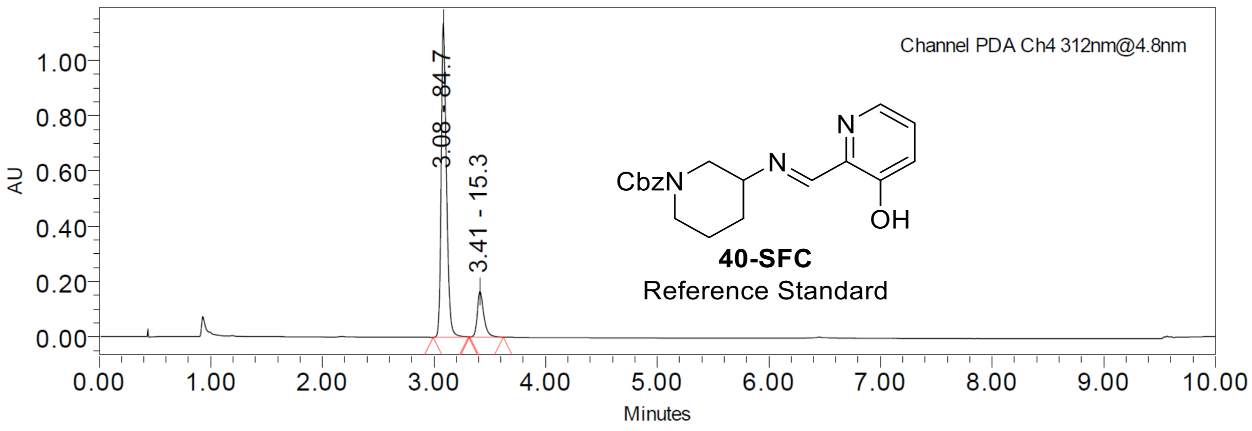

Note: This standard was prepared from commercially available amine precursor. Both peaks exhibit the expected MS signal of 340 *m/z*. The SFC traces from the enzymatic reactions show the expected difference in the major peak retention time between the (*S*)- and (*R*)-selective enzymes. Therefore, it appears that the material was not fully racemic.

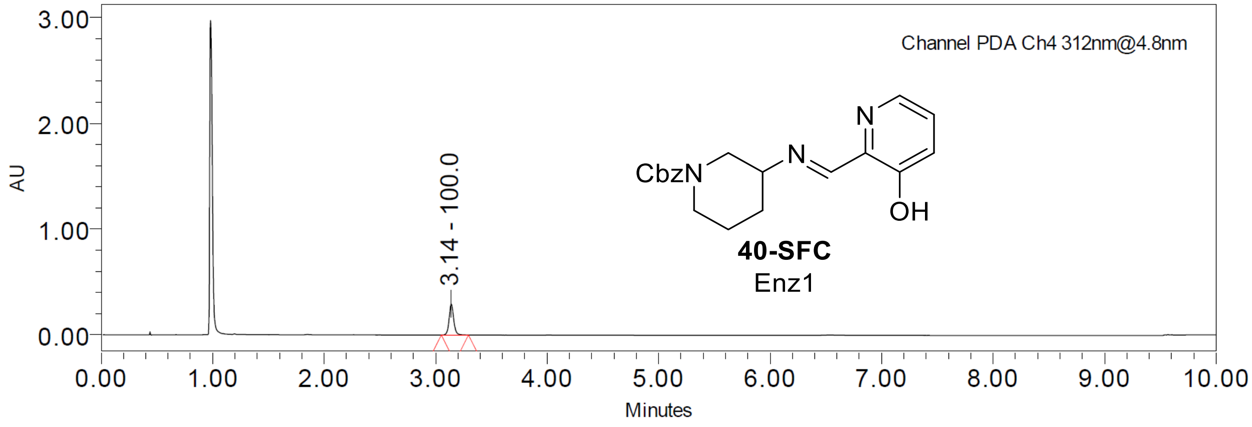

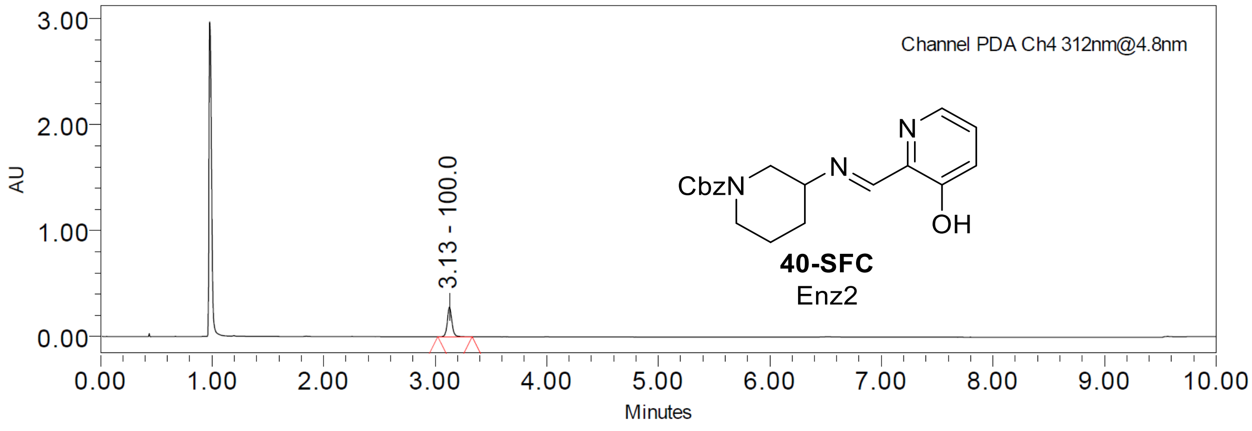

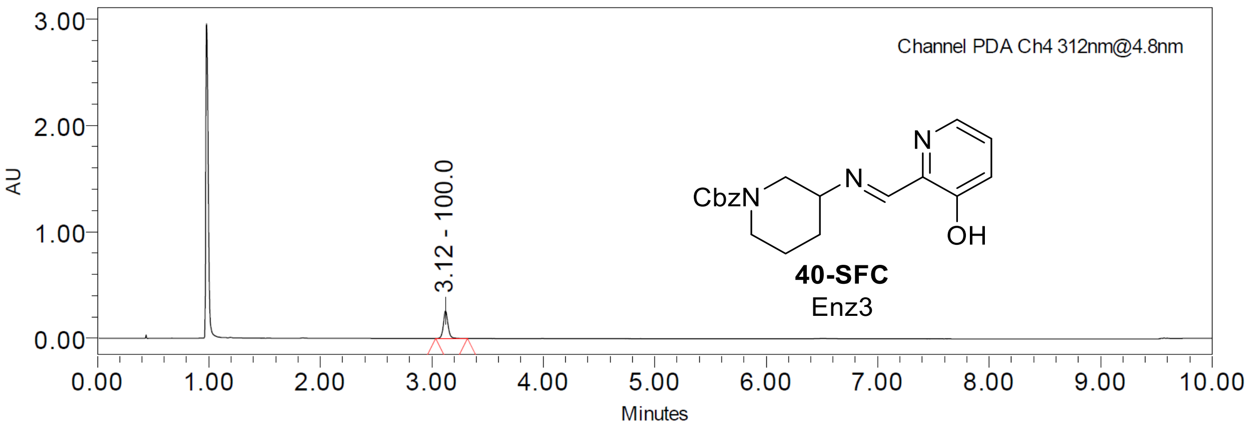

Channel PDA Ch4 312nm@4.8nm
AU
3.00
2.00
1.00
0.00
3.12 - 100.0
CbzN
N
N
OH
40-SFC
Enz3
0.00
1.00
2.00
3.00
4.00
5.00
6.00
7.00
8.00
9.00
10.00
Minutes

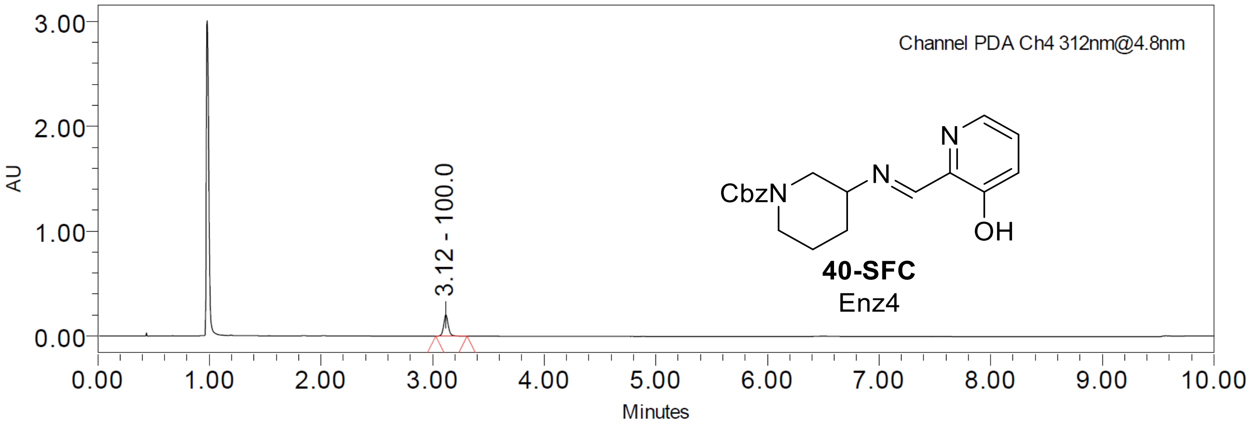

Channel PDA Ch4 312nm@4.8nm
AU
3.00
2.00
1.00
0.00
3.12 - 100.0
CbzN
N
N
OH
40-SFC
Enz4
0.00
1.00
2.00
3.00
4.00
5.00
6.00
7.00
8.00
9.00
10.00
Minutes

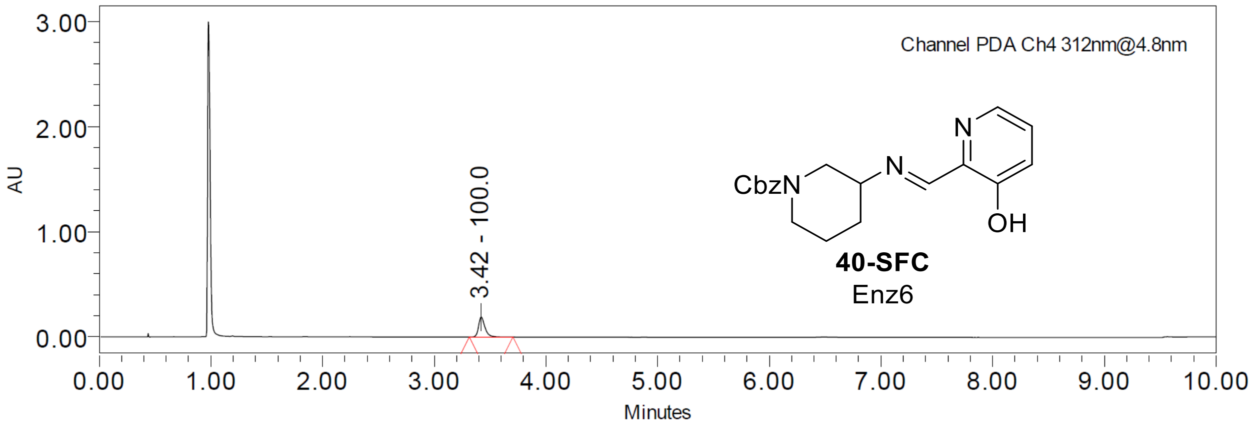

Channel PDA Ch4 312nm@4.8nm
AU
3.00
2.00
1.00
0.00
3.42 - 100.0
CbzN
N
N
OH
40-SFC
Enz6
0.00
1.00
2.00
3.00
4.00
5.00
6.00
7.00
8.00
9.00
10.00
Minutes

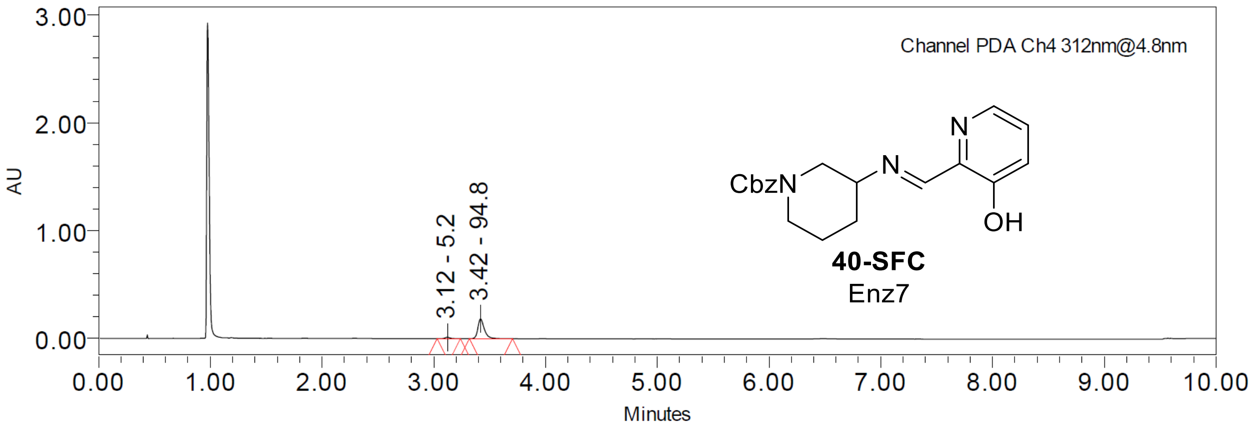
Channel PDA Ch4 312nm@4.8nm
CbzN
N
N
OH
40-SFC
Enz7
3.12 - 5.2
3.42 - 94.8
AU
Minutes

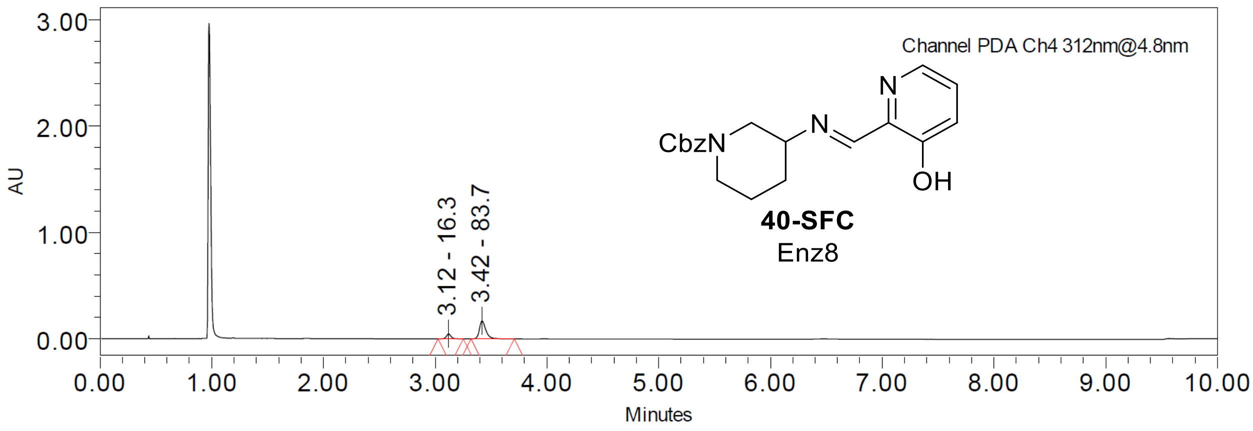
Channel PDA Ch4 312nm@4.8nm
CbzN
N
N
OH
40-SFC
Enz8
3.12 - 16.3
3.42 - 83.7
AU
Minutes

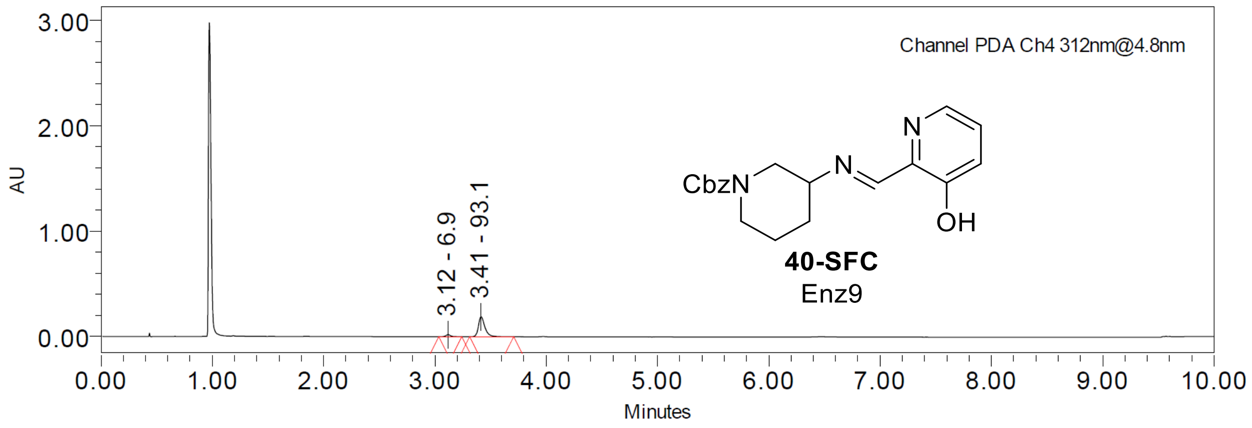
Channel PDA Ch4 312nm@4.8nm
CbzN
N
N
OH
40-SFC
Enz9
3.12 - 6.9
3.41 - 93.1
AU
Minutes

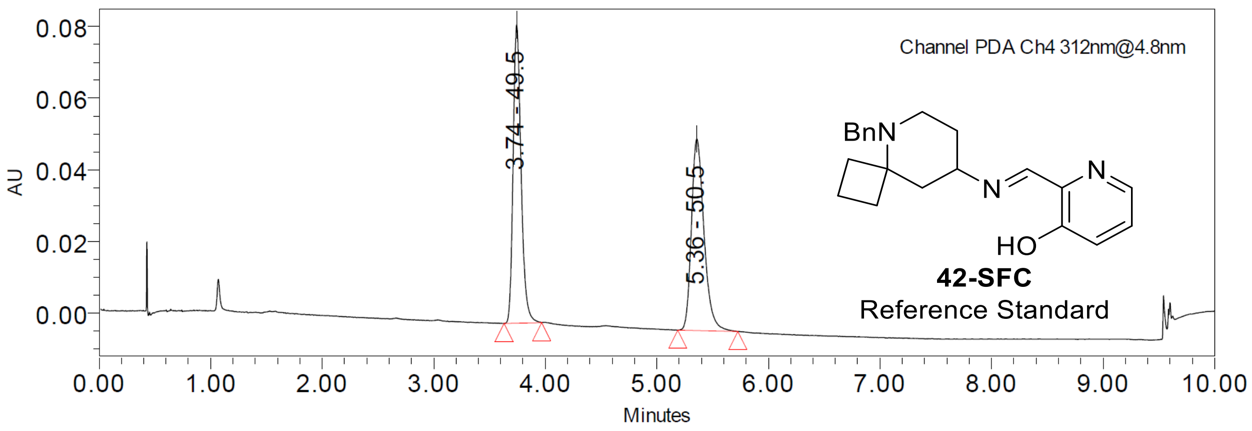

Channel PDA Ch4 312nm@4.8nm
AU
0.08
0.06
0.04
0.02
0.00
3.74 - 49.5
5.36 - 50.5
BnN
N
N
HO
42-SFC
Reference Standard
0.00
1.00
2.00
3.00
4.00
5.00
6.00
7.00
8.00
9.00
10.00
Minutes

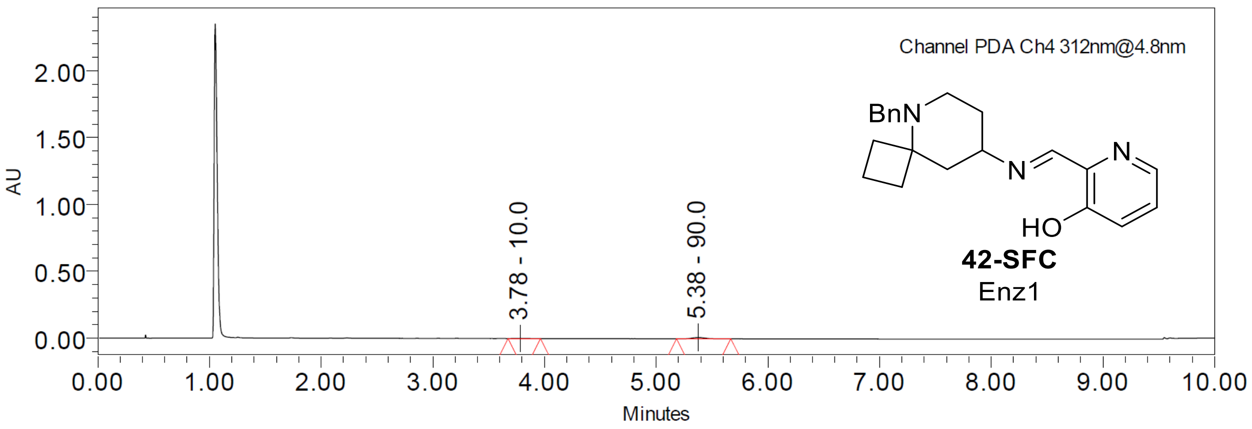

Channel PDA Ch4 312nm@4.8nm
AU
2.00
1.50
1.00
0.50
0.00
3.78 - 10.0
5.38 - 90.0
BnN
N
N
HO
42-SFC
Enz1
0.00
1.00
2.00
3.00
4.00
5.00
6.00
7.00
8.00
9.00
10.00
Minutes

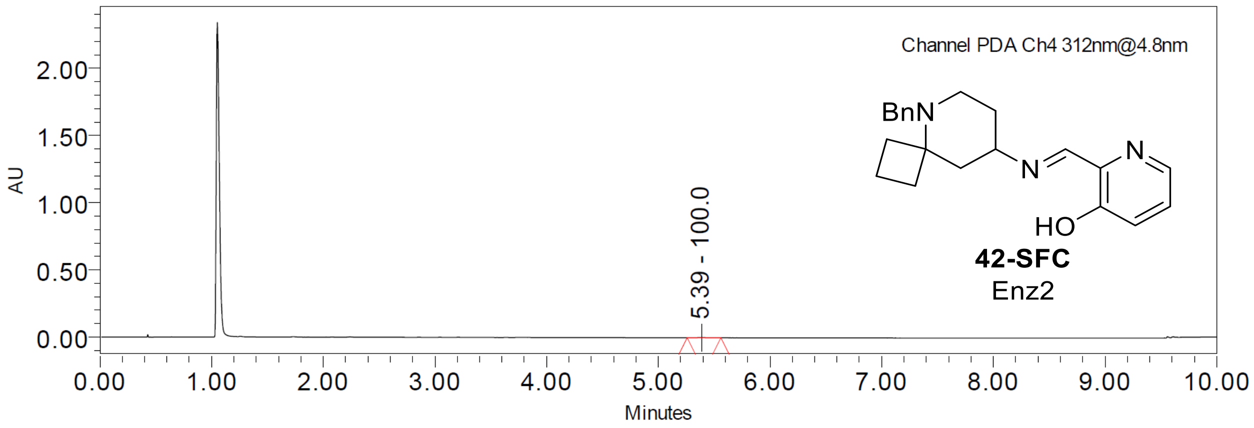

Channel PDA Ch4 312nm@4.8nm
AU
2.00
1.50
1.00
0.50
0.00
5.39 - 100.0
BnN
N
N
HO
42-SFC
Enz2
0.00
1.00
2.00
3.00
4.00
5.00
6.00
7.00
8.00
9.00
10.00
Minutes

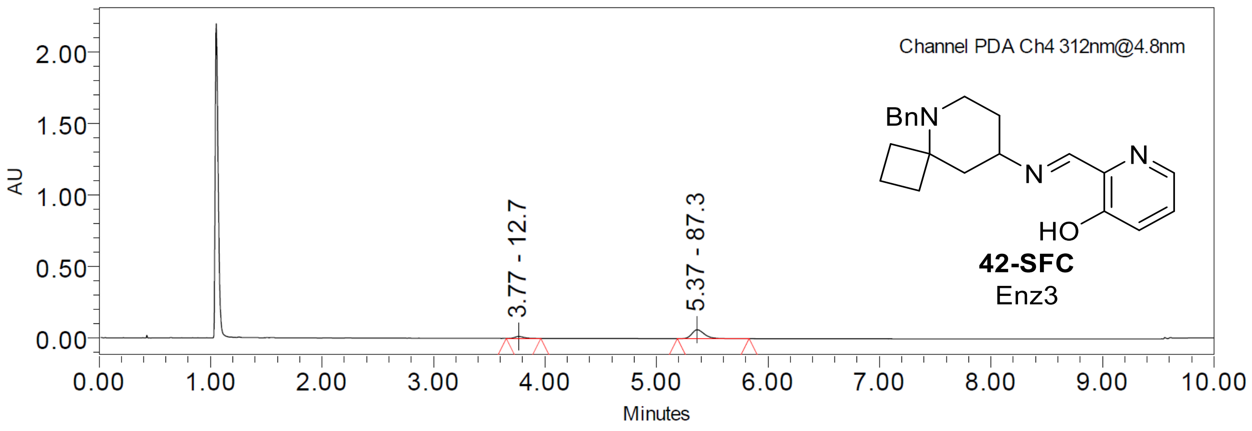
Channel PDA Ch4 312nm@4.8nm
BnN
N
N
HO
42-SFC
Enz3
3.77 - 12.7
5.37 - 87.3
AU
Minutes

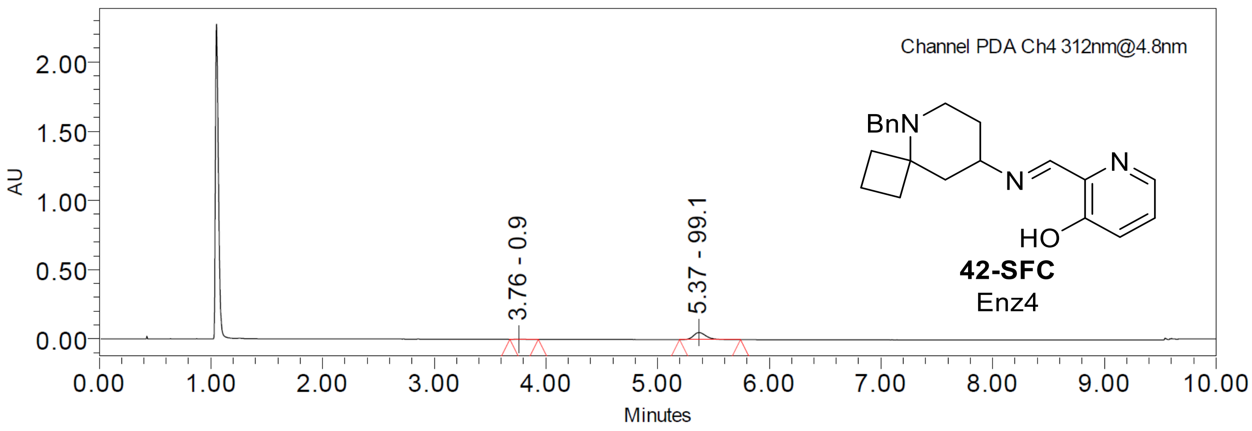
Channel PDA Ch4 312nm@4.8nm
BnN
N
N
HO
42-SFC
Enz4
3.76 - 0.9
5.37 - 99.1
AU
Minutes

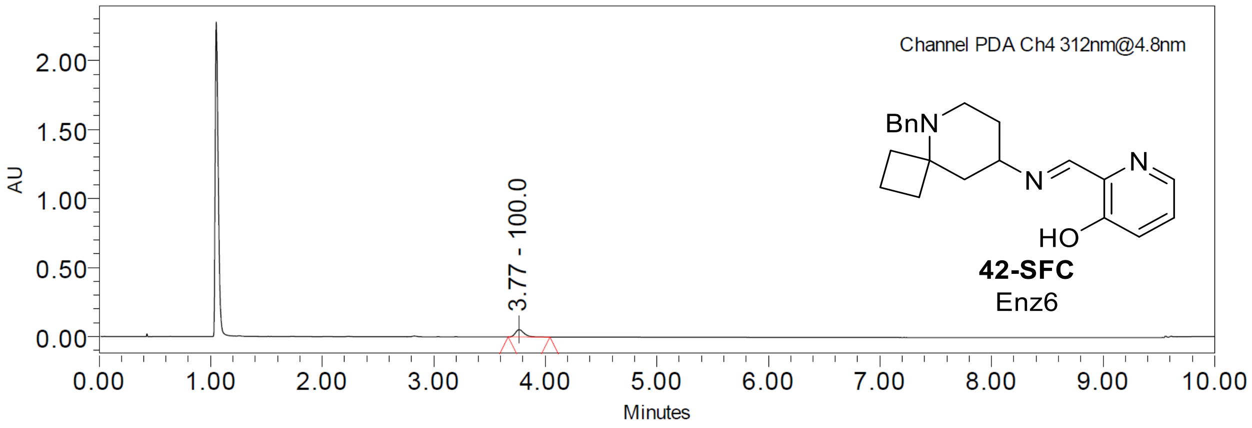
Channel PDA Ch4 312nm@4.8nm
BnN
N
N
HO
42-SFC
Enz6
3.77 - 100.0
AU
Minutes

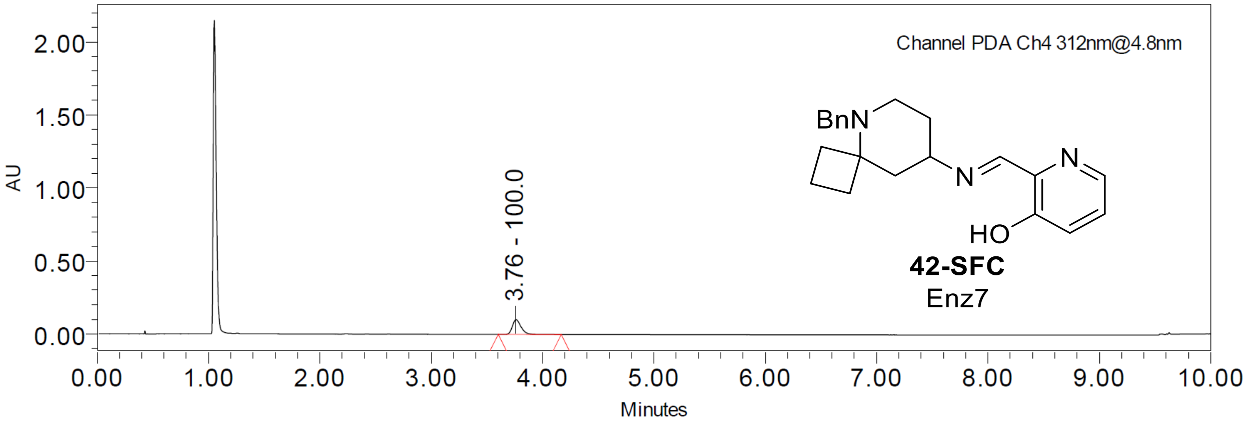
Channel PDA Ch4 312nm@4.8nm
BnN
N
N
HO
42-SFC
Enz7
3.76 - 100.0
AU
Minutes
0.00
0.50
1.00
1.50
2.00
1.00
2.00
3.00
4.00
5.00
6.00
7.00
8.00
9.00
10.00

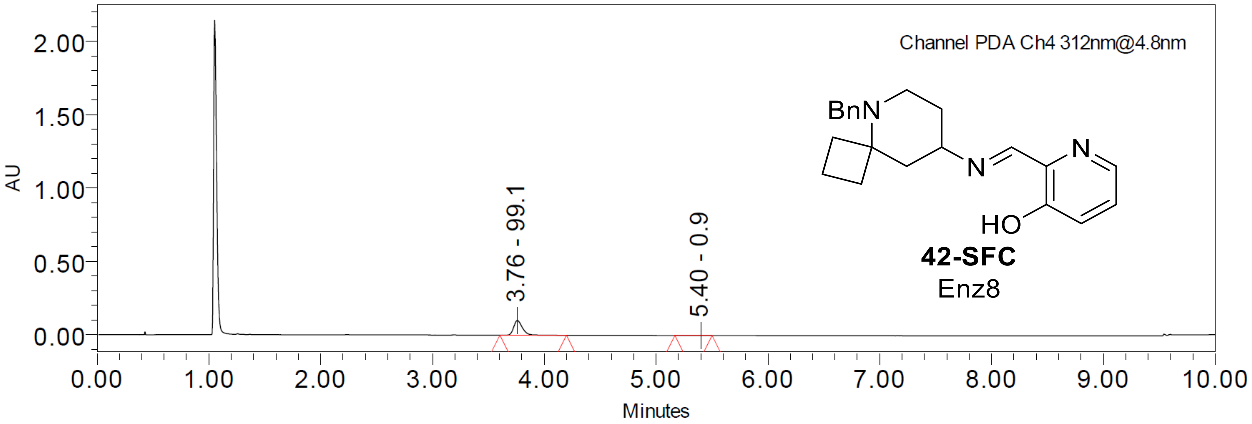
Channel PDA Ch4 312nm@4.8nm
BnN
N
N
HO
42-SFC
Enz8
3.76 - 99.1
5.40 - 0.9
AU
Minutes
0.00
0.50
1.00
1.50
2.00
1.00
2.00
3.00
4.00
5.00
6.00
7.00
8.00
9.00
10.00

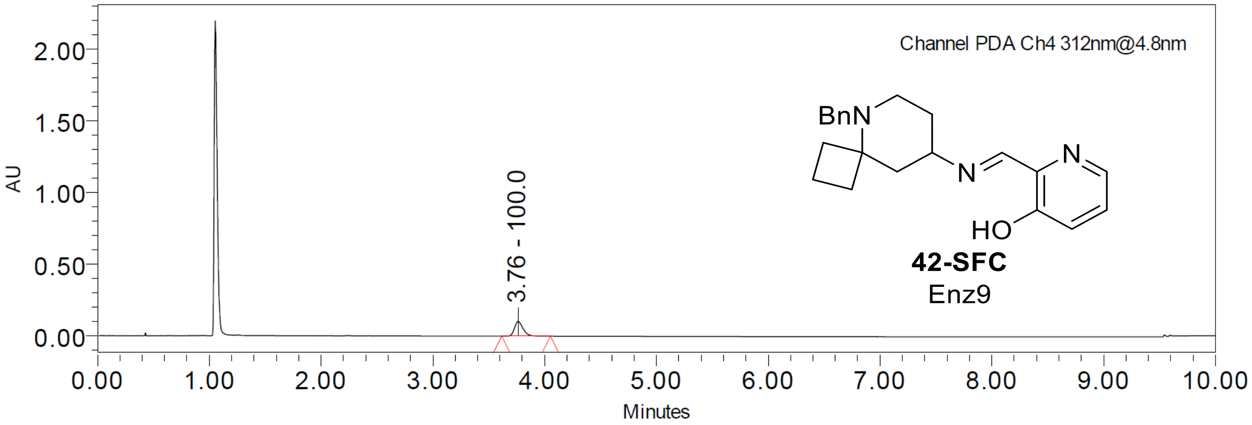
Channel PDA Ch4 312nm@4.8nm
BnN
N
N
HO
42-SFC
Enz9
3.76 - 100.0
AU
Minutes
0.00
0.50
1.00
1.50
2.00
1.00
2.00
3.00
4.00
5.00
6.00
7.00
8.00
9.00
10.00

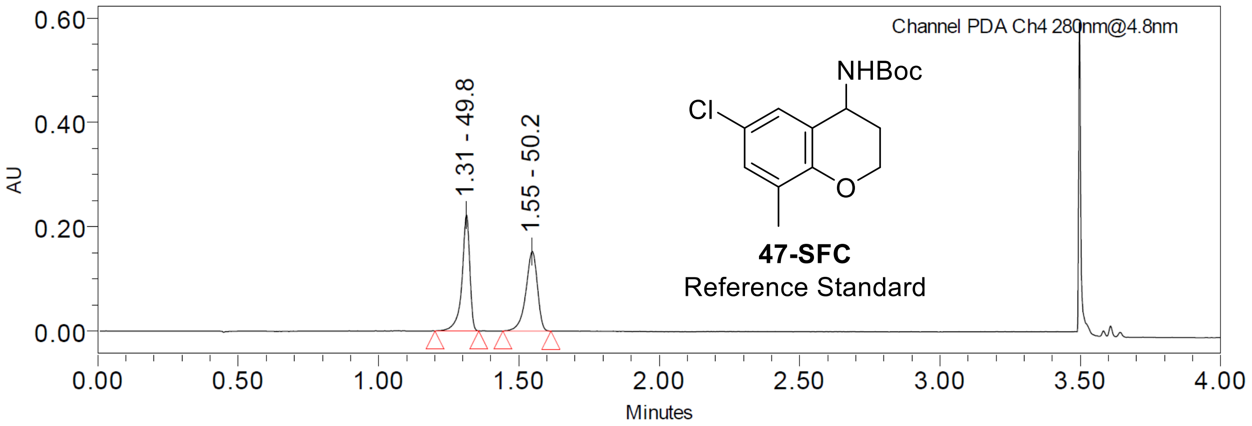
Channel PDA Ch4 280nm@4.8nm
NHBoc
Cl
O
47-SFC
Reference Standard
1.31 - 49.8
1.55 - 50.2
AU
Minutes

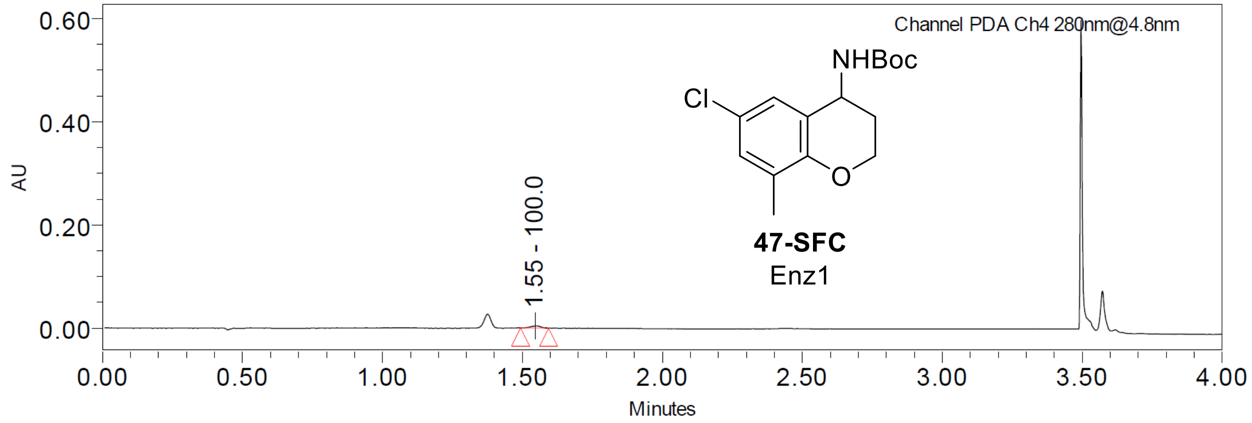
Channel PDA Ch4 280nm@4.8nm
NHBoc
Cl
O
47-SFC
Enz1
1.55 - 100.0
AU
Minutes

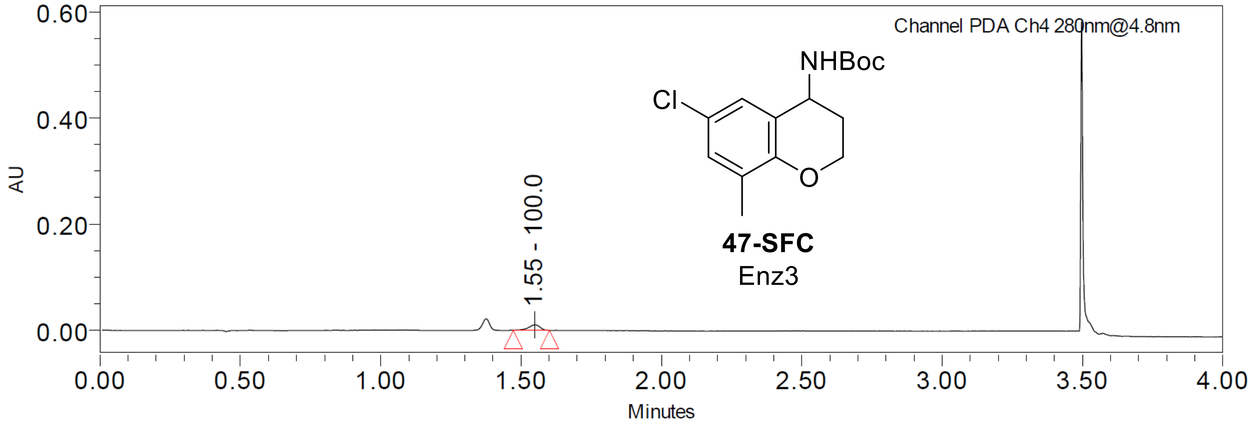
Channel PDA Ch4 280nm@4.8nm
NHBoc
Cl
O
47-SFC
Enz3
1.55 - 100.0
AU
Minutes

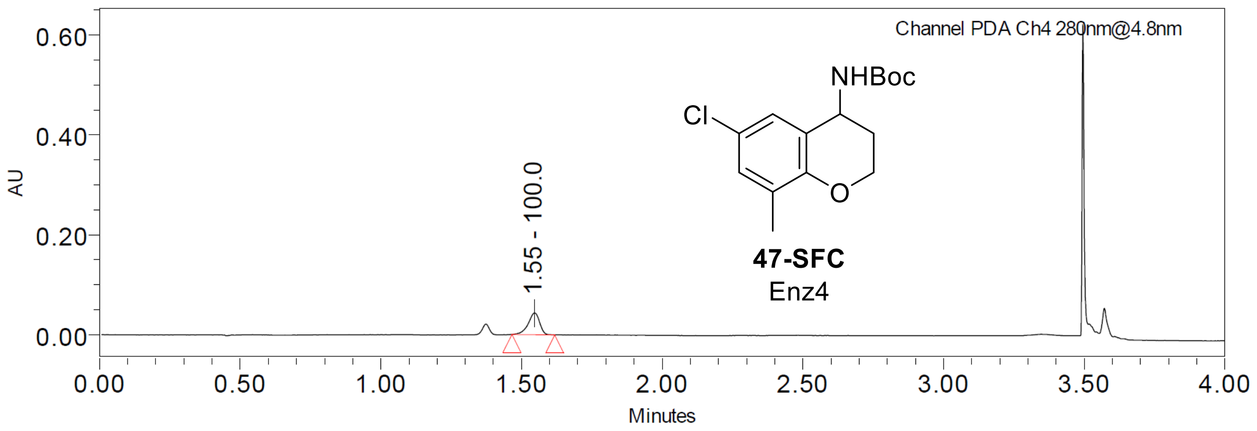
Channel PDA Ch4 280nm@4.8nm
NHBoc
Cl
O
47-SFC
Enz4
1.55 - 100.0
AU
Minutes

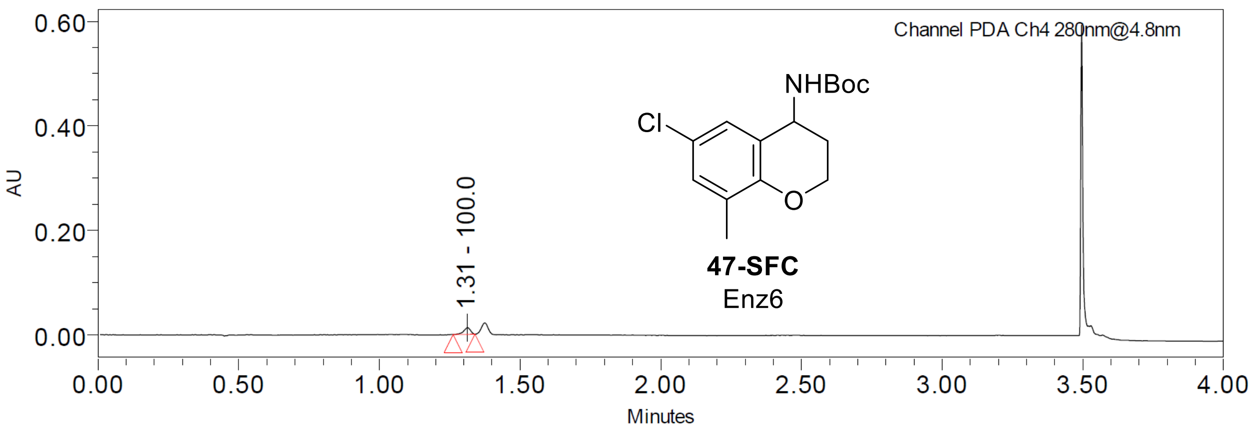
Channel PDA Ch4 280nm@4.8nm
NHBoc
Cl
O
47-SFC
Enz6
1.31 - 100.0
AU
Minutes

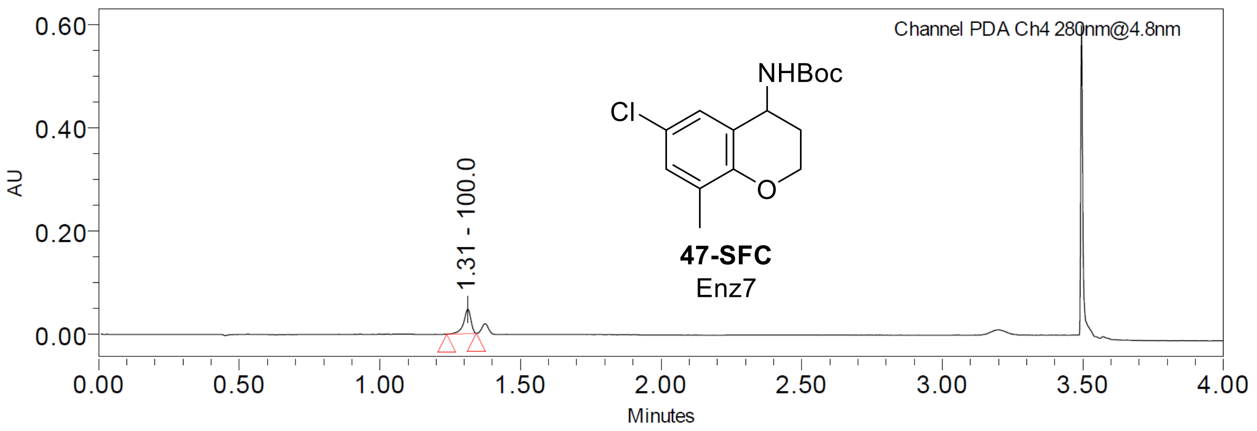
Channel PDA Ch4 280nm@4.8nm
NHBoc
Cl
O
47-SFC
Enz7
1.31 - 100.0
AU
Minutes

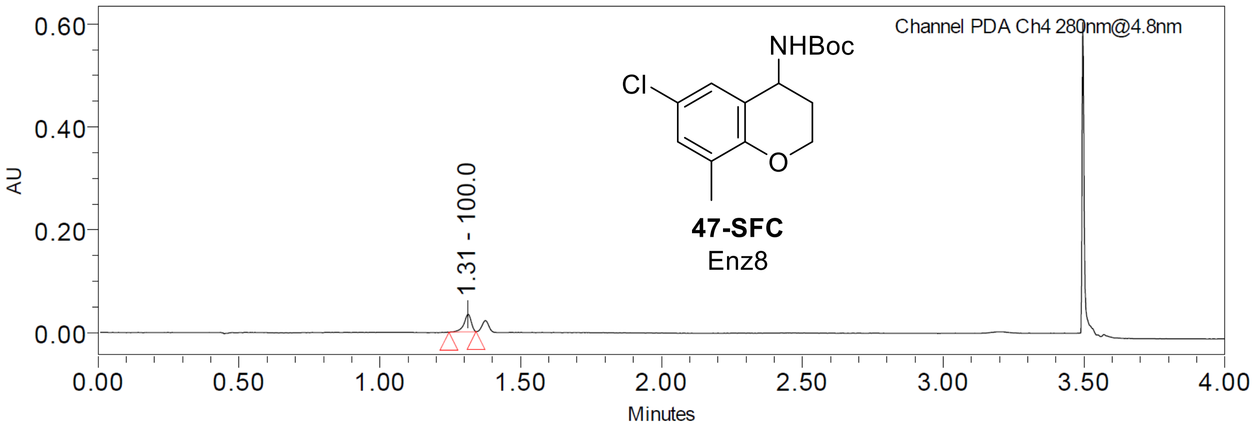
Channel PDA Ch4 280nm@4.8nm
NHBoc
Cl
O
47-SFC
Enz8
1.31 - 100.0
AU
Minutes

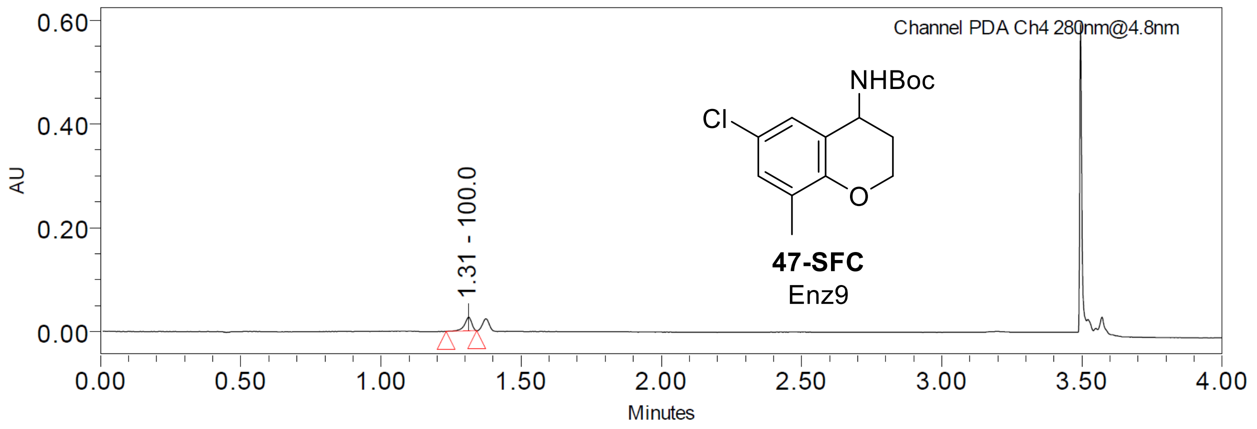
Channel PDA Ch4 280nm@4.8nm
NHBoc
Cl
O
47-SFC
Enz9
1.31 - 100.0
AU
Minutes

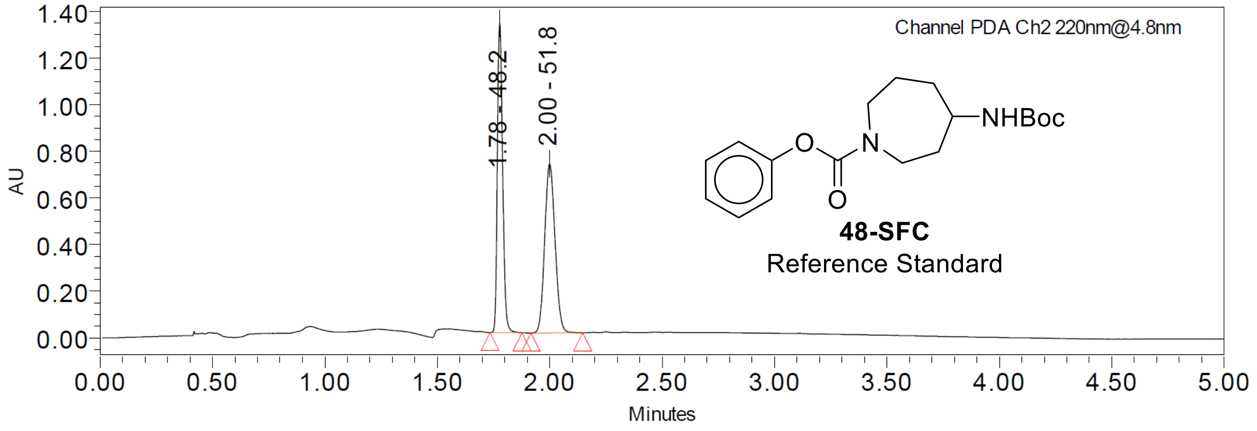
Channel PDA Ch2 220nm@4.8nm
O
N
NHBoc
O
48-SFC
Reference Standard
1.78 - 48.2
2.00 - 51.8
AU
Minutes

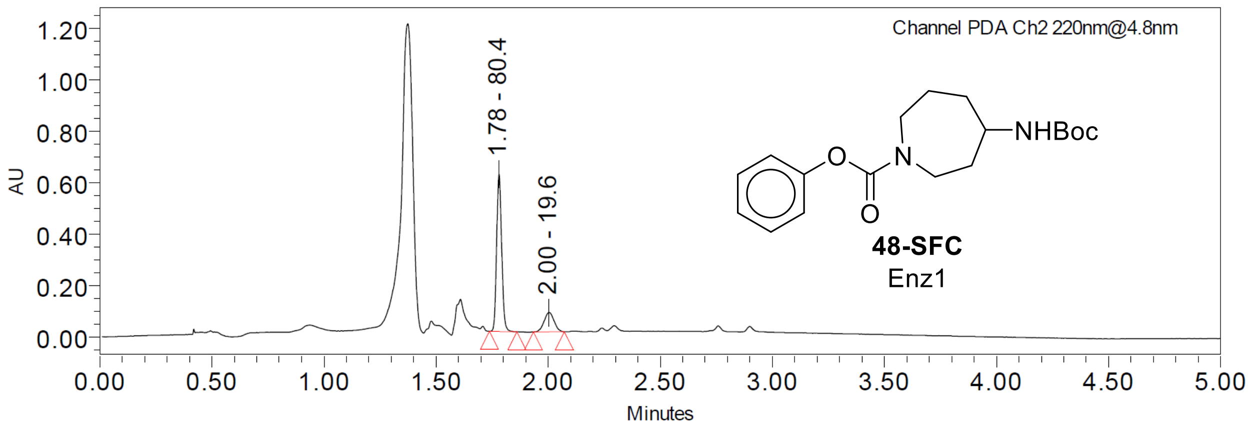
Channel PDA Ch2 220nm@4.8nm
1.78 - 80.4
2.00 - 19.6
NHBoc
O
N
O
48-SFC
Enz1
AU
Minutes

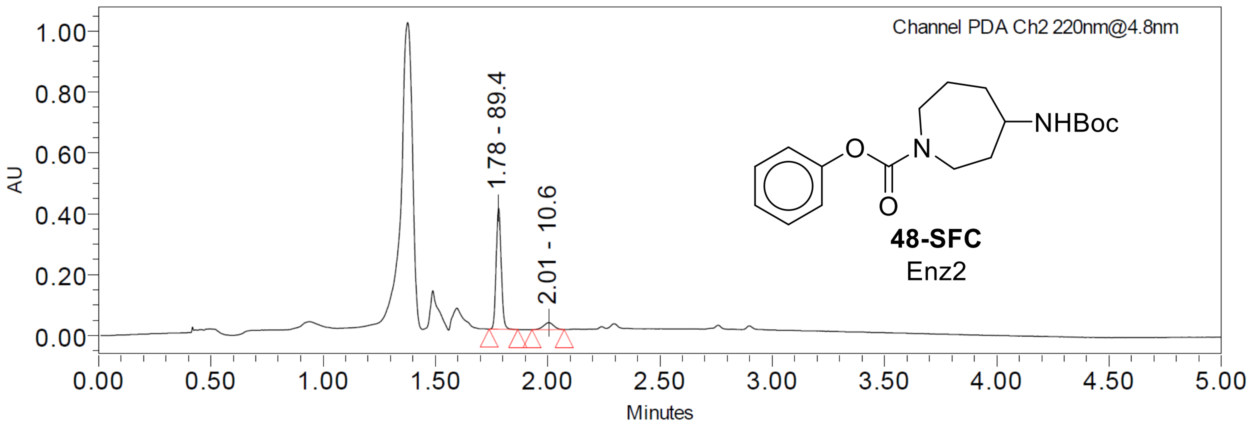
Channel PDA Ch2 220nm@4.8nm
1.78 - 89.4
2.01 - 10.6
NHBoc
O
N
O
48-SFC
Enz2
AU
Minutes

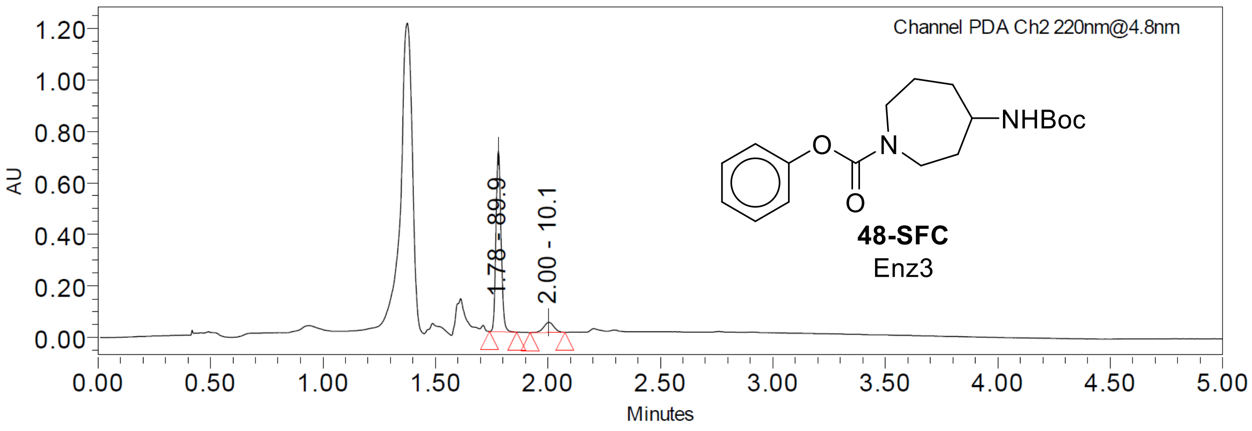
Channel PDA Ch2 220nm@4.8nm
1.78 - 89.9
2.00 - 10.1
NHBoc
O
N
O
48-SFC
Enz3
AU
Minutes

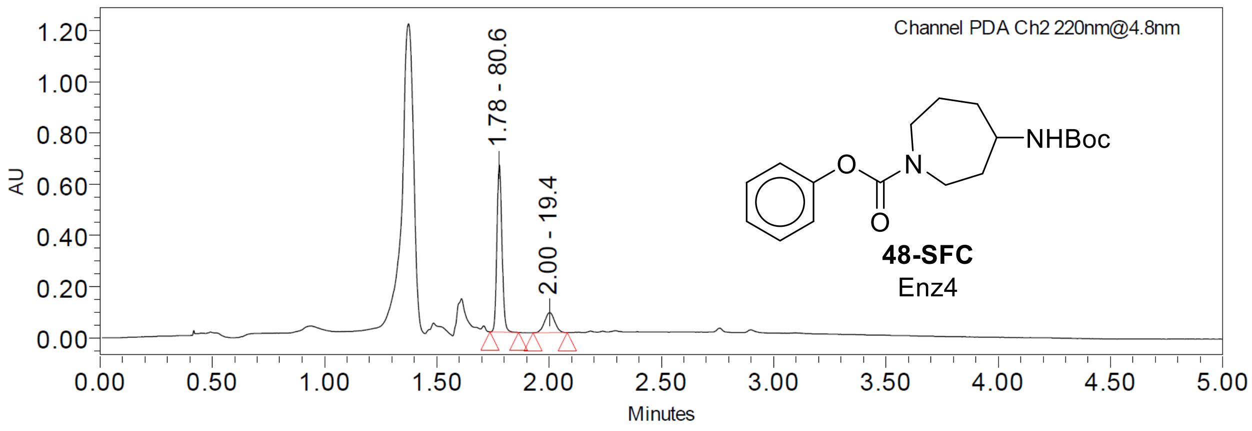
Channel PDA Ch2 220nm@4.8nm
1.78 - 80.6
2.00 - 19.4
AU
Minutes
NHBoc
O
N
O
48-SFC
Enz4

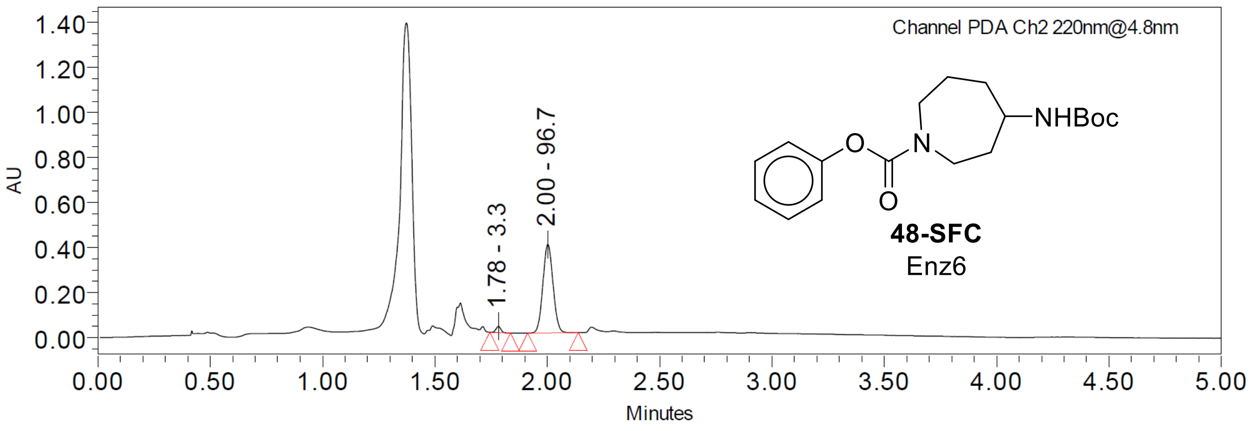
Channel PDA Ch2 220nm@4.8nm
1.78 - 3.3
2.00 - 96.7
AU
Minutes
NHBoc
O
N
O
48-SFC
Enz6

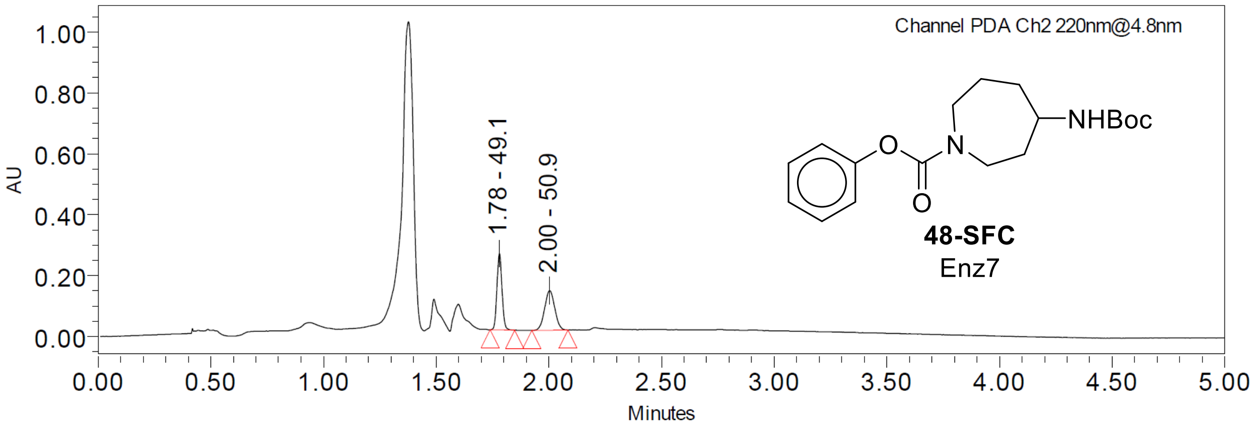
Channel PDA Ch2 220nm@4.8nm
1.78 - 49.1
2.00 - 50.9
AU
Minutes
NHBoc
O
N
O
48-SFC
Enz7

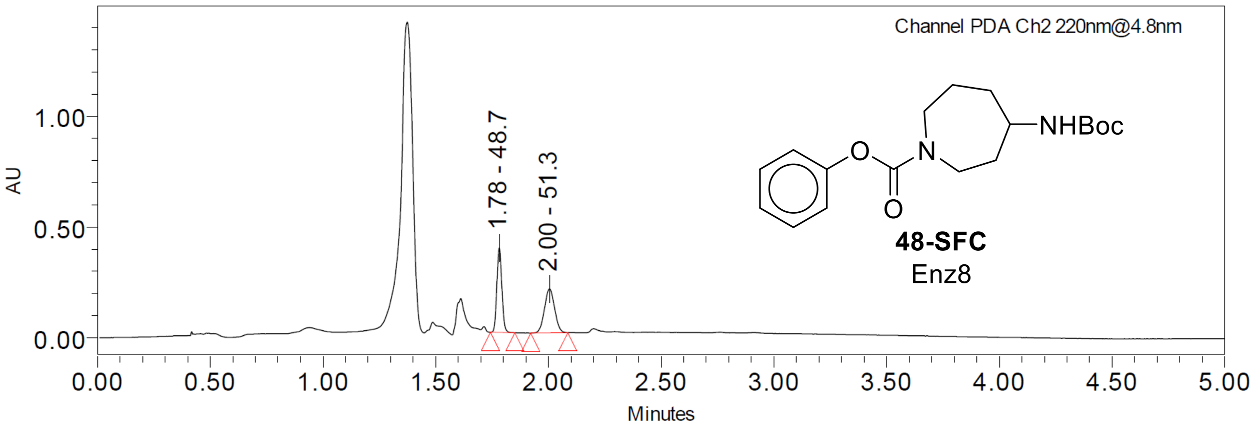
Channel PDA Ch2 220nm@4.8nm
1.78 - 48.7
2.00 - 51.3
NHBoc
O
N
O
48-SFC
Enz8
AU
Minutes

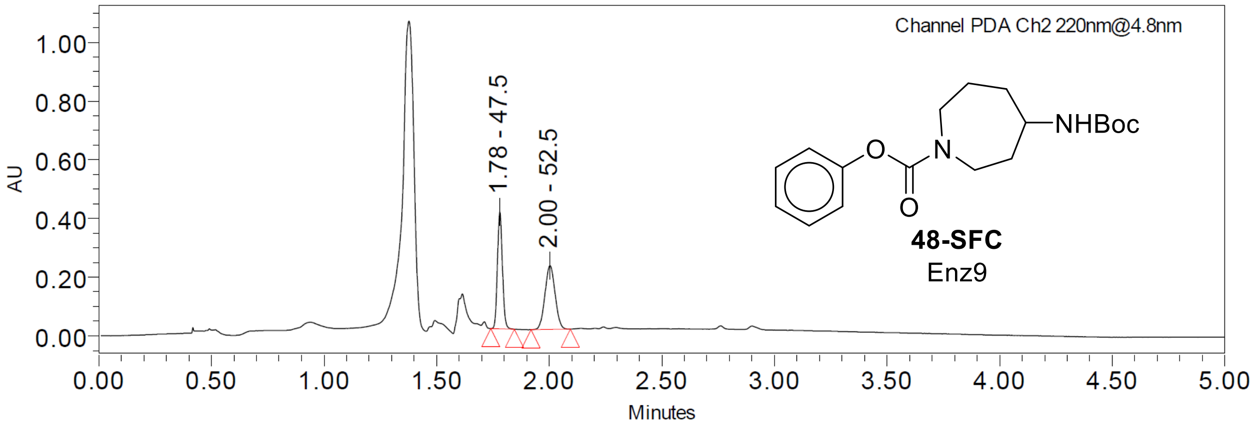
Channel PDA Ch2 220nm@4.8nm
1.78 - 47.5
2.00 - 52.5
NHBoc
O
N
O
48-SFC
Enz9
AU
Minutes

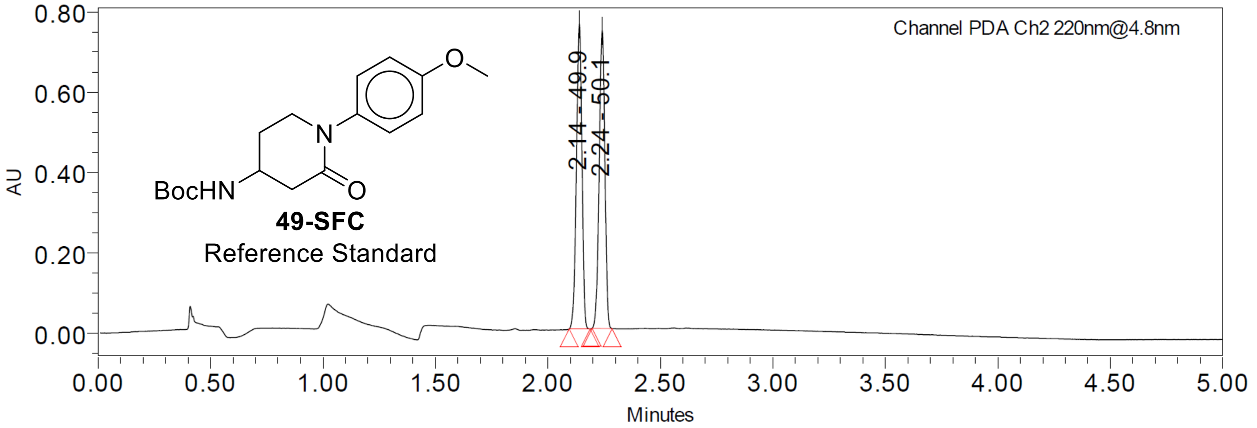
Channel PDA Ch2 220nm@4.8nm
2.14 - 49.9
2.24 - 50.1
O
N
BocHN
O
49-SFC
Reference Standard
AU
Minutes

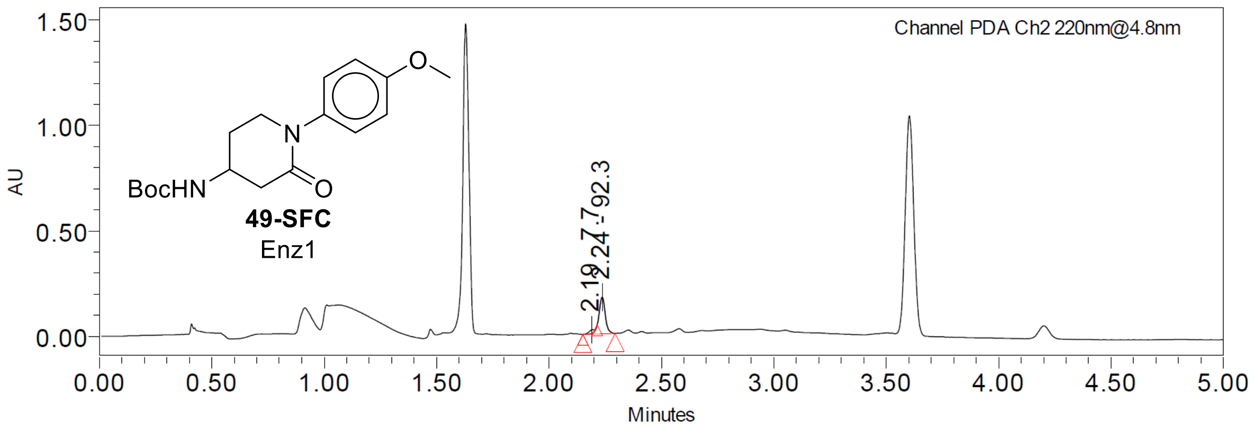
Channel PDA Ch2 220nm@4.8nm
1.50
1.00
0.50
0.00
AU
BocHN
N
O
O
49-SFC
Enz1
2.19 - 7.7
2.24 - 92.3
0.00
0.50
1.00
1.50
2.00
2.50
3.00
3.50
4.00
4.50
5.00
Minutes

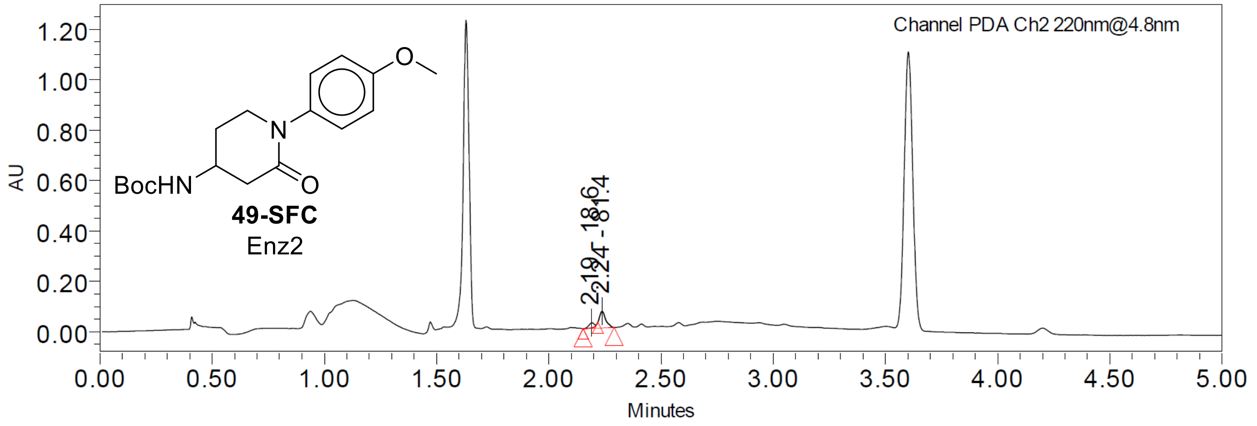
Channel PDA Ch2 220nm@4.8nm
1.20
1.00
0.80
0.60
0.40
0.20
0.00
AU
BocHN
N
O
O
49-SFC
Enz2
2.19 - 18.6
2.24 - 81.4
0.00
0.50
1.00
1.50
2.00
2.50
3.00
3.50
4.00
4.50
5.00
Minutes

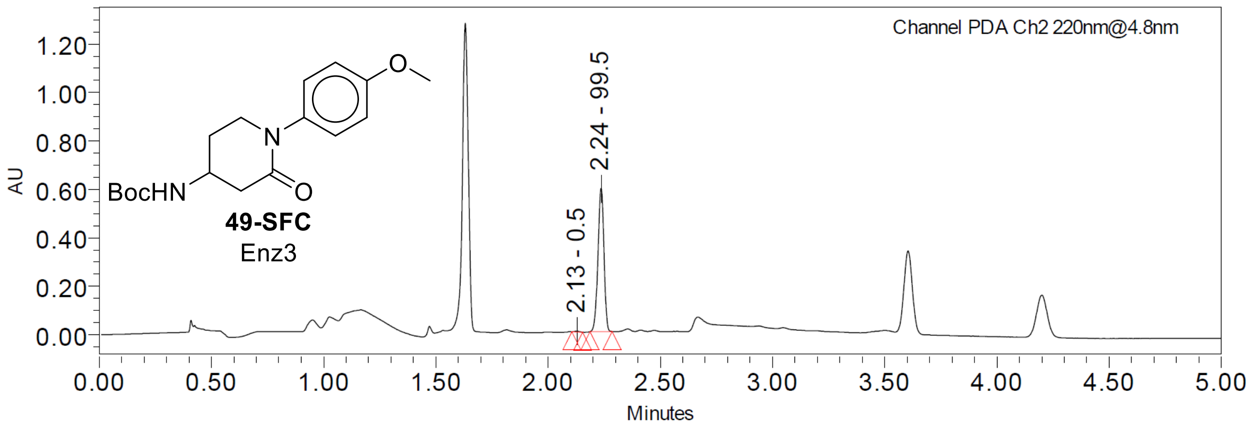
Channel PDA Ch2 220nm@4.8nm
1.20
1.00
0.80
0.60
0.40
0.20
0.00
AU
BocHN
N
O
O
49-SFC
Enz3
2.13 - 0.5
2.24 - 99.5
0.00
0.50
1.00
1.50
2.00
2.50
3.00
3.50
4.00
4.50
5.00
Minutes

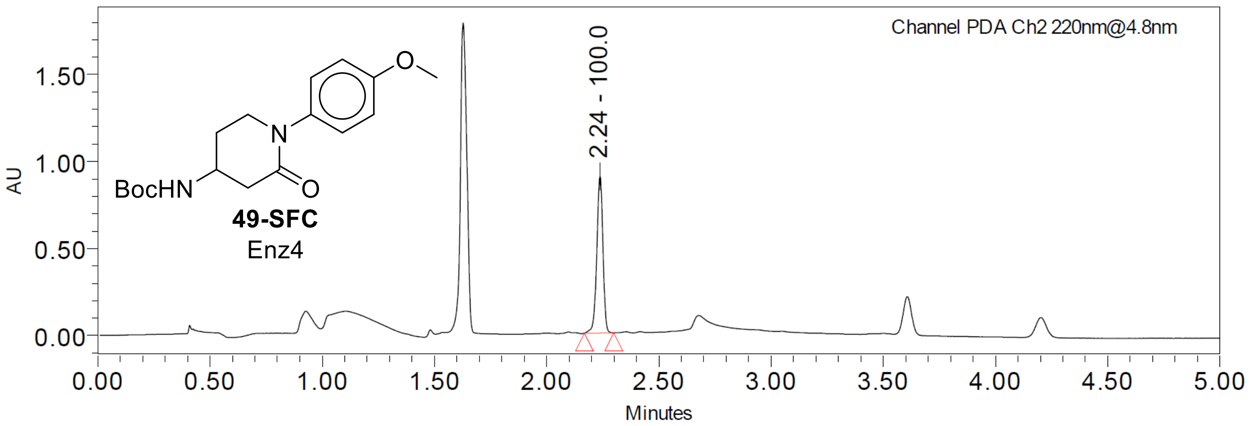
Channel PDA Ch2 220nm@4.8nm
BocHN
N
O
O
49-SFC
Enz4
2.24 - 100.0
AU
1.50
1.00
0.50
0.00
0.00 0.50 1.00 1.50 2.00 2.50 3.00 3.50 4.00 4.50 5.00
Minutes

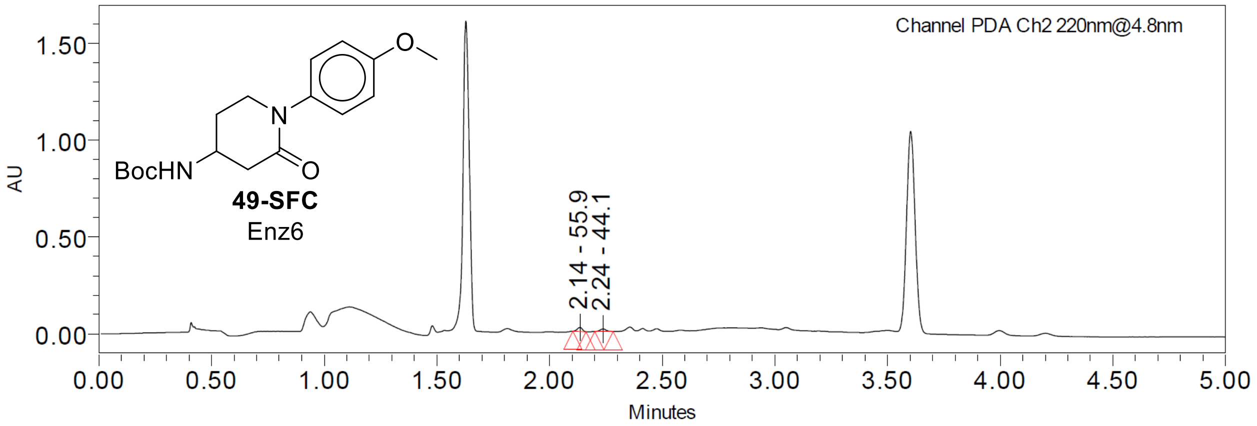
Channel PDA Ch2 220nm@4.8nm
BocHN
N
O
O
49-SFC
Enz6
2.14 - 55.9
2.24 - 44.1
AU
1.50
1.00
0.50
0.00
0.00 0.50 1.00 1.50 2.00 2.50 3.00 3.50 4.00 4.50 5.00
Minutes

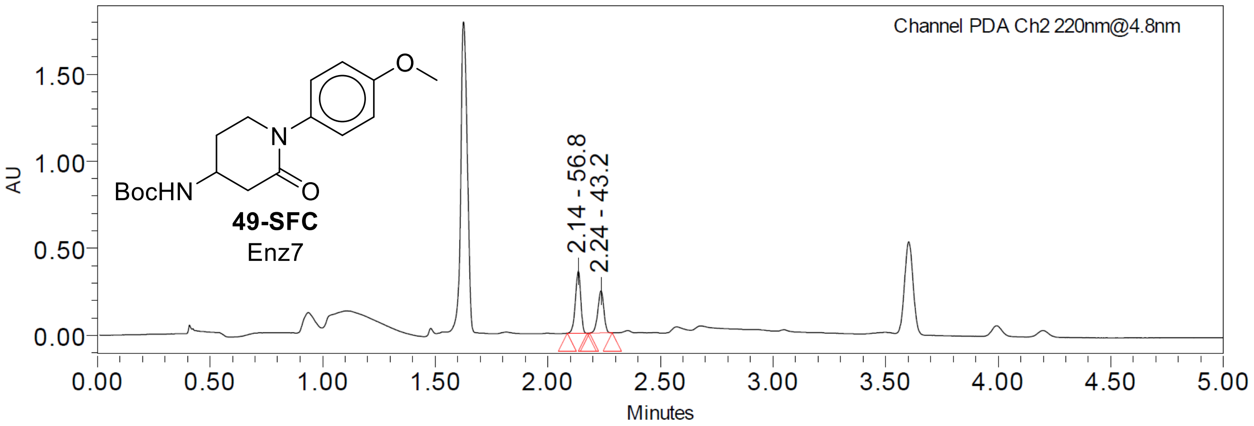
Channel PDA Ch2 220nm@4.8nm
BocHN
N
O
O
49-SFC
Enz7
2.14 - 56.8
2.24 - 43.2
AU
1.50
1.00
0.50
0.00
0.00 0.50 1.00 1.50 2.00 2.50 3.00 3.50 4.00 4.50 5.00
Minutes

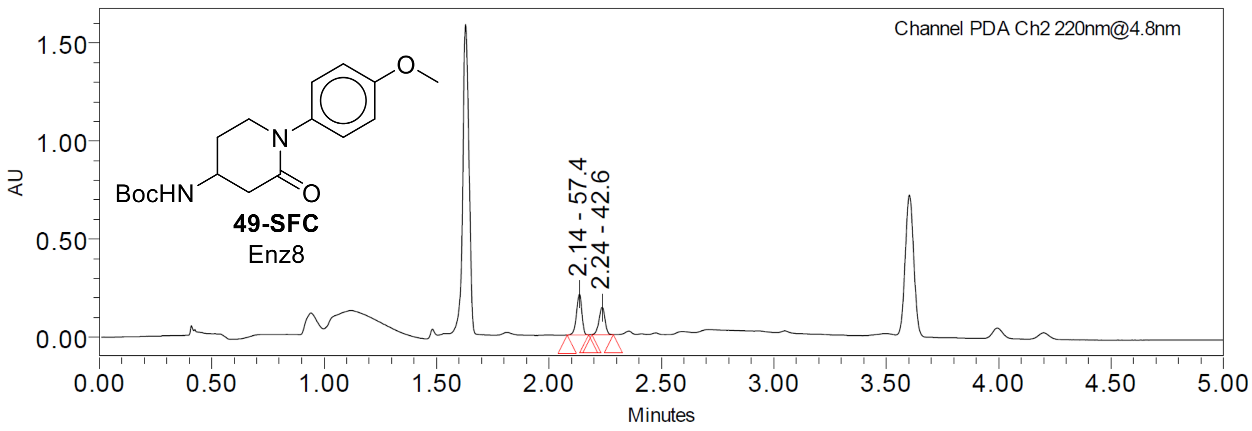

Channel PDA Ch2 220nm@4.8nm
BocHN
N
O
O
49-SFC
Enz8
2.14 - 57.4
2.24 - 42.6
AU
Minutes

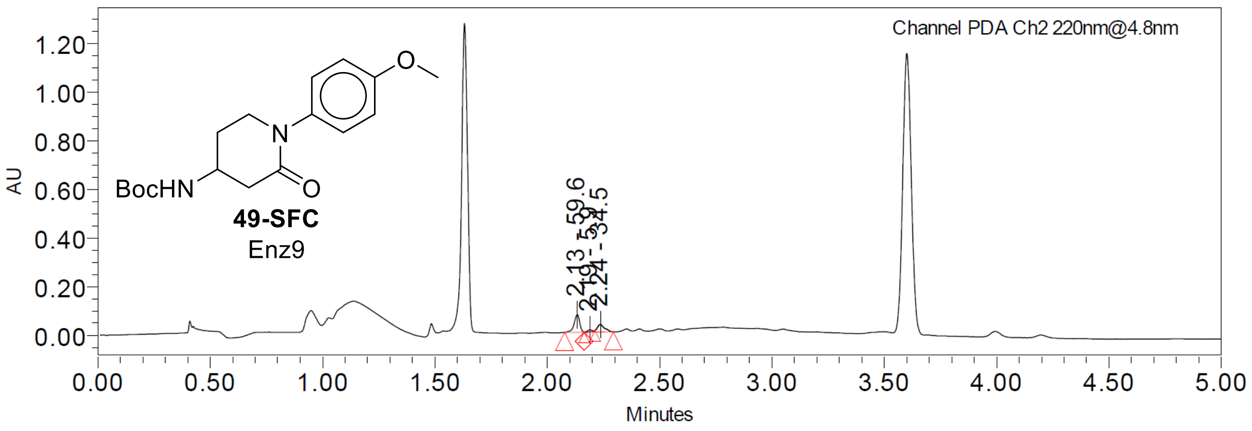

Channel PDA Ch2 220nm@4.8nm
BocHN
N
O
O
49-SFC
Enz9
2.13 - 59.6
2.24 - 34.5
AU
Minutes

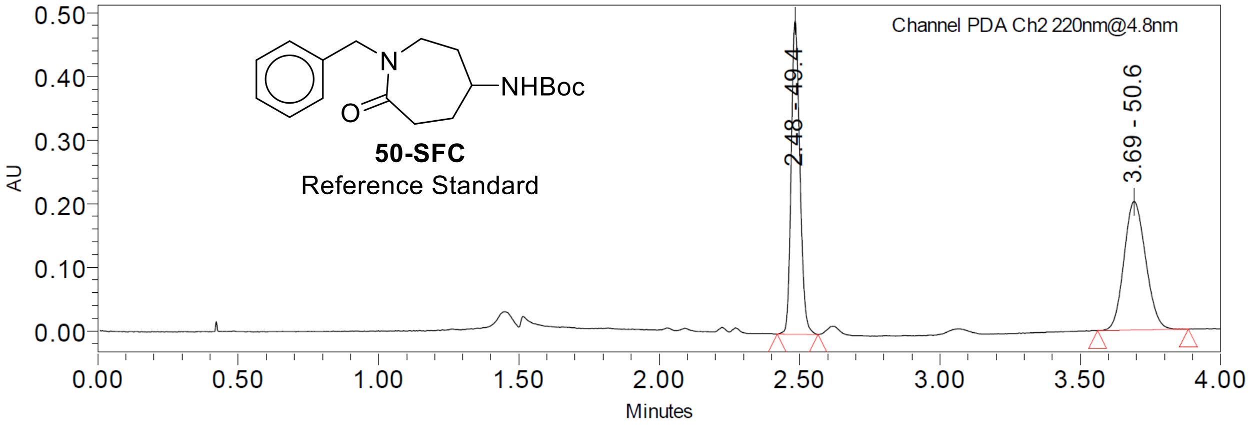

Channel PDA Ch2 220nm@4.8nm
N
O
NHBoc
50-SFC
Reference Standard
2.48 - 49.4
3.69 - 50.6
AU
Minutes

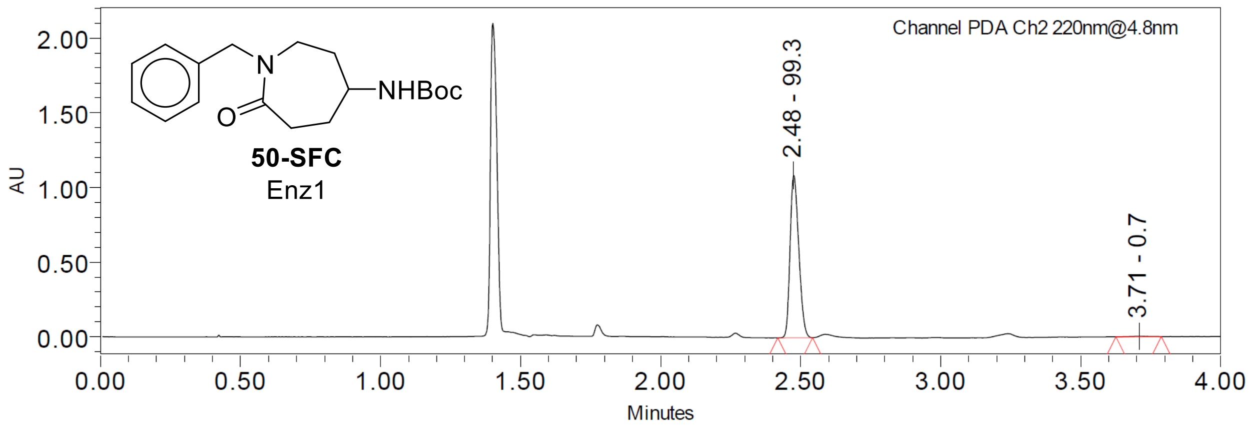

Channel PDA Ch2 220nm@4.8nm
NHBoc
N
O
50-SFC
Enz1
2.48 - 99.3
3.71 - 0.7
AU
Minutes

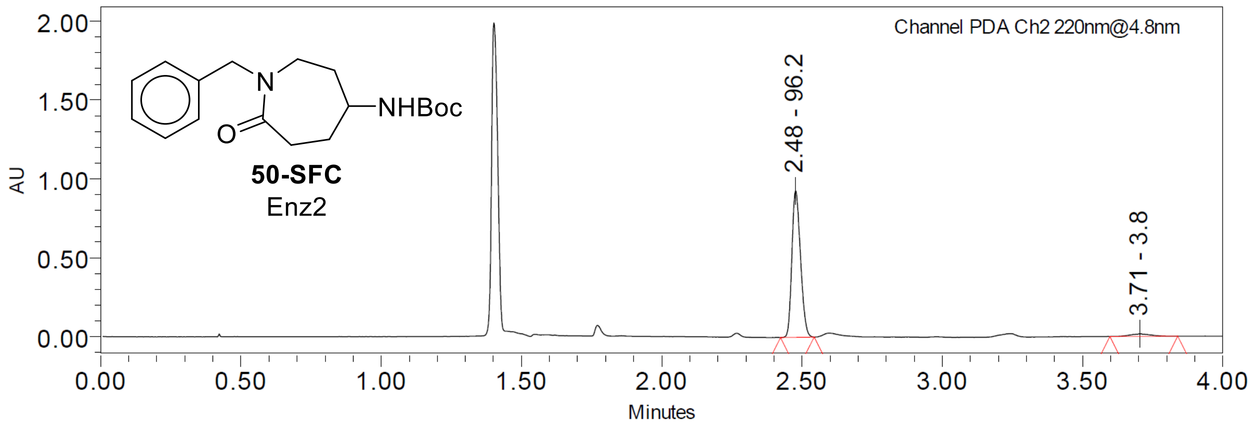

Channel PDA Ch2 220nm@4.8nm
NHBoc
N
O
50-SFC
Enz2
2.48 - 96.2
3.71 - 3.8
AU
Minutes

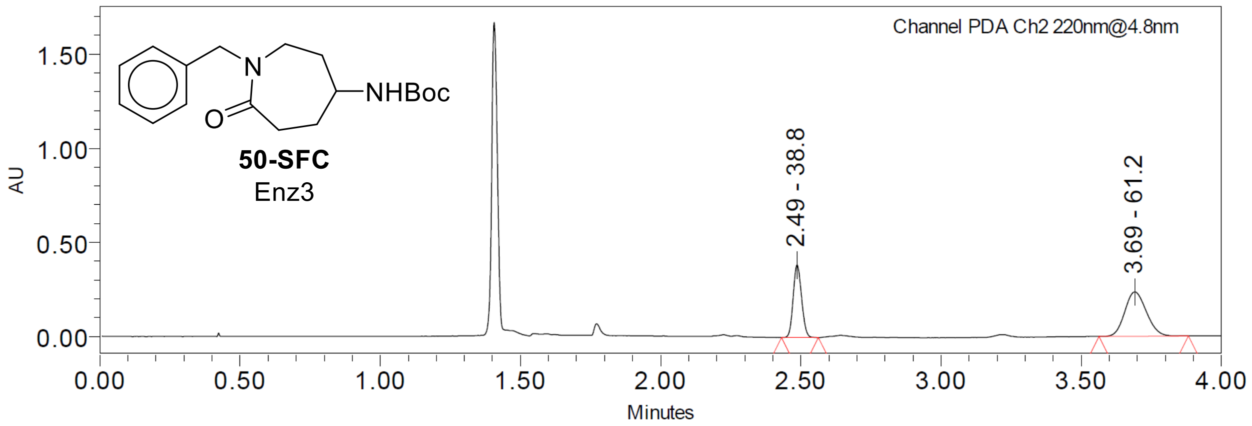

Channel PDA Ch2 220nm@4.8nm
NHBoc
N
O
50-SFC
Enz3
2.49 - 38.8
3.69 - 61.2
AU
Minutes

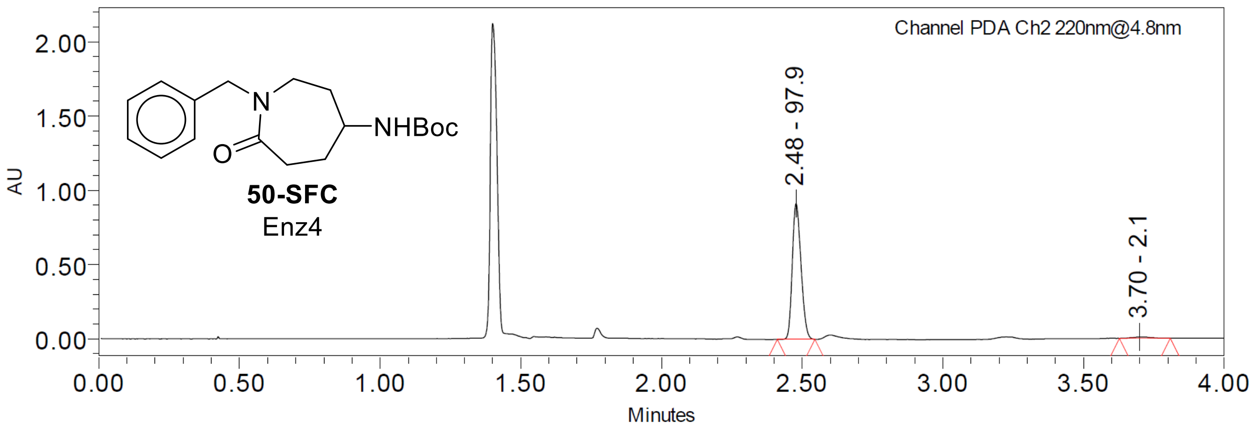
Channel PDA Ch2 220nm@4.8nm
NHBoc
N
O
50-SFC
Enz4
2.48 - 97.9
3.70 - 2.1
AU
Minutes

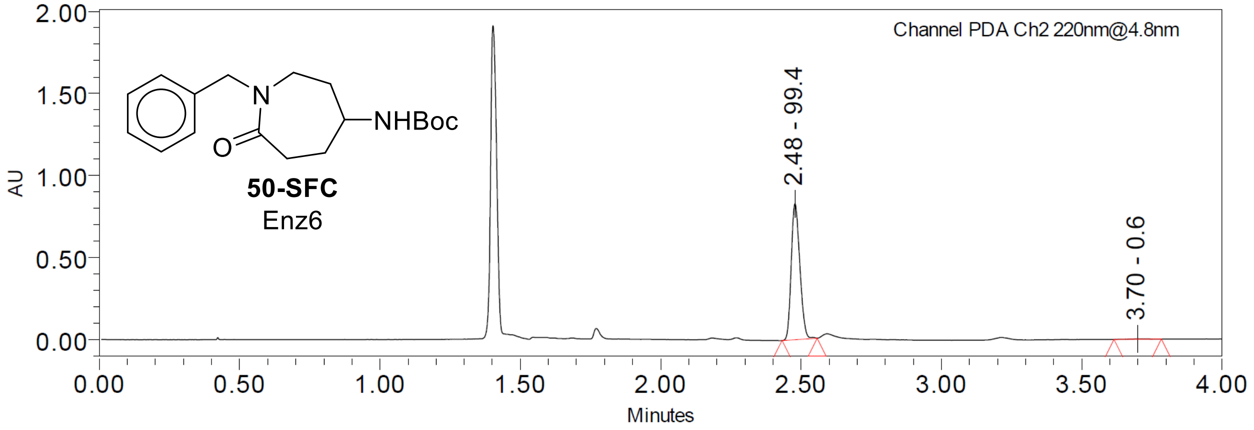
Channel PDA Ch2 220nm@4.8nm
NHBoc
N
O
50-SFC
Enz6
2.48 - 99.4
3.70 - 0.6
AU
Minutes

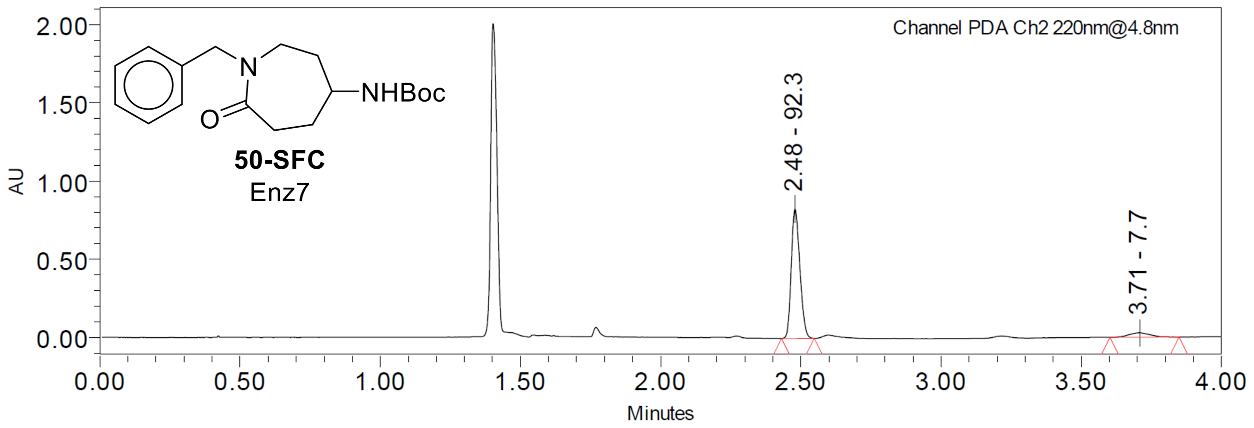
Channel PDA Ch2 220nm@4.8nm
NHBoc
N
O
50-SFC
Enz7
2.48 - 92.3
3.71 - 7.7
AU
Minutes

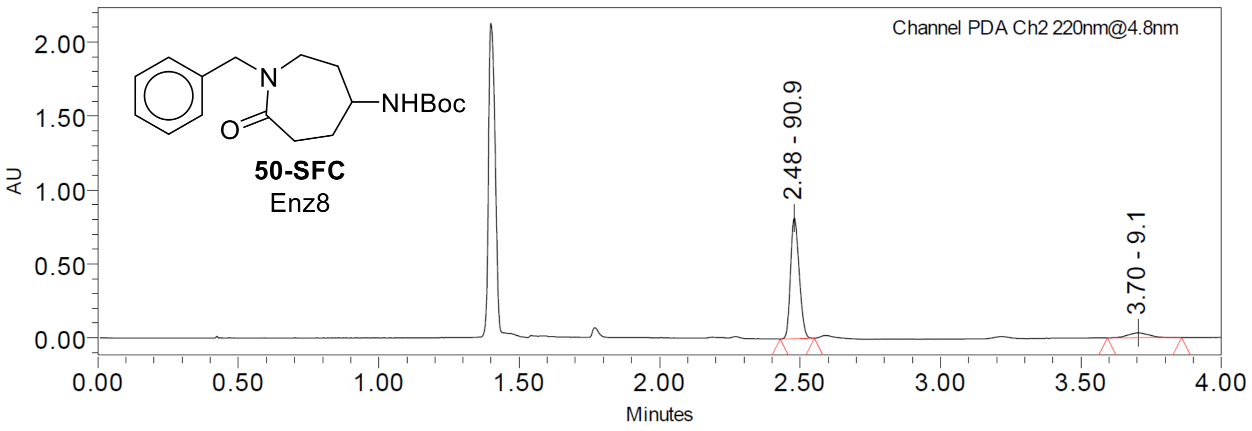

Channel PDA Ch2 220nm@4.8nm
NHBoc
N
O
50-SFC
Enz8
2.48 - 90.9
3.70 - 9.1
AU
Minutes

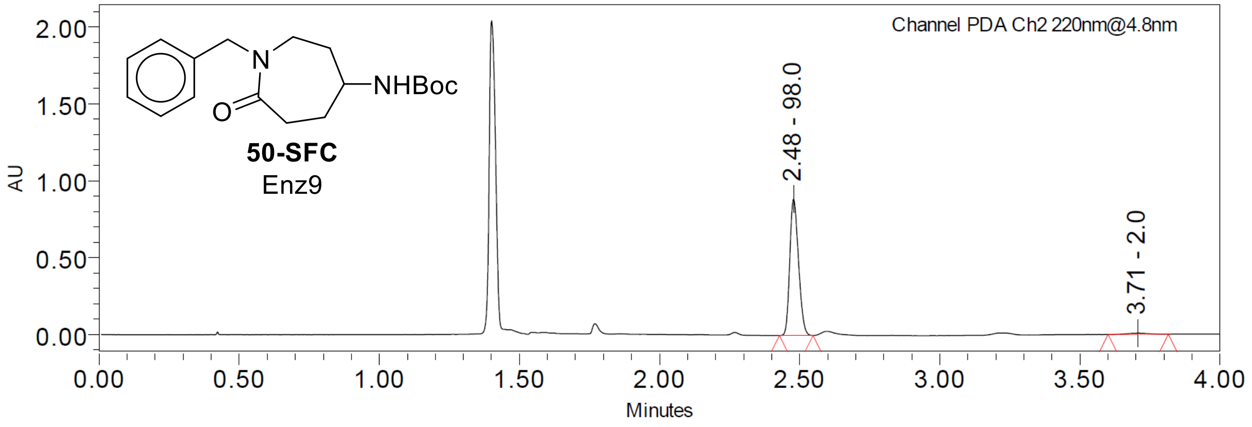

Channel PDA Ch2 220nm@4.8nm
NHBoc
N
O
50-SFC
Enz9
2.48 - 98.0
3.71 - 2.0
AU
Minutes

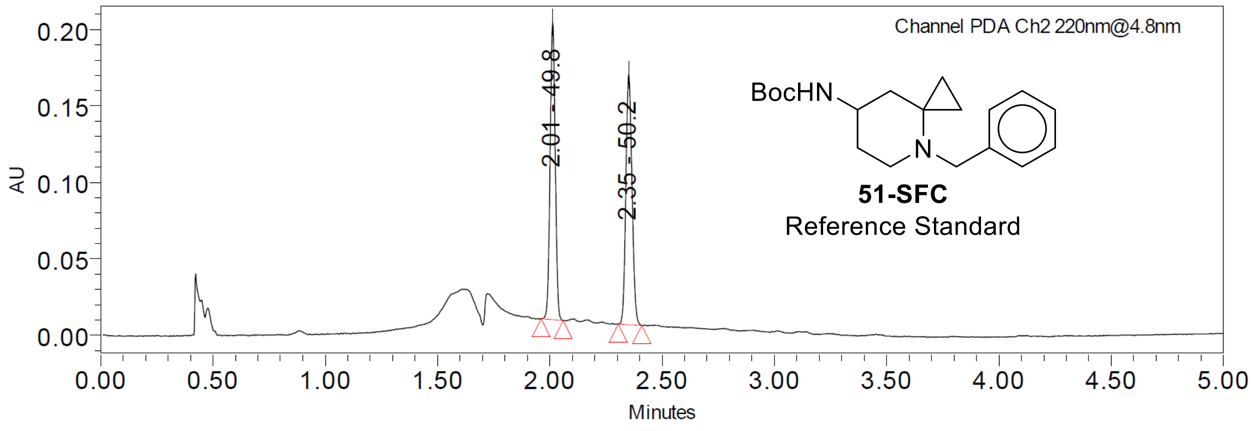

Channel PDA Ch2 220nm@4.8nm
BocHN
N
51-SFC
Reference Standard
2.01 - 49.8
2.35 - 50.2
AU
Minutes

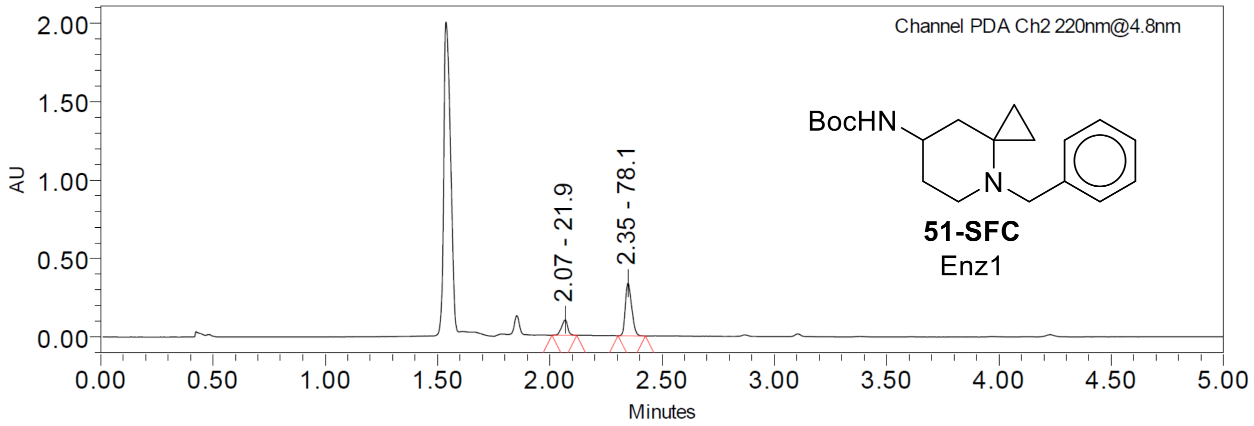

Note: Impurity present at T=2.07

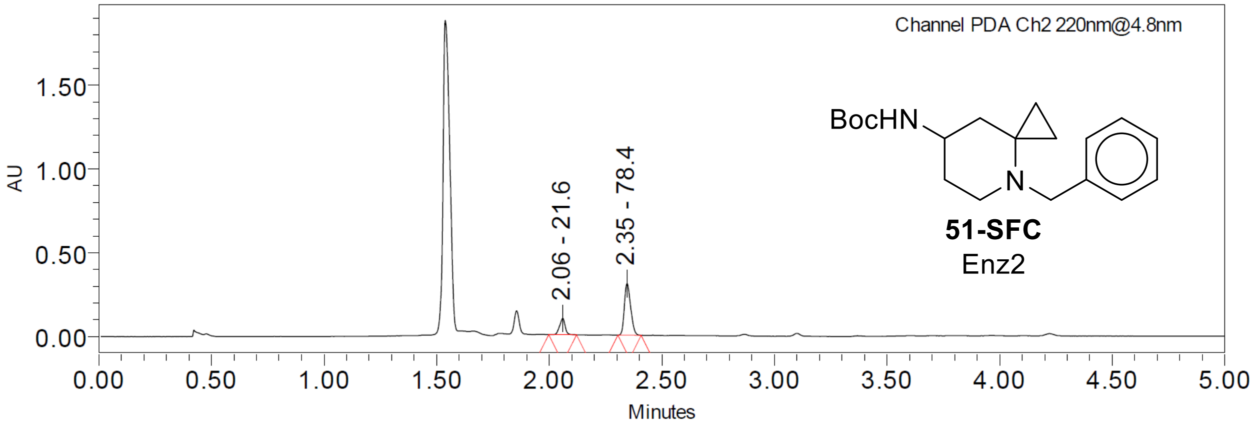

Note: Impurity present at T=2.06

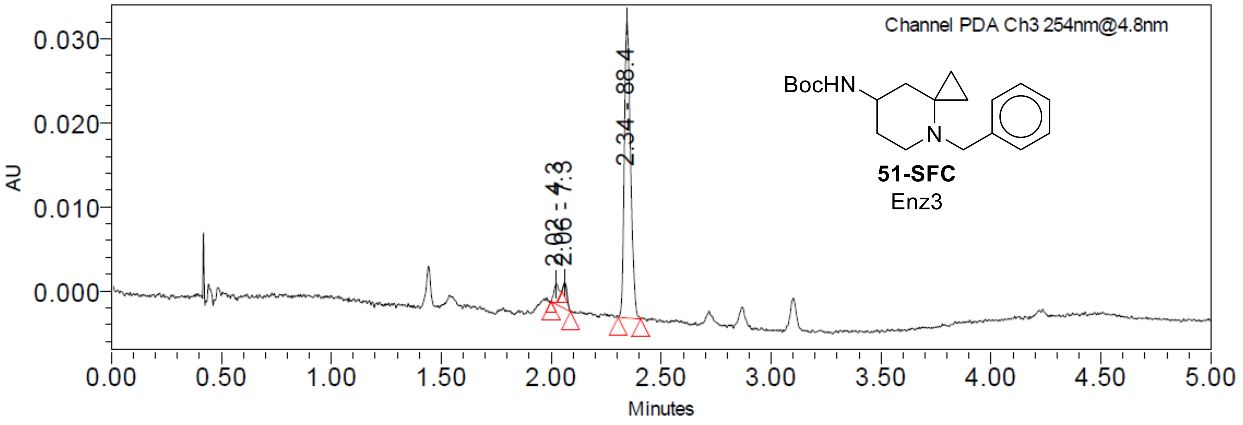

Note: Impurity present at T=2.06

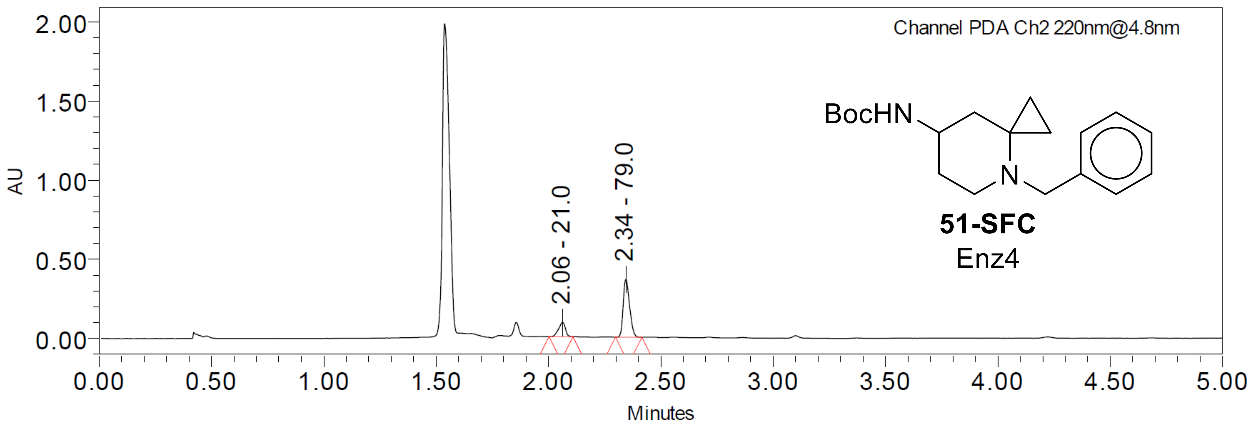

Note: Impurity present at T=2.06

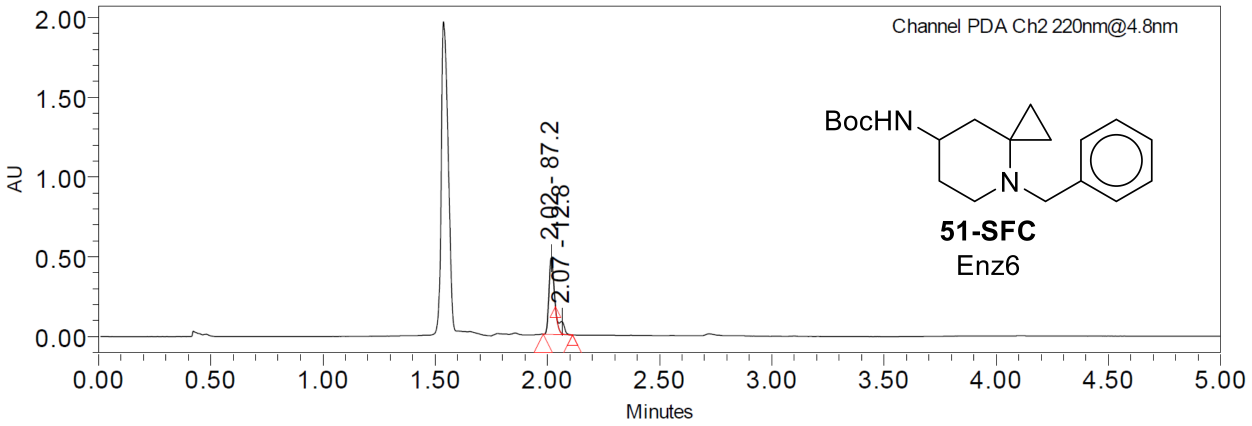

Note: Advanced integration was used for Peak 1; impurity present at T=2.07

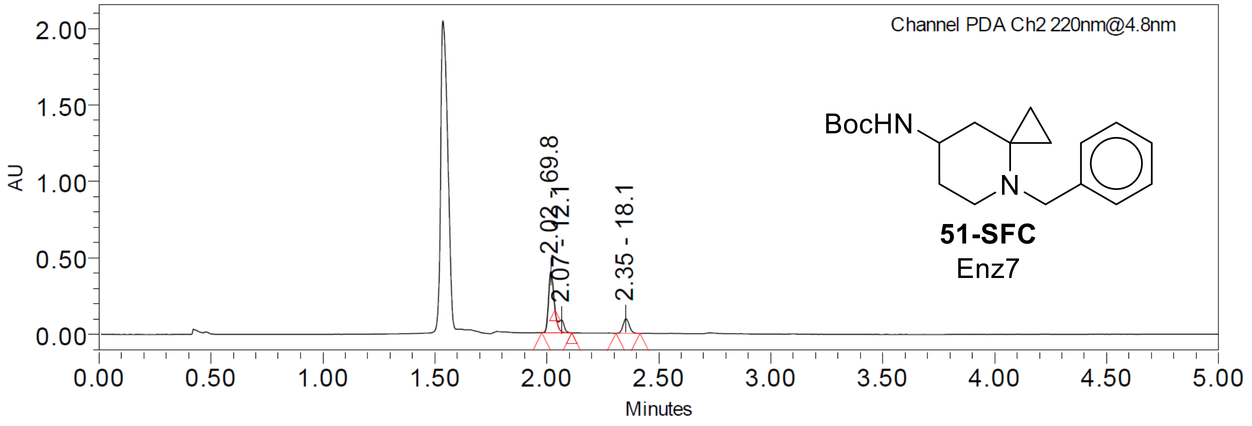

Note: Advanced integration was used for Peak 1; impurity present at T=2.07

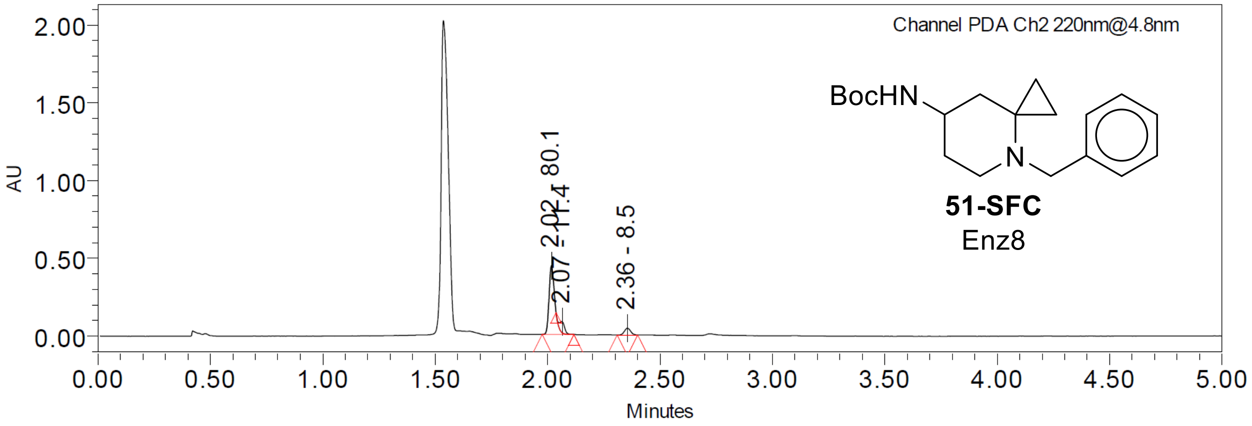

Note: Advanced integration was used for Peak 1; impurity present at T=2.07

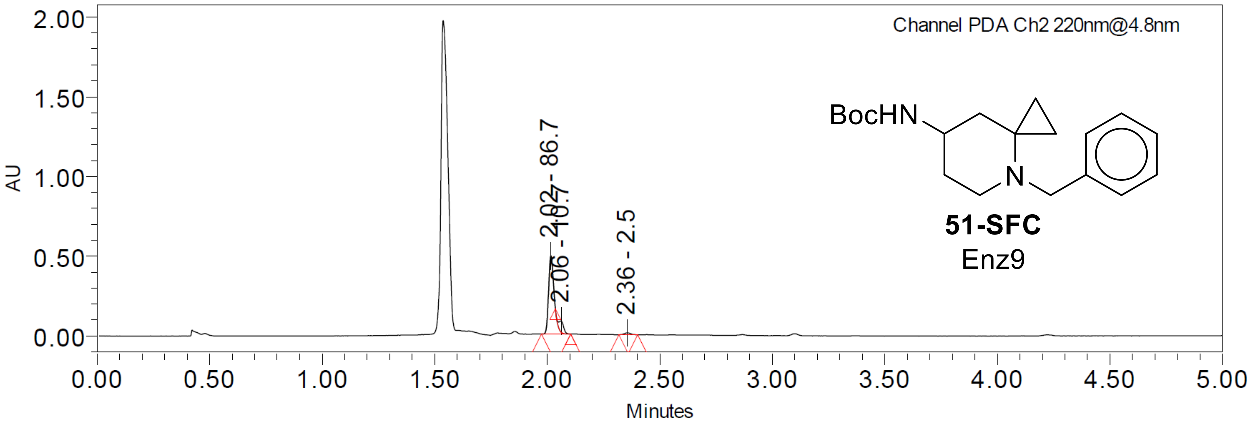

Note: Advanced integration was used for Peak 1; impurity present at T=2.07

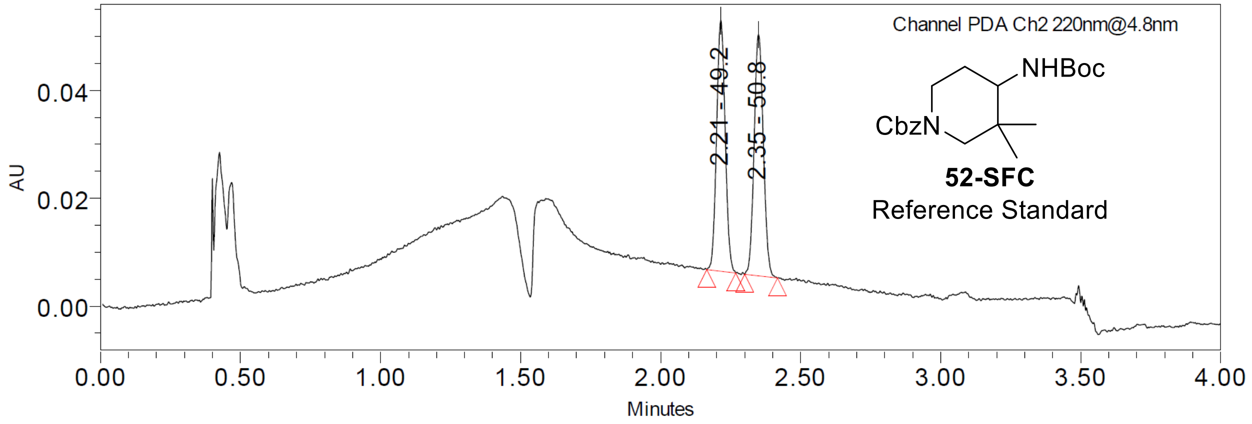

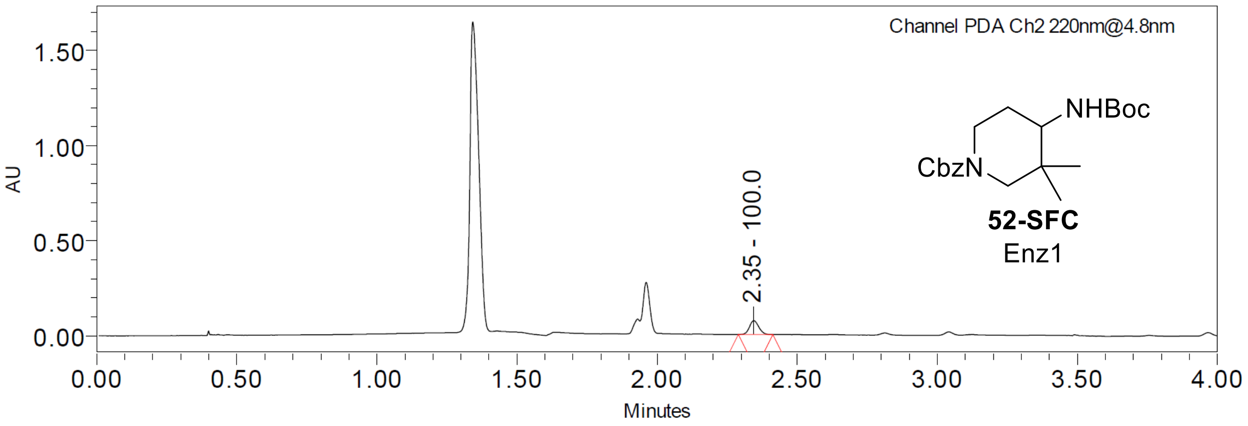

Channel PDA Ch2 220nm@4.8nm
NHBoc
CbzN
52-SFC
Enz1
2.35 - 100.0
AU
Minutes

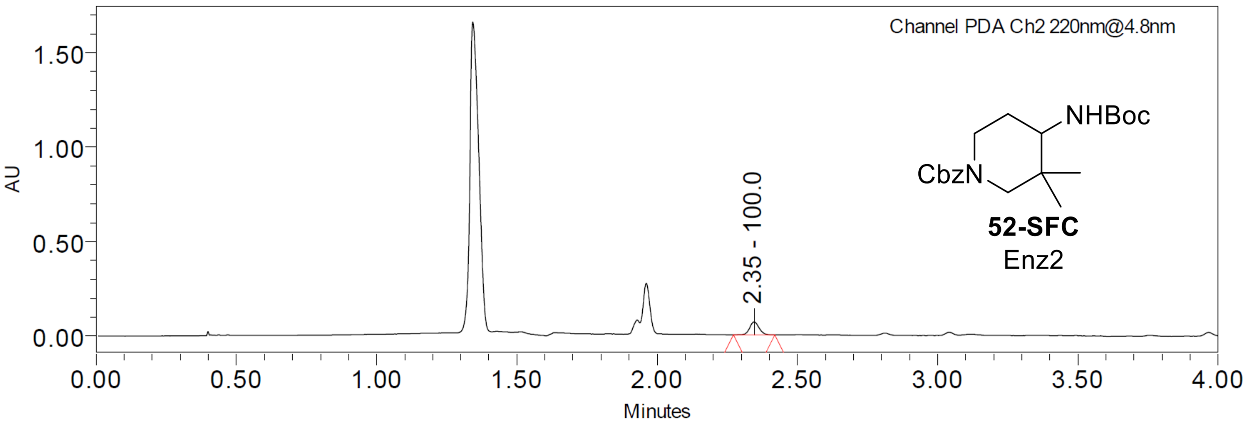

Channel PDA Ch2 220nm@4.8nm
NHBoc
CbzN
52-SFC
Enz2
2.35 - 100.0
AU
Minutes

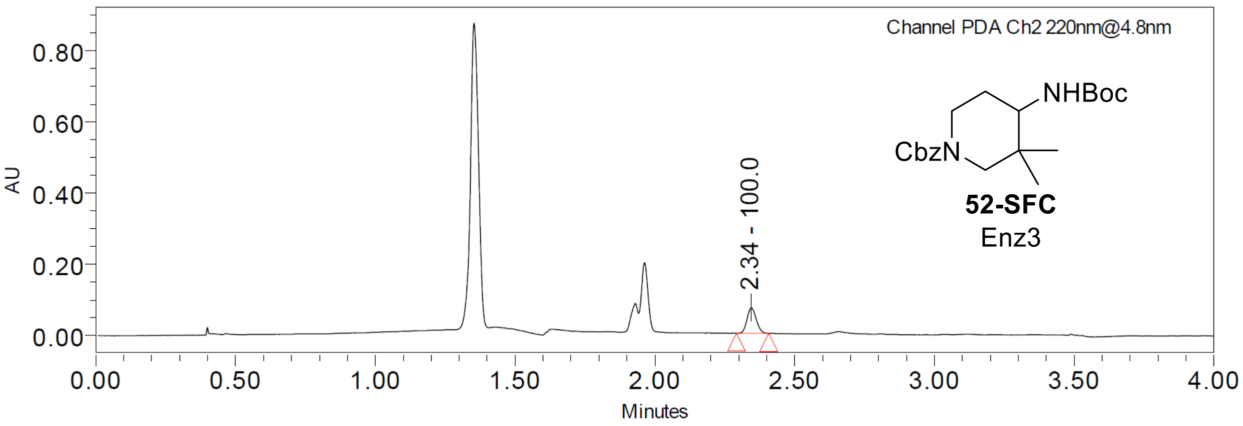

Channel PDA Ch2 220nm@4.8nm
NHBoc
CbzN
52-SFC
Enz3
2.34 - 100.0
AU
Minutes

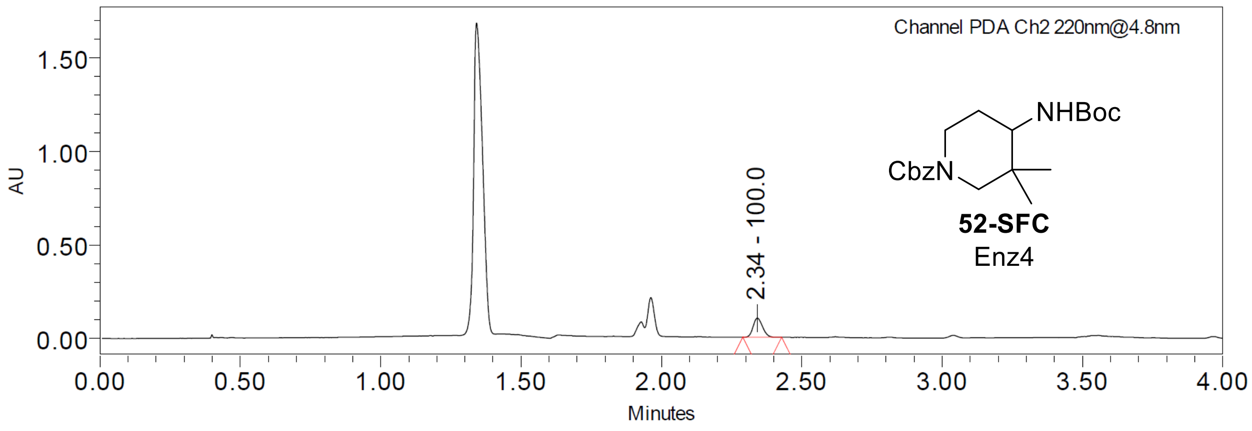

Channel PDA Ch2 220nm@4.8nm
NHBoc
CbzN
52-SFC
Enz4
2.34 - 100.0
AU
1.50
1.00
0.50
0.00
0.00
0.50
1.00
1.50
2.00
2.50
3.00
3.50
4.00
Minutes

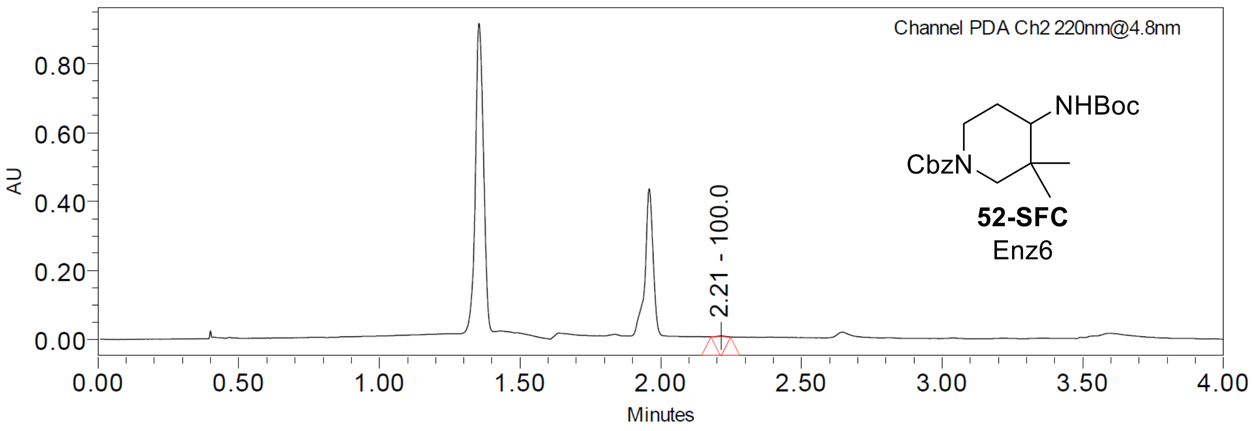

Channel PDA Ch2 220nm@4.8nm
NHBoc
CbzN
52-SFC
Enz6
2.21 - 100.0
AU
0.80
0.60
0.40
0.20
0.00
0.00
0.50
1.00
1.50
2.00
2.50
3.00
3.50
4.00
Minutes

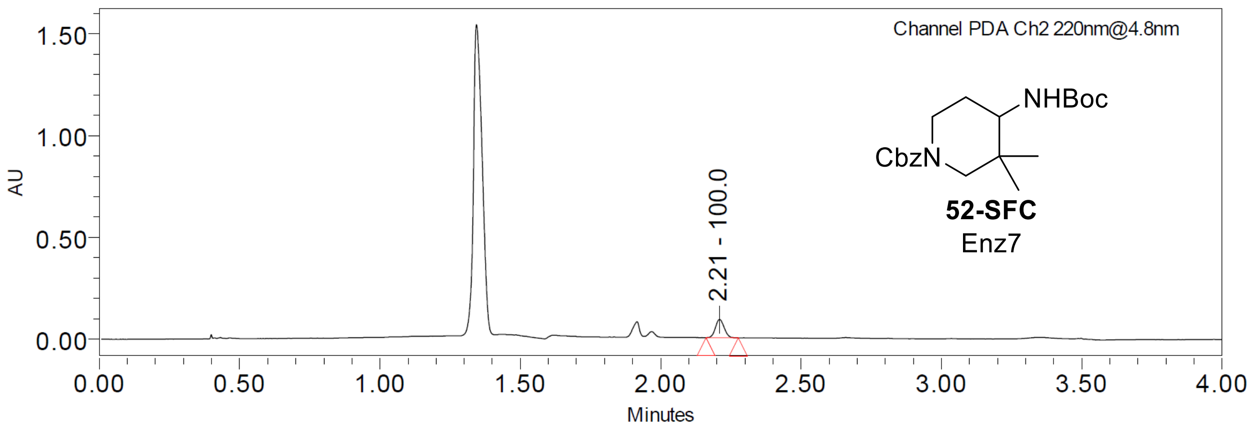

Channel PDA Ch2 220nm@4.8nm
NHBoc
CbzN
52-SFC
Enz7
2.21 - 100.0
AU
1.50
1.00
0.50
0.00
0.00
0.50
1.00
1.50
2.00
2.50
3.00
3.50
4.00
Minutes

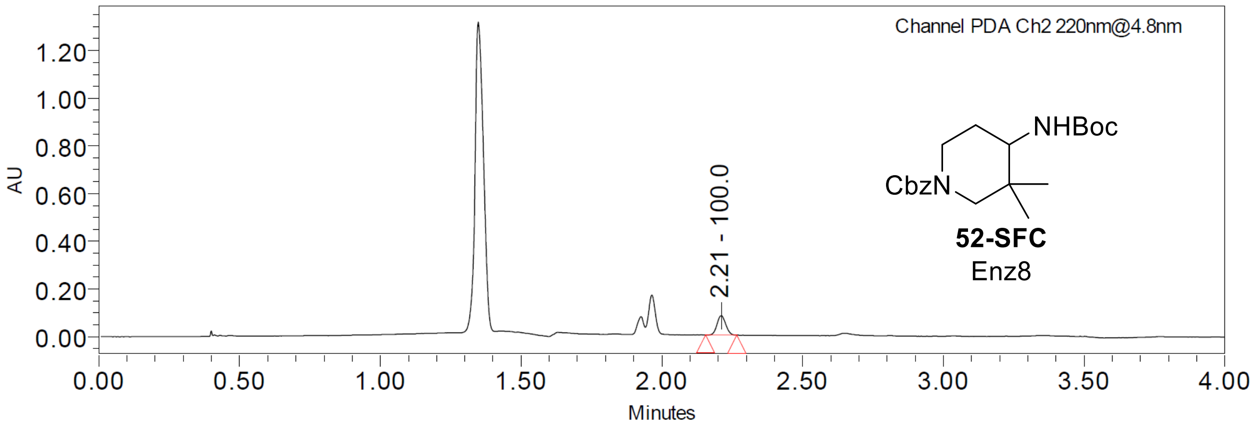
Channel PDA Ch2 220nm@4.8nm
2.21 - 100.0
NHBoc
CbzN
52-SFC
Enz8
AU
Minutes

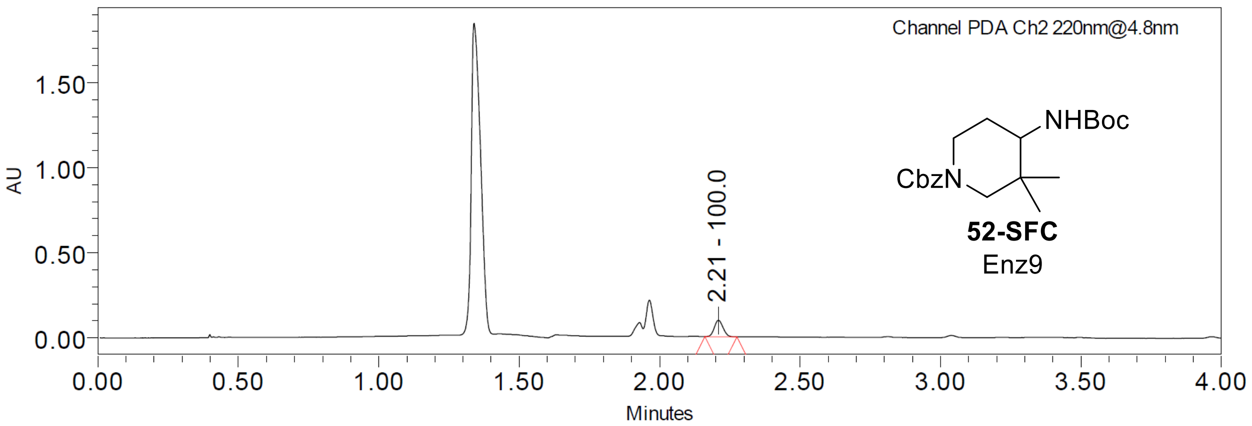
Channel PDA Ch2 220nm@4.8nm
2.21 - 100.0
NHBoc
CbzN
52-SFC
Enz9
AU
Minutes

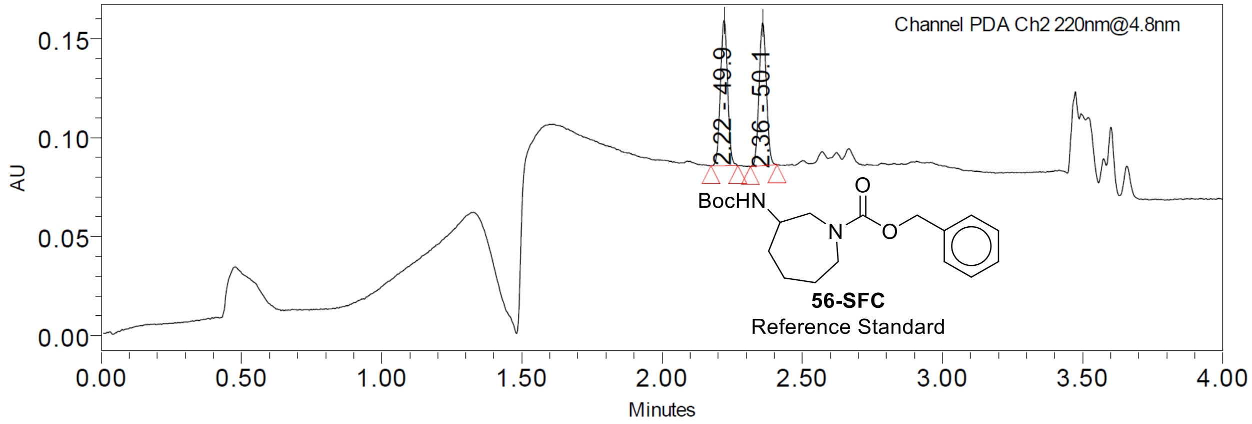
Channel PDA Ch2 220nm@4.8nm
2.22 - 49.9
2.36 - 50.1
BocHN
N
O
O
56-SFC
Reference Standard
AU
Minutes

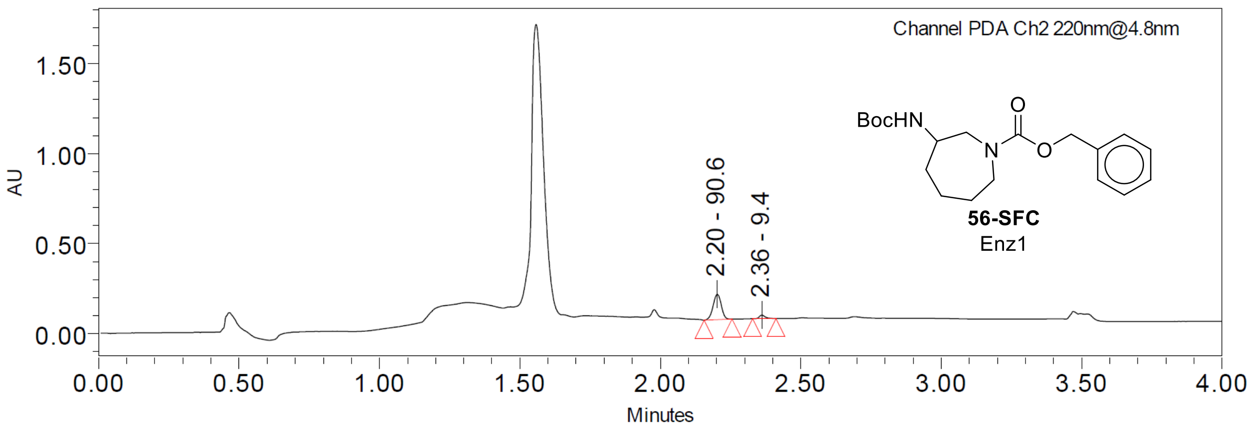
Channel PDA Ch2 220nm@4.8nm
BocHN
N
O
O
56-SFC
Enz1
2.20 - 90.6
2.36 - 9.4
AU
Minutes

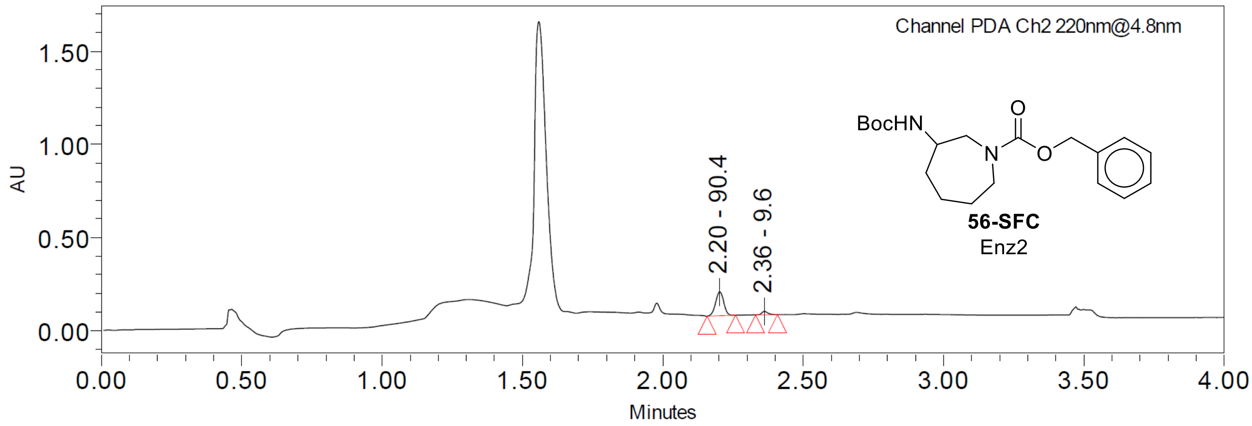
Channel PDA Ch2 220nm@4.8nm
BocHN
N
O
O
56-SFC
Enz2
2.20 - 90.4
2.36 - 9.6
AU
Minutes

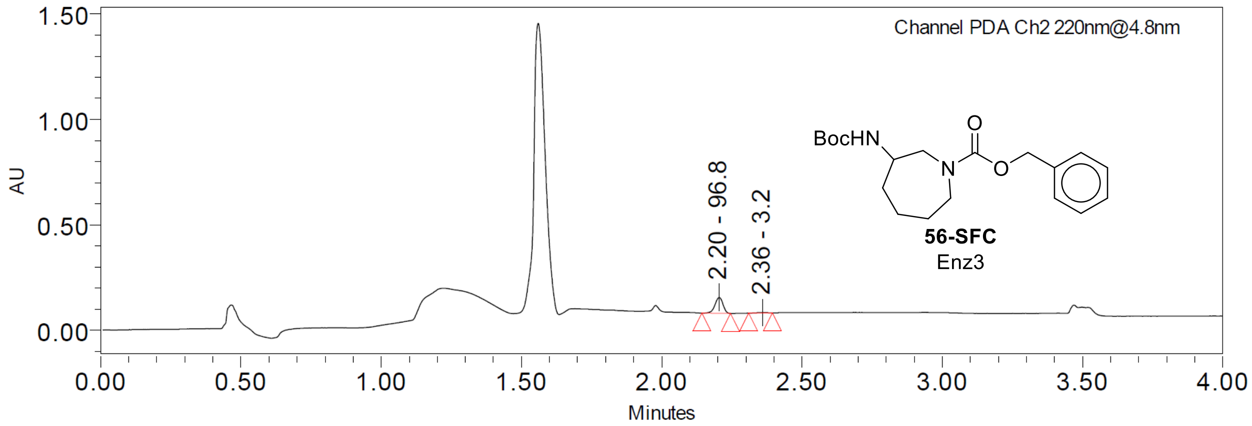
Channel PDA Ch2 220nm@4.8nm
BocHN
N
O
O
56-SFC
Enz3
2.20 - 96.8
2.36 - 3.2
AU
Minutes

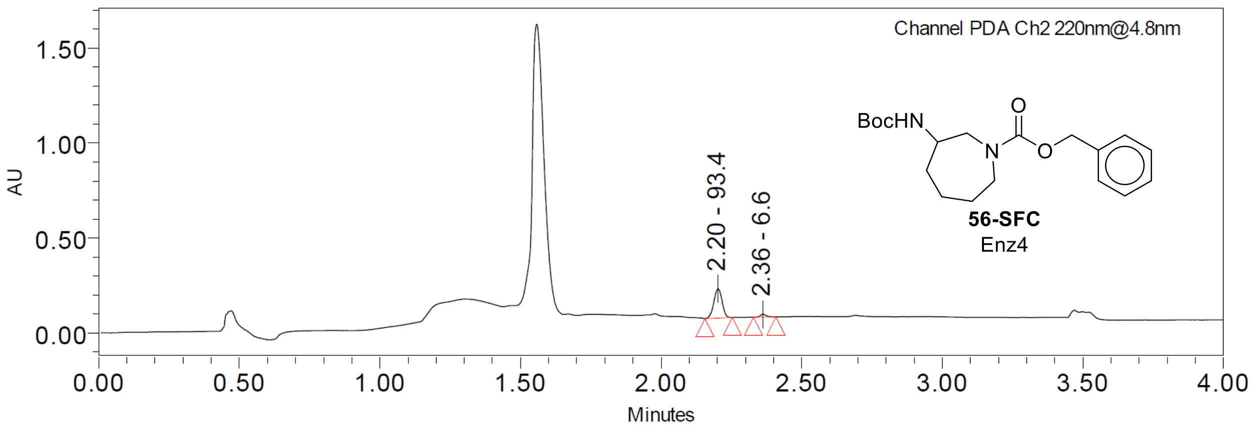
Channel PDA Ch2 220nm@4.8nm
BocHN
O
N
O
56-SFC
Enz4
2.20 - 93.4
2.36 - 6.6
AU
1.50
1.00
0.50
0.00
0.00
0.50
1.00
1.50
2.00
2.50
3.00
3.50
4.00
Minutes

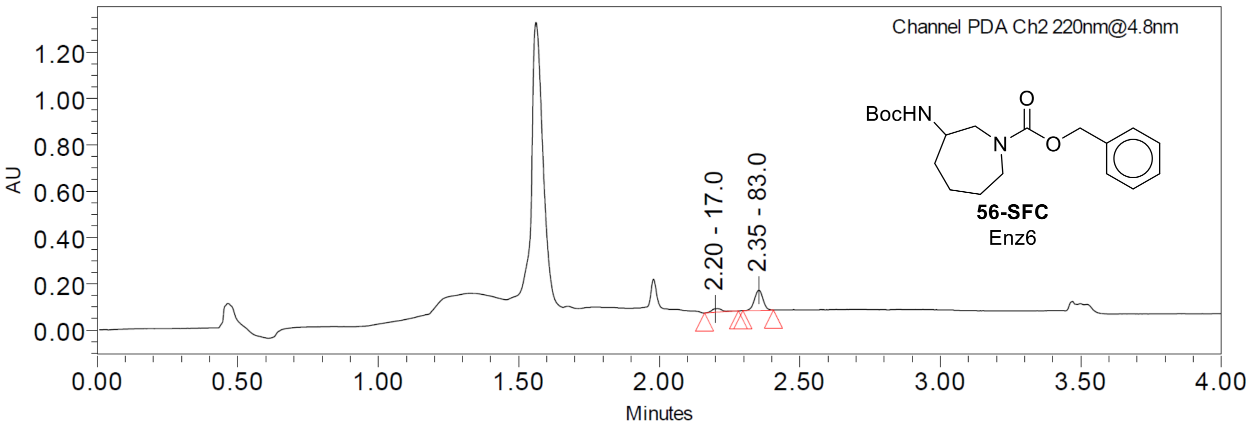
Channel PDA Ch2 220nm@4.8nm
BocHN
O
N
O
56-SFC
Enz6
2.20 - 17.0
2.35 - 83.0
AU
1.20
1.00
0.80
0.60
0.40
0.20
0.00
0.00
0.50
1.00
1.50
2.00
2.50
3.00
3.50
4.00
Minutes

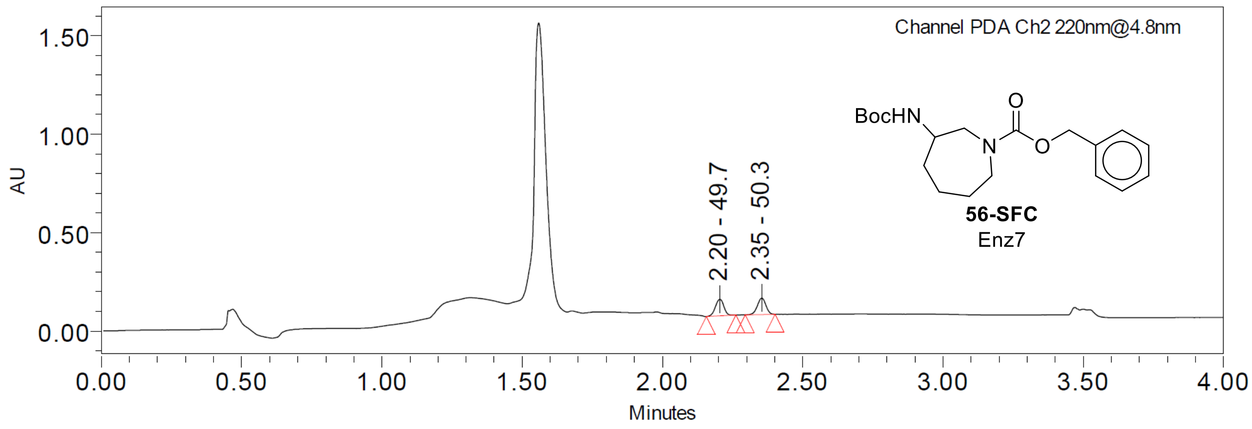
Channel PDA Ch2 220nm@4.8nm
BocHN
O
N
O
56-SFC
Enz7
2.20 - 49.7
2.35 - 50.3
AU
1.50
1.00
0.50
0.00
0.00
0.50
1.00
1.50
2.00
2.50
3.00
3.50
4.00
Minutes

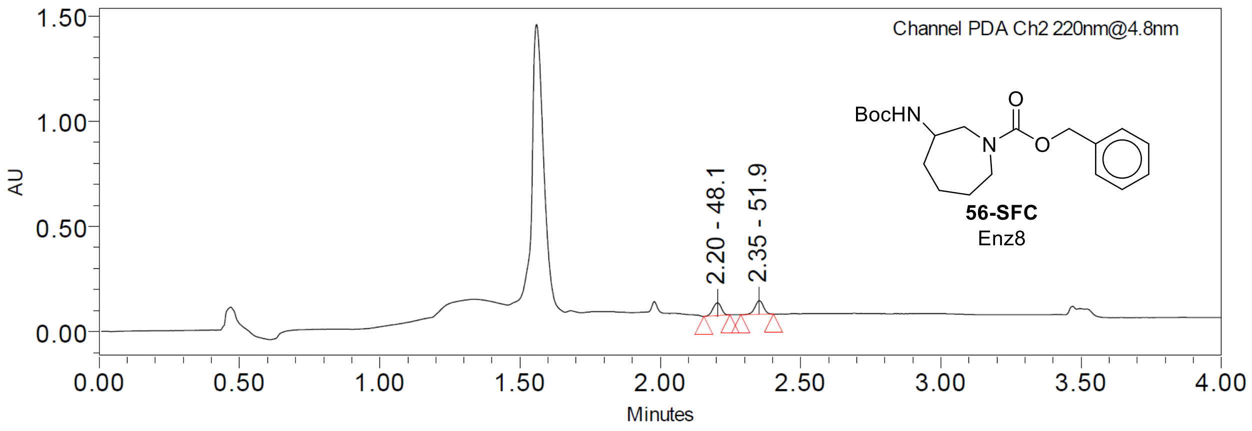
Channel PDA Ch2 220nm@4.8nm
BocHN
N
O
O
56-SFC
Enz8
2.20 - 48.1
2.35 - 51.9
AU
Minutes

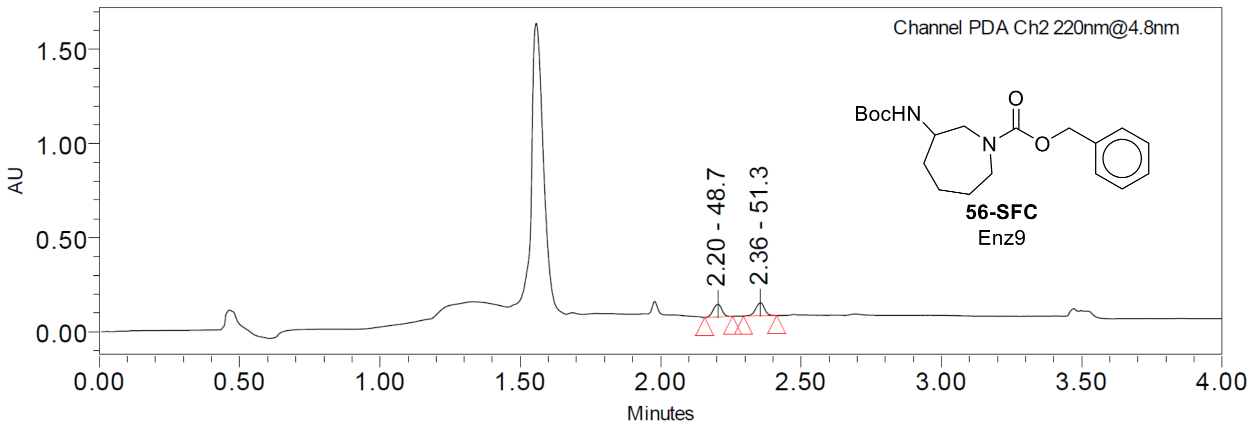
Channel PDA Ch2 220nm@4.8nm
BocHN
N
O
O
56-SFC
Enz9
2.20 - 48.7
2.36 - 51.3
AU
Minutes

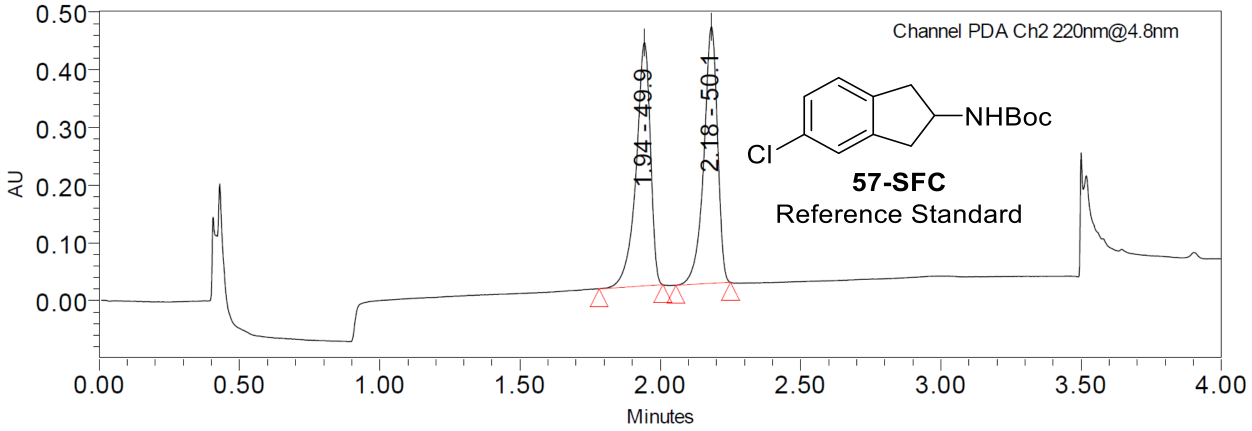
Channel PDA Ch2 220nm@4.8nm
Cl
NHBoc
57-SFC
Reference Standard
1.94 - 49.9
2.18 - 50.1
AU
Minutes

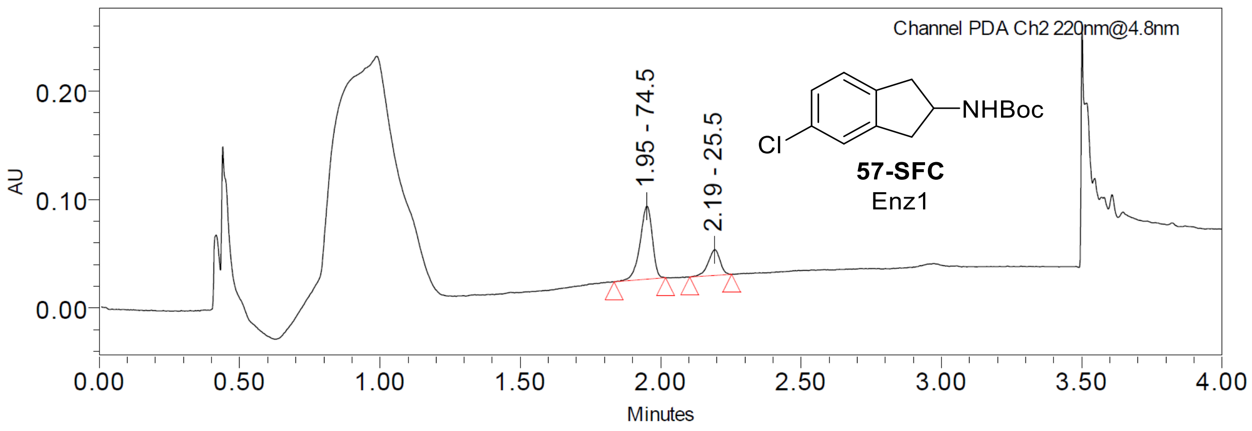
Channel PDA Ch2 220nm@4.8nm
1.95 - 74.5
2.19 - 25.5
Cl
NHBoc
57-SFC
Enz1
AU
Minutes

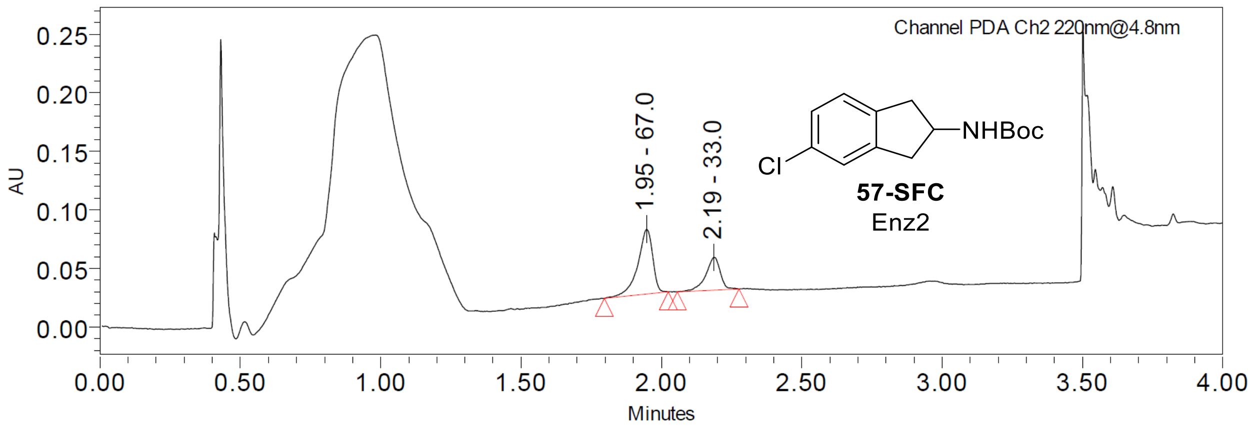
Channel PDA Ch2 220nm@4.8nm
1.95 - 67.0
2.19 - 33.0
Cl
NHBoc
57-SFC
Enz2
AU
Minutes

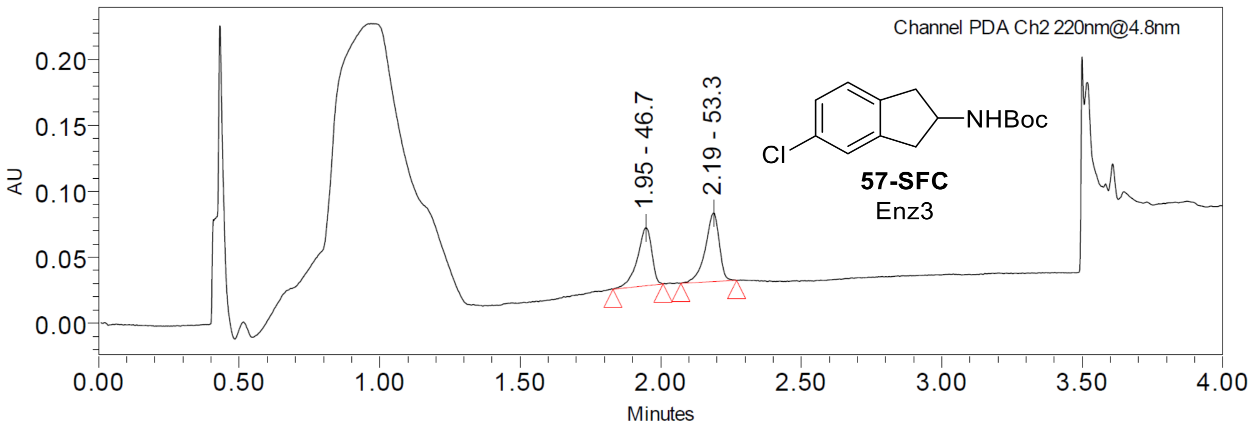
Channel PDA Ch2 220nm@4.8nm
1.95 - 46.7
2.19 - 53.3
Cl
NHBoc
57-SFC
Enz3
AU
Minutes

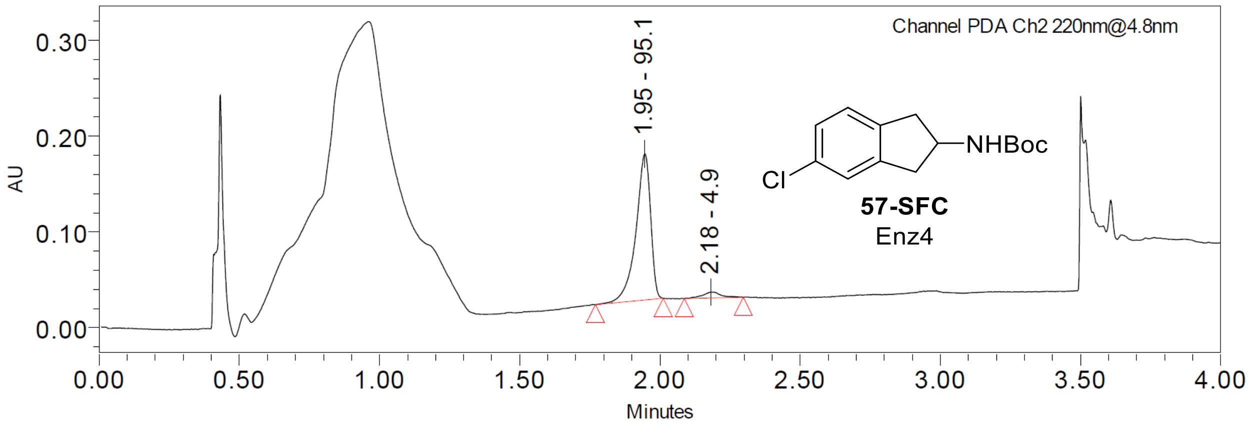
Channel PDA Ch2 220nm@4.8nm
1.95 - 95.1
2.18 - 4.9
Cl
NHBoc
57-SFC
Enz4
AU
Minutes

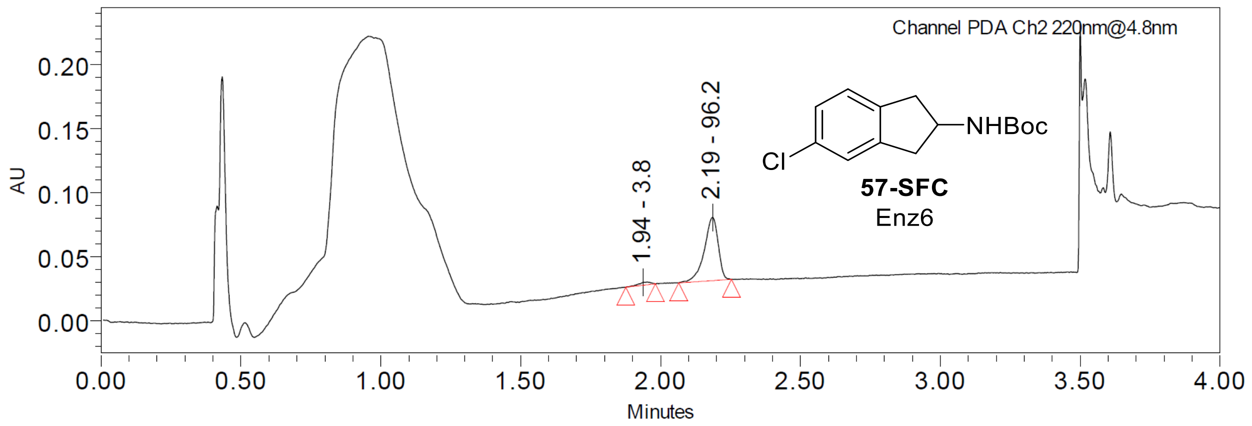
Channel PDA Ch2 220nm@4.8nm
1.94 - 3.8
2.19 - 96.2
Cl
NHBoc
57-SFC
Enz6
AU
Minutes

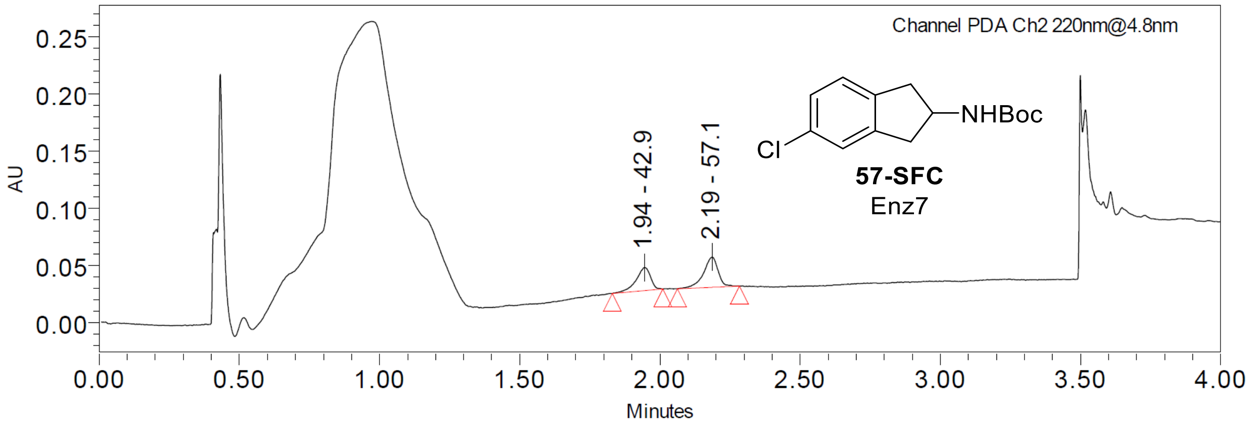
Channel PDA Ch2 220nm@4.8nm
1.94 - 42.9
2.19 - 57.1
Cl
NHBoc
57-SFC
Enz7
AU
Minutes

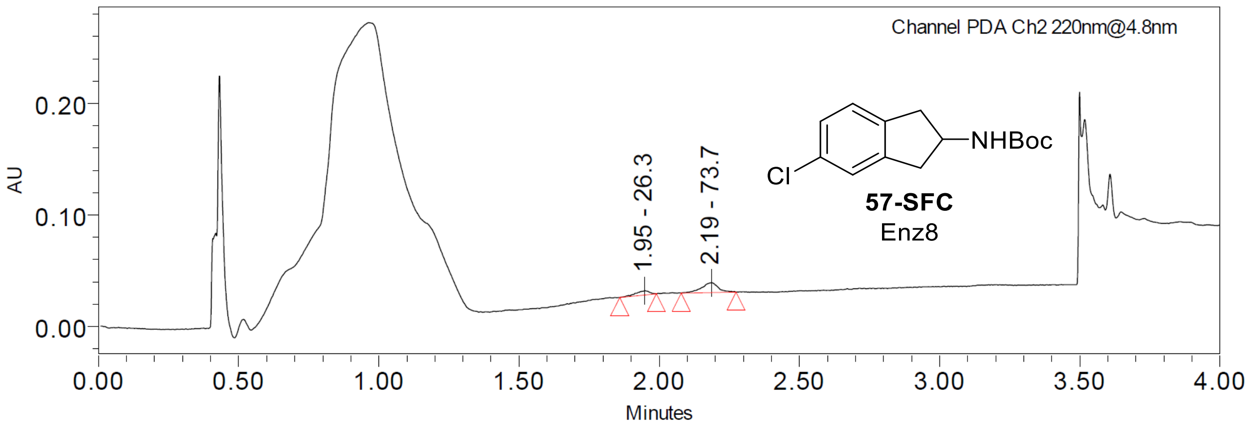
Channel PDA Ch2 220nm@4.8nm
AU
0.20
0.10
0.00
1.95 - 26.3
2.19 - 73.7
Cl
NHBoc
57-SFC
Enz8
0.00
0.50
1.00
1.50
2.00
2.50
3.00
3.50
4.00
Minutes

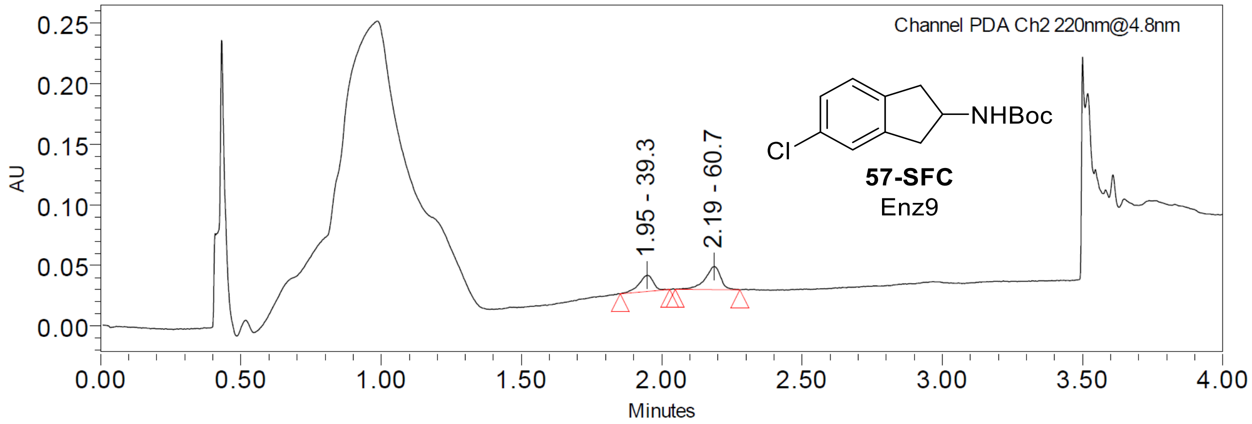
Channel PDA Ch2 220nm@4.8nm
AU
0.25
0.20
0.15
0.10
0.05
0.00
1.95 - 39.3
2.19 - 60.7
Cl
NHBoc
57-SFC
Enz9
0.00
0.50
1.00
1.50
2.00
2.50
3.00
3.50
4.00
Minutes

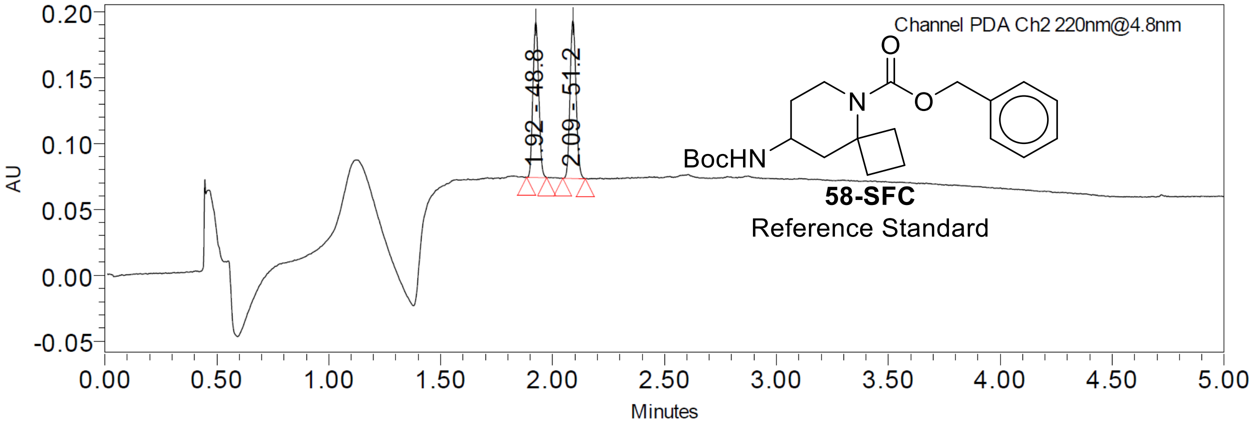
Channel PDA Ch2 220nm@4.8nm
AU
0.20
0.15
0.10
0.05
0.00
-0.05
1.92 - 48.8
2.09 - 51.2
BocHN
N
O
O
58-SFC
Reference Standard
0.00
0.50
1.00
1.50
2.00
2.50
3.00
3.50
4.00
4.50
5.00
Minutes

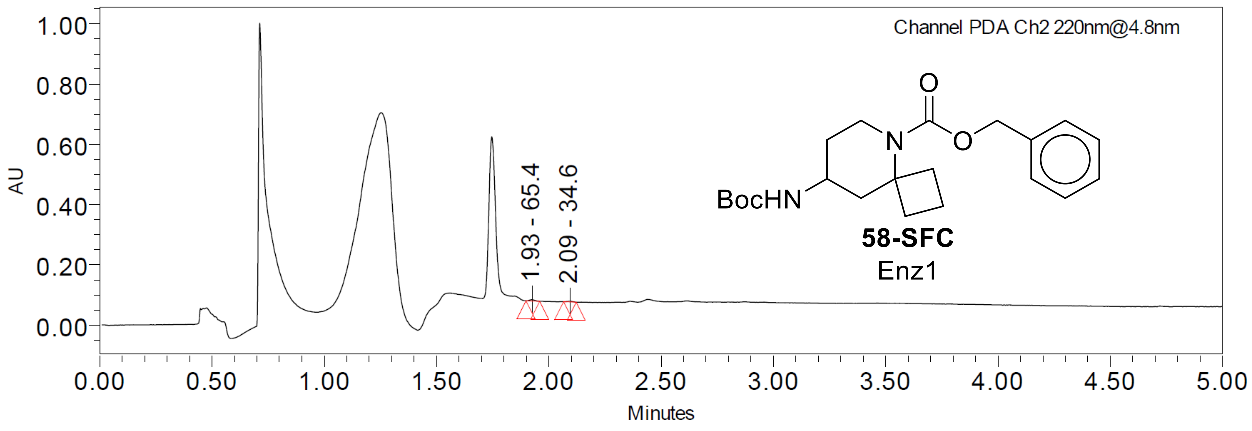
Channel PDA Ch2 220nm@4.8nm
1.93 - 65.4
2.09 - 34.6
BocHN
N
O
O
58-SFC
Enz1
AU
Minutes

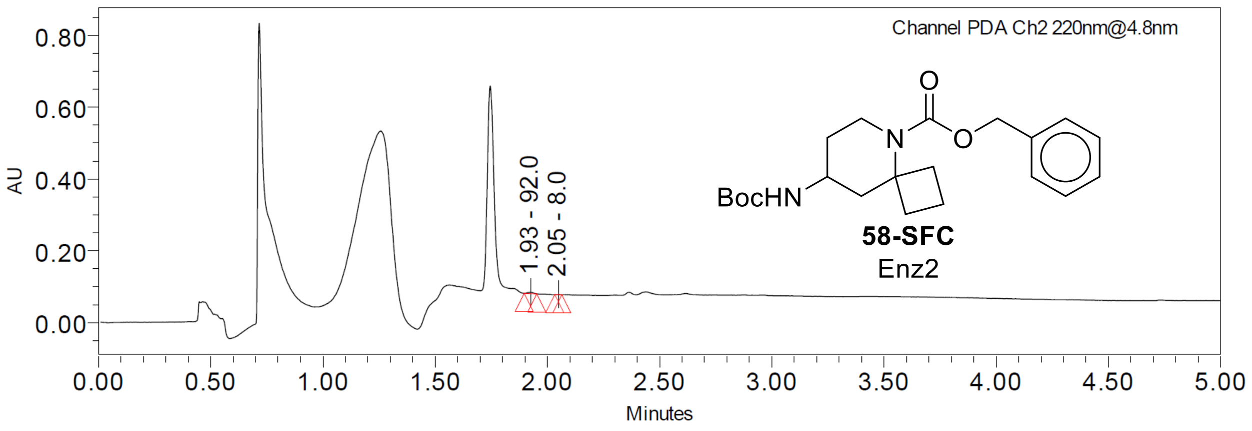
Channel PDA Ch2 220nm@4.8nm
1.93 - 92.0
2.05 - 8.0
BocHN
N
O
O
58-SFC
Enz2
AU
Minutes

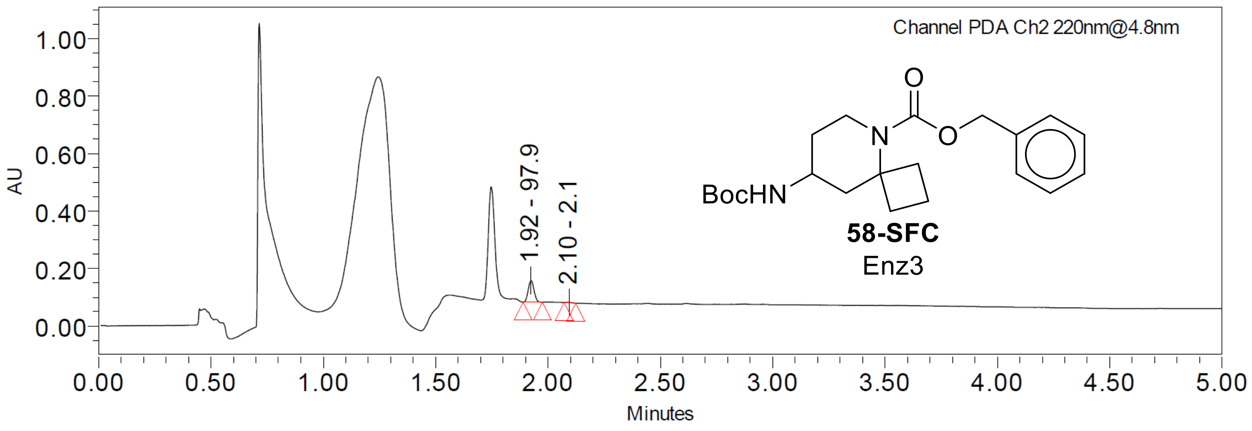
Channel PDA Ch2 220nm@4.8nm
1.92 - 97.9
2.10 - 2.1
BocHN
N
O
O
58-SFC
Enz3
AU
Minutes

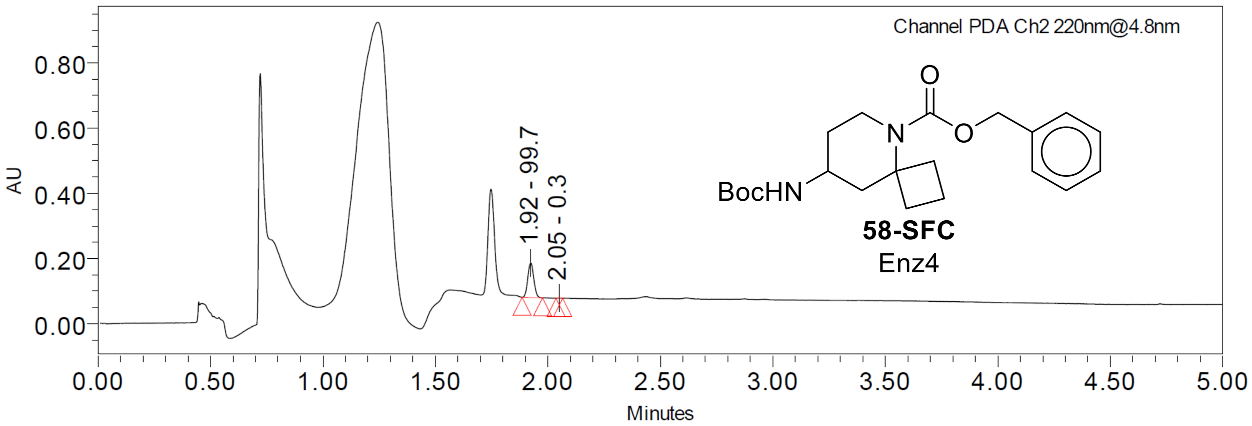
Channel PDA Ch2 220nm@4.8nm
1.92 - 99.7
2.05 - 0.3
BocHN
58-SFC
Enz4
AU
Minutes

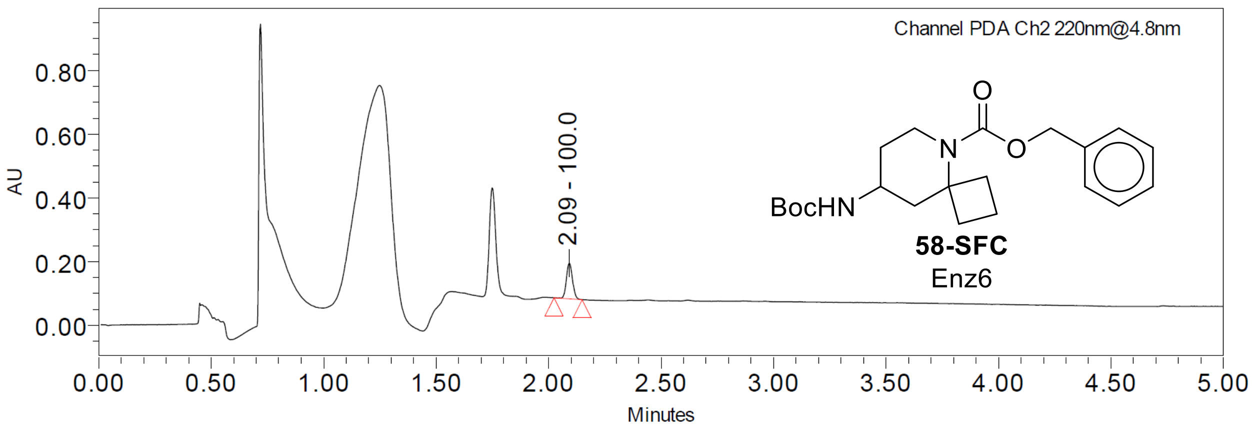
Channel PDA Ch2 220nm@4.8nm
2.09 - 100.0
BocHN
58-SFC
Enz6
AU
Minutes

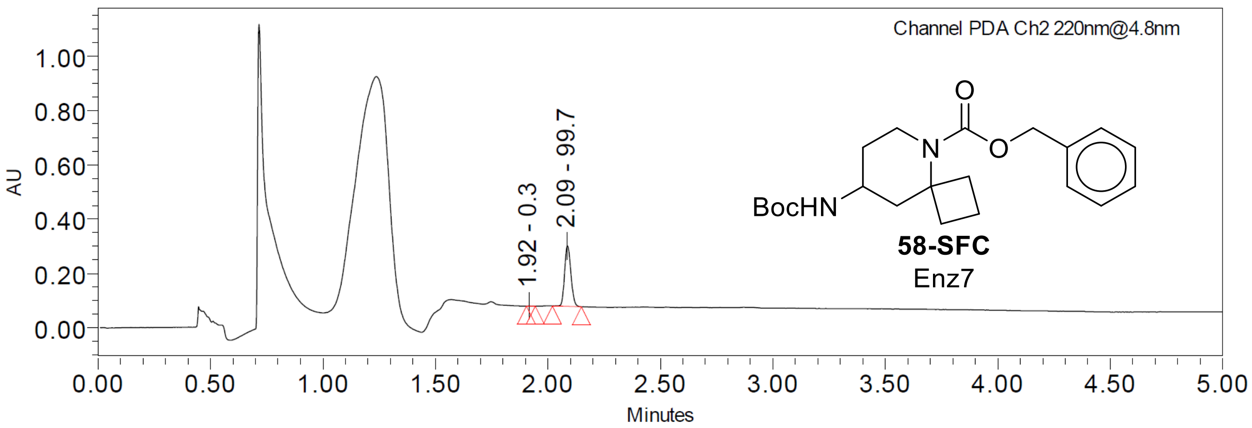
Channel PDA Ch2 220nm@4.8nm
1.92 - 0.3
2.09 - 99.7
BocHN
58-SFC
Enz7
AU
Minutes

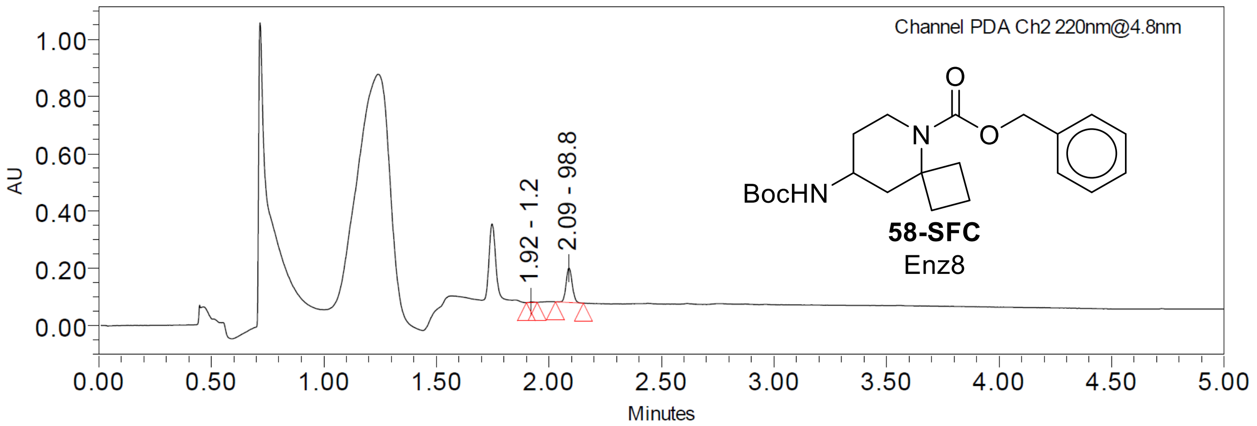
Channel PDA Ch2 220nm@4.8nm
1.92 - 1.2
2.09 - 98.8
BocHN
O
N
O
58-SFC
Enz8
AU
Minutes
1.00
0.80
0.60
0.40
0.20
0.00
0.00
0.50
1.00
1.50
2.00
2.50
3.00
3.50
4.00
4.50
5.00

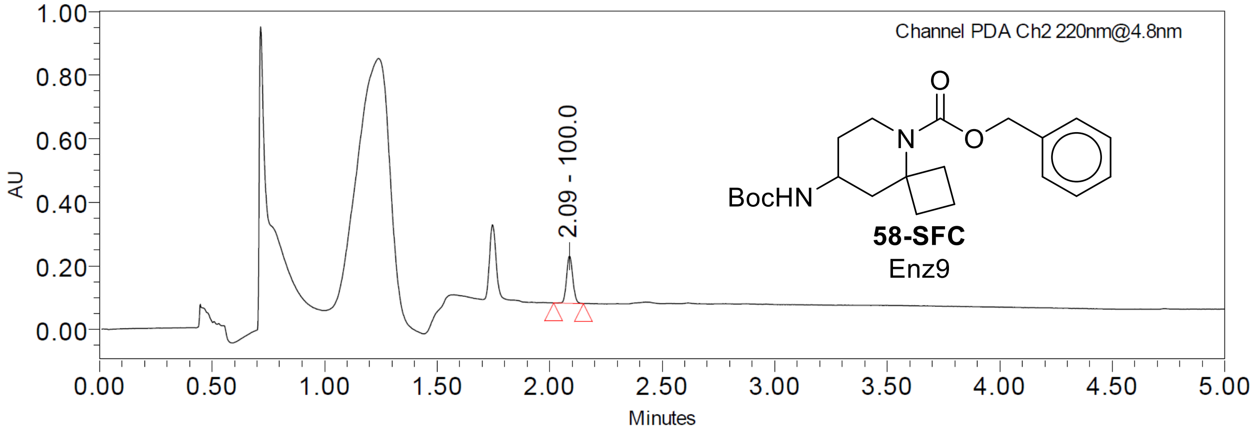
Channel PDA Ch2 220nm@4.8nm
2.09 - 100.0
BocHN
O
N
O
58-SFC
Enz9
AU
Minutes
1.00
0.80
0.60
0.40
0.20
0.00
0.00
0.50
1.00
1.50
2.00
2.50
3.00
3.50
4.00
4.50
5.00

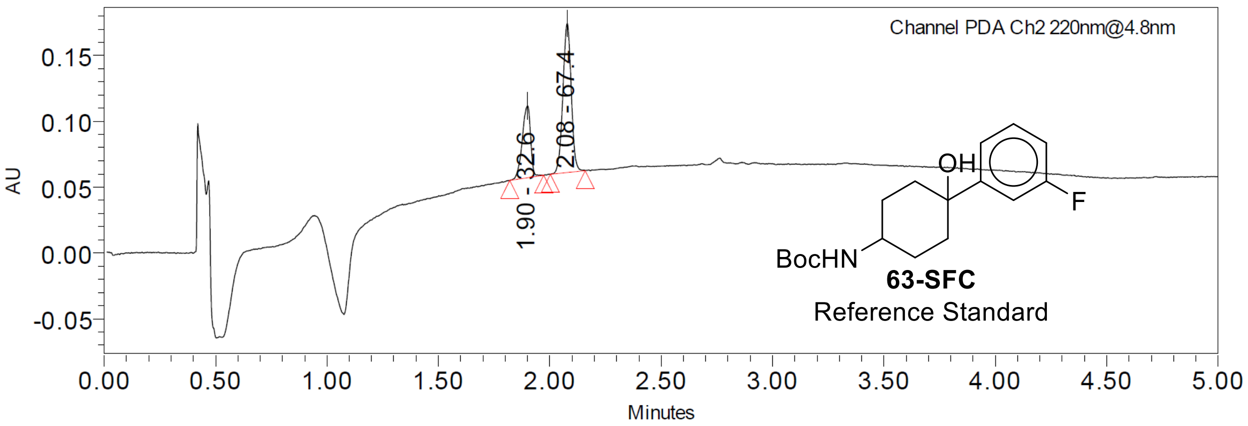
Channel PDA Ch2 220nm@4.8nm
1.90 - 32.6
2.08 - 67.4
OH
F
BocHN
63-SFC
Reference Standard
AU
Minutes
0.15
0.10
0.05
0.00
-0.05
0.00
0.50
1.00
1.50
2.00
2.50
3.00
3.50
4.00
4.50
5.00

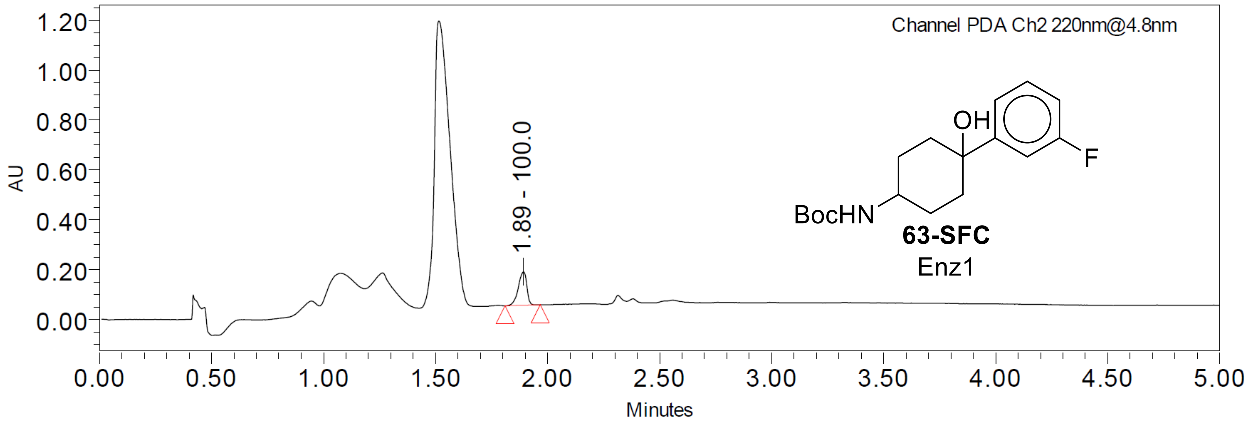
Channel PDA Ch2 220nm@4.8nm
1.89 - 100.0
OH
F
BocHN
63-SFC
Enz1
AU
Minutes

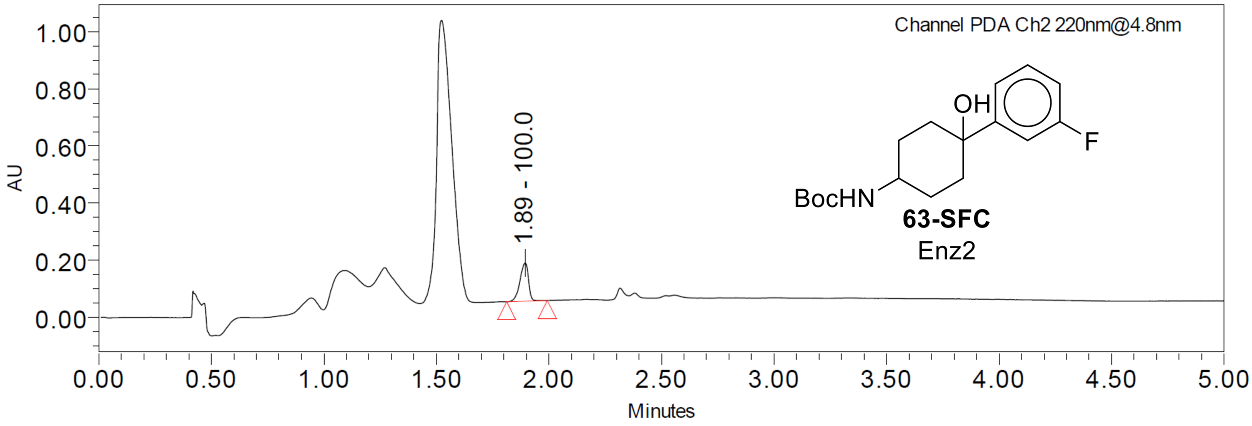
Channel PDA Ch2 220nm@4.8nm
1.89 - 100.0
OH
F
BocHN
63-SFC
Enz2
AU
Minutes

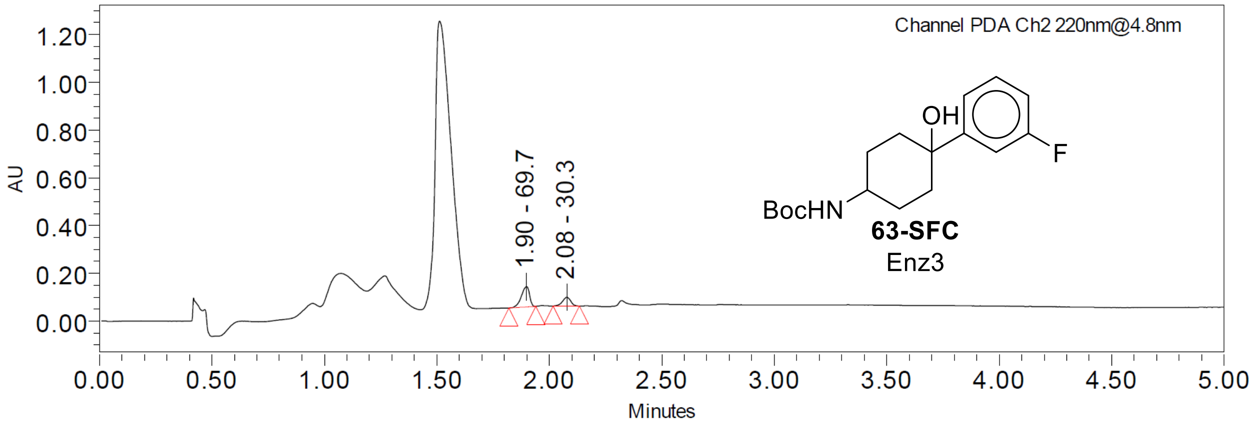
Channel PDA Ch2 220nm@4.8nm
1.90 - 69.7
2.08 - 30.3
OH
F
BocHN
63-SFC
Enz3
AU
Minutes

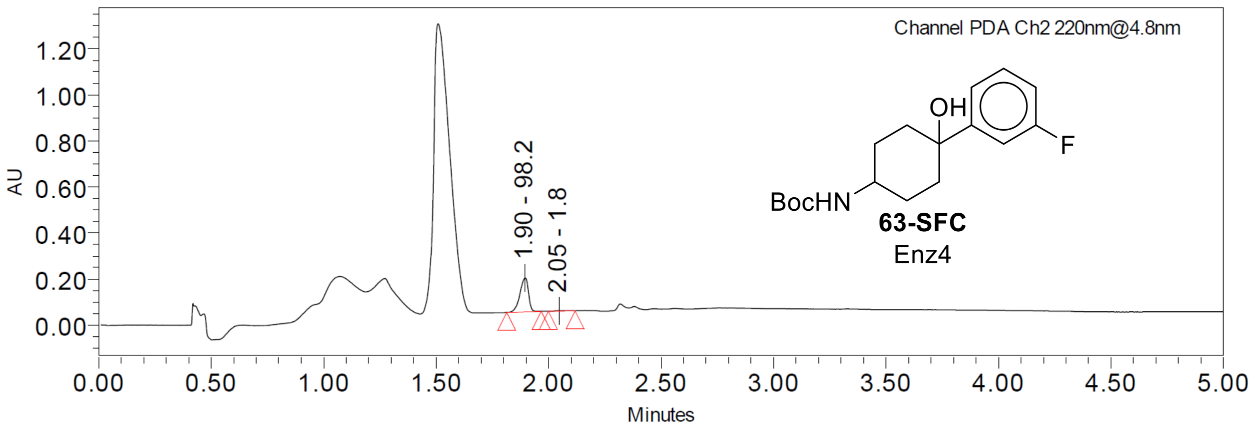
Channel PDA Ch2 220nm@4.8nm
OH
F
BocHN
63-SFC
Enz4
1.90 - 98.2
2.05 - 1.8
AU
Minutes

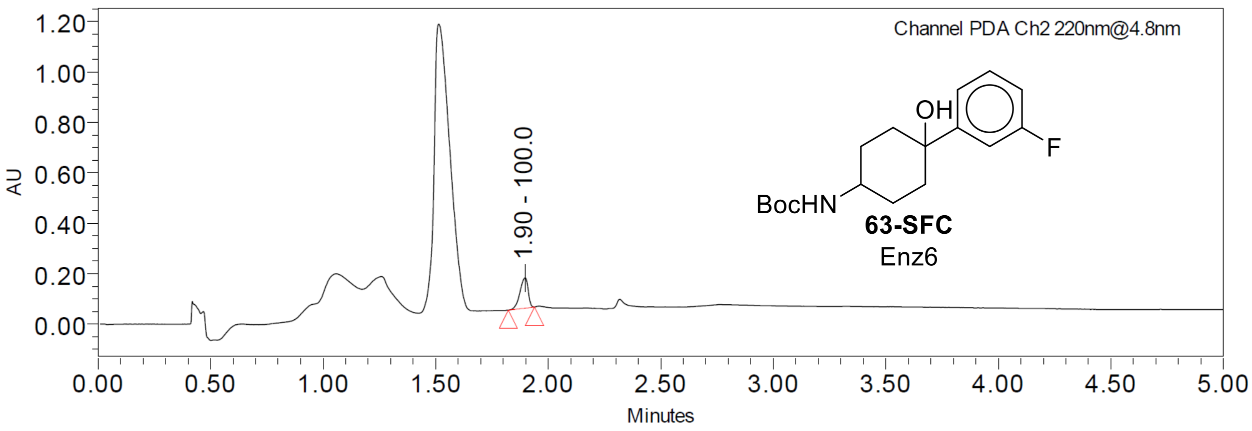
Channel PDA Ch2 220nm@4.8nm
OH
F
BocHN
63-SFC
Enz6
1.90 - 100.0
AU
Minutes

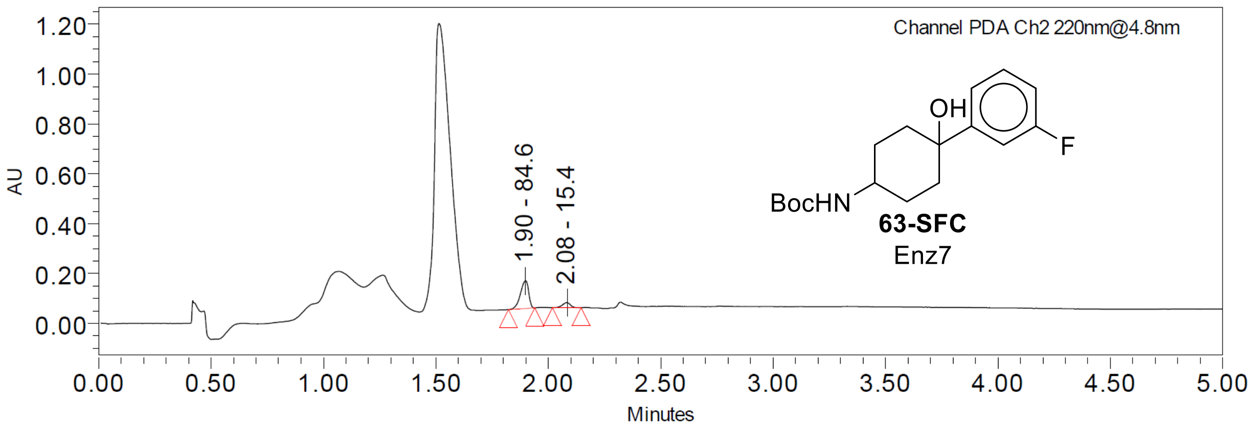
Channel PDA Ch2 220nm@4.8nm
OH
F
BocHN
63-SFC
Enz7
1.90 - 84.6
2.08 - 15.4
AU
Minutes

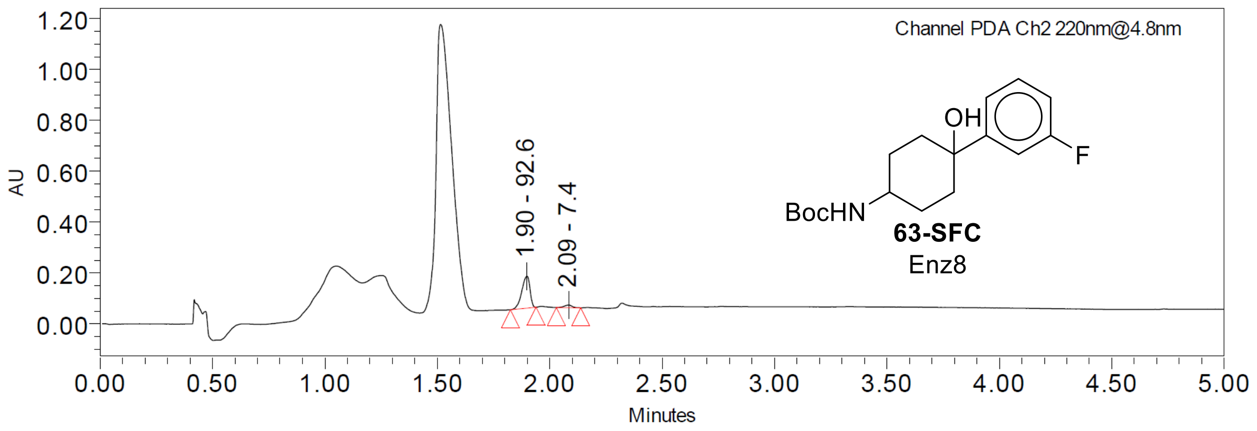
Channel PDA Ch2 220nm@4.8nm
AU
Minutes
1.90 - 92.6
2.09 - 7.4
OH
F
BocHN
63-SFC
Enz8

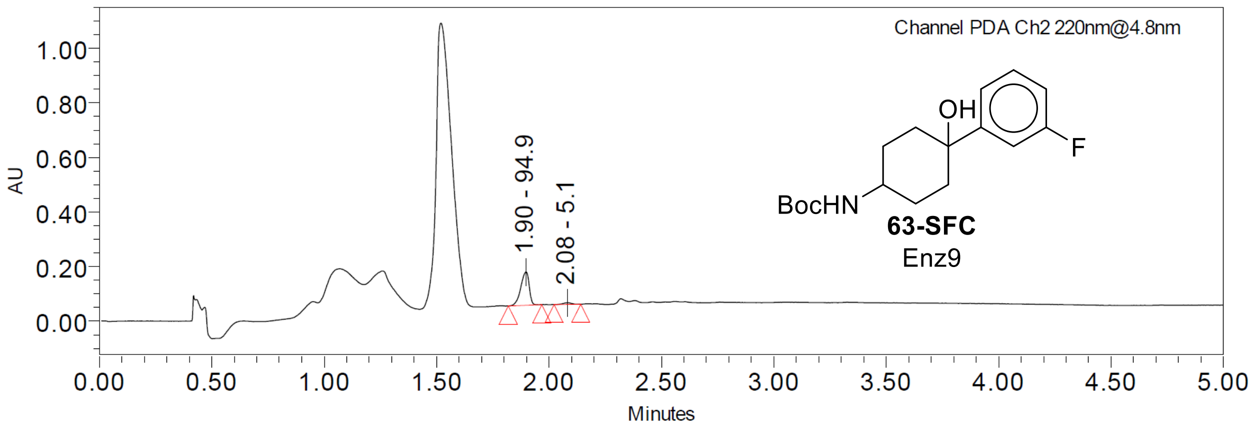
Channel PDA Ch2 220nm@4.8nm
AU
Minutes
1.90 - 94.9
2.08 - 5.1
OH
F
BocHN
63-SFC
Enz9

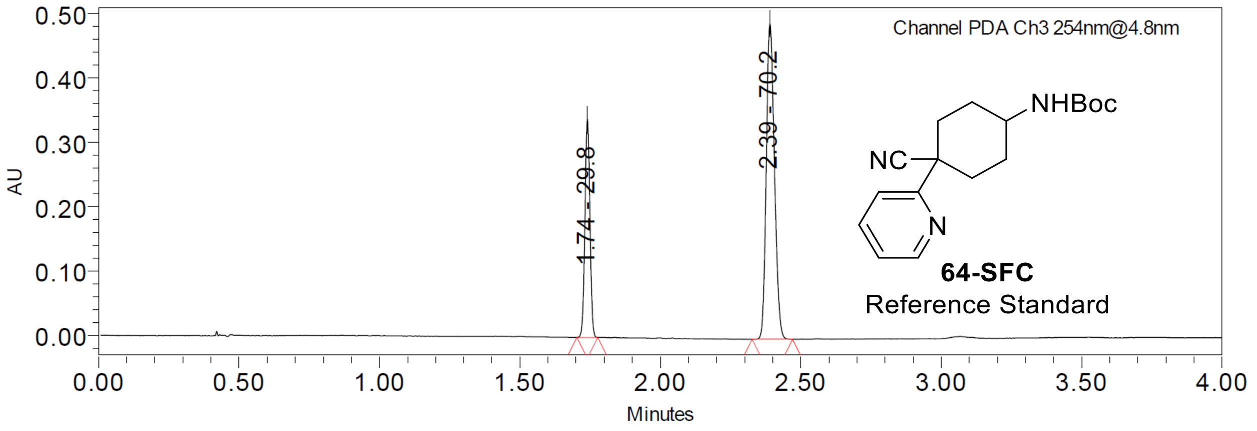
Channel PDA Ch3 254nm@4.8nm
AU
Minutes
1.74 - 29.8
2.39 - 70.2
NHBoc
NC
N
64-SFC
Reference Standard

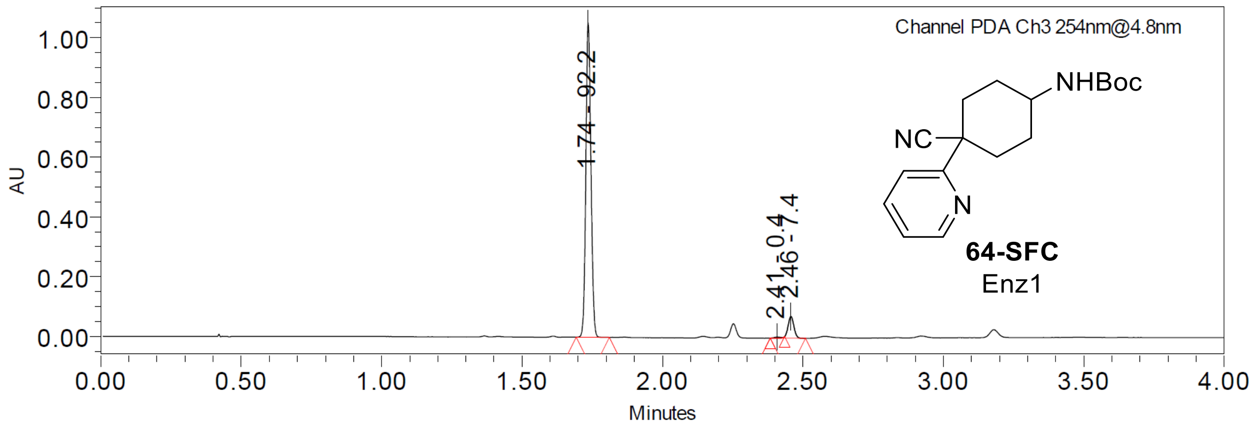

Note: Impurity present at t=2.46min

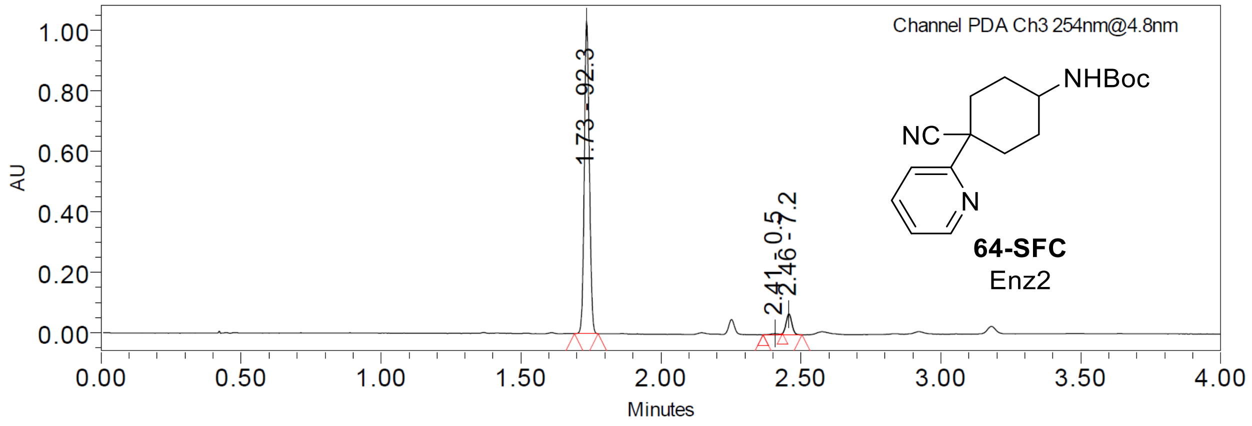

Note: Impurity present at t=2.46min

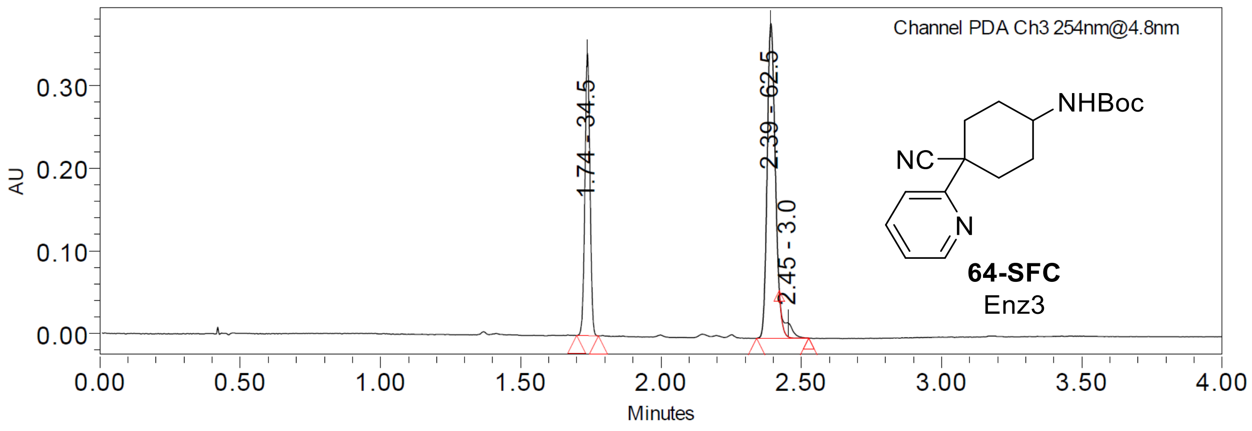

Note: Advanced integration was used for peak 2; impurity present at t=2.45min

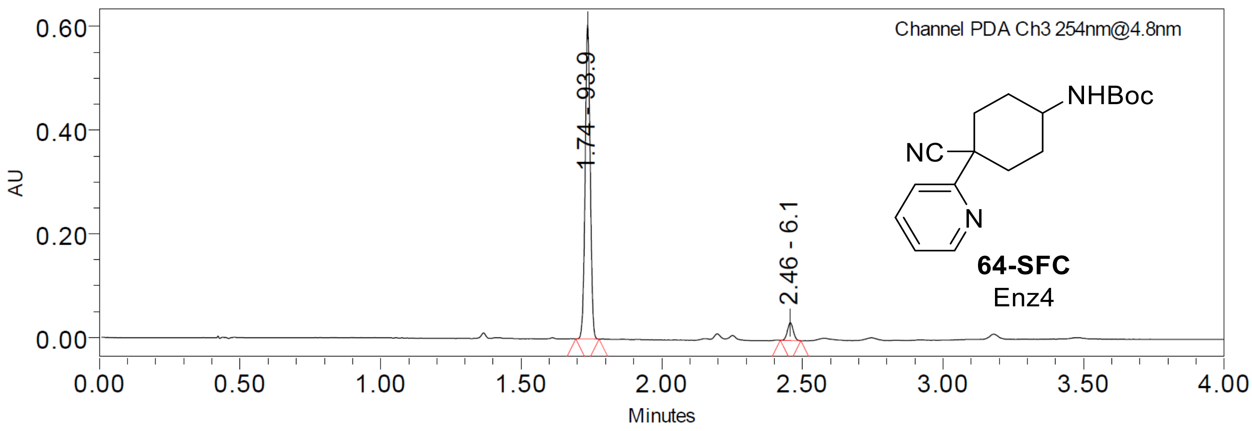

Note: Impurity present at t=2.46min

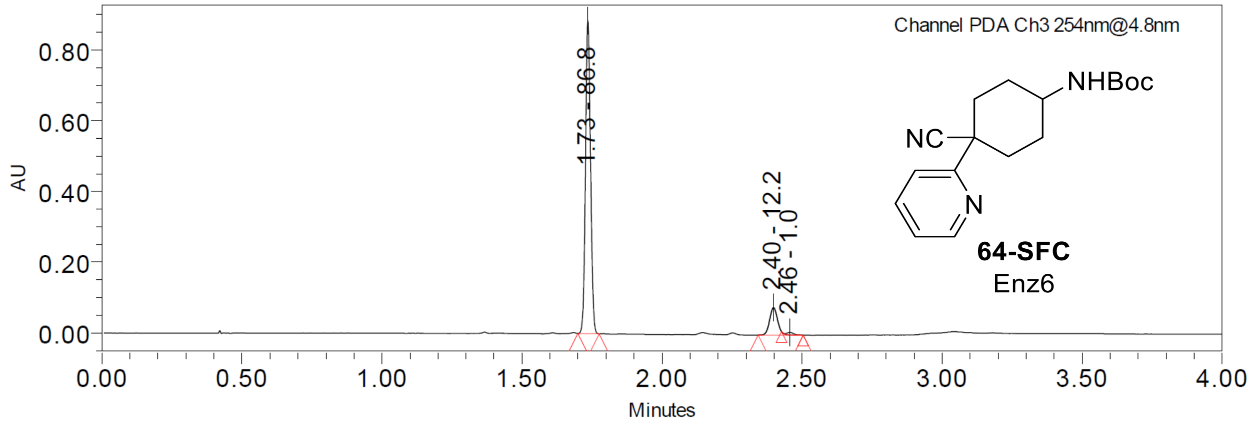

Note: Impurity present at t=2.46min

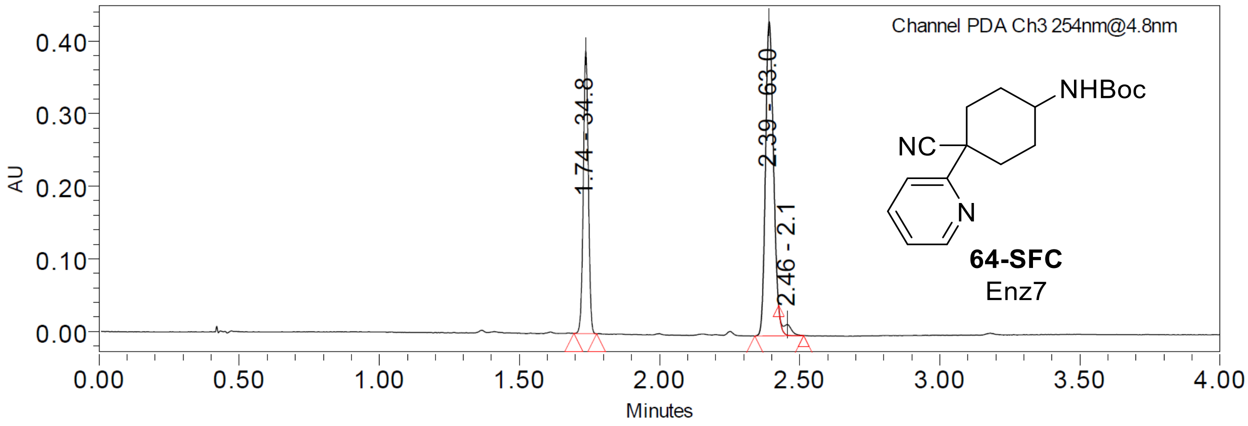

Note: Advanced integration was used for peak 2; impurity present at t=2.46min

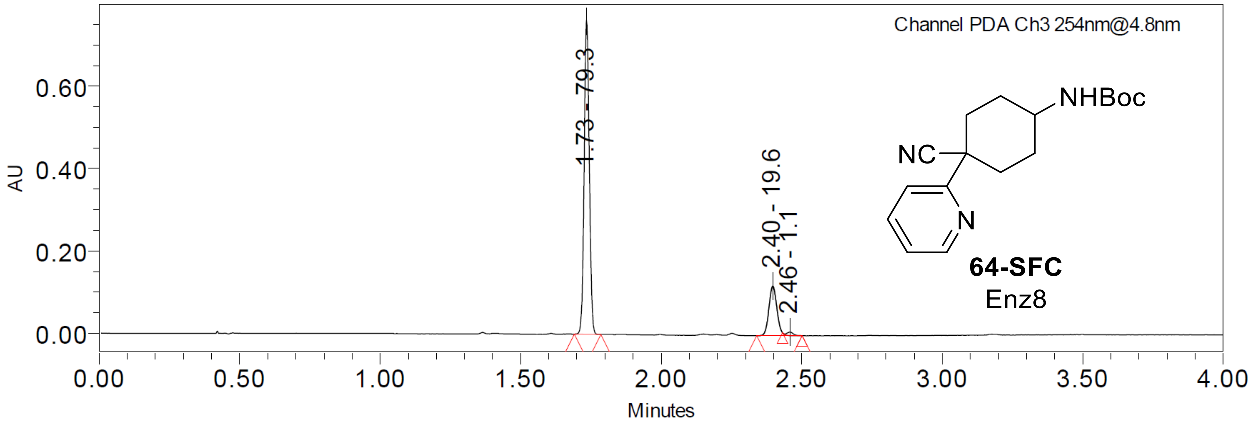

Note: Advanced integration was used for peak 2; impurity present at t=2.46min

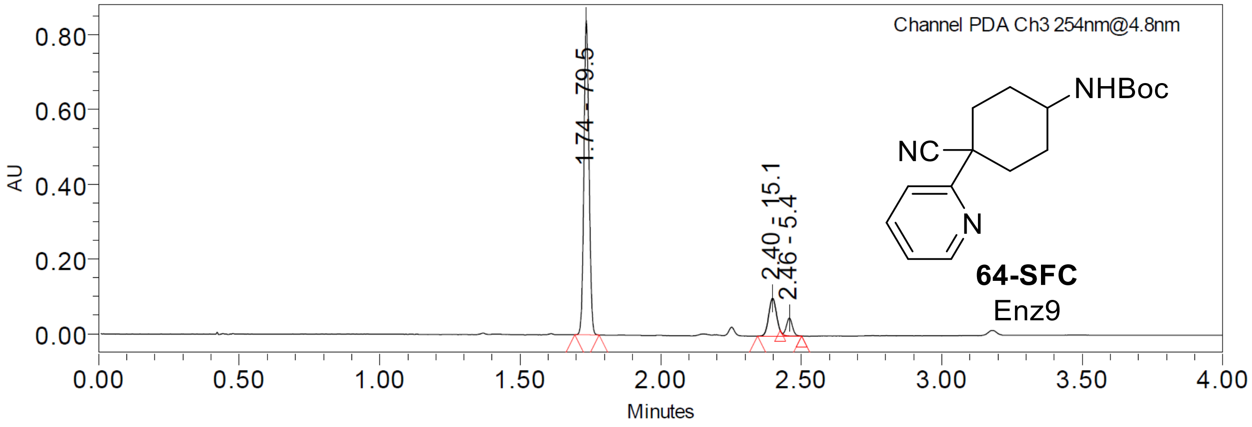

Note: Advanced integration was used for peak 2; impurity present at t=2.46min

----

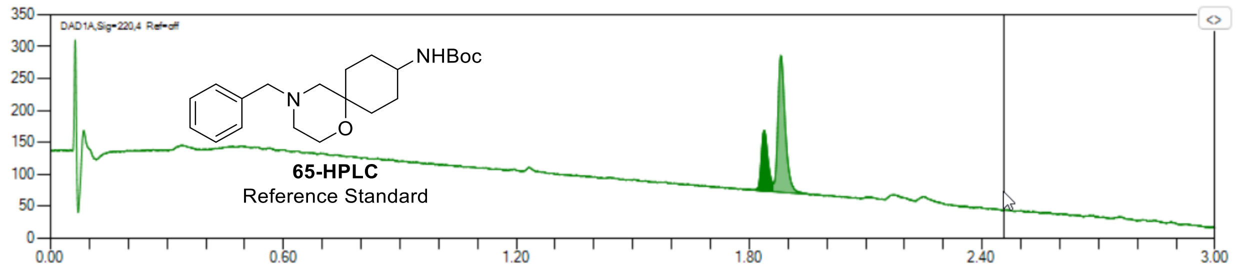

Note: Reverse-phase HPLC was used for this sample set's *de* measurements

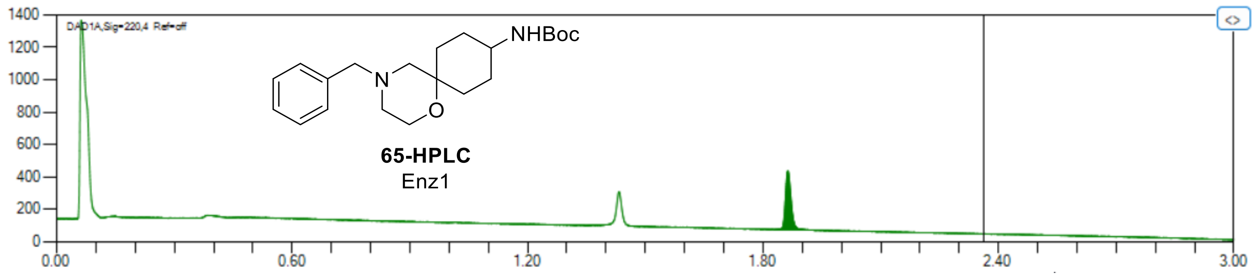
NHBoc
N
O
65-HPLC
Enz1

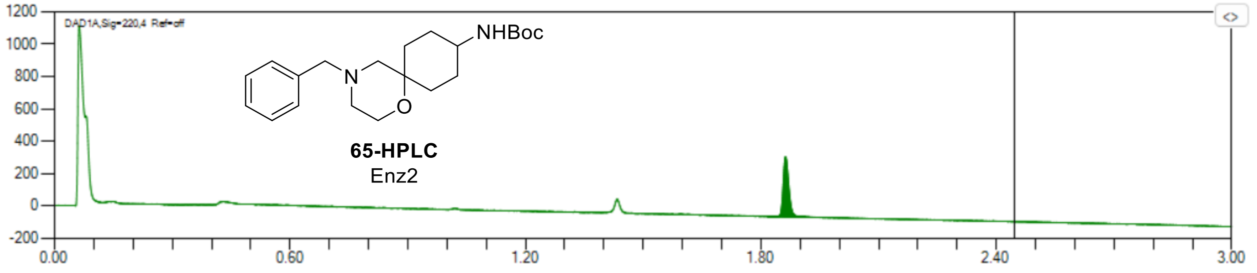
NHBoc
N
O
65-HPLC
Enz2

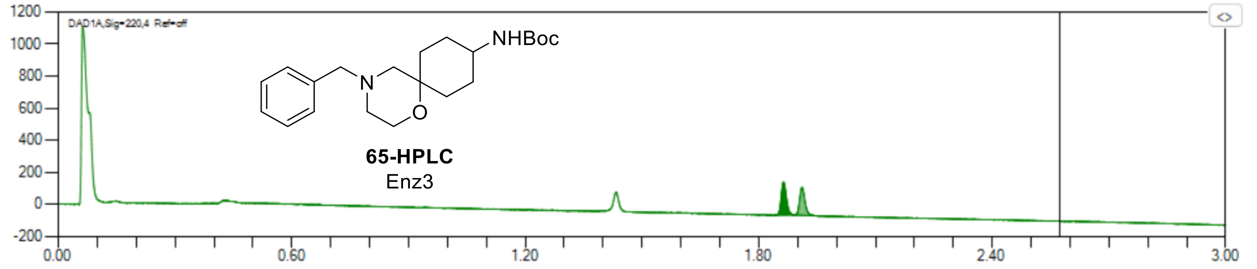
NHBoc
N
O
65-HPLC
Enz3

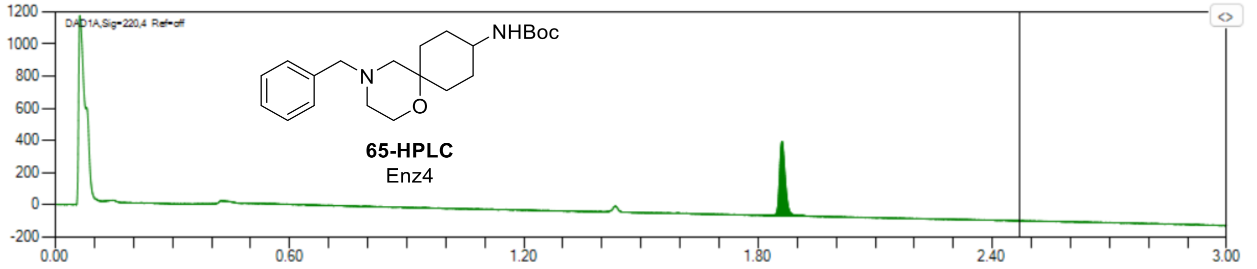
NHBoc
N
O
65-HPLC
Enz4

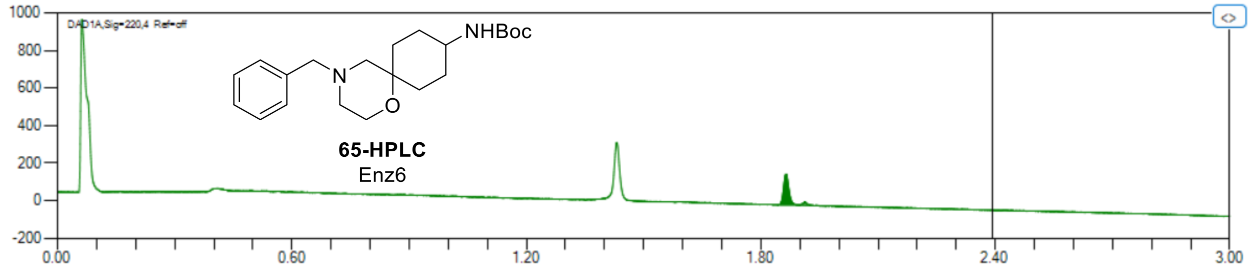
NHBoc
N
O
65-HPLC
Enz6

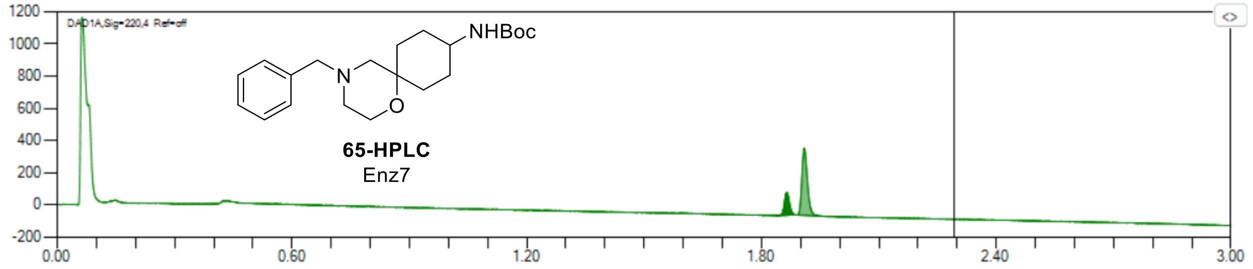

DAD1A,Sig=220,4 Ref=off
NHBoc
N
O
65-HPLC
Enz7

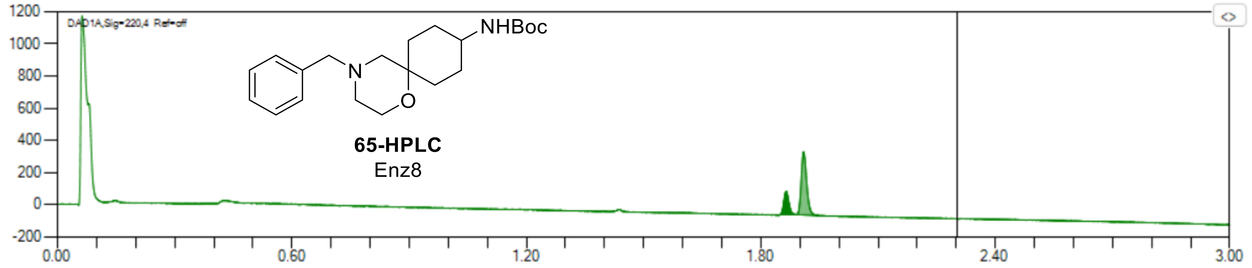

DAD1A,Sig=220,4 Ref=off
NHBoc
N
O
65-HPLC
Enz8

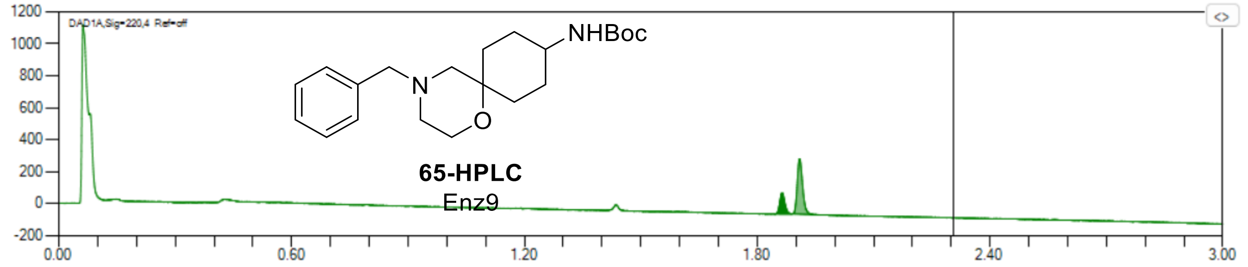

DAD1A,Sig=220,4 Ref=off
NHBoc
N
O
65-HPLC
Enz9

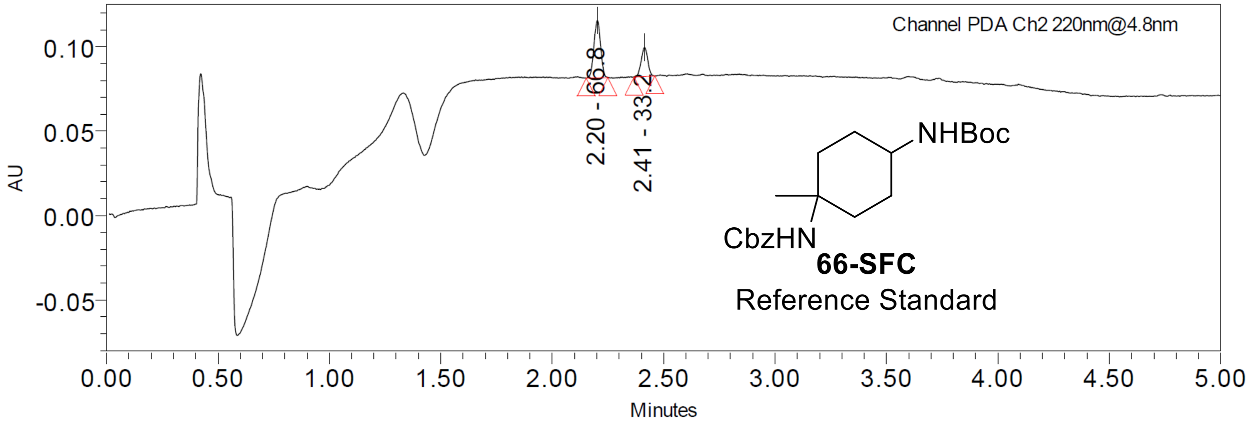

Channel PDA Ch2 220nm@4.8nm
2.20 - 66.8
2.41 - 33.2
NHBoc
CbzHN
66-SFC
Reference Standard
AU
Minutes

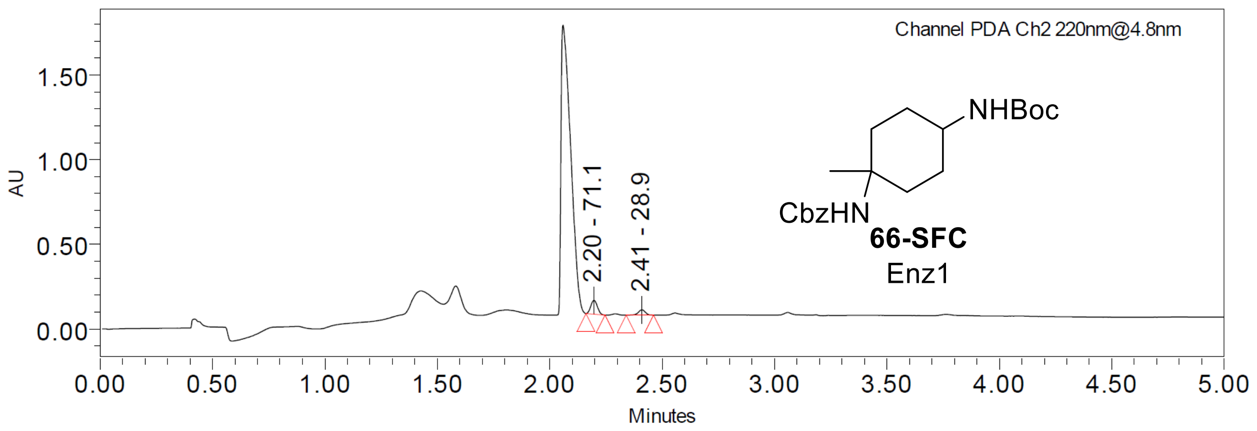
Channel PDA Ch2 220nm@4.8nm
AU
Minutes
2.20 - 71.1
2.41 - 28.9
NHBoc
CbzHN
66-SFC
Enz1

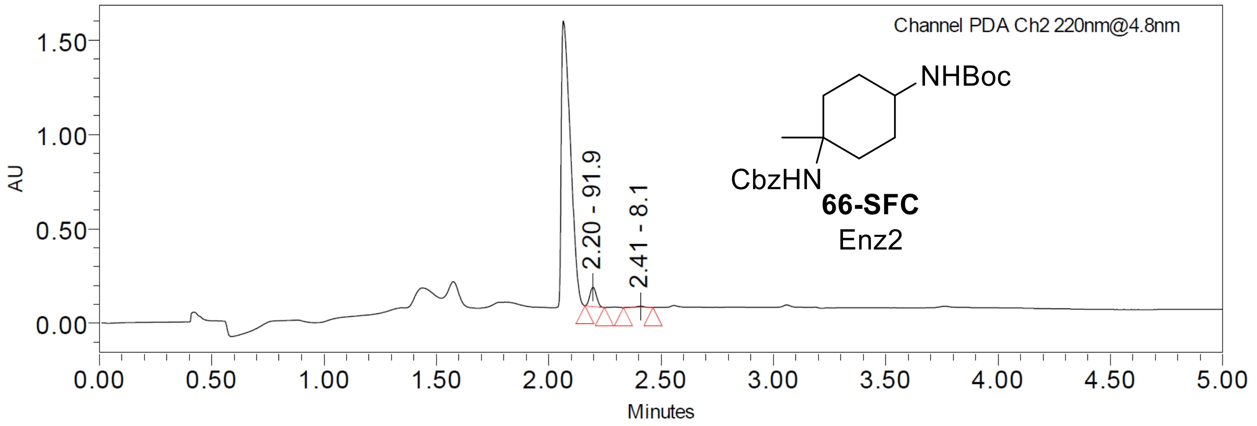
Channel PDA Ch2 220nm@4.8nm
AU
Minutes
2.20 - 91.9
2.41 - 8.1
NHBoc
CbzHN
66-SFC
Enz2

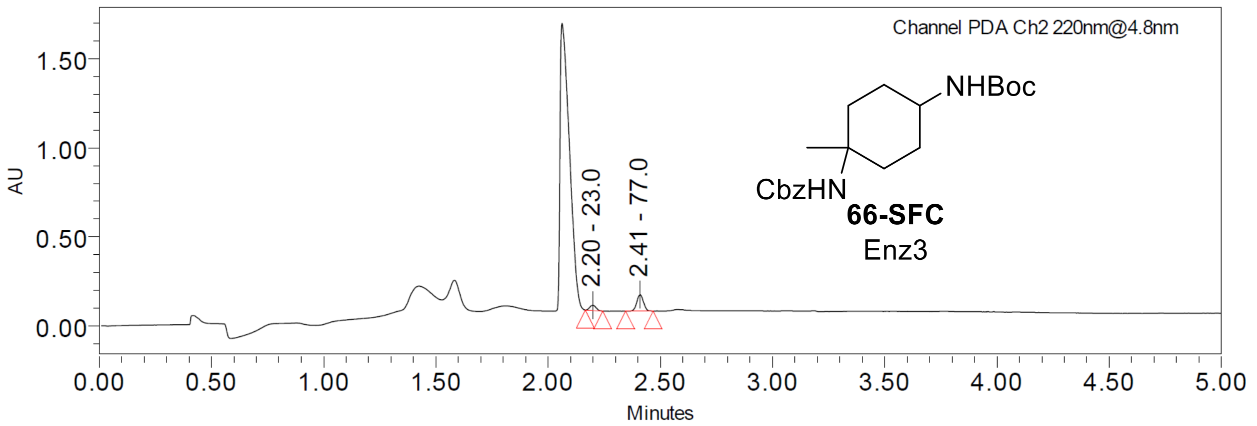
Channel PDA Ch2 220nm@4.8nm
AU
Minutes
2.20 - 23.0
2.41 - 77.0
NHBoc
CbzHN
66-SFC
Enz3

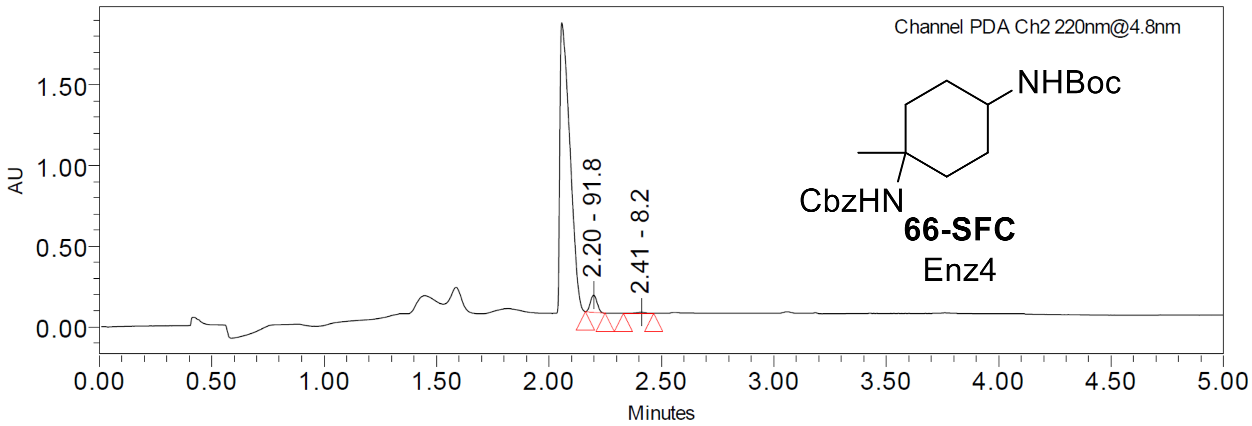
Channel PDA Ch2 220nm@4.8nm
AU
Minutes
2.20 - 91.8
2.41 - 8.2
NHBoc
CbzHN
66-SFC
Enz4

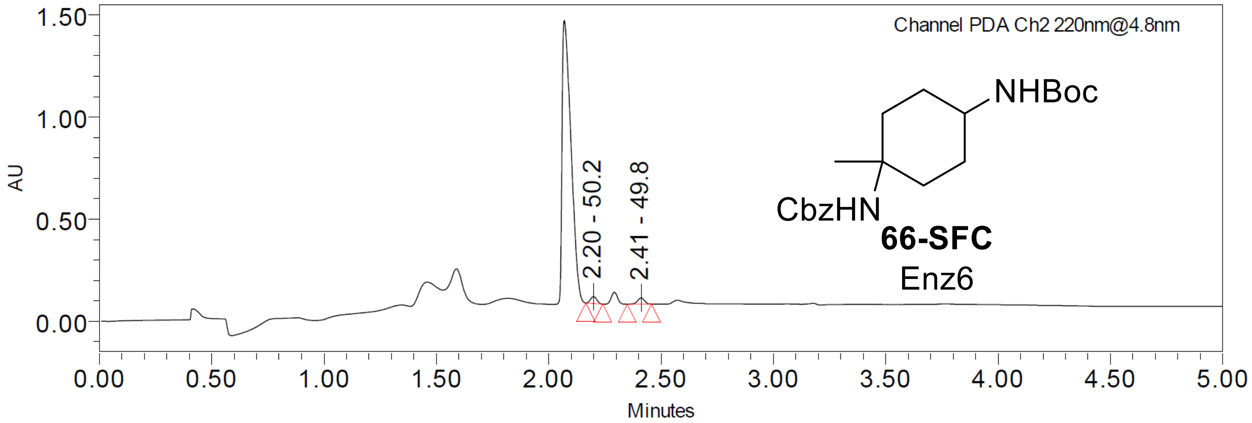
Channel PDA Ch2 220nm@4.8nm
AU
Minutes
2.20 - 50.2
2.41 - 49.8
NHBoc
CbzHN
66-SFC
Enz6

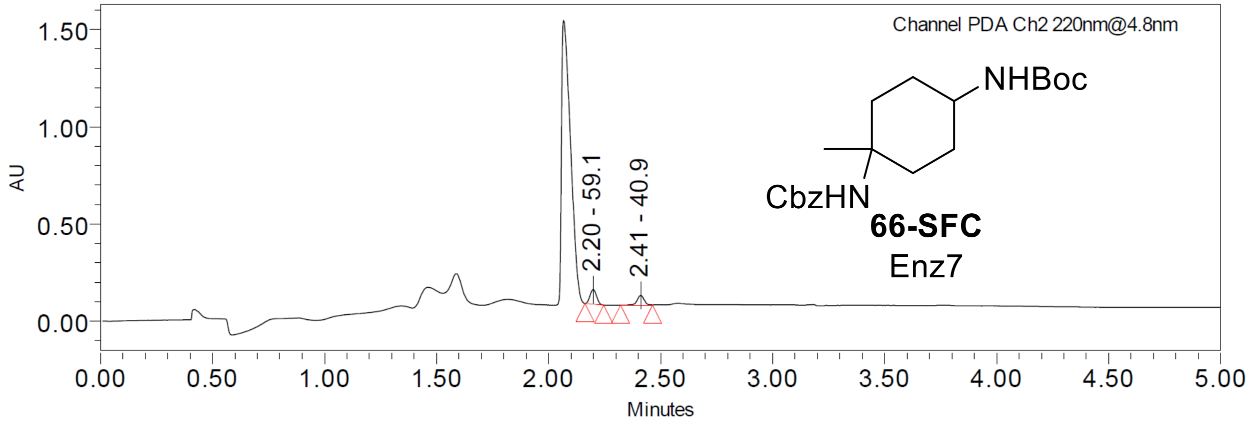
Channel PDA Ch2 220nm@4.8nm
AU
Minutes
2.20 - 59.1
2.41 - 40.9
NHBoc
CbzHN
66-SFC
Enz7

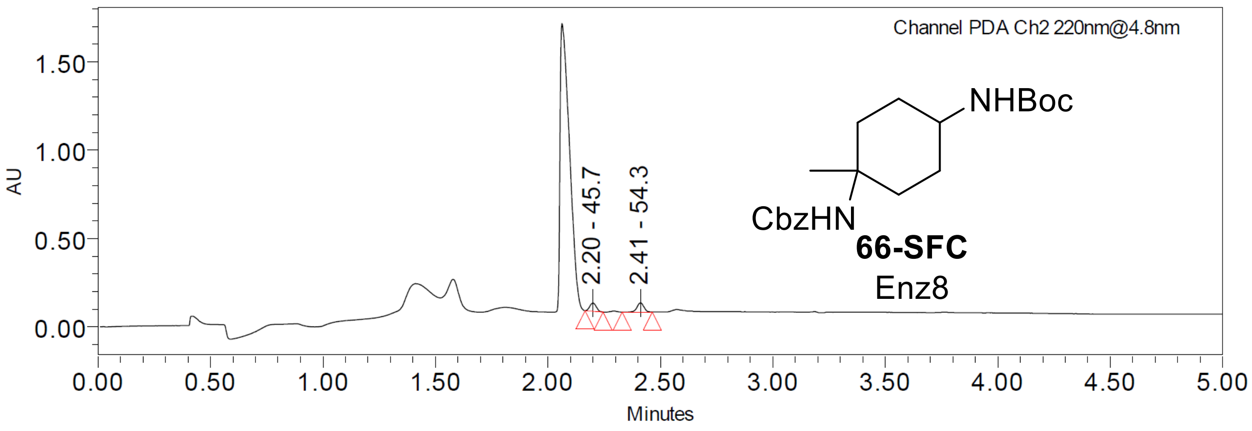

Channel PDA Ch2 220nm@4.8nm
AU
Minutes
2.20 - 45.7
2.41 - 54.3
NHBoc
CbzHN
66-SFC
Enz8

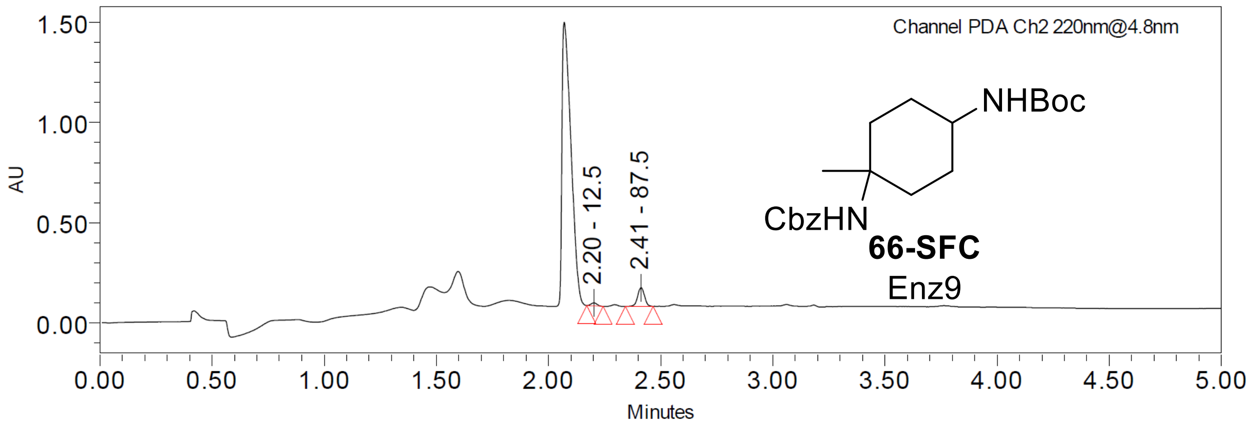

Channel PDA Ch2 220nm@4.8nm
AU
Minutes
2.20 - 12.5
2.41 - 87.5
NHBoc
CbzHN
66-SFC
Enz9

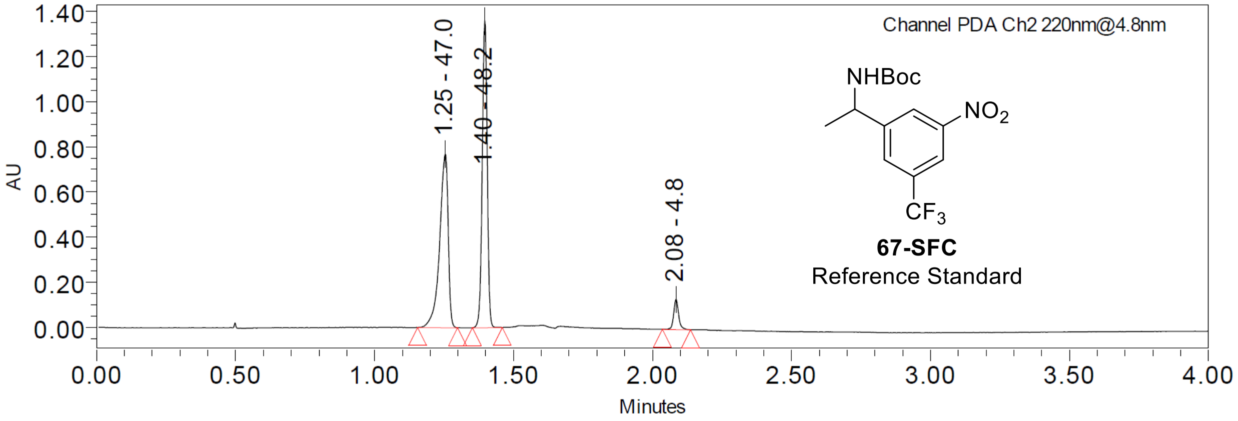

Channel PDA Ch2 220nm@4.8nm
AU
Minutes
1.25 - 47.0
1.40 - 48.2
2.08 - 4.8
NHBoc
NO_2
CF_3
67-SFC
Reference Standard

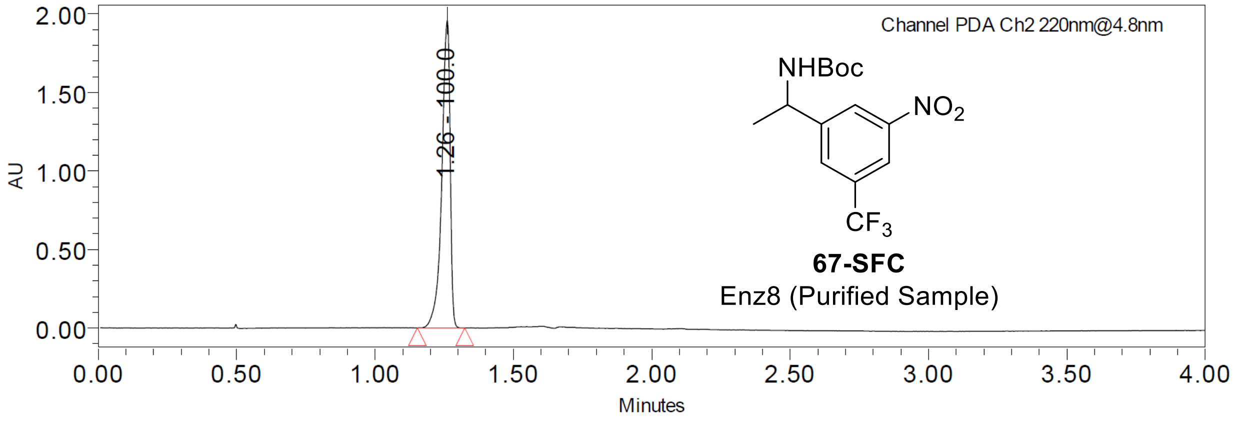
Channel PDA Ch2 220nm@4.8nm
AU
2.00
1.50
1.00
0.50
0.00
1.26 - 100.0
NHBoc
NO_2
CF_3
67-SFC
Enz8 (Purified Sample)
0.00
0.50
1.00
1.50
2.00
2.50
3.00
3.50
4.00
Minutes

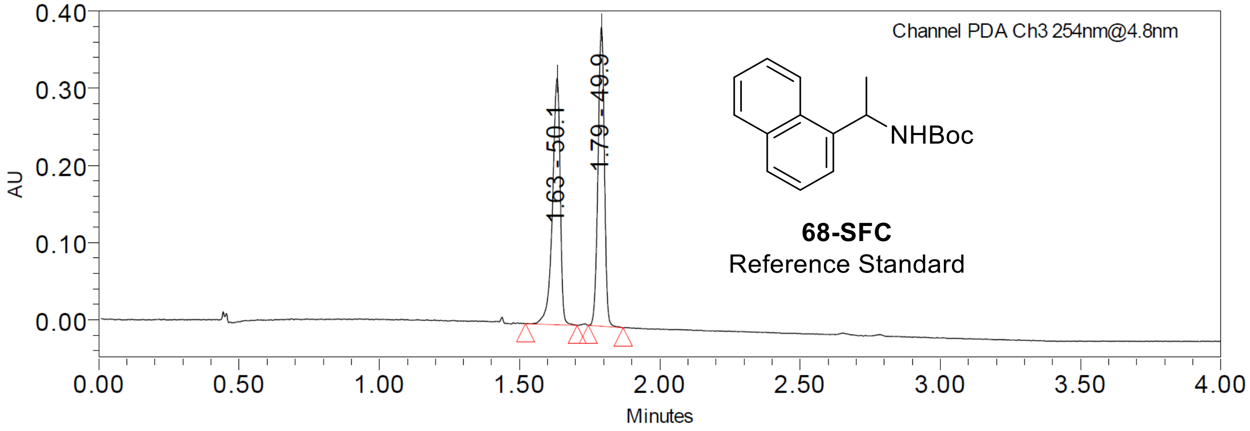
Channel PDA Ch3 254nm@4.8nm
AU
0.40
0.30
0.20
0.10
0.00
1.63 - 50.1
1.79 - 49.9
NHBoc
68-SFC
Reference Standard
0.00
0.50
1.00
1.50
2.00
2.50
3.00
3.50
4.00
Minutes

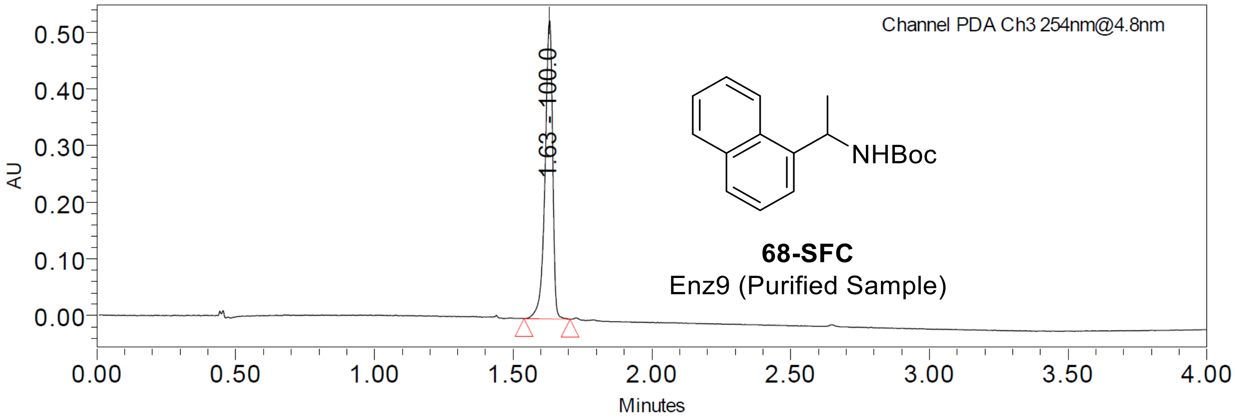
Channel PDA Ch3 254nm@4.8nm
AU
0.50
0.40
0.30
0.20
0.10
0.00
1.63 - 100.0
NHBoc
68-SFC
Enz9 (Purified Sample)
0.00
0.50
1.00
1.50
2.00
2.50
3.00
3.50
4.00
Minutes

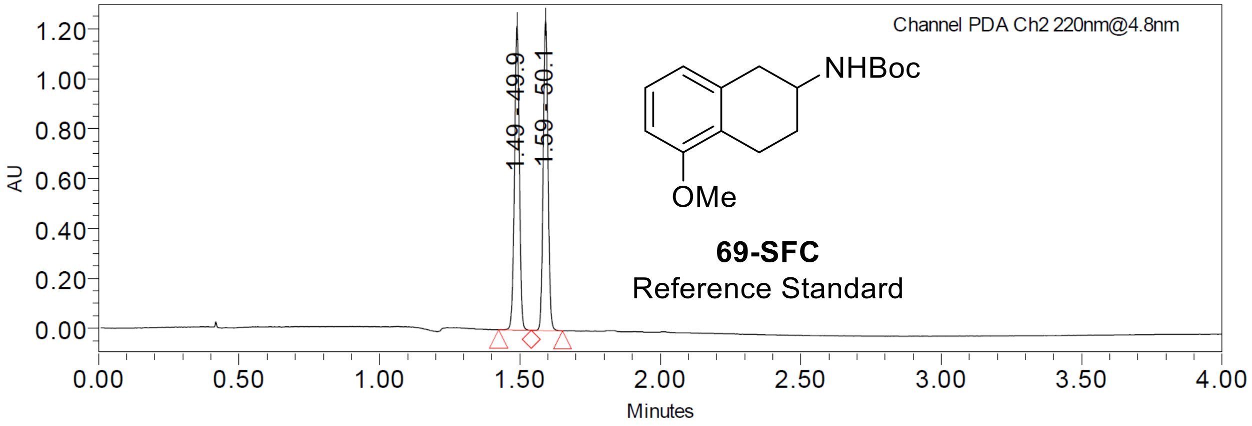

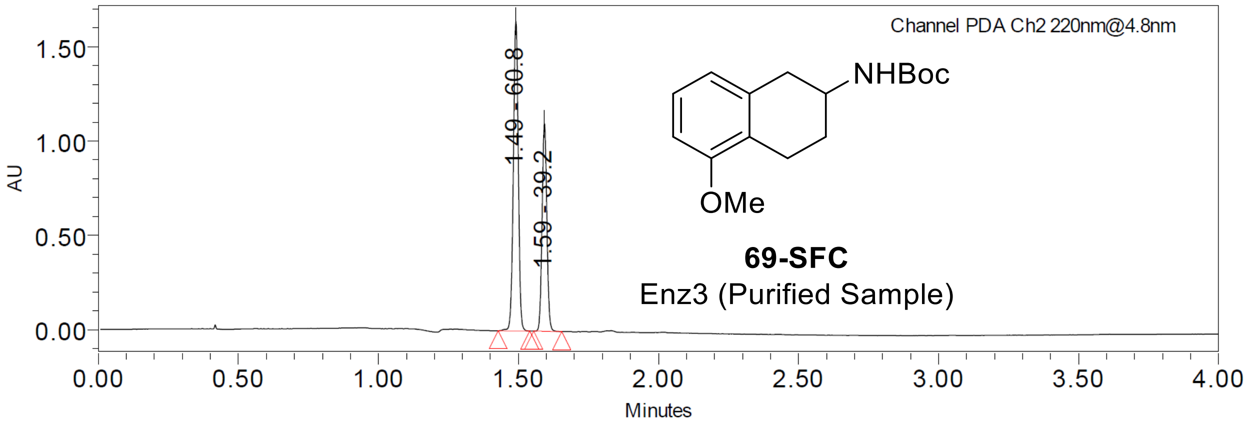

Micro-Scale Enzyme Screen:

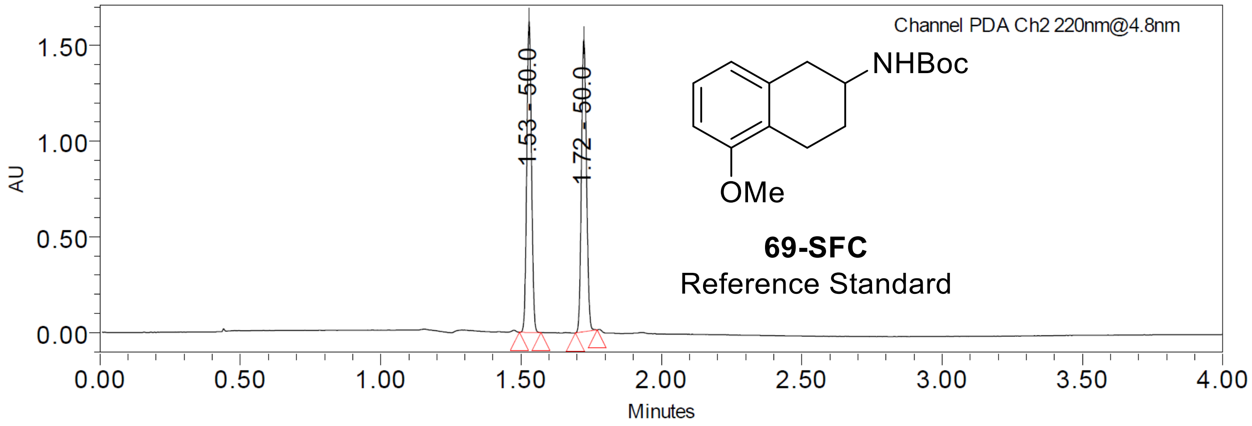

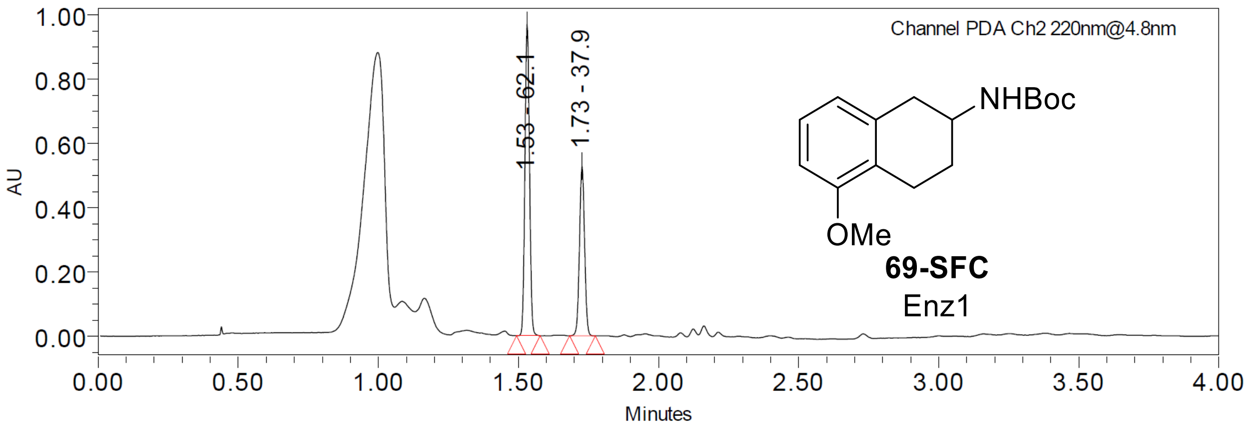
Channel PDA Ch2 220nm@4.8nm
1.53 - 62.1
1.73 - 37.9
NHBoc
OMe
69-SFC
Enz1
AU
Minutes

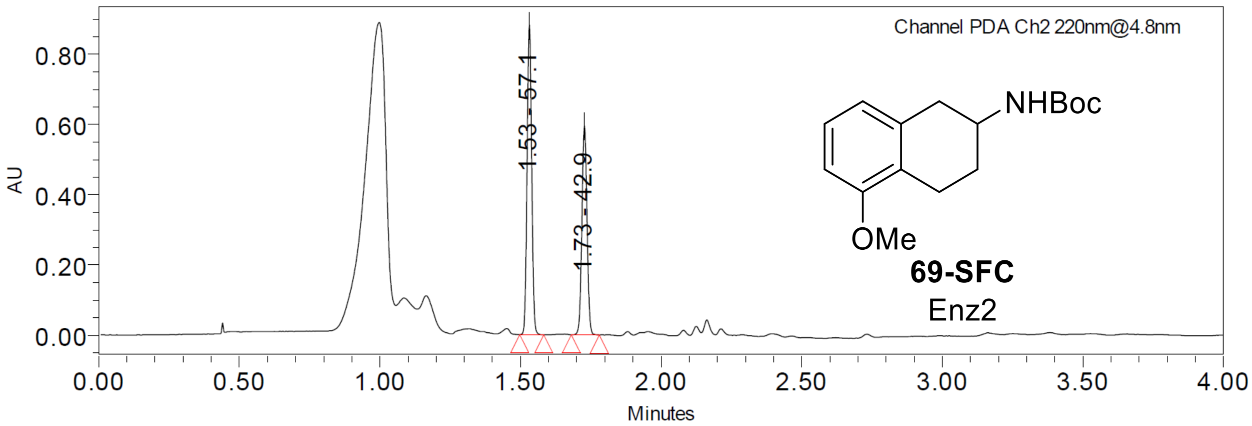
Channel PDA Ch2 220nm@4.8nm
1.53 - 57.1
1.73 - 42.9
NHBoc
OMe
69-SFC
Enz2
AU
Minutes

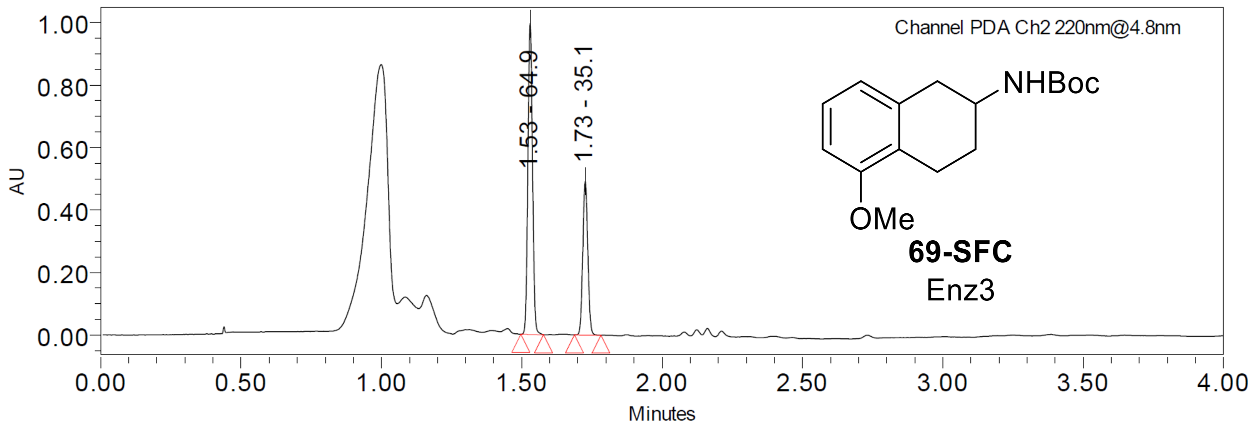
Channel PDA Ch2 220nm@4.8nm
1.53 - 64.9
1.73 - 35.1
NHBoc
OMe
69-SFC
Enz3
AU
Minutes

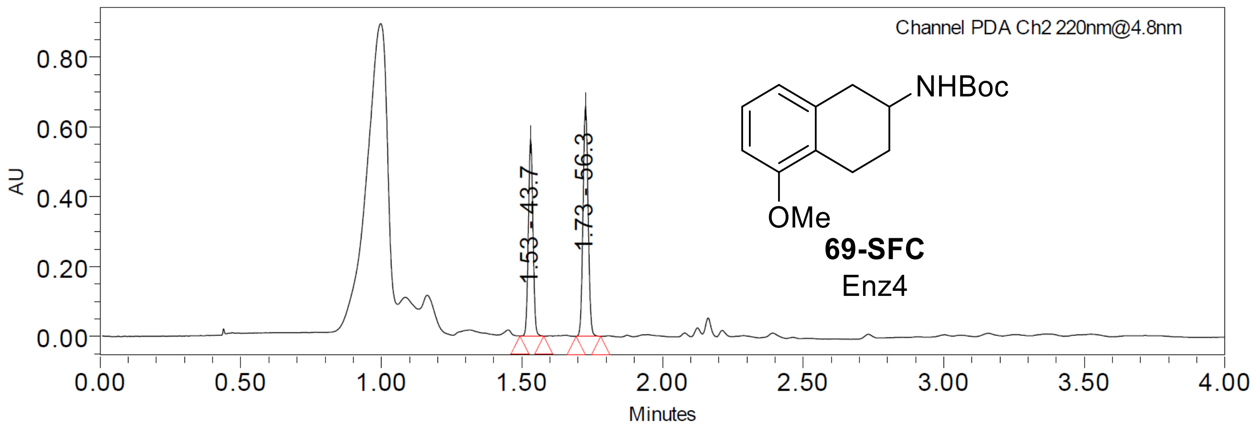
Channel PDA Ch2 220nm@4.8nm
NHBoc
OMe
69-SFC
Enz4
1.53 - 43.7
1.73 - 56.3
AU
Minutes

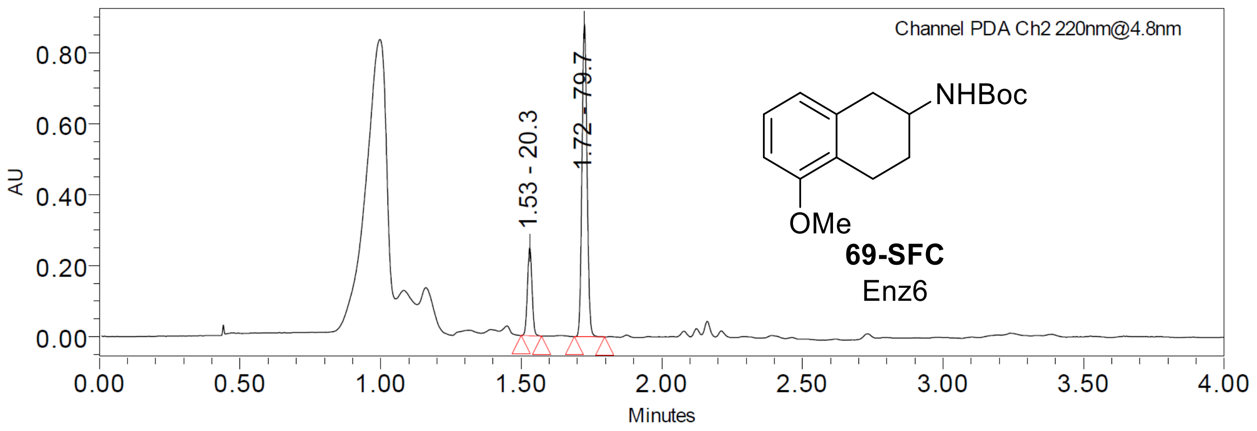
Channel PDA Ch2 220nm@4.8nm
NHBoc
OMe
69-SFC
Enz6
1.53 - 20.3
1.72 - 79.7
AU
Minutes

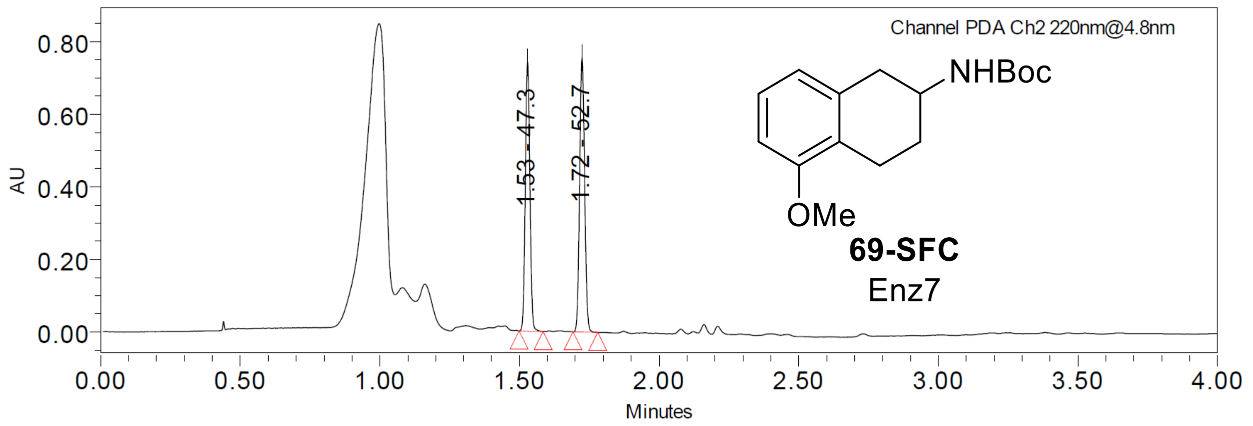
Channel PDA Ch2 220nm@4.8nm
NHBoc
OMe
69-SFC
Enz7
1.53 - 47.3
1.72 - 52.7
AU
Minutes

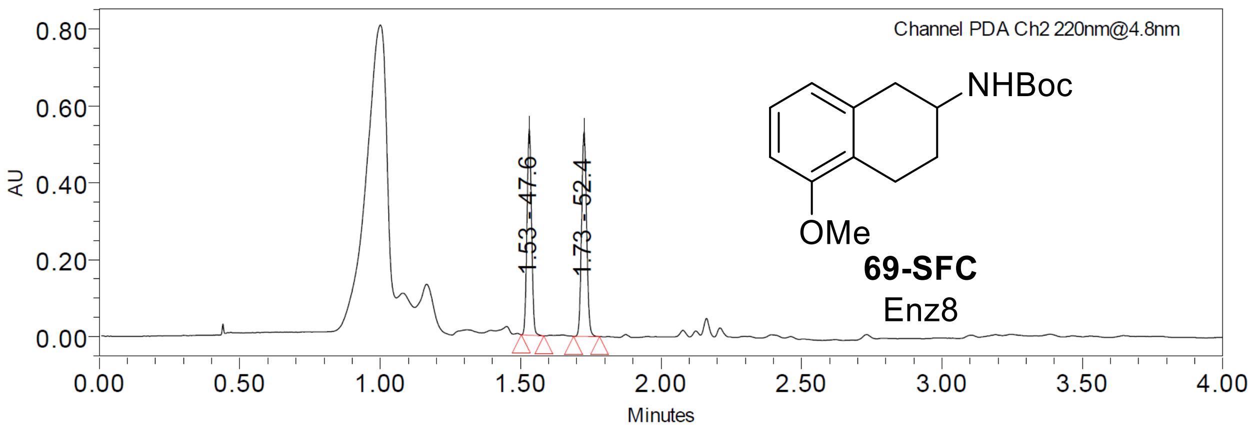

Channel PDA Ch2 220nm@4.8nm
NHBoc
OMe
69-SFC
Enz8
1.53 - 47.6
1.73 - 52.4
AU
Minutes

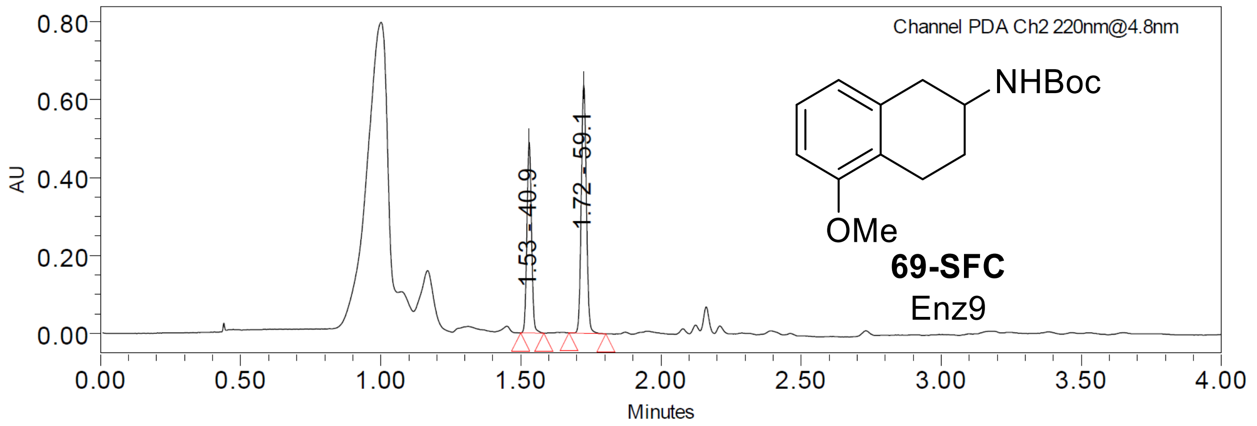

Channel PDA Ch2 220nm@4.8nm
NHBoc
OMe
69-SFC
Enz9
1.53 - 40.9
1.72 - 59.1
AU
Minutes

4. Experimental Procedures for Racemic Standard Syntheses

General Procedure for Primary Amine Synthesis: Amines were synthesized following a modified procedure.[8] To a 0.5-2.0mL Biotage microwave vial was added ketone (1 equiv., 0.17 mmol), ammonium acetate (15 equiv.), ethanol (1 mL), and $NaBH_3CN$ (1.2 equiv). The vials were sealed and heated in a microwave reactor using the following settings: 130 °C, 5 min, high absorbance. The vials were then opened and an aliquot was taken for crude LCMS analysis. The reactions were then concentrated, diluted with 1mL 2N NaOH, and extracted with CH_2Cl_2 using an ISOLUTE phase separator. The combined organics were dried with $MgSO_4$, filtered, and concentrated. The crude residue was directly used in the subsequent derivatization step.

General Procedure for Hydroxypyridyl Imine Synthesis: Amine (1.0 equiv.) was mixed with 3-hydroxypicolinaldehyde (1.0 equiv.) in acetonitrile (0.2M). The reactions were stirred until full conversion of the starting materials as measured by UPLC/LCMS. An aliquot was taken and directly used for SFC analysis. For characterization, a significant amount of the material was diverted for NMR analysis and was not included in the final sample weight. The remainder of the sample was dried down via blowing N_2 gas to obtain the hydroxypyridyl imine. Samples were not purified unless noted otherwise.

General Procedure for Boc-Amine Synthesis: Amine (0.17mmol, 1.0 equiv.) was dissolved in CH_2Cl_2 (0.1M), followed by addition of Boc_2O (2.5 equiv.) and triethylamine (5.0 equiv.). The reaction was sealed and stirred at 45 °C overnight. The reaction was concentrated by blowing N_2 gas and diluted to ~1-2mL with methanol, filtered through a syringe filter, and purified via reverse-phase preparative HPLC using an ammonium acetate (10mM)/acetonitrile gradient. The HPLC fractions were concentrated using a Savant centrifuge concentrator to obtain the pure Boc-amine.

General Procedure for Cbz-Amine Synthesis: Amine (0.2mmol, 1.0 equiv.) was dissolved in THF (0.1M), followed by addition of DIEA (3.0 equiv.). Benzyl chloroformate (1.05 equiv., 50% in toluene) was added slowly and the reaction was cooled to 0 °C and stirred until complete. The reaction was concentrated by blowing N_2 gas and diluted to ~2mL with DMSO/water (9:1), filtered through a syringe filter, and purified via reverse-phase preparative HPLC using an ammonium acetate (10mM)/acetonitrile gradient. The HPLC fractions were concentrated using a Savant centrifuge concentrator to obtain the pure Cbz-amine.

5. Experimental Procedures for Transaminase HTE and Scale-Up Reactions

150mM KPi buffer: 2.07g KH_2PO_4 was dissolved in 95mL NanoPure water and was stirred until fully dissolved. 5mL isopropylamine was added to the aqueous solution and the buffer was pH adjusted to pH=7.6 using concentrated HCl.

General Procedure for Transaminase HTE: Stock solutions of the ketone substrates in DMSO were prepared (500mM). A stock solution of PLP hydrate cofactor in KPi buffer was prepared (0.5 mg/mL; note: mixture changes color to yellow upon addition of PLP). The 8 enzymes were weighed into separate vials, to which the PLP-KPi buffer stock solution was added (10mg/mL enzyme for each). The vials were gently inverted to ensure complete mixing of the enzyme into the solution. A 96 deep-well plate was used as the HTE plate. The enzyme stock solutions were then dispensed into different columns in the HTE plate (300µL each), followed by the ketone stock solutions into different rows of the HTE plate (15µL/7.5µmol each). The plate was then sealed with breathable tape (to allow for evaporation of acetone co-product) and the plates were shaken at 35 °C/230rpm in an incubator with an integrated shaker platform for 24 hours. After 24 hours, the reactions were quenched with 500µL methanol. 30µL of each reaction were transferred to a 96-well UPLC filter plate, followed by 130µL methanol, and the plates were filtered via centrifugation at 2000xg prior to UPLC analysis.

The HTE plate was centrifuged at 2000xg to pellet the enzymatic material, and the supernatant was transferred to a 96-well HTE plate with 850µL glass vials *via* pipet. The reactions were concentrated via blowing N_2 gas and derivatized by hydroxypyridyl imine formation, Boc protection, or Cbz protection. A small number of sample sets were re-dissolved in MeOH or MeCN and directly analyzed. (Note: it can be challenging to resolve primary amines with SFC; peak resolution can be improved by basifying the enzymatic reactions with 2N NaOH prior to concentrating).

- **General Procedure for hydroxypyridyl imine derivatization:** A stock solution of 3-hydroxypicolinaldehyde in MeCN was prepared (7.5µmol in 40µL MeCN). The HTE plates were sealed and shaken overnight. Aliquots were taken for direct LCMS and chiral SFC analysis.
- **General Procedure for Boc derivatization:** A stock solution of Boc_2O (15 µmol per reaction) and triethylamine (37.5 µmol per reaction) in CH_2Cl_2 (0.5mL per reaction) was prepared and added to each reaction, followed by 200µL 0.01N NaOH. The HTE plate was sealed and the reactions were shaken overnight. The reactions were then concentrated by blowing N_2 gas and were taken up into 400µL methanol. Aliquots were taken for LCMS and chiral SFC analysis to determine enantioselectivity of the reactions.
- **General Procedure for Cbz derivatization:** A stock solution of CbzCl (1.1 equiv.) and DIEA (3 equiv.) in THF (0.0375M) was prepared. The solution was added to the plate (200 µL) and the reactions were shaken at room temperature for 2h. The reactions were then concentrated by blowing N_2 and diluted using 400µL MeOH prior to analysis by SFC and LCMS.

General Procedure for Transaminase Scale-Up Reactions: Ketone substrate (0.1125 mmol) was placed in an empty 20mL vial, followed by DMSO (225µL). Separately, a stock solution of PLP hydrate cofactor in KPi buffer was prepared (0.5 mg/mL; note: mixture changes color to yellow upon addition of PLP). A 10 mg/mL enzyme/PLP-buffer solution was prepared using the desired enzyme, of which 4.5mL was added to the 20mL vial. The vial was covered with breathable tape (to allow for evaporation of the acetone co-product) and was shaken at 35℃ at 230rpm in an incubator with an integrated orbital shaker. After 24 hours, the vial was removed and was quenched with 7.5mL methanol. An aliquot was taken for LCMS analysis. The reaction was filtered through a celite plug, which was washed with MeOH. The filtrate was concentrated to remove MeOH, then basified with concentrated NaOH and diluted with water. The mixture

was extracted 3x with diethyl ether. The combined organics were dried over $MgSO_4$, filtered, and concentrated. The crude residue was directly used for Boc-protection.

CH_2Cl_2 (6mL), Boc_2O (281.25μmol), and triethylamine (562.5μmol) were added to the vial containing the crude residue. The vial was sealed and stirred overnight, after which it was concentrated, diluted with DMSO/water (9:1, 1.5 mL), filtered via syringe filter, and purified via reverse-phase preparative HPLC using an ammonium acetate (10mM)/acetonitrile gradient. The fractions were concentrated using a Savant centrifuge concentrator and the purified Boc-amine was analyzed by chiral SFC to determine ee.

6. Racemic Standards Characterization

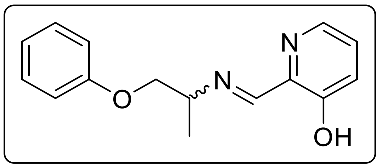

(*E*)-2-(((1-phenoxypropan-2-yl)imino)methyl)pyridin-3-ol (1-SFC): Synthesized from **1-amine** according to the general procedure for hydroxypyridyl imine synthesis. Product was not purified. 0.1 mmol scale: 68% yield (17.3 mg). Dark green oil. **^{1}H NMR (400 MHz, $CDCl_3$)** δ 13.27 – 13.22 (br s, 1H), 8.64 (d, J = 0.7 Hz, 1H), 8.20 (dd, J = 4.4, 1.5 Hz, 1H), 7.32 – 7.18 (m, 4H), 6.99 – 6.84 (m, 3H), 4.13 – 3.99 (m, 2H), 3.91 (tdd, J = 6.4, 5.5, 0.8 Hz, 1H), 1.43 (d, J = 6.5 Hz, 3H). **^{13}C NMR (101 MHz, $CDCl_3$)** δ 166.8, 158.7, 158.4, 140.9, 137.1, 129.6, 126.3, 124.8, 121.2, 114.8, 71.7, 63.5, 18.7. **HRMS** (ESI) Calcd for $C_{15}H_{16}N_2O_2$ $[M+H]^+$: 257.1285; Found: 257.1285.

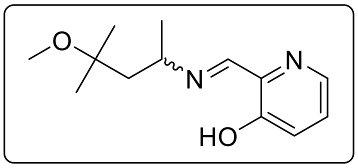

(E)-2-(((4-methoxy-4-methylpentan-2-yl)imino)methyl)pyridin-3-ol (2-SFC): Synthesized from **2-amine** according to the general procedure for hydroxypyridyl imine synthesis. Product was not purified. 0.1 mmol scale: 60% yield (14.1 mg). Yellow oil. **^{1}H NMR (400 MHz, $CDCl_3$)** δ 13.67 (s, 1H), 8.53 (s, 1H), 8.15 (dd, J = 4.4, 1.4 Hz, 1H), 7.29 – 7.24 (m, 1H), 7.19 (dd, J = 8.4, 4.4 Hz, 1H), 3.83 – 3.71 (m, 1H), 3.14 (s, 3H), 1.95 (dd, J = 14.7, 7.8 Hz, 1H), 1.78 (dd, J = 14.7, 3.6 Hz, 1H), 1.32 (d, J = 6.5 Hz, 3H), 1.17 (s, 3H), 1.14 (s, 3H). **^{13}C NMR (101 MHz, $CDCl_3$)** δ 164.7, 159.0, 140.6, 137.1, 126.1, 124.9, 74.4, 60.8, 49.3, 47.3, 26.2, 25.6, 24.5. **HRMS** (ESI) Calcd for $C_{13}H_{20}N_2O_2$ $[M+H]^+$: 237.1598; Found: 237.1596.

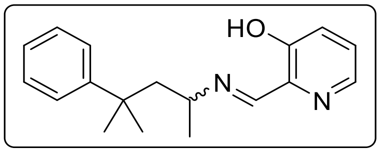

(E)-2-(((4-methyl-4-phenylpentan-2-yl)imino)methyl)pyridin-3-ol (3-SFC): Synthesized from **3-amine** according to the general procedure for hydroxypyridyl imine synthesis. Product was not purified. 0.1 mmol

scale: 67% yield (19.0 mg). Yellow oil. **^{1}H NMR (500 MHz, CDCl$_3$)** δ 13.61 – 13.25 (m, 1H), 8.10 (dd, J = 4.4, 1.5 Hz, 1H), 8.05 (s, 1H), 7.29 – 7.23 (m, 2H), 7.23 – 7.14 (m, 4H), 7.02 – 6.95 (m, 1H), 3.39 – 3.29 (m, 1H), 2.16 (dd, J = 14.4, 8.3 Hz, 1H), 1.92 (dd, J = 14.4, 3.0 Hz, 1H), 1.36 (s, 3H), 1.32 (s, 3H), 1.15 (d, J = 6.5 Hz, 3H). **^{13}C NMR (101 MHz, CDCl$_3$)** δ 164.3, 158.7, 148.1, 140.3, 137.0, 128.3, 126.0, 125.9, 125.7, 124.7, 62.3, 51.9, 37.8, 29.9, 29.8, 24.8. **HRMS** (ESI) Calcd for $C_{18}H_{22}N_2O$ $[M+H]^+$: 283.1805; Found: 283.1803.

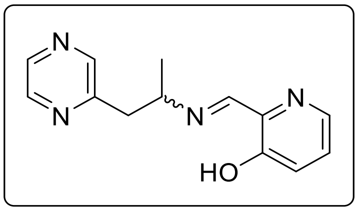

(E)-2-(((1-(pyrazin-2-yl)propan-2-yl)imino)methyl)pyridin-3-ol (4-SFC): Synthesized from **4-amine** according to the general procedure for hydroxypyridyl imine synthesis. Product was not purified. 0.1 mmol scale: 65% yield (15.8 mg). Dark brown oil. **^{1}H NMR (500 MHz, CDCl$_3$)** δ 13.14 (s, 1H), 8.50 (dd, J = 2.5, 1.5 Hz, 1H), 8.39 (dd, J = 4.6, 2.0 Hz, 2H), 8.36 (d, J = 0.6 Hz, 1H), 8.12 (dd, J = 4.4, 1.4 Hz, 1H), 7.28 – 7.22 (m, 1H), 7.18 (dd, J = 8.4, 4.4 Hz, 1H), 4.07 – 3.99 (m, 1H), 3.14 (d, J = 0.8 Hz, 1H), 3.12 (d, J = 2.4 Hz, 1H), 1.41 (d, J = 6.4 Hz, 3H). **^{13}C NMR (101 MHz, CDCl$_3$)** δ 165.8, 158.3, 154.4, 145.5, 144.5, 142.9, 140.8, 136.8, 126.3, 124.8, 64.3, 43.5, 22.4. **HRMS** (ESI) Calcd for $C_{13}H_{14}N_4O$ $[M+H]^+$: 243.1240; Found: 243.1239.

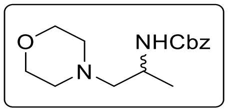

benzyl (1-morpholinopropan-2-yl)carbamate (5-SFC): Synthesized from **5-amine** according to the general procedure for Cbz amine synthesis. 0.2 mmol scale: 66% yield (36.9 mg). White solid. **^{1}H NMR (400 MHz, CDCl$_3$)** δ 7.39 – 7.28 (m, 5H), 5.10 (s, 2H), 5.05 (br s, 1H), 3.79 (s, 1H), 3.67 (dt, J = 5.6, 2.6 Hz, 4H), 2.58 – 2.44 (m, 2H), 2.43 – 2.22 (m, 4H), 1.19 (d, J = 6.4 Hz, 3H). The ^{1}H NMR spectral data are in agreement with previously published data.[9]

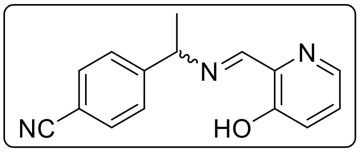

(E)-4-(1-(((3-hydroxypyridin-2-yl)methylene)amino)ethyl)benzonitrile (6-SFC): Synthesized from **6-amine** according to the general procedure for hydroxypyridyl imine synthesis. Product was purified *via* reverse-phase preparative HPLC using a focused 10mM ammonium acetate/acetonitrile gradient. 0.1 mmol scale: 36% yield (9.0 mg). Yellow solid. **^{1}H NMR (600 MHz, CDCl$_3$)** δ 12.99 (s, 1H), 8.62 (t, J = 0.6 Hz, 1H), 8.21 (dd, J = 4.4, 1.4 Hz, 1H), 7.69 – 7.65 (m, 2H), 7.51 – 7.46 (m, 2H), 7.31 (ddd, J = 8.5, 1.4, 0.5 Hz, 1H), 7.27 – 7.23 (m, 1H), 4.69 (q, J = 6.7 Hz, 1H), 1.66 (d, J = 6.7 Hz, 3H). **^{13}C NMR (101 MHz,**

$CDCl_3$) δ 166.4, 158.1, 148.6, 141.2, 136.9, 132.8, 127.4, 126.6, 124.9, 118.8, 111.6, 68.2, 24.7 **HRMS** (ESI) Calcd for $C_{15}H_{13}N_3O$ $[M+H]^+$: 252.1131; Found: 252.1130.

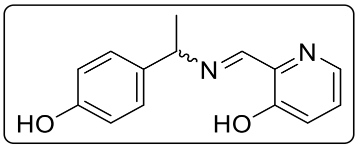

(E)-2-(((1-(4-hydroxyphenyl)ethyl)imino)methyl)pyridin-3-ol (7-SFC): Synthesized from **7-amine** according to the general procedure for hydroxypyridyl imine synthesis. Product was not purified. 0.1 mmol scale: 64% yield (15.4 mg). Dark red oil. **^{1}H NMR (500 MHz, $CDCl_3$)** δ 8.54 – 8.51 (m, 1H), 8.14 (dd, J = 4.4, 1.4 Hz, 1H), 7.32 (dd, J = 8.5, 1.4 Hz, 1H), 7.25 (dd, J = 8.5, 4.4 Hz, 1H), 7.18 – 7.12 (m, 2H), 6.82 – 6.76 (m, 2H), 4.65 (q, J = 6.7 Hz, 1H), 1.64 (d, J = 6.8 Hz, 3H). **^{13}C NMR (101 MHz, $CDCl_3$)** δ 164.3, 160.2, 156.1, 140.2, 136.3, 133.7, 128.1, 126.9, 126.1, 115.9, 66.4, 23.9. **HRMS** (ESI) Calcd for $C_{14}H_{14}N_2O_2$ $[M+H]^+$: 243.1128; Found: 243.1127.

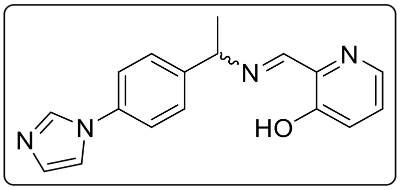

(E)-2-(((1-(4-(1H-imidazol-1-yl)phenyl)ethyl)imino)methyl)pyridin-3-ol (8-SFC): Synthesized from **8-amine** according to the general procedure for hydroxypyridyl imine synthesis. Product was purified via normal phase chromatography using a heptanes/ethyl acetate gradient. 0.1 mmol scale: 47% yield (13.6 mg). Yellow oil. **^{1}H NMR (600 MHz, $CDCl_3$)** δ 13.23 (s, 1H), 8.62 (t, J = 0.7 Hz, 1H), 8.20 (dd, J = 4.4, 1.4 Hz, 1H), 7.85 (t, J = 1.1 Hz, 1H), 7.50 – 7.47 (m, 2H), 7.41 – 7.38 (m, 2H), 7.31 (ddd, J = 8.4, 1.4, 0.5 Hz, 1H), 7.27 (q, J = 0.9 Hz, 1H), 7.24 (dd, J = 8.4, 4.4 Hz, 1H), 7.21 (t, J = 1.1 Hz, 1H), 4.72 (q, J = 6.7 Hz, 1H), 1.70 (d, J = 6.7 Hz, 3H). **^{13}C NMR (101 MHz, $CDCl_3$)** δ 165.8, 158.2, 142.8, 141.1, 137.0, 136.8, 135.7, 130.7, 128.1, 126.465 124.8, 122.0, 118.4, 67.7, 24.6. **HRMS** (ESI) Calcd for $C_{17}H_{16}N_4O$ $[M+H]^+$: 293.1397; Found: 293.1394.

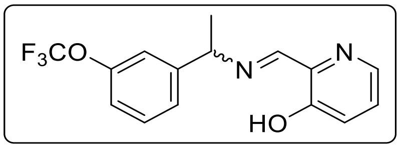

(E)-2-(((1-(3-(trifluoromethoxy)phenyl)ethyl)imino)methyl)pyridin-3-ol (9-SFC): Synthesized from **9-amine** according to the general procedure for hydroxypyridyl imine synthesis. Product was not purified. 0.1 mmol scale: 67% yield (20.9 mg). Dark brown oil. **^{1}H NMR (400 MHz, $CDCl_3$)** δ 13.14 (s, 1H), 8.61 (s, 1H), 8.20 (dd, J = 4.4, 1.4 Hz, 1H), 7.39 (t, J = 7.9 Hz, 1H), 7.31 (dd, J = 8.3, 1.5 Hz, 2H), 7.27 – 7.23 (m, 1H), 7.22 (dd, J = 2.6, 1.4 Hz, 1H), 7.14 (ddt, J = 8.0, 2.4, 1.2 Hz, 1H), 4.67 (q, J = 6.7 Hz, 1H), 1.66 (d, J = 6.7 Hz, 3H). **^{13}C NMR (101 MHz, $CDCl_3$)** δ 166.0, 158.2, 149.7 (d, J = 1.9 Hz), 145.7, 141.1, 137.0, 130.3, 126.5, 125.0, 124.9, 120.6 (q, J = 257.6 Hz) 119.9 (d, J = 1.1 Hz), 119.3 (d, J = 1.1 Hz), 67.9, 24.6.

^{19}F NMR (376 MHz, $CDCl_3$) δ -57.7 (s). **HRMS** (ESI) Calcd for $C_{15}H_{13}F_3N_2O_2$ $[M+H]^+$: 311.1002; Found: 311.0999.

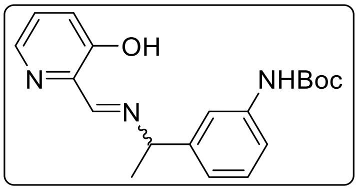

tert-butyl (E)-(3-(1-(((3-hydroxypyridin-2-yl)methylene)amino)ethyl)phenyl)carbamate (10-SFC): Synthesized from **10-amine** according to the general procedure for hydroxypyridyl imine synthesis. Product was not purified. 0.1 mmol scale: 72% yield (24.7 mg). Yellow oil. **^{1}H NMR (600 MHz, $CDCl_3$)** δ 13.44 (s, 1H), 8.56 (t, J = 0.7 Hz, 1H), 8.17 (dd, J = 4.4, 1.4 Hz, 1H), 7.37 (d, J = 2.1 Hz, 1H), 7.30 – 7.25 (m, 3H), 7.21 (dd, J = 8.4, 4.4 Hz, 1H), 7.06 – 7.02 (m, 1H), 6.59 (s, 1H), 4.66 – 4.60 (m, 1H), 1.65 (d, J = 6.7 Hz, 3H), 1.51 (s, 9H). **^{13}C NMR (101 MHz, $CDCl_3$)** δ 165.4, 158.4, 152.8, 144.2, 140.8, 138.9, 137.1, 129.6, 126.3, 124.8, 121.2, 117.8, 116.8, 80.7, 68.2, 28.5, 24.5. **HRMS** (ESI) Calcd for $C_{19}H_{23}N_3O_3$ $[M+H]^+$: 342.1812; Found: 342.1810.

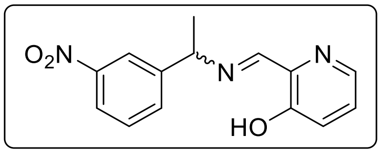

(E)-2-(((1-(3-nitrophenyl)ethyl)imino)methyl)pyridin-3-ol (11-SFC): Synthesized from **11-amine** according to the general procedure for hydroxypyridyl imine synthesis. Product was not purified. 0.1 mmol scale: 72% yield (19.6 mg). Yellow oil. **^{1}H NMR (500 MHz, $CDCl_3$)** δ 12.93 (s, 1H), 8.64 (s, 1H), 8.27 – 8.12 (m, 3H), 7.72 (dt, J = 7.9, 1.5 Hz, 1H), 7.55 (t, J = 8.0 Hz, 1H), 7.31 (dd, J = 8.5, 1.4 Hz, 1H), 7.27 – 7.20 (m, 1H), 4.76 (q, J = 6.7 Hz, 1H), 1.71 (d, J = 6.7 Hz, 3H). **^{13}C NMR (101 MHz, $CDCl_3$)** δ 166.5, 158.1, 148.7, 145.4, 141.2, 136.9, 132.8, 130.0, 126.6, 124.9, 122.7, 121.7, 67.8, 24.5. **HRMS** (ESI) Calcd for $C_{14}H_{13}N_3O_3$ $[M+H]^+$: 272.1030; Found: 272.1028.

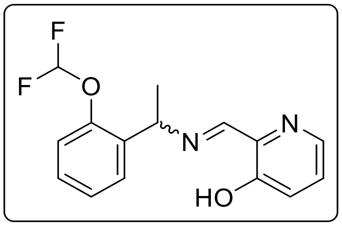

(E)-2-(((1-(2-(difluoromethoxy)phenyl)ethyl)imino)methyl)pyridin-3-ol (12-SFC): Synthesized from **12-amine** according to the general procedure for hydroxypyridyl imine synthesis. Product was not purified. 0.1 mmol scale: 31% yield (9.1 mg). Yellow oil. **^{1}H NMR (400 MHz, $CDCl_3$)** δ 13.41 (s, 1H), 8.60 (s, 1H), 8.18 (dd, J = 4.4, 1.4 Hz, 1H), 7.52 (dd, J = 7.5, 2.0 Hz, 1H), 7.34 – 7.18 (m, 4H), 7.12 (dt, J = 7.8, 1.1 Hz, 1H), 6.55 (t, J = 73.7 Hz, 1H), 5.10 (q, J = 6.7 Hz, 1H), 1.65 (d, J = 6.7 Hz, 3H). **^{13}C NMR (101 MHz, $CDCl_3$)** δ 166.1, 158.4, 148.6, 140.9, 137.1, 134.5, 128.8, 128.1, 126.4, 126.1, 124.8, 118.9, 116.4 (t, J =

259.7 Hz), 61.3, 23.4. **^{19}F NMR (376 MHz, $CDCl_3$)** δ -79.3 – -80.6 (m). **HRMS** (ESI) Calcd for $C_{15}H_{14}F_2N_2O_2$ $[M+H]^+$: 293.1096; Found: 293.1095.

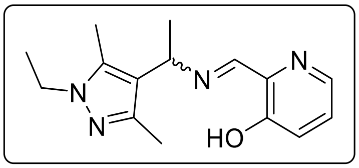

(E)-2-(((1-(1-ethyl-3,5-dimethyl-1H-pyrazol-4-yl)ethyl)imino)methyl)pyridin-3-ol (15-SFC): Synthesized from **15-amine** according to the general procedure for hydroxypyridyl imine synthesis. Product was not purified. 0.1 mmol scale: 59% yield (16.1 mg). Yellow oil. **^{1}H NMR (400 MHz, $CDCl_3$)** δ 13.59 (s, 1H), 8.46 (d, J = 1.3 Hz, 1H), 8.15 (dd, J = 4.4, 1.5 Hz, 1H), 7.30 – 7.25 (m, 1H), 7.20 (dd, J = 8.4, 4.4 Hz, 1H), 4.64 (qd, J = 7.0, 1.1 Hz, 1H), 4.01 (q, J = 7.3 Hz, 2H), 2.26 (s, 3H), 2.25 (s, 3H), 1.64 (d, J = 7.0 Hz, 3H), 1.36 (t, J = 7.2 Hz, 3H). **^{13}C NMR (101 MHz, $CDCl_3$)** δ 164.9, 158.6, 145.0, 140.7, 137.2, 135.2, 126.2, 124.8, 116.9, 59.5, 43.8, 23.1, 15.6, 13.1, 10.1. **HRMS** (ESI) Calcd for $C_{15}H_{20}N_4O$ $[M+H]^+$: 273.1710; Found: 273.1709.

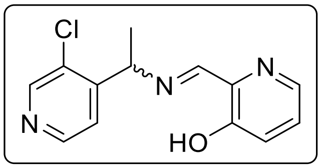

(E)-2-(((1-(3-chloropyridin-4-yl)ethyl)imino)methyl)pyridin-3-ol (16-SFC): Synthesized from **16-amine** according to the general procedure for hydroxypyridyl imine synthesis. Product was not purified. 0.1 mmol scale: 70% yield (18.3 mg). Pale yellow solid. **^{1}H NMR (400 MHz, $CDCl_3$)** δ 12.94 (s, 1H), 8.67 (s, 1H), 8.59 (s, 1H), 8.51 (d, J = 5.1 Hz, 1H), 8.23 (dd, J = 4.4, 1.5 Hz, 1H), 7.44 (d, J = 5.1 Hz, 1H), 7.33 (dd, J = 8.5, 1.5 Hz, 1H), 7.26 (dd, J = 8.5, 4.3 Hz, 1H), 5.09 (q, J = 6.7 Hz, 1H), 1.66 (d, J = 6.7 Hz, 3H). **^{13}C NMR (101 MHz, $CDCl_3$)** δ 167.3, 158.1, 150.0, 149.1, 148.6, 141.3, 136.8, 130.5, 126.7, 124.9, 122.1, 63.9, 22.8. **HRMS** (ESI) Calcd for $C_{13}H_{12}ClN_3O$ $[M+H]^+$: 262.0742; Found: 262.0740.

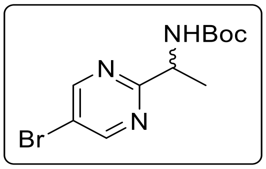

tert-butyl (1-(5-bromopyrimidin-2-yl)ethyl)carbamate (17-SFC): Synthesized from **17-amine** according to the general procedure for Boc-amine synthesis. 0.1 mmol scale: 56% yield (16.9 mg). White solid. **^{1}H NMR (500 MHz, $CDCl_3$)** δ 8.73 (s, 2H), 5.57 (s, 1H), 4.95 (t, J = 7.3 Hz, 1H), 1.48 (d, J = 7.0 Hz, 3H), 1.44 (s, 9H). **^{13}C NMR (101 MHz, $CDCl_3$)** δ 169.3, 157.9, 155.3, 118.5, 79.7, 52.2, 28.5, 22.1. **HRMS** (ESI) Calcd for $C_{11}H_{16}BrN_3O_2$ $[M-tBu+2H]^+$: 245.9873; Found: 245.9871.

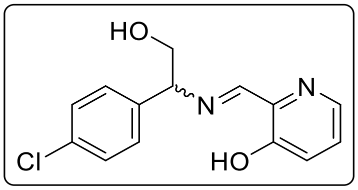

(E)-2-(((1-(4-chlorophenyl)-2-hydroxyethyl)imino)methyl)pyridin-3-ol (18-SFC): Synthesized from **18-amine** according to the general procedure for hydroxypyridyl imine synthesis. Product was not purified. 0.1 mmol scale: 27% yield (7.5 mg). Yellow oil. **^{1}H NMR (400 MHz, DMSO-*d$_6$*, 90 °C)** 1H NMR (400 MHz, DMSO) δ 13.39 (s, 1H), 8.65 (s, 1H), 8.20 (dd, J = 3.5, 2.4 Hz, 1H), 7.46 (s, 4H), 7.40 – 7.36 (m, 2H), 5.15 (s, 1H), 4.62 (dd, J = 8.3, 4.4 Hz, 1H), 3.70 (qd, J = 11.0, 6.5 Hz, 2H). **^{13}C NMR (101 MHz, DMSO-*d$_6$*, 90 °C)** δ 166.6, 157.2, 140.1, 138.5, 136.3, 131.7, 128.5, 128.0, 126.0, 123.8, 72.7, 65.2. **HRMS** (ESI) Calcd for $C_{14}H_{13}ClN_2O_2$ $[M+H]^+$: 277.0738; Found: 277.0737.

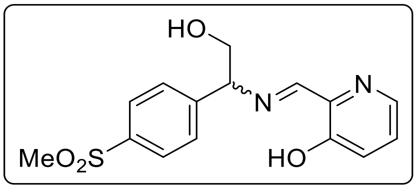

(E)-2-(((2-hydroxy-1-(4-(methylsulfonyl)phenyl)ethyl)imino)methyl)pyridin-3-ol (19-SFC): Synthesized from **19-amine** according to the general procedure for hydroxypyridyl imine synthesis. Product was not purified. 0.1 mmol scale: 54% yield (17.3 mg). Dark yellow oil. **^{1}H NMR (400 MHz, DMSO-*d$_6$*, 90 °C)** δ 13.10 (s, 1H), 8.69 (s, 1H), 8.20 (dd, J = 3.6, 2.3 Hz, 1H), 7.99 – 7.91 (m, 2H), 7.74 – 7.67 (m, 2H), 7.43 – 7.31 (m, 2H), 4.78 (dd, J = 7.6, 4.7 Hz, 1H), 3.87 (dd, J = 11.0, 4.8 Hz, 1H), 3.81 (dd, J = 11.0, 7.6 Hz, 1H), 3.17 (s, 3H). **^{13}C NMR (101 MHz, DMSO-*d$_6$*, 90 °C)** δ 167.1, 157.1, 145.3, 140.3, 139.9, 136.3, 127.7, 126.6, 126.1, 123.8, 73.1, 65.2, 43.3. **HRMS** (ESI) Calcd for $C_{15}H_{16}N_2O_4S$ $[M+H]^+$: 321.0904; Found: 321.0901.

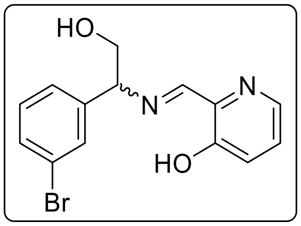

(E)-2-(((1-(3-bromophenyl)-2-hydroxyethyl)imino)methyl)pyridin-3-ol (20-SFC): Synthesized from **20-amine** according to the general procedure for hydroxypyridyl imine synthesis. Product was not purified. 0.1 mmol scale: 54% yield (17.3 mg). Yellow oil. **^{1}H NMR (400 MHz, DMSO-*d$_6$*, 90 °C)** δ 13.14 (s, 1H), 8.66 (d, J = 0.7 Hz, 1H), 8.19 (dd, J = 3.6, 2.2 Hz, 1H), 7.62 (t, J = 1.8 Hz, 1H), 7.49 (ddd, J = 7.8, 2.0, 1.1 Hz, 1H), 7.44 (dt, J = 7.8, 1.4 Hz, 1H), 7.38 – 7.32 (m, 3H), 4.65 (dd, J = 7.8, 4.7 Hz, 1H), 3.83 (dd, J = 11.0, 4.7 Hz, 1H), 3.76 (dd, J = 11.0, 7.8 Hz, 1H). **^{13}C NMR (101 MHz, DMSO-*d$_6$*, 90 °C)** δ 166.8, 157.2, 142.4, 140.2, 136.3, 130.1, 129.8, 129.5, 126.0, 125.8, 123.8, 121.3, 72.8, 65.2. **HRMS** (ESI) Calcd for $C_{14}H_{13}BrN_2O_2$ $[M+H]^+$: 321.0233; Found: 321.0231.

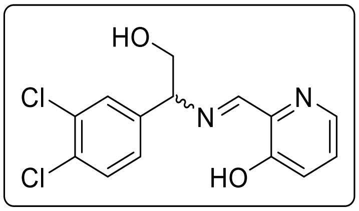

(E)-2-(((1-(3,4-dichlorophenyl)-2-hydroxyethyl)imino)methyl)pyridin-3-ol (21-SFC): Synthesized from **21-amine** according to the general procedure for hydroxypyridyl imine synthesis. Product was not purified. 0.1 mmol scale: 49% yield (15.3 mg). Yellow solid. **^{1}H NMR (400 MHz, DMSO-*d*$_6$, 90 °C)** δ 13.03 (s, 1H), 8.66 (d, J = 0.7 Hz, 1H), 8.20 (dd, J = 3.7, 2.1 Hz, 1H), 7.66 (d, J = 2.1 Hz, 1H), 7.62 (d, J = 8.2 Hz, 1H), 7.43 (dd, J = 8.3, 2.0 Hz, 1H), 7.38 – 7.30 (m, 2H), 4.90 (s, 1H), 4.68 (dd, J = 7.6, 4.8 Hz, 1H), 3.83 (dd, J = 11.0, 4.8 Hz, 1H), 3.76 (dd, J = 11.0, 7.5 Hz, 1H). **^{13}C NMR (101 MHz, DMSO-*d*$_6$, 90 °C)** δ 167.1, 157.1, 140.7, 140.2, 136.3, 130.8, 130.2, 129.7, 128.9, 127.0, 126.1, 123.8, 72.2, 65.0. **HRMS** (ESI) Calcd for $C_{14}H_{12}Cl_2N_2O_2$ [M+H]$^+$: 311.0349; Found: 311.0347.

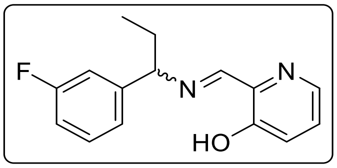

(E)-2-(((1-(3-fluorophenyl)propyl)imino)methyl)pyridin-3-ol (22-SFC): Synthesized from **22-amine** according to the general procedure for hydroxypyridyl imine synthesis. Product was not purified. 0.1 mmol scale: 70% yield (18.0 mg). Yellow oil. **^{1}H NMR (400 MHz, CDCl$_3$)** δ 13.27 (s, 1H), 8.56 (s, 1H), 8.19 (dd, J = 4.4, 1.5 Hz, 1H), 7.37 – 7.28 (m, 2H), 7.23 (dd, J = 8.4, 4.4 Hz, 1H), 7.13 (dt, J = 7.7, 1.3 Hz, 1H), 7.07 (ddd, J = 9.8, 2.6, 1.6 Hz, 1H), 6.97 (tdd, J = 8.4, 2.6, 1.0 Hz, 1H), 4.30 (t, J = 6.9 Hz, 1H), 1.99 (p, J = 7.3 Hz, 2H), 0.93 (t, J = 7.4 Hz, 3H). **^{13}C NMR (101 MHz, CDCl$_3$)** δ 166.2, 163.2 (d, J = 246.1 Hz), 158.3, 145.0 (d, J = 7.0 Hz), 141.0, 137.0, 130.4 (d, J = 8.4 Hz), 126.4, 124.8, 122.7 (d, J = 2.9 Hz), 114.5 (d, J = 21.0 Hz), 114.0 (d, J = 21.7 Hz), 75.1 (d, J = 1.8 Hz), 31.5, 10.9. **^{19}F NMR (376 MHz, CDCl$_3$)** δ -113.1e (td, J = 9.2, 6.0 Hz). **HRMS** (ESI) Calcd for $C_{15}H_{15}FN_2O$ [M+H]$^+$: 259.1241; Found: 259.1240.

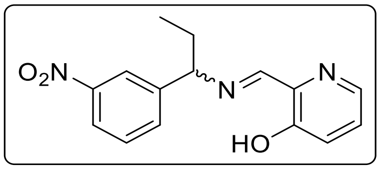

(E)-2-(((1-(3-nitrophenyl)propyl)imino)methyl)pyridin-3-ol (23-SFC): Synthesized from **23-amine** according to the general procedure for hydroxypyridyl imine synthesis. Product was not purified. 0.1 mmol scale: 48% yield (13.7 mg). Yellow oil. **^{1}H NMR (400 MHz, CDCl$_3$)** δ 12.98 (s, 1H), 8.62 (s, 1H), 8.25 – 8.19 (m, 2H), 8.15 (ddd, J = 8.2, 2.3, 1.1 Hz, 1H), 7.72 (dt, J = 7.8, 1.4 Hz, 1H), 7.55 (t, J = 7.9 Hz, 1H), 7.33 (dd, J = 8.5, 1.4 Hz, 1H), 7.26 (dd, J = 8.5, 4.3 Hz, 1H), 4.42 (t, J = 6.9 Hz, 1H), 2.11 – 1.99 (m, 2H), 0.96 (t, J = 7.4 Hz, 3H). **^{13}C NMR (101 MHz, CDCl$_3$)** δ 166.8, 158.2, 148.6, 144.6, 141.2, 136.8, 133.1,

129.9, 126.7, 124.9, 122.7, 122.1, 75.0, 31.6, 10.9. **HRMS** (ESI) Calcd for $C_{15}H_{15}N_3O_3$ $[M+H]^+$: 286.1186; Found: 286.1184.

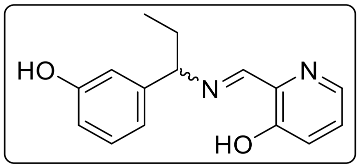

(E)-2-(((1-(3-hydroxyphenyl)propyl)imino)methyl)pyridin-3-ol (24-SFC): Synthesized from **24-amine** according to the general procedure for hydroxypyridyl imine synthesis. Product was not purified. 0.1 mmol scale: 74% yield (19.0 mg). Yellow oil. **^{1}H NMR (400 MHz, $CDCl_3$)** δ 8.51 (s, 1H), 8.17 (dd, J = 4.4, 1.5 Hz, 1H), 7.31 (dd, J = 8.4, 1.5 Hz, 1H), 7.25 (dd, J = 8.4, 4.5 Hz, 1H), 7.19 (t, J = 7.9 Hz, 1H), 6.86 (dt, J = 7.8, 1.2 Hz, 1H), 6.78 (t, J = 2.1 Hz, 1H), 6.76 – 6.71 (m, 1H), 4.21 (t, J = 6.9 Hz, 1H), 1.95 (p, J = 7.3 Hz, 2H), 0.89 (t, J = 7.3 Hz, 3H). **^{13}C NMR (101 MHz, $CDCl_3$)** δ 165.0, 159.4, 156.9, 143.7, 140.3, 136.4, 130.2, 126.8, 125.8, 118.8, 114.9, 114.1, 74.8, 31.2, 11.0. **HRMS** (ESI) Calcd for $C_{15}H_{16}N_2O_3$ $[M+H]^+$: 257.1285; Found: 257.1283.

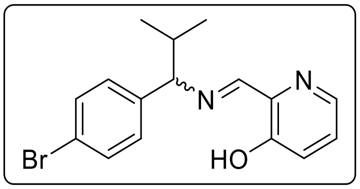

(E)-2-(((1-(4-bromophenyl)-2-methylpropyl)imino)methyl)pyridin-3-ol (25-SFC): Synthesized from **25-amine** according to the general procedure for hydroxypyridyl imine synthesis. Product was not purified. 0.1 mmol scale: 69% yield (23.1 mg). Yellow oil. **^{1}H NMR (400 MHz, $CDCl_3$)** δ 13.34 (s, 1H), 8.52 (s, 1H), 8.18 (dd, J = 4.4, 1.4 Hz, 1H), 7.51 – 7.43 (m, 2H), 7.30 (dd, J = 8.4, 1.4 Hz, 1H), 7.25 – 7.18 (m, 3H), 4.00 (d, J = 7.4 Hz, 1H), 2.20 (dq, J = 13.6, 6.8 Hz, 1H), 0.96 (d, J = 6.7 Hz, 3H), 0.86 (d, J = 6.7 Hz, 3H). **^{13}C NMR (101 MHz, $CDCl_3$)** δ 166.3, 158.3, 141.0, 140.9, 137.0, 131.8, 129.2, 126.4, 124.8, 121.4, 80.4, 35.1, 19.7, 19.2. **HRMS** (ESI) Calcd for $C_{16}H_{17}BrN_2O$ $[M+H]^+$: 333.0597; Found: 333.0596.

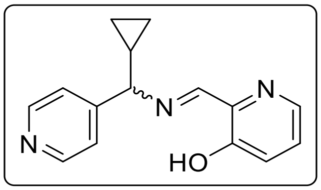

(E)-2-(((cyclopropyl(pyridin-4-yl)methyl)imino)methyl)pyridin-3-ol (28-SFC): Synthesized from **28-amine** according to the general procedure for hydroxypyridyl imine synthesis. Product was not purified. 0.1 mmol scale: 50% yield (12.7 mg). Orange oil. **^{1}H NMR (400 MHz, $CDCl_3$)** δ 13.06 (s, 1H), 8.61 (dd, J = 5.9, 1.3 Hz, 3H), 8.22 (dd, J = 4.4, 1.4 Hz, 1H), 7.36 – 7.31 (m, 3H), 7.26 (dd, J = 8.5, 4.3 Hz, 1H), 3.77 (d, J = 8.4 Hz, 1H), 1.40 (qt, J = 8.2, 5.0 Hz, 1H), 0.78 – 0.63 (m, 2H), 0.57 – 0.47 (m, 1H), 0.47 – 0.40 (m,

1H). **^{13}C NMR (101 MHz, $CDCl_3$)** δ 166.7, 158.2, 150.6, 150.3, 141.2, 137.0, 126.6, 124.9, 122.0, 76.3, 18.3, 4.2, 4.0. **HRMS** (ESI) Calcd for $C_{15}H_{15}N_3O$ $[M+H]^+$: 254.1288; Found: 254.1286.

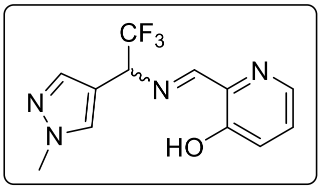

(E)-2-(((2,2,2-trifluoro-1-(1-methyl-1H-pyrazol-4-yl)ethyl)imino)methyl)pyridin-3-ol (31-SFC): Synthesized from **31-amine** according to the general procedure for hydroxypyridyl imine synthesis. Product was not purified. 0.1 mmol scale: 70% yield (20.0 mg). Yellow oil. **^{1}H NMR (500 MHz, $CDCl_3$)** δ 12.29 (s, 1H), 8.56 (s, 1H), 8.22 (dd, J = 4.3, 1.4 Hz, 1H), 7.54 (s, 1H), 7.46 (s, 1H), 7.34 (dd, J = 8.5, 1.4 Hz, 1H), 7.28 (dd, J = 8.5, 4.4 Hz, 1H), 5.03 (qd, J = 7.3, 1.1 Hz, 1H), 3.92 (s, 3H). **^{13}C NMR (101 MHz, $CDCl_3$)** δ 170.8, 158.2, 141.5, 138.9 (d, J = 1.1 Hz), 136.5, 129.8 (d, J = 1.1 Hz), 127.3, 125.2, 124.3 (q, J = 279.9 Hz), 113.1 (d, J = 1.5 Hz), 65.3 (q, J = 30.9 Hz), 39.4. **^{19}F NMR (376 MHz, $CDCl_3$)** δ -75.3 (d, J = 7.3 Hz). **HRMS** (ESI) Calcd for $C_{12}H_{11}F_3N_4O$ $[M+H]^+$: 285.0958; Found: 285.0956.

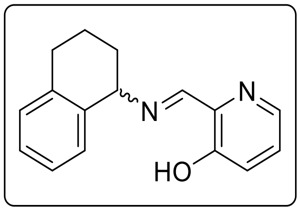

(E)-2-(((1,2,3,4-tetrahydronaphthalen-1-yl)imino)methyl)pyridin-3-ol (35-SFC): Synthesized from **35-amine** according to the general procedure for hydroxypyridyl imine synthesis. Product was not purified. 0.1 mmol scale: 63% yield (16.0 mg). Dark orange oil. **^{1}H NMR (400 MHz, $CDCl_3$)** δ 13.45 (s, 1H), 8.60 (s, 1H), 8.19 (dd, J = 4.4, 1.5 Hz, 1H), 7.30 – 7.25 (m, 1H), 7.24 – 7.18 (m, 2H), 7.18 – 7.11 (m, 2H), 7.10 – 7.04 (m, 1H), 4.67 (t, J = 5.7 Hz, 1H), 2.88 (qt, J = 16.8, 6.2 Hz, 2H), 2.19 – 1.96 (m, 3H), 1.95 – 1.83 (m, 1H). **^{13}C NMR (101 MHz, $CDCl_3$)** δ 165.8, 158.6, 140.8, 137.3, 137.2, 135.5, 129.5, 129.0, 127.7, 126.3, 126.2, 124.9, 66.5, 31.4, 29.3, 19.6. **HRMS** (ESI) Calcd for $C_{16}H_{16}N_2O$ $[M+H]^+$: 253.1335; Found: 253.1335.

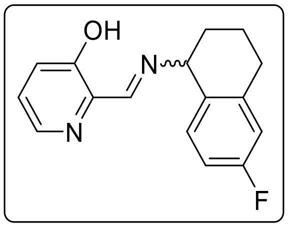

(E)-2-(((6-fluoro-1,2,3,4-tetrahydronaphthalen-1-yl)imino)methyl)pyridin-3-ol (36-SFC): Synthesized from **36-amine** according to the general procedure for hydroxypyridyl imine synthesis. Product was not purified. 0.1 mmol scale: 68% yield (18.5 mg). Yellow oil. **^{1}H NMR (400 MHz, $CDCl_3$)** δ 13.33

(s, 1H), 8.57 (s, 1H), 8.19 (dd, J = 4.4, 1.5 Hz, 1H), 7.31 – 7.25 (m, 1H), 7.22 (dd, J = 8.4, 4.4 Hz, 1H), 7.03 (dd, J = 8.6, 5.8 Hz, 1H), 6.84 (dd, J = 8.7, 7.3 Hz, 2H), 4.63 (t, J = 5.6 Hz, 1H), 2.96 – 2.76 (m, 2H), 2.17 – 1.81 (m, 4H). **^{13}C NMR (101 MHz, $CDCl_3$)** δ 165.8, 162.1 (d, J = 246.1 Hz), 158.5, 140.9, 139.7 (d, J = 7.5 Hz), 137.1, 131.3 (d, J = 3.0 Hz), 130.7 (d, J = 8.3 Hz), 126.3, 124.9, 115.6 (d, J = 20.6 Hz), 113.5 (d, J = 21.6 Hz), 65.9, 31.3, 29.5 (d, J = 1.5 Hz), 19.2. **^{19}F NMR (376 MHz, $CDCl_3$)** δ -115.7 (td, J = 9.0, 6.0 Hz). **HRMS** (ESI) Calcd for $C_{16}H_{15}FN_2O$ $[M+H]^+$: 271.1241; Found: 271.1240.

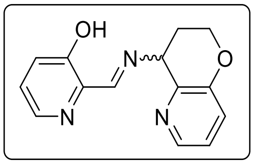

(E)-2-(((3,4-dihydro-2H-pyrano[3,2-b]pyridin-4-yl)imino)methyl)pyridin-3-ol (37-SFC): Synthesized from **37-amine** according to the general procedure for hydroxypyridyl imine synthesis. Product was purified via normal phase chromatography using a heptanes/EtOAc gradient. 0.1 mmol scale: 46% yield (11.7 mg). Yellow oil. **^{1}H NMR (500 MHz, $CDCl_3$)** δ 13.04 (s, 1H), 8.80 – 8.76 (m, 1H), 8.21 (dd, J = 4.4, 1.5 Hz, 1H), 8.19 (dd, J = 4.4, 1.5 Hz, 1H), 7.28 – 7.24 (m, 1H), 7.24 – 7.18 (m, 2H), 7.15 (dd, J = 8.3, 4.4 Hz, 1H), 4.76 (dd, J = 4.7, 3.6 Hz, 1H), 4.43 – 4.30 (m, 2H), 2.51 – 2.40 (m, 1H), 2.31 (dtd, J = 14.3, 3.8, 2.5 Hz, 1H). **^{13}C NMR (101 MHz, $CDCl_3$)** δ 167.4, 158.1, 151.7, 142.5, 141.1, 140.9, 137.2, 126.3, 124.9, 124.7, 124.6, 63.0, 62.7, 29.8. **HRMS** (ESI) Calcd for $C_{14}H_{13}N_3O_2$ $[M+H]^+$: 256.1081; Found: 256.1079.

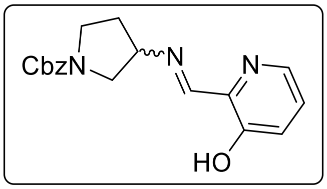

benzyl (E)-3-(((3-hydroxypyridin-2-yl)methylene)amino)pyrrolidine-1-carboxylate (38-SFC): Synthesized from **38-amine** according to the general procedure for hydroxypyridyl imine synthesis. Product was not purified. 0.1 mmol scale: 43% yield (14.1 mg). Yellow oil. **^{1}H NMR (400 MHz, DMSO-d_6, 90 °C)** δ 8.67 (s, 1H), 8.19 (dd, J = 4.0, 1.8 Hz, 1H), 7.41 – 7.26 (m, 7H), 5.12 (s, 2H), 4.28 (p, J = 5.3 Hz, 1H), 3.73 (dd, J = 11.1, 6.0 Hz, 1H), 3.59 – 3.52 (m, 2H), 3.43 (ddd, J = 11.1, 4.2, 0.8 Hz, 1H), 2.28 (dtd, J = 13.2, 7.5, 5.8 Hz, 1H), 2.02 (ddt, J = 12.7, 7.3, 5.5 Hz, 1H). **^{13}C NMR (101 MHz, DMSO-d_6, 90 °C)** δ 166.2, 157.0, 153.6, 140.2, 136.7, 136.2, 127.8, 127.1, 126.8, 126.0, 123.8, 65.5, 65.0, 51.9, 44.0, 32.1. **HRMS** (ESI) Calcd for $C_{18}H_{19}N_3O_3$ $[M+H]^+$: 326.1499; Found: 326.1497.

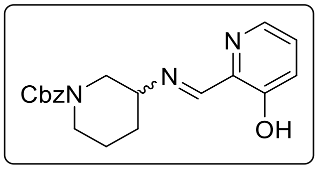

benzyl (E)-3-(((3-hydroxypyridin-2-yl)methylene)amino)piperidine-1-carboxylate (39-SFC): Synthesized from **39-amine** according to the general procedure for hydroxypyridyl imine synthesis. Product

was not purified. 0.1 mmol scale: 55% yield (18.6 mg). Yellow oil. **^{1}H NMR (400 MHz, DMSO-*d$_6$*, 90 °C)** δ 8.67 (d, J = 0.7 Hz, 1H), 8.18 (dd, J = 4.0, 1.8 Hz, 1H), 7.41 – 7.23 (m, 7H), 5.09 (s, 2H), 3.81 (ddt, J = 13.0, 3.8, 1.1 Hz, 1H), 3.63 (tt, J = 7.5, 4.0 Hz, 2H), 3.45 – 3.29 (m, 2H), 2.04 – 1.93 (m, 1H), 1.84 – 1.68 (m, 2H), 1.61 – 1.48 (m, 1H). **^{13}C NMR (101 MHz, DMSO-*d$_6$*, 90 °C)** δ 166.0, 157.3, 154.3, 140.0, 136.5, 136.2, 127.8, 127.1, 126.8, 125.9, 123.8, 65.8, 61.7, 48.7, 43.3, 30.7, 21.7. **HRMS** (ESI) Calcd for $C_{19}H_{21}N_3O_3$ [M+H]$^+$: 340.1656; Found: 340.1653.

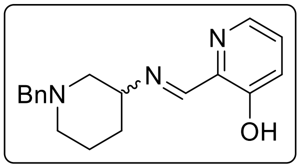

(E)-2-(((1-benzylpiperidin-3-yl)imino)methyl)pyridin-3-ol (40-SFC): Synthesized from **40-amine** according to the general procedure for hydroxypyridyl imine synthesis. Product was not purified. 0.1 mmol scale: 69% yield (20.3 mg). Orange oil. **^{1}H NMR (600 MHz, CDCl$_3$)** δ 13.51 (s, 1H), 8.55 (t, J = 0.7 Hz, 1H), 8.16 (dd, J = 4.4, 1.4 Hz, 1H), 7.34 – 7.29 (m, 4H), 7.28 – 7.23 (m, 2H), 7.20 (dd, J = 8.4, 4.4 Hz, 1H), 3.61 – 3.50 (m, 3H), 2.91 (ddt, J = 10.8, 3.9, 1.5 Hz, 1H), 2.81 (dd, J = 10.4, 5.0 Hz, 1H), 2.26 – 2.19 (m, 1H), 2.10 (td, J = 11.1, 3.0 Hz, 1H), 1.94 – 1.86 (m, 1H), 1.84 – 1.76 (m, 1H), 1.70 (dddt, J = 13.4, 11.9, 11.0, 3.9 Hz, 1H), 1.54 (dddd, J = 12.7, 11.9, 10.2, 4.3 Hz, 1H). **^{13}C NMR (101 MHz, CDCl$_3$)** δ 165.6, 158.8, 140.7, 138.2, 137.0, 129.2, 128.4, 127.2, 126.2, 125.0, 65.2, 63.3, 59.6, 53.3, 32.3, 23.9. **HRMS** (ESI) Calcd for $C_{18}H_{21}N_3O$ [M+H]$^+$: 296.1757; Found: 296.1756.

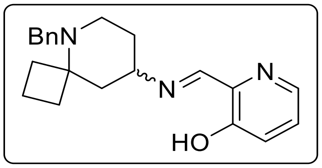

(E)-2-(((5-benzyl-5-azaspiro[3.5]nonan-8-yl)imino)methyl)pyridin-3-ol (42-SFC): Synthesized from **42-amine** according to the general procedure for hydroxypyridyl imine synthesis. Product was not purified. 0.1 mmol scale: 68% yield (22.7 mg). Dark brown oil. **^{1}H NMR (400 MHz, CDCl$_3$)** δ 13.52 (s, 1H), 8.54 (s, 1H), 8.14 (dd, J = 4.4, 1.4 Hz, 1H), 7.35 (d, J = 7.4 Hz, 2H), 7.28 (td, J = 8.7, 7.9, 2.2 Hz, 2H), 7.25 – 7.15 (m, 3H), 3.91 (d, J = 14.0 Hz, 1H), 3.51 (tt, J = 11.1, 4.4 Hz, 1H), 3.40 (d, J = 13.9 Hz, 1H), 2.66 (ddd, J = 13.2, 4.2, 2.9 Hz, 1H), 2.45 (td, J = 12.8, 2.8 Hz, 1H), 2.30 (td, J = 7.0, 3.4 Hz, 1H), 2.14 (t, J = 9.7 Hz, 1H), 2.04 (ddd, J = 12.9, 4.3, 2.0 Hz, 1H), 1.96 – 1.86 (m, 1H), 1.85 – 1.70 (m, 5H), 1.52 (ddd, J = 12.9, 4.8, 2.3 Hz, 1H). **^{13}C NMR (101 MHz, CDCl$_3$)** δ 164.8, 158.7, 140.7 (two signals overlapping), 137.2, 128.6, 128.4, 126.9, 126.2, 124.9, 63.5, 61.2, 51.4, 43.7, 40.8, 32.3, 29.6, 28.3, 13.6. **HRMS** (ESI) Calcd for $C_{21}H_{25}N_3O$ [M+H]$^+$: 336.2070; Found: 336.2067.

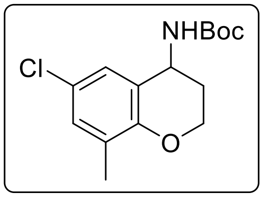

***tert*-butyl (6-chloro-8-methylchroman-4-yl)carbamate (47-SFC):** Synthesized from **47-ketone** according to the general procedures for amine and Boc amine synthesis. 0.17 mmol scale: 46% yield (23.3 mg). White solid. **^{1}H NMR (600 MHz, CDCl$_3$)** δ 7.08 (d, J = 2.6 Hz, 1H), 6.99 (d, J = 2.6 Hz, 1H), 4.78 (d, J = 7.4 Hz, 2H), 4.27 (ddd, J = 11.3, 6.6, 3.2 Hz, 1H), 4.16 (ddd, J = 11.5, 8.8, 2.8 Hz, 1H), 2.13 (s, 4H), 2.06 – 1.97 (m, 1H), 1.48 (s, 9H).. **^{13}C NMR (151 MHz, CDCl$_3$)** δ 155.2, 151.9, 130.1, 128.4, 126.3, 124.7, 123.3, 80.1, 63.6, 44.9, 29.4, 28.5, 16.1. **HRMS** (ESI) Calcd for $C_{15}H_{20}ClNO_3$ [M-Boc-NH$_2$+H]$^+$: 181.0415; Found: 181.0415.

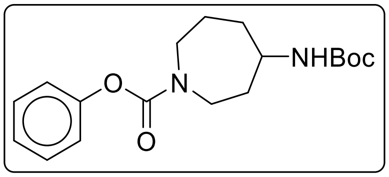

phenyl 4-((*tert*-butoxycarbonyl)amino)azepane-1-carboxylate (48-SFC): Synthesized from **48-ketone** according to the general procedures for amine and Boc amine synthesis. 0.1 mmol scale: 66% yield (37.8 mg). Colorless oil. **^{1}H NMR (400 MHz, DMSO-*d$_6$*, 90 °C)** δ 7.42 – 7.33 (m, 2H), 7.20 (td, J = 7.3, 1.2 Hz, 1H), 7.15 – 7.08 (m, 2H), 6.43 (d, J = 7.4 Hz, 1H), 3.76 – 3.42 (m, 4H), 3.36 (s, 1H), 1.96 (s, 1H), 1.92 – 1.76 (m, 2H), 1.59 (dd, J = 30.2, 20.0 Hz, 3H), 1.41 (s, 9H).. **^{13}C NMR (101 MHz, DMSO-*d$_6$*, 90 °C)** δ 154.3, 153.2, 151.2, 128.6, 124.3, 121.1, 77.2, 50.2, 46.0, 42.6, 33.8, 32.6, 27.9, 23.5. **HRMS** (ESI) Calcd for $C_{18}H_{26}N_2O_4$ [M-Boc+2H]$^+$: 235.1441; Found: 235.1442.

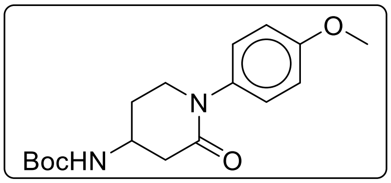

***tert*-butyl (1-(4-methoxyphenyl)-2-oxopiperidin-4-yl)carbamate (49-SFC):** Synthesized from **49-ketone** according to the general procedures for amine and Boc amine synthesis. 0.17 mmol scale: 42% yield (28.7 mg). White solid. **^{1}H NMR (400 MHz, CDCl$_3$)** δ 7.17 – 7.07 (m, 2H), 6.96 – 6.83 (m, 2H), 4.70 (s, 1H), 4.04 (s, 1H), 3.79 (s, 3H), 3.70 – 3.57 (m, 2H), 2.89 (ddd, J = 17.4, 5.6, 1.7 Hz, 1H), 2.39 (dd, J = 17.3, 8.9 Hz, 1H), 2.24 (d, J = 13.2 Hz, 1H), 1.90 (dtd, J = 13.2, 9.1, 6.0 Hz, 1H), 1.46 (s, 9H). **^{13}C NMR (101 MHz, CDCl$_3$)** δ 168.3, 158.5, 155.2, 135.5, 127.5, 114.7, 80.1, 55.6, 48.9, 45.4, 39.3, 29.8, 28.5. **HRMS** (ESI) Calcd for $C_{17}H_{24}N_2O_4$ [M+H]$^+$: 321.1809 Found: 321.1810.

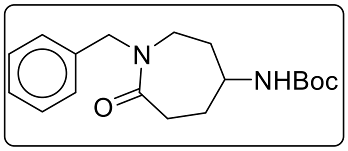

***tert*-butyl (1-benzyl-7-oxoazepan-4-yl)carbamate (50-SFC):** Synthesized in two steps from **50-ketone**. A modified procedure was used to synthesize **50-amine**. Substrate was placed in a 4mL vial with MeOH (0.193M), ammonia in MeOH (10 equiv., 7N), and sodium cyanoborohydride (1.2 equiv.). The reaction was stirred at room temperature for 48h, after which point the reaction was concentrated, diluted with 2N NaOH, and extracted with DCM. The combined organics were dried over $MgSO_4$, filtered, and concentrated to afford the crude amine. The crude residue was then subjected to the general procedure for Boc amine synthesis. 0.1 mmol scale: 29% yield (15.5 mg). White solid. **^{1}H NMR (400 MHz, $CDCl_3$)** δ 7.36 – 7.20 (m, 5H), 4.53 (dd, J = 35.5, 6.7 Hz, 3H), 3.63 (s, 1H), 3.36 (dd, J = 15.6, 10.4 Hz, 1H), 3.23 (ddd, J = 15.7, 6.9, 1.8 Hz, 1H), 2.64 (ddd, J = 14.4, 8.2, 1.8 Hz, 1H), 2.55 (ddd, J = 14.3, 11.7, 1.8 Hz, 1H), 2.20 – 2.03 (m, 1H), 2.02 – 1.88 (m, 1H), 1.42 (m, 10H), 1.20 (p, J = 11.6 Hz, 1H). **^{13}C NMR (101 MHz, $CDCl_3$)** δ 174.8, 155.0, 137.7, 128.8, 128.3, 127.6, 79.8, 51.9, 51.2, 45.6, 35.0, 33.6, 30.1, 28.5. **HRMS** (ESI) Calcd for $C_{18}H_{26}N_2O_3$ $[M\text{-}tBu+2H]^+$: 263.1390; Found: 263.1391.

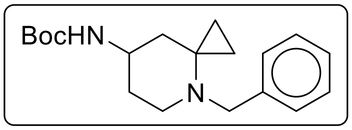

***tert*-butyl (4-benzyl-4-azaspiro[2.5]octan-7-yl)carbamate (51-SFC):** Synthesized from **51-ketone** according to the general procedures for amine and Boc amine synthesis. 0.17 mmol scale: 37% yield (25.2 mg). Off-white solid. **^{1}H NMR (600 MHz, $CDCl_3$)** δ 7.27 (ddd, J = 13.5, 8.1, 6.6 Hz, 4H), 7.23 – 7.19 (m, 1H), 4.55 (s, 1H), 3.82 (d, J = 13.6 Hz, 1H), 3.76 (d, J = 13.6 Hz, 2H), 2.77 (pt, J = 8.2, 3.7 Hz, 2H), 1.83 (t, J = 11.8 Hz, 1H), 1.64 (d, J = 14.7 Hz, 1H), 1.55 (dtd, J = 13.3, 10.9, 4.9 Hz, 1H), 1.45 (s, 9H), 1.25 (s, 1H), 0.74 – 0.61 (m, 2H), 0.55 – 0.37 (m, 2H).**^{13}C NMR (151 MHz, $CDCl_3$)** δ 155.3, 139.9, 128.7, 128.3, 127.0, 79.4, 54.0, 48.5, 46.1, 41.9, 34.9, 28.6, 25.7, 14.9, 13.9. **HRMS** (ESI) Calcd for $C_{19}H_{28}N_2O_2$ $[M+H]^+$: 317.2224; Found: 317.2225.

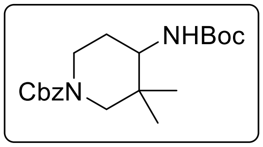

benzyl 4-((*tert*-butoxycarbonyl)amino)-3,3-dimethylpiperidine-1-carboxylate (52-SFC): Synthesized from **52-ketone** according to the general procedures for amine and Boc amine synthesis. 0.17 mmol scale: 46% yield (29.0 mg). White solid. **^{1}H NMR (400 MHz, $CDCl_3$)** δ 7.40 – 7.28 (m, 5H), 5.11 (d, J = 2.5 Hz, 2H), 4.37 (d, J = 9.8 Hz, 1H), 4.13 (d, J = 29.3 Hz, 1H), 3.94 – 3.65 (m, 1H), 3.47 (t, J = 10.4 Hz, 1H), 3.02 – 2.77 (m, 1H), 2.66 (s, 1H), 1.71 (d, J = 16.3 Hz, 2H), 1.44 (s, 10H), 0.93 (s, 3H), 0.81 (s, 3H). **^{13}C NMR (101 MHz, $CDCl_3$)** δ 155.7, 155.6, 137.0, 128.6, 128.1, 128.0, 79.6, 67.3, 55.3, 55.1, 43.6, 35.6, 28.5 (two overlapping signals), 25.0, 18.1. **HRMS** (ESI) Calcd for $C_{20}H_{30}N_2O_4$ $[M\text{-}Boc+2H]^+$: 263.1754; Found: 263.1755.

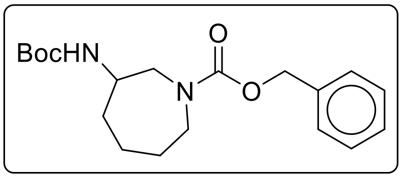

benzyl 3-((*tert*-butoxycarbonyl)amino)azepane-1-carboxylate (56-SFC): Synthesized from **56-ketone** according to the general procedures for amine and Boc amine synthesis. 0.17 mmol scale: 59% yield (34.7 mg). Pale yellow oil. **^{1}H NMR (400 MHz, DMSO-d_6, 90 °C)** δ 7.41 – 7.22 (m, 5H), 6.27 (d, J = 7.7 Hz, 1H), 5.10 (s, 2H), 3.78 – 3.58 (m, 2H), 3.53 (dt, J = 12.8, 5.8 Hz, 1H), 3.35 – 3.19 (m, 1H), 3.12 (dd, J = 13.8, 8.4 Hz, 1H), 1.80 – 1.64 (m, 3H), 1.39 (m, 12H).. **^{13}C NMR (101 MHz, DMSO-d_6, 90 °C)** δ 154.9, 154.3, 136.7, 127.8, 127.1, 126.8, 77.3, 65.8, 50.7, 49.9, 47.0, 32.9, 27.8, 27.0, 21.7. **HRMS** (ESI) Calcd for $C_{19}H_{28}N_2O_4$ [M-Boc+2H]$^+$: 249.1598; Found: 249.1598.

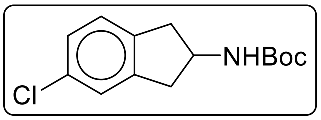

tert-butyl (5-chloro-2,3-dihydro-1H-inden-2-yl)carbamate (57-SFC): Synthesized from **57-ketone** according to the general procedures for amine and Boc amine synthesis. 0.17 mmol scale: 21% yield (9.6 mg). Off-white powder. **^{1}H NMR (400 MHz, CDCl$_3$)** δ 7.19 (t, J = 1.3 Hz, 1H), 7.13 (d, J = 1.7 Hz, 2H), 4.73 (s, 1H), 4.46 (s, 1H), 3.24 (ddd, J = 16.4, 9.7, 7.0 Hz, 2H), 2.75 (ddd, J = 16.6, 12.4, 5.0 Hz, 2H), 1.44 (s, 9H). **^{13}C NMR (101 MHz, CDCl$_3$)** δ 155.6, 143.1, 139.5, 132.5, 127.0, 125.9, 125.1, 79.7, 52.2, 40.4, 39.9, 28.6. **HRMS** (ESI) Calcd for $C_{14}H_{18}ClNO_2$ [M-tBu+2H]$^+$: 212.0473; Found: 212.0473.

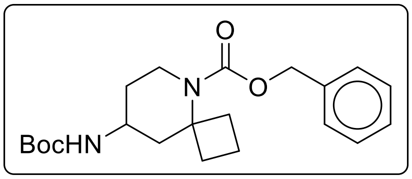

benzyl 8-((*tert*-butoxycarbonyl)amino)-5-azaspiro[3.5]nonane-5-carboxylate (58-SFC): Synthesized from **58-ketone** according to the general procedures for amine and Boc amine synthesis. 0.17 mmol scale: 40% yield (25.4 mg). Colorless oil. **^{1}H NMR (400 MHz, CDCl$_3$)** δ 7.41 – 7.28 (m, 5H), 5.16 – 5.01 (m, 2H), 4.36 (s, 1H), 3.92 (ddd, J = 14.0, 4.4, 2.3 Hz, 1H), 3.81 (s, 1H), 2.79 – 2.61 (m, 2H), 2.22 (ddd, J = 12.6, 4.5, 1.5 Hz, 1H), 2.18 – 2.10 (m, 1H), 2.10 – 1.99 (m, 1H), 1.94 (qd, J = 10.2, 1.8 Hz, 1H), 1.88 – 1.78 (m, 1H), 1.69 (ddt, J = 10.8, 7.7, 5.9 Hz, 2H), 1.44 (s, 9H), 1.40 – 1.30 (m, 1H), 1.10 (qd, J = 12.6, 4.3 Hz, 1H). **^{13}C NMR (101 MHz, CDCl$_3$)** δ 155.7, 155.2, 136.9, 128.6, 128.1 (two overlapping peaks), 79.6, 66.9, 59.7, 45.7, 40.9, 39.1, 34.3, 32.4, 31.7, 28.5, 14.6. **HRMS** (ESI) Calcd for $C_{21}H_{30}N_2O_4$ [M-Boc+2H]$^+$: 275.1754; Found: 275.1756.

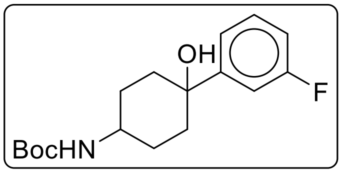

***tert*-butyl (4-(3-fluorophenyl)-4-hydroxycyclohexyl)carbamate (63-SFC):** Synthesized from **63-ketone** according to the general procedures for amine and Boc amine synthesis as a mixture of diastereomers

(~2:1). 0.17 mmol scale: 18% yield (12.2 mg). White solid. **^{1}H NMR (600 MHz, CDCl$_3$)** zδ 7.31 (dqd, J = 16.8, 8.4, 6.4 Hz, 1H), 7.25 – 7.18 (m, 2H), 6.99 – 6.91 (m, 1H), 4.55 (d, J = 70.0 Hz, 1H), 3.52 (s, 1H), 2.06 (dd, J = 10.1, 3.3 Hz, 1H), 1.97 – 1.79 (m, 4H), 1.68 (s, 4H), 1.45 (d, J = 3.0 Hz, 9H). **^{13}C NMR (151 MHz, CDCl$_3$)** δ 164.0, 163.9, 162.3, 162.3, 155.4, 151.7, 151.7, 130.1, 130.1, 130.0, 129.9, 120.7, 120.1, 120.1, 114.2, 114.1, 114.0, 113.8, 112.6, 112.4, 112.0, 111.9, 79.4, 72.48, 72.47, 72.1, 72.1, 49.1, 37.8, 28.9, 28.58, 28.57. **^{19}F NMR (565 MHz, CDCl$_3$)** δ -113.07 – -113.20 (m), -113.25 – -113.35 (m). **HRMS** (ESI) Calcd for $C_{17}H_{24}FNO_3$ [M-tBu-H$_2$O+2H]$^+$: 236.1081; Found: 236.1082.

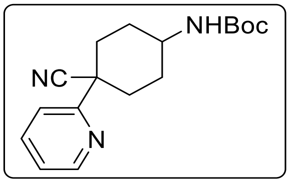

***tert*-butyl (4-cyano-4-(pyridin-2-yl)cyclohexyl)carbamate (64-SFC):** Synthesized from **64-ketone** according to the general procedure for amine and Boc-amine synthesis as a mixture of diastereomers (~2:1). 0.17 mmol scale: 46% yield (23.7 mg). White solid. **^{1}H NMR (600 MHz, CDCl$_3$)** δ 8.63 – 8.57 (m, 1H), 7.80 – 7.69 (m, 1H), 7.61 (dd, J = 52.3, 8.1 Hz, 1H), 7.30 – 7.21 (m, 1H), 5.04 – 4.18 (m, 1H), 4.04 – 3.31 (m, 1H), 2.33 (td, J = 13.4, 3.6 Hz, 1H), 2.22 – 1.96 (m, 5H), 1.90 (dd, J = 14.4, 4.1 Hz, 1H), 1.72 – 1.62 (m, 1H), 1.45 (s, 8H). **^{13}C NMR (151 MHz, CDCl$_3$)** δ 158.6, 158.4, 155.4, 155.3, 149.7, 149.4, 137.9, 137.4, 123.3, 123.1, 122.1, 121.2, 120.6, 79.5, 48.4, 46.0, 45.6, 44.5, 35.0, 30.9, 30.4, 28.6, 28.5, 27.7. **HRMS** (ESI) Calcd for $C_{17}H_{23}N_3O_2$ [M-tBu+2H]$^+$: 246.1237; Found: 246.1237.

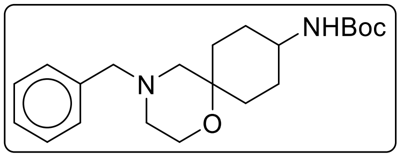

***tert*-butyl (4-benzyl-1-oxa-4-azaspiro[5.5]undecan-9-yl)carbamate (65-SFC):** Synthesized from **65-ketone** according to the general procedure for amine and Boc-amine synthesis as a mixture of diastereomers (~2:1). 0.1 mmol scale: 47% yield (28.5 mg). White solid. **^{1}H NMR (600 MHz, CDCl$_3$)** δ 7.34 – 7.28 (m, 4H), 7.27 – 7.21 (m, 1H), 4.42 (d, J = 8.6 Hz, 1H), 3.71 (dt, J = 33.9, 4.9 Hz, 2H), 3.44 (d, J = 16.2 Hz, 3H), 2.41 (d, J = 26.5 Hz, 2H), 2.29 – 2.03 (m, 3H), 1.92 – 1.81 (m, 1H), 1.81 – 1.57 (m, 3H), 1.43 (d, J = 5.4 Hz, 10H), 1.19 (td, J = 12.7, 7.1 Hz, 2H). **^{13}C NMR (151 MHz, CDCl$_3$)** δ 155.4, 138.5, 128.9, 128.4, 128.4, 127.2, 79.1, 72.0, 70.9, 63.2, 63.0, 61.1, 60.7, 60.1, 54.2, 53.8, 49.7, 48.1, 31.8, 30.7, 28.57, 28.55, 27.8. **HRMS** (ESI) Calcd for $C_{21}H_{32}N_2O_3$ [M+H]$^+$: 361.2486; Found: 361.2487.

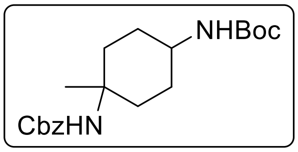

benzyl *tert*-butyl (1-methylcyclohexane-1,4-diyl)dicarbamate (66-SFC): Synthesized from **66-ketone** according to the general procedure for amine and Boc-amine synthesis as a mixture of diastereomers (~2:1). 0.17 mmol scale: 46% yield (28.6 mg). Colorless oil. **^{1}H NMR (600 MHz, CDCl$_3$)** δ 7.39 – 7.28 (m, 5H), 5.04 (d, J = 4.9 Hz, 2H), 4.61 (d, J = 82.6 Hz, 1H), 4.45 (d, J = 55.0 Hz, 1H), 3.47 (d, J = 73.9 Hz, 1H), 2.12 (d, J = 13.8 Hz, 1H), 1.86 – 1.67 (m, 3H), 1.43 (d, J = 4.6 Hz, 9H), 1.40 – 1.21 (m, 7H). **^{13}C NMR (151 MHz, CDCl$_3$)** δ 155.4, 154.7, 136.8, 128.68, 128.66, 128.24, 128.22, 128.2, 79.4, 66.3, 53.6, 52.0,

51.6, 49.0, 48.0, 35.3, 34.5, 28.63, 28.55, 28.5, 28.40, 28.36, 27.4. **HRMS** (ESI) Calcd for $C_{20}H_{30}N_2O_4$ [M-Boc+2H]$^+$: 263.1754; Found: 263.1755.

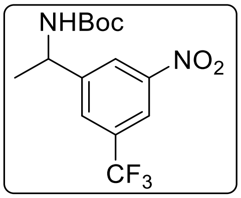

***tert*-butyl (1-(3-nitro-5-(trifluoromethyl)phenyl)ethyl)carbamate (67-SFC):** Synthesized from **67-ketone** according to the general procedures for amine and Boc amine synthesis. Racemic, 0.17 mmol scale: 50% yield (28.5 mg). White solid. Enzymatic reaction was conducted using the general procedure for scale-up biocatalysis reactions using the same ketone substrate with ATA-415 (R-selective). Enzymatic, 0.1125 mmol scale: 40% yield (15.1 mg). **SFC Data** Peak 1: 3999075 (1.26 min), Peak 2: not detected (1.40 min); >99.9% *ee*. **^{1}H NMR (600 MHz, CDCl$_3$)** δ 8.39 – 8.34 (m, 2H), 7.88 (d, J = 2.1 Hz, 1H), 4.98 (d, J = 6.7 Hz, 1H), 4.90 (s, 1H), 1.50 (d, J = 7.1 Hz, 3H), 1.42 (s, 9H). **^{13}C NMR (151 MHz, CDCl$_3$)** δ 156.0, 155.0, 148.8, 132.6 (q, *J* = 34.0 Hz), 128.7 (q, *J* = 3.5 Hz), 124.02, 123.0 (q, *J* = 273.0 Hz), 119.6 (q, *J* = 3.8 Hz), 80.6, 50.0, 28.4, 22.8. **^{19}F NMR (376 MHz, CDCl$_3$)** -62.82 (s). **HRMS** (ESI) Calcd for $C_{14}H_{17}F_3N_2O_4$ [M-Boc+2H]$^+$: 235.0689; Found: 235.0683 (amine fragment). Calcd for $C_{14}H_{17}F_3N_2O_4$ [M-tBu+2H]$^+$: 279.0587; Found: 279.0581 (carbamic acid fragment).

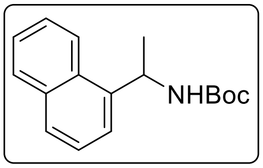

***tert*-butyl (1-(naphthalen-1-yl)ethyl)carbamate (68-SFC):** Synthesized from **68-ketone** according to the general procedures for amine and Boc amine synthesis. Racemic, 0.17 mmol scale: 56% yield (25.8 mg). White solid. Enzymatic reaction was conducted using the general procedure for scale-up biocatalysis reactions using the same ketone substrate with ATA-R123 (R-selective). Enzymatic, 0.1125 mmol scale: 46% yield (14.2 mg). **SFC Data** Peak 1: 1004500 (1.63 min), Peak 2: not detected (1.79 min); >99.9% *ee*. **^{1}H NMR (499 MHz, CDCl$_3$)** δ 8.14 (d, J = 8.5 Hz, 1H), 7.86 (dd, J = 8.1, 1.5 Hz, 1H), 7.80 – 7.74 (m, 1H), 7.57 – 7.41 (m, 4H), 5.61 (s, 1H), 4.87 (s, 1H), 1.62 (d, J = 6.7 Hz, 3H), 1.44 (s, 9H). The NMR data are in alignment with previously reported spectra from Enamine (*via* CAS SciFinder).

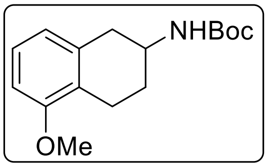

tert-butyl (5-methoxy-1,2,3,4-tetrahydronaphthalen-2-yl)carbamate (69-SFC): Synthesized from **69-ketone** according to the general procedures for amine and Boc amine synthesis. Racemic, 0.17 mmol scale: 53% yield (24.8 mg). White solid. Enzymatic reaction was conducted using the general procedure for scale-

up biocatalysis reactions using the same ketone substrate with ATA-237 (S-selective). Enzymatic, 0.1125 mmol scale: 16% yield (4.9 mg). **SFC Data** Peak 1: 2028982 (1.49 min), Peak 2: 1310716 (1.61 min); 21.5% ee. **^{1}H NMR (600 MHz, $CDCl_3$)** δ 7.10 (t, J = 7.9 Hz, 1H), 6.68 (t, J = 7.3 Hz, 2H), 4.59 (s, 1H), 3.94 (d, J = 15.0 Hz, 1H), 3.82 (s, 3H), 3.09 (dd, J = 16.3, 4.9 Hz, 1H), 2.81 (dt, J = 18.6, 6.1 Hz, 1H), 2.69 (dt, J = 18.0, 7.3 Hz, 1H), 2.62 (dd, J = 16.2, 7.9 Hz, 1H), 2.04 (dtdd, J = 12.6, 6.2, 3.0, 1.4 Hz, 1H), 1.79 – 1.66 (m, 1H), 1.45 (s, 9H). **^{13}C NMR (151 MHz, $CDCl_3$)** δ 157.3, 155.5, 135.7, 126.5, 124.6, 121.7, 107.4, 79.3, 55.4, 45.9, 36.2, 28.6, 21.2. **HRMS** (ESI) Calcd for $C_{16}H_{23}NO_3$ [M-Boc-NH_3+H]$^+$: 161.0961; Found: 161.0961 (elimination fragment); Calcd for $C_{16}H_{23}NO_3$ [M-Boc+2H]$^+$: 178.1226; Found: 178.1227 (amine fragment); Calcd for $C_{16}H_{23}NO_3$ [M-tBu+2H]$^+$: 222.1125; Found: 222.1125 (carbamic acid fragment).

7. References

1. M. J. Frisch, G. W. Trucks, H. B. Schlegel, G. E. Scuseria, M. A. Robb, J. R. Cheeseman, G. Scalmani, V. Barone, G. A. Petersson, H. Nakatsuji, X. Li, M. Caricato, A. V. Marenich, J. Bloino, B. G. Janesko, R. Gomperts, B. Mennucci, H. P. Hratchian, J. V. Ortiz, A. F. Izmaylov, J. L. Sonnenberg, Williams, F. Ding, F. Lipparini, F. Egidi, J. Goings, B. Peng, A. Petrone, T. Henderson, D. Ranasinghe, V. G. Zakrzewski, J. Gao, N. Rega, G. Zheng, W. Liang, M. Hada, M. Ehara, K. Toyota, R. Fukuda, J. Hasegawa, M. Ishida, T. Nakajima, Y. Honda, O. Kitao, H. Nakai, T. Vreven, K. Throssell, J. A. Montgomery Jr., J. E. Peralta, F. Ogliaro, M. J. Bearpark, J. J. Heyd, E. N. Brothers, K. N. Kudin, V. N. Staroverov, T. A. Keith, R. Kobayashi, J. Normand, K. Raghavachari, A. P. Rendell, J. C. Burant, S. S. Iyengar, J. Tomasi, M. Cossi, J. M. Millam, M. Klene, C. Adamo, R. Cammi, J. W. Ochterski, R. L. Martin, K. Morokuma, O. Farkas, J. B. Foresman and D. J. Fox, Gaussian 16 (Rev. C.02), Wallingford, CT, 2016.
2. G. Landrum et al., rdkit (Ver. 2023.3.3), 2023.
3. L. Jacot-Descombes, L. Turcani and K. Jorner, Morfeus (Ver. 0.7.2), 2022.
4. S. A. Wildman and G. M. Crippen, Prediction of Physicochemical Parameters by Atomic Contributions, *J. Chem. Inf. Comput. Sci.*, 1999, **39**, 868-873.
5. *CN Pat.,* CN107383026A, 2017.
6. A. K. Tripathi, S. Koul and S. C. Taneja, Microwave-assisted facile and efficient synthesis of benzopyran, *Indian J. Chem.*, 2009, **48B**, 301-304.
7. *WO Pat.,* WO2012116176 A2, 2012.
8. L. Dong, S. Aleem and C. A. Fink, Microwave-accelerated reductive amination between ketones and ammonium acetate, *Tetrahedron Lett.*, 2010, **51**, 5210-5212.
9. Y.-F. Zhang, X.-Y. Dong, J.-T. Cheng, N.-Y. Yang, L.-L. Wang, F.-L. Wang, C. Luan, J. Liu, Z.-L. Li, Q.-S. Gu and X.-Y. Liu, Enantioconvergent Cu-Catalyzed Radical C–N Coupling of Racemic Secondary Alkyl Halides to Access α-Chiral Primary Amines, *J. Am. Chem. Soc.*, 2021, **143**, 15413-15419.

8. ^{1}H, ^{13}C, and ^{19}F NMR Spectra

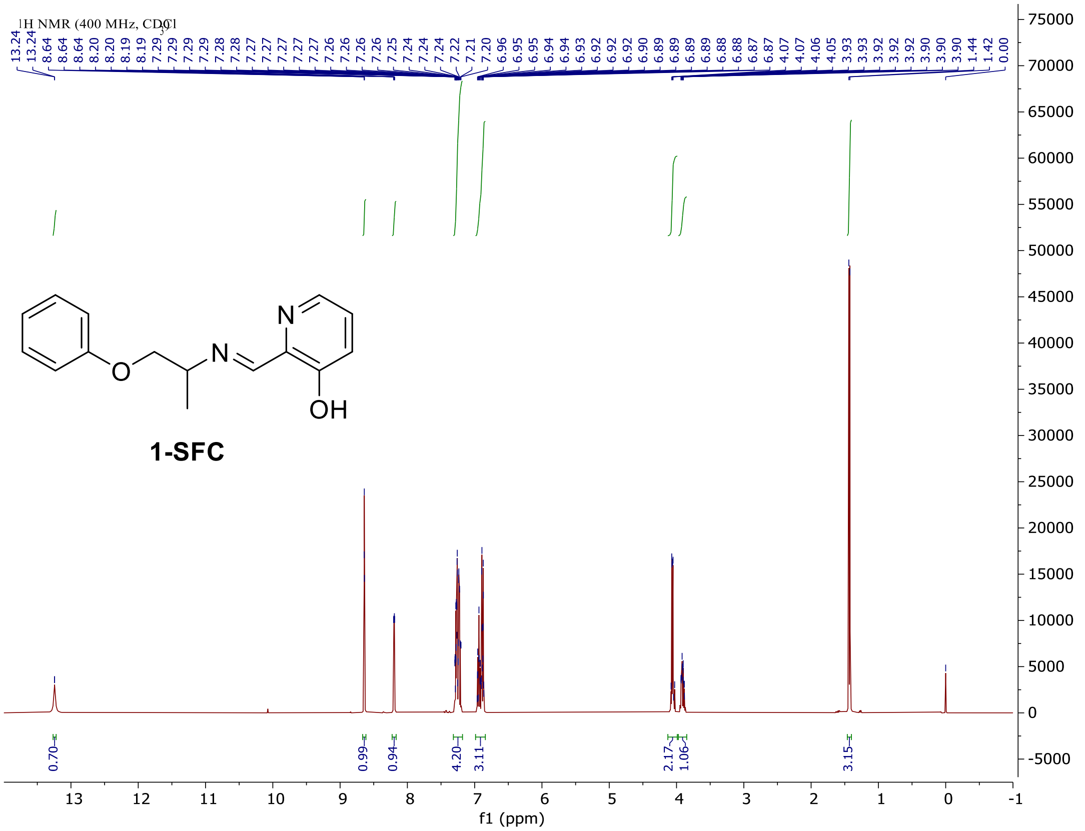

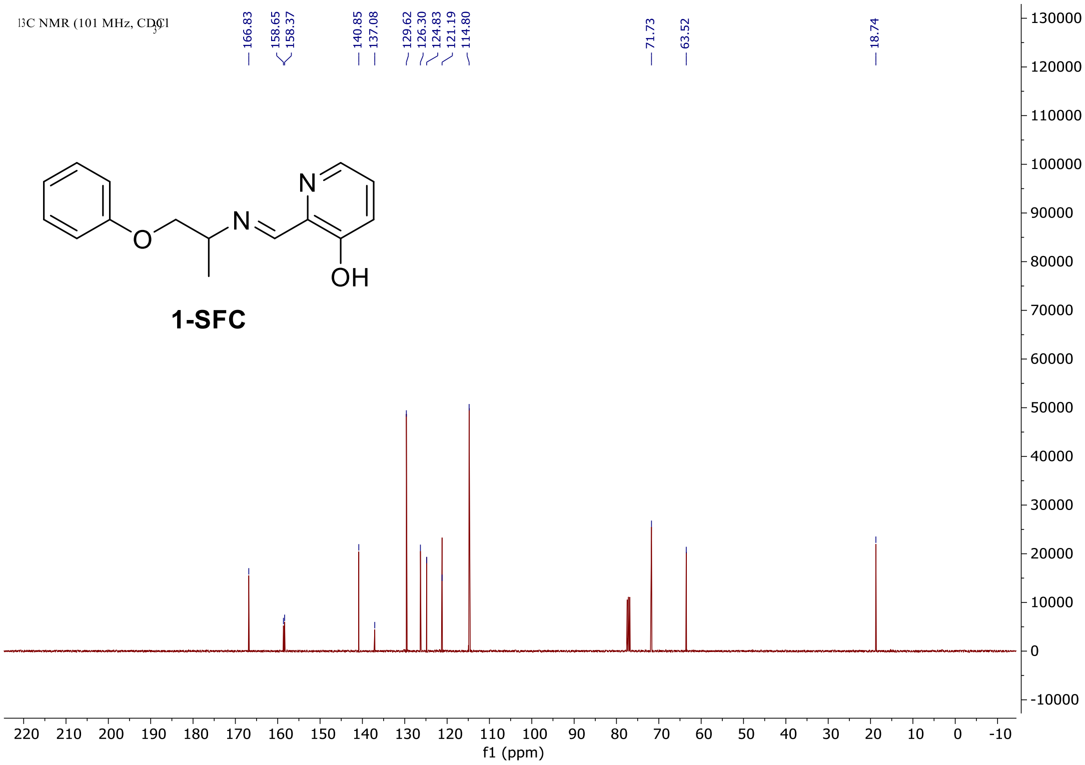

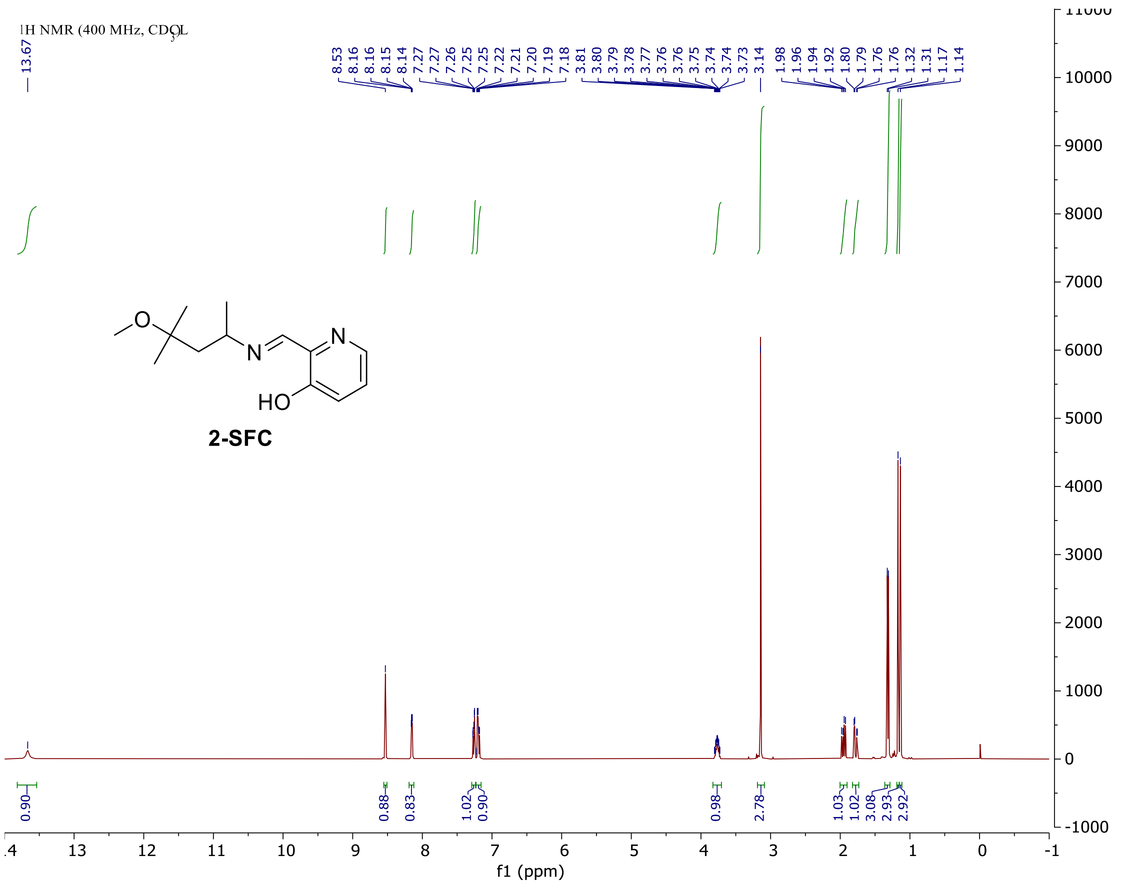

1H NMR (400 MHz, CDCl3)
13.67
8.53 8.16 8.16 8.15 8.14 7.27 7.27 7.26 7.25 7.25 7.22 7.21 7.20 7.19 7.18
3.81 3.80 3.79 3.78 3.77 3.76 3.76 3.75 3.74 3.74 3.73 3.14
1.98 1.96 1.94 1.92 1.80 1.79 1.76 1.76 1.32 1.31 1.17 1.14
N
HO
O
2-SFC
0.90 0.88 0.83 1.02 0.90 0.98 2.78 1.03 1.02 3.08 2.93 2.92
f1 (ppm)

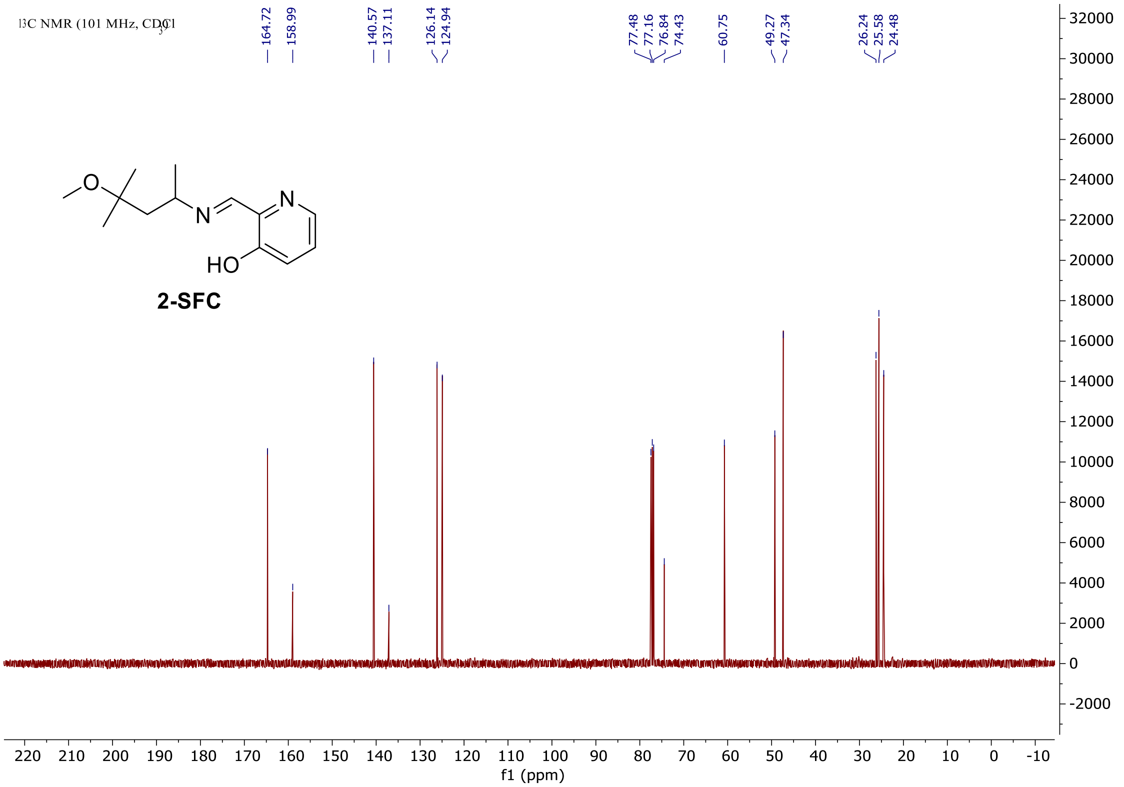

13C NMR (101 MHz, CDCl3)
164.72 158.99 140.57 137.11 126.14 124.94
77.48 77.16 76.84 74.43 60.75 49.27 47.34 26.24 25.58 24.48
N
HO
O
2-SFC
f1 (ppm)

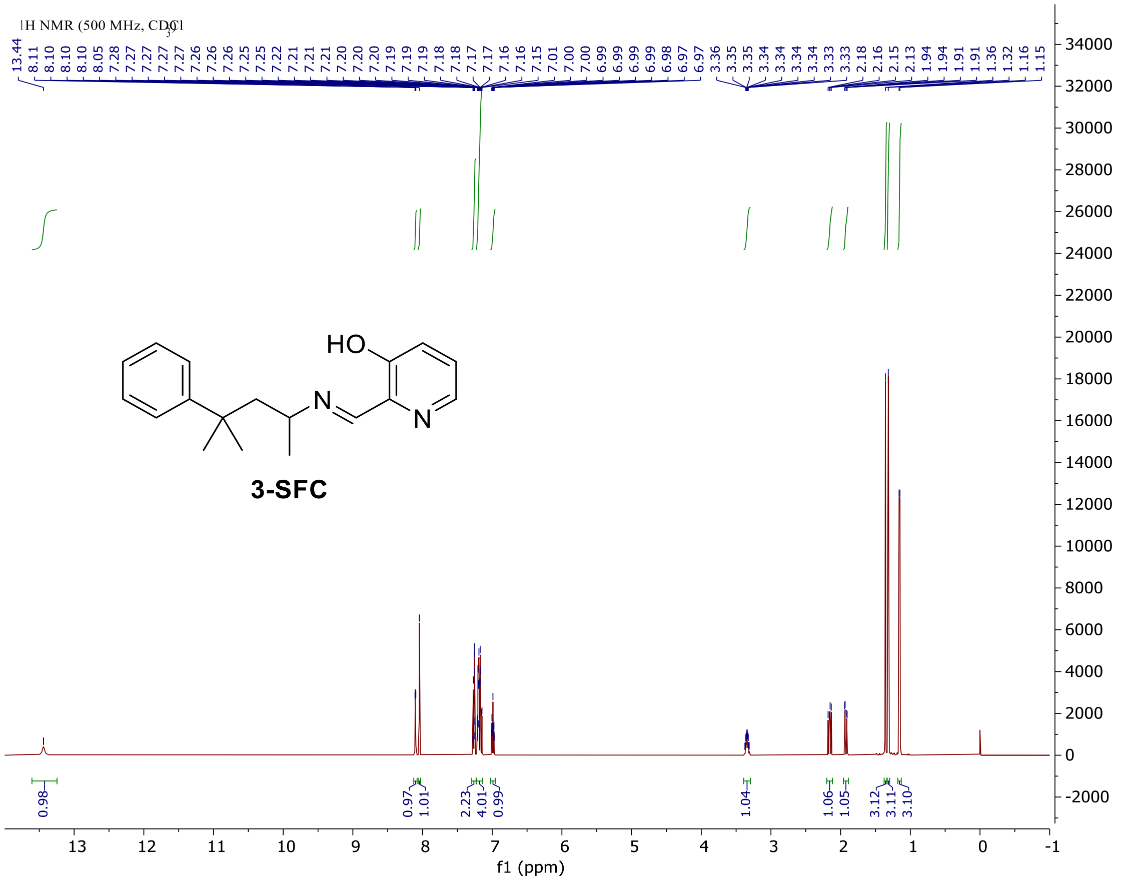
1H NMR (500 MHz, CDCl3)
3-SFC
HO
N
N
f1 (ppm)

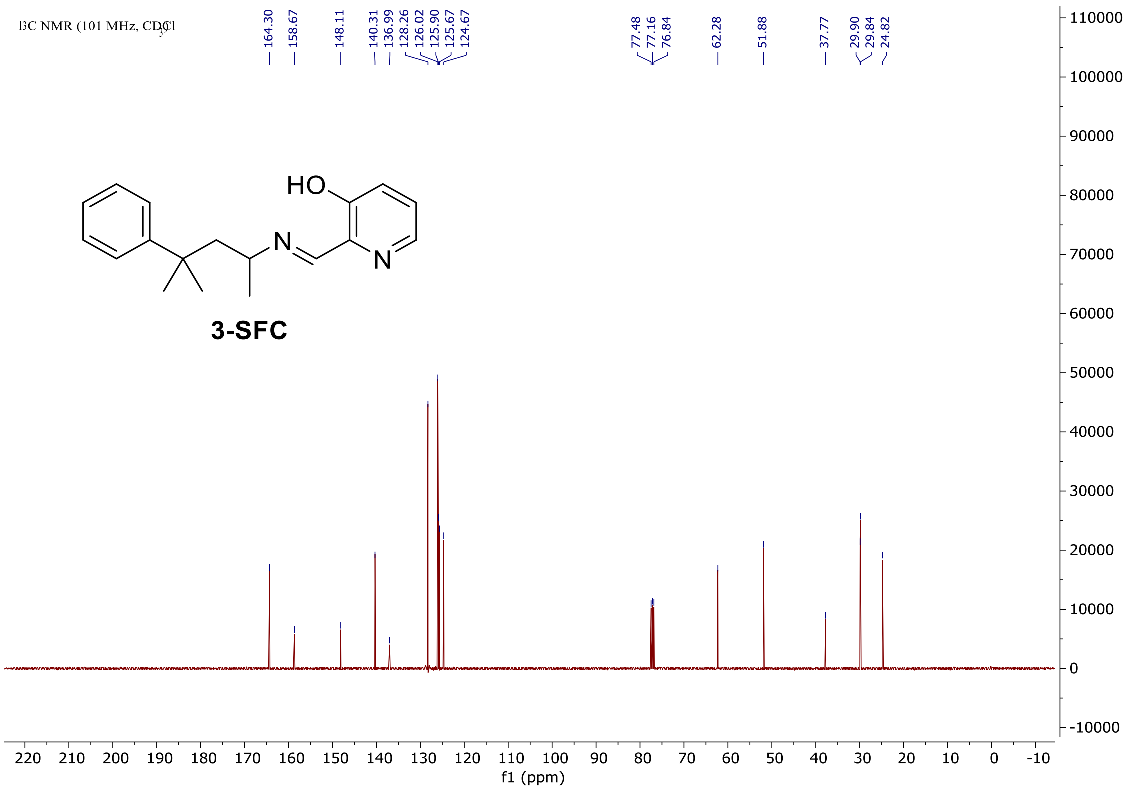
13C NMR (101 MHz, CDCl3)
164.30
158.67
148.11
140.31
136.99
128.26
126.02
125.90
125.67
124.67
77.48
77.16
76.84
62.28
51.88
37.77
29.90
29.84
24.82
3-SFC
HO
N
N
f1 (ppm)

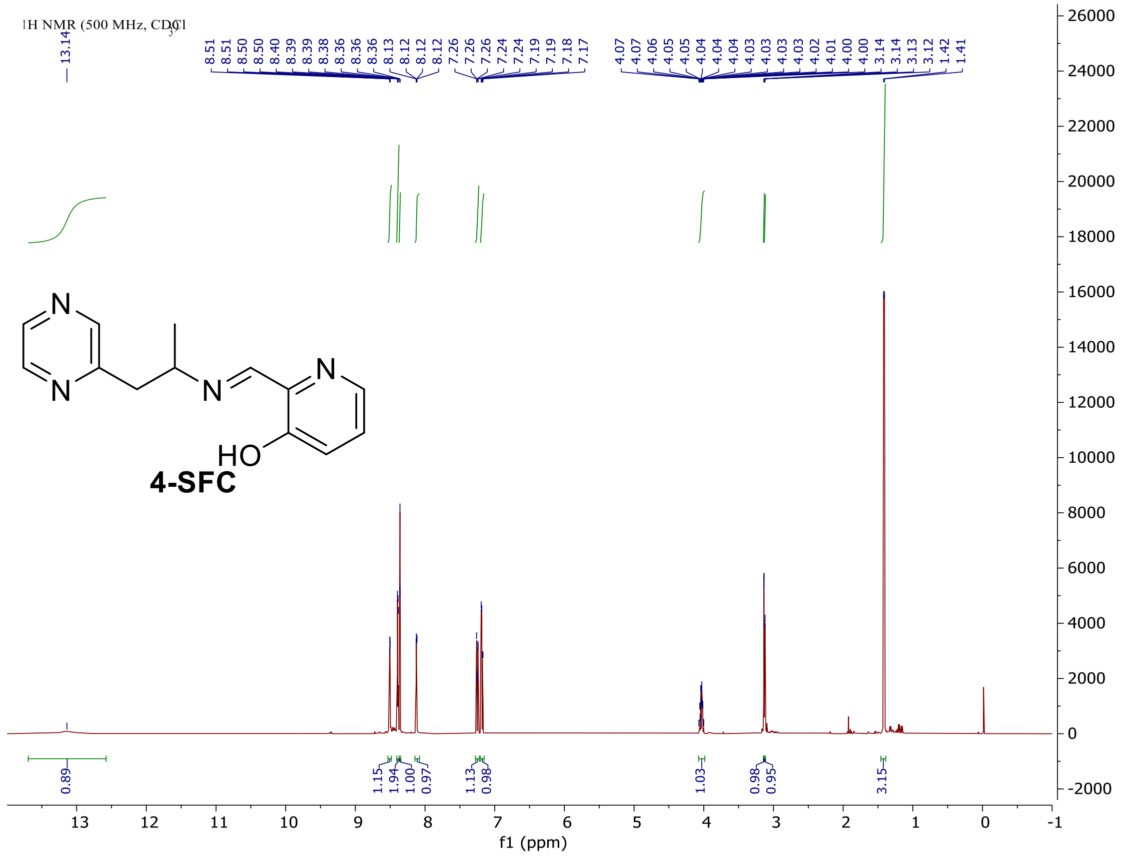
1H NMR (500 MHz, CDCl3
13.14
8.51 8.51 8.50 8.50 8.40 8.39 8.39 8.38 8.36 8.36 8.36 8.13 8.12 8.12 8.12 7.26 7.26 7.26 7.24 7.24 7.19 7.19 7.18 7.17
4.07 4.07 4.06 4.05 4.05 4.04 4.04 4.04 4.03 4.03 4.03 4.03 4.02 4.01 4.00 4.00 3.14 3.14 3.13 3.12 1.42 1.41
4-SFC
0.89
1.15 1.94 1.00 0.97
1.13 0.98
1.03
0.98 0.95
3.15
f1 (ppm)

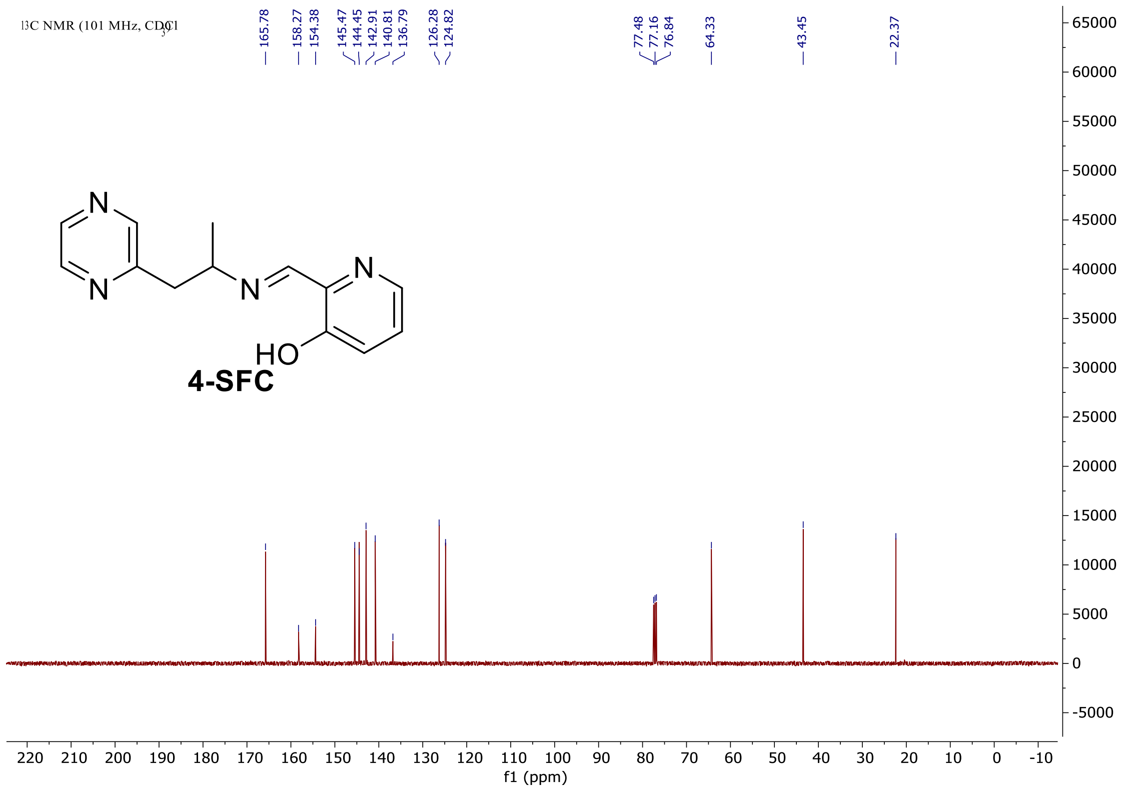
13C NMR (101 MHz, CDCl3
165.78
158.27
154.38
145.47 144.45 142.91 140.81 136.79
126.28 124.82
77.48 77.16 76.84
64.33
43.45
22.37
4-SFC
f1 (ppm)

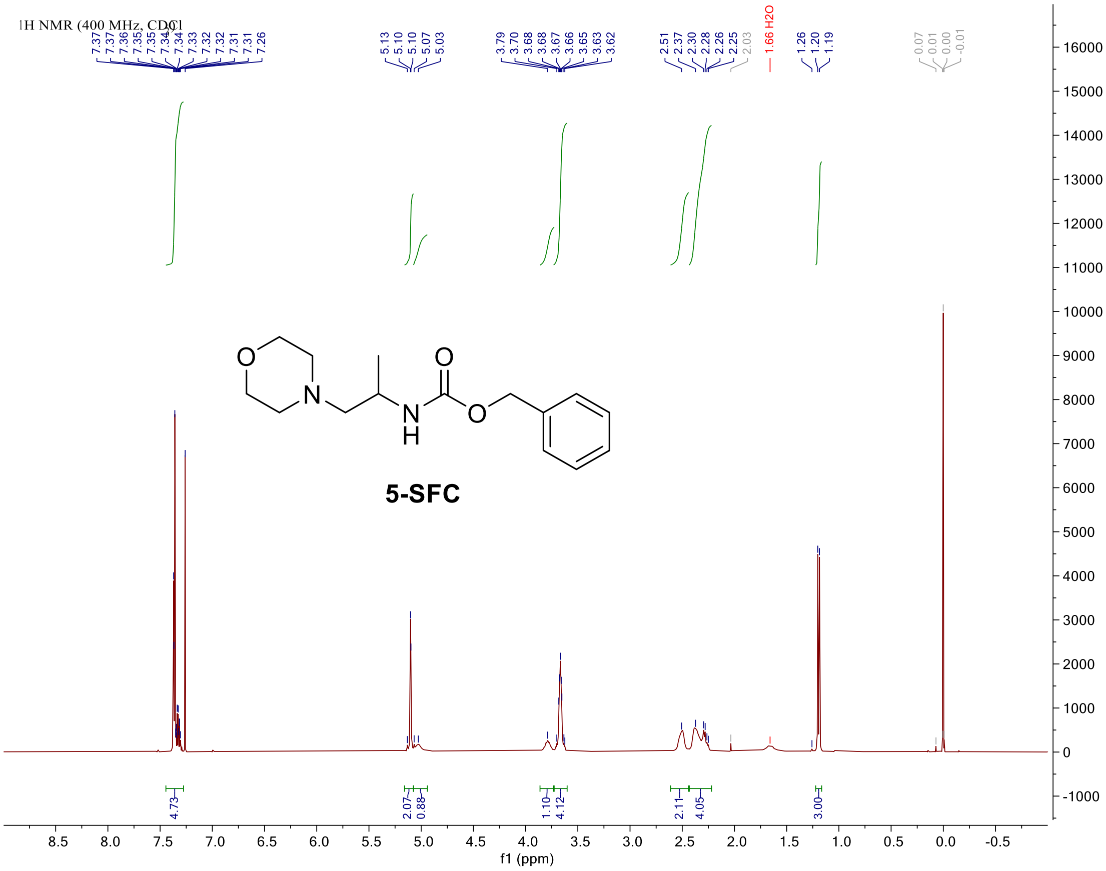
1H NMR (400 MHz, CDCl3)
7.37 7.37 7.36 7.35 7.35 7.34 7.34 7.33 7.32 7.32 7.31 7.31 7.26
5.13 5.10 5.10 5.07 5.03
3.79 3.70 3.68 3.68 3.67 3.66 3.65 3.63 3.62
2.51 2.37 2.30 2.28 2.26 2.25 2.03
1.66 H2O
1.26 1.20 1.19
0.07 0.01 0.00 -0.01
O
N
N
H
O
O
5-SFC
4.73
2.07
0.88
1.10
4.12
2.11
4.05
3.00
f1 (ppm)

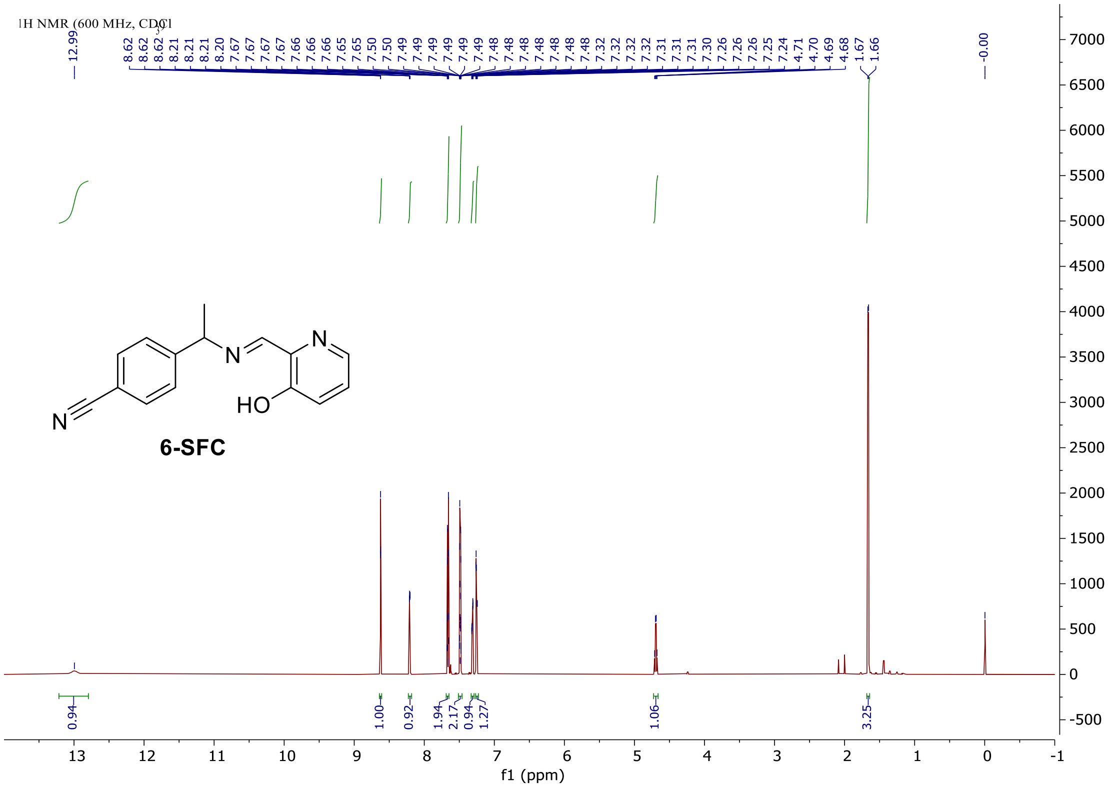
1H NMR (600 MHz, CDCl3)
12.99
8.62 8.62 8.62 8.21 8.21 8.20 7.67 7.67 7.67 7.66 7.66 7.66 7.65 7.50 7.49 7.49 7.49 7.49 7.49 7.49 7.48 7.48 7.48 7.48 7.48 7.48 7.32 7.32 7.32 7.32 7.31 7.31 7.30 7.26 7.26 7.25 7.24 4.71 4.70 4.69 4.68 1.67 1.66
-0.00
N
HO
6-SFC
0.94 1.00 0.92 1.94 2.17 0.94 1.27 1.06 3.25
f1 (ppm)

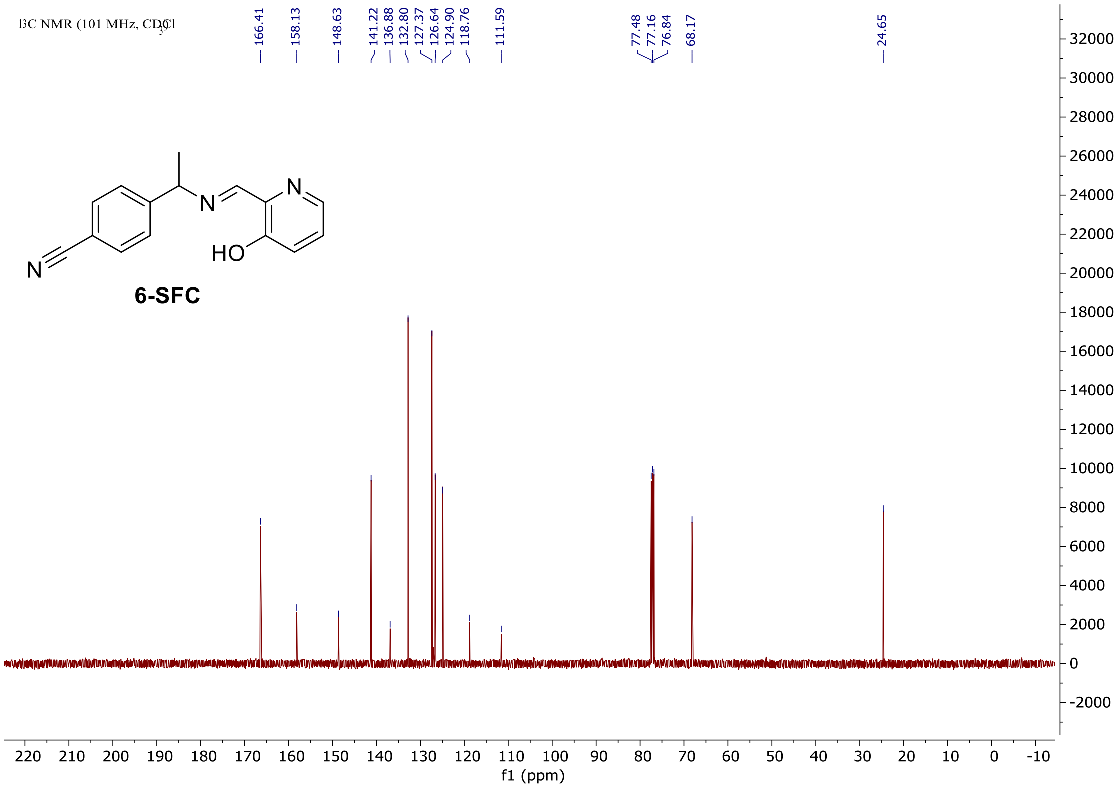
13C NMR (101 MHz, CDCl3)
166.41 158.13 148.63 141.22 136.88 132.80 127.37 126.64 124.90 118.76 111.59
77.48 77.16 76.84 68.17
24.65
N
HO
6-SFC
f1 (ppm)

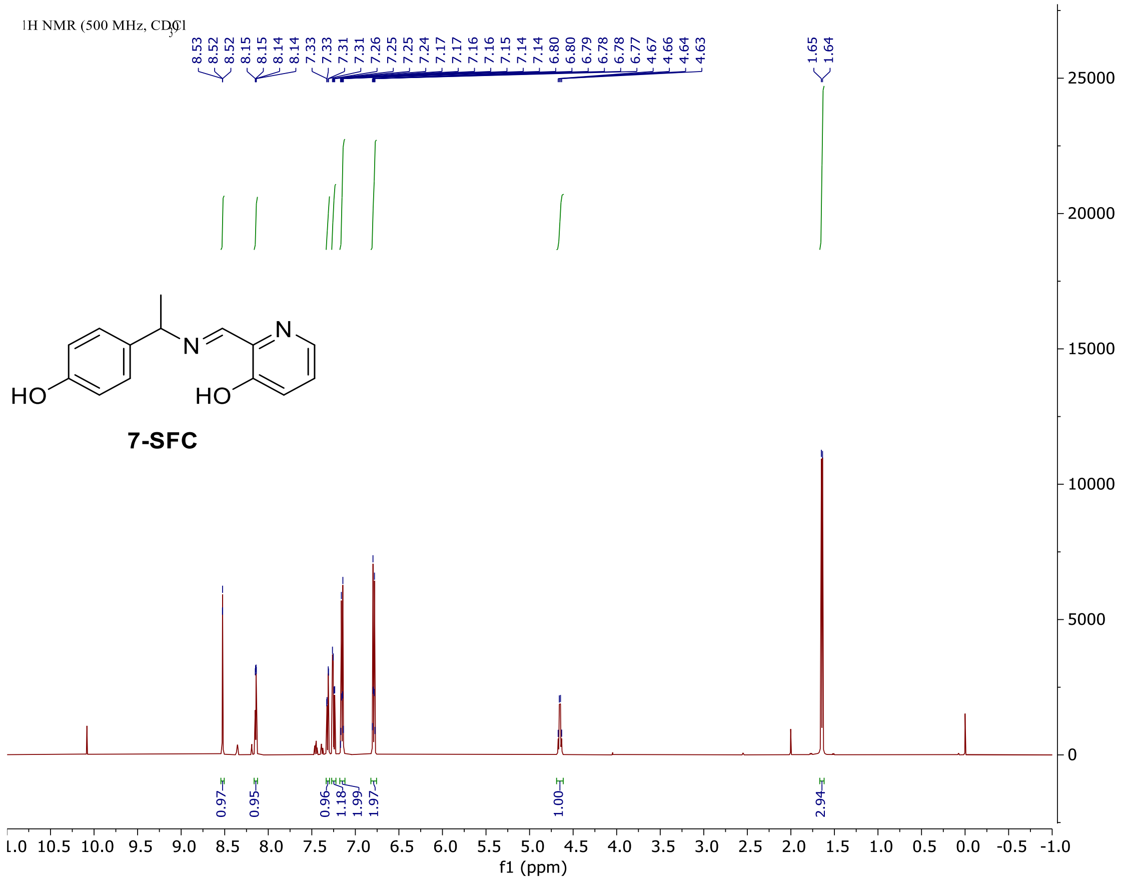
1H NMR (500 MHz, CDCl3)
8.53 8.52 8.52 8.15 8.15 8.14 8.14 7.33 7.33 7.31 7.31 7.26 7.25 7.25 7.24 7.17 7.17 7.16 7.16 7.15 7.14 7.14 6.80 6.80 6.79 6.78 6.78 6.77 4.67 4.66 4.64 4.63
1.65 1.64
HO
N
HO
7-SFC
0.97 0.95 0.96 1.18 1.99 1.97 1.00 2.94
f1 (ppm)

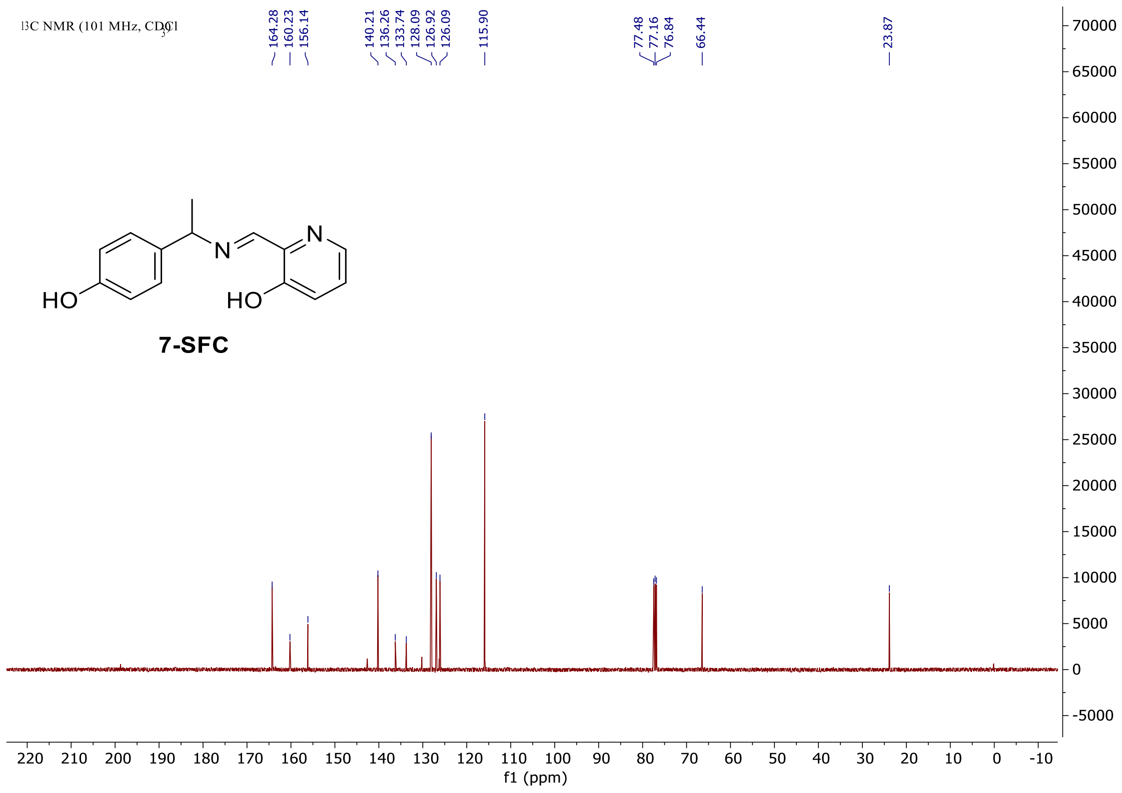
13C NMR (101 MHz, CDCl3)
164.28 160.23 156.14 140.21 136.26 133.74 128.09 126.92 126.09 115.90
77.48 77.16 76.84 66.44
23.87
HO
N
HO
7-SFC
f1 (ppm)

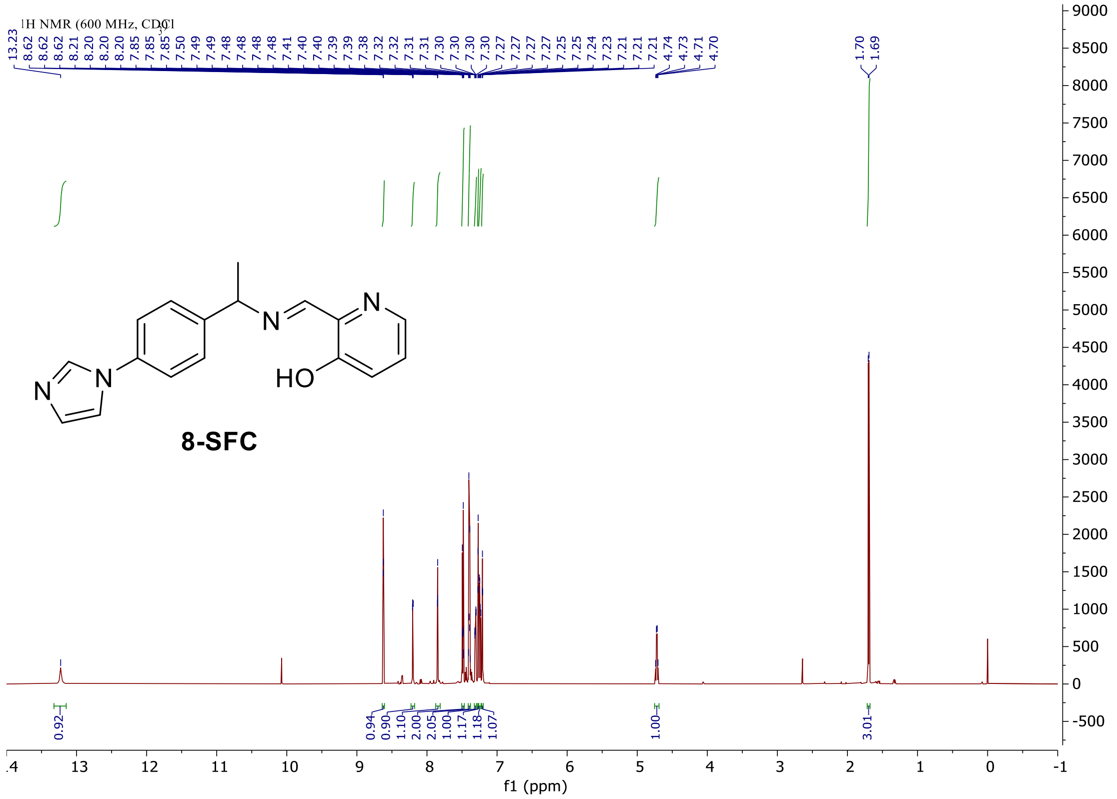
1H NMR (600 MHz, CDCl3
13.23 8.62 8.62 8.62 8.21 8.20 8.20 8.20 7.85 7.85 7.85 7.50 7.49 7.48 7.48 7.48 7.48 7.41 7.40 7.40 7.39 7.39 7.38 7.32 7.32 7.31 7.31 7.30 7.30 7.30 7.30 7.27 7.27 7.27 7.27 7.25 7.25 7.24 7.23 7.23 7.21 7.21 7.21 4.74 4.73 4.71 4.70
1.70 1.69
HO
N
8-SFC
0.92 0.94 0.90 1.10 2.00 2.05 1.00 1.17 1.18 1.07 1.00 3.01
f1 (ppm)

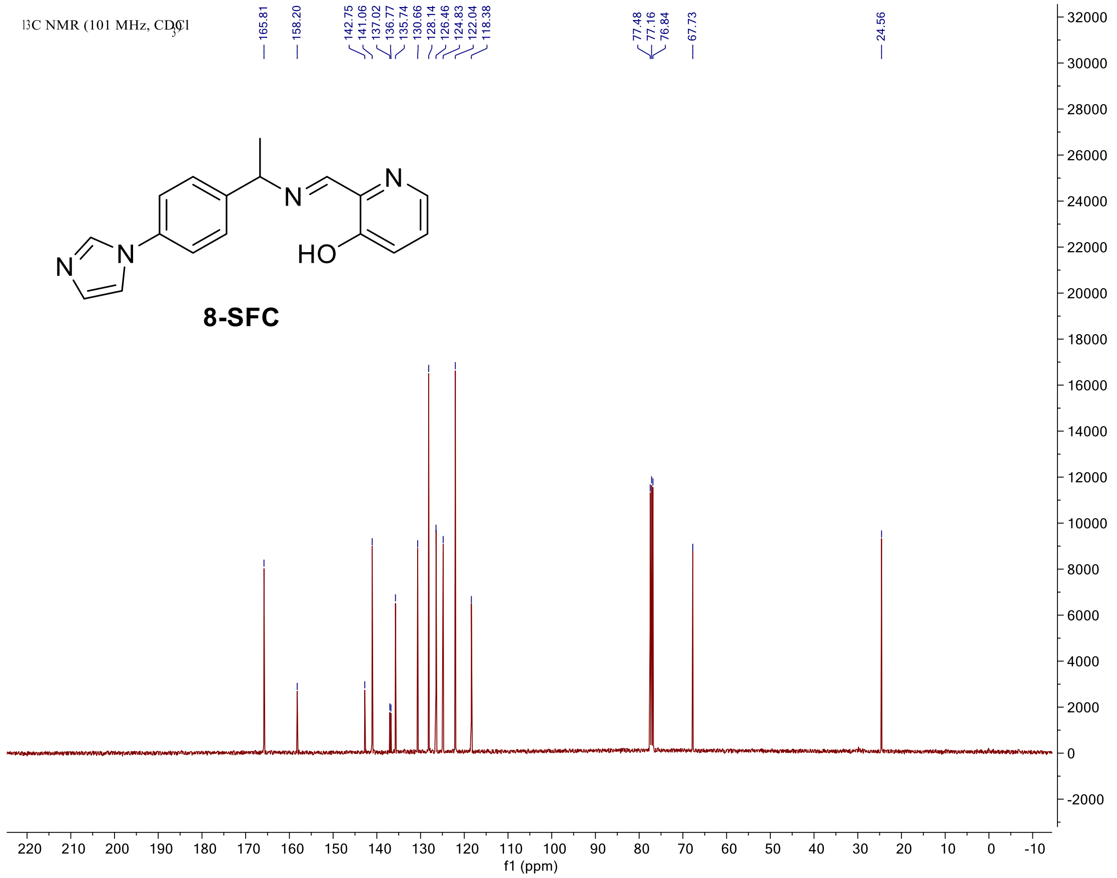
13C NMR (101 MHz, CDCl3
165.81
158.20
142.75 141.06 137.02 136.77 135.74 130.66 128.14 126.46 124.83 122.04 118.38
77.48 77.16 76.84
67.73
24.56
HO
N
8-SFC
f1 (ppm)

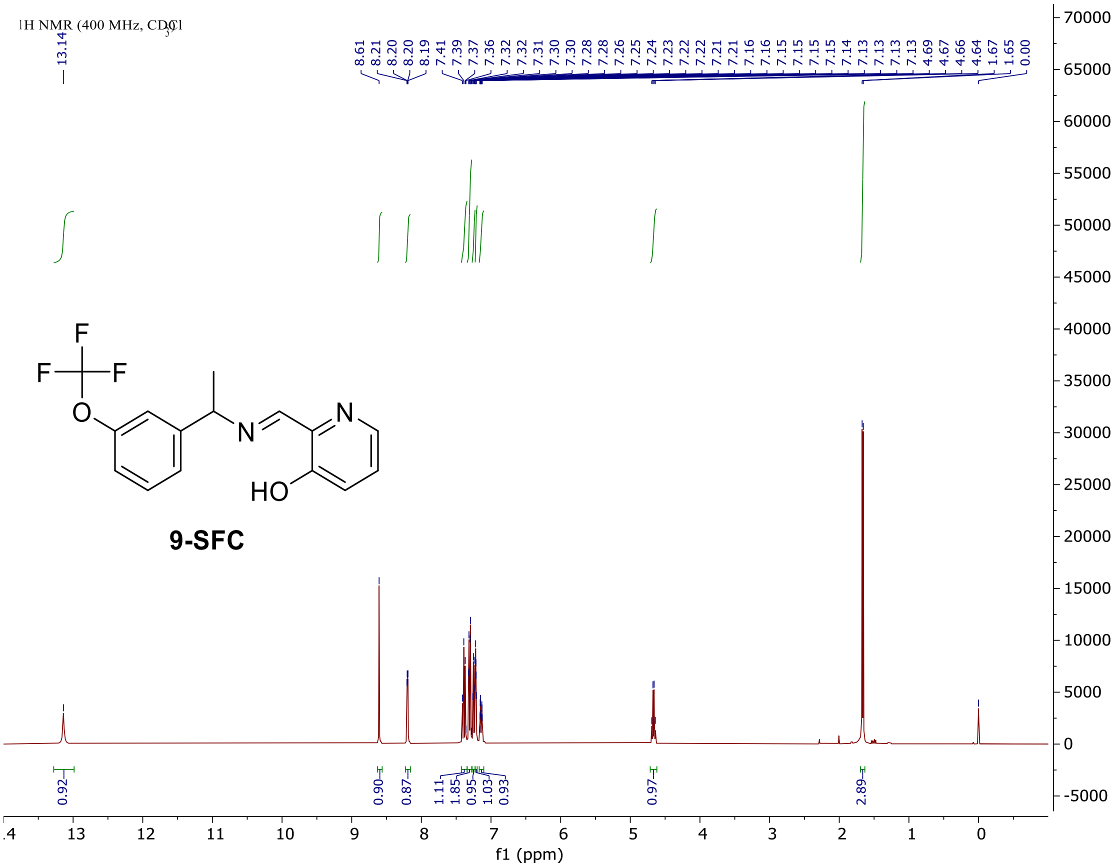
1H NMR (400 MHz, CDCl3
13.14
8.61 8.21 8.20 8.20 8.19 7.41 7.39 7.37 7.36 7.32 7.32 7.31 7.30 7.30 7.28 7.28 7.26 7.25 7.24 7.23 7.22 7.22 7.21 7.21 7.16 7.16 7.15 7.15 7.15 7.15 7.14 7.13 7.13 7.13 7.13 7.13 4.69 4.67 4.66 4.64 1.67 1.65 0.00
0.92 0.90 0.87 1.11 1.85 0.95 1.03 0.93 0.97 2.89
9-SFC
f1 (ppm)

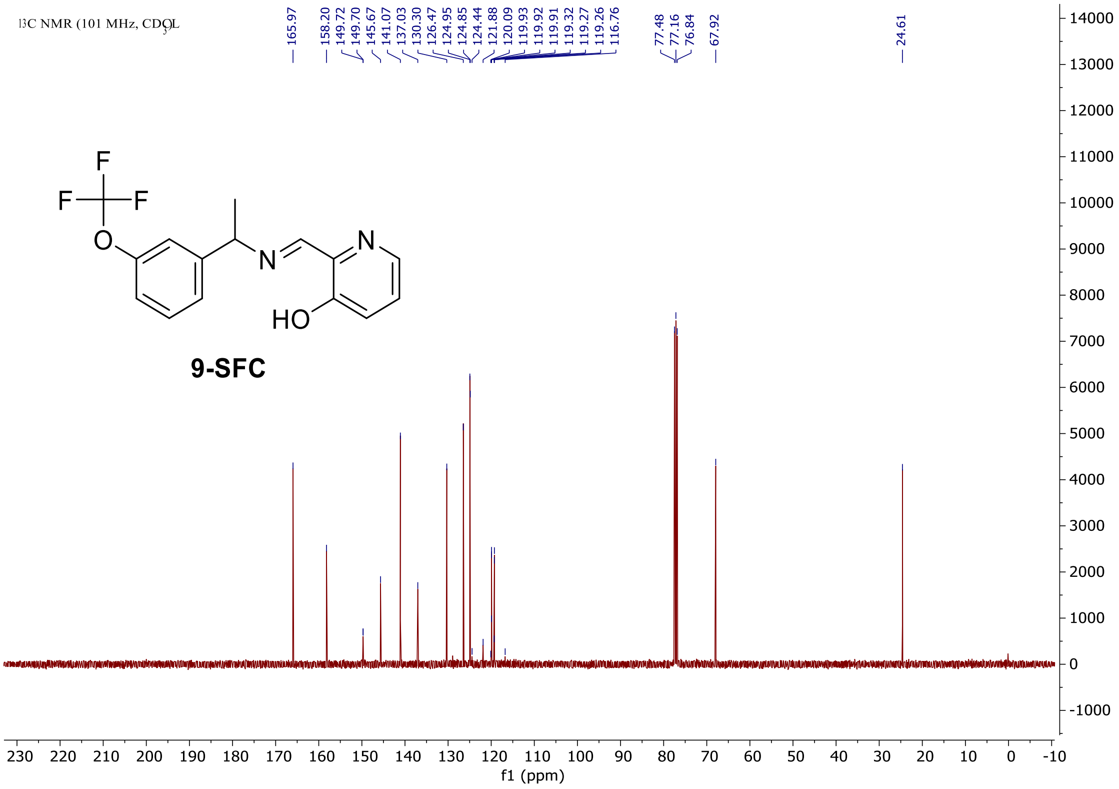
13C NMR (101 MHz, CDCl3
165.97 158.20 149.72 149.70 145.67 141.07 137.03 130.30 126.47 124.95 124.85 124.44 121.88 120.09 119.93 119.92 119.91 119.32 119.27 119.26 116.76
77.48 77.16 76.84 67.92
24.61
9-SFC
f1 (ppm)

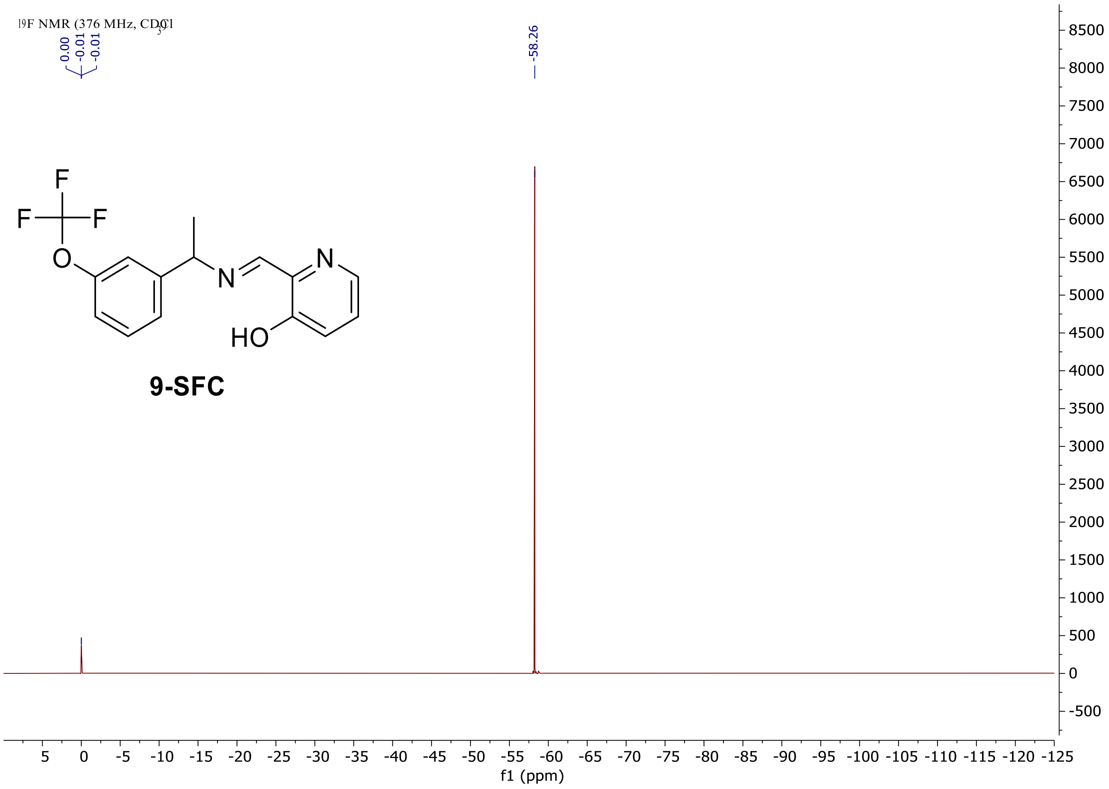
19F NMR (376 MHz, CDCl3)
0.00
-0.01
-0.01
-58.26
9-SFC
f1 (ppm)

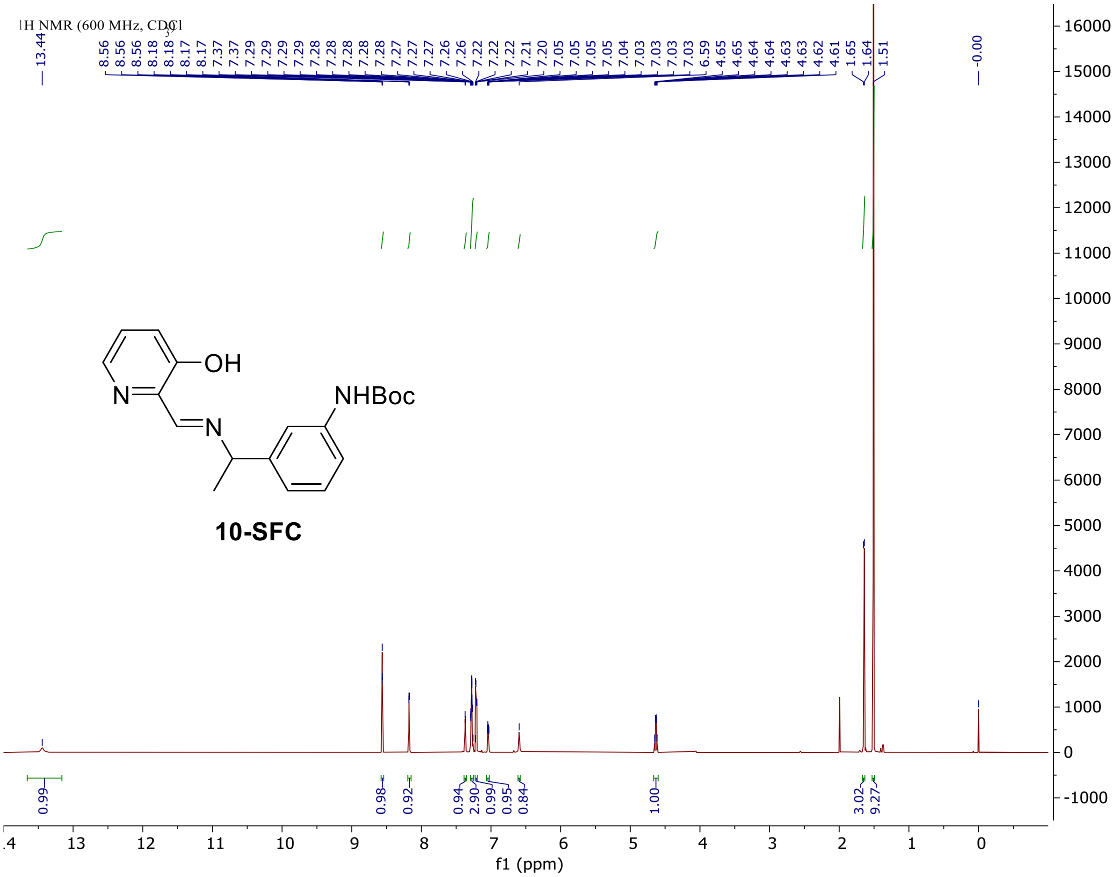

1H NMR (600 MHz, CDCl3)
13.44
8.56 8.56 8.56 8.18 8.18 8.17 8.17 7.37 7.37 7.29 7.29 7.29 7.29 7.28 7.28 7.28 7.28 7.27 7.27 7.27 7.26 7.26 7.22 7.22 7.22 7.22 7.21 7.20 7.05 7.05 7.05 7.05 7.04 7.03 7.03 7.03 7.03 6.59 4.65 4.65 4.64 4.64 4.63 4.63 4.62 4.61 1.65 1.64 1.51
-0.00
0.99 0.98 0.92 0.94 2.90 0.99 0.95 0.84 1.00 3.02 9.27
OH
N
NHBoc
N
10-SFC
f1 (ppm)

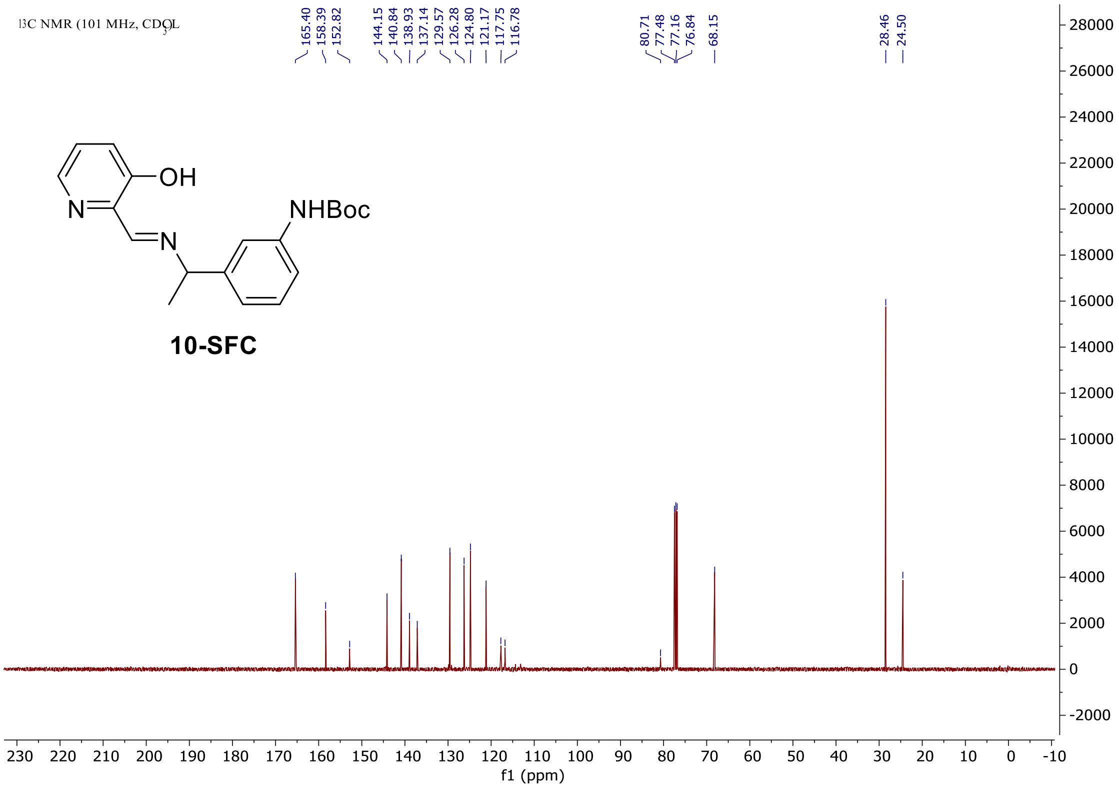

13C NMR (101 MHz, CDCl3)
165.40 158.39 152.82 144.15 140.84 138.93 137.14 129.57 126.28 124.80 121.17 117.75 116.78
80.71 77.48 77.16 76.84 68.15
28.46 24.50
OH
N
NHBoc
N
10-SFC
f1 (ppm)

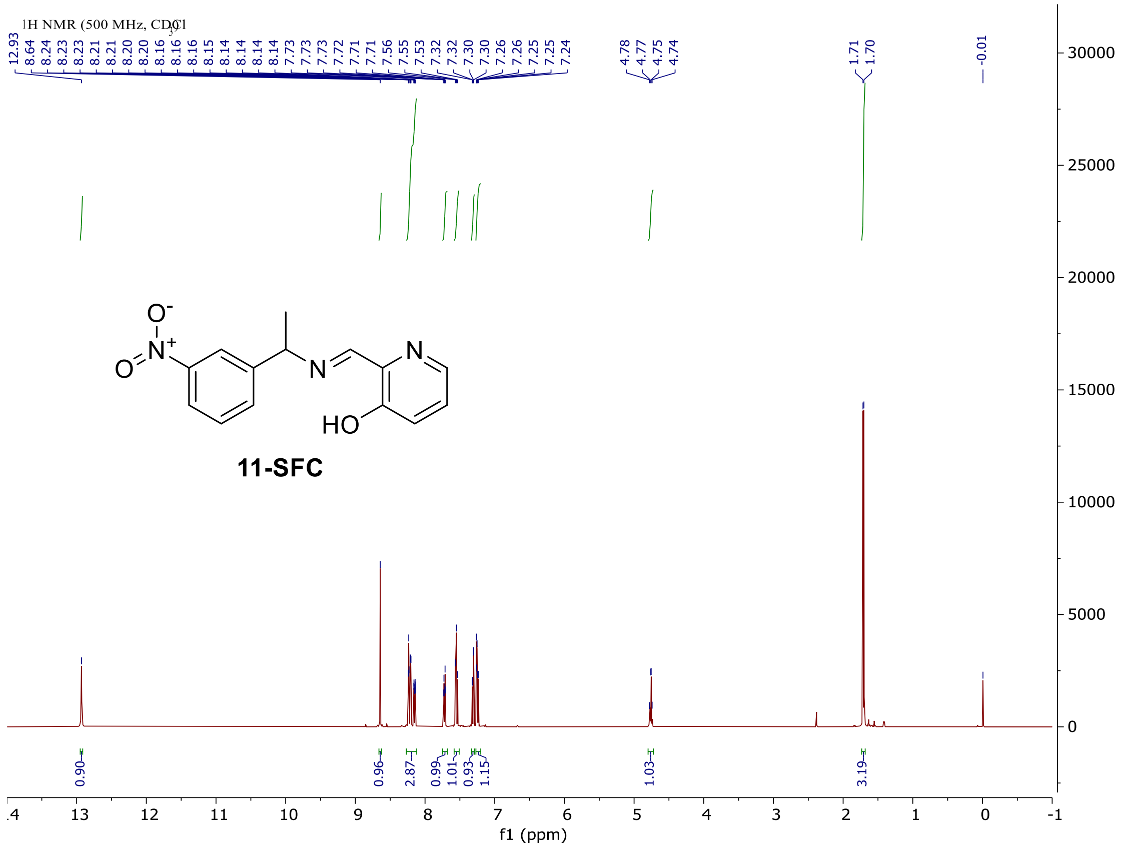

1H NMR (500 MHz, CDCl3
12.93
8.64
8.24
8.23
8.23
8.21
8.21
8.20
8.20
8.16
8.16
8.16
8.15
8.14
8.14
8.14
8.14
7.73
7.73
7.73
7.72
7.71
7.71
7.56
7.55
7.53
7.32
7.32
7.30
7.30
7.26
7.26
7.25
7.24
4.78
4.77
4.75
4.74
1.71
1.70
-0.01
0.90
0.96
2.87
0.99
1.01
0.93
1.15
1.03
3.19
11-SFC
f1 (ppm)

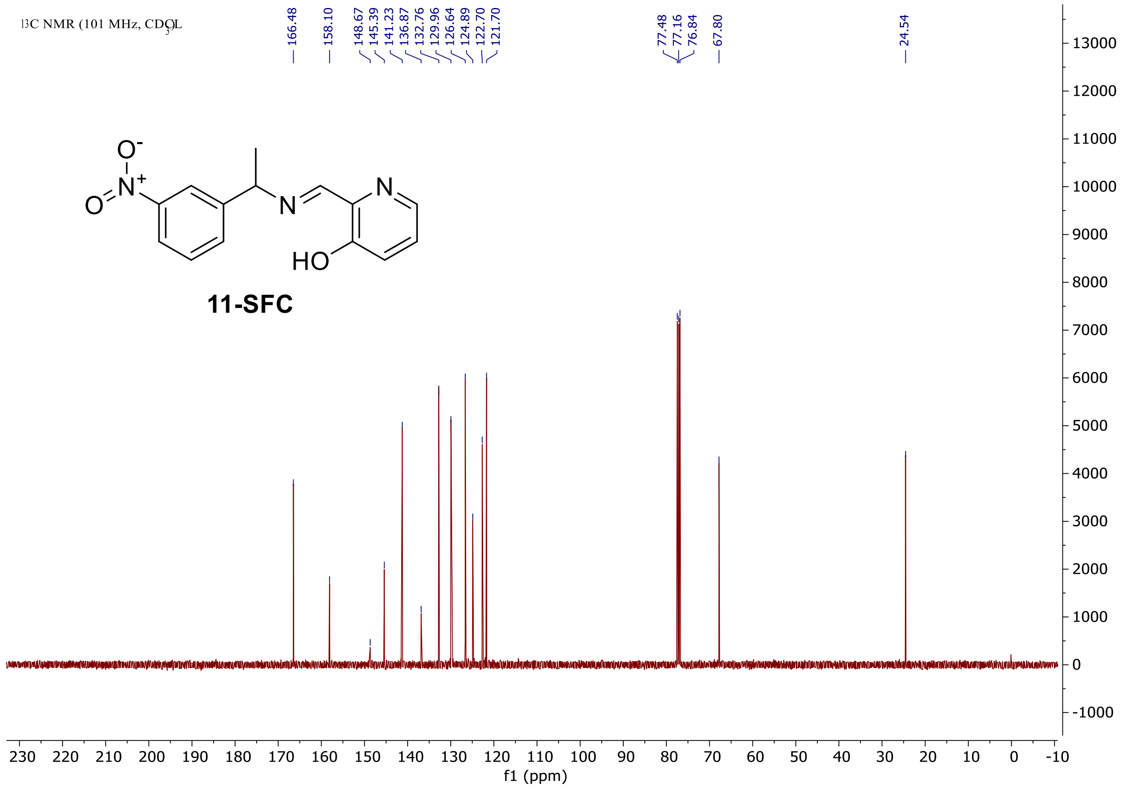

13C NMR (101 MHz, CDCl3
166.48
158.10
148.67
145.39
141.23
136.87
132.76
129.96
126.64
124.89
122.70
121.70
77.48
77.16
76.84
67.80
24.54
11-SFC
f1 (ppm)

1H NMR (400 MHz, CDCl3)

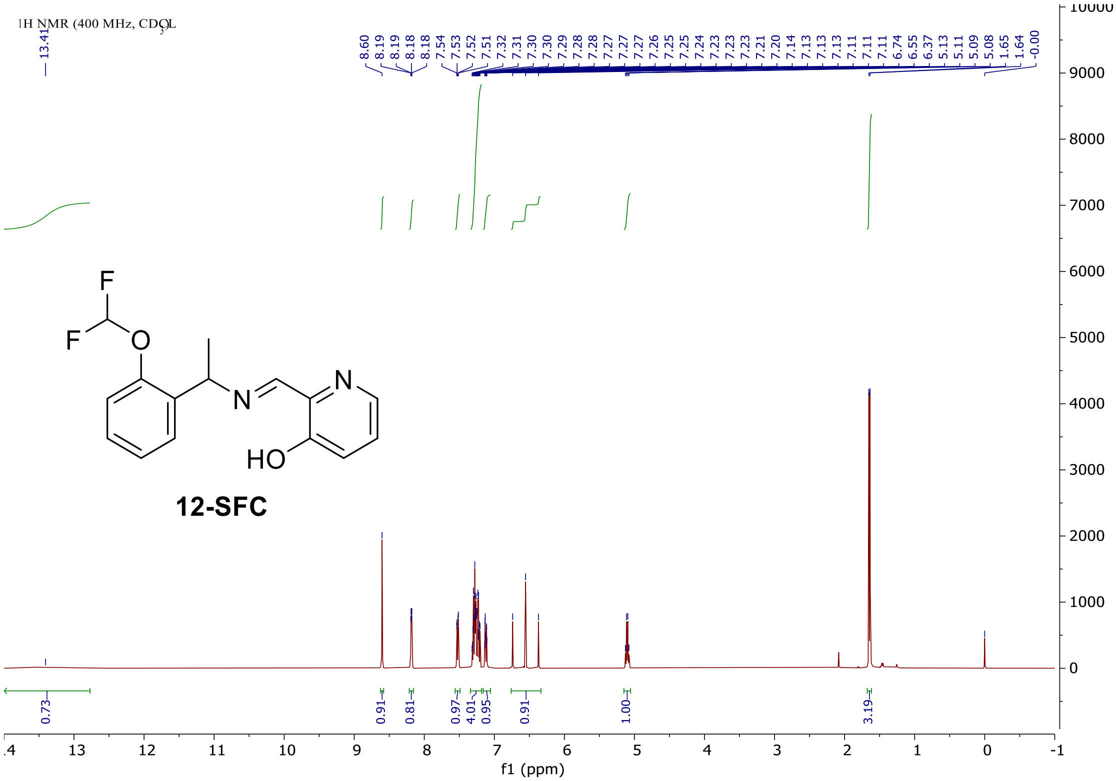

12-SFC

13C NMR (101 MHz, CDCl3)

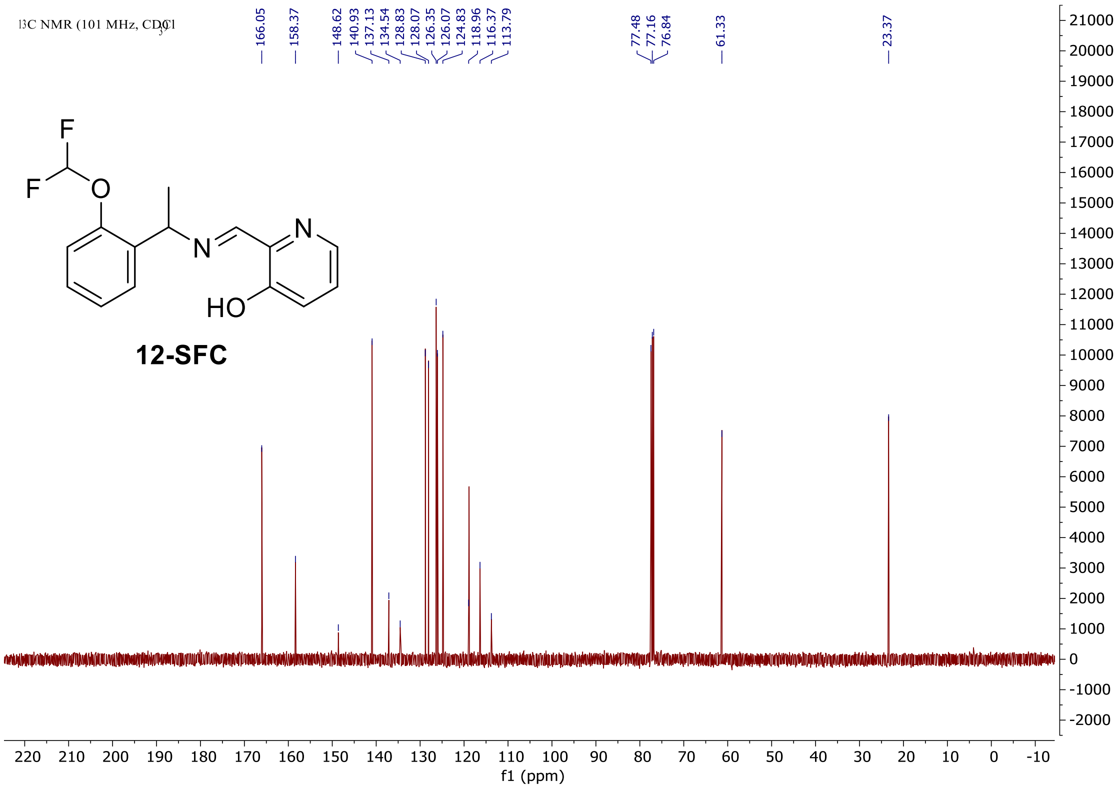

12-SFC

19F NMR (376 MHz, $CDCl_3$)

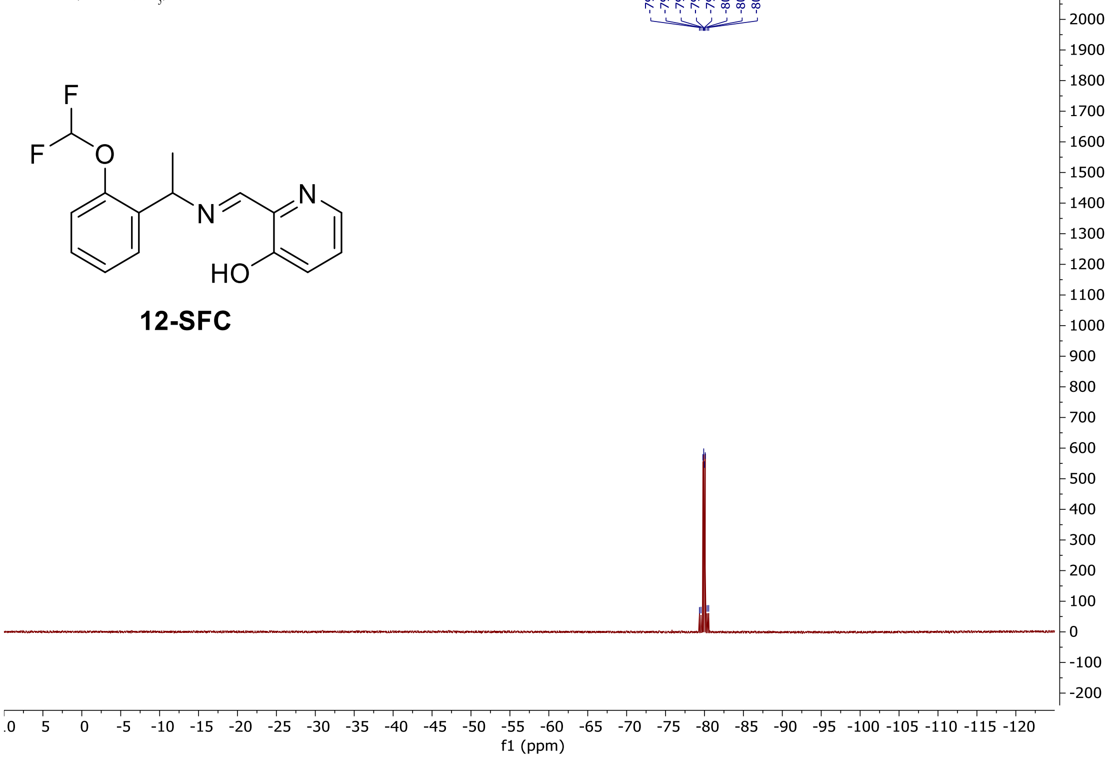

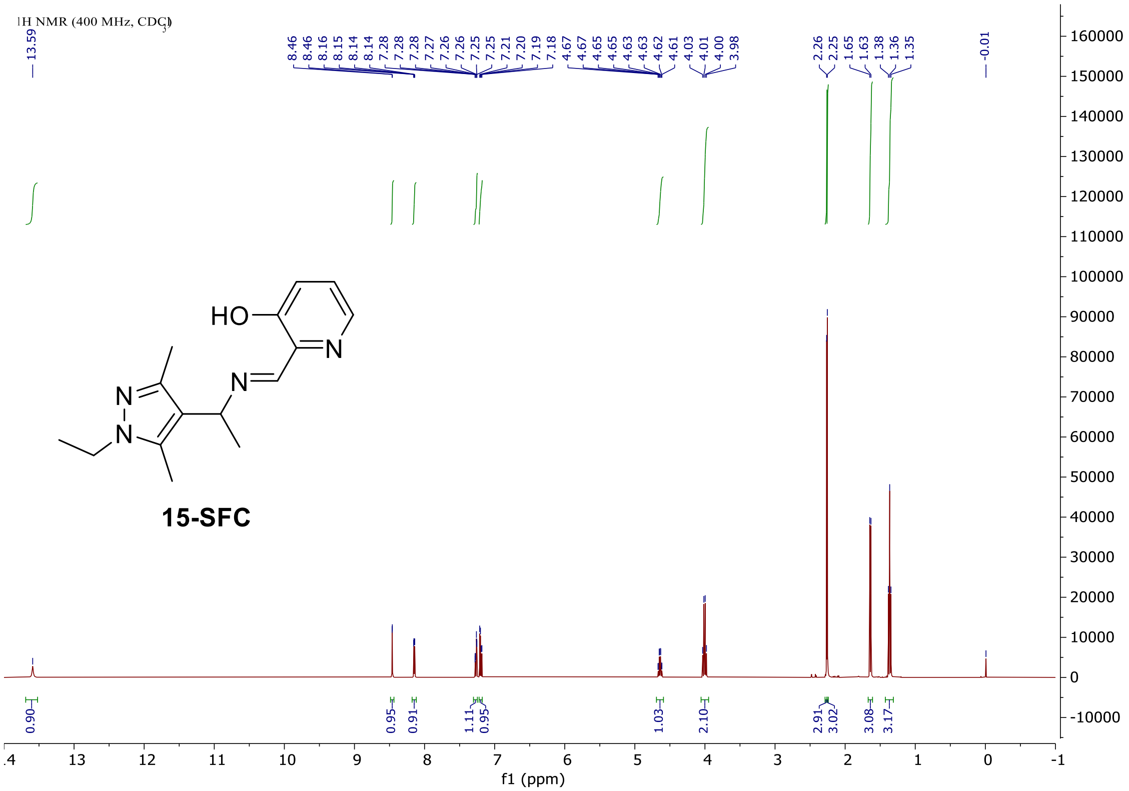
1H NMR (400 MHz, CDCl3)
15-SFC
f1 (ppm)

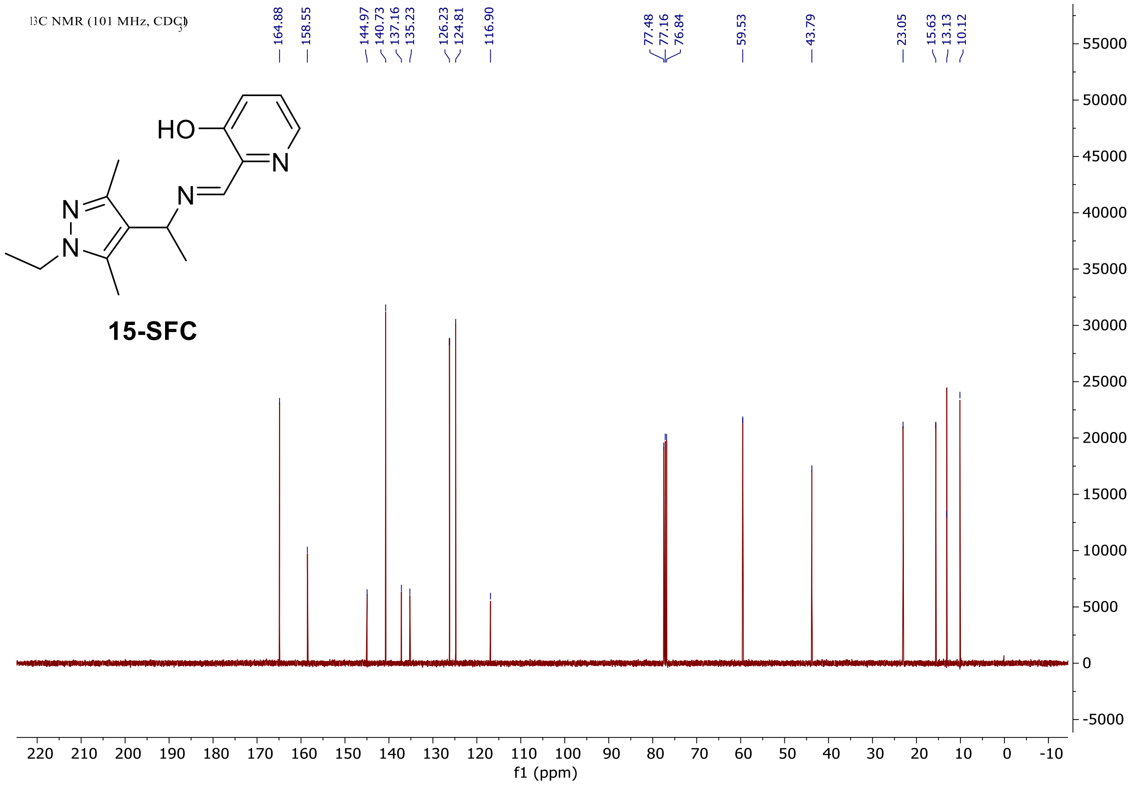
13C NMR (101 MHz, CDCl3)
15-SFC
f1 (ppm)

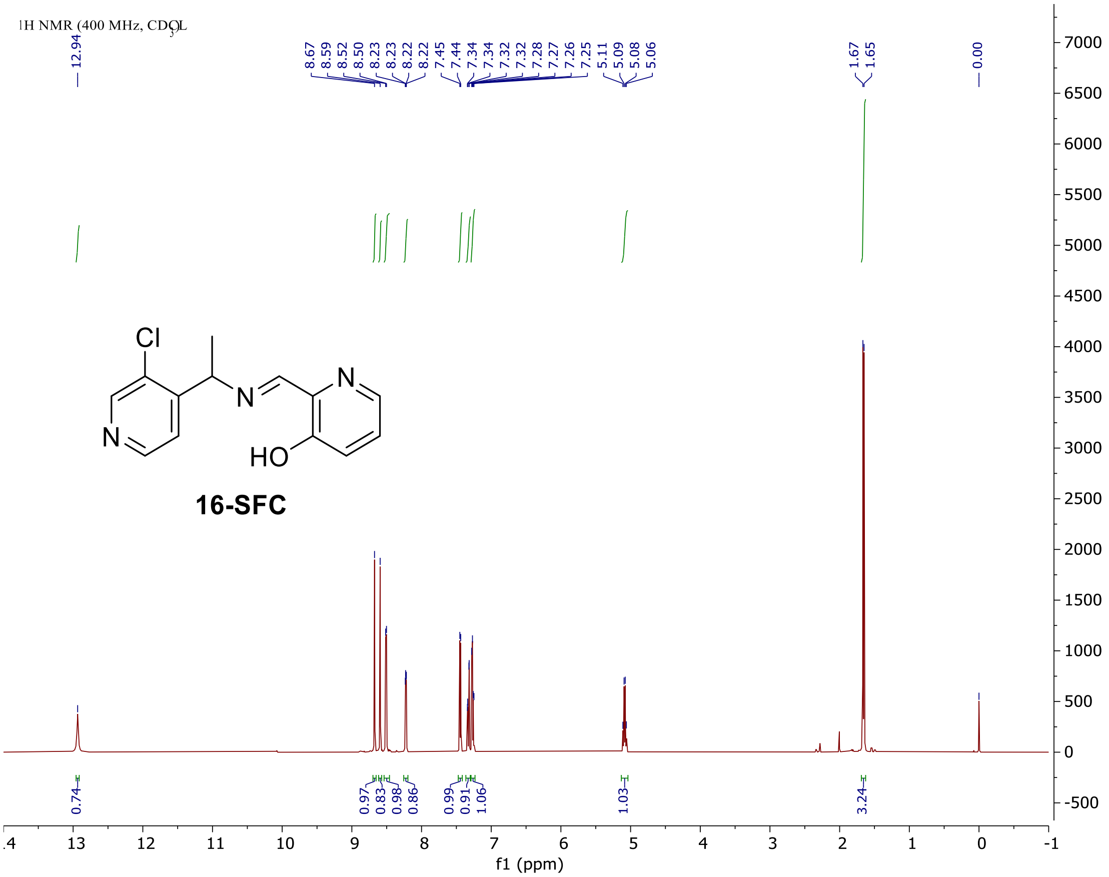
1H NMR (400 MHz, CDCl3)
12.94
8.67 8.59 8.52 8.50 8.23 8.23 8.22 8.22 7.45 7.44 7.34 7.34 7.32 7.32 7.28 7.27 7.26 7.25 5.11 5.09 5.08 5.06
1.67 1.65
0.00
Cl
N
N
N
HO
16-SFC
0.74
0.97 0.83 0.98 0.86
0.99 0.91 1.06
1.03
3.24
f1 (ppm)

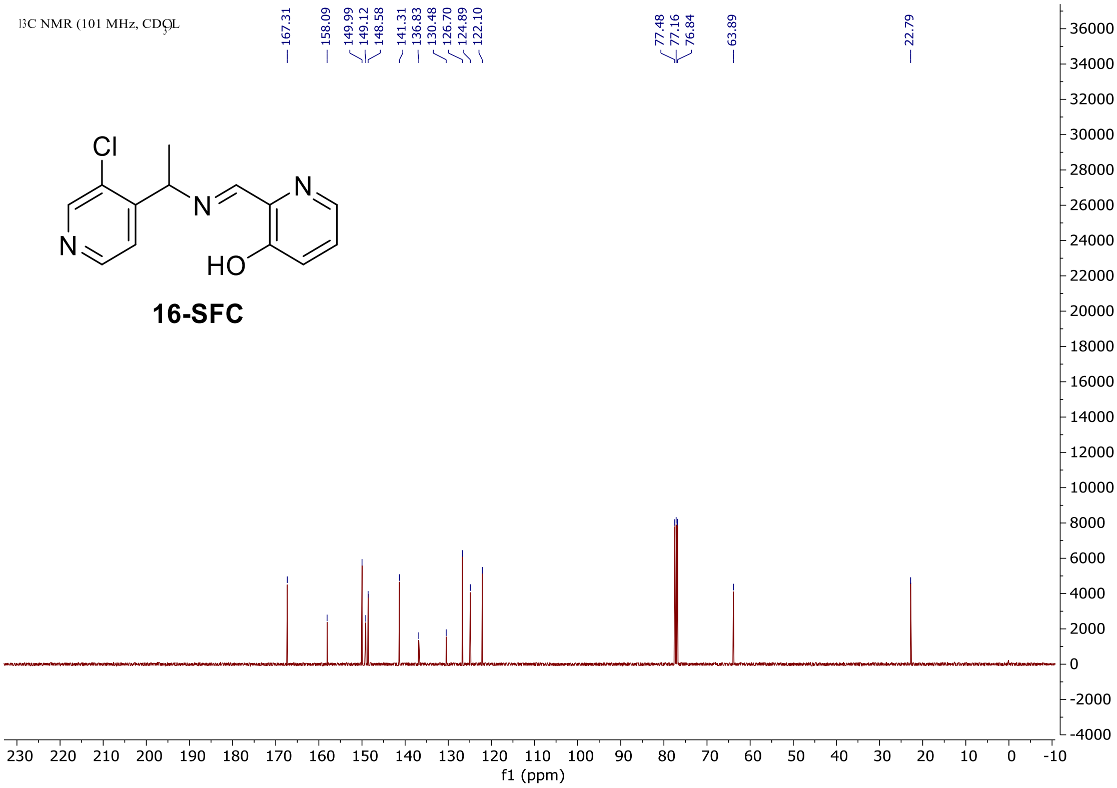
13C NMR (101 MHz, CDCl3)
167.31
158.09
149.99 149.12 148.58
141.31 136.83 130.48 126.70 124.89 122.10
77.48 77.16 76.84
63.89
22.79
Cl
N
N
N
HO
16-SFC
f1 (ppm)

1H NMR (500 MHz, CDCl3)

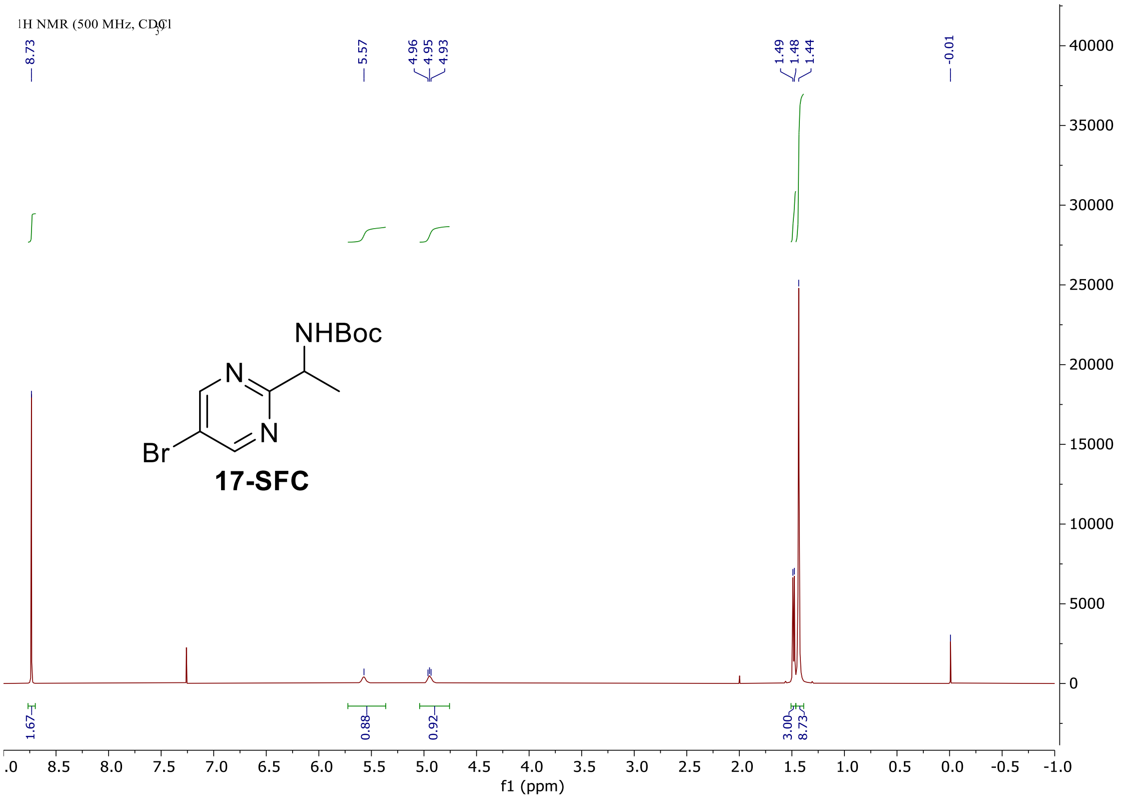

17-SFC

13C NMR (101 MHz, CDCl3)

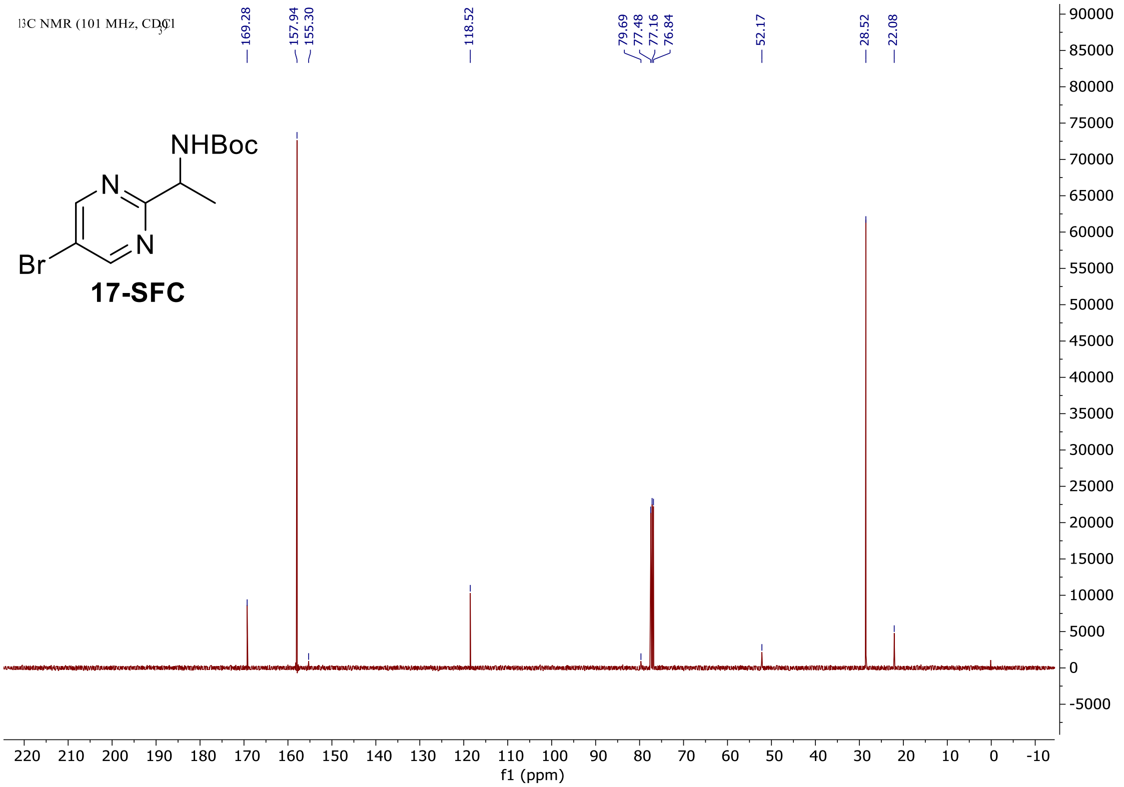

17-SFC

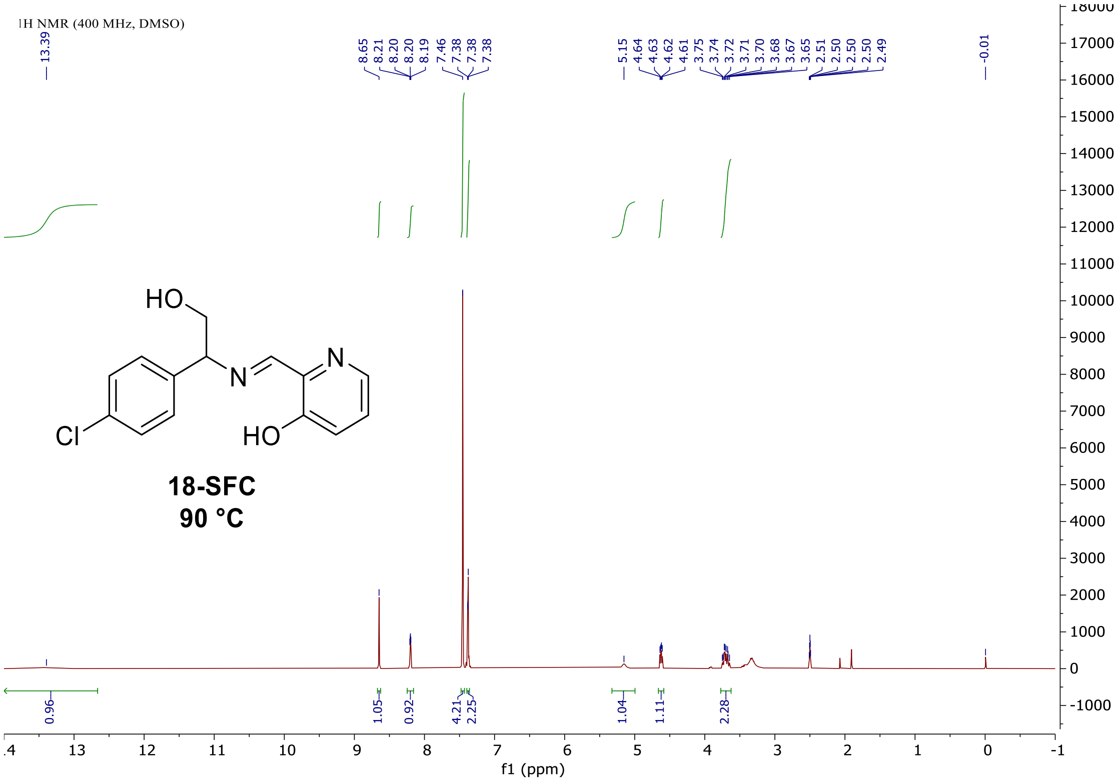
1H NMR (400 MHz, DMSO)
13.39
8.65
8.21
8.20
8.20
8.19
7.46
7.38
7.38
7.38
5.15
4.64
4.63
4.62
4.61
3.75
3.74
3.72
3.71
3.70
3.68
3.67
3.65
2.51
2.50
2.50
2.50
2.49
-0.01
HO
N
N
Cl
HO
18-SFC
90 °C
0.96
1.05
0.92
4.21
2.25
1.04
1.11
2.28
f1 (ppm)

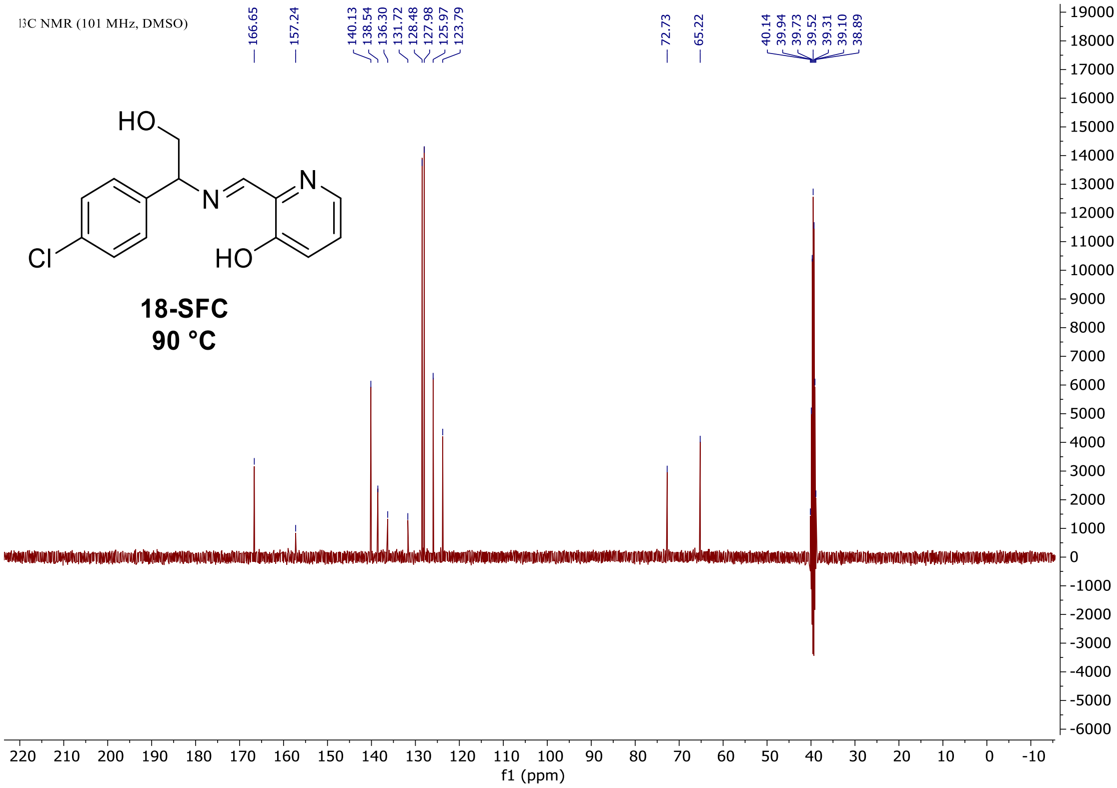
13C NMR (101 MHz, DMSO)
166.65
157.24
140.13
138.54
136.30
131.72
128.48
127.98
125.97
123.79
72.73
65.22
40.14
39.94
39.73
39.52
39.31
39.10
38.89
HO
N
N
Cl
HO
18-SFC
90 °C
f1 (ppm)

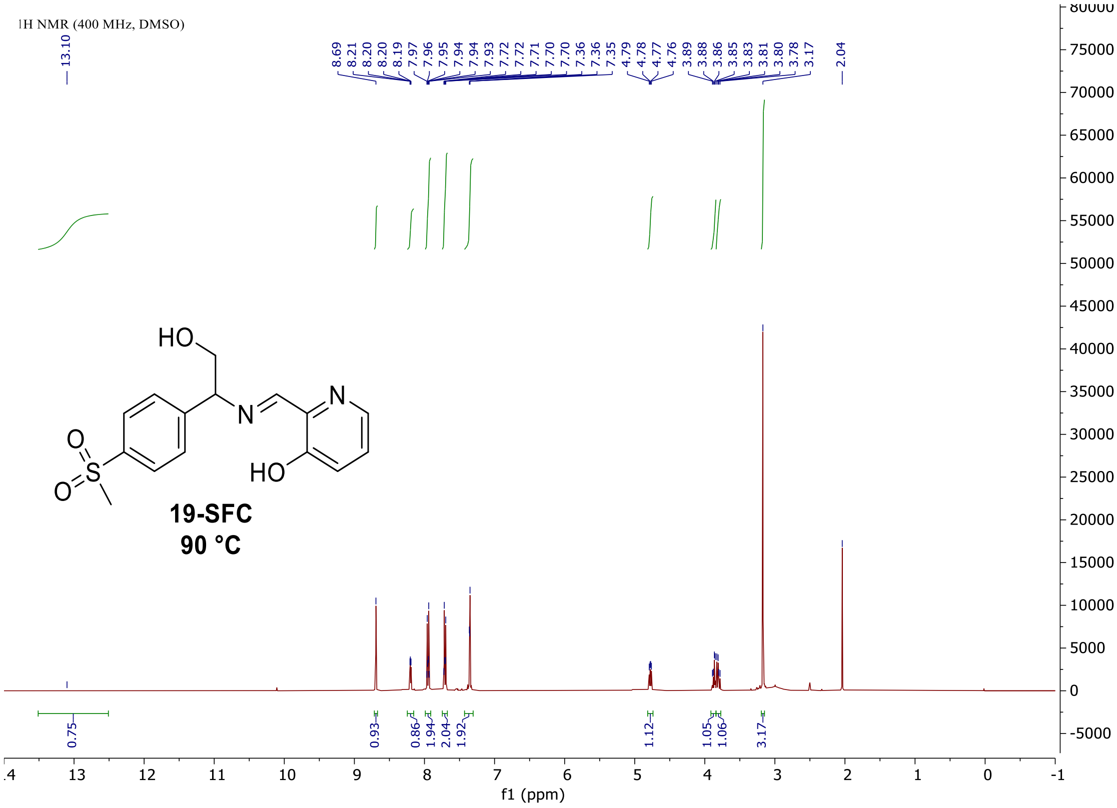
1H NMR (400 MHz, DMSO)
19-SFC
90 °C
f1 (ppm)

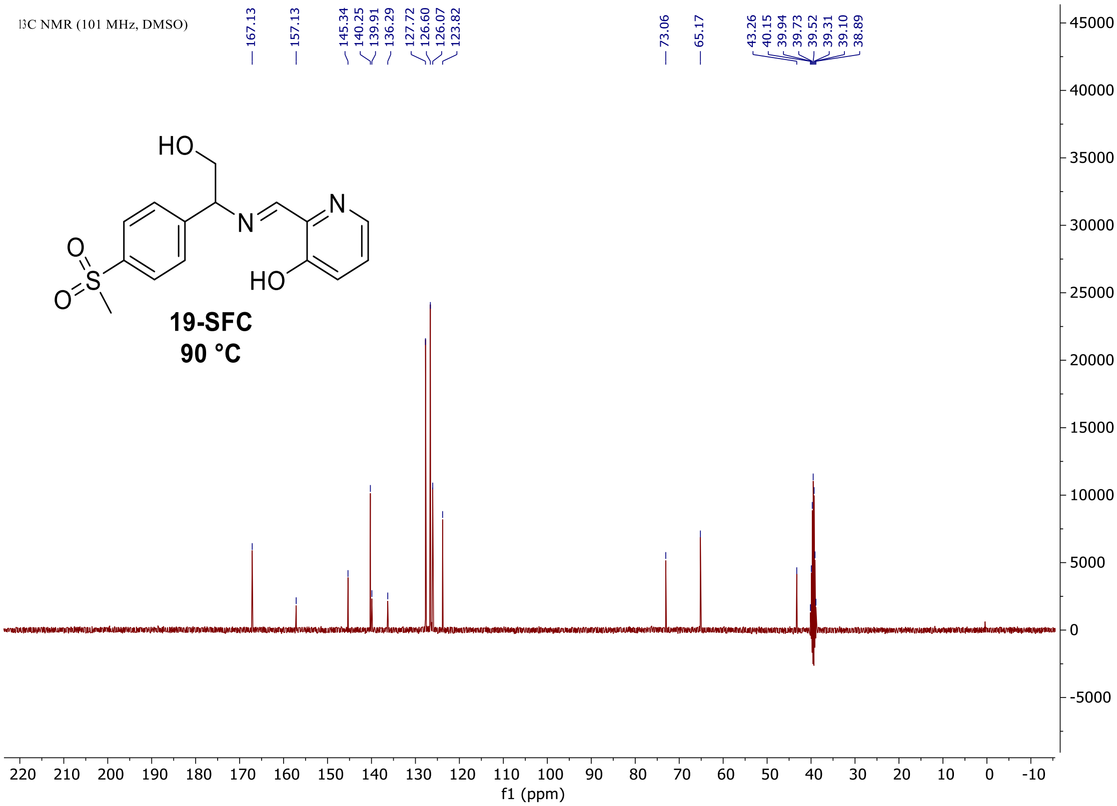
13C NMR (101 MHz, DMSO)
19-SFC
90 °C
f1 (ppm)

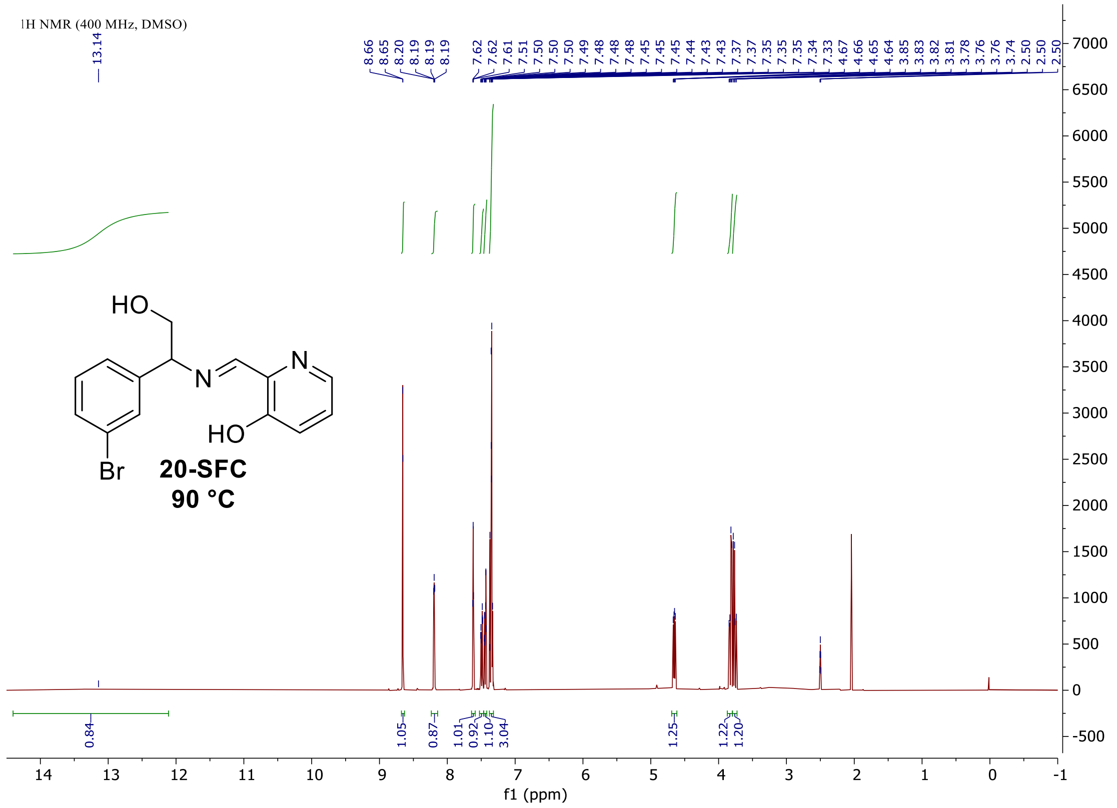
1H NMR (400 MHz, DMSO)
20-SFC
90 °C
f1 (ppm)

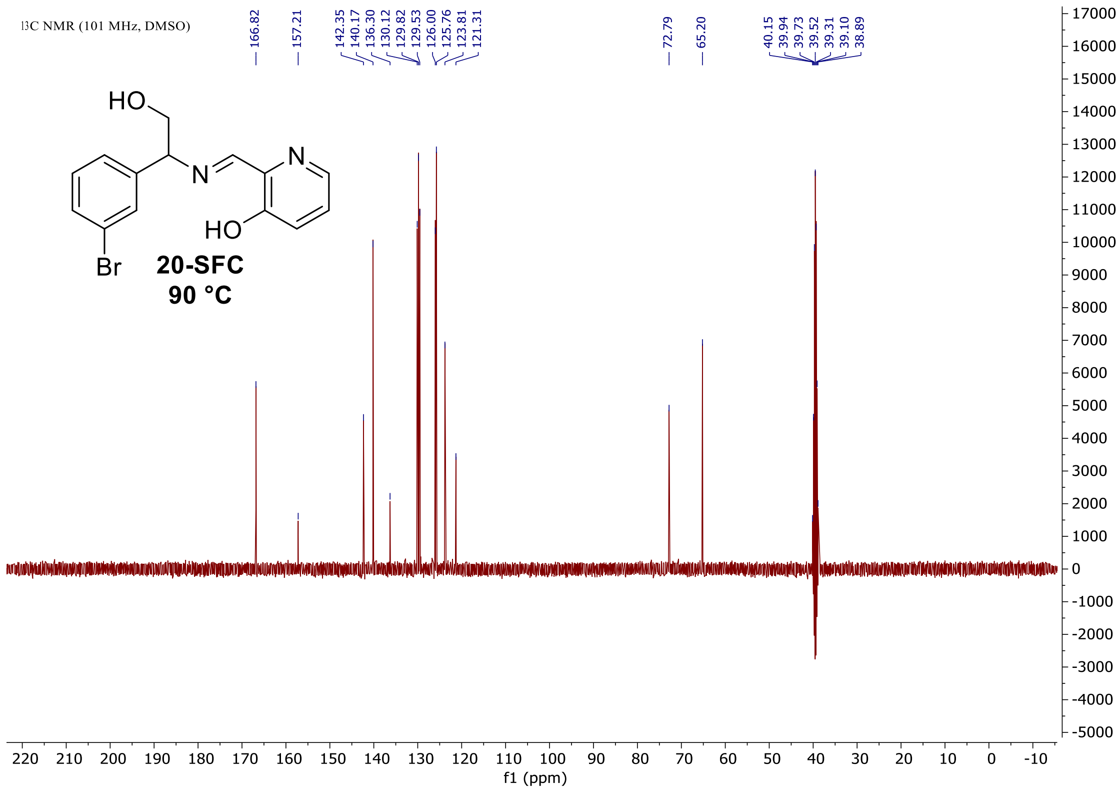
13C NMR (101 MHz, DMSO)
20-SFC
90 °C
f1 (ppm)

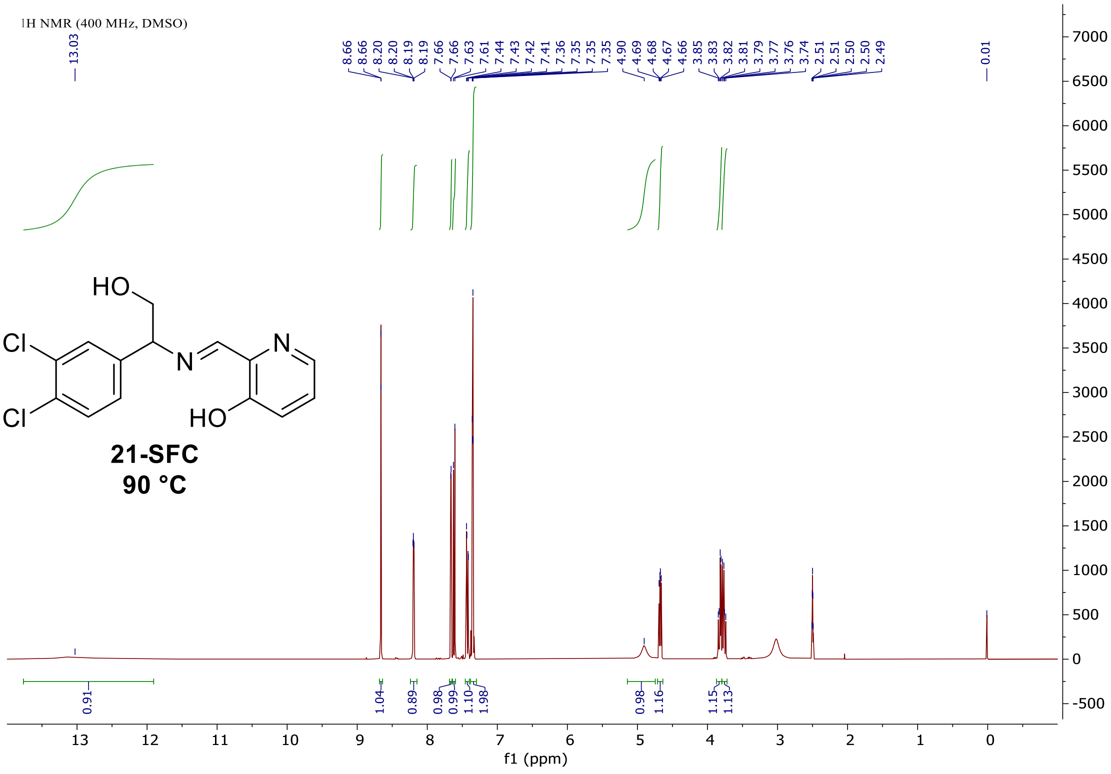
1H NMR (400 MHz, DMSO)
21-SFC
90 °C
f1 (ppm)

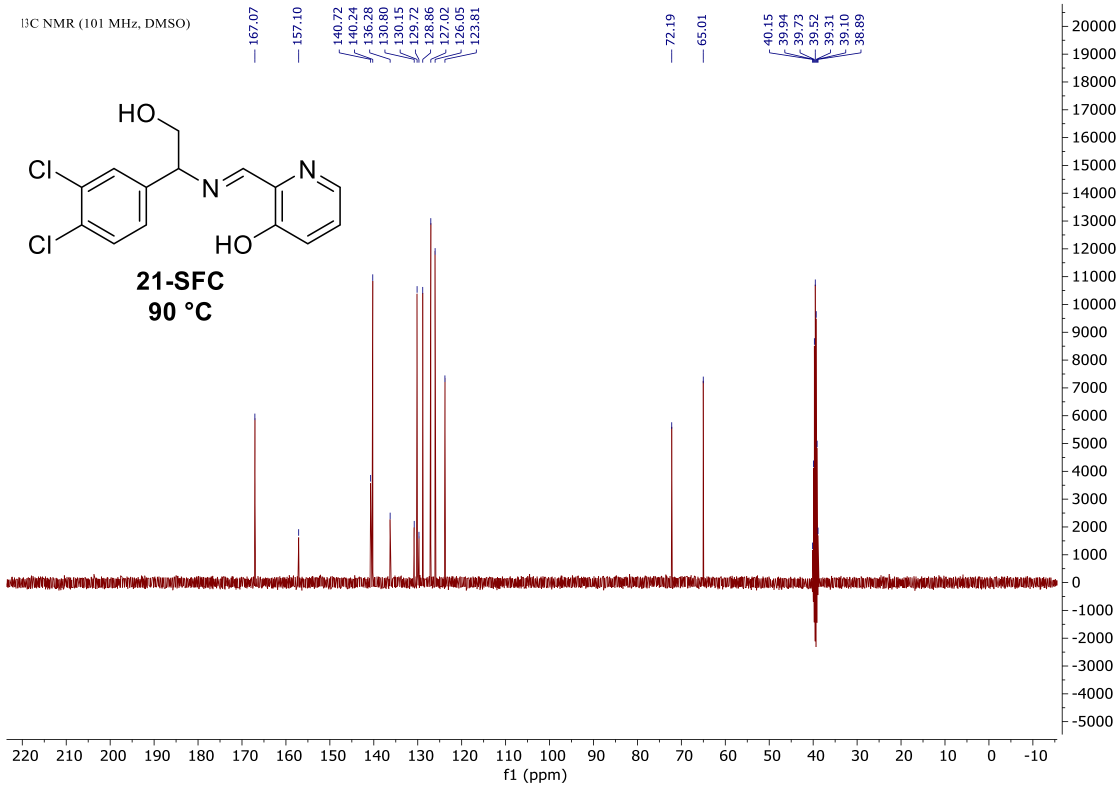
13C NMR (101 MHz, DMSO)
21-SFC
90 °C
f1 (ppm)

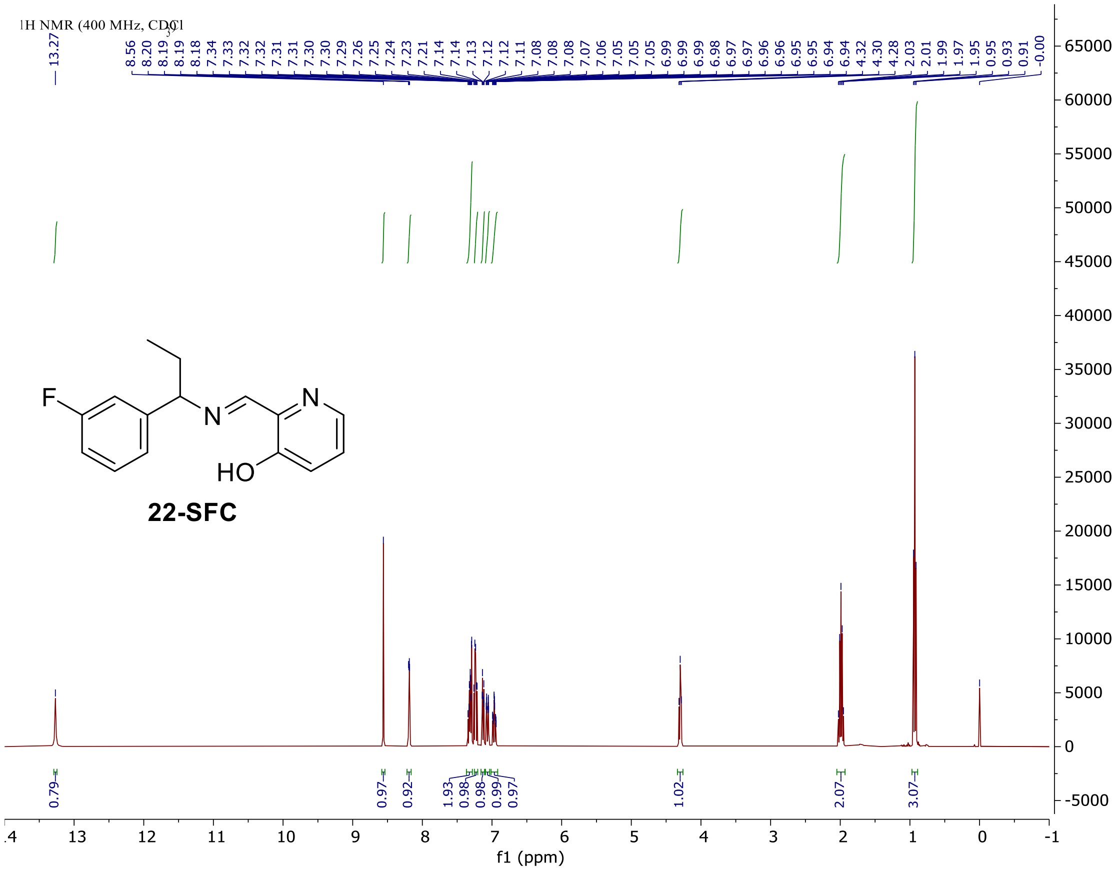
1H NMR (400 MHz, CDCl3
13.27
8.56 8.20 8.19 8.19 8.18 7.34 7.33 7.32 7.32 7.31 7.31 7.30 7.30 7.29 7.26 7.25 7.24 7.23 7.21 7.14 7.14 7.13 7.12 7.12 7.11 7.08 7.08 7.07 7.06 7.05 7.05 7.05 6.99 6.99 6.99 6.98 6.97 6.97 6.96 6.96 6.95 6.95 6.94 4.32 4.30 4.28 2.03 2.01 1.99 1.97 1.95 0.95 0.93 0.91 -0.00
F
N
N
HO
22-SFC
0.79 0.97 0.92 1.93 0.98 0.98 0.99 0.97 1.02 2.07 3.07
f1 (ppm)

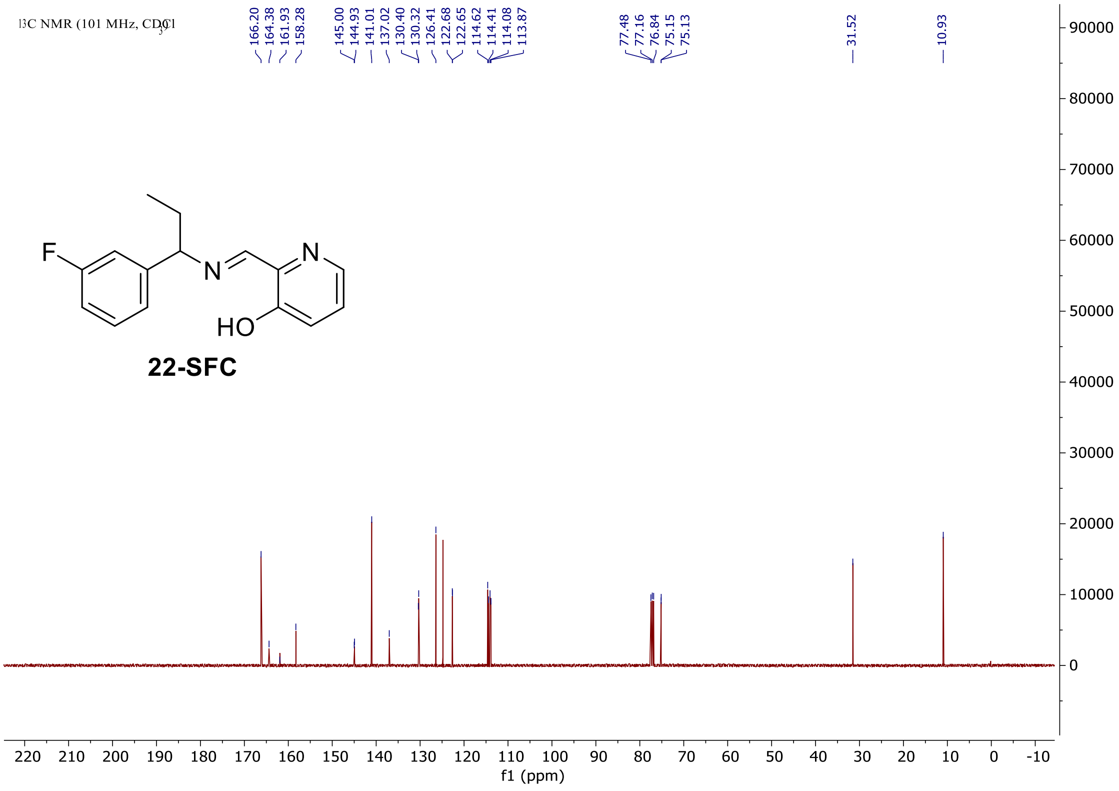
13C NMR (101 MHz, CDCl3
166.20 164.38 161.93 158.28 145.00 144.93 141.01 137.02 130.40 130.32 126.41 122.68 122.65 114.62 114.41 114.08 113.87 77.48 77.16 76.84 75.15 75.13 31.52 10.93
F
N
N
HO
22-SFC
f1 (ppm)

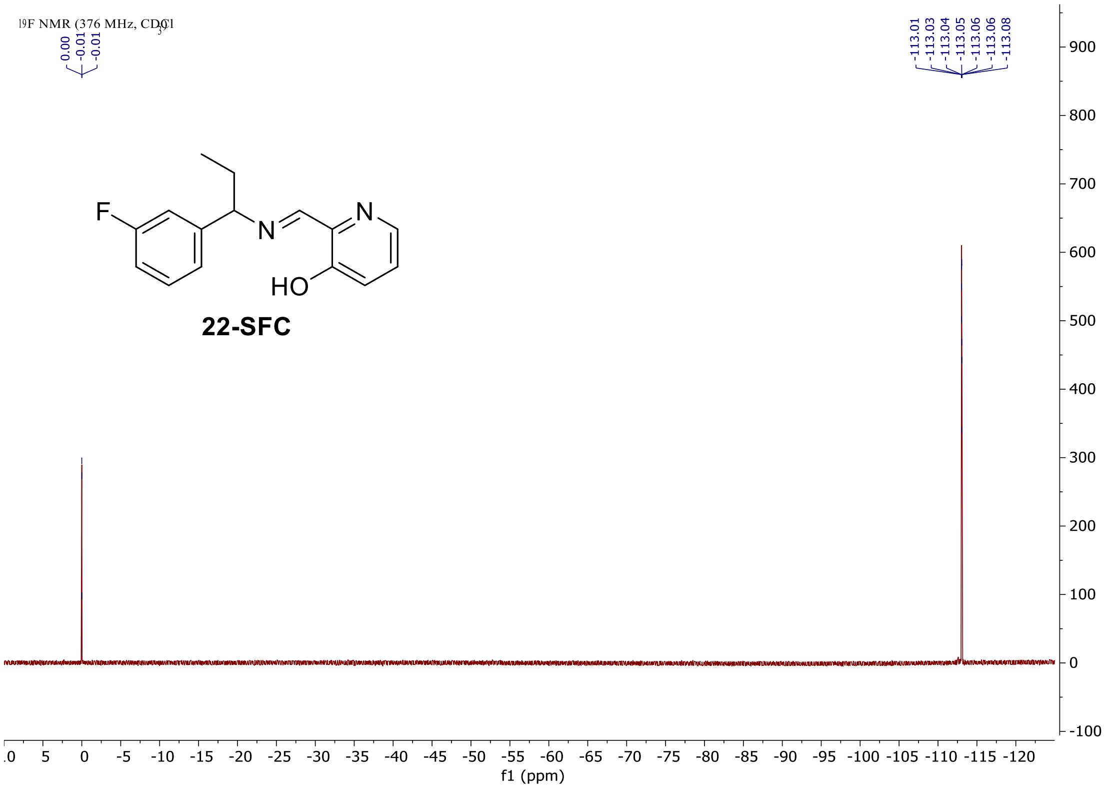
19F NMR (376 MHz, CDCl3
0.00
-0.01
-0.01
-113.01
-113.03
-113.04
-113.05
-113.06
-113.06
-113.08
F
N
N
HO
22-SFC
f1 (ppm)

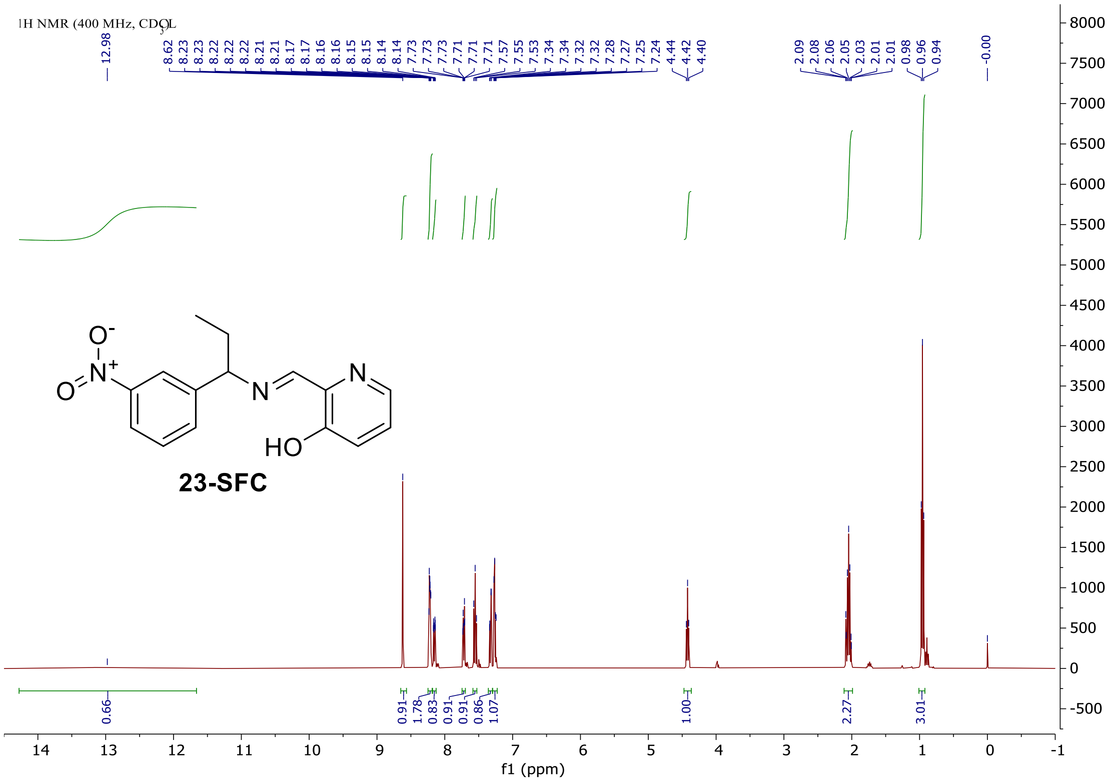
1H NMR (400 MHz, CDCl3)
12.98
8.62 8.23 8.23 8.22 8.22 8.22 8.21 8.21 8.17 8.17 8.16 8.16 8.15 8.15 8.14 8.14 7.73 7.73 7.73 7.71 7.71 7.71 7.57 7.55 7.53 7.34 7.34 7.32 7.32 7.28 7.27 7.25 7.24 4.44 4.42 4.40
2.09 2.08 2.06 2.05 2.03 2.01 2.01 0.98 0.96 0.94
-0.00
0.66
0.91 1.78 0.83 0.91 0.91 0.86 1.07
1.00
2.27
3.01
23-SFC
f1 (ppm)

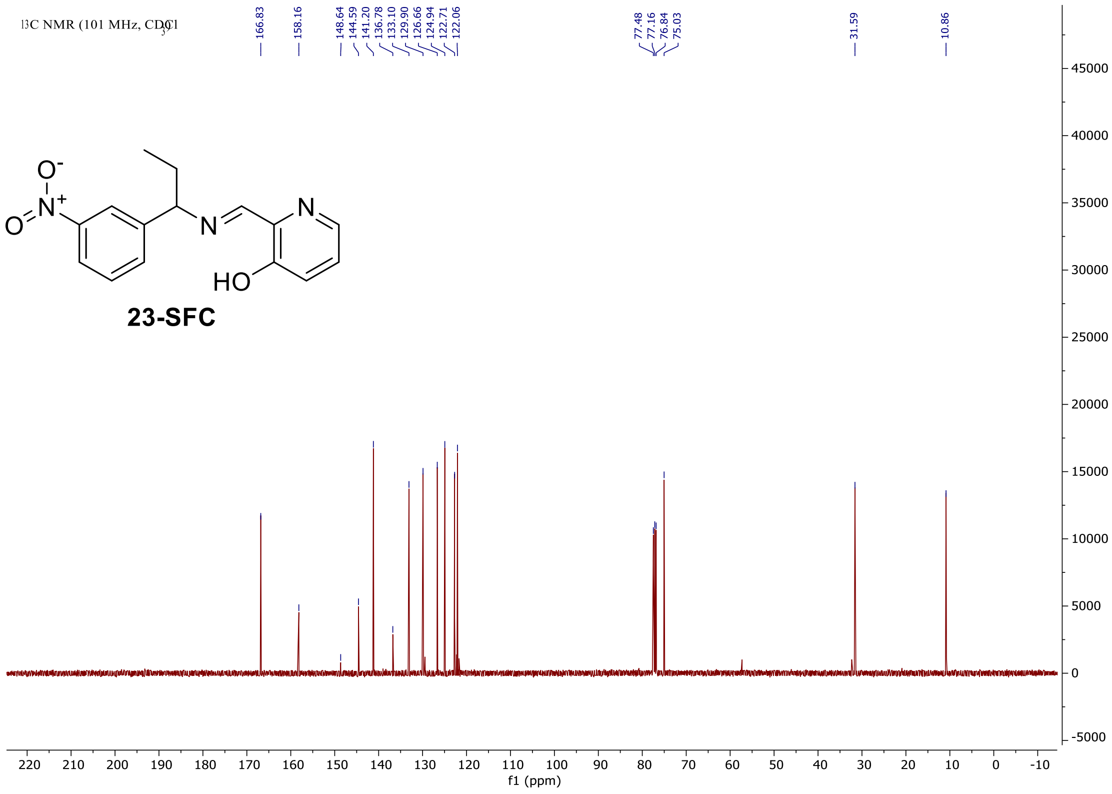
13C NMR (101 MHz, CDCl3)
166.83
158.16
148.64 144.59 141.20 136.78 133.10 129.90 126.66 124.94 122.71 122.06
77.48 77.16 76.84 75.03
31.59
10.86
23-SFC
f1 (ppm)

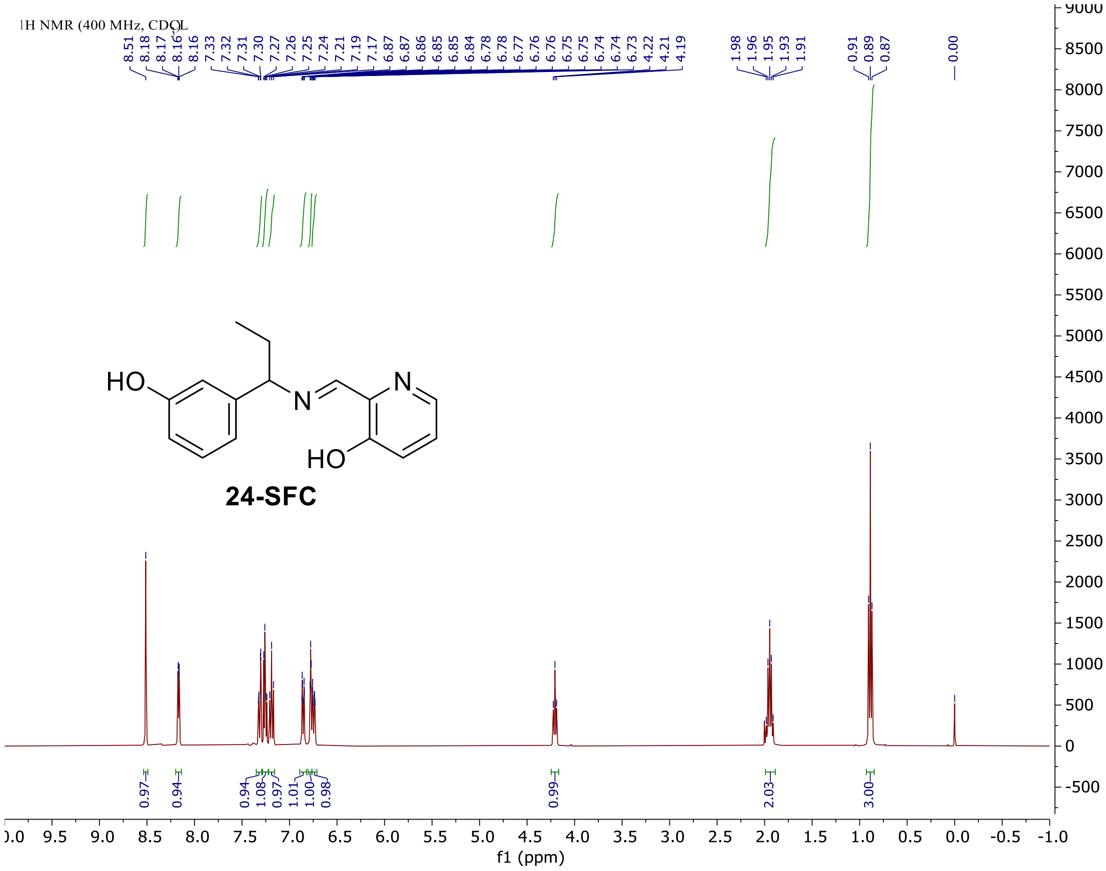
1H NMR (400 MHz, CDCl3)
HO
N
N
HO
24-SFC
f1 (ppm)

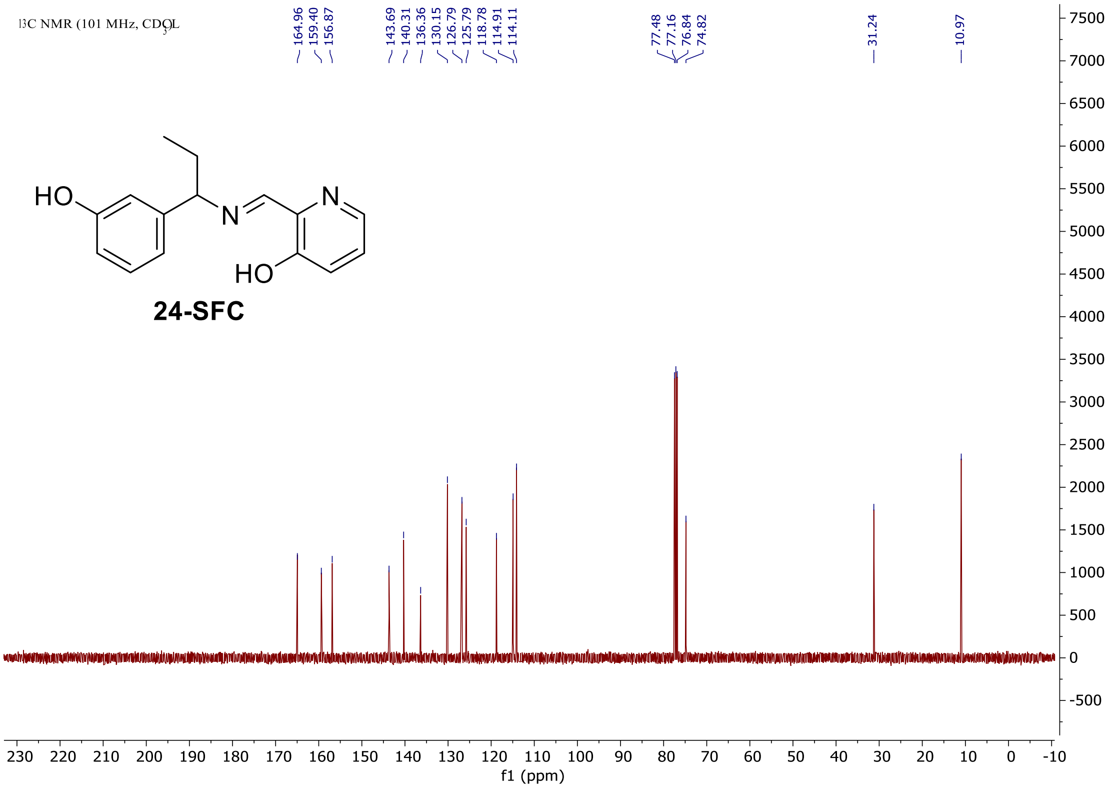
13C NMR (101 MHz, CDCl3)
164.96
159.40
156.87
143.69
140.31
136.36
130.15
126.79
125.79
118.78
114.91
114.11
77.48
77.16
76.84
74.82
31.24
10.97
HO
N
N
HO
24-SFC
f1 (ppm)

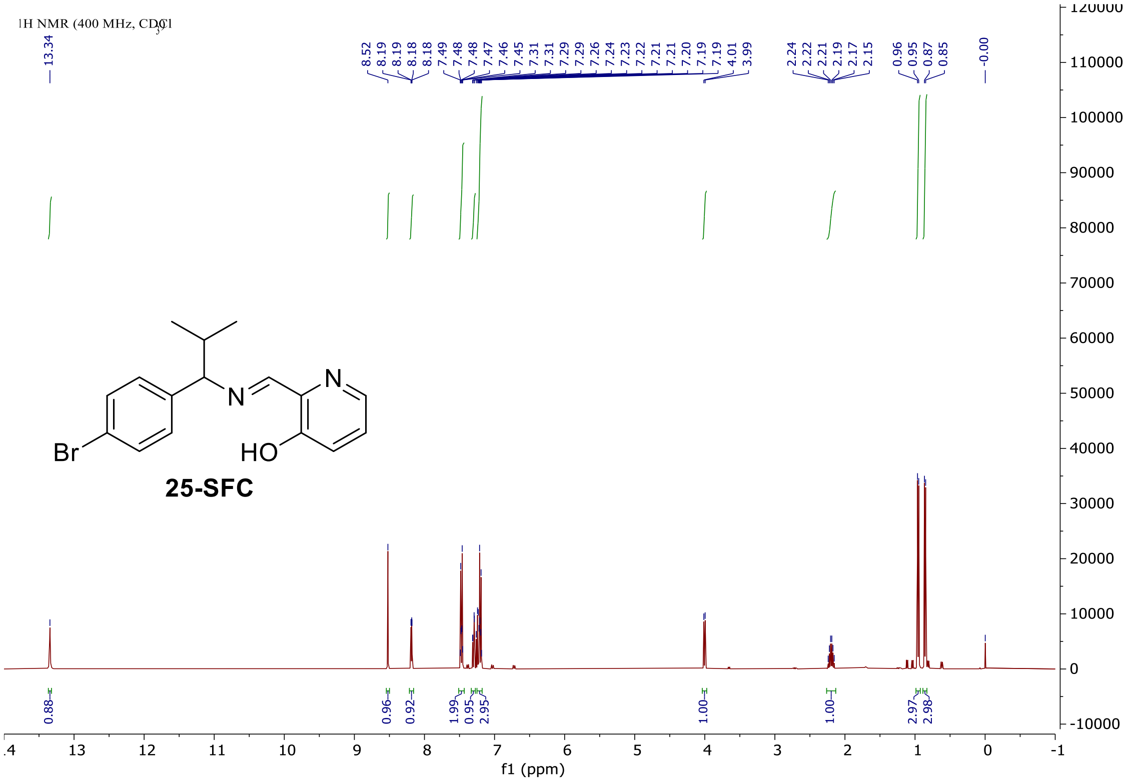
1H NMR (400 MHz, CDCl3)
13.34
8.52 8.19 8.19 8.18 8.18 7.49 7.48 7.48 7.47 7.46 7.45 7.31 7.31 7.29 7.29 7.26 7.24 7.24 7.23 7.22 7.21 7.21 7.20 7.19 7.19 4.01 3.99
2.24 2.22 2.21 2.19 2.17 2.15
0.96 0.95 0.87 0.85
-0.00
Br
N
N
HO
25-SFC
0.88
0.96 0.92 1.99 0.95 2.95
1.00
1.00
2.97 2.98
f1 (ppm)

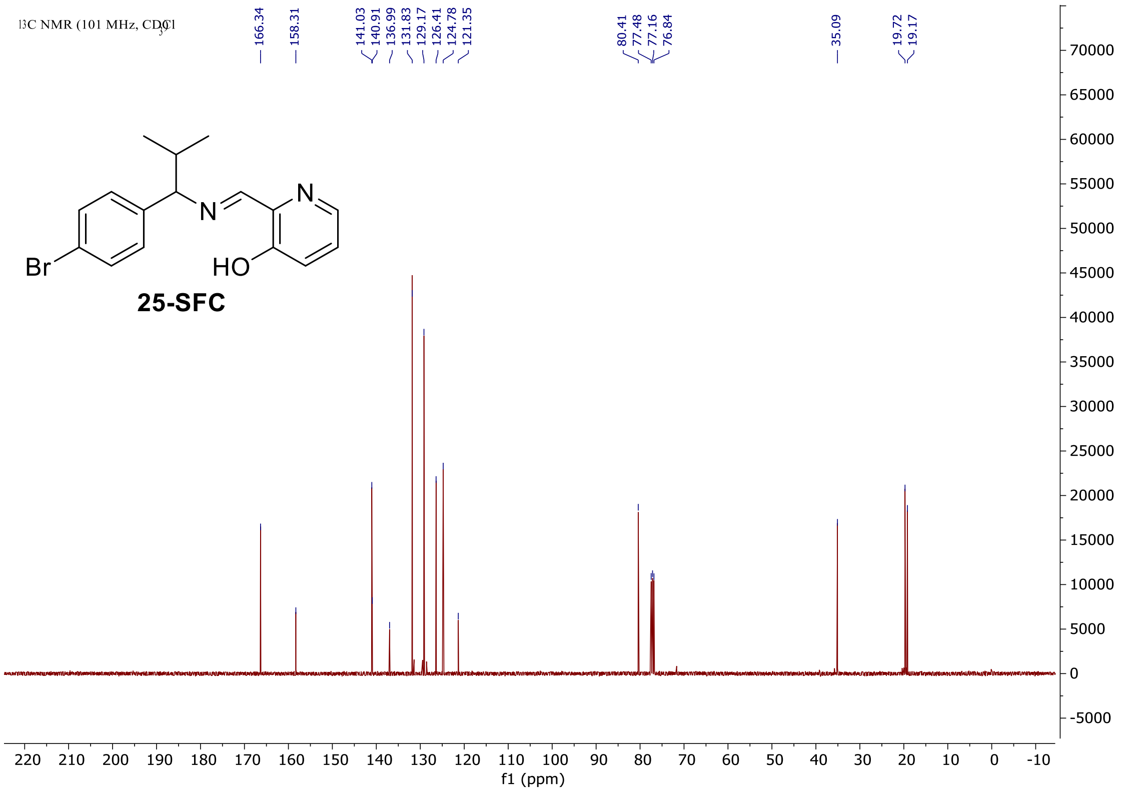
13C NMR (101 MHz, CDCl3)
166.34
158.31
141.03 140.91 136.99 131.83 129.17 126.41 124.78 121.35
80.41 77.48 77.16 76.84
35.09
19.72 19.17
Br
N
N
HO
25-SFC
f1 (ppm)

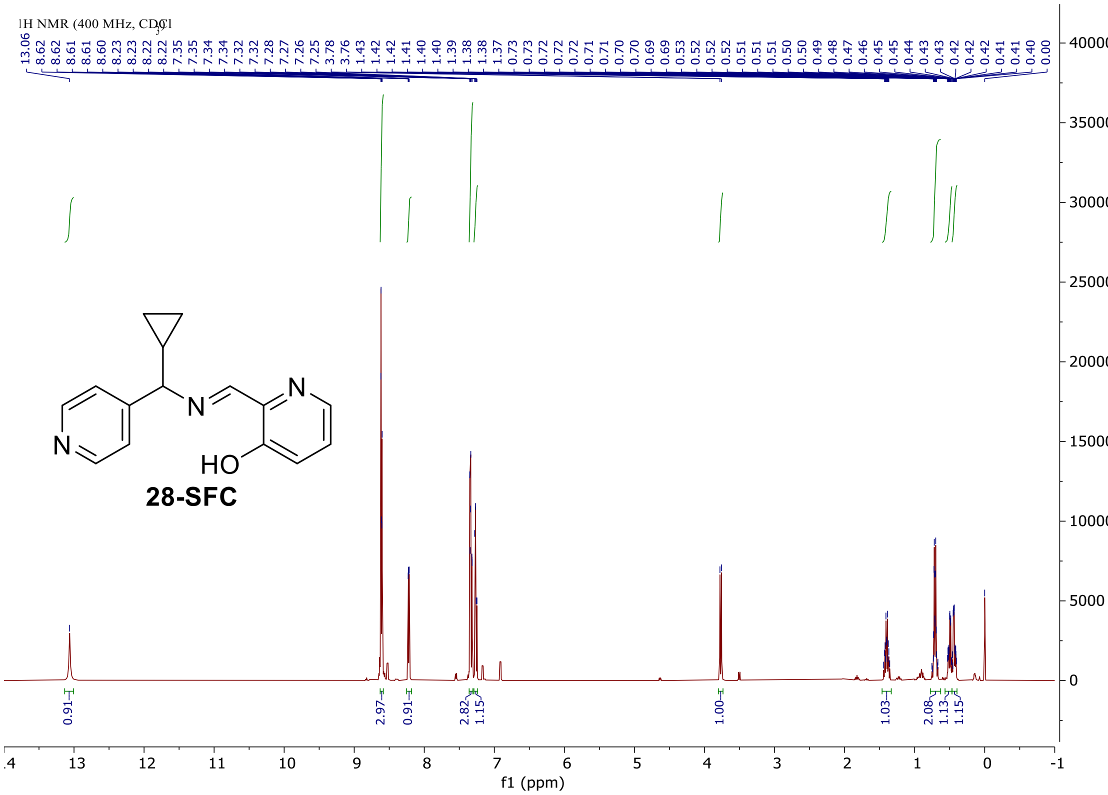
1H NMR (400 MHz, CDCl3
13.06 8.62 8.62 8.61 8.61 8.60 8.23 8.23 8.22 8.22 7.35 7.35 7.34 7.32 7.32 7.28 7.27 7.26 7.25 3.78 3.76 1.43 1.42 1.42 1.41 1.40 1.39 1.38 1.38 1.37 0.73 0.73 0.72 0.72 0.71 0.71 0.70 0.70 0.69 0.69 0.53 0.52 0.52 0.51 0.51 0.51 0.50 0.50 0.49 0.48 0.47 0.46 0.45 0.45 0.44 0.43 0.43 0.42 0.42 0.42 0.41 0.40 0.00
0.91 2.97 0.91 2.82 1.15 1.00 1.03 2.08 1.13 1.15
28-SFC
f1 (ppm)

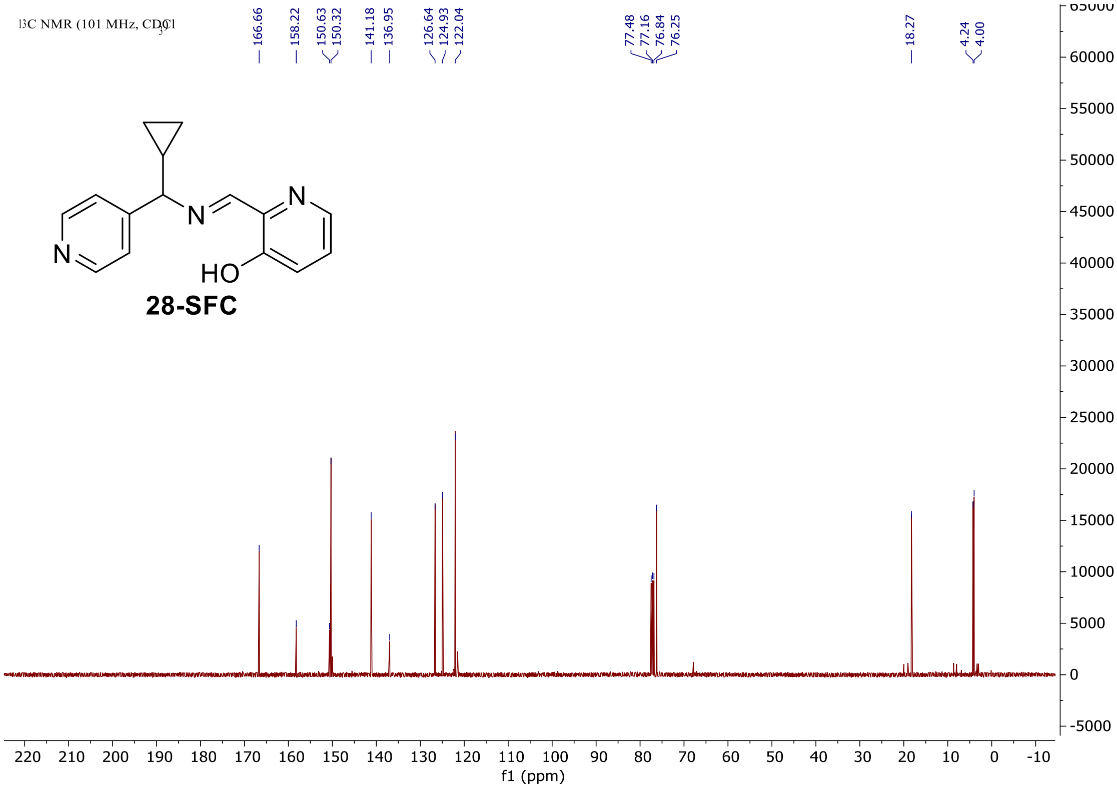
13C NMR (101 MHz, CDCl3
166.66 158.22 150.63 150.32 141.18 136.95 126.64 124.93 122.04 77.48 77.16 76.84 76.25 18.27 4.24 4.00
28-SFC
f1 (ppm)

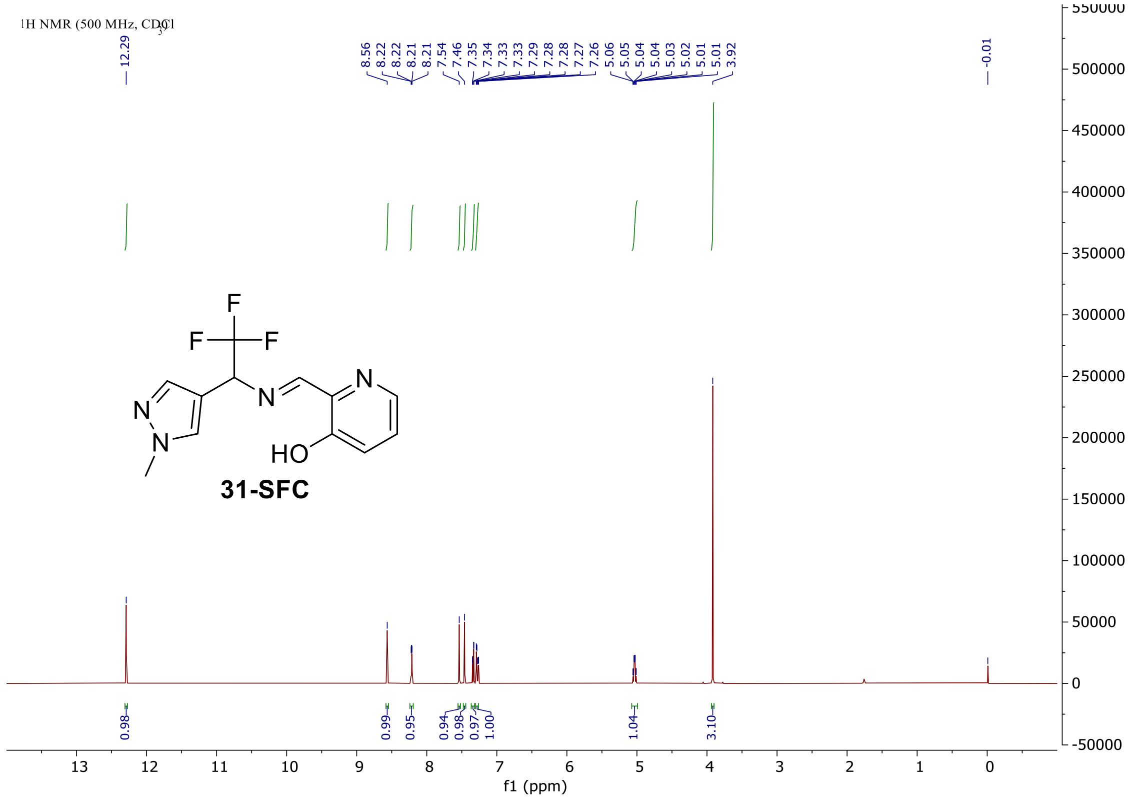
1H NMR (500 MHz, CDCl3
12.29
8.56
8.22
8.22
8.21
8.21
7.54
7.46
7.35
7.34
7.33
7.33
7.29
7.28
7.28
7.27
7.26
5.06
5.05
5.04
5.04
5.03
5.02
5.01
5.01
3.92
-0.01
F
F
F
N
N
N
N
HO
31-SFC
0.98
0.99
0.95
0.94
0.98
0.97
1.00
1.04
3.10
f1 (ppm)

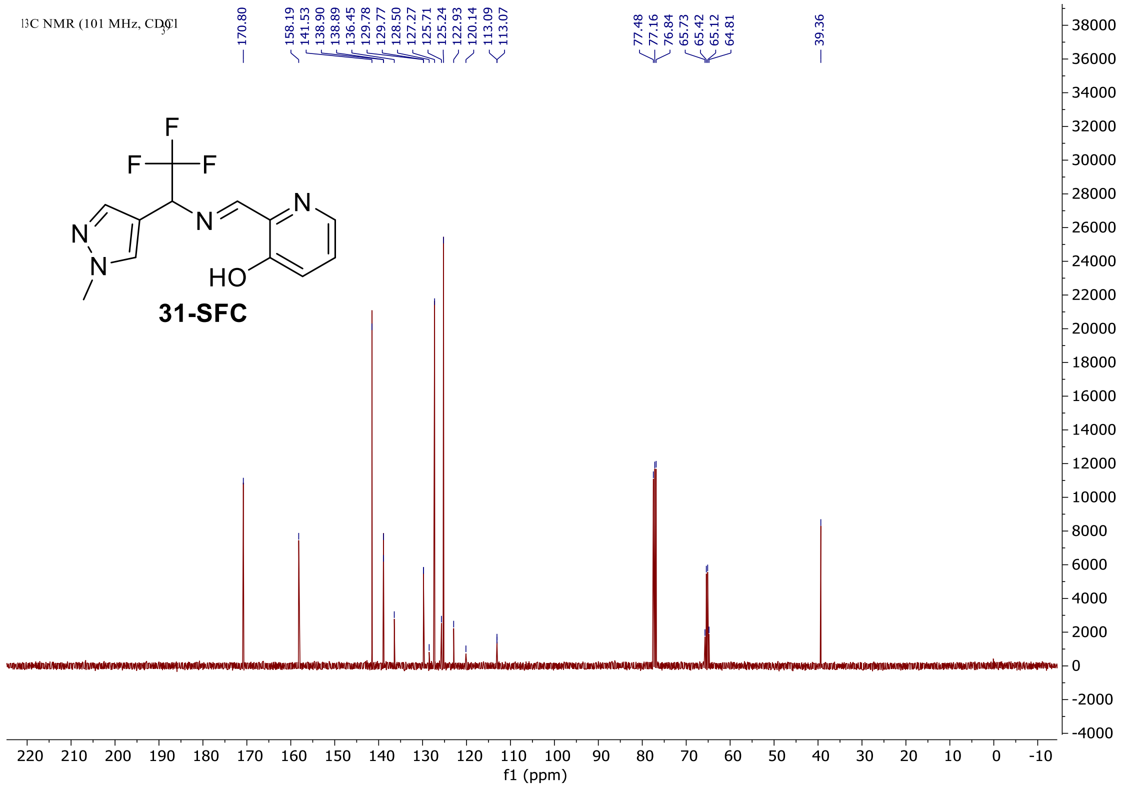
13C NMR (101 MHz, CDCl3
170.80
158.19
141.53
138.90
138.89
136.45
129.78
129.77
128.50
127.27
125.71
125.24
122.93
120.14
113.09
113.07
77.48
77.16
76.84
65.73
65.42
65.12
64.81
39.36
F
F
F
N
N
N
N
HO
31-SFC
f1 (ppm)

19F NMR (376 MHz, $CDCl_3$)

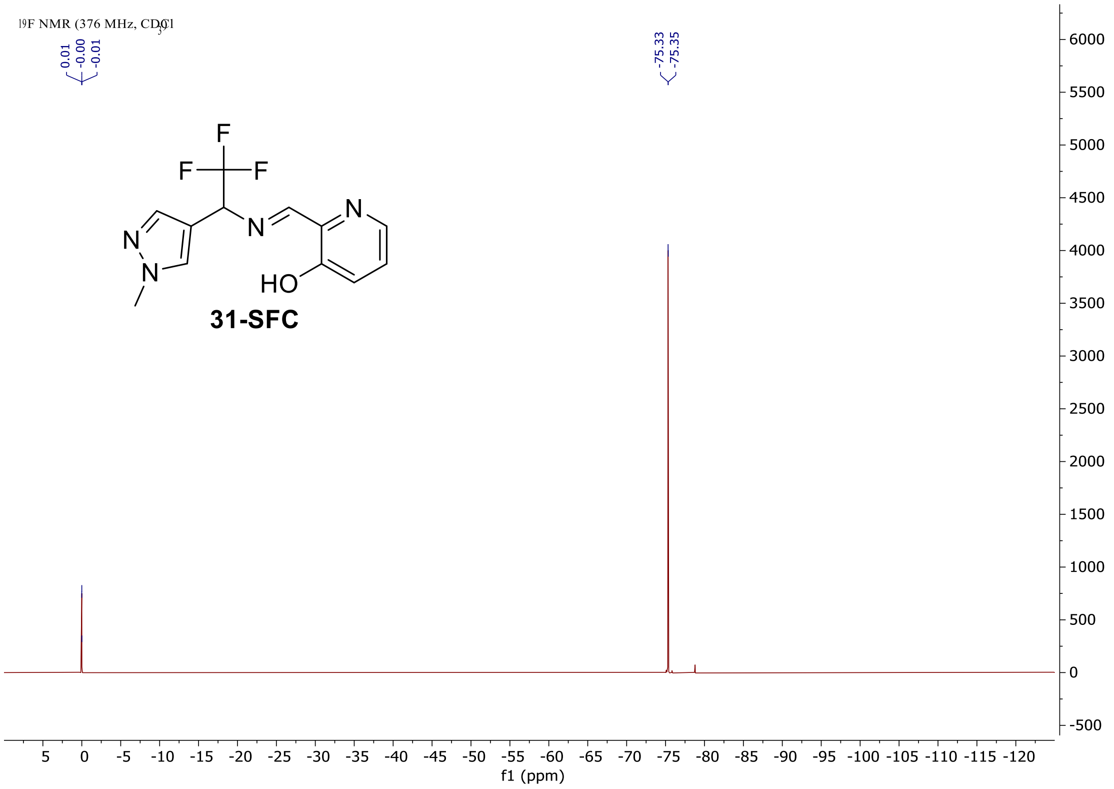

31-SFC

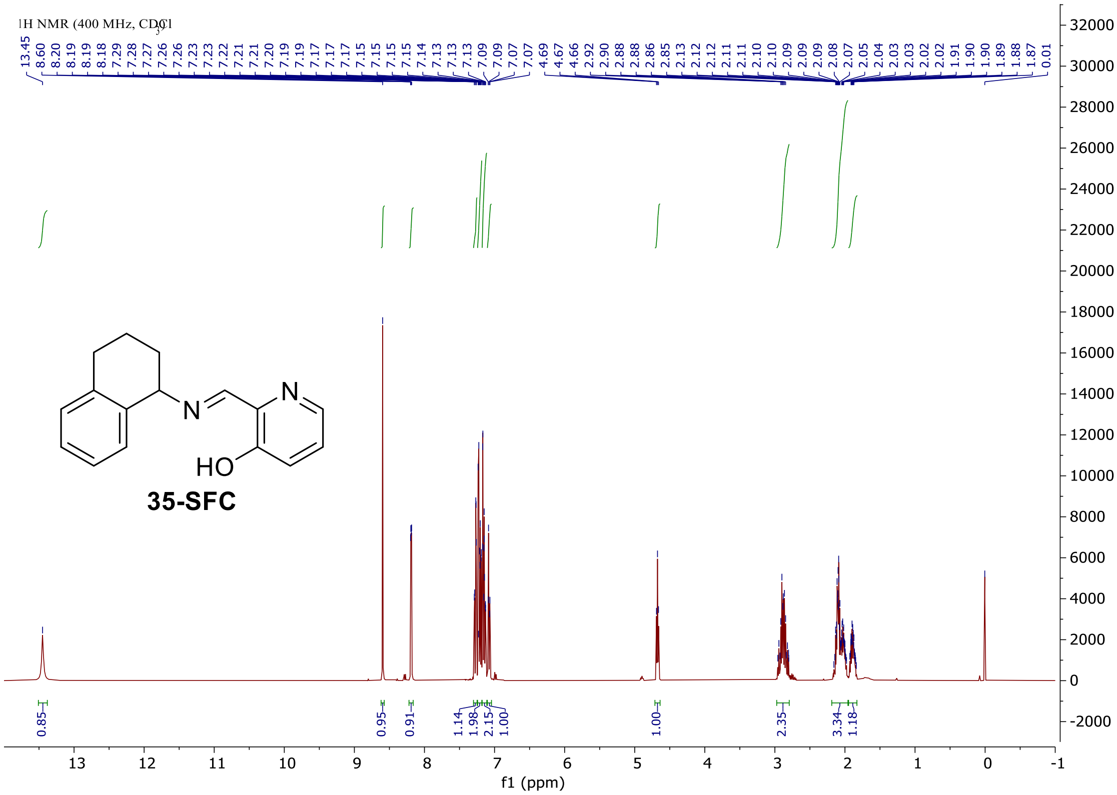
1H NMR (400 MHz, CDCl3
13.45 8.60 8.20 8.19 8.19 8.18 7.29 7.28 7.27 7.26 7.26 7.23 7.23 7.22 7.21 7.21 7.20 7.19 7.19 7.17 7.17 7.17 7.15 7.15 7.15 7.15 7.14 7.13 7.13 7.13 7.09 7.09 7.07 7.07 4.69 4.67 4.66 2.92 2.90 2.88 2.88 2.86 2.85 2.13 2.12 2.12 2.11 2.11 2.10 2.10 2.09 2.09 2.09 2.08 2.07 2.05 2.04 2.03 2.03 2.02 2.02 1.91 1.90 1.89 1.88 1.87 0.01
35-SFC
f1 (ppm)

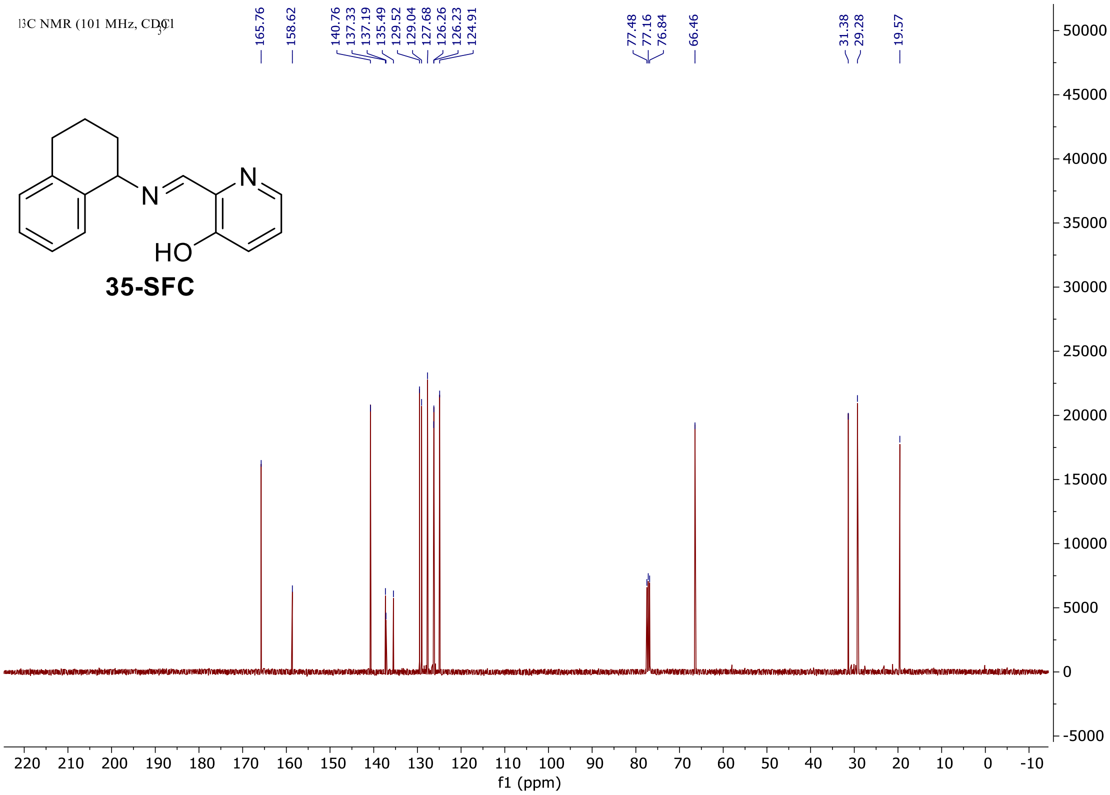
13C NMR (101 MHz, CDCl3
165.76 158.62 140.76 137.33 137.19 135.49 129.52 129.04 127.68 126.26 126.23 124.91 77.48 77.16 76.84 66.46 31.38 29.28 19.57
35-SFC
f1 (ppm)

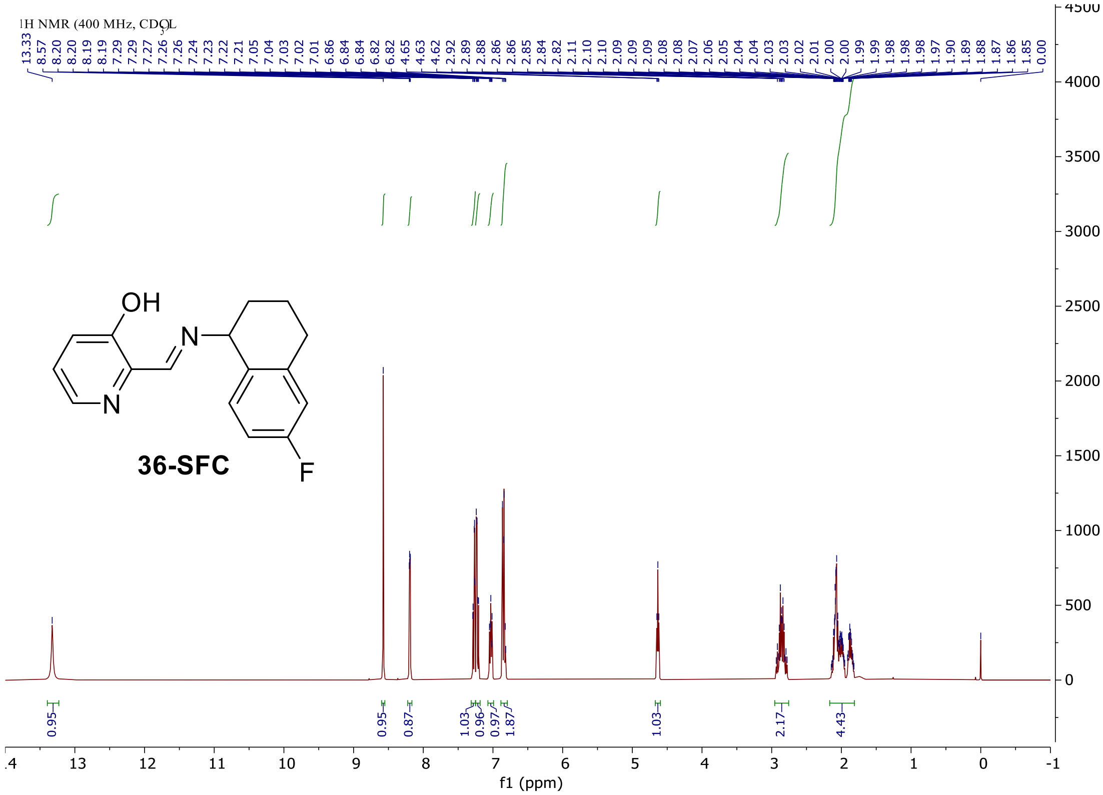
1H NMR (400 MHz, CDCl3)
36-SFC
f1 (ppm)

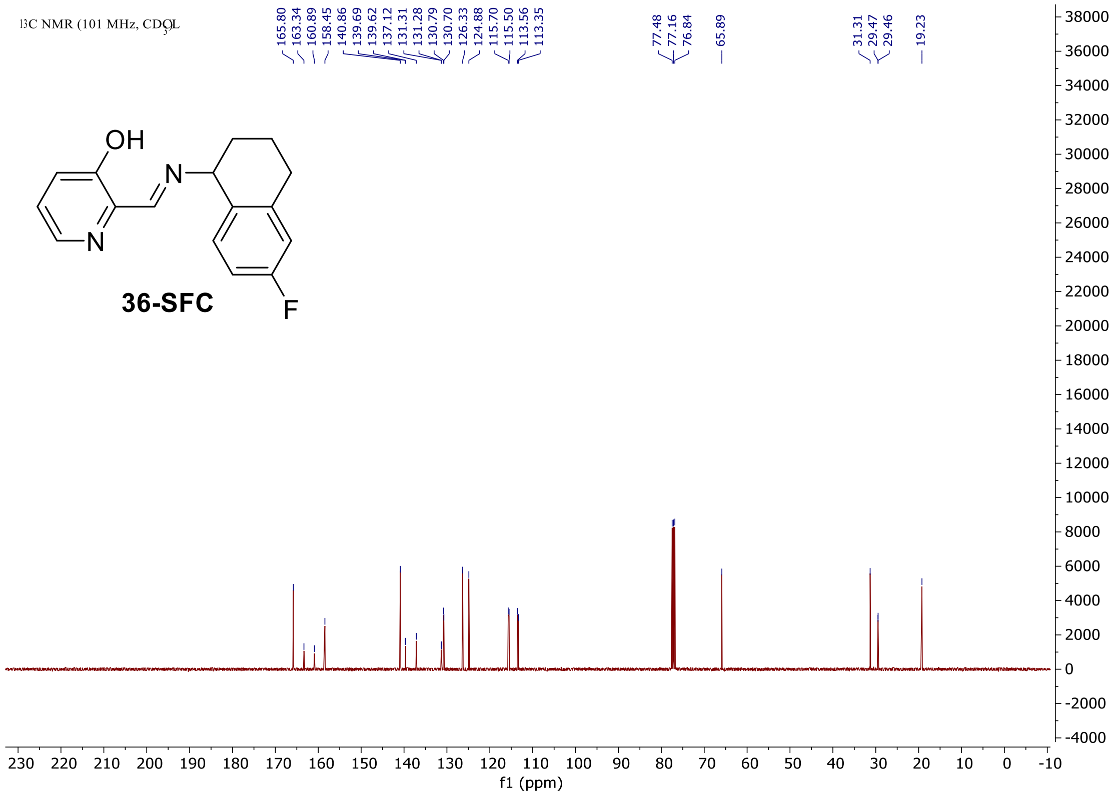
13C NMR (101 MHz, CDCl3)
36-SFC
f1 (ppm)

19F NMR (376 MHz, CDCl3)

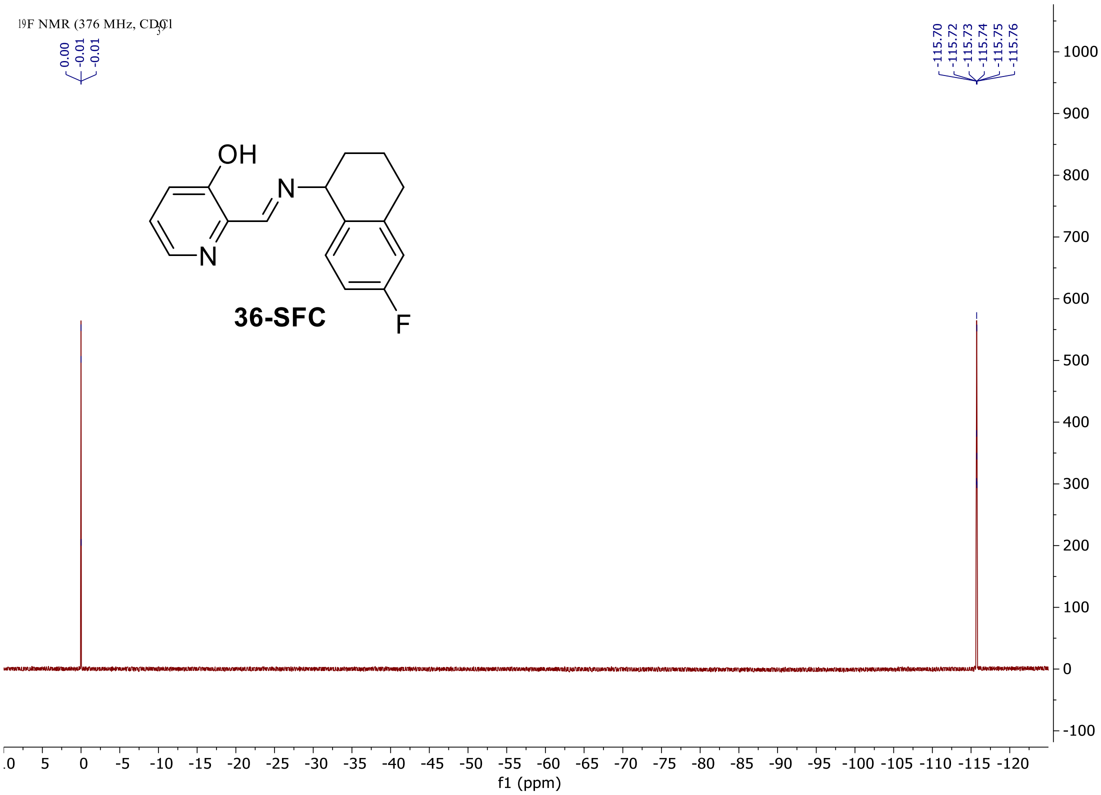

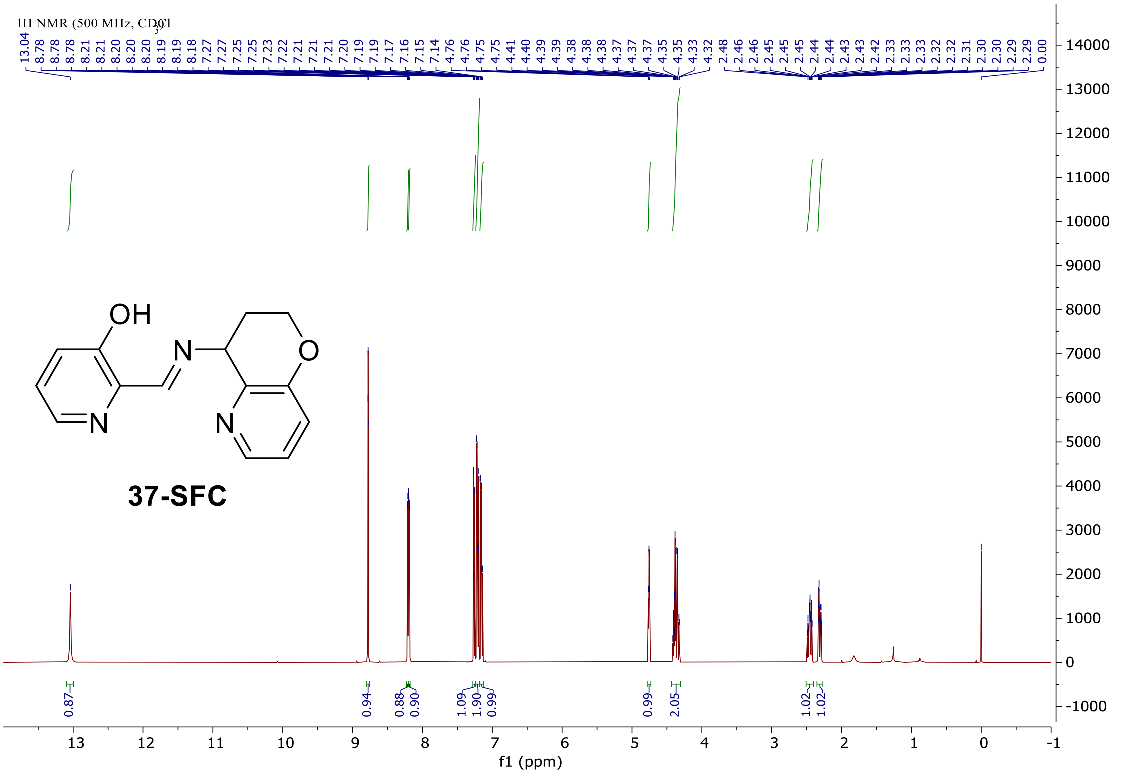
1H NMR (500 MHz, CDCl3)
37-SFC
f1 (ppm)

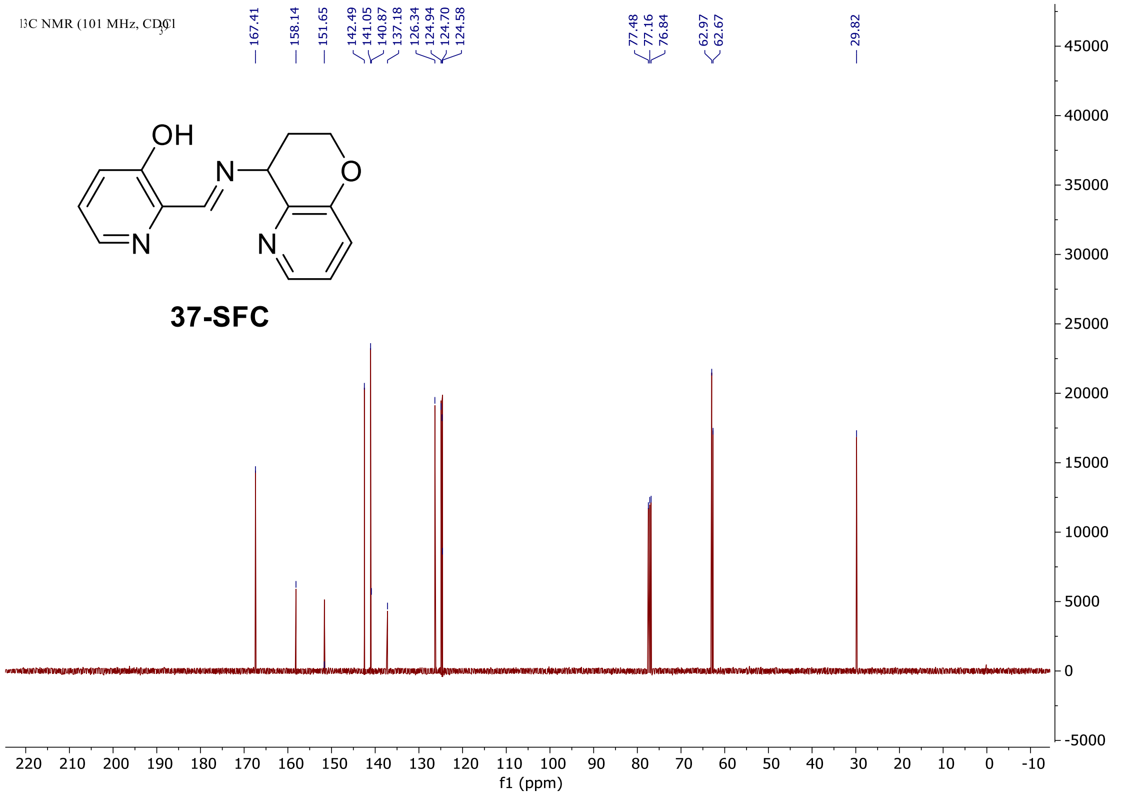
13C NMR (101 MHz, CDCl3)
167.41
158.14
151.65
142.49
141.05
140.87
137.18
126.34
124.94
124.70
124.58
77.48
77.16
76.84
62.97
62.67
29.82
37-SFC
f1 (ppm)

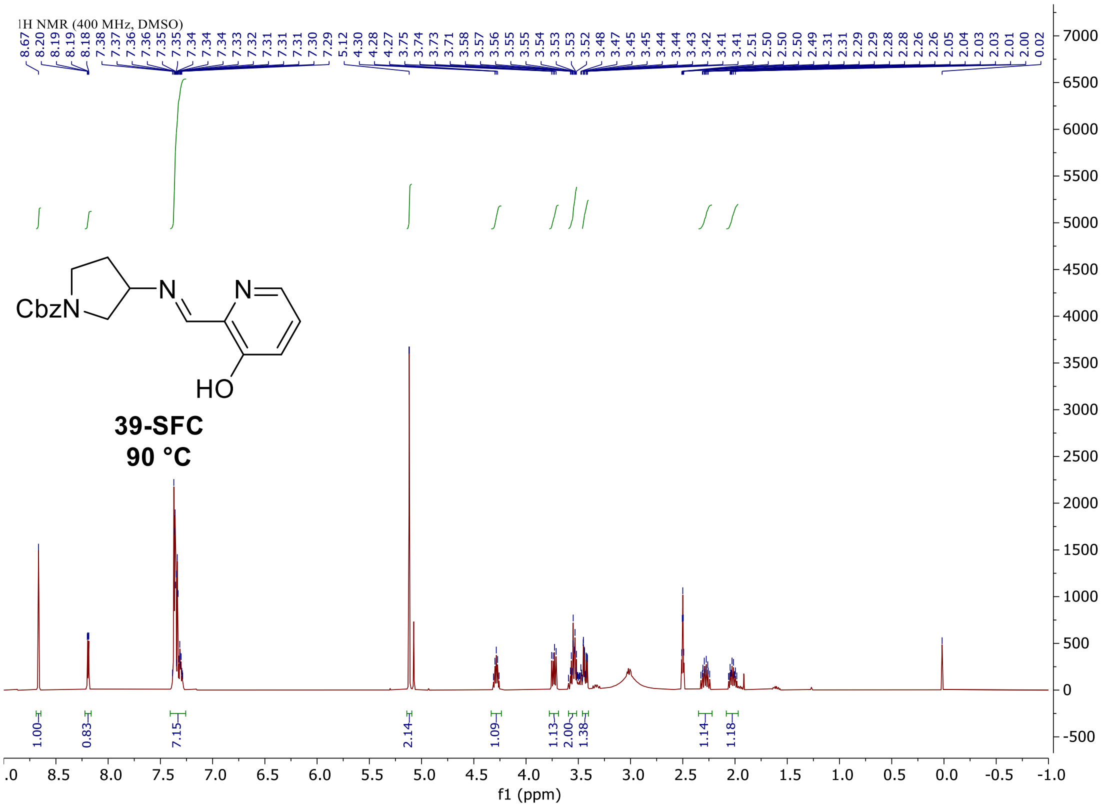
1H NMR (400 MHz, DMSO)
CbzN
N
N
HO
39-SFC
90 °C
f1 (ppm)

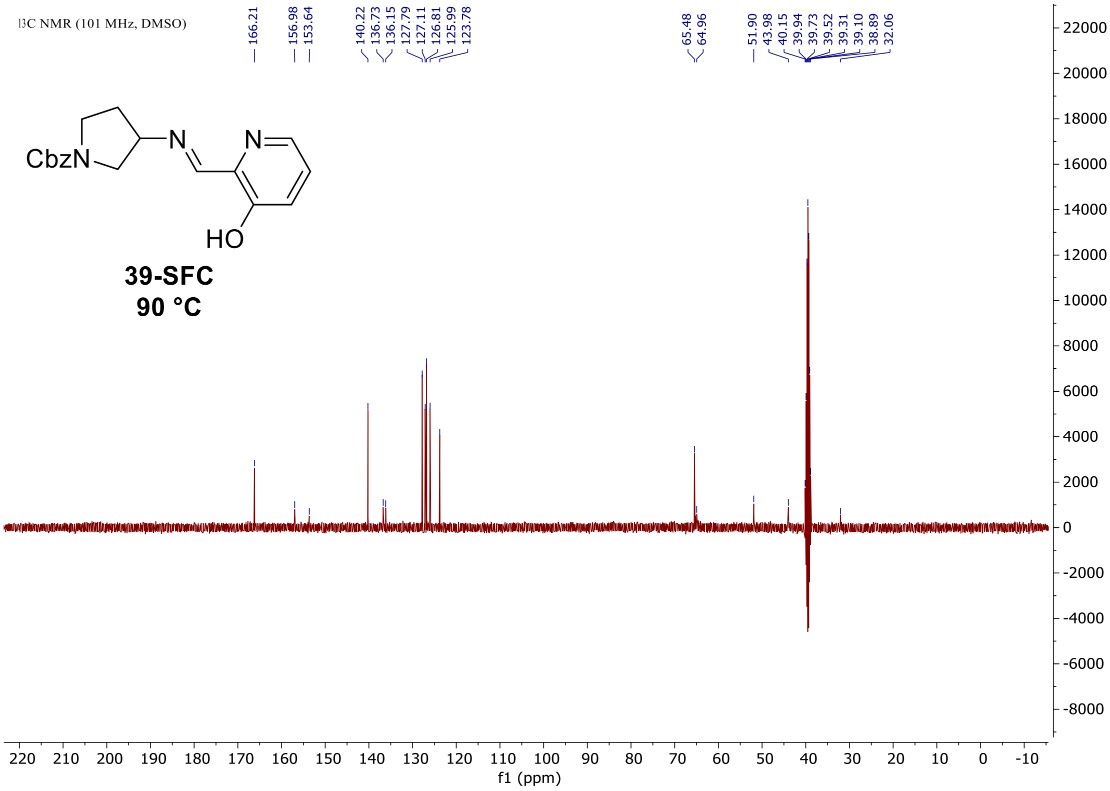
13C NMR (101 MHz, DMSO)
CbzN
N
N
HO
39-SFC
90 °C
f1 (ppm)

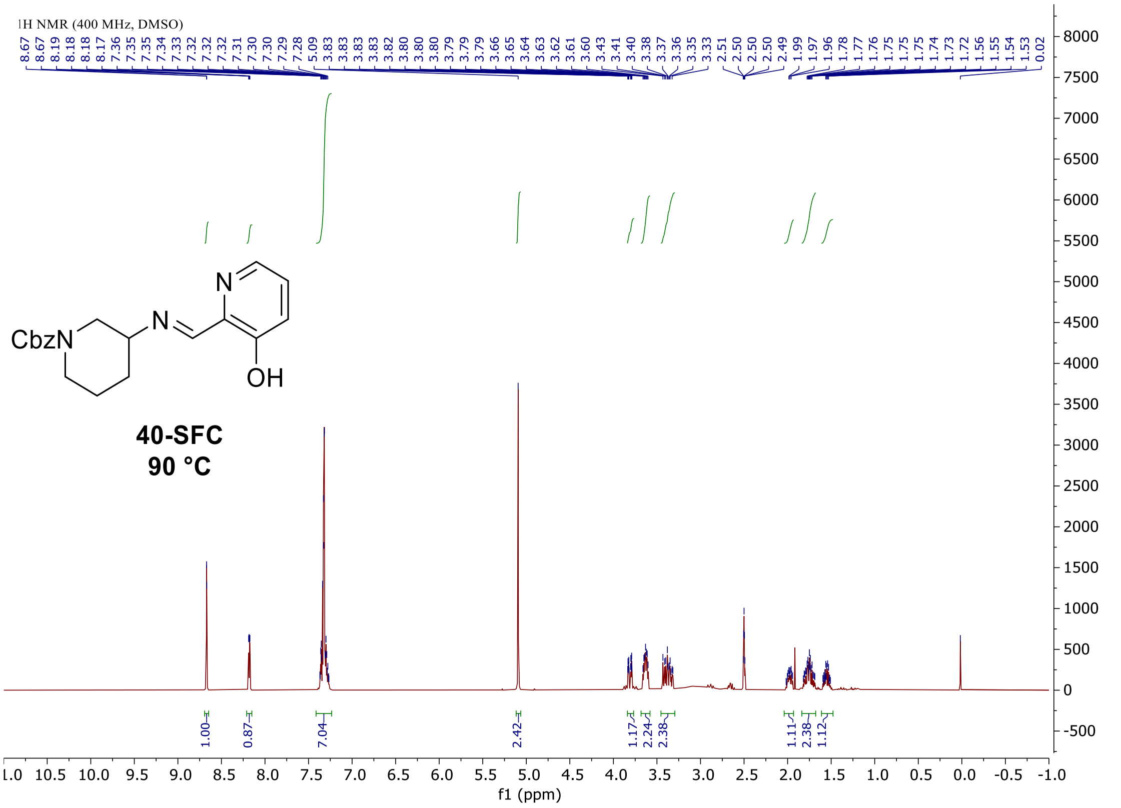
1H NMR (400 MHz, DMSO)
8.67 8.67 8.19 8.18 8.18 8.17 7.36 7.35 7.35 7.34 7.33 7.32 7.32 7.32 7.31 7.30 7.30 7.29 7.28 5.09 3.83 3.83 3.83 3.83 3.82 3.80 3.80 3.80 3.79 3.79 3.79 3.66 3.65 3.64 3.63 3.62 3.61 3.60 3.43 3.41 3.40 3.38 3.37 3.36 3.35 3.33 2.51 2.50 2.50 2.50 2.49 1.99 1.97 1.96 1.78 1.77 1.76 1.75 1.75 1.75 1.74 1.73 1.72 1.56 1.55 1.54 1.53 0.02
CbzN
N
N
OH
40-SFC
90 °C
1.00
0.87
7.04
2.42
1.17
2.24
2.38
1.11
2.38
1.12
f1 (ppm)

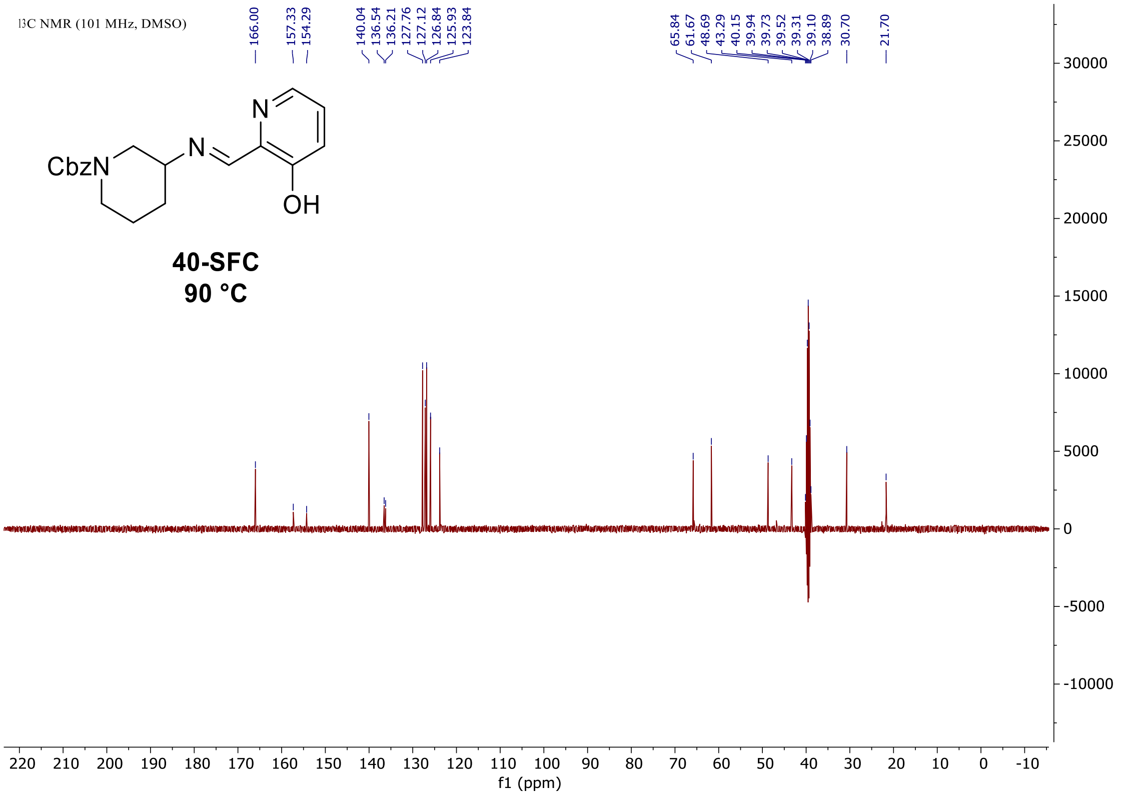
13C NMR (101 MHz, DMSO)
166.00 157.33 154.29 140.04 136.54 136.21 127.76 127.12 126.84 125.93 123.84 65.84 61.67 48.69 43.29 40.15 39.94 39.73 39.52 39.31 39.10 38.89 30.70 21.70
CbzN
N
N
OH
40-SFC
90 °C
f1 (ppm)

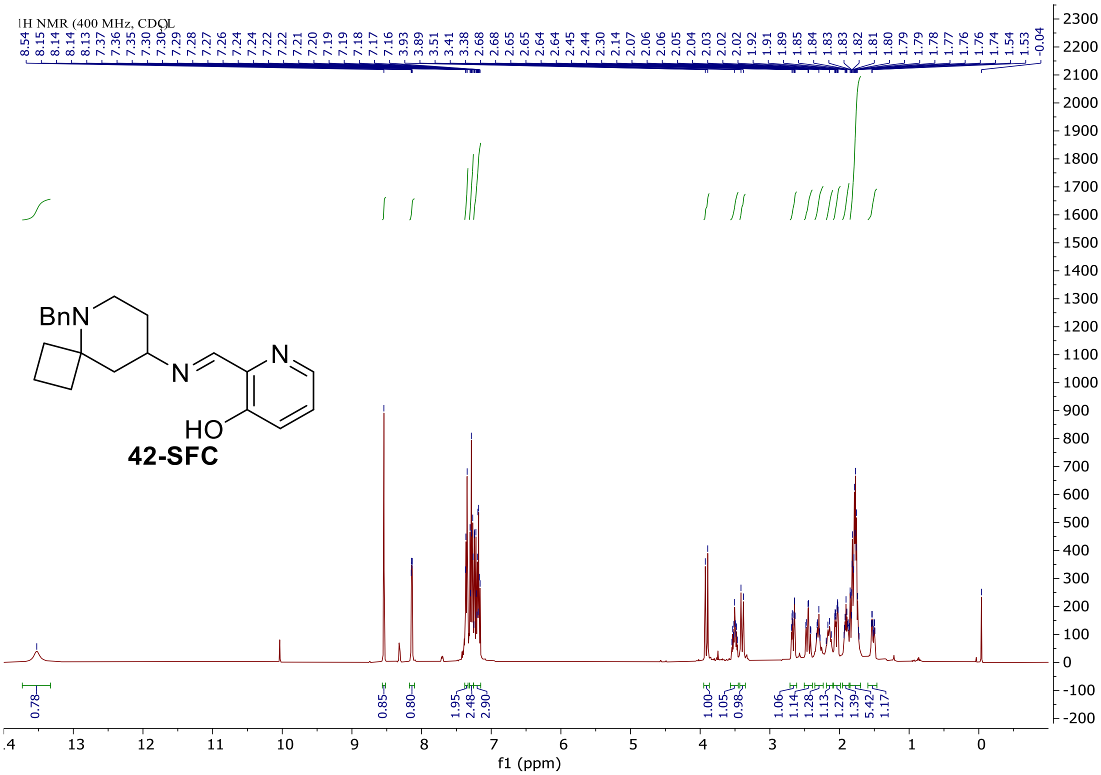
1H NMR (400 MHz, CDCl3)
8.54 8.15 8.14 8.13 7.37 7.36 7.35 7.30 7.30 7.29 7.28 7.27 7.26 7.24 7.24 7.22 7.22 7.21 7.20 7.19 7.19 7.18 7.17 7.16 3.93 3.89 3.51 3.41 3.38 2.68 2.68 2.65 2.65 2.64 2.45 2.44 2.30 2.14 2.07 2.06 2.06 2.05 2.04 2.03 2.02 2.02 1.92 1.91 1.89 1.85 1.84 1.83 1.83 1.82 1.81 1.80 1.79 1.79 1.78 1.77 1.76 1.76 1.74 1.54 1.53 -0.04
BnN
N
N
HO
42-SFC
0.78 0.85 0.80 1.95 2.48 2.90 1.00 1.05 0.98 1.06 1.14 1.28 1.13 1.27 1.39 5.42 1.17
f1 (ppm)

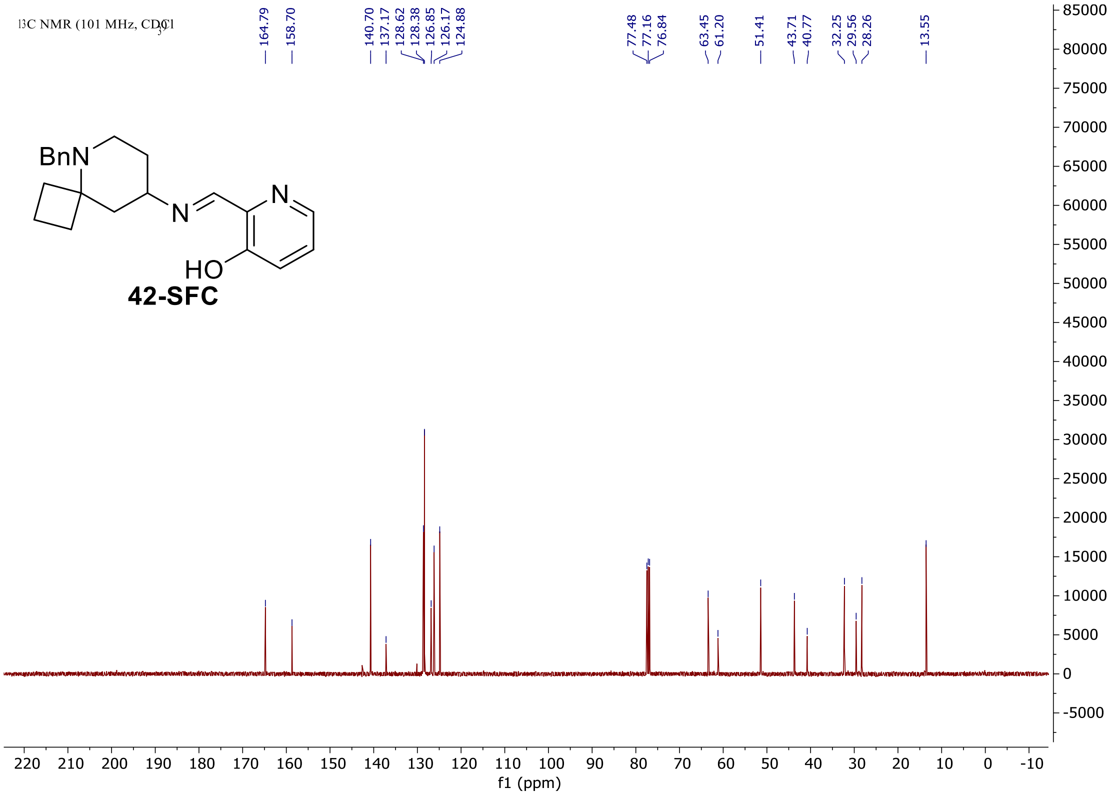
13C NMR (101 MHz, CDCl3)
164.79 158.70 140.70 137.17 128.62 128.38 126.85 126.17 124.88 77.48 77.16 76.84 63.45 61.20 51.41 43.71 40.77 32.25 29.56 28.26 13.55
BnN
N
N
HO
42-SFC
f1 (ppm)

1H NMR (600 MHz, $CDCl_3$)

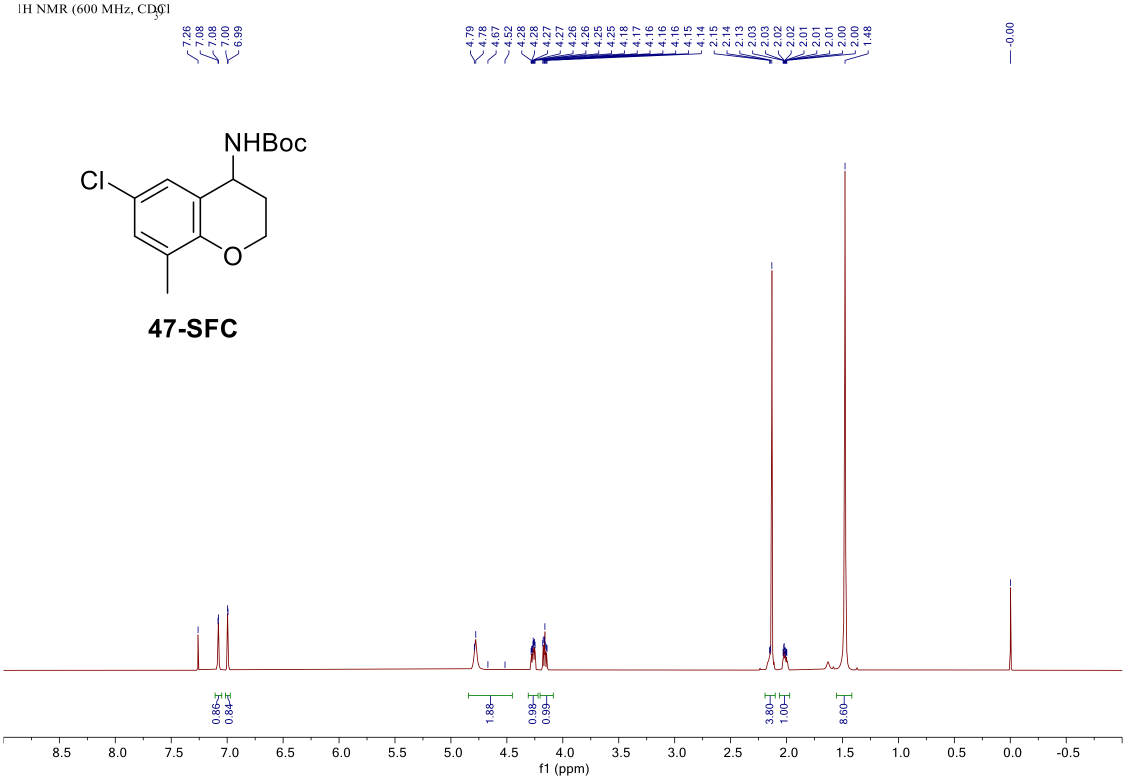

47-SFC

13C NMR (151 MHz, $CDCl_3$)

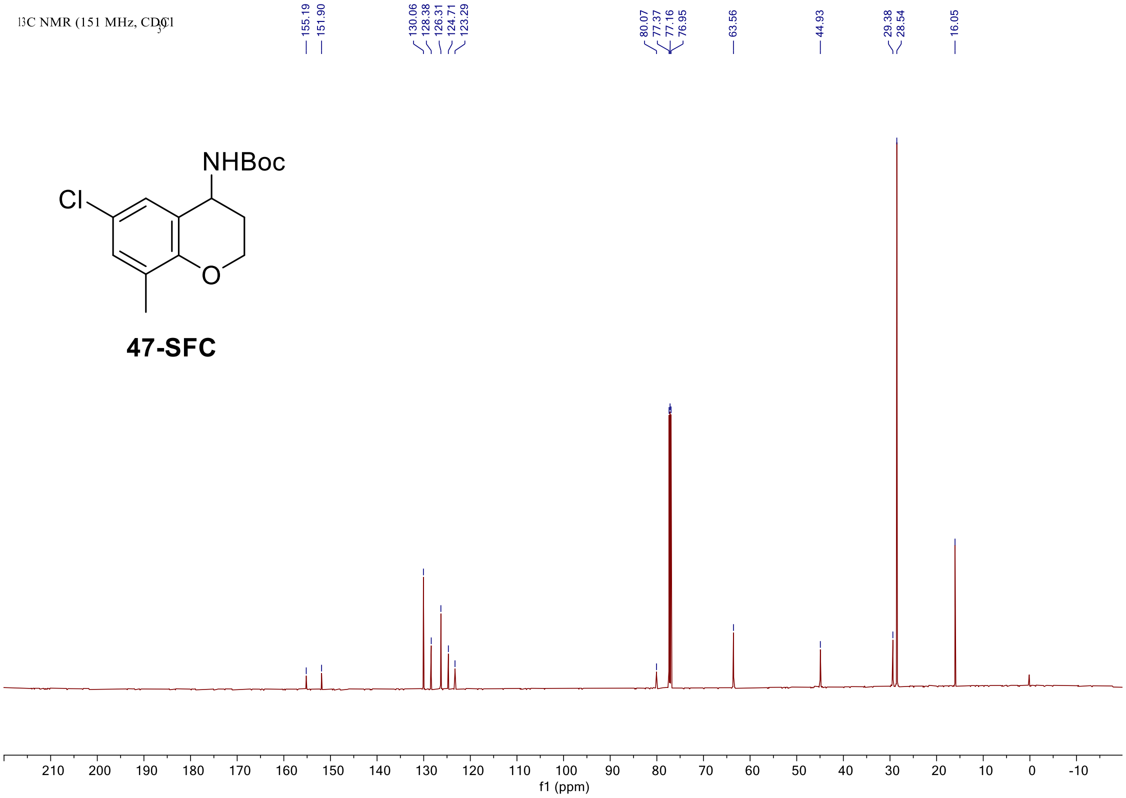

47-SFC

1H NMR (400 MHz, DMSO)

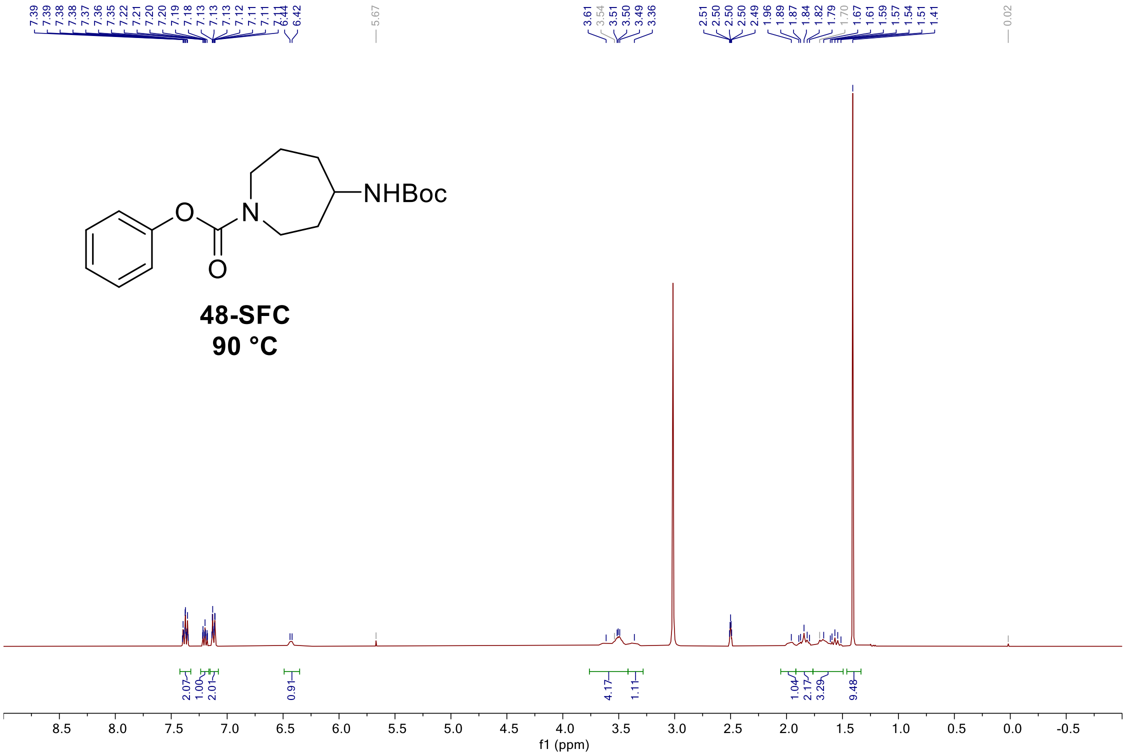

13C NMR (101 MHz, DMSO)

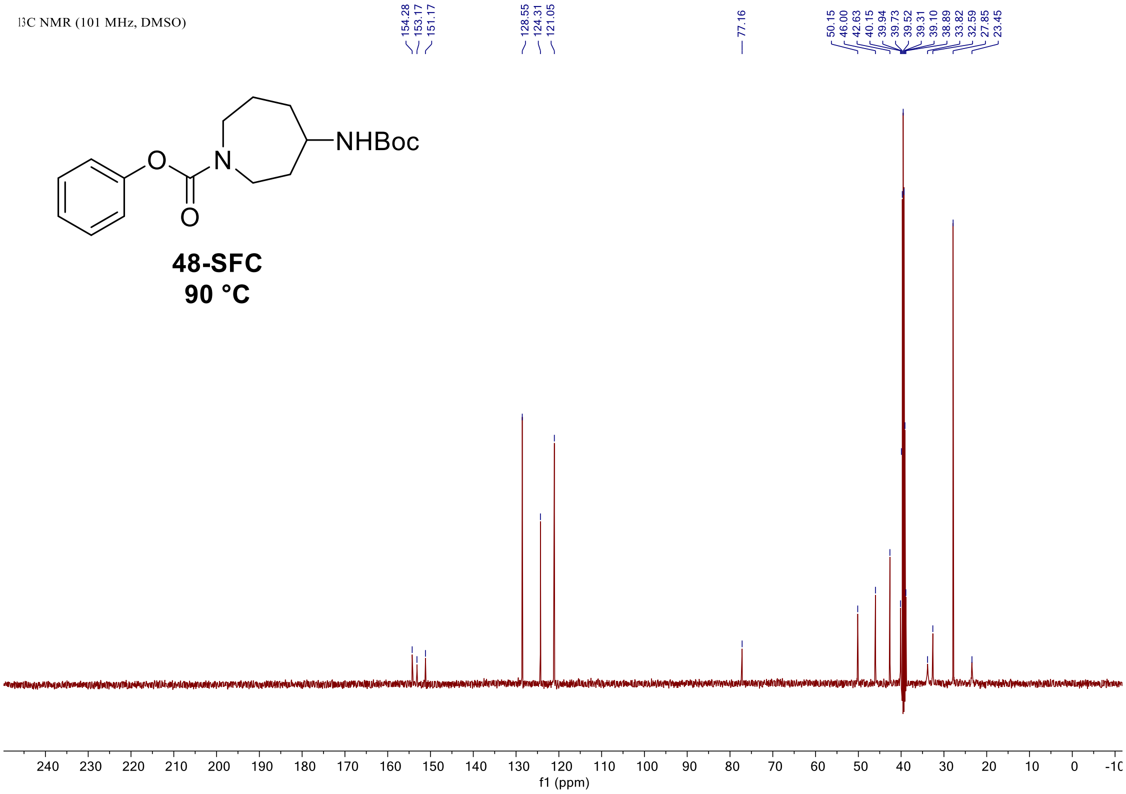

1H NMR (400 MHz, CDCl3

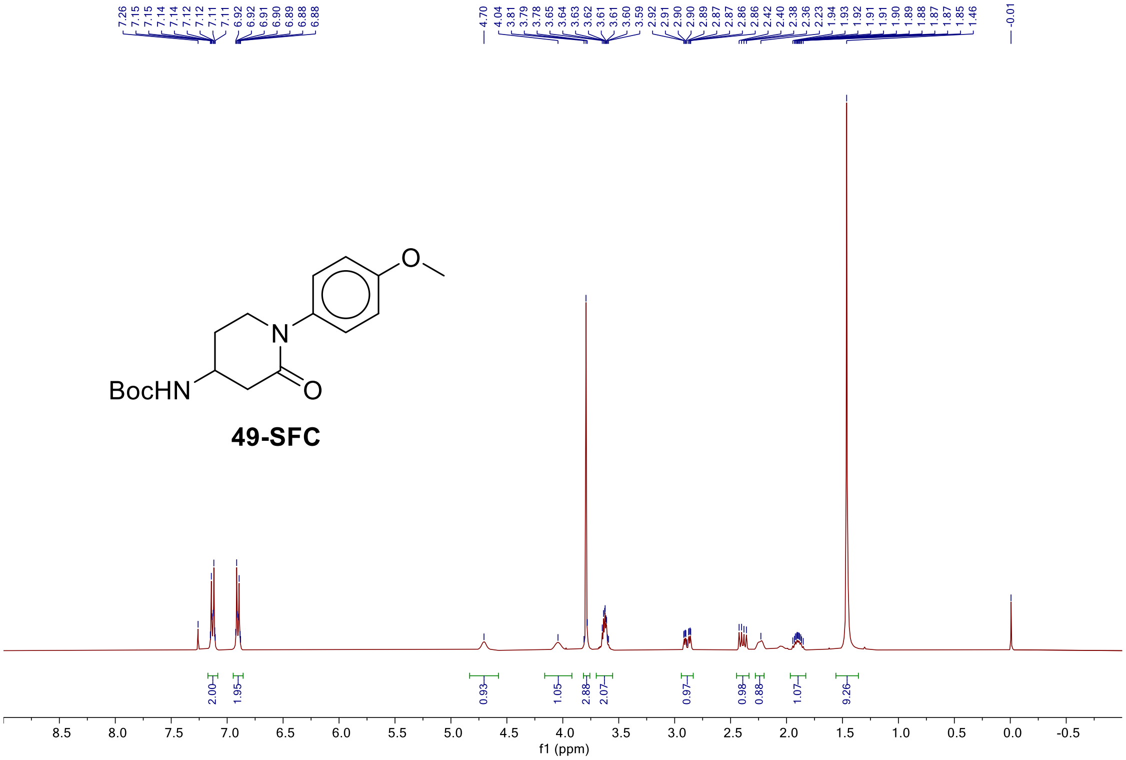

13C NMR (101 MHz, CDCl3

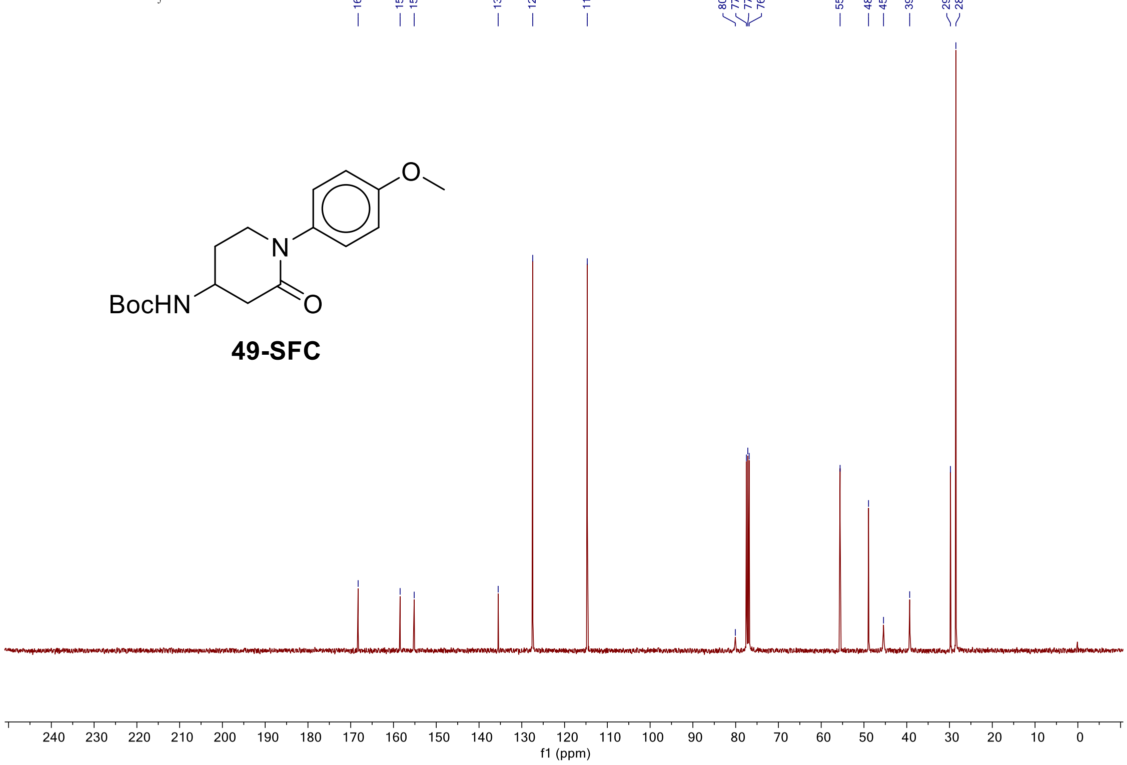

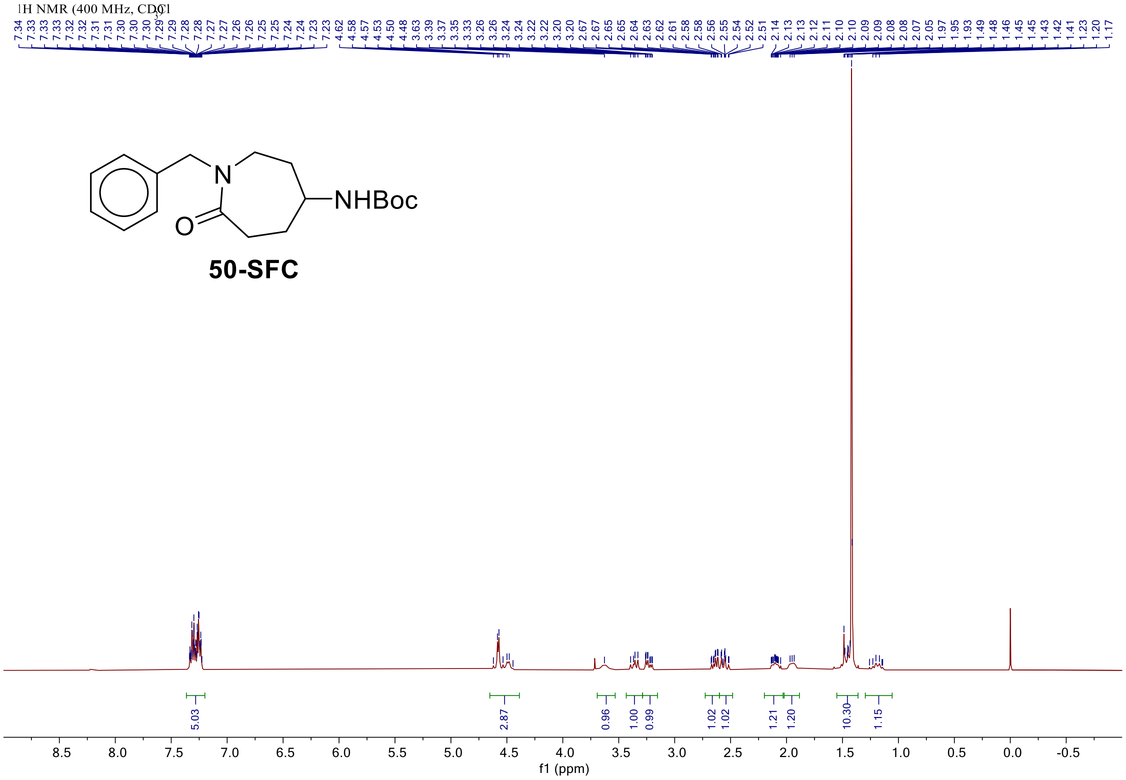
1H NMR (400 MHz, CDCl3)
7.34 7.33 7.33 7.32 7.32 7.31 7.31 7.30 7.30 7.30 7.29 7.29 7.28 7.28 7.27 7.27 7.26 7.26 7.25 7.25 7.24 7.24 7.23 7.23 4.62 4.58 4.57 4.53 4.50 4.48 3.63 3.39 3.37 3.35 3.33 3.26 3.26 3.24 3.24 3.22 3.22 3.20 3.20 2.67 2.67 2.65 2.65 2.64 2.63 2.62 2.61 2.58 2.58 2.56 2.55 2.54 2.52 2.51 2.14 2.13 2.13 2.12 2.11 2.10 2.10 2.09 2.09 2.08 2.08 2.07 2.05 1.97 1.95 1.93 1.49 1.48 1.46 1.45 1.45 1.43 1.42 1.41 1.23 1.20 1.17
NHBoc
N
O
50-SFC
5.03
2.87
0.96
1.00
0.99
1.02
1.02
1.21
1.20
10.30
1.15
8.5 8.0 7.5 7.0 6.5 6.0 5.5 5.0 4.5 4.0 3.5 3.0 2.5 2.0 1.5 1.0 0.5 0.0 -0.5
f1 (ppm)

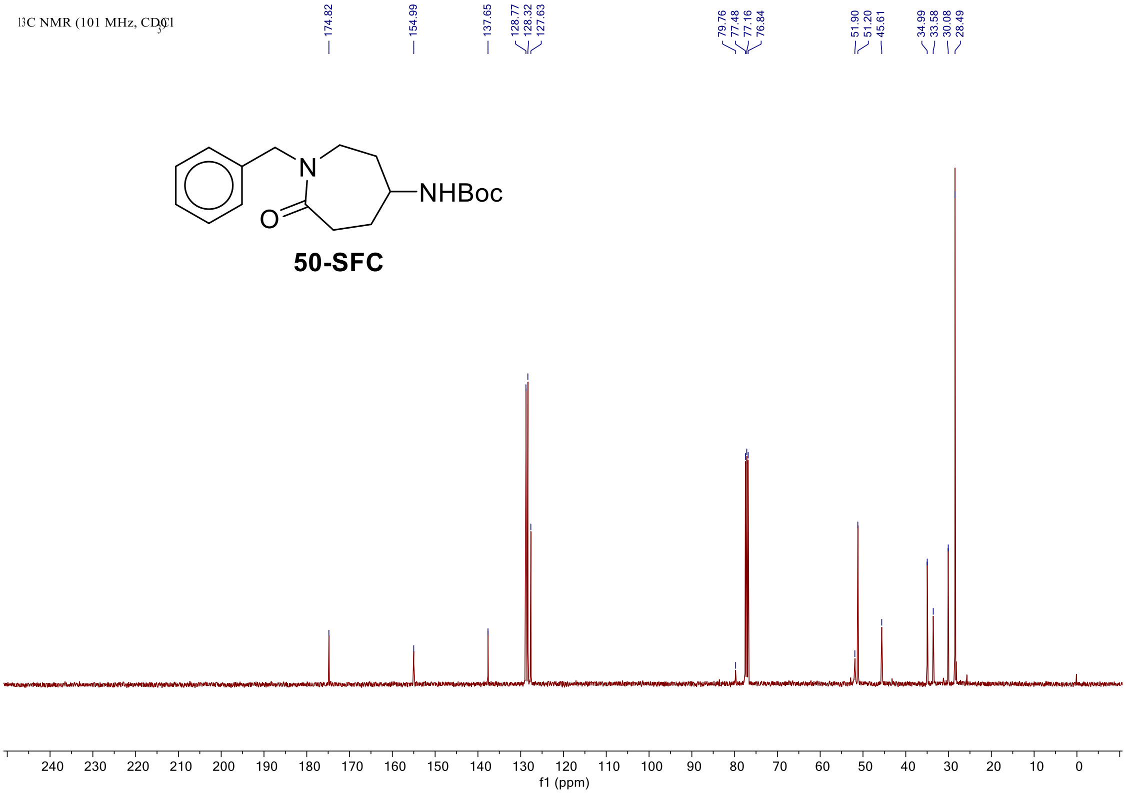
13C NMR (101 MHz, CDCl3)
174.82
154.99
137.65 128.77 128.32 127.63
79.76 77.48 77.16 76.84
51.90 51.20 45.61
34.99 33.58 30.08 28.49
NHBoc
N
O
50-SFC
240 230 220 210 200 190 180 170 160 150 140 130 120 110 100 90 80 70 60 50 40 30 20 10 0
f1 (ppm)

1H NMR (600 MHz, $CDCl_3$)

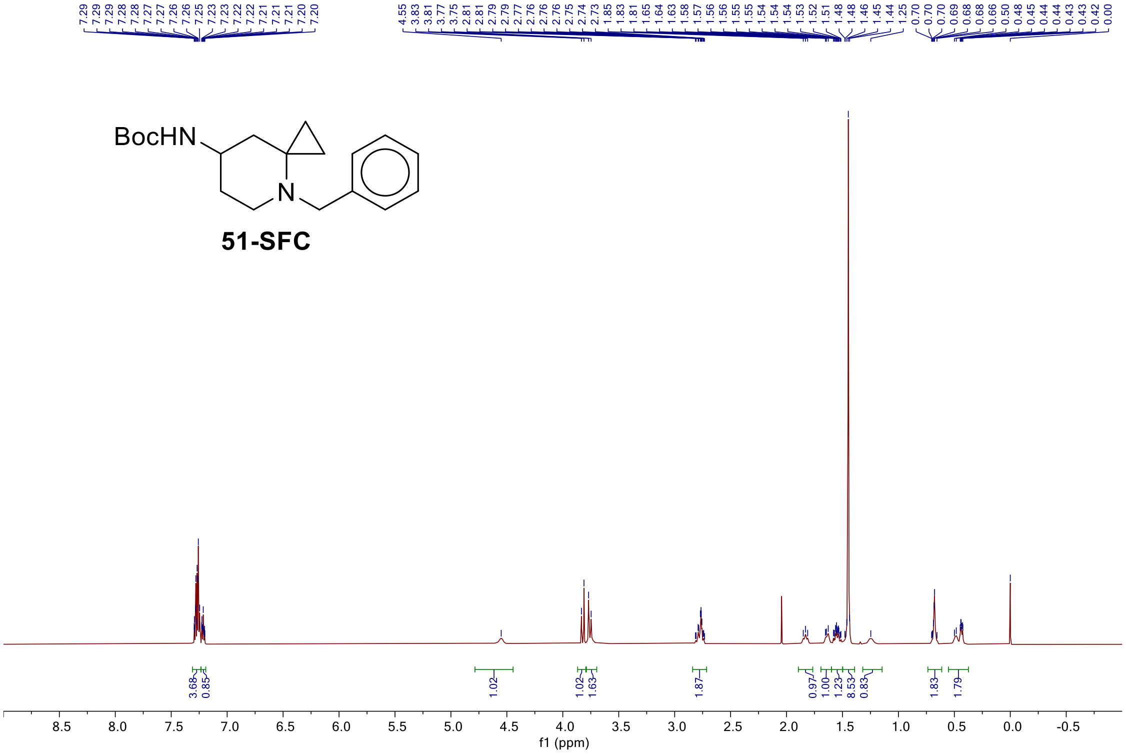

13C NMR (151 MHz, $CDCl_3$)

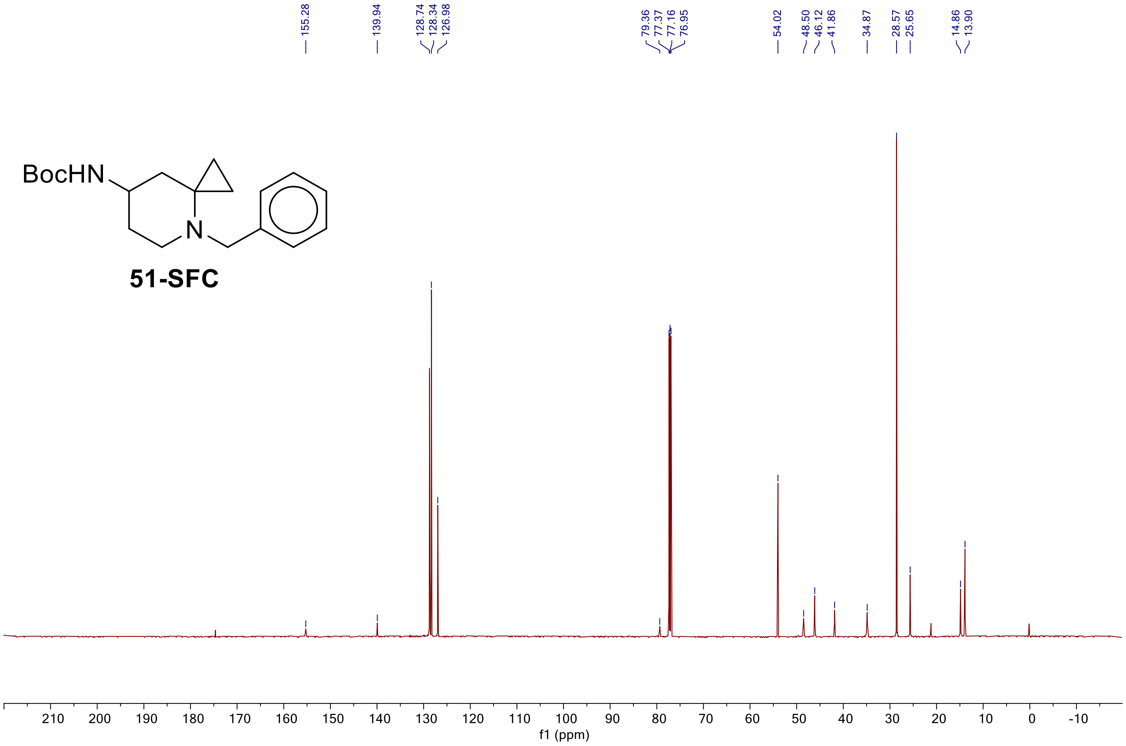

1H NMR (400 MHz, $CDCl_3$)

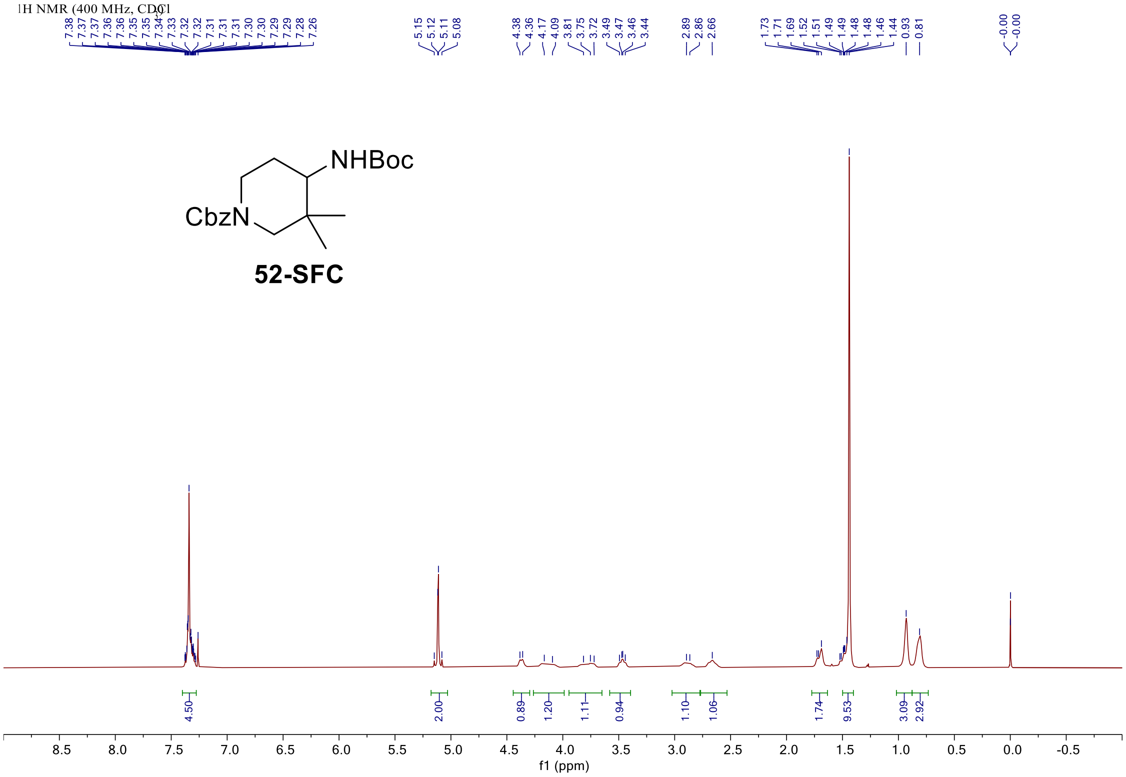

13C NMR (101 MHz, $CDCl_3$)

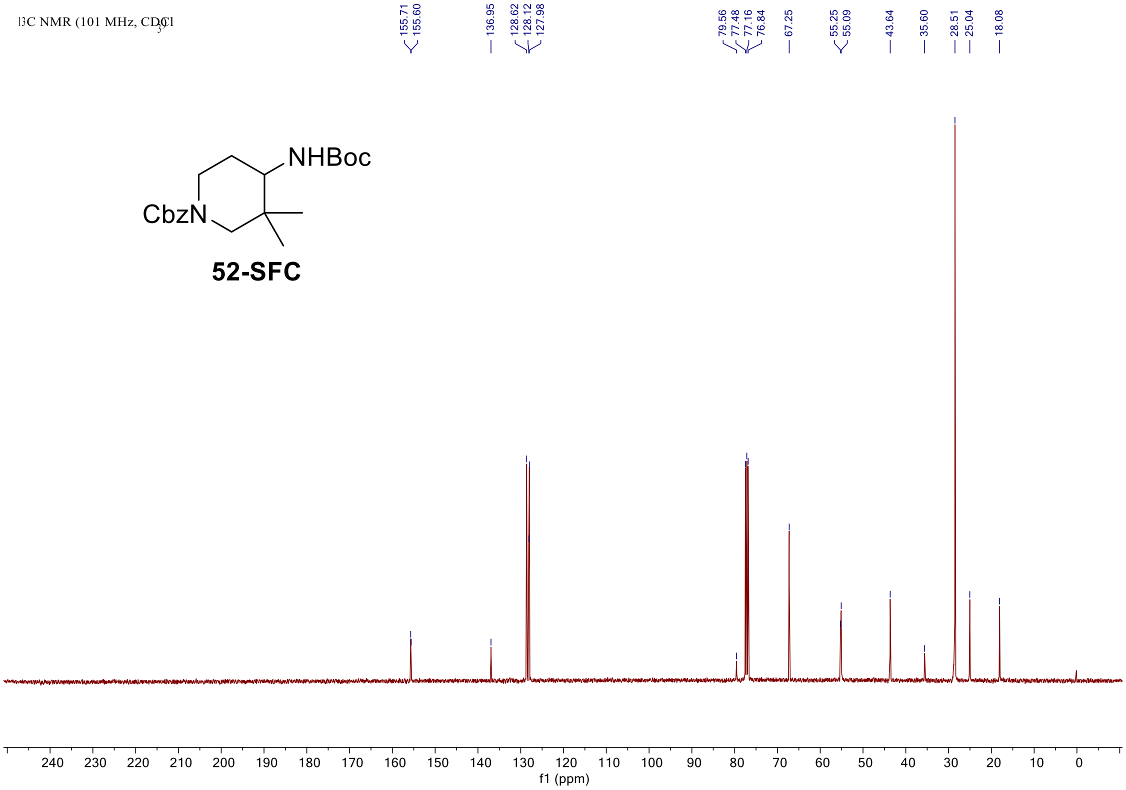

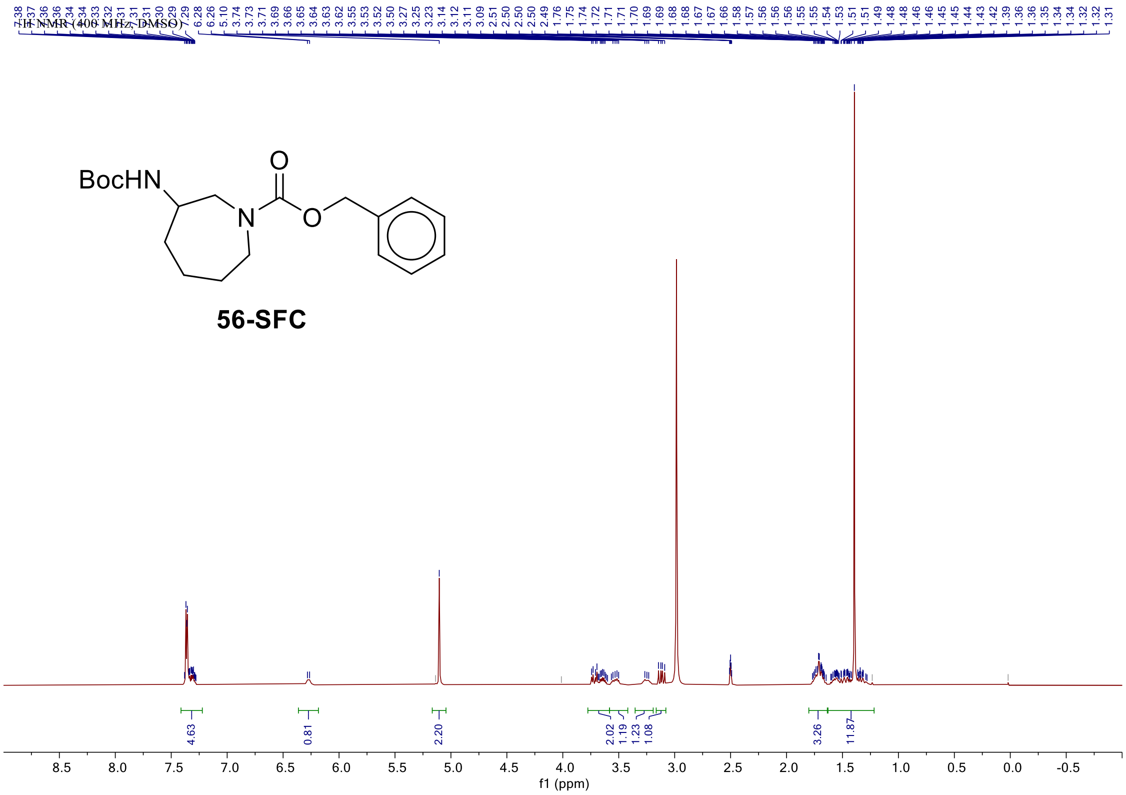
1H NMR (400 MHz, DMSO)
7.38 7.37 7.36 7.36 7.34 7.33 7.32 7.31 7.31 7.30 7.29 7.29 6.28 6.26 5.10 3.74 3.73 3.71 3.69 3.66 3.65 3.64 3.63 3.62 3.55 3.53 3.52 3.50 3.27 3.25 3.23 3.14 3.12 3.11 3.09 2.51 2.50 2.50 2.49 1.76 1.75 1.74 1.72 1.71 1.71 1.70 1.69 1.69 1.68 1.68 1.67 1.66 1.58 1.57 1.56 1.56 1.55 1.55 1.54 1.53 1.51 1.51 1.49 1.48 1.48 1.46 1.45 1.45 1.44 1.43 1.42 1.39 1.36 1.36 1.35 1.34 1.32 1.31
BocHN
O
N
O
56-SFC
4.63
0.81
2.20
2.02
1.19
1.23
1.08
3.26
11.87
f1 (ppm)

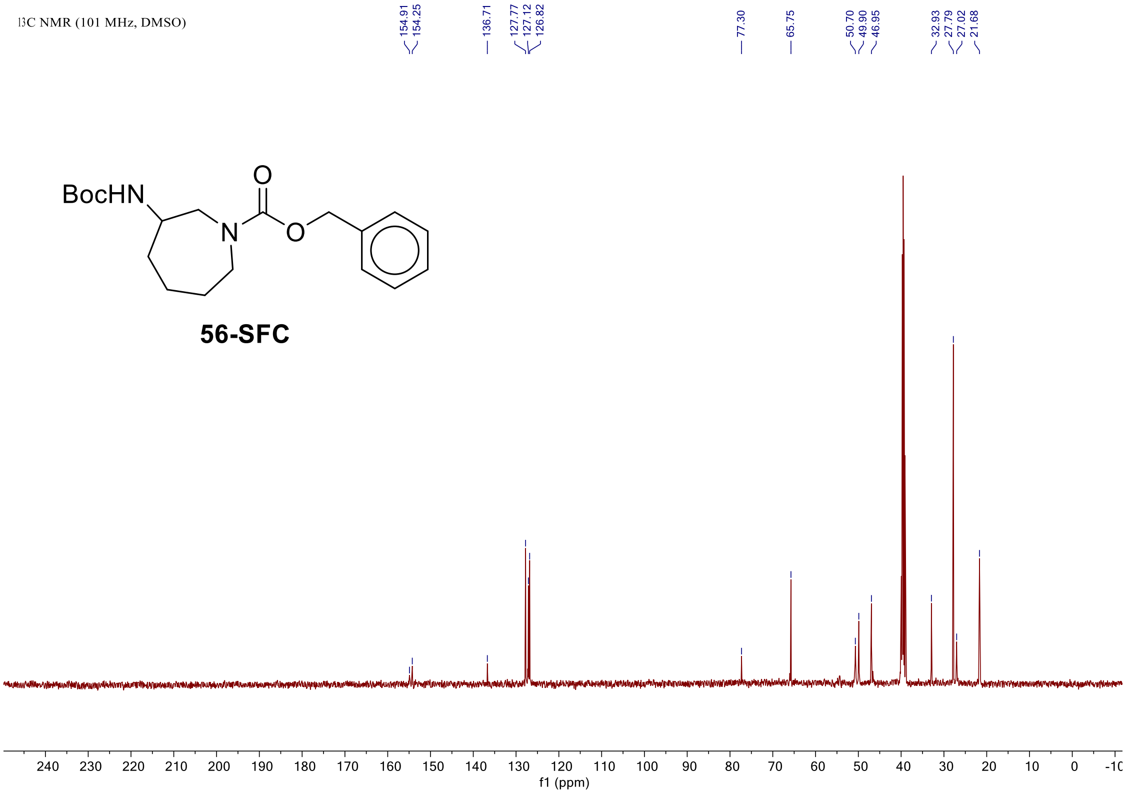
13C NMR (101 MHz, DMSO)
154.91 154.25 136.71 127.77 127.12 126.82 77.30 65.75 50.70 49.90 46.95 32.93 27.79 27.02 21.68
BocHN
O
N
O
56-SFC
f1 (ppm)

1H NMR (400 MHz, CD_3Cl

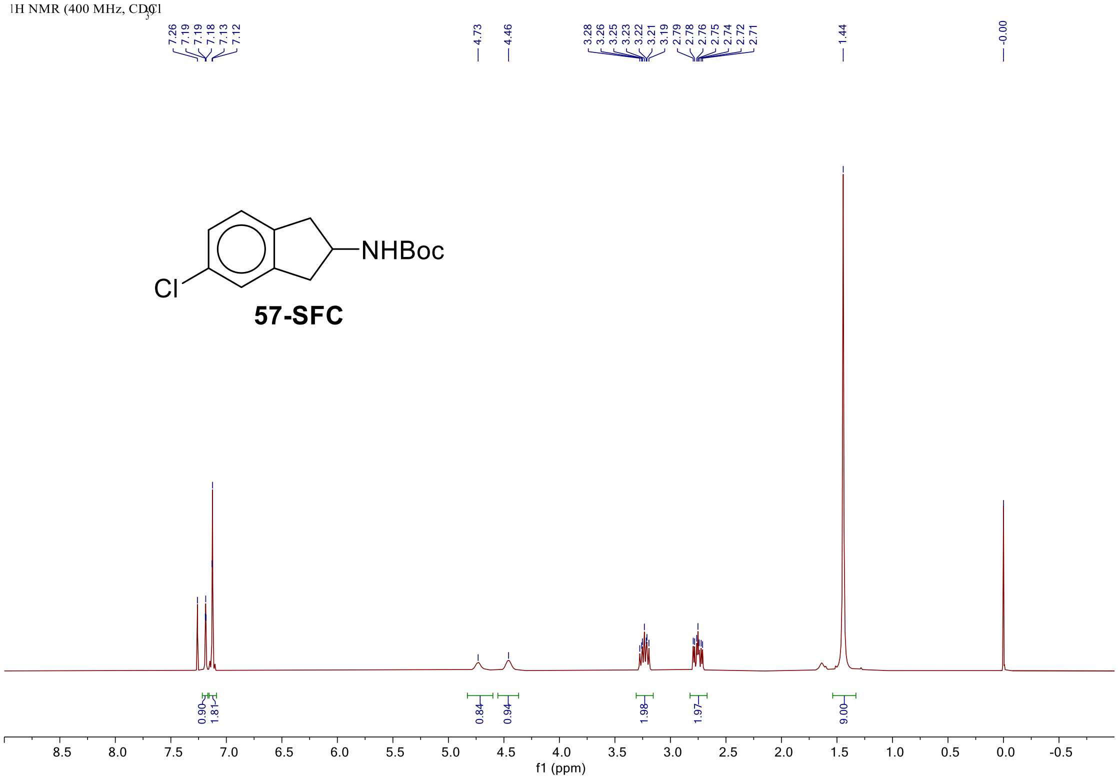

13C NMR (101 MHz, CD_3Cl

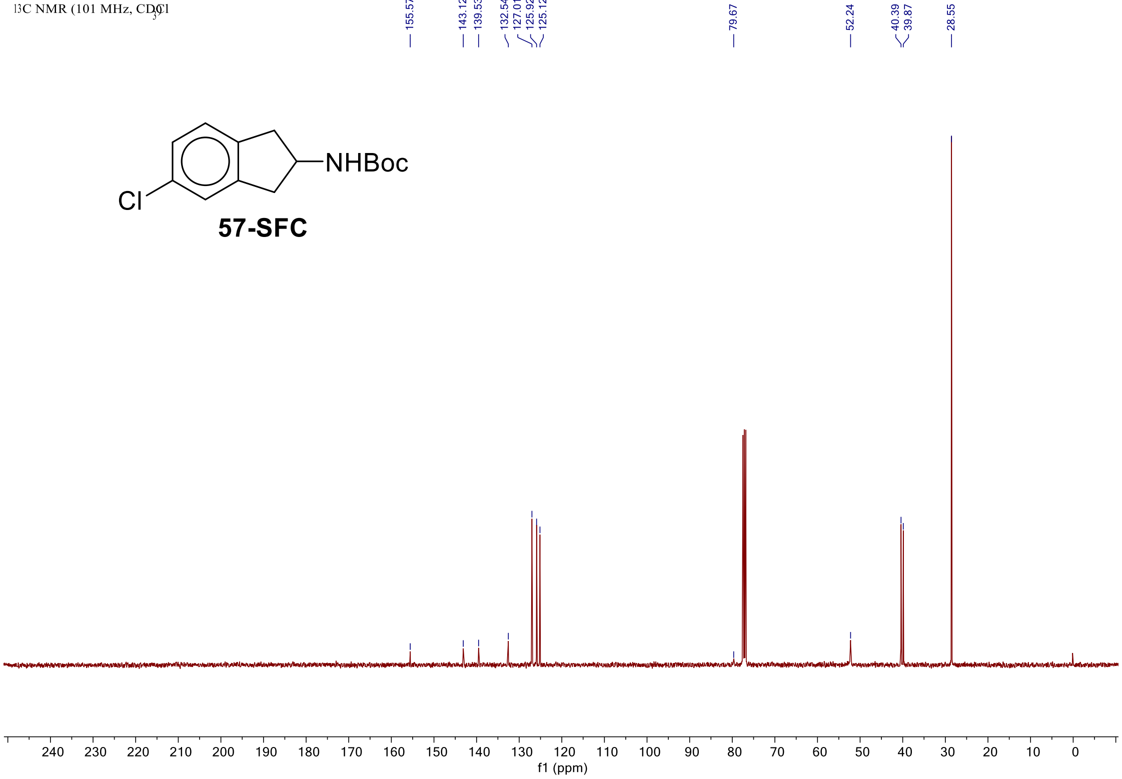

1H NMR (400 MHz, $CDCl_3$

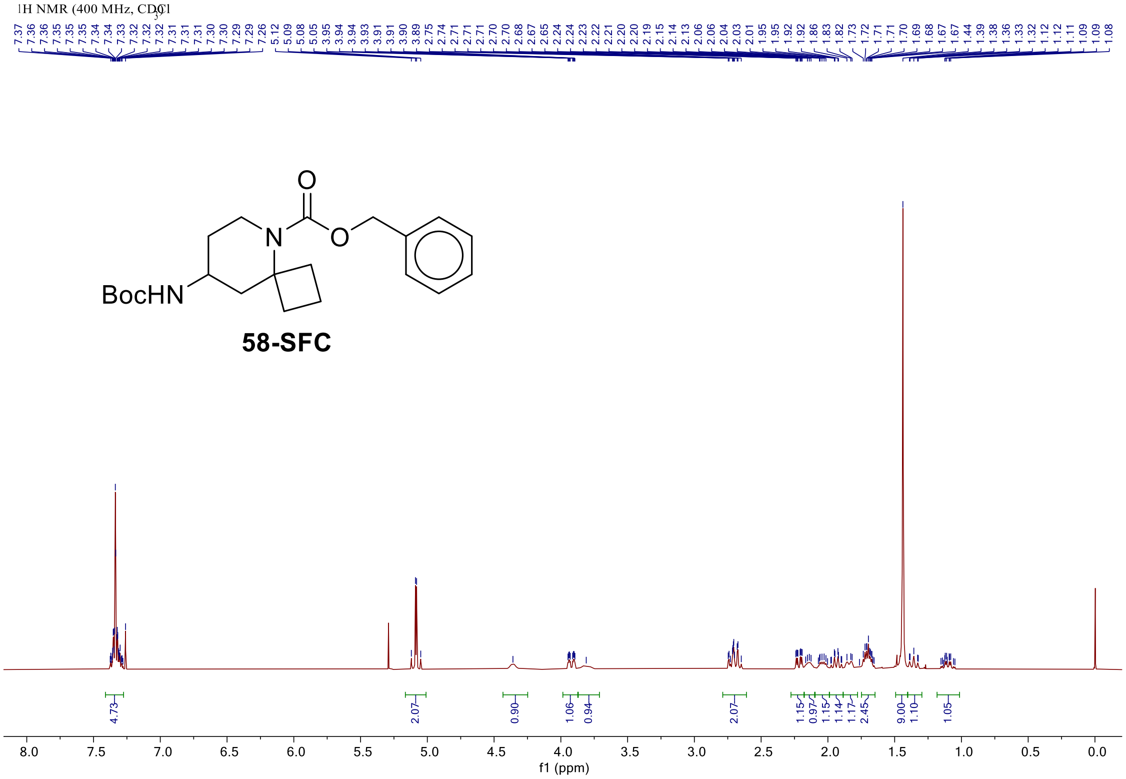

13C NMR (101 MHz, $CDCl_3$

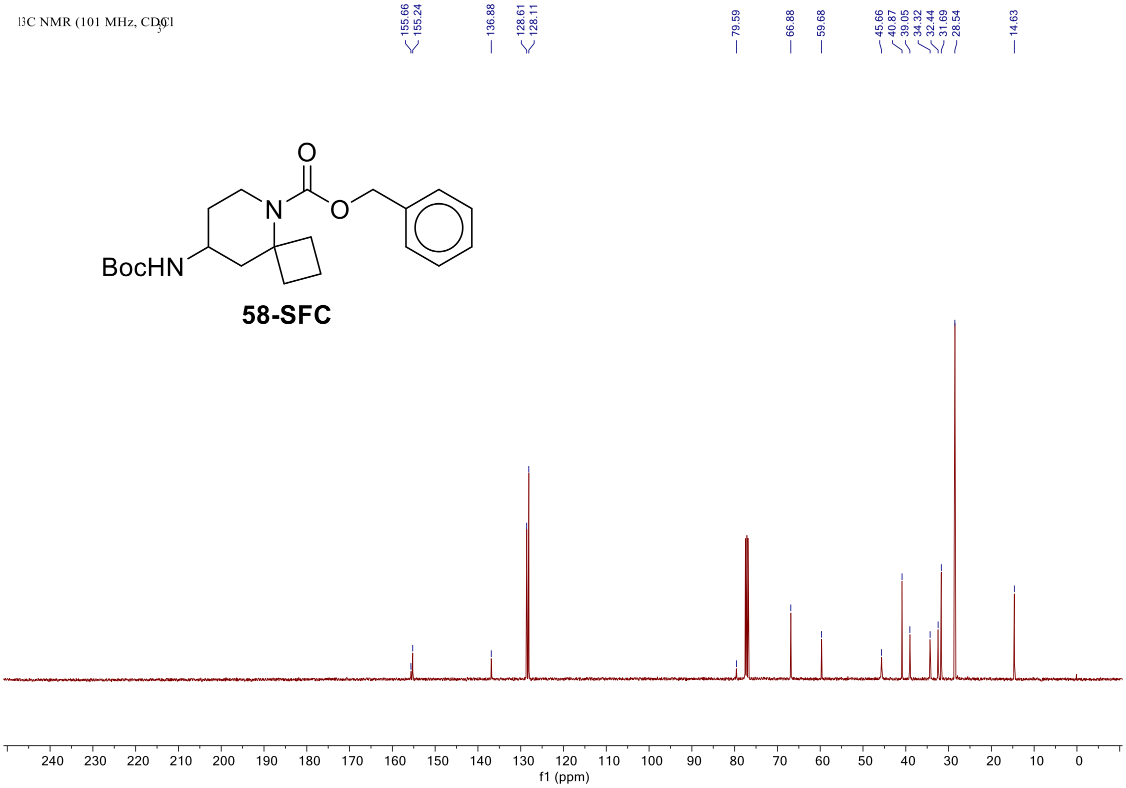

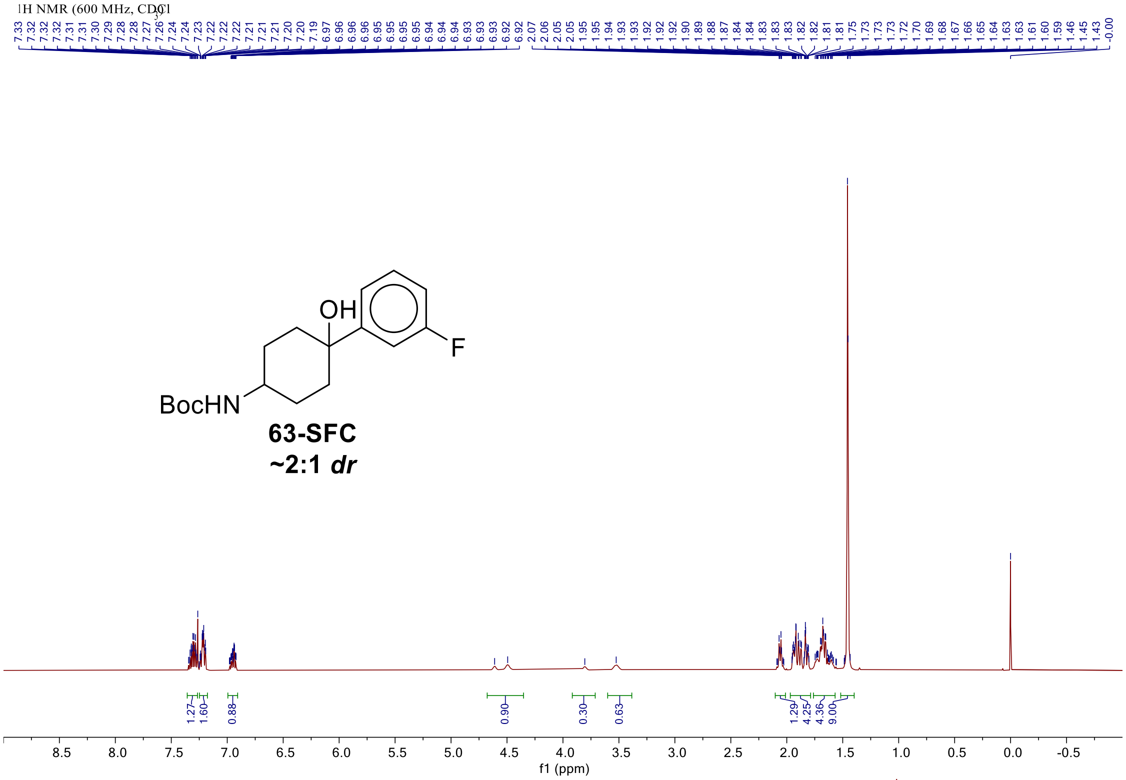
1H NMR (600 MHz, CDCl
63-SFC
~2:1 dr
f1 (ppm)

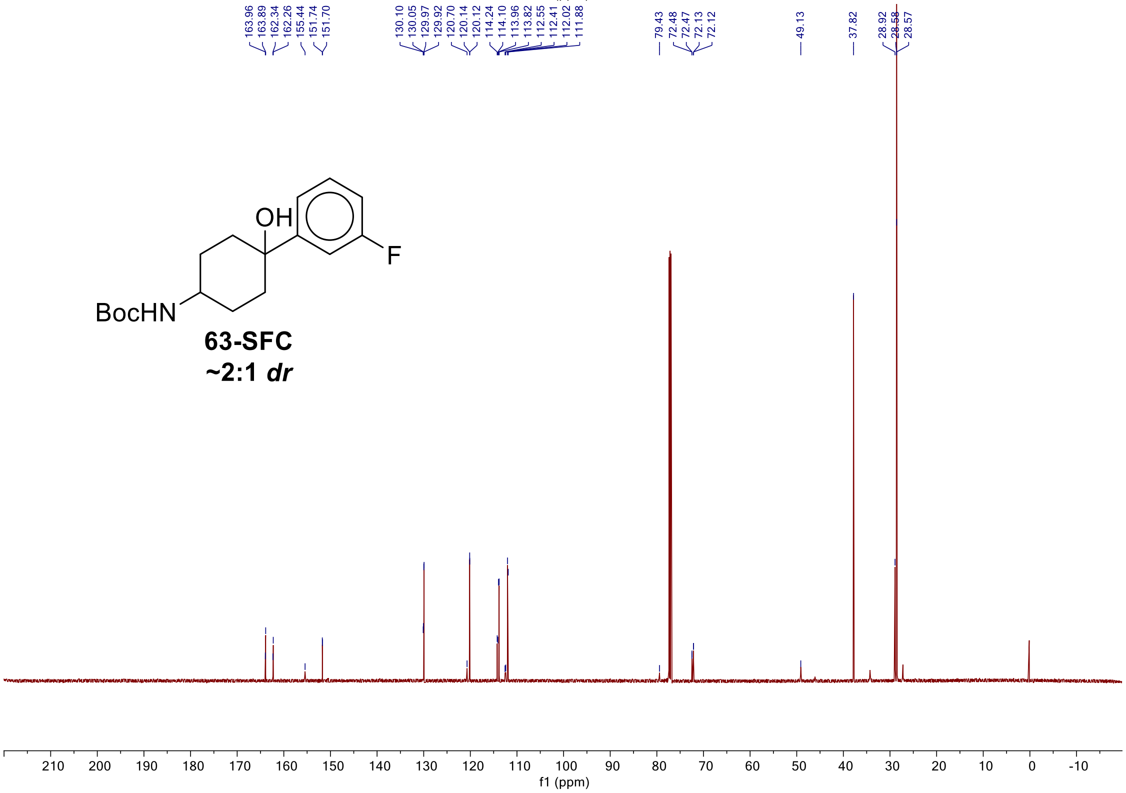
163.96
163.89
162.34
162.26
155.44
151.74
151.70
130.10
130.05
129.97
129.92
120.70
120.14
120.12
114.24
114.10
113.96
113.82
112.55
112.41
112.02
111.88
79.43
72.48
72.47
72.13
72.12
49.13
37.82
28.92
28.58
28.57
63-SFC
~2:1 dr
f1 (ppm)

19F NMR (565 MHz, $CDCl_3$)

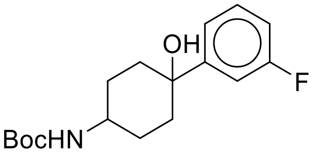

63-SFC
~2:1 *dr*

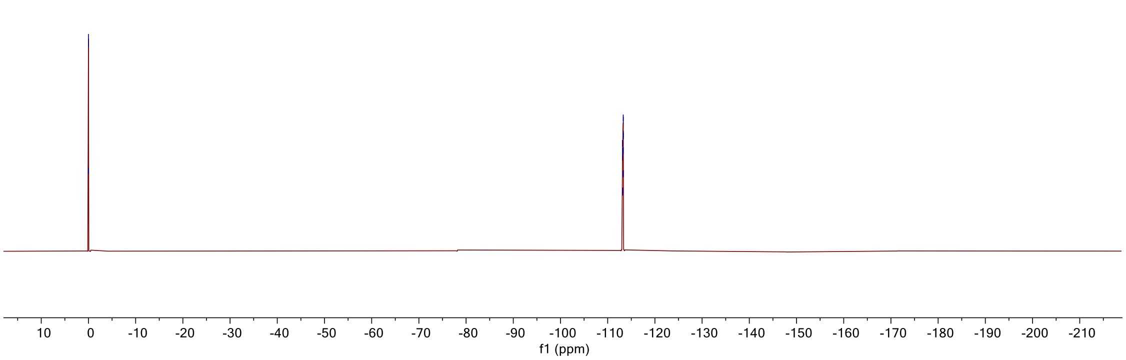

1H NMR (600 MHz, CDCl3)

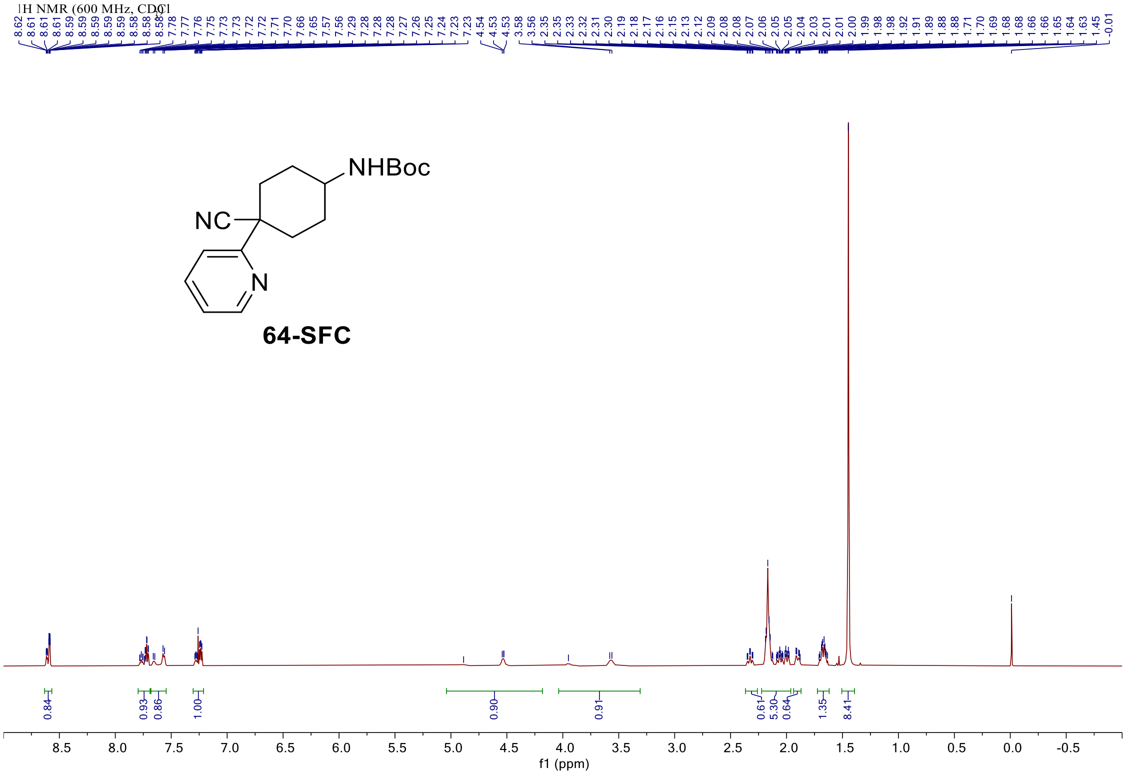

13C NMR (151 MHz, CDCl3)

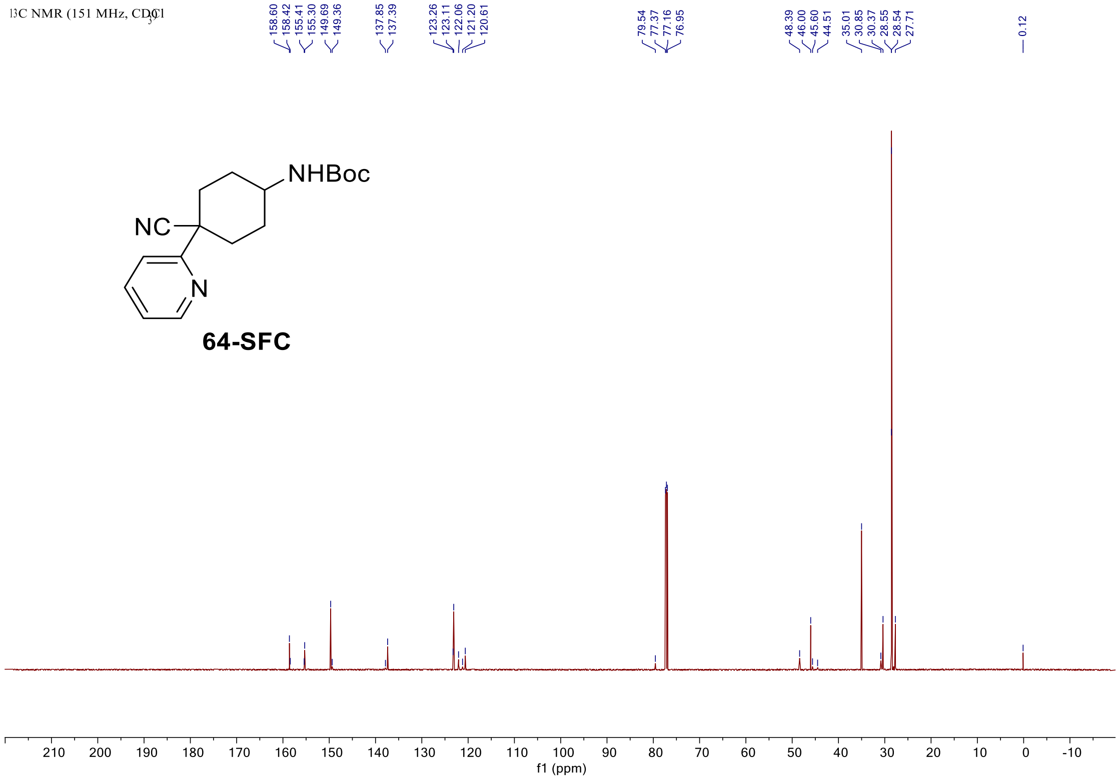

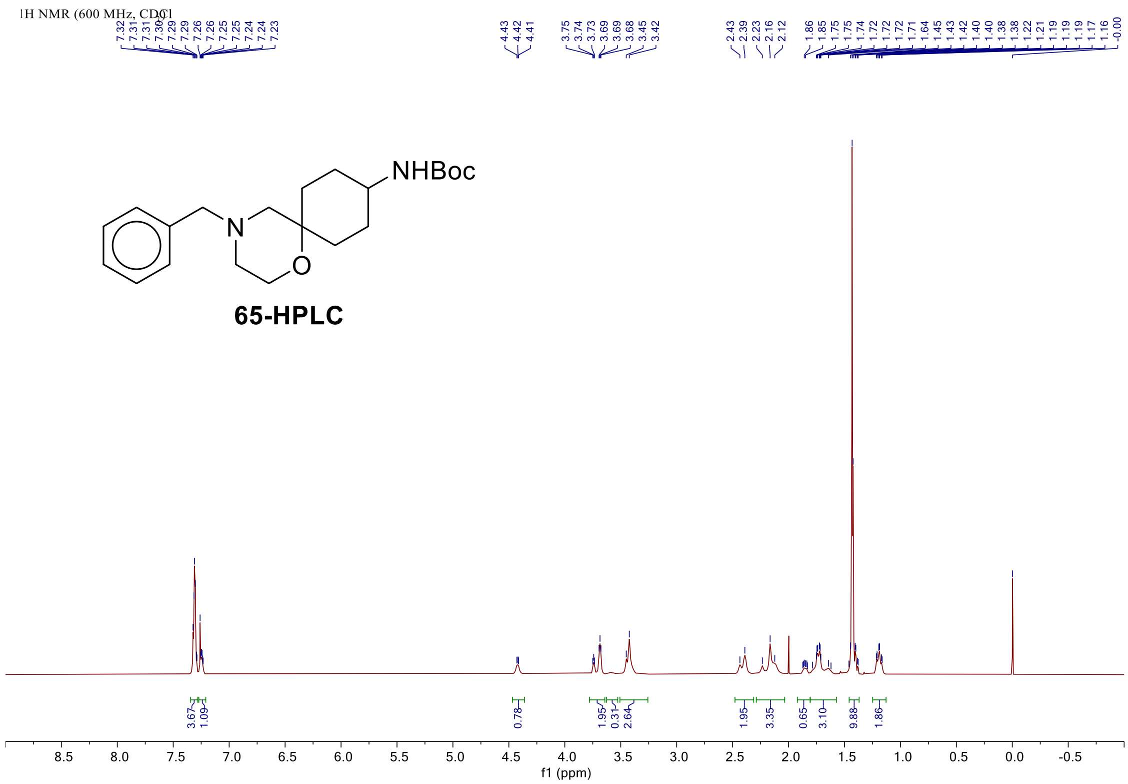

65-HPLC

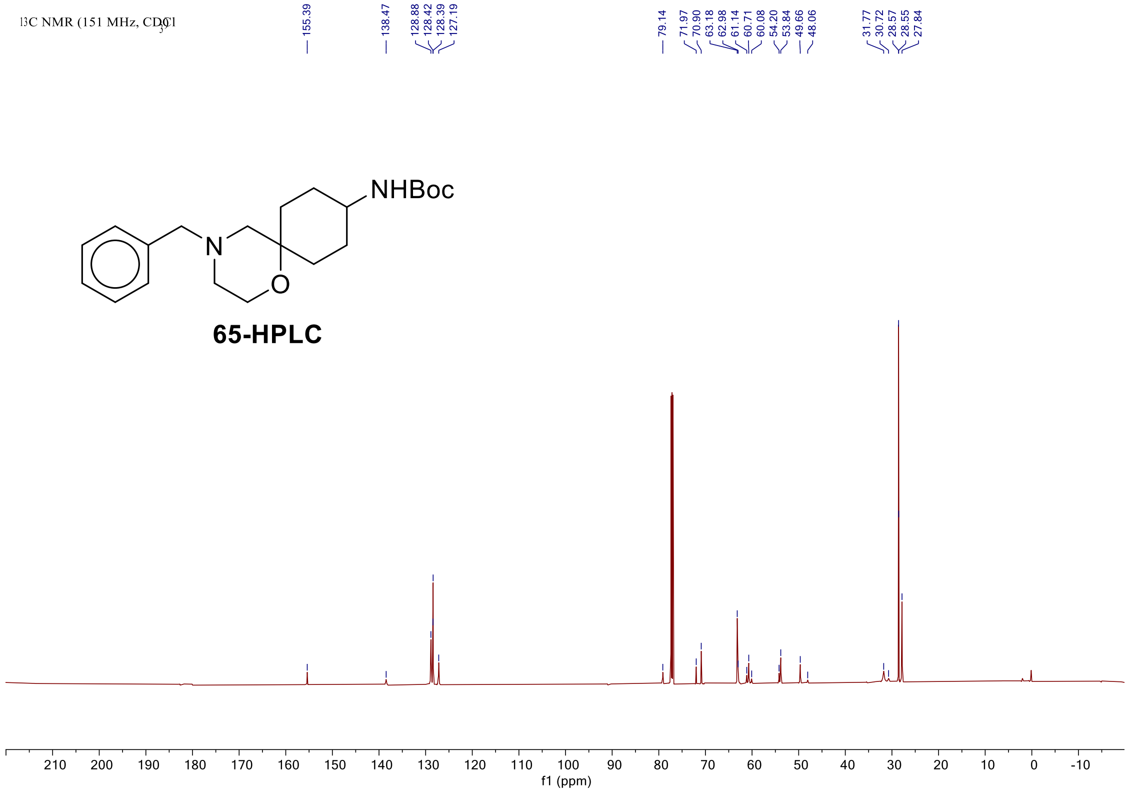

65-HPLC

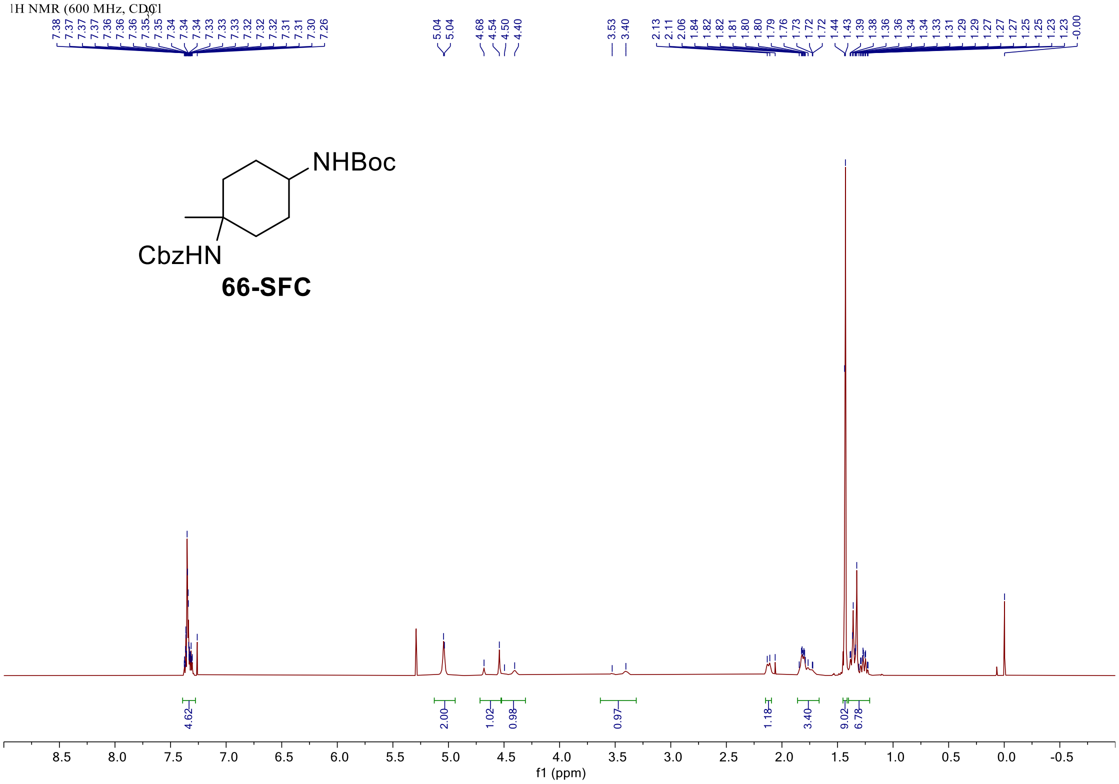
1H NMR (600 MHz, CDCl3)
NHBoc
CbzHN
66-SFC
f1 (ppm)

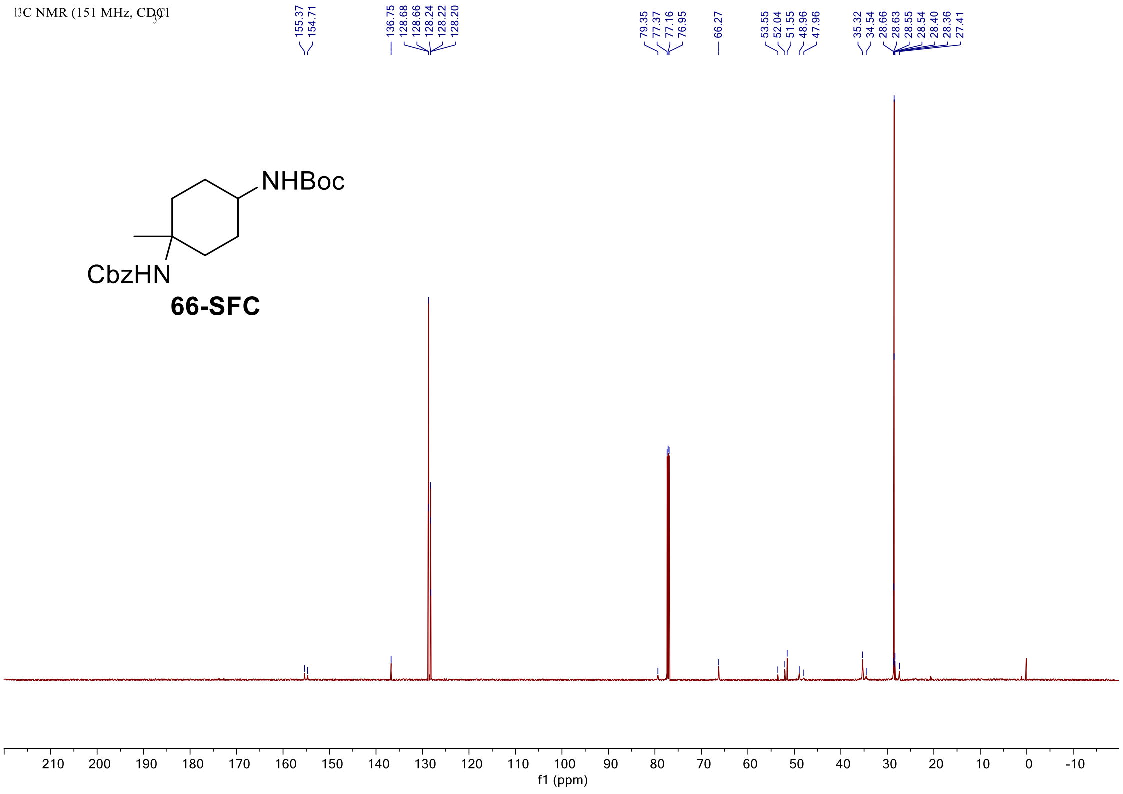
13C NMR (151 MHz, CDCl3)
NHBoc
CbzHN
66-SFC
f1 (ppm)

1H NMR (600 MHz, CDCl3)

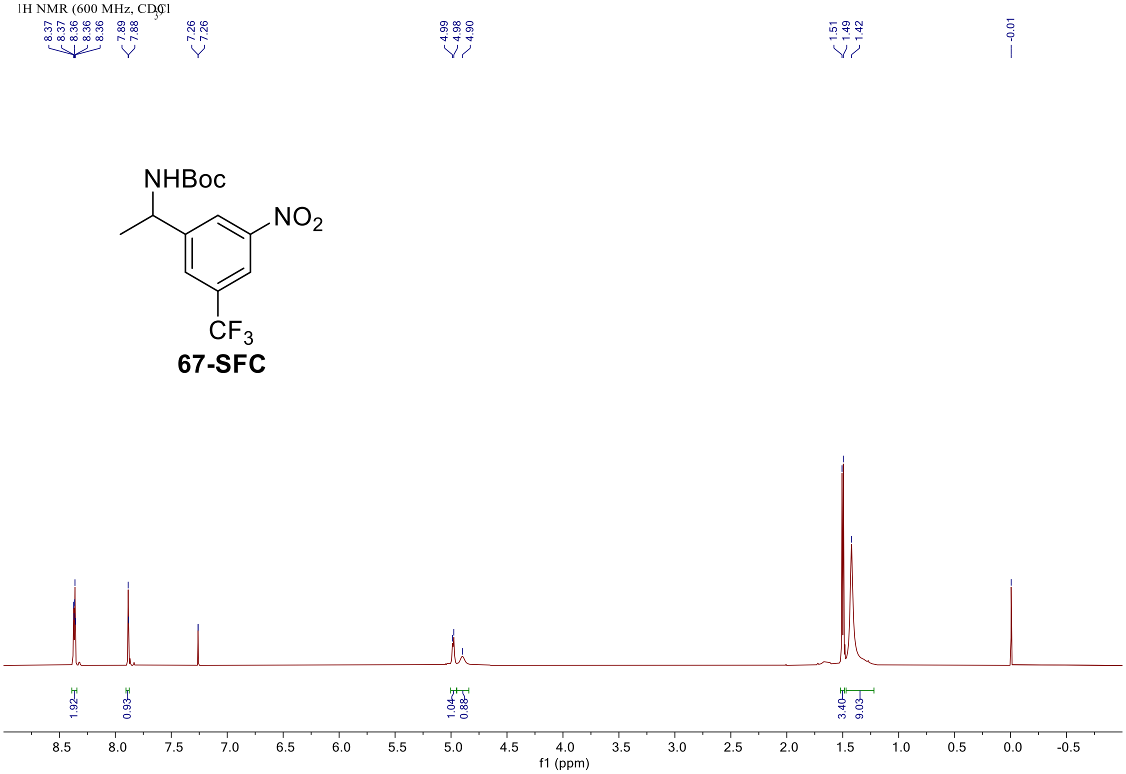

67-SFC

13C NMR (151 MHz, CDCl3)

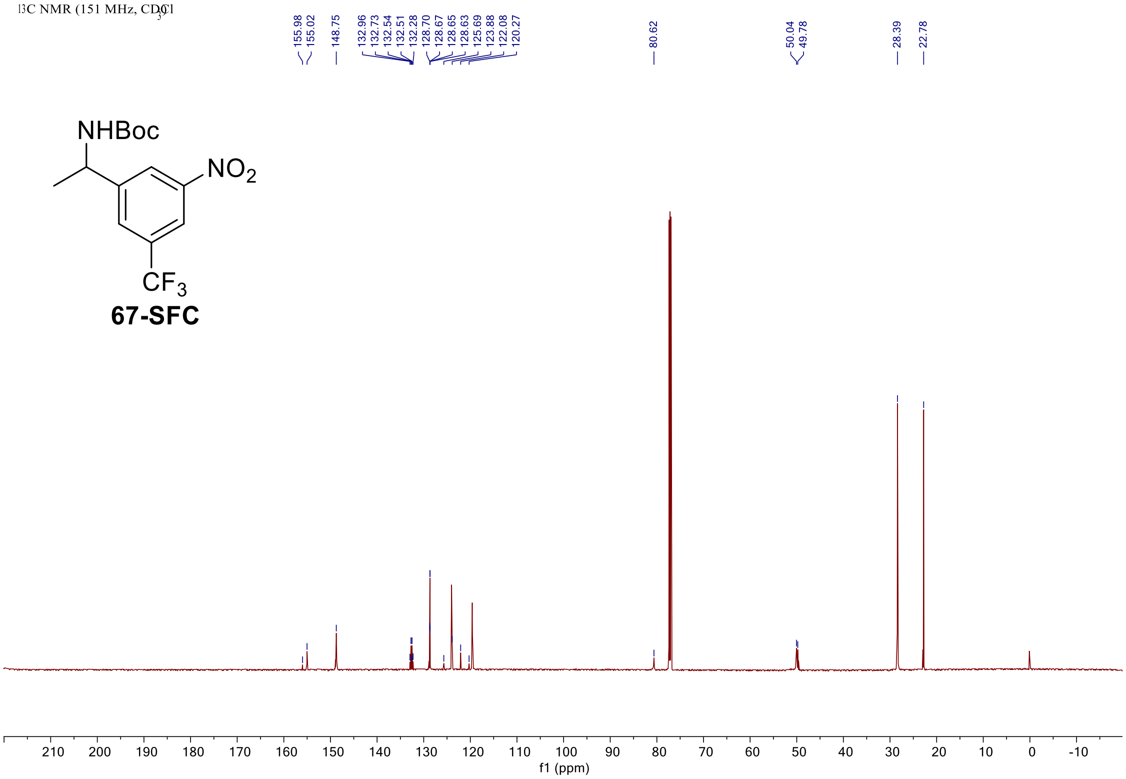

67-SFC

19F NMR (376 MHz, CDCl3)

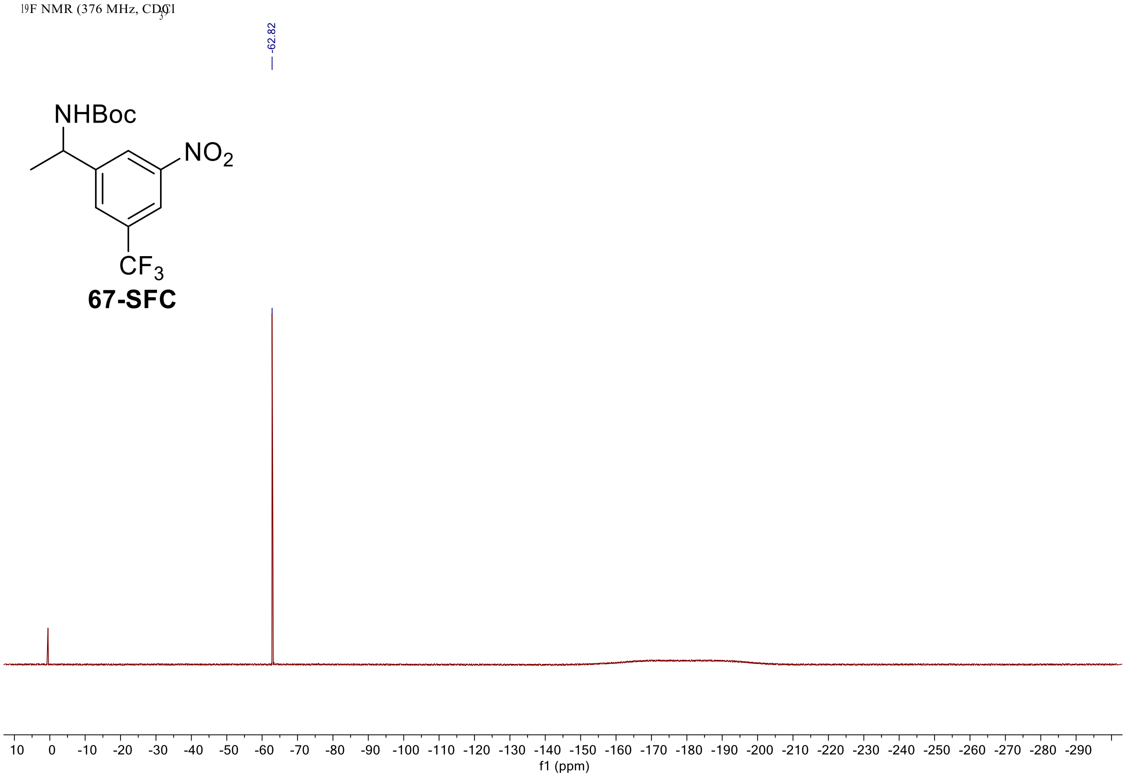

1H NMR (499 MHz, CDCl3)

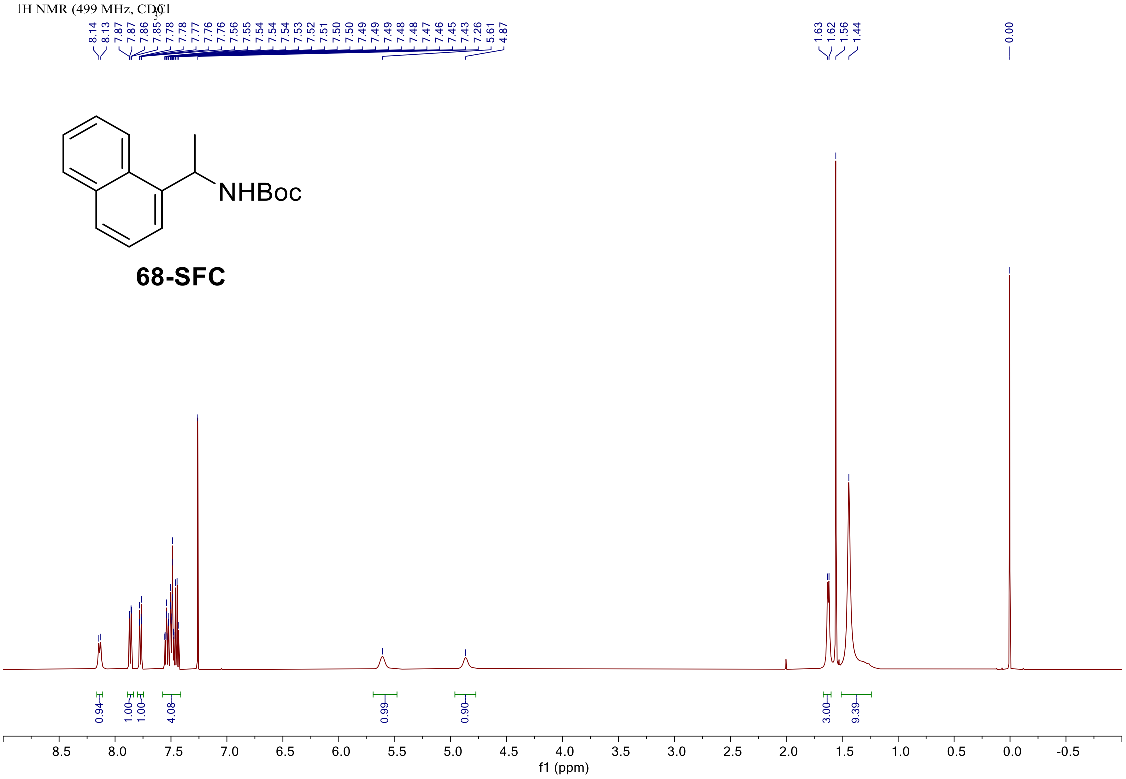

1H NMR (600 MHz, $CDCl_3$

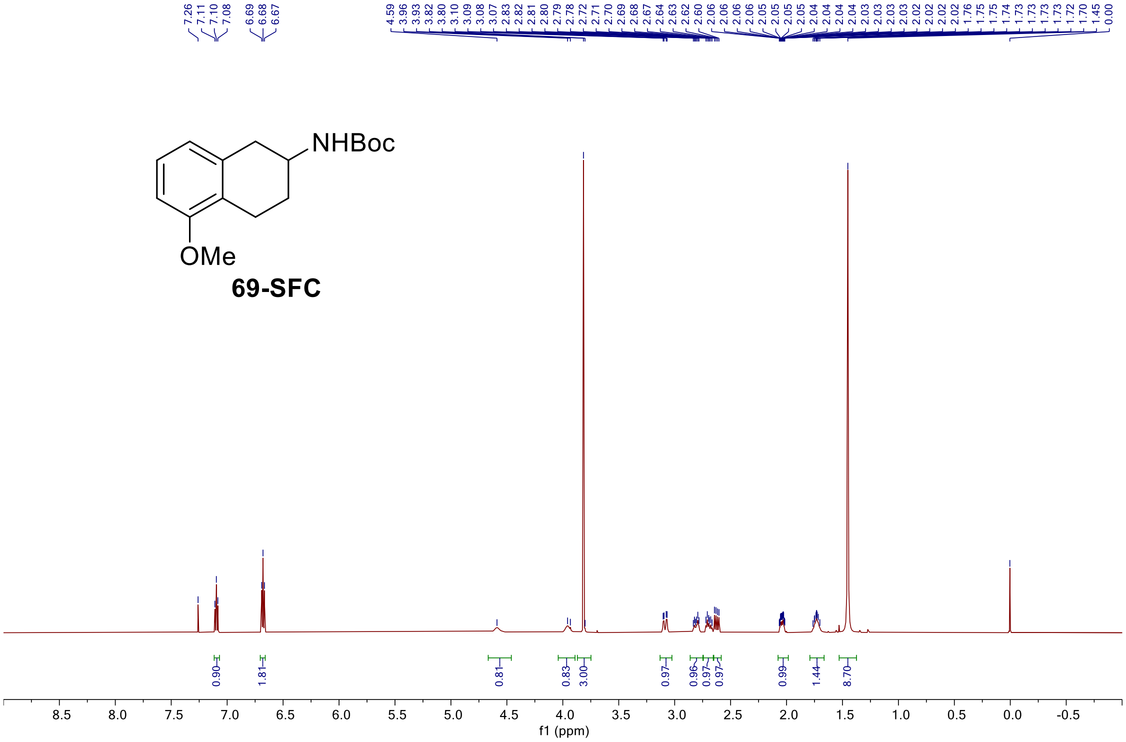

13C NMR (151 MHz, $CDCl_3$

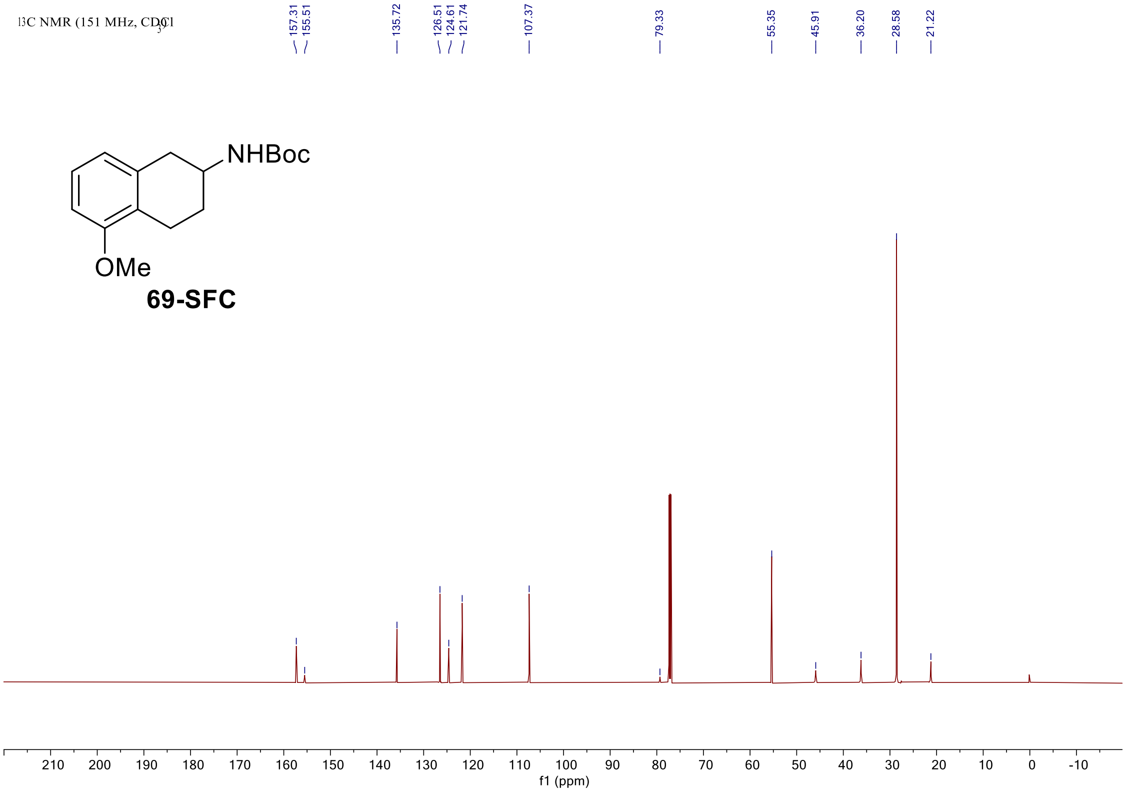

Supplement: SC-017-D6SC00852F-s001 [file SC-017-D6SC00852F-s001.pdf]
